# Biomimetic Frustrated Lewis Pair Catalysts for Hydrogenation of CO to Methanol at Low Temperatures

**DOI:** 10.1021/acsorginorgau.3c00064

**Published:** 2024-01-31

**Authors:** Jiejing Zhang, Longfei Li, Xiaofeng Xie, Xue-Qing Song, Henry F. Schaefer

**Affiliations:** † College of Pharmacy, Key Laboratory of Pharmaceutical Quality Control of Hebei Province, Key Laboratory of Medicinal Chemistry and Molecular Diagnosis of Ministry of Education, Hebei University, Baoding 071002, Hebei, P. R. China; ‡ Center for Computational Quantum Chemistry, University of Georgia, Athens, Georgia 30602, United States

**Keywords:** CO hydrogenation, biomimetic FLP catalysts, density functional theory, 1,1-addition, base-free

## Abstract

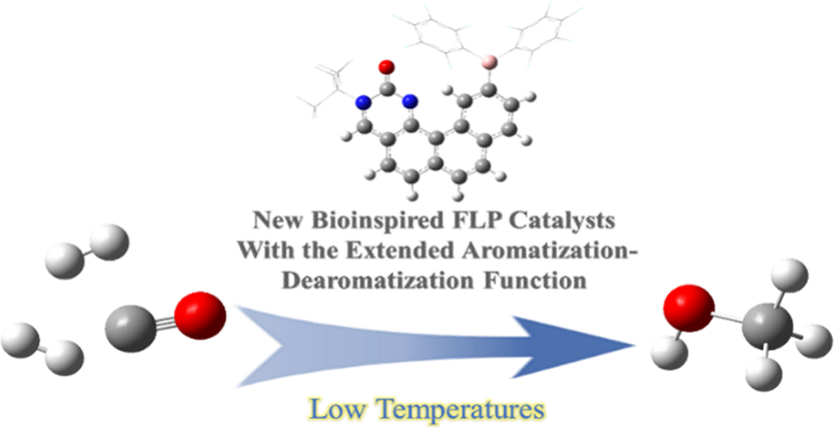

The industrial production of methanol through CO hydrogenation using the Cu/ZnO/Al_2_O_3_ catalyst requires harsh conditions, and the development of new catalysts with low operating temperatures is highly desirable. In this study, organic biomimetic FLP catalysts with good tolerance to CO poison are theoretically designed. The base-free catalytic reaction contains the 1,1-addition of CO into a formic acid intermediate and the hydrogenation of the formic acid intermediate into methanol. Low-energy spans (25.6, 22.1, and 20.6 kcal/mol) are achieved, indicating that CO can be hydrogenated into methanol at low temperatures. The new extended aromatization**–**dearomatization effect involving multiple rings is proposed to effectively facilitate the rate-determining CO 1,1-addition step, and a new CO activation model is proposed for organic catalysts.

## 1 Introduction

**Scheme 1:**
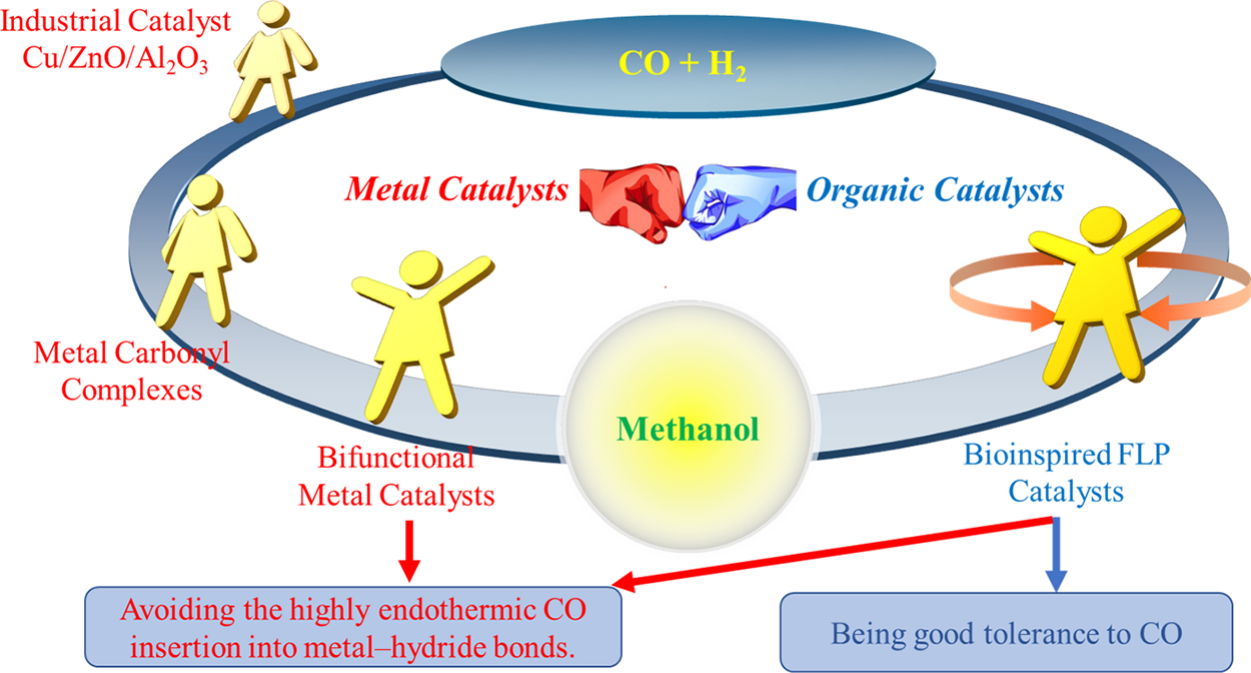
Catalysts for the Hydrogenation of CO to Methanol

Alternative energy sources have received great attention due to the limited supply and increasing cost of fossil fuel resources.^1,2^ Methanol is an excellent fuel in its own right and can also produce energy in electrochemical cells or other energy vectors such as hydrogen.^3−5^ Besides, methanol is a key feedstock for the production of small-chain hydrocarbons.^6,7^ Olah and co-workers previously advocated “methanol economy” as a way toward the sustainable economy, in which petroleum-based fuels and chemicals are replaced with methanol.^8^ However, methanol is industrially produced from CO hydrogenation using Cu/ZnO/Al_2_O_3_ heterogeneous catalysts at elevated pressures (50–100 bar) and temperatures (200–300 °C).^9,10^ Since the hydrogenation of CO to methanol is an exothermic reversible reaction, CO conversion in current processes is thermodynamically limited to around 30% at such high operating temperatures.^11^ Therefore, the development of homogeneous catalysts for CO hydrogenation at low temperatures would be a significant progress toward increasing the energy efficiency and CO conversions.^12^ The main challenges are the high endothermic migratory insertion process of CO into metal hydride bonds and the poisons of CO to metal catalysts (Scheme 1).

The reported homogeneous CO hydrogenations rely on metal catalysts, and the mechanisms can be broadly divided into direct and indirect mechanisms. In the early 1950s, DuPont Company first reported the direct hydrogenation of CO to methanol catalyzed by cobalt carbonyl complexes, but the reaction required ultrahigh pressures (1500–5000 atm).^13^ In the following three decades, the direct hydrogenation of CO to methanol catalyzed by various transition-metal carbonyls has been reported, but the problem of extreme reaction conditions was not solved.^14−20^ In general, CO is a high-field ligand, and its migratory insertion into a metal–hydride bond is highly endothermic, which is responsible for the harsh reaction conditions.^21,22^ Instead, avoiding this highly endothermic CO insertion step could allow mild reaction conditions for the synthesis of methanol.^23^

**Scheme 2:**
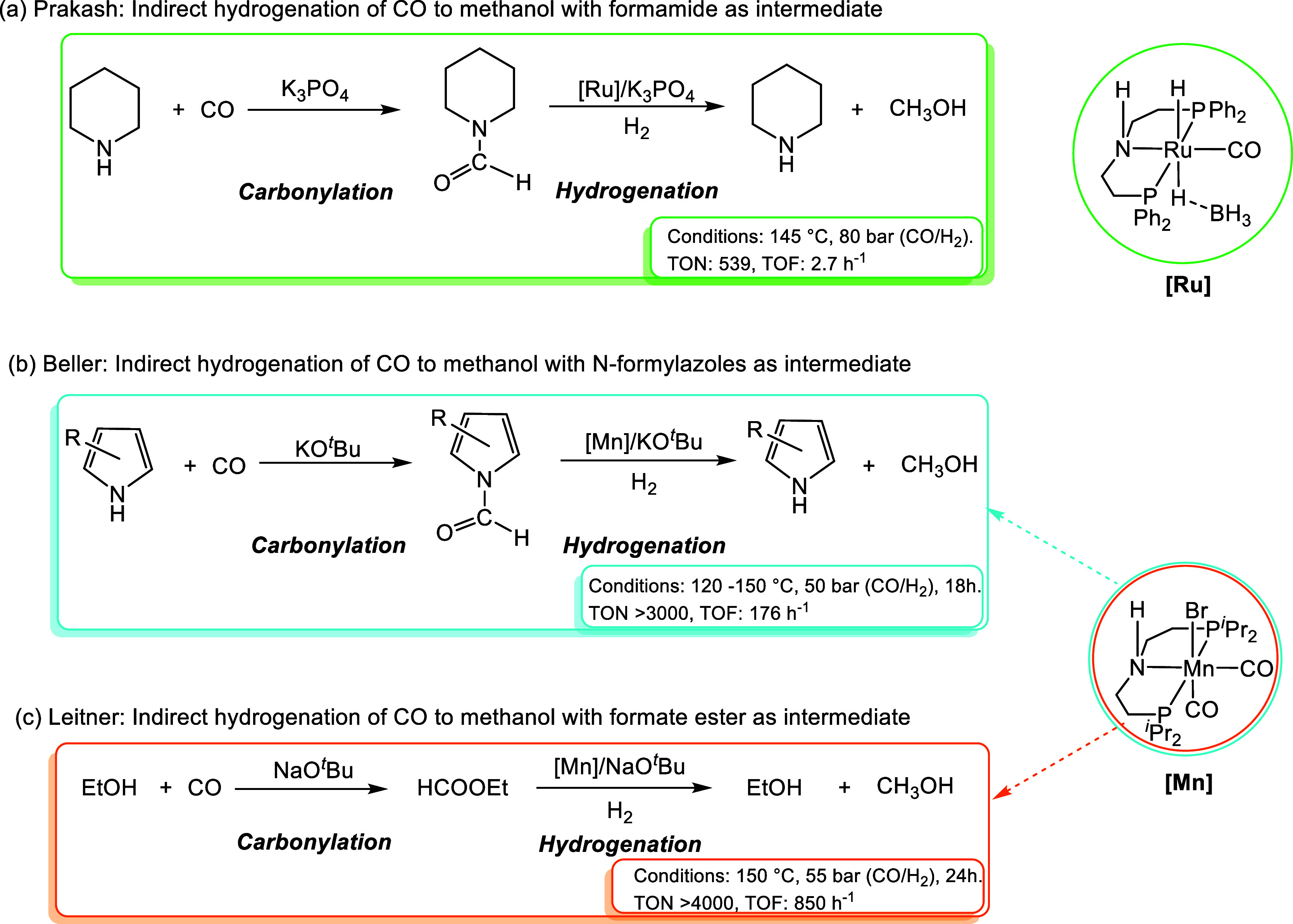
Reported Bifunctional Metal Catalysts for the Indirect Hydrogenation of CO to Methanol

Recently, bifunctional metal catalysts have been reported to catalyze the hydrogenolysis of esters, amides, and their derivatives to produce alcohols.^24−29^ Those research studies inspired the design of bifunctional metal catalysts for the indirect hydrogenation of CO to methanol via ester or amide intermediates. In 2019, Prakash and co-workers found that electron-withdrawing phenyl groups could render the Ru–Macho–BH complex resistant to CO poisoning and achieved the Ru–Macho–BH catalyzed indirect hydrogenation of CO to methanol at 145 °C with a turnover number (TON) of 539 (Scheme 2a).^12^ Prakash also proposed that CO was first anchored onto the amine as a formamide and then hydrogenated in situ to methanol. In the same year, Beller and co-workers investigated a series of metal complexes for the indirect hydrogenation of CO to methanol through N-formylazole intermediates, and the manganese pincer complex exhibited the best tolerance to CO and gave the optimal TON (3170) at 120–150 °C (Scheme 2b).^30^ In 2021, Leitner and co-workers reported an alcohol-assisted indirect hydrogenation of CO to methanol catalyzed by a manganese pincer complex with a catalytic amount of base at 150 °C (Scheme 2c).^31^ They found that the increased pressure of CO could inhibit methanol formation. These remarkable bifunctional metal catalysts facilitate indirect CO hydrogenation but are still limited by high reaction temperatures and low yields, which could be associated with the deactivation of transition-metal catalysts in the presence of CO.^32^

Main-group element catalysts are desirable alternatives to transition-metal catalysts because of their natural abundance and low cost.^33,34^ More importantly, main-group element catalysts have low binding affinity to CO, owing to the lack of suitable π-back-bonding orbitals.^35^ This implies good tolerance to CO and inspires us to explore main-group element catalysts for the indirect hydrogenation of CO to methanol. Frustrated Lewis pairs (FLPs) introduced by Stephan and co-workers are now an important branch of main-group element chemistry^36,37^ and have been applied in the catalytic hydrogenation of various unsaturated compounds including CO_2_,^38^ olefins,^39^ alkynes,^40^ ketones,^41^ and imines.^42^ Particularly, the FLP-catalyzed hydrogenation of amides^43^ and esters^44,45^ to provide alcohols was also reported recently. However, FLP-catalyzed CO hydrogenation has not been reported yet. How can we endow FLP catalysts with high activities toward CO hydrogenations?

**Scheme 3:**
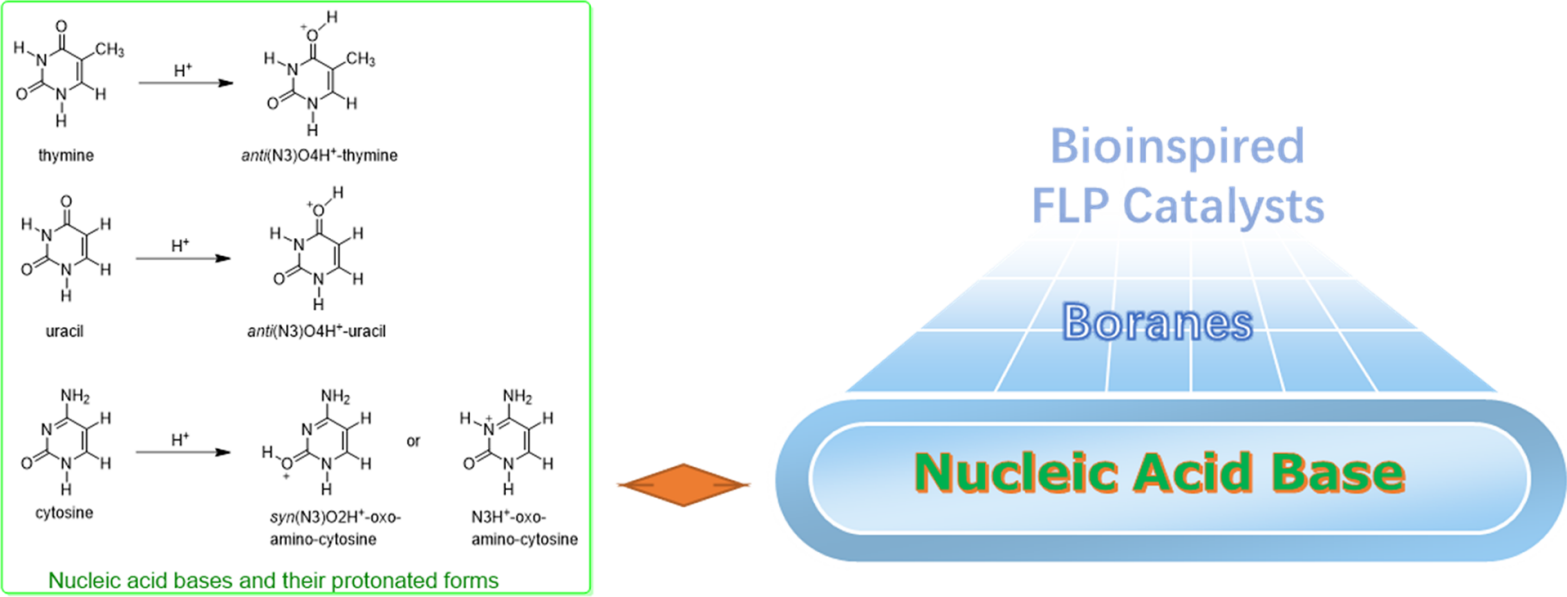
Nucleic Acid Bases and Bioinspired FLP Catalysts

The design of novel bioinspired catalysts mimicking the mechanism of biocatalysts is an important strategy to obtain high catalytic activity, yield, and selectivity.^46−49^ The nucleic acid bases (Scheme 3) with resonance stabilization effects can undergo protonation or tautomerization^50−53^ and play an important role in the reproduction and transmission of genetic information.^54^ Can the Lewis basic nucleic acid bases form novel FLP catalysts with borane counterparts and achieve new chemical reactivities? The theoretical design plays a more and more important role in discovering new chemistry, and many achievements have been made to solve important problems in chemistry.^55−60^ Inspired by our and other groups’ silico reaction discoveries,^61−67^ we theoretically design bioinspired FLP catalysts based on nucleic acid bases and compare their catalytic activities in the CO hydrogenation reaction to methanol with the traditional FLP catalysts.

## 2 Computational Methods

In accord with our previous theoretical studies,^68−70^ this research was carried out with the DFT *ω*B97X-D^71^ method using the Gaussian 09 program.^72^ Geometries were optimized in a dichloromethane solvent using the 6-311G(d,p)^73^ basis sets. The single-point energy refinements were further performed with the 6-311++G(2d,p) basis sets. The refined energies were then corrected to Gibbs energies at 298.15 K and 1 atm by using the *ω*B97X-D/6-311G(d,p) harmonic frequencies. Solvent effects were evaluated using the SMD (solution model based on density) solvation model.^74,75^ All transition states were demonstrated to exhibit only one imaginary vibrational frequency. Intrinsic reaction coordinate (IRC) analyses were performed to confirm that all transition states connect the two minima in question.^76^ Natural bond orbital (NBO) analyses were performed using the NBO-7.0 program.^77^ The Cartesian coordinates of all optimized structures are presented in the Supporting Information.

## 3 Results and Discussion

**Figure 1:**
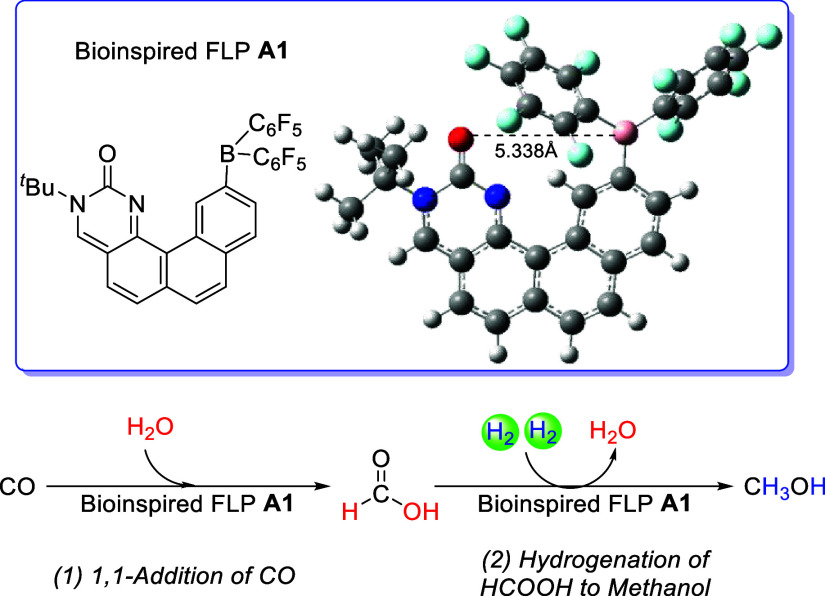
Structure of bioinspired FLP **A1** and the catalyzed hydrogenation of CO to methanol.

The bioinspired FLP **A1** featuring a nucleic acid base-fused π-conjugated linker is designed, and the corresponding structure is shown in Figure 1. The distance between the Lewis acidic B atom and the Lewis basic carbonylic O atom is large (5.34 Å), which provides a suitable pocket for the synchronous activation of two substrate molecules (e.g., water and CO). The **A1-**catalyzed CO hydrogenation to methanol is designed and is composed of two stages: (1) the 1,1-addition of CO into formic acid and (2) the hydrogenation of formic acid into methanol.

### 3.1 1,1-Addition of CO to the Formic Acid Intermediate

**Figure 2:**
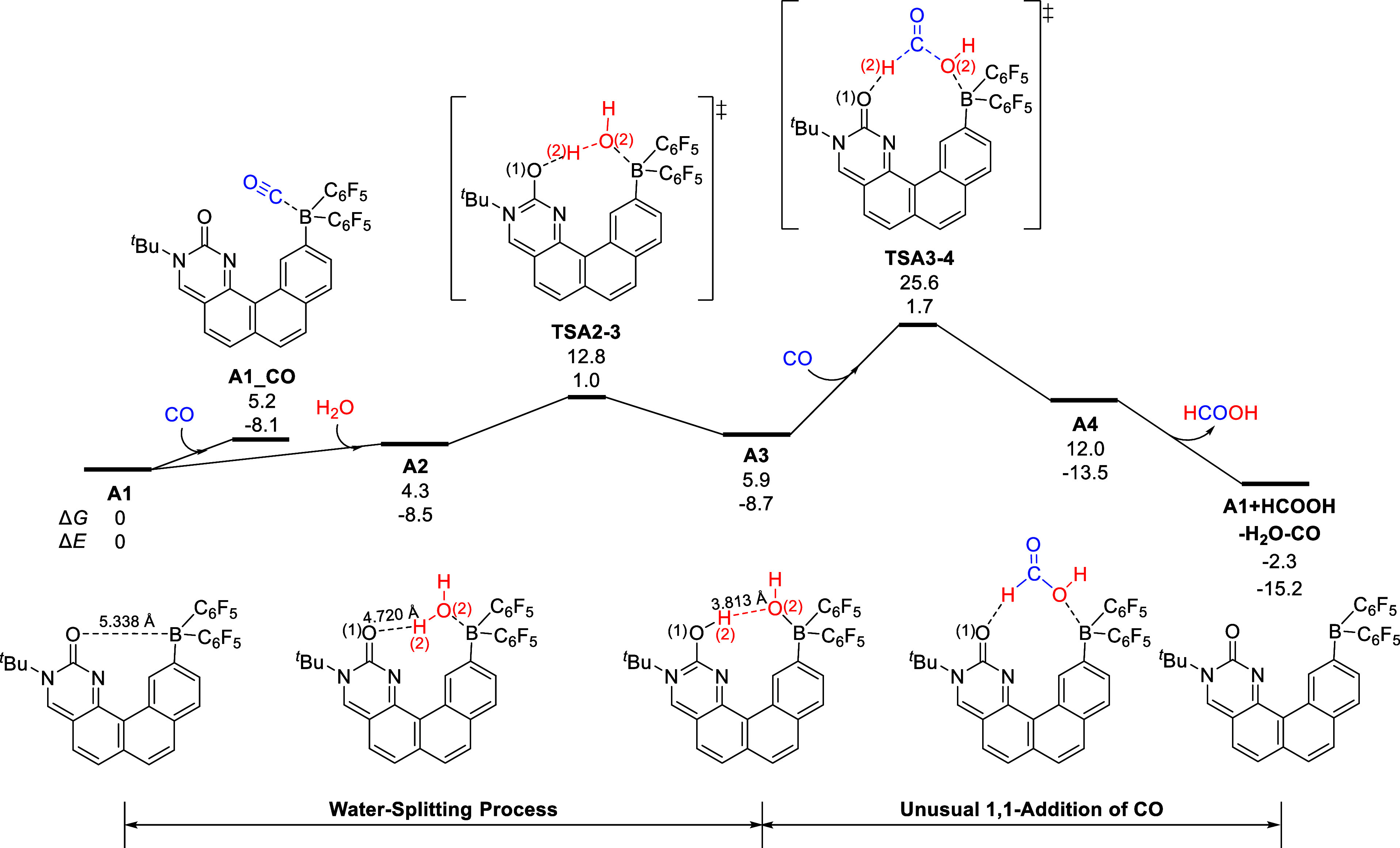
Gibbs energy profile for the 1,1-addition of CO to a formic acid intermediate catalyzed by the bioinspired FLP **A1**. The relative Gibbs energies (Δ*G*) and potential energies (Δ*E*) are in kcal/mol.

**Figure 3:**
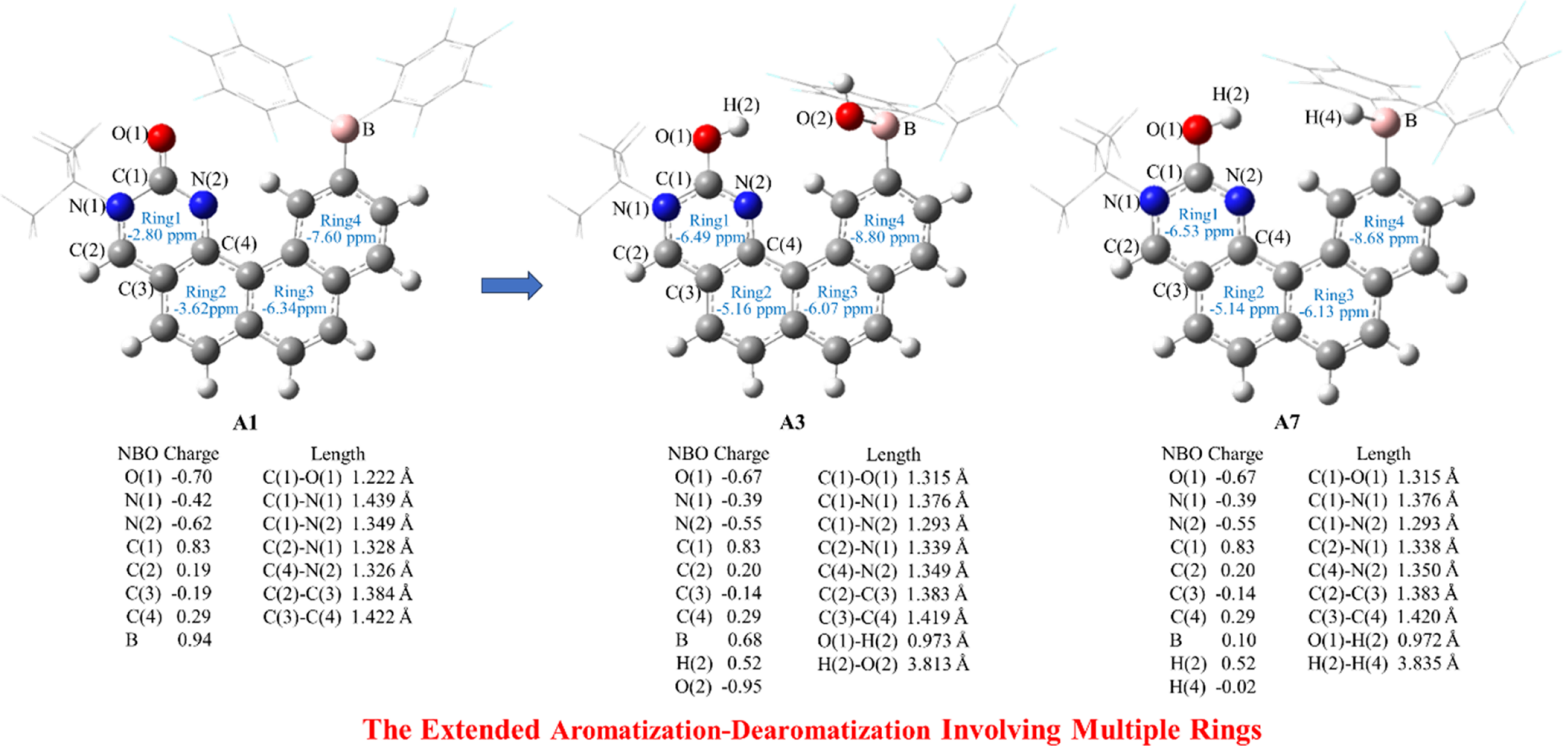
Atomic charges, bond lengths, and the nucleus-independent chemical shift (NICS) values of the rings in **A1**, **A3,** and **A7**. The −^*t*^Bu and −C_6_F_5_ groups are drawn in a wireframe for simplicity.

The bioinspired FLP **A1** features good tolerance to CO, and the coordination of CO to **A1** will afford a thermodynamically unstable intermediate **A1_CO**, being endergonic by 5.2 kcal/mol as shown in Figure 2. The dimerization of **A1** through forming a Lewis adduct is thermodynamically disfavored, being endergonic by 2.4 kcal/mol (Figure S2), suggesting that the monomer of **A1** is the major species in solution. The B atom of **A1** is Lewis acidic with an NBO charge of 0.94 and can bind a H_2_O molecule through the O → B donor–acceptor interaction, being endergonic by 4.3 kcal/mol. Through the transition state **TSA2–3**, the O–H bond of the coordinated H_2_O molecule is cleaved, and the hydroxy proton H(2) moves to the adjacent carbonylic O atom with an energy barrier of 8.5 kcal/mol. Subsequently, the zwitterionic proton**–**hydroxo intermediate **A3** is formed, in which the C(1)–N(1) and C(1)–N(2) bonds are significantly shortened compared with those in **A1**. The nucleus-independent chemical shift (NICS) method is widely utilized for determining aromaticity.^78^ The NICS value of the pyrimidinol ring in **A3** (ring 1; −6.49 ppm) is much more negative than that of the pyrimidinone ring in **A1** (ring 1; −2.80 ppm), suggesting that the pyrimidinol ring in **A3** has become aromatized. As for ring 2 and ring 4 in **A3**, the NICS values become more negative by 1.54 and 1.20 ppm, respectively; therefore, the protonation of the carbonylic O atom also renders the remote ring 2 and ring 4 more aromatic. The NICS value of ring 3 in **A3** increases a bit (0.27 ppm) (Figure 3). The extended aromatization**–**dearomatization effect involving multiple rings was not reported previously.

The proton and hydroxy moieties in zwitterionic **A3** can be added to CO through an unusual 1,1-addition process. The H(2)^**...**^O(2) distance in **A3** is 3.81 Å, and the O(2) atom is nucleophilic with an NBO charge of −0.95. After the transition state **TSA3–4**, proton H(2) and hydroxo transfer to the carbonylic C atom of CO, simultaneously. The 1,1-addition of CO also involves the extended aromatization**–**dearomatization effect, and intermediate **A4** with a dearomatized pyrimidinone ring is formed. The following dissociation of the formic acid molecule from **A4** will regenerate catalyst **A1**, and the stage of CO 1,1-addition is achieved. Including the energy profile of Stage 2 included in Section 3.2, the 1,1-addition of CO via **TSA3–4** is the rate-determining step of the overall catalytic reaction with an energy span of 25.6 kcal/mol. Other promoters are also considered for the 1,1-addition of CO in Figure S3 in SI, and the methanol promoter under catalyst **A1** is predicted to own a high-energy barrier of 28.1 kcal/mol.

**Figure 4:**
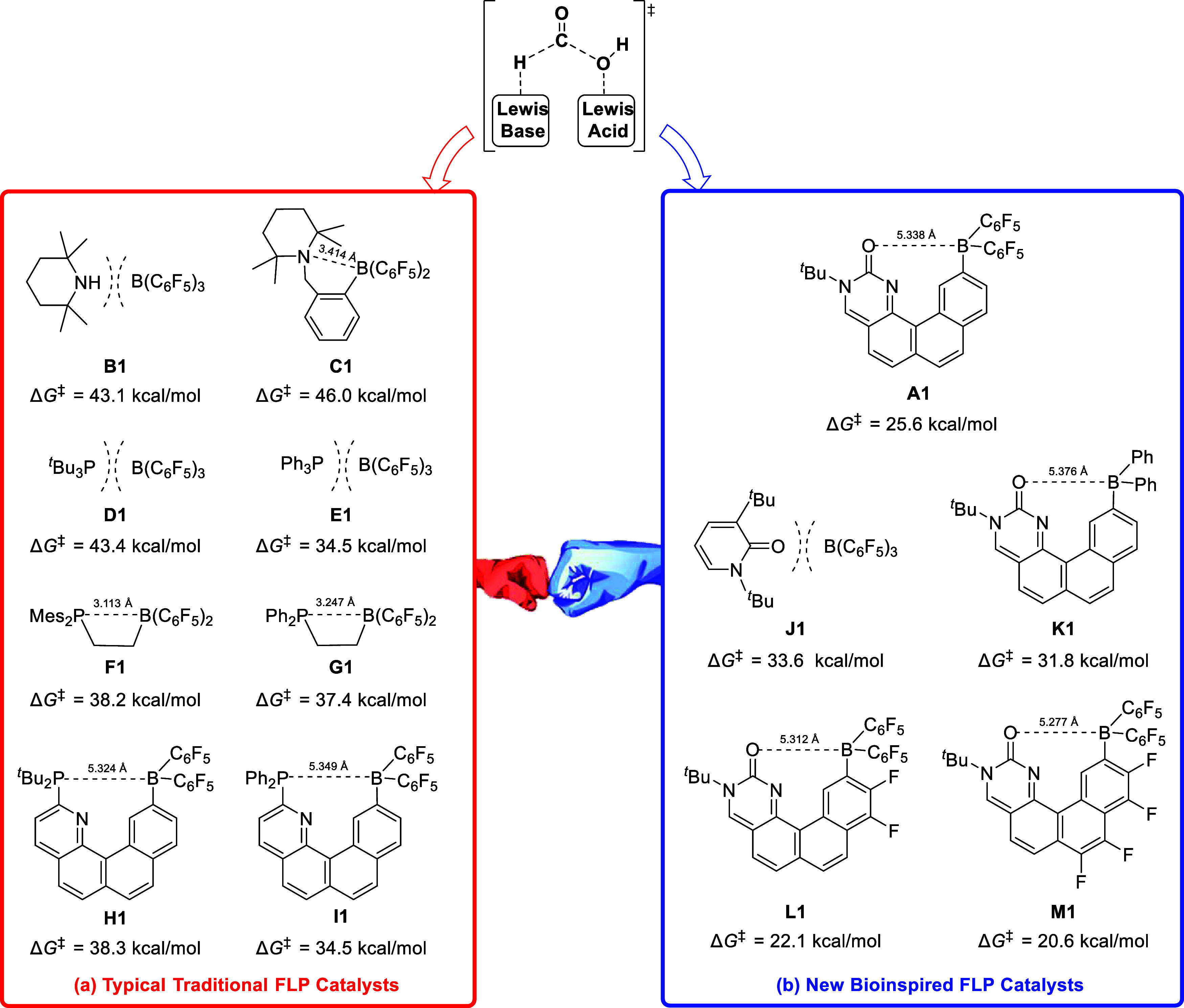
Comparisons between the typical traditional FLP catalysts and the new bioinspired FLP catalysts via the Gibbs energy barriers (Δ*G*^‡^) of the rate-determining CO 1,1-addition.

The acceleration effects of the extended aromatization**–**dearomatization of our designed bioinspired FLP catalysts are studied. The typical traditional catalysts and the designed bioinspired FLP catalysts are compared to the Gibbs energy barriers (Δ*G*^‡^) of the rate-determining CO 1,1-addition steps via **TSA3–4**. The corresponding whole catalytic pathways are shown in Figures S6–S17 in the SI. The typical traditional FLP catalysts including intermolecular and intramolecular systems are predicted to involve high-energy barriers (34–46 kcal/mol; Figure 4a). It is worth noting that the traditional FLPs **H1** and **I1** have the similar linker structure with the designed bioinspired FLP **A1**, and the distances of Lewis acid–base sites are almost the same for **H1**, **I1,** and **A1** (ca. 5.3 Å). Differently, **H1** and **I1** cannot exhibit the extended aromatization**–**dearomatization effect. Consequently, the energy barriers of **H1** and **I1** catalysts (38.3 and 34.5 kcal/mol) are predicted to be much higher than that of the bioinspired FLP **A1** catalysts (25.6 kcal/mol), suggesting that the extended aromatization**–**dearomatization in **A1** could play an important role in facilitating the CO 1,1-addition step.

Further, the activities of the bioinspired FLP catalysts can be controlled. As shown in Figure 4b, the intermolecular catalyst **J1** has a higher energy barrier of the rate-determining CO 1,1-addition steps than the intramolecular ones, which could be associated with the entropy penalty. The intramolecular bioinspired FLP catalyst **K1** with −Ph groups is predicted to own an energy barrier (31.8 kcal/mol) higher than that of **A1** with −B(C_6_F_5_)_2_ groups (25.6 kcal/mol), suggesting that the higher Lewis acidity of B atom will result in lower energy barriers. Pursuing this idea, we introduced more F substituents on the linkers, and the modified FLP catalysts **L1** and **M1** are predicted to yield even lower energy barriers (22.1 and 20.6 kcal/mol), which can allow for low operating temperatures.

**Figure 5:**
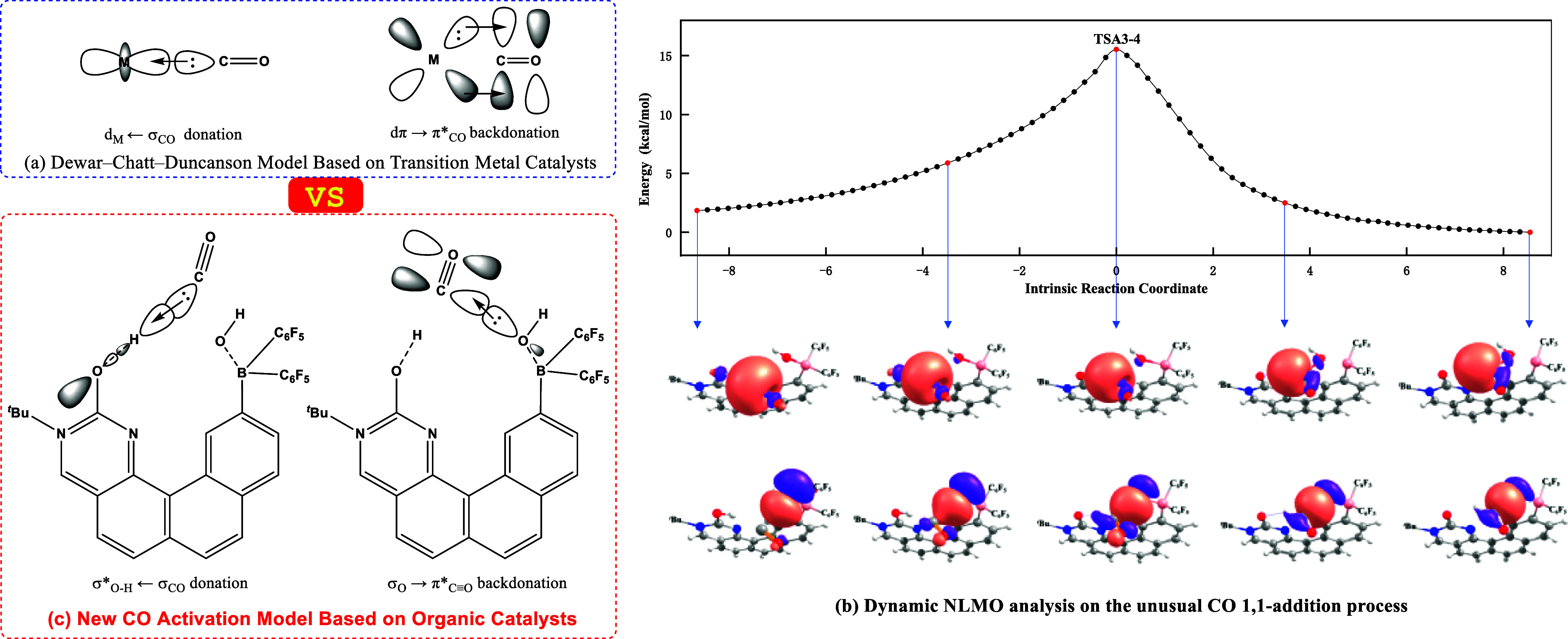
(a) Dewar**–**Chatt**–**Duncanson model for CO activation based on metal catalysts; (b) dynamic natural localized molecular orbital (NLMO) analysis for the unusual CO 1,1-addition process; and (c) new CO activation model based on organic catalysts.

The activation of CO by metal complexes is a highly important field in chemistry, and it has been explained in terms of the Dewar–Chatt–Duncanson model, which involves the σ-donation and dπ-back-donation interactions (Figure 5a).^79^ In contrast, the CO activation by organic catalysts does not involve dπ-back-donation, and it is still unclear. In order to unveil the reasons for CO activation by the bioinspired FLP, the unusual 1,1-addition of CO was simulated. This was accomplished via performing the intrinsic reaction coordinate (IRC) computation on the transition state **TSA3–4**. The natural localized molecular orbital (NLMO) analysis was applied to five frames of the IRC trajectory, as shown in Figure 5b. When the CO molecule approaches **A3**, the lone pair of the carbonyl carbon atom is delocalized to the σ*_O–H_ antibonding orbital, and the lone pair electron of the O(2) atom is delocalized to the π*_C≡O_ orbital. According to our dynamic NLMO analysis, a new CO activation model involving the σ-donation and σ-back-donation interactions is proposed, as illustrated in Figure 5c. This, hopefully, will guide the development of organic catalysts for CO hydrogenations.

### 3.2 Hydrogenation of Formic Acid Intermediate into Methanol

**Figure 6:**
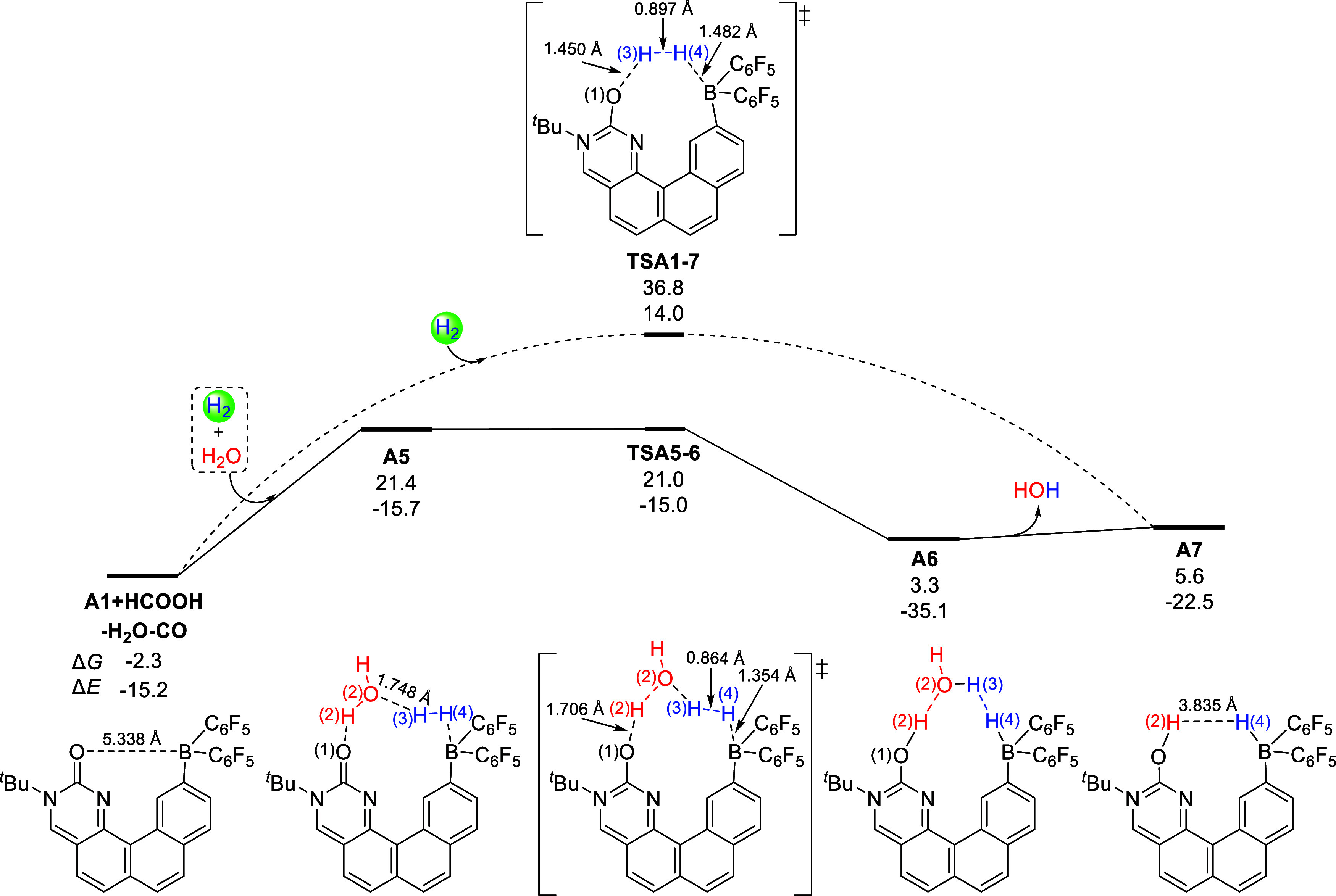
Hydrogen shuttle mechanism for hydrogen activation by bioinspired FLP **A1**. The relative Gibbs energies (Δ*G*) and potential energies (Δ*E*) are in kcal/mol.

The Lewis basic O and acidic B atoms in **A1** cannot form the effective orbital overlap with the hydrogen molecule due to the long O^**...**^B distance (5.34 Å) and make the common hydrogen activation pathway difficult. As shown in Figure 6, the hydrogen molecule cleaves across the B and O atoms of **A1** with a high energy barrier (39.1 kcal/mol), due to the high strain of the hydrogen-transfer transition state **TSA1–7**, indicating that this pathway is not available. Instead, a hydrogen shuttle mechanism becomes available for the bioinspired FLP-facilitated hydrogen activation. The water and hydrogen molecules can approach **A1** to form the complex **A5**, and this step is endothermic by 23.7 kcal/mol due to the entropy penalty. Through transition state **TSA5–6**, the H(3) atom moves from the hydrogen moiety to the nearby O(2) atom of the water moiety, which is accompanied by the H(2) atom transfer from the water moiety to the carbonylic O(1) atom. Actually, the water molecule serves as a hydrogen shuttle and decreases the energy barrier of the hydrogen activation to 23.3 kcal/mol. Note that although the Gibbs energy of **TSA5–6** (21.0 kcal/mol) appears to be lower than that of **A5** (21.4 kcal/mol), the potential energy of **TSA5–6** (−15.0 kcal/mol) is higher than that of **A5** (−15.7 kcal/mol). In the following, the intermediate **A6** is generated and can release a water molecule to form the hydrogenated intermediate **A7**. As shown in Figure 3, the bond lengths and NICS values of **A7** are almost the same with those in **A3**, suggesting that the hydrogen activation also experiences the extended aromatization**–**dearomatization process.

**Figure 7:**
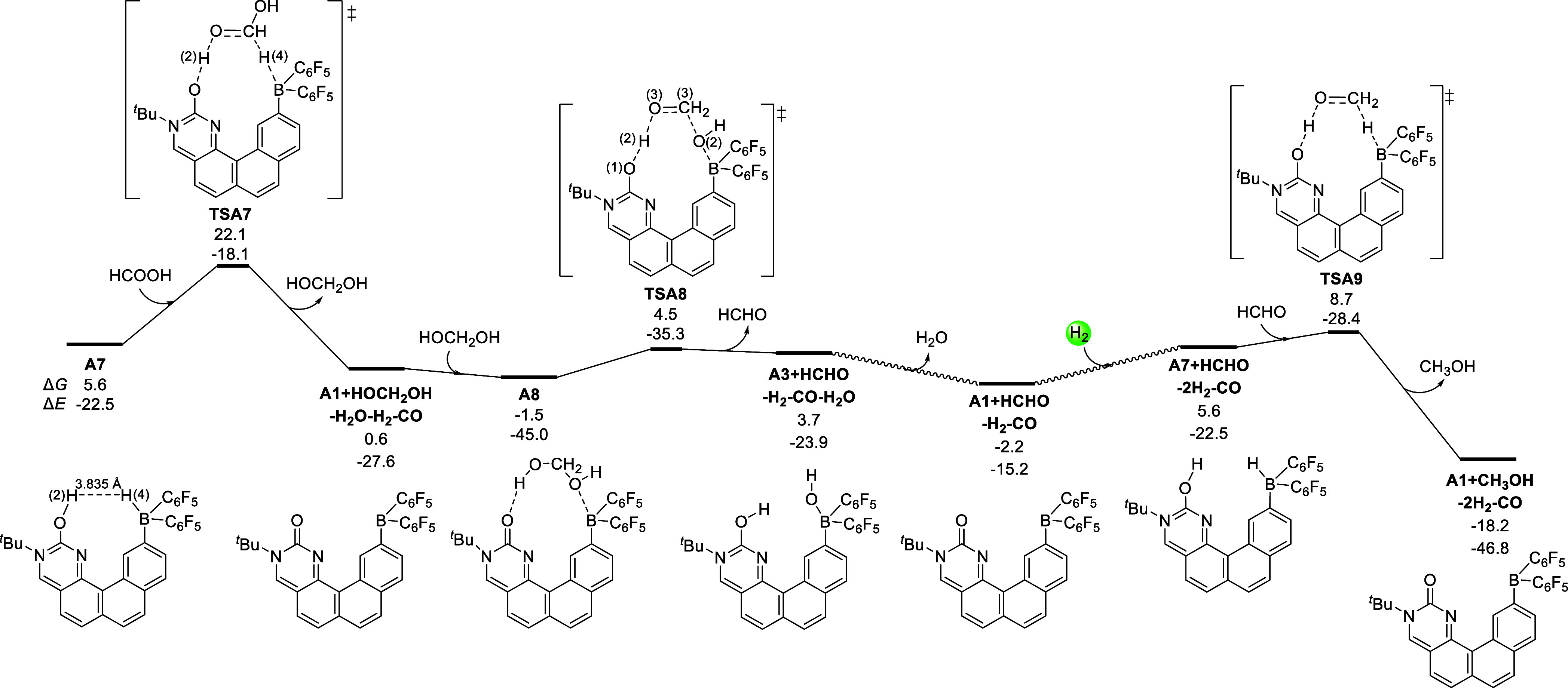
Gibbs energy profile for the hydrogenation of formic acid to methanol catalyzed by the bioinspired FLP. The relative Gibbs energies (Δ*G*) and potential energies (Δ*E*) are given in kcal/mol.

As shown in Figure 7, the H(2)^**...**^H(4) distance in the structure of **A7** is 3.84 Å, and the NBO charges for H(2) and H(4) atoms are 0.52 and −0.02, respectively. Through the transition state **TSA7**, the H(2) and H(4) atoms can transfer to the C=O bond of formic acid in a concerted manner with an energy barrier of 16.5 kcal/mol. As a result, methane diol and the catalyst **A1** are formed, being exoenergic by 5.0 kcal/mol. In the following, FLP **A1** can facilitate the decomposition of methane diol to form formaldehyde. Through the H^**...**^O hydrogen-bonding and O→B coordinative interactions, **A1** can bind methane diol to afford the intermediate **A8**. Over the transition state **TSA8**, the methane diol moiety in **A8** can be decomposed with the cleavage of C(3)–O(2) and O(3)–H(2) bonds, and the corresponding energy barrier is only 6.0 kcal/mol. After that, formaldehyde is released. The generated intermediate **A3** will release a water molecule through the water activation pathway in Figure 2 (**A3 → TSA2–3 → A2 → A1**). The bioinspired FLP **A1** then undergoes hydrogen activation (see pathway in Figure 6) and the C=O bond hydrogenation process and forms the final methanol product.

In brief, the reaction heat of the whole reaction is −18.2 kcal/mol, and the 1,1-addition of CO via the transition state **TSA3–4** (stage 1) is the rate-determining step with an energy span of 25.6 kcal/mol for the bioinspired FLP **A1**. Moreover, the pathways created by the modified bioinspired FLPs **L1** and **M1** are shown in Figures S16 and S17 in SI and predicted to own even lower energy spans (22.1 and 20.6 kcal/mol), suggesting that the designed CO hydrogenation to methanol could be achieved at room temperatures according to both reported theoretical studies^80−84^ and Eyring equation that correlates the activation energy (Δ*G*^‡^) and the rate constant (k) of the rate-determining step.^85^

## 4 Conclusions

The organic bioinspired FLP **A1** featuring a nucleic acid base fused π-conjugated linker is theoretically designed and can provide a suitable pocket for the synchronous activation of two substrate molecules. The computations suggest that the biomimetic FLP catalyst can exhibit good tolerance to CO poison. The base-free water-assisted indirect CO hydrogenation to methanol catalyzed by the bioinspired FLP catalysts contains two stages: the 1,1-addition of CO into the formic acid intermediate and the hydrogenation of the formic acid intermediate into methanol. The 1,1-addition of CO via the transition state **TSA3–4** is the rate-determining step with an energy span of 25.6 kcal/mol. Furthermore, the activities of the bioinspired FLP catalysts can be controlled through changing the Lewis acidity of the B atom, and the modified catalysts **L1** and **M1** yield lower energy spans (22.1 and 20.6 kcal/mol). These low-energy spans suggest that CO can be hydrogenated into methanol at low operating temperatures.

The comparisons between the typical traditional catalysts and the designed bioinspired FLP catalysts unveil that the new extended aromatization**–**dearomatization effect involving multiple rings of bioinspired FLP catalysts is an effective strategy to facilitate the rate-determining CO 1,1-addition step. According to the dynamic NLMO analysis, a new model involving σ-donation and σ-back-donation interactions is proposed for CO activation and should guide the development of organic catalysts for CO hydrogenations. The present study will pave the way to a new method for the hydrogenation of CO to methanol with good stability, low cost, environmental friendliness, and low temperatures.

## Data Availability

The data underlying this study are available in the published article and its Supporting Information.

## Supporting Information Available

Optimized geometries and energies of all stationary points along the reaction pathways and the imaginary vibrational frequencies of transition states (PDF)
