# Supplementary material for: Biomimetic Frustrated Lewis Pair Catalysts for Hydrogenation of CO to Methanol at Low Temperatures
Source: ACS Org Inorg Au. 2024 Jan 31;4(2):258–67. doi: 10.1021/acsorginorgau.3c00064 (PMC10996047; doi:10.1021/acsorginorgau.3c00064)
Supplement: Supplementary file 1 — gg3c00064_si_001.pdf [file gg3c00064_si_001.pdf]

Supporting Information

For

Biomimetic Frustrated Lewis Pair Catalysts for Hydrogenation of CO to Methanol at Low Temperatures

Jiejing Zhang,[†] Longfei Li,*[,†] Xiaofeng Xie,[†] Xue-Qing Song,*[,†] Henry F. Schaefer III*[,‡]

[†] *College of Pharmacy, Key Laboratory of Pharmaceutical Quality Control of Hebei Province, Key Laboratory of Medicinal Chemistry and Molecular Diagnosis of Ministry of Education, Hebei University, Baoding 071002, Hebei, P. R. China*

[‡] *Center for Computational Quantum Chemistry, University of Georgia, Athens, Georgia 30602, United States*

*Email: lilongfei@hbu.edu.cn (L.L.); sxqing@hbu.edu.cn (X.S.); ccq@uga.edu (H.F.)

Table S1. The computed potential energies (*E*, a.u.) and relative potential energies (Δ*E*, kcal/mol) at the ωB97X-D/6-311++G(2d,p) level. The relative Gibbs energies (Δ*G*, 298.15K) are in kcal/mol. S3

Table S2. The imaginary frequencies (in cm^{-1}) of transition states at the ωB97X-D/6-311G(d,p) level........ S7

Figure S1. The geometric structures and NBO atomic charges for the intermediates and transition states in the hydrogenation of CO to methanol catalyzed by the bioinspired FLP **A1**. The pentafluorophenyl groups and the tertiary butyl groups are drawn in wireframe for simplicity. S10

Figure S2. The dimerization of **A1** through forming a Lewis adduct. S11

Figure S3. The steric and electronic effects of promoters on the rate-determining CO 1,1-addition step. S12

Figure S4. The solvation effects on the rate-determining CO 1,1-addition step by the bioinspired FLP **A1**. The relative Gibbs energies (Δ*G*) is in kcal/mol. S13

Figure S5. A possible synthesis route for the biomimetic FLP **A1** catalyst. S14

Figure S6. The pathway for the hydrogenation of CO to methanol by the traditional FLP **B1**. The relative Gibbs energies (Δ*G*) and potential energies (Δ*E*) are in kcal/mol. S15

Figure S7. The pathway for the hydrogenation of CO to methanol by the traditional FLP **C1**. The relative Gibbs energies (Δ*G*) and potential energies (Δ*E*) are in kcal/mol. S16

Figure S8. The pathway for the hydrogenation of CO to methanol by the traditional FLP **D1**. The relative Gibbs energies (Δ*G*) and potential energies (Δ*E*) are in kcal/mol. S17
Figure S9. The pathway for the hydrogenation of CO to methanol by the traditional FLP **E1**. The relative Gibbs energies (Δ*G*) and potential energies (Δ*E*) are in kcal/mol. S18
Figure S10. The pathway for the hydrogenation of CO to methanol by the traditional FLP **F1**. The relative Gibbs energies (Δ*G*) and potential energies (Δ*E*) are in kcal/mol. S19
Figure S11. The pathway for the hydrogenation of CO to methanol by the traditional FLP **G1**. The relative Gibbs energies (Δ*G*) and potential energies (Δ*E*) are in kcal/mol. S20
Figure S12. The pathway for the hydrogenation of CO to methanol by the traditional FLP **H1**. The relative Gibbs energies (Δ*G*) and potential energies (Δ*E*) are in kcal/mol. S21
Figure S13. The pathway for the hydrogenation of CO to methanol by the traditional FLP **I1**. The relative Gibbs energies (Δ*G*) and potential energies (Δ*E*) are in kcal/mol. S22
Figure S14. The pathway for the hydrogenation of CO to methanol by the designed bioinspired FLP **J1**. The relative Gibbs energies (Δ*G*) and potential energies (Δ*E*) are in kcal/mol. S23
Figure S15. The pathway for the hydrogenation of CO to methanol by the designed bioinspired FLP **K1**. The relative Gibbs energies (Δ*G*) and potential energies (Δ*E*) are in kcal/mol. S24
Figure S16. The pathway for the hydrogenation of CO to methanol by the designed bioinspired FLP **L1**. The relative Gibbs energies (Δ*G*) and potential energies (Δ*E*) are in kcal/mol............ S25
Figure S17. The pathway for the hydrogenation of CO to methanol by the designed bioinspired FLP **M1**. The relative Gibbs energies (Δ*G*) and potential energies (Δ*E*) are in kcal/mol........... S26
Figure S18. The pathway for the hydrogenation of CO to methanol by the designed bioinspired FLP **A1** with the B3LYP-D3BJ functional. The relative Gibbs energies (ΔG) and potential energies (ΔE) are in kcal/mol. S27
Figure S19. The H_2 molecule cleave between the N(2) atom and the C atom of CO with the ωB97X-D functional. The relative Gibbs energies (Δ*G*) and potential energies (Δ*E*) are in kcal/mol. ... S29
Cartesian coordinates of all stationary points optimized at ωB97X-D/6-311G(d,p) level in dichloromethane solvent. S30

Table S1. The computed potential energies (E, a.u.) and relative potential energies (ΔE, kcal/mol) at the ωB97X-D/6-311++G(2d,p) level. The relative Gibbs energies (ΔG, 298.15K) are in kcal/mol.

Complex	E	ΔE	G	ΔG
**A1**	-2437.6352	0.0	-2437.2736	0.0
**A1_CO**	-2550.9533	-8.1	-2550.5846	5.2
**A2**	-2514.0899	-8.5	-2513.7048	4.3
**A3**	-2514.0902	-8.7	-2513.7022	5.9
**A4**	-2627.4032	-13.5	-2627.0117	12.0
**A5**	-2515.2530	-15.7	-2514.8511	21.4
**A6**	-2515.2839	-35.1	-2514.8800	3.3
**A7**	-2438.8226	-22.5	-2438.4383	5.6
**A8**	-2628.6292	-45.0	-2628.2105	-1.5
**TSA2-3**	-2514.0748	1.0	-2513.6911	12.8
**TSA3-4**	-2627.3789	1.7	-2626.9901	25.6
**TSA1-7**	-2438.7645	14.0	-2438.3885	36.8
**TSA5-6**	-2515.2519	-15.0	-2514.8517	21.0
**TSA7**	-2628.5862	-18.1	-2628.1729	22.1
**TSA8**	-2628.6137	-35.3	-2628.2009	4.5
**TSA9**	-2553.3372	-28.4	-2552.9334	8.7
**TSA3-4_CH_3OH**	-2666.6685	2.1	-2666.2506	28.1
**TSA3-4_EtOH**	-2705.9873	2.7	-2705.5436	28.7
**TSA3-4_PhOH**	-2858.3948	8.4	-2857.9295	34.2
**TSA3-4_^{i}PrOH**	-2745.2958	9.9	-2744.8241	36.9
**TSA3-4_^{t}BuOH**	-2784.6076	14.3	-2784.1112	40.4
**TSA3-4_PhOH-NH_2**	-2913.7587	6.9	-2913.2774	33.4
**TSA3-4_PhOH-OCH_3**	-2972.9187	7.0	-2972.4239	33.7
**TSA3-4_PhOH-F**	-2957.6386	7.6	-2957.1815	34.3
**TSA3-4_PhOH-NO_2**	-3062.9026	9.0	-3062.4379	35.1
**TSA3-4_PhOH-CH_3**	-2897.7123	7.7	-2897.2198	35.2
**A1_toluene**	-2437.6245	0.0	-2437.2630	0.0
**A2_toluene**	-2514.0753	-8.3	-2513.6902	4.1
**A3_toluene**	-2514.0711	-5.6	-2513.6830	8.6
**A4_toluene**	-2627.3916	-14.7	-2626.9996	10.7
**TSA2-3_toluene**	-2514.0626	-0.3	-2513.6787	11.3
**TSA3-4_toluene**	-2627.3662	1.2	-2626.9782	24.1
**A1_ethanol**	-2437.6309	0.0	-2437.2700	0.0
**A2_ethanol**	-2514.0918	-9.2	-2513.7061	3.9
**A3_ethanol**	-2514.0912	-8.8	-2513.7010	7.1
**A4_ethanol**	-2627.4015	-14.1	-2627.0117	10.3
**TSA2-3_ethanol**	-2514.0708	4.0	-2513.6869	15.9
**TSA3-4_ethanol**	-2627.3728	3.9	-2626.9855	26.8
**A1_methanol**	-2437.6309	0.0	-2437.2693	0.0
**A2_methanol**	-2514.0902	-8.0	-2513.7032	5.5
**A3_methanol**	-2514.0915	-8.8	-2513.7017	6.5
**A4_methanol**	-2627.4016	-14.4	-2627.0105	10.4
**TSA2-3_methanol**	-2514.0702	4.5	-2513.6867	15.9
**TSA3-4_methanol**	-2627.3715	4.5	-2626.9840	27.0
**B1($B(C_6F_5)_3$)**	-2208.3410	0.0	-2208.2488	0.0
**B1(TMP)**	-409.1710	0.0	-408.9347	0.0
**B1_CO**	-2321.6633	-10.7	-2321.5616	4.1
**B1-2**	-2617.5213	-5.8	-2617.1541	18.4
**B3**	-2694.0399	-54.3	-2693.6543	-20.6
**B4**	-2807.3038	-28.4	-2806.9173	14.7
**B7**	-2618.7669	-64.8	-2618.3869	-18.7
**B8**	-2808.5273	-58.3	-2808.1155	1.6
**TSB3-4**	-2807.2901	-19.8	-2806.9049	22.5
**TSB1-7**	-2618.7043	-25.5	-2618.3344	14.2
**TSB7**	-2808.4942	-37.6	-2808.0845	21.0
**TSB8**	-2808.5230	-55.6	-2808.1130	3.1
**TSB9**	-2733.2567	-55.1	-2732.8528	2.7
**C1**	-2159.4243	0.0	-2159.0100	0.0
**C1_CO**	-2272.7576	-17.6	-2272.3331	-2.4
**C3**	-2235.9299	-40.4	-2235.4907	-26.8
**C4**	-2349.1963	-16.0	-2348.7508	10.4
**C7**	-2160.6569	-50.8	-2160.2237	-25.1

**C8**	-2350.4162	-43.7	-2349.9430	1.0
**TSC3-4**	-2349.1793	-5.3	-2348.7366	19.2
**TSC1-7**	-2160.6013	-15.9	-2160.1732	6.5
**TSC7**	-2350.3802	-21.1	-2349.9151	18.5
**TSC8**	-2350.4013	-34.3	-2349.9340	6.6
**TSC9**	-2275.1406	-37.3	-2274.6802	2.2
**D1(B(C_6F_5)$_3$)**	-2208.3410	0.0	-2208.2488	0.0
**D1(^{t}Bu$_3$P)**	-814.9126	0.0	-814.5824	0.0
**D1_CO**	-2321.6633	-10.7	-2321.5616	4.1
**D1-2**	-3023.2597	-3.9	-3022.8113	12.5
**D2**	-3099.7486	-33.8	-3099.2748	-3.5
**D3**	-3099.7708	-47.7	-3099.2978	-17.9
**D4**	-3213.0470	-29.5	-3212.5638	15.5
**D7**	-3024.5039	-62.0	-3024.0365	-19.9
**D8**	-3214.2629	-54.6	-3213.7549	6.8
**TSD2-3**	-3099.7486	-33.8	-3099.2754	-3.8
**TSD3-4**	-3213.0240	-15.0	-3212.5478	25.5
**TSD1-7**	-3024.4382	-20.7	-3023.9734	19.7
**TSD7**	-3214.2293	-33.6	-3213.7287	23.2
**TSD8**	-3214.2503	-46.7	-3213.7504	9.6
**TSD9**	-3138.9871	-48.1	-3138.4947	6.4
**E1(B(C_6F_5)$_3$)**	-2208.3410	0.0	-2208.2488	0.0
**E1(Ph$_3$P)**	-1036.2750	0.0	-1036.0441	0.0
**E1_CO**	-2321.6633	-10.7	-2321.5616	4.1
**E1-2**	-3244.6472	-19.6	-3244.2905	1.5
**E2**	-3321.1084	-32.2	-3320.7325	-1.0
**E3**	-3321.1108	-33.7	-3320.7367	-3.6
**E4**	-3434.4092	-29.4	-3434.0248	15.9
**E7**	-3245.8428	-47.2	-3245.4749	-5.3
**E8**	-3435.6226	-52.9	-3435.2153	7.6
**TSE2-3**	-3321.1024	-28.4	-3320.7305	0.3
**TSE3-4**	-3434.3792	-10.5	-3434.0010	30.9
**TSE1-7**	-3245.8021	-21.7	-3245.4340	20.4
**TSE7**	-3435.5845	-29.0	-3435.1852	26.5
**TSE8**	-3435.6015	-39.7	-3435.2016	16.2
**TSE9**	-3360.3321	-37.2	-3359.9401	16.6
**F1**	-2599.6776	0.0	-2599.2385	0.0
**F1_CO**	-2713.0179	-22.0	-2712.5685	-6.8
**F2**	-2676.1440	-15.8	-2675.6780	-0.9
**F3**	-2676.1619	-27.0	-2675.6963	-12.4
**F4**	-2789.4450	-13.1	-2788.9760	12.4
**F7**	-2600.8921	-39.5	-2600.4293	-10.8
**F8**	-2790.6602	-37.9	-2790.1662	4.2
**TSF2-3**	-2676.1430	-15.2	-2675.6798	-2.1
**TSF3-4**	-2789.4201	2.5	-2788.9546	25.8
**TSF1-7**	-2600.8480	-11.8	-2600.3931	11.9
**TSF7**	-2790.6262	-16.5	-2790.1355	23.5
**TSF8**	-2790.6414	-26.0	-2790.1514	13.5
**TSF9**	-2715.3794	-28.2	-2714.8976	9.1
**G1**	-2363.7860	0.0	-2363.5101	0.0
**G1_CO**	-2477.1089	-11.0	-2476.8234	3.7
**G2**	-2440.2498	-14.1	-2439.9424	3.6
**G3**	-2440.2582	-19.4	-2439.9546	-4.1
**G4**	-2553.5530	-12.8	-2553.2438	14.8
**G7**	-2364.9938	-35.3	-2364.6923	-5.3
**G8**	-2554.7694	-38.3	-2554.4337	6.9
**TSG2-3**	-2440.2441	-10.6	-2439.9423	3.7
**TSG3-4**	-2553.5191	8.4	-2553.2144	33.3
**TSG1-7**	-2364.9503	-8.0	-2364.6547	18.2
**TSG7**	-2554.7299	-13.5	-2554.4022	26.6
**TSG8**	-2554.7473	-24.4	-2554.4207	15.0
**TSG9**	-2479.4808	-23.8	-2479.1587	15.7
**H1**	-2845.5697	0.0	-2845.0832	0.0
**H1_CO**	-2958.8901	-9.5	-2958.3955	4.3
**H2**	-2922.0264	-9.7	-2921.5147	4.1
**H3**	-2922.0434	-20.4	-2921.5345	-8.3
**H4**	-3035.3345	-11.5	-3034.8165	15.1
**H5**	-2923.1824	-12.5	-2922.6574	23.5
**H6**	-2923.2274	-40.8	-2922.6987	-2.4

**H7**	-2846.7793	-36.4	-2846.2721	-9.6
**H8**	-3036.5588	-41.9	-3036.0144	2.1
**TSH2-3**	-2922.0211	-6.4	-2921.5151	3.9
**TSH3-4**	-3035.3046	7.2	-3034.7926	30.0
**TSH5-6**	-2923.1820	-12.3	-2922.6576	23.4
**TSH7**	-3036.5205	-17.9	-3035.9824	22.1
**TSH8**	-3036.5396	-29.9	-3036.0013	10.3
**TSH9**	-2961.2756	-30.8	-2960.7440	8.1
**I1**	-2993.1402	0.0	-2992.7195	0.0
**I1_CO**	-3106.4607	-9.6	-3106.0320	4.2
**I2**	-3069.5989	-11.0	-3069.1505	4.4
**I3**	-3069.5887	-4.6	-3069.1471	6.5
**I4**	-3182.9033	-10.5	-3182.4488	17.5
**I5**	-3070.7431	-6.3	-3070.2827	30.4
**I6**	-3070.7775	-27.9	-3070.3136	11.0
**I7**	-2994.3274	-22.4	-2993.8832	6.2
**I8**	-3184.1185	-35.2	-3183.6411	8.1
**TSI2-3**	-3069.5761	3.4	-3069.1349	14.2
**TSI3-4**	-3182.8644	14.0	-3182.4218	34.5
**TSI5-6**	-3070.7420	-5.6	-3070.2818	30.9
**TSI7**	-3184.0839	-13.4	-3183.6101	27.6
**TSI8**	-3184.0957	-20.8	-3183.6263	17.4
**TSI9**	-3108.8284	-19.7	-3108.3639	18.4
**J1(B(C_6F_5)$_3$)**	-2208.3410	0.0	-2208.2488	0.0
**J1**	-638.0265	0.0	-637.7476	0.0
**J1_CO**	-2321.6633	-10.7	-2321.5616	4.1
**J1-2**	-2846.3910	-14.7	-2845.9873	5.7
**J2**	-2922.8622	-33.6	-2922.4392	-3.0
**J3**	-2922.8630	-34.1	-2922.4397	-3.3
**J4**	-3036.1625	-30.5	-3035.7302	14.7
**J7**	-2847.5831	-40.2	-2847.1604	6.0
**J8**	-3037.3817	-57.7	-3036.9228	5.1
**TSJ2-3**	-2922.8612	-33.0	-2922.4417	-4.6
**TSJ3-4**	-3036.1304	-10.3	-3035.7054	30.3
**TSJ1-7**	-2847.5534	-21.6	-2847.1362	21.2
**TSJ7**	-3037.3434	-33.6	-3036.8889	26.3
**TSJ8**	-3037.3610	-44.7	-3036.9118	12.0
**TSJ9**	-2962.0948	-44.2	-2961.6484	13.6
**K1**	-1445.2572	0.0	-1444.8020	0.0
**K1_CO**	-1558.5721	-6.0	-1558.1135	4.8
**K2**	-1521.7049	-4.1	-1521.2274	7.9
**K3**	-1521.6946	2.4	-1521.2162	14.9
**K4**	-1635.0517	-30.2	-1634.5674	-5.1
**K5**	-1522.8888	-24.4	-1522.4012	7.8
**K6**	-1522.8870	-23.3	-1522.3910	14.2
**K7**	-1446.4221	-8.4	-1445.9512	15.3
**K8**	-1636.2435	-40.2	-1635.7319	2.8
**TSK2-3**	-1521.6868	7.2	-1521.2128	17.1
**TSK3-4**	-1634.9876	10.1	-1634.5085	31.8
**TSK1-7**	-1446.3781	19.2	-1445.9110	40.5
**TSK5-6**	-1522.8665	-10.4	-1522.3752	24.1
**TSK7**	-1636.1990	-12.3	-1635.6947	26.2
**TSK8**	-1636.2205	-25.7	-1635.7124	15.1
**TSK9**	-1560.9454	-19.7	-1560.4450	19.2
**L1**	-2636.1144	0.0	-2635.7717	0.0
**L1_CO**	-2749.4303	-6.7	-2749.0830	5.0
**L2**	-2712.5719	-10.2	-2712.2037	3.8
**L3**	-2712.5748	-12.0	-2712.2075	1.4
**L4**	-2825.8860	-15.8	-2825.5121	10.6
**L5**	-2713.7353	-17.6	-2713.3545	18.1
**L6**	-2713.7666	-37.3	-2713.3802	2.0
**L7**	-2637.3065	-25.4	-2636.9408	2.8
**L8**	-2827.1124	-47.5	-2826.7126	-4.0
**TSL2-3**	-2712.5585	-1.8	-2712.1947	9.5
**TSL3-4**	-2825.8634	-1.6	-2825.4938	22.1
**TSL1-7**	-2637.2463	12.3	-2636.8899	34.8
**TSL5-6**	-2713.7350	-17.5	-2713.3524	19.4
**TSL7**	-2827.0705	-21.2	-2826.6775	18.1
**TSL8**	-2827.0981	-38.6	-2826.7027	2.2

**TSL9**	-2751.8207	-31.0	-2751.4346	6.8
**M1**	-2834.5863	0.0	-2834.2614	0.0
**M1_CO**	-2947.9024	-6.8	-2947.5752	3.4
**M2**	-2911.0446	-10.7	-2910.6960	2.2
**M3**	-2911.0456	-11.4	-2910.6951	2.8
**M4**	-3024.3588	-16.3	-3024.0040	9.2
**M5**	-2912.2077	-17.9	-2911.8454	17.3
**M6**	-2912.2402	-38.4	-2911.8736	-0.4
**M7**	-2835.7781	-25.2	-2835.4314	2.3
**M8**	-3025.5849	-47.9	-3025.2018	-3.7
**TSM2-3**	-2911.0318	-2.7	-2910.6855	8.8
**TSM3-4**	-3024.3361	-2.0	-3023.9858	20.6
**TSM1-7**	-2835.7196	11.5	-2835.3806	34.2
**TSM5-6**	-2912.2075	-17.8	-2911.8448	17.8
**TSM7**	-3025.5433	-21.8	-3025.1666	18.4
**TSM8**	-3025.5703	-38.7	-3025.1941	1.2
**TSM9**	-2950.2931	-31.3	-2949.9250	6.3

Table S2. The imaginary frequencies (in cm^{-1}) of transition states at the ωB97X-D/6-311G(d,p) level.

Complex	Imaginary frequencies (in cm^{-1})
**TSA2-3**	1165.2400*i*
**TSA3-4**	1102.9515*i*
**TSA1-7**	781.2186*i*
**TSA5-6**	507.9973*i*
**TSA7**	367.7982*i*
**TSA8**	620.7411*i*
**TSA9**	943.0869*i*
**TSA3-4_CH_3OH**	1123.2823*i*
**TSA3-4_EtOH**	1109.3670*i*
**TSA3-4_PhOH**	575.2172*i*
**TSA3-4_^{i}PrOH**	1055.0610*i*
**TSA3-4-^{t}BuOH**	981.3438*i*
**TSA3-4_PhOH-NH_2**	701.1005*i*
**TSA3-4_PhOH-OCH_3**	650.5031*i*
**TSA3-4_PhOH-F**	563.6531*i*
**TSA3-4_PhOH-NO_2**	451.8271*i*
**TSA3-4_PhOH-CH_3**	599.3989*i*
**TSA2-3_toluene**	1086.4560*i*
**TSA3-4_toluene**	1055.0445*i*
**TSA2-3_ethanol**	1212.6900*i*
**TSA3-4_ethanol**	1170.3757*i*
**TSA2-3_methanol**	1252.9833*i*
**TSA3-4_methanol**	1181.2852*i*
**TSB3-4**	1227.9306*i*
**TSB1-7**	83.5689*i*
**TSB7**	114.8665*i*
**TSB8**	956.2719*i*
**TSB9**	1084.1674*i*
**TSC3-4**	1161.6099*i*
**TSC1-7**	279.0169*i*
**TSC7**	856.9239*i*
**TSC8**	992.0812*i*
**TSC9**	1050.7979*i*
**TSD2-3**	157.7555*i*
**TSD3-4**	1152.4264*i*
**TSD1-7**	93.0436*i*
**TSD7**	329.2645*i*
**TSD8**	1190.0679*i*
**TSD9**	1232.2131*i*
**TSE2-3**	1069.2517*i*
**TSE3-4**	1028.4303*i*
**TSE1-7**	179.7699*i*
**TSE7**	199.2895*i*
**TSE8**	1103.3991*i*
**TSE9**	1031.7928*i*
**TSF2-3**	1006.2751*i*
**TSF3-4**	1002.7797*i*
**TSF1-7**	430.8209*i*
**TSF7**	1333.7670*i*
**TSF8**	1023.6101*i*
**TSF9**	1075.1353*i*
**TSG2-3**	1205.5718*i*
**TSG3-4**	976.6912*i*
**TSG1-7**	586.9401*i*
**TSG7**	1311.4870*i*
**TSG8**	1078.8310*i*
**TSG9**	914.4640*i*
**TSH2-3**	1186.5132*i*
**TSH3-4**	855.8159*i*
**TSH5-6**	438.1149*i*
**TSH7**	228.8833*i*
**TSH8**	762.2181*i*
**TSH9**	716.9414*i*
**TSI2-3**	1211.2911*i*

**TSI3-4**	1011.6015*i*
**TSI5-6**	543.4686*i*
**TSI7**	134.3709*i*
**TSI8**	947.9578*i*
**TSI9**	646.3764*i*
**TSJ2-3**	733.2452*i*
**TSJ3-4**	779.3657*i*
**TSJ1-7**	162.5183*i*
**TSJ7**	210.0482*i*
**TSJ8**	760.3296*i*
**TSJ9**	934.1347*i*
**TSK2-3**	1021.9740*i*
**TSK3-4**	1126.6933*i*
**TSK1-7**	1265.6578*i*
**TSK5-6**	821.3867*i*
**TSK7**	355.8557*i*
**TSK8**	99.4993*i*
**TSK9**	91.9445*i*
**TSL2-3**	1172.6723*i*
**TSL3-4**	1076.0643*i*
**TSL1-7**	735.8329*i*
**TSL5-6**	161.5271*i*
**TSL7**	347.2829*i*
**TSL8**	673.0610*i*
**TSL9**	927.4020*i*
**TSM2-3**	1156.6942*i*
**TSM3-4**	1068.4506*i*
**TSM1-7**	726.9126*i*
**TSM5-6**	201.4287*i*
**TSM7**	339.7541*i*
**TSM8**	664.2418*i*
**TSM9**	873.9210*i*

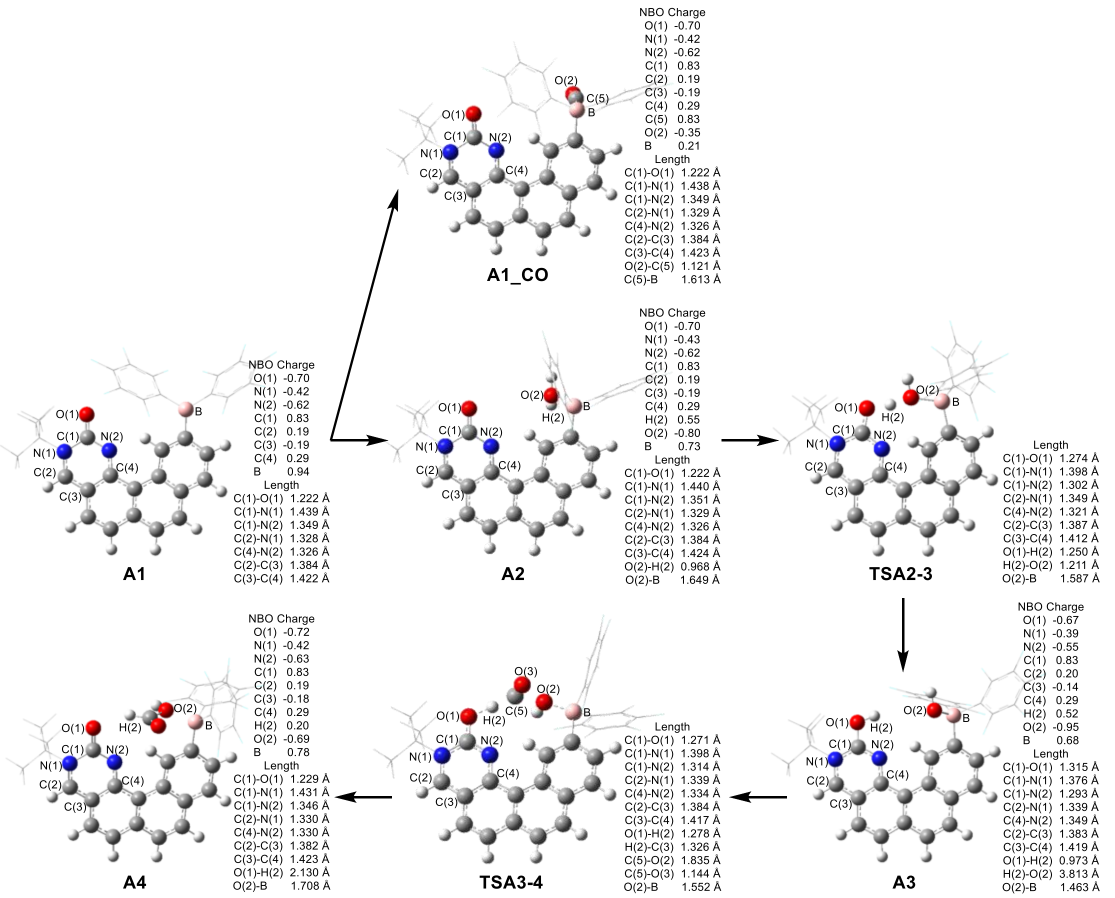
A1_CO
NBO Charge
O(1) -0.70
N(1) -0.42
N(2) -0.62
C(1) 0.83
C(2) 0.19
C(3) -0.19
C(4) 0.29
C(5) 0.83
O(2) -0.35
B 0.21
Length
C(1)-O(1) 1.222 Å
C(1)-N(1) 1.438 Å
C(1)-N(2) 1.349 Å
C(2)-N(1) 1.329 Å
C(4)-N(2) 1.326 Å
C(2)-C(3) 1.384 Å
C(3)-C(4) 1.423 Å
O(2)-C(5) 1.121 Å
C(5)-B 1.613 Å
A1
NBO Charge
O(1) -0.70
N(1) -0.42
N(2) -0.62
C(1) 0.83
C(2) 0.19
C(3) -0.19
C(4) 0.29
B 0.94
Length
C(1)-O(1) 1.222 Å
C(1)-N(1) 1.439 Å
C(1)-N(2) 1.349 Å
C(2)-N(1) 1.328 Å
C(4)-N(2) 1.326 Å
C(2)-C(3) 1.384 Å
C(3)-C(4) 1.422 Å
A2
NBO Charge
O(1) -0.70
N(1) -0.43
N(2) -0.62
C(1) 0.83
C(2) 0.19
C(3) -0.19
C(4) 0.29
H(2) 0.55
O(2) -0.80
B 0.73
Length
C(1)-O(1) 1.222 Å
C(1)-N(1) 1.440 Å
C(1)-N(2) 1.351 Å
C(2)-N(1) 1.329 Å
C(4)-N(2) 1.326 Å
C(2)-C(3) 1.384 Å
C(3)-C(4) 1.424 Å
O(2)-H(2) 0.968 Å
O(2)-B 1.649 Å
TSA2-3
Length
C(1)-O(1) 1.274 Å
C(1)-N(1) 1.398 Å
C(1)-N(2) 1.302 Å
C(2)-N(1) 1.349 Å
C(4)-N(2) 1.321 Å
C(2)-C(3) 1.387 Å
C(3)-C(4) 1.412 Å
O(1)-H(2) 1.250 Å
H(2)-O(2) 1.211 Å
O(2)-B 1.587 Å
A4
NBO Charge
O(1) -0.72
N(1) -0.42
N(2) -0.63
C(1) 0.83
C(2) 0.19
C(3) -0.18
C(4) 0.29
H(2) 0.20
O(2) -0.69
B 0.78
Length
C(1)-O(1) 1.229 Å
C(1)-N(1) 1.431 Å
C(1)-N(2) 1.346 Å
C(2)-N(1) 1.330 Å
C(4)-N(2) 1.330 Å
C(2)-C(3) 1.382 Å
C(3)-C(4) 1.423 Å
O(1)-H(2) 2.130 Å
O(2)-B 1.708 Å
TSA3-4
Length
C(1)-O(1) 1.271 Å
C(1)-N(1) 1.398 Å
C(1)-N(2) 1.314 Å
C(2)-N(1) 1.339 Å
C(4)-N(2) 1.334 Å
C(2)-C(3) 1.384 Å
C(3)-C(4) 1.417 Å
O(1)-H(2) 1.278 Å
H(2)-C(3) 1.326 Å
C(5)-O(2) 1.835 Å
C(5)-O(3) 1.144 Å
O(2)-B 1.552 Å
A3
NBO Charge
O(1) -0.67
N(1) -0.39
N(2) -0.55
C(1) 0.83
C(2) 0.20
C(3) -0.14
C(4) 0.29
H(2) 0.52
O(2) -0.95
B 0.68
Length
C(1)-O(1) 1.315 Å
C(1)-N(1) 1.376 Å
C(1)-N(2) 1.293 Å
C(2)-N(1) 1.339 Å
C(4)-N(2) 1.349 Å
C(2)-C(3) 1.383 Å
C(3)-C(4) 1.419 Å
O(1)-H(2) 0.973 Å
H(2)-O(2) 3.813 Å
O(2)-B 1.463 Å

(a)

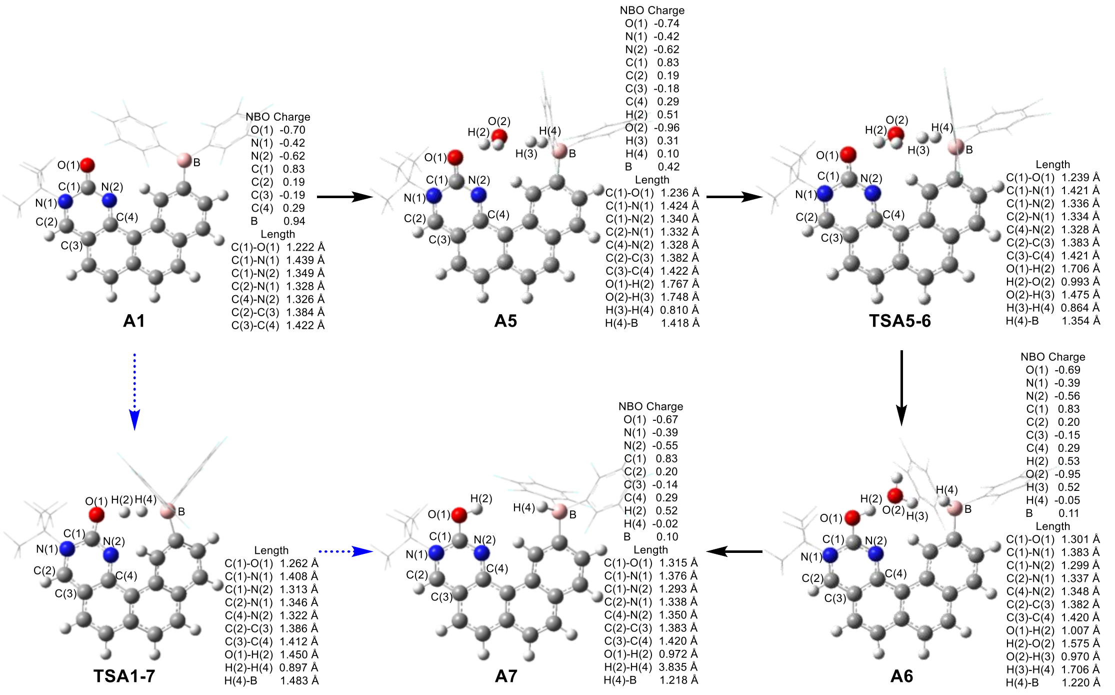
A1
NBO Charge
O(1) -0.70
N(1) -0.42
N(2) -0.62
C(1) 0.83
C(2) 0.19
C(3) -0.19
C(4) 0.29
B 0.94
Length
C(1)-O(1) 1.222 Å
C(1)-N(1) 1.439 Å
C(1)-N(2) 1.349 Å
C(2)-N(1) 1.328 Å
C(4)-N(2) 1.326 Å
C(2)-C(3) 1.384 Å
C(3)-C(4) 1.422 Å
A5
NBO Charge
O(1) -0.74
N(1) -0.42
N(2) -0.62
C(1) 0.83
C(2) 0.19
C(3) -0.18
C(4) 0.29
H(2) 0.51
O(2) -0.96
H(3) 0.31
H(4) 0.10
B 0.42
Length
C(1)-O(1) 1.236 Å
C(1)-N(1) 1.424 Å
C(1)-N(2) 1.340 Å
C(2)-N(1) 1.332 Å
C(4)-N(2) 1.328 Å
C(2)-C(3) 1.382 Å
C(3)-C(4) 1.422 Å
O(1)-H(2) 1.767 Å
O(2)-H(3) 1.748 Å
H(3)-H(4) 0.810 Å
H(4)-B 1.418 Å
TSA5-6
Length
C(1)-O(1) 1.239 Å
C(1)-N(1) 1.421 Å
C(1)-N(2) 1.336 Å
C(2)-N(1) 1.334 Å
C(4)-N(2) 1.328 Å
C(2)-C(3) 1.383 Å
C(3)-C(4) 1.421 Å
O(1)-H(2) 1.706 Å
H(2)-O(2) 0.993 Å
O(2)-H(3) 1.475 Å
H(3)-H(4) 0.864 Å
H(4)-B 1.354 Å
TSA1-7
Length
C(1)-O(1) 1.262 Å
C(1)-N(1) 1.408 Å
C(1)-N(2) 1.313 Å
C(2)-N(1) 1.346 Å
C(4)-N(2) 1.322 Å
C(2)-C(3) 1.386 Å
C(3)-C(4) 1.412 Å
O(1)-H(2) 1.450 Å
H(2)-H(4) 0.897 Å
H(4)-B 1.483 Å
A7
NBO Charge
O(1) -0.67
N(1) -0.39
N(2) -0.55
C(1) 0.83
C(2) 0.20
C(3) -0.14
C(4) 0.29
H(2) 0.52
H(4) -0.02
B 0.10
Length
C(1)-O(1) 1.315 Å
C(1)-N(1) 1.376 Å
C(1)-N(2) 1.293 Å
C(2)-N(1) 1.338 Å
C(4)-N(2) 1.350 Å
C(2)-C(3) 1.383 Å
C(3)-C(4) 1.420 Å
O(1)-H(2) 0.972 Å
H(2)-H(4) 3.835 Å
H(4)-B 1.218 Å
A6
NBO Charge
O(1) -0.69
N(1) -0.39
N(2) -0.56
C(1) 0.83
C(2) 0.20
C(3) -0.15
C(4) 0.29
H(2) 0.53
O(2) -0.95
H(3) 0.52
H(4) -0.05
B 0.11
Length
C(1)-O(1) 1.301 Å
C(1)-N(1) 1.383 Å
C(1)-N(2) 1.299 Å
C(2)-N(1) 1.337 Å
C(4)-N(2) 1.348 Å
C(2)-C(3) 1.382 Å
C(3)-C(4) 1.420 Å
O(1)-H(2) 1.007 Å
H(2)-O(2) 1.575 Å
O(2)-H(3) 0.970 Å
H(3)-H(4) 1.706 Å
H(4)-B 1.220 Å

(b)

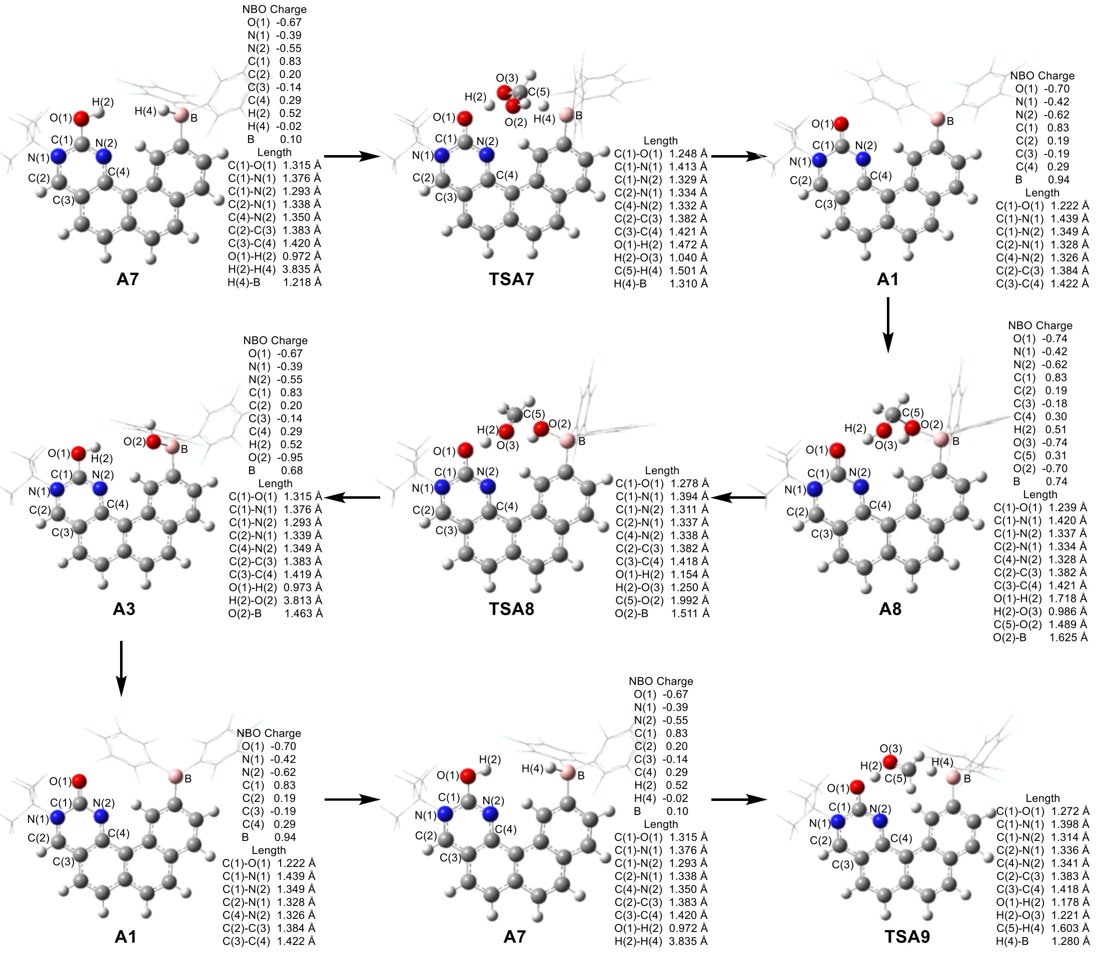

(c)

Figure S1. The geometric structures and NBO atomic charges for the intermediates and transition states in the hydrogenation of CO to methanol catalyzed by the bioinspired FLP **A1**. The pentafluorophenyl groups and the tertiary butyl groups are drawn in wireframe for simplicity.

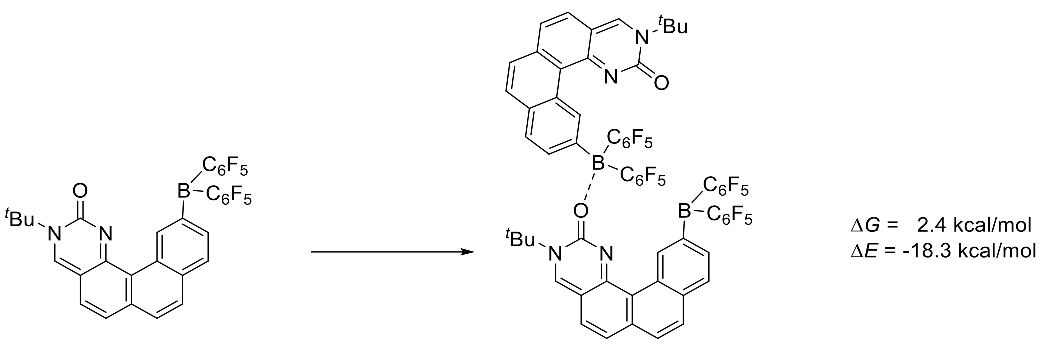

Figure S2. The dimerization of **A1** through forming a Lewis adduct.

The dimerization of **A1** through forming a Lewis adduct is considered in Figure S2. The step is thermodynamically disfavored with being endergonic by 2.4 kcal/mol. The monomer of **A1** is the major species in solution.

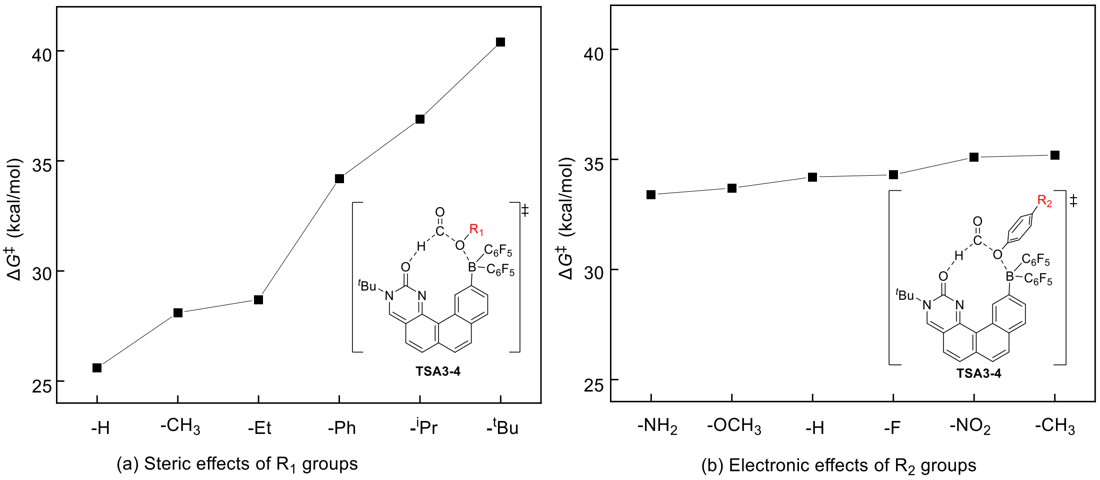

Figure S3. The steric and electronic effects of promoters on the rate-determining CO 1,1-addition step.

Various promoters are considered for the rate-determining CO 1,1-addition step, and the corresponding energy barriers of transition states (**TSA3-4**) are compared in Figure S3. It is found the steric effects of R_1 groups in HOR_1 influences the energy barriers largely, and the HO-H owns the lowest energy barrier. In contrast, the electronic effects of R_2 groups in HO-Ph-R_2 have small effects on the energy barriers.

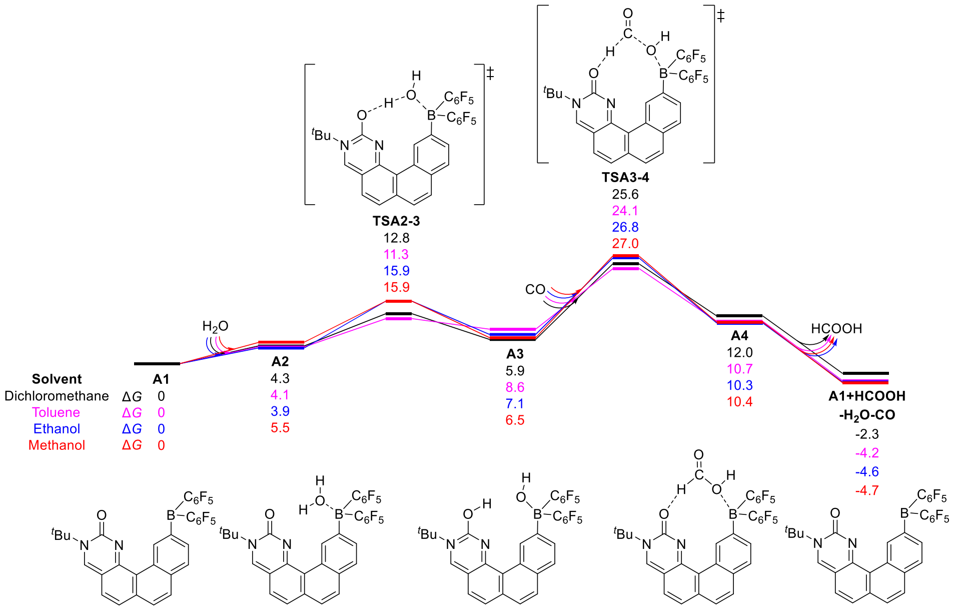

Figure S4. The solvation effects on the rate-determining CO 1,1-addition step by the bioinspired FLP **A1**. The relative Gibbs energies (ΔG) is in kcal/mol.

Different solvents including toluene (ε = 2.3741), dichloromethane (ε = 8.93), ethanol (ε = 24.852), and methanol (ε = 32.613) are compared for the rate-determining CO 1,1-addition step by the bioinspired FLP **A1**, and the Gibbs energy profiles are shown in Figure S4. It is found that these solvents have small effects on the energy barriers of **TSA3-4**.

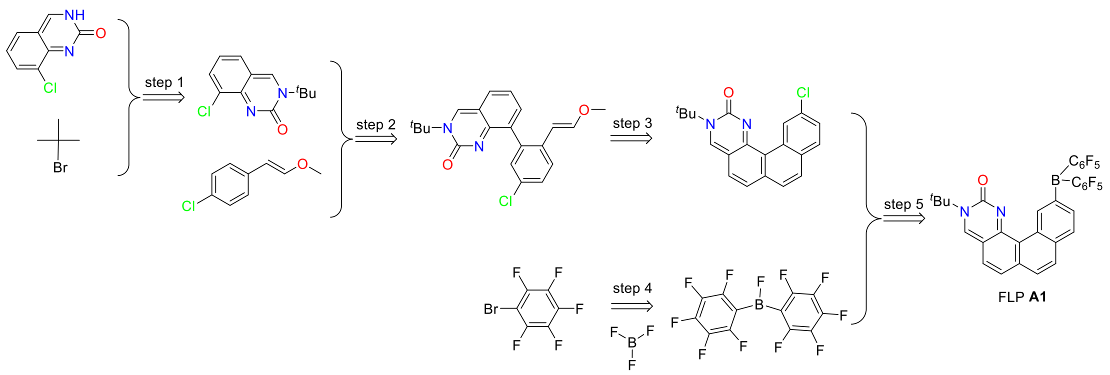

Figure S5. A possible synthesis route for the biomimetic FLP **A1** catalyst.

The pathways for the hydrogenation of CO to methanol by other FLP catalysts in Figure 4 of maintext.

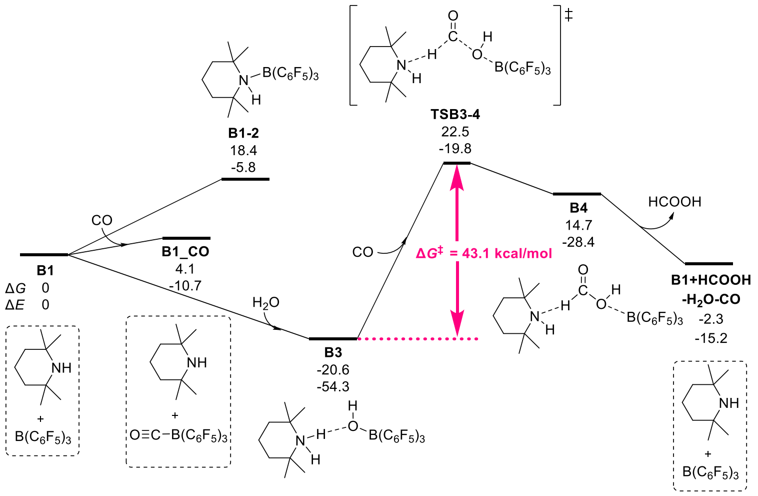

(a)

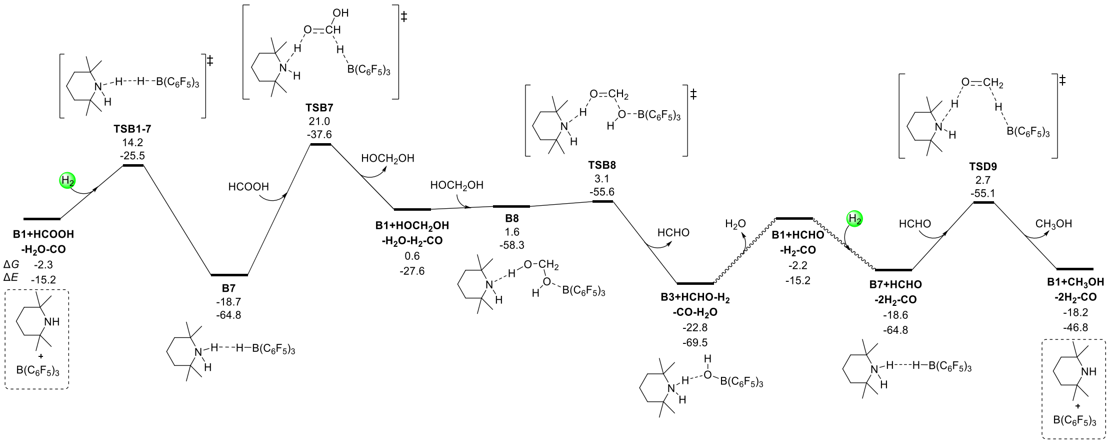

(b)

Figure S6. The pathway for the hydrogenation of CO to methanol by the traditional FLP **B1**. The relative Gibbs energies (ΔG) and potential energies (ΔE) are in kcal/mol.

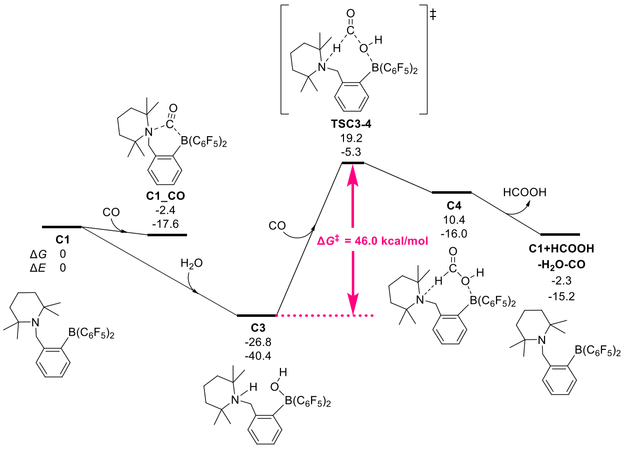

(a)

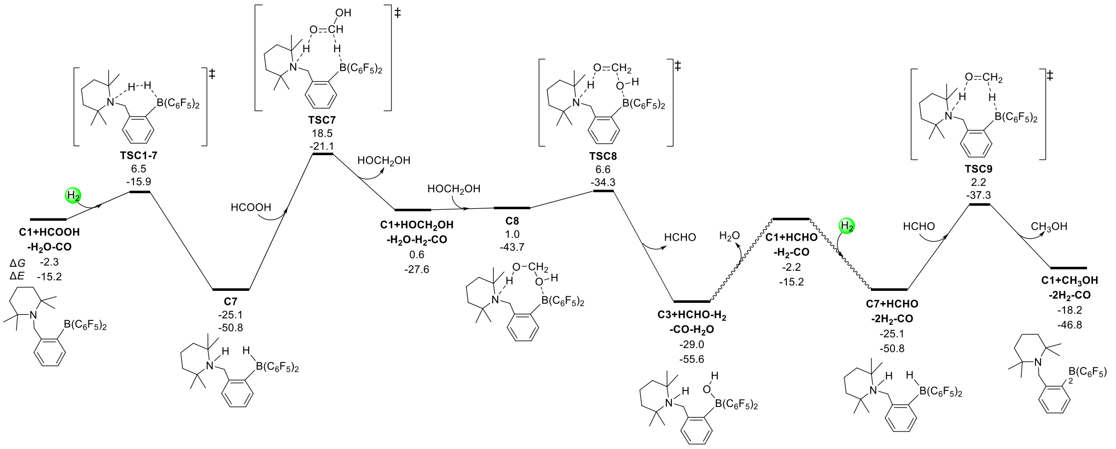

(b)

Figure S7. The pathway for the hydrogenation of CO to methanol by the traditional FLP **C1**. The relative Gibbs energies (ΔG) and potential energies (ΔE) are in kcal/mol.

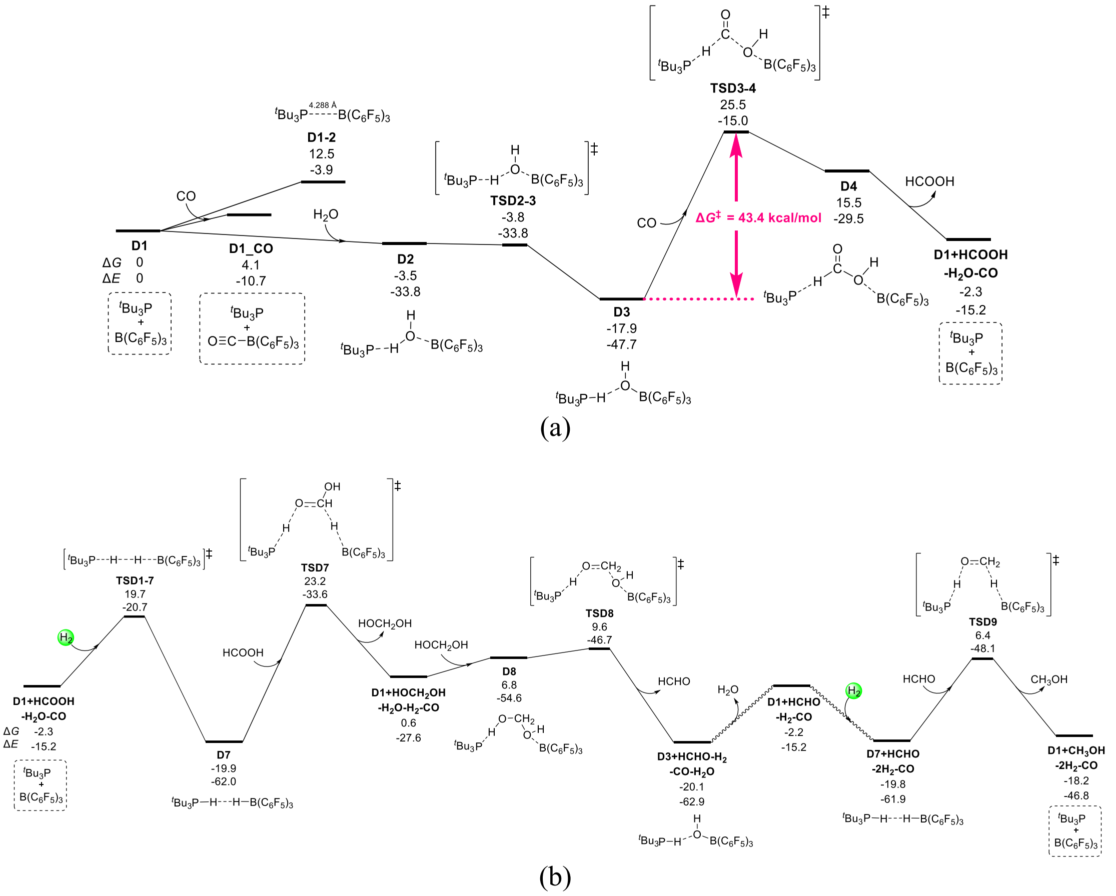

Figure S8. The pathway for the hydrogenation of CO to methanol by the traditional FLP **D1**. The relative Gibbs energies (ΔG) and potential energies (ΔE) are in kcal/mol.

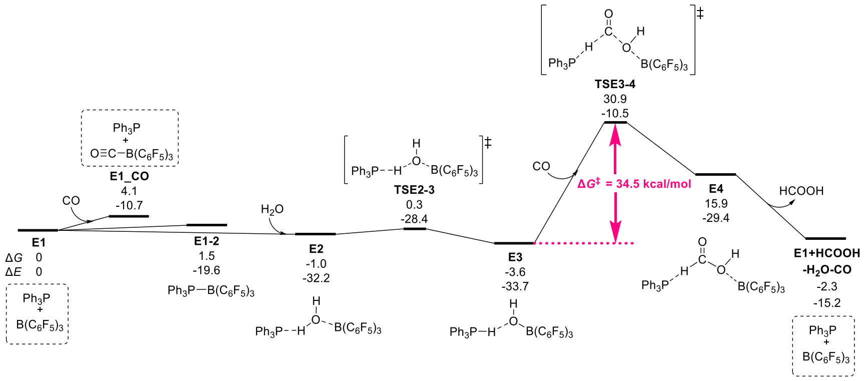

(a)

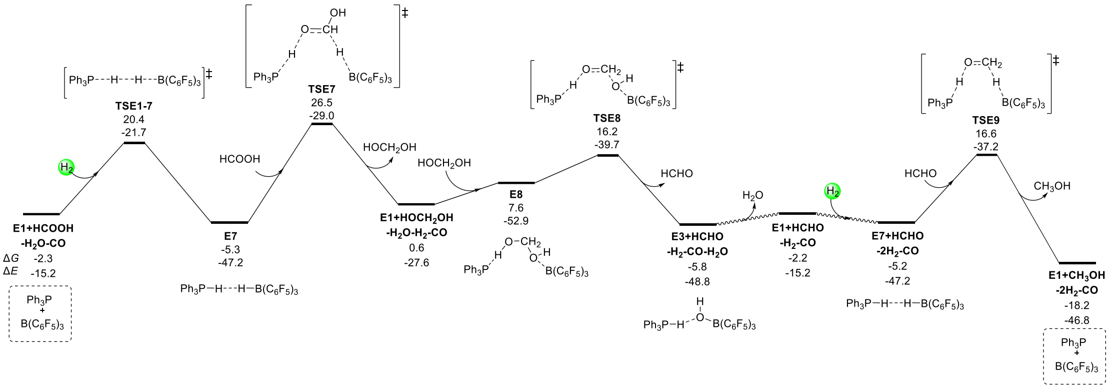

(b)

Figure S9. The pathway for the hydrogenation of CO to methanol by the traditional FLP **E1**. The relative Gibbs energies (ΔG) and potential energies (ΔE) are in kcal/mol.

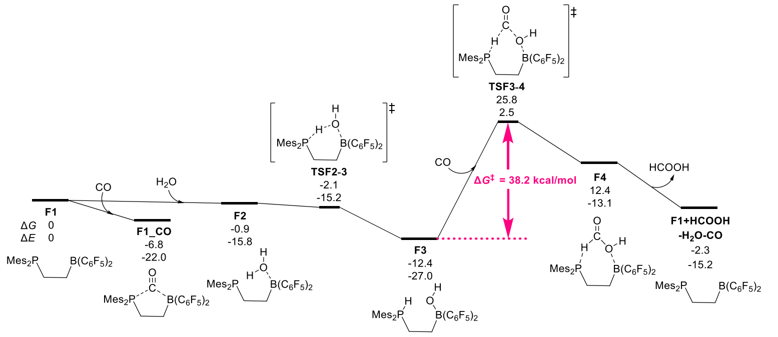

(a)

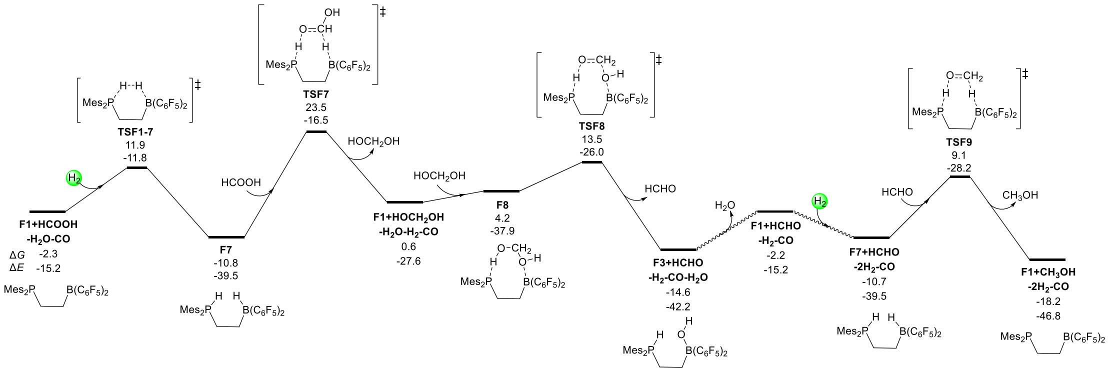

(b)

Figure S10. The pathway for the hydrogenation of CO to methanol by the traditional FLP **F1**. The relative Gibbs energies (Δ*G*) and potential energies (Δ*E*) are in kcal/mol.

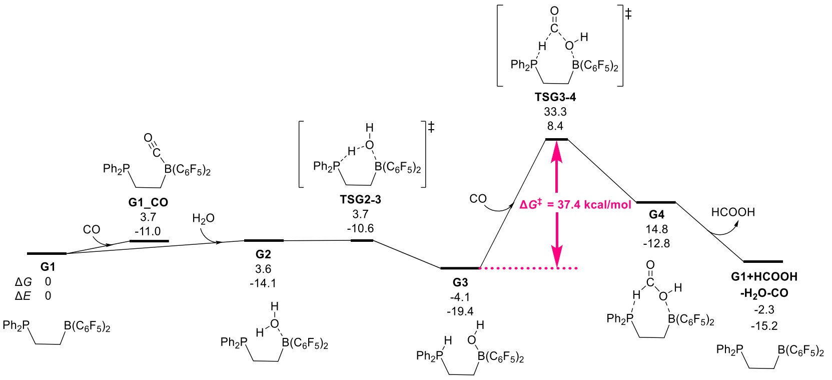

(a)

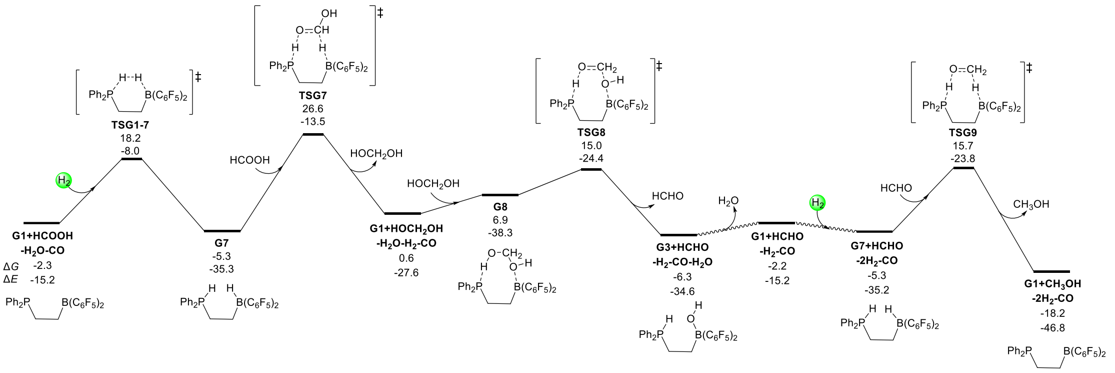

(b)

Figure S11. The pathway for the hydrogenation of CO to methanol by the traditional FLP **G1**. The relative Gibbs energies (ΔG) and potential energies (ΔE) are in kcal/mol.

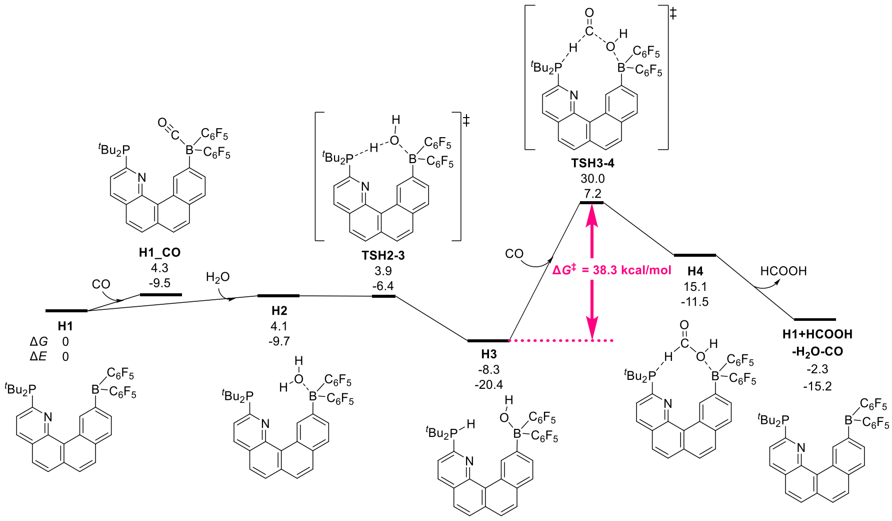

(a)

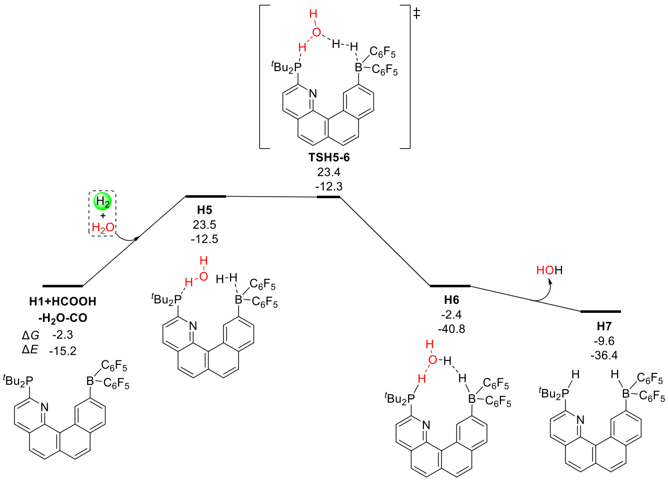

(b)

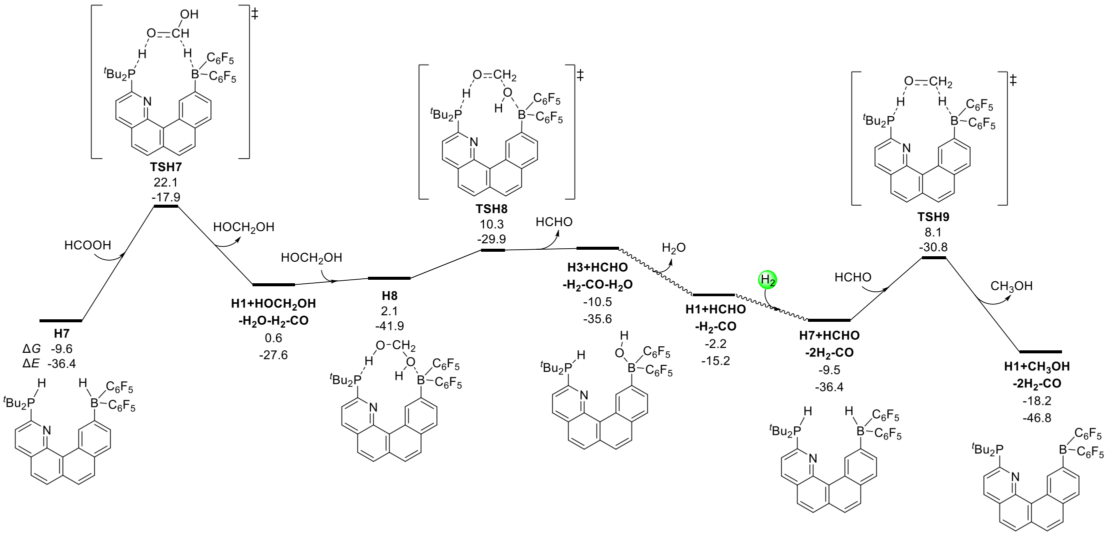

(c)

Figure S12. The pathway for the hydrogenation of CO to methanol by the traditional FLP **H1**. The relative Gibbs energies (ΔG) and potential energies (ΔE) are in kcal/mol.

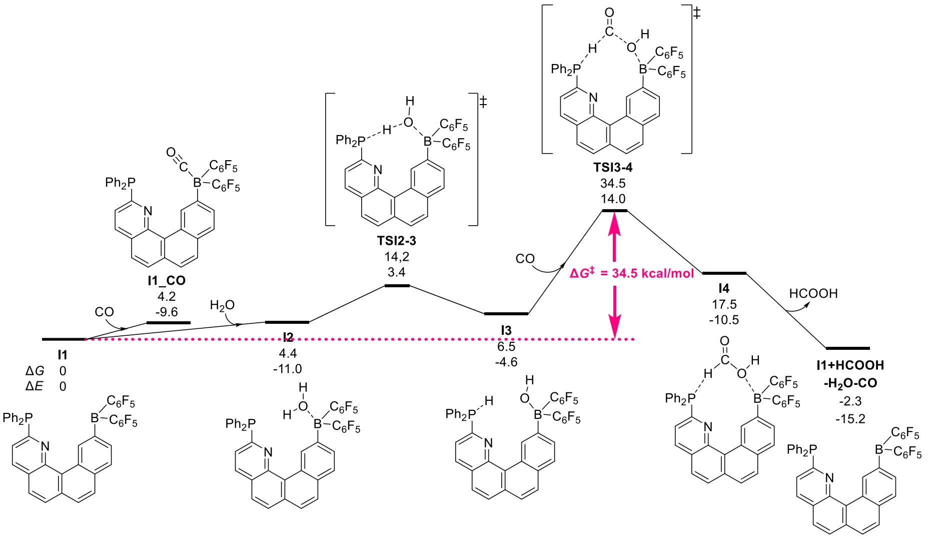

(a)

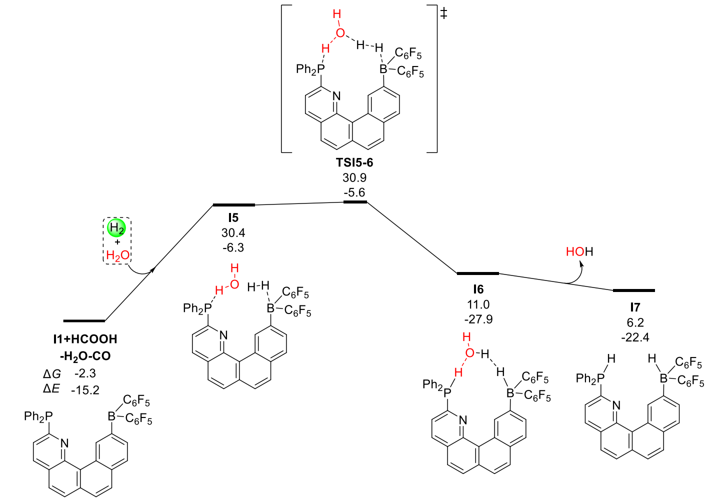

(b)

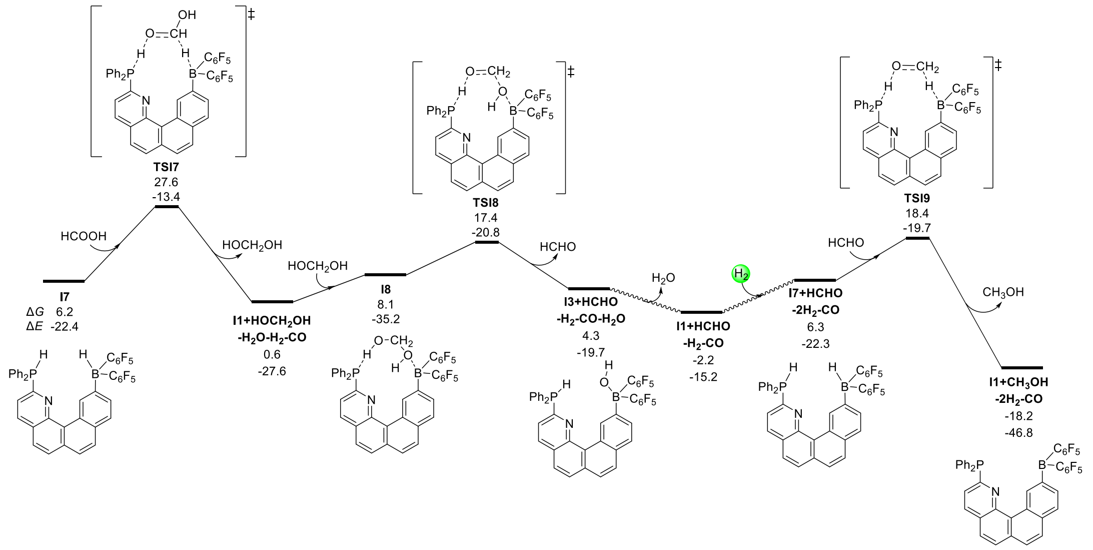

(c)

Figure S13. The pathway for the hydrogenation of CO to methanol by the traditional FLP **I1**. The relative Gibbs energies (ΔG) and potential energies (ΔE) are in kcal/mol.

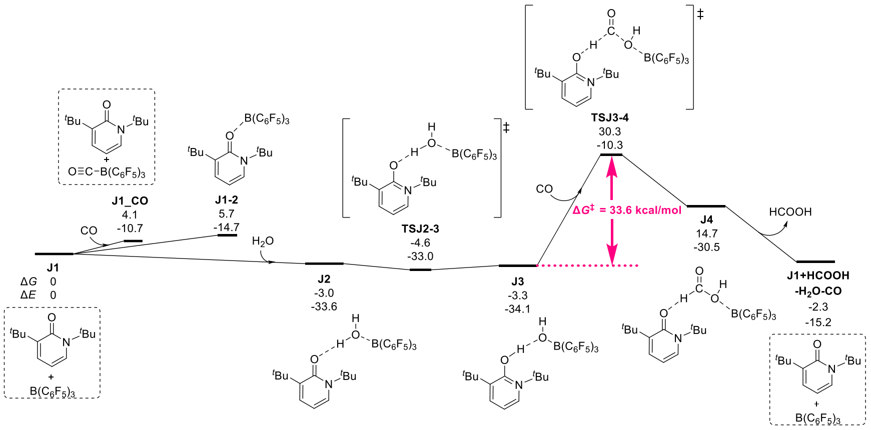

(a)

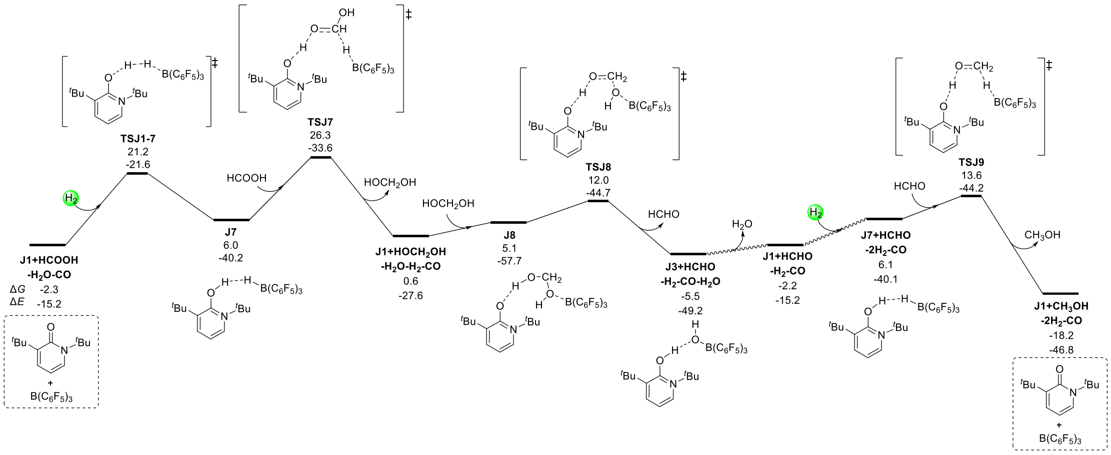

(b)

Figure S14. The pathway for the hydrogenation of CO to methanol by the designed bioinspired FLP **J1**. The relative Gibbs energies (Δ*G*) and potential energies (Δ*E*) are in kcal/mol.

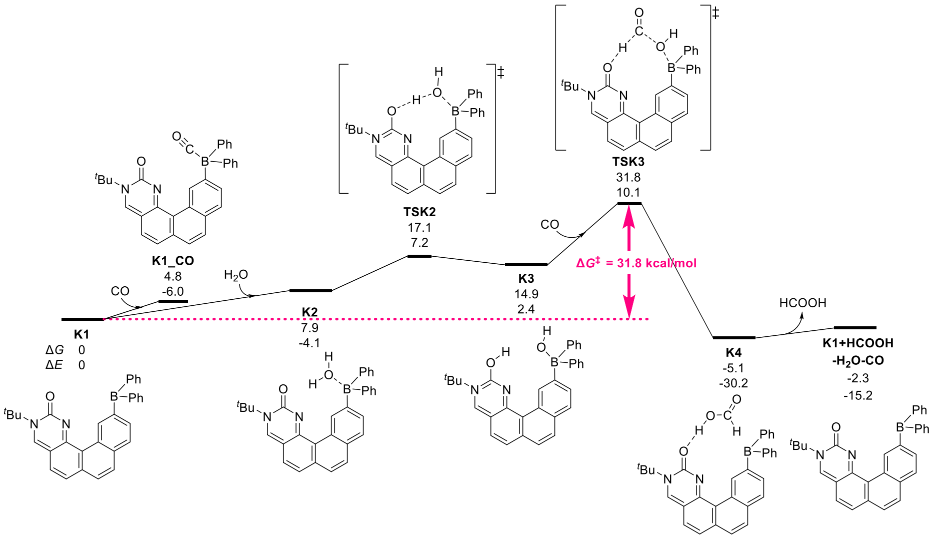

(a)

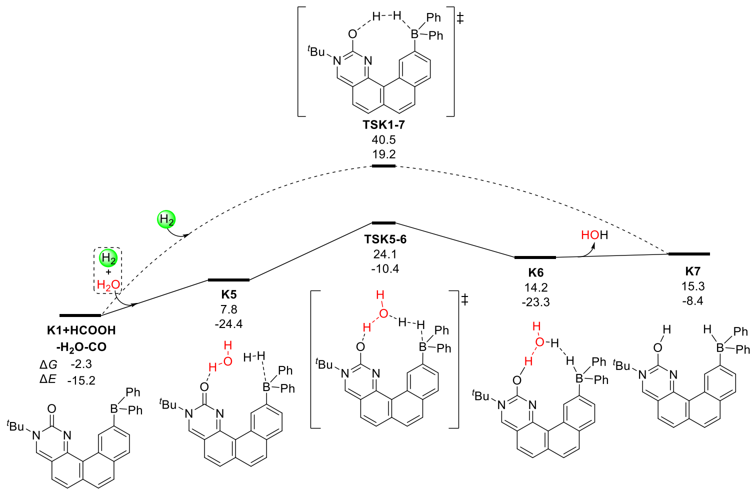

(b)

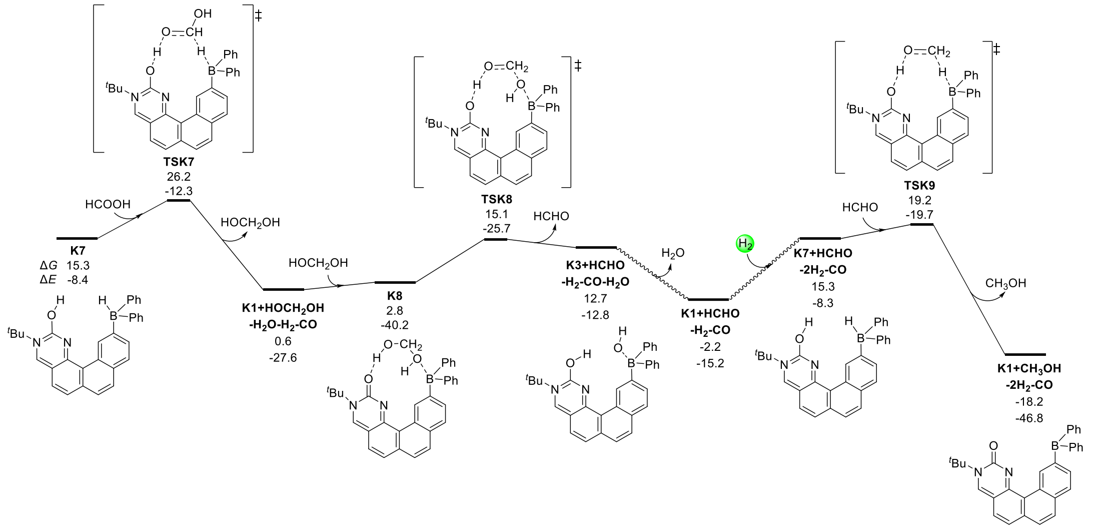

(c)

Figure S15. The pathway for the hydrogenation of CO to methanol by the designed bioinspired FLP **K1**. The relative Gibbs energies (ΔG) and potential energies (ΔE) are in kcal/mol.

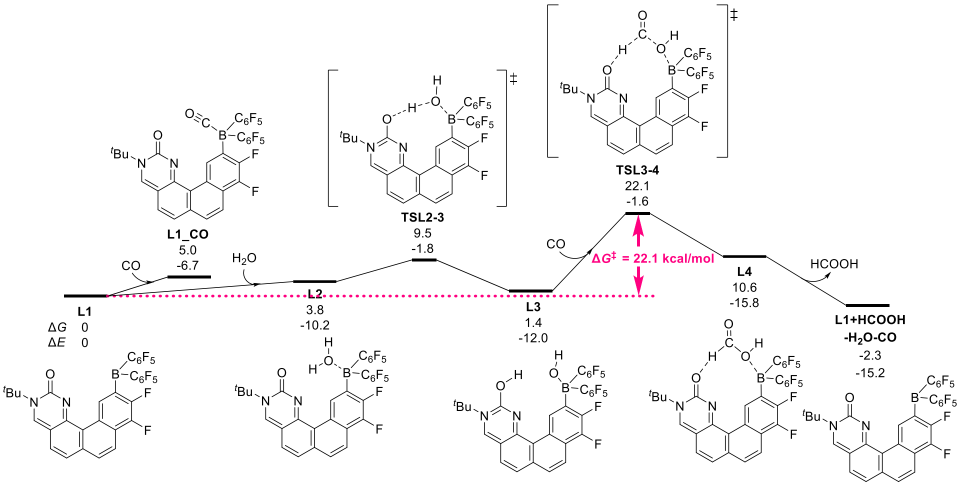

(a)

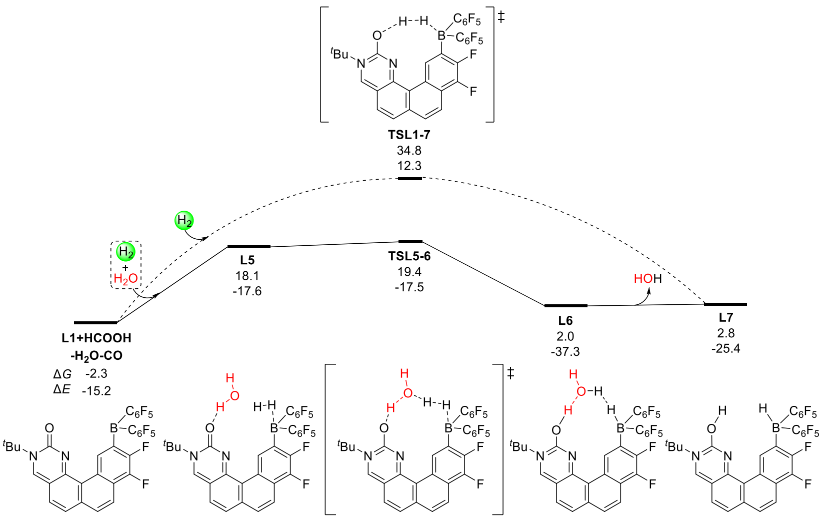

(b)

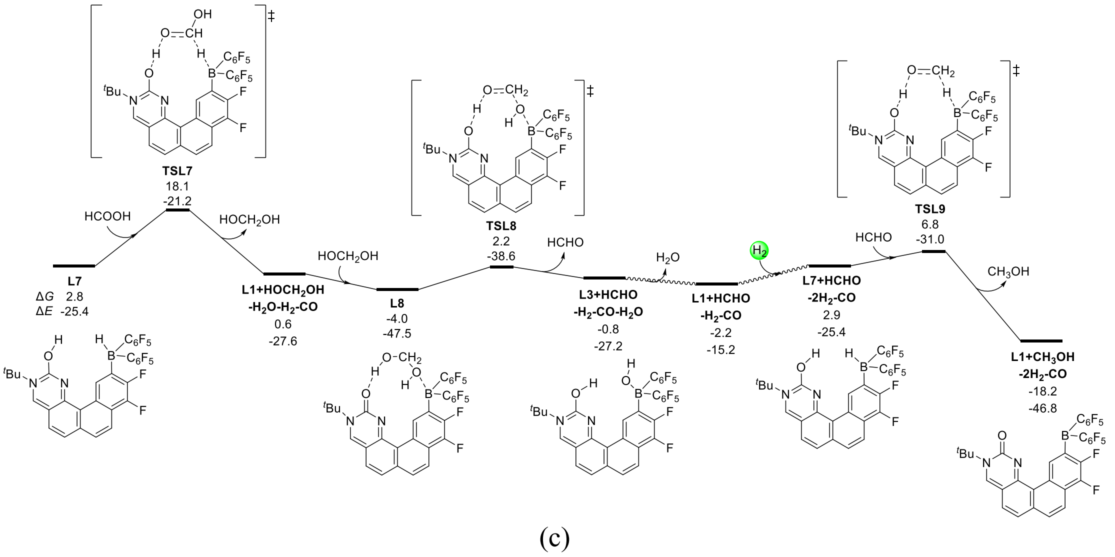

(c)

Figure S16. The pathway for the hydrogenation of CO to methanol by the designed bioinspired FLP **L1**. The relative Gibbs energies (Δ*G*) and potential energies (Δ*E*) are in kcal/mol.

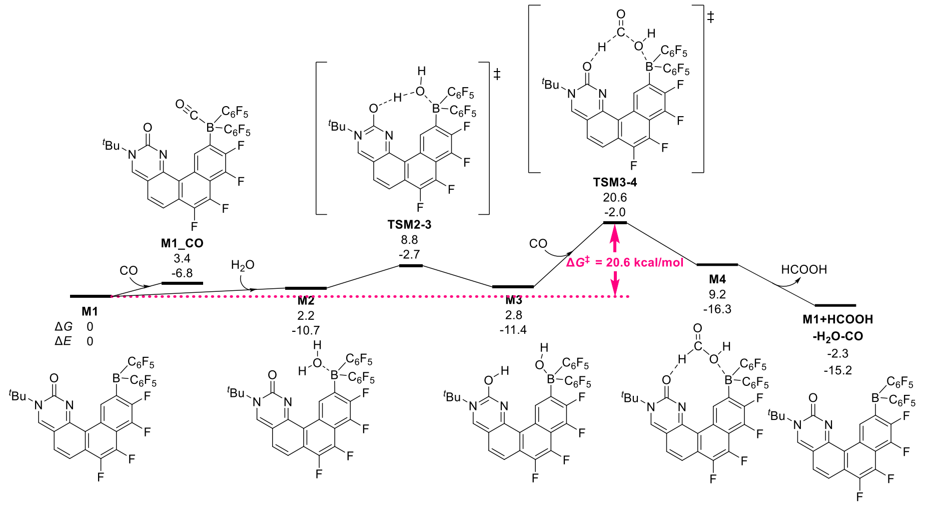

(a)

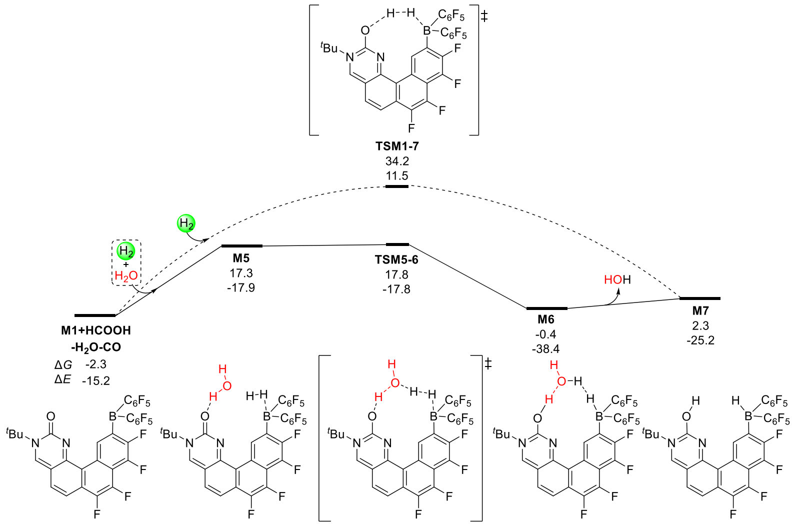

(b)

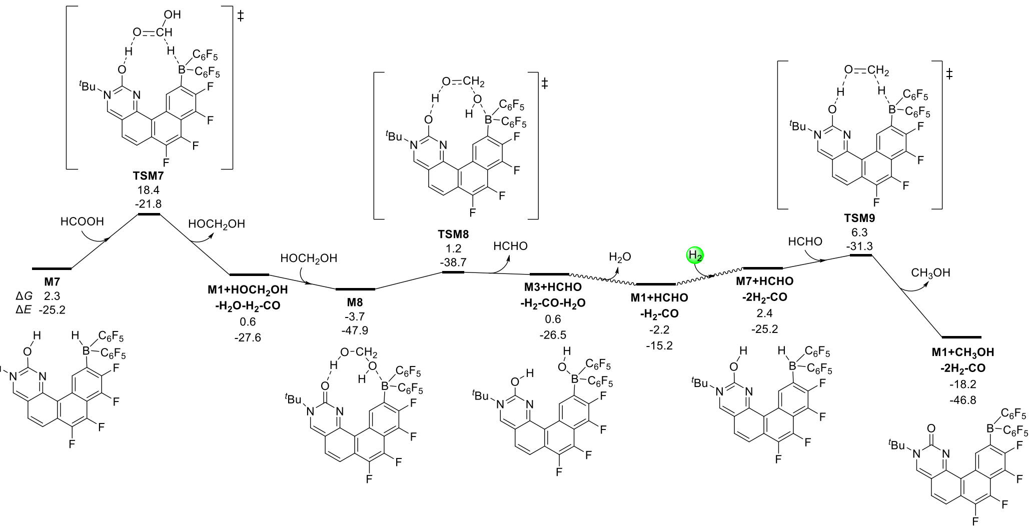

(c)

Figure S17. The pathway for the hydrogenation of CO to methanol by the designed bioinspired FLP **M1**. The relative Gibbs energies (ΔG) and potential energies (ΔE) are in kcal/mol.

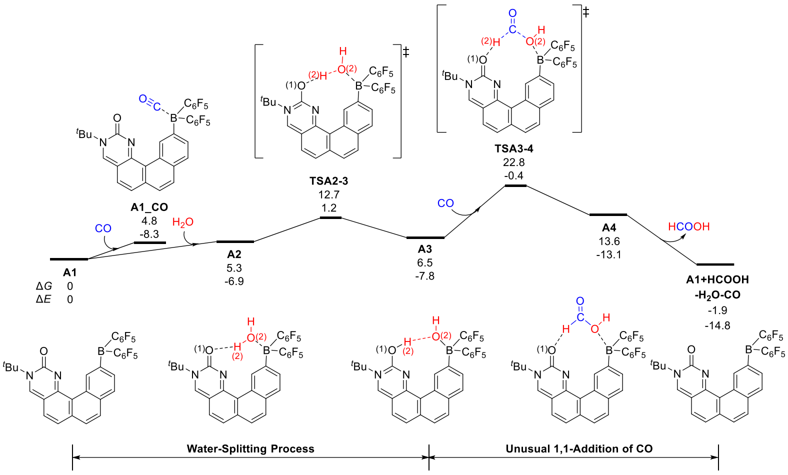

(a)

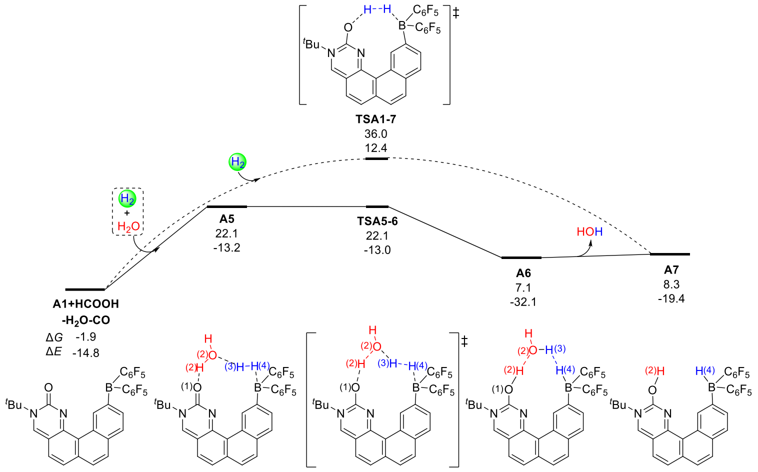

(b)

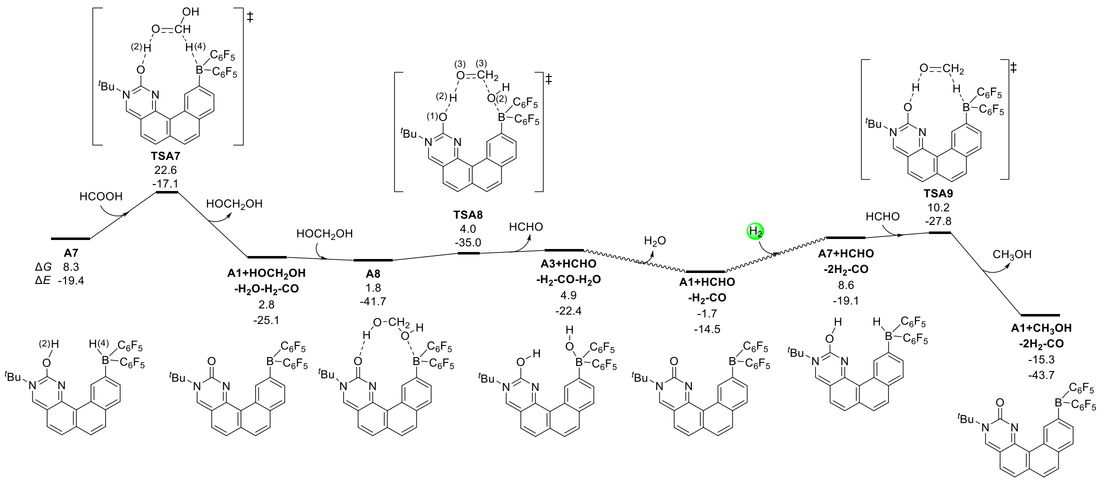

(c)

Figure S18. The pathway for the hydrogenation of CO to methanol by the designed bioinspired FLP **A1** with the B3LYP-D3BJ functional. The relative Gibbs energies (ΔG) and potential energies (ΔE) are in kcal/mol.

As shown in Figure S18, the whole pathway of the hydrogenation of CO to methanol by the designed bioinspired FLP **A1** is computed with the B3LYP-D3BJ

method[1, 2] using the Gaussian 09 program.[3] Geometries were optimized in a dichloromethane solvent using the 6-311G(d,p)[4] basis sets. The single-point energy refinements were further performed with the 6-311++G(2d,p) basis sets. The refined energies were then corrected to Gibbs energies at 298.15 K and 1 atm by using the B3LYP-D3BJ/6-311G(d,p) harmonic frequencies. Solvent effects were evaluated using the SMD (Solution Model based on Density) solvation model.[5, 6] All transition states were demonstrated to exhibit only one imaginary vibrational frequency. Intrinsic reaction coordinate (IRC) analyses were performed to confirm that all transition states connect the two minima in question.[7] The energy span under the B3LYP-D3BJ method is 24 kcal/mol and almost same with that under the ωB97X-D method (25.6 kcal/mol). The conclusion does not change.

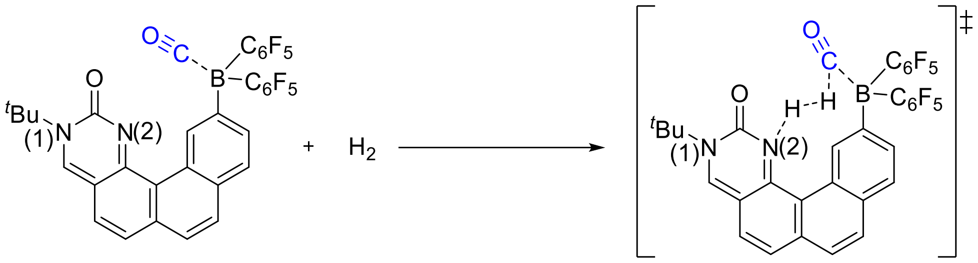

**A1_CO**		**TSA1_CO_H_2**	
Δ*G*	5.2	Δ*G*	40.9
Δ*E*	-8.1	Δ*E*	17.7

Figure S19. The cleavage of H_2 molecule between the N(2) atom and the C atom of CO. The relative Gibbs energies (Δ*G*) and potential energies (Δ*E*) are in kcal/mol.

Cartesian coordinates of all stationary points optimized at ωB97X-D/6-311G(d,p) level in dichloromethane solvent.

H_2O

	Coordinates (Angstroms)		
	X	Y	Z
O	0.000000000	0.118630000	0.000000000
H	0.754776000	-0.474523000	0.000000000
H	-0.754776000	-0.474514000	0.000000000

CO

	Coordinates (Angstroms)		
	X	Y	Z
C	0.000000000	0.000000000	-0.642706000
O	0.000000000	0.000000000	0.482029000

H_2

	Coordinates (Angstroms)		
	X	Y	Z
H	0.000000000	0.000000000	0.372565000
H	0.000000000	0.000000000	-0.372565000

HCOOH

	Coordinates (Angstroms)		
	X	Y	Z
O	-1.044528000	-0.281995000	-0.000004000
C	0.124414000	0.364408000	0.000046000
H	0.032182000	1.462131000	-0.000083000
O	1.168218000	-0.218716000	-0.000015000
H	-1.768183000	0.357106000	-0.000043000

$HOCH_2OH$

	Coordinates (Angstroms)		
	X	Y	Z
O	1.171933000	-0.226462000	-0.141081000
C	-0.000126000	0.503777000	0.094455000
H	-0.000230000	1.318415000	-0.630021000
O	-1.171870000	-0.226502000	-0.140994000
H	-0.000170000	0.916489000	1.112956000
H	-1.315621000	-0.817347000	0.603236000
H	1.316279000	-0.816499000	0.603693000

HCHO

	Coordinates (Angstroms)		
	X	Y	Z
O	0.000000000	0.674171000	0.000000000
C	0.000000000	-0.527548000	0.000000000
H	-0.938187000	-1.114038000	0.000000000
H	0.938187000	-1.114039000	0.000000000

CH_3OH

	Coordinates (Angstroms)		
	X	Y	Z

C	-0.659995000	-0.019038000	0.000000000
H	-1.090801000	0.984482000	-0.000001000
H	-1.026949000	-0.545748000	-0.890875000
H	-1.026949000	-0.545747000	0.890876000
O	0.747277000	0.122515000	0.000000000
H	1.126456000	-0.758882000	0.000000000

A1

	Coordinates (Angstroms)		
	X	Y	Z
B	-2.072081000	0.257350000	0.230563000
C	-3.636207000	0.165923000	0.051814000
C	-4.413420000	-0.617322000	0.895684000
C	-4.309080000	0.824023000	-0.969992000
C	-5.784101000	-0.736557000	0.753407000
C	-5.676532000	0.716076000	-1.151071000
C	-6.416140000	-0.066979000	-0.280993000
C	-1.248154000	-1.087999000	0.317378000
C	-1.323840000	-2.034396000	-0.694642000
C	-0.344003000	-1.353685000	1.339406000
C	-0.529262000	-3.166988000	-0.720138000
C	0.457364000	-2.478621000	1.349735000
C	0.368100000	-3.384547000	0.308499000
F	-3.839895000	-1.275722000	1.909550000
F	-6.497081000	-1.483590000	1.593562000
F	-7.729390000	-0.175680000	-0.438165000
F	-6.285105000	1.350864000	-2.150635000
F	-3.631800000	1.569637000	-1.849000000
F	-0.225466000	-0.504117000	2.367295000
F	1.337367000	-2.683338000	2.329004000
F	1.159569000	-4.449324000	0.287685000
F	-0.606688000	-4.033522000	-1.729666000
F	-2.160456000	-1.844705000	-1.724638000
C	-1.240962000	4.006685000	0.523163000
C	0.165676000	3.990035000	0.358144000
C	0.846871000	2.761680000	0.148674000
C	-1.324328000	1.606804000	0.281588000
C	-1.971662000	2.851501000	0.488502000
C	3.105969000	1.557913000	-0.170515000
C	4.522125000	1.689329000	-0.182731000
C	5.254423000	0.517855000	-0.273747000
C	3.259433000	-0.768018000	-0.499694000
O	2.738606000	-1.850719000	-0.722063000
H	6.333095000	0.567486000	-0.237880000
N	4.691455000	-0.679214000	-0.393092000
C	5.523860000	-1.945535000	-0.433323000
H	-3.046199000	2.890672000	0.633543000
H	-1.727222000	4.962985000	0.685506000
C	2.295608000	2.759226000	0.023537000
C	5.164860000	2.958001000	-0.074570000
C	2.977217000	3.983588000	0.118152000
C	0.901996000	5.207999000	0.415884000
C	2.250849000	5.200330000	0.299963000
C	4.410474000	4.068793000	0.054824000
H	6.248129000	3.006714000	-0.102425000
H	4.867856000	5.048224000	0.133868000
H	2.811941000	6.127075000	0.353373000

H	0.364204000	6.138531000	0.563130000
N	2.547776000	0.365942000	-0.333502000
C	0.052778000	1.594757000	0.110446000
H	0.560002000	0.661112000	-0.065238000
C	5.067529000	-2.860880000	0.708849000
C	5.356173000	-2.603456000	-1.806580000
C	7.005000000	-1.625079000	-0.227234000
H	5.999526000	-3.485586000	-1.854413000
H	4.326738000	-2.908256000	-1.979426000
H	5.664047000	-1.912430000	-2.596566000
H	5.178145000	-2.351213000	1.670304000
H	4.032708000	-3.172399000	0.588110000
H	5.702726000	-3.749861000	0.718754000
H	7.543892000	-2.574073000	-0.230800000
H	7.418816000	-1.013493000	-1.033492000
H	7.197342000	-1.140848000	0.734058000

A1_CO

	Coordinates (Angstroms)		
	X	Y	Z
B	2.022172000	-0.077251000	-0.603641000
C	3.565618000	-0.012136000	-0.113227000
C	3.875315000	0.275443000	1.209276000
C	4.652276000	-0.279695000	-0.930325000
C	5.173598000	0.311404000	1.689750000
C	5.964822000	-0.254269000	-0.492622000
C	6.226190000	0.047580000	0.831250000
C	1.125646000	1.227786000	-0.199769000
C	0.262457000	1.268084000	0.891357000
C	1.160539000	2.384885000	-0.964299000
C	-0.547730000	2.356326000	1.162942000
C	0.371766000	3.495028000	-0.720593000
C	-0.499648000	3.472679000	0.350880000
F	2.901437000	0.523123000	2.092408000
F	5.416296000	0.594288000	2.968388000
F	7.478042000	0.077513000	1.276441000
F	6.968271000	-0.519667000	-1.327141000
F	4.451577000	-0.595096000	-2.223930000
F	1.987797000	2.449137000	-2.027718000
F	0.438146000	4.567748000	-1.508971000
F	-1.293211000	4.510809000	0.593063000
F	-1.394705000	2.326712000	2.191688000
F	0.165896000	0.239513000	1.737532000
C	1.345620000	-3.891818000	0.036123000
C	-0.066427000	-3.946905000	0.021680000
C	-0.818831000	-2.760778000	-0.189391000
C	1.288608000	-1.518865000	-0.374431000
C	2.013046000	-2.705978000	-0.138559000
C	-3.138833000	-1.641295000	-0.168457000
C	-4.535387000	-1.817297000	0.038653000
C	-5.304125000	-0.668032000	0.095403000
C	-3.413318000	0.681900000	-0.436869000
O	-2.971211000	1.778839000	-0.744750000
H	-6.360726000	-0.752423000	0.303843000
N	-4.799876000	0.549409000	-0.079934000
H	3.097277000	-2.686418000	-0.092917000
H	1.894794000	-4.812602000	0.205441000

C	-2.269091000	-2.813881000	-0.102104000
C	-5.112571000	-3.107189000	0.235999000
C	-2.883425000	-4.057082000	0.123328000
C	-0.739192000	-5.186526000	0.225248000
C	-2.091985000	-5.239128000	0.256421000
C	-4.308298000	-4.190134000	0.262623000
H	-6.185471000	-3.193301000	0.370279000
H	-4.715822000	-5.182859000	0.416170000
H	-2.603120000	-6.182804000	0.412839000
H	-0.147954000	-6.086324000	0.360127000
C	-0.088143000	-1.578863000	-0.441518000
H	-0.652273000	-0.685967000	-0.653259000
N	-2.650983000	-0.430319000	-0.401131000
C	-5.656581000	1.790242000	0.064832000
C	-5.006275000	2.714895000	1.101198000
C	-5.787143000	2.462001000	-1.305416000
C	-7.054375000	1.424534000	0.566530000
H	-4.879467000	2.190550000	2.052603000
H	-4.039226000	3.082137000	0.767190000
H	-5.667543000	3.568506000	1.267601000
H	-6.221402000	1.765942000	-2.028961000
H	-6.455629000	3.321837000	-1.215547000
H	-4.821447000	2.802370000	-1.673599000
H	-7.603823000	2.357889000	0.700518000
H	-7.615430000	0.817801000	-0.148681000
H	-7.028013000	0.915341000	1.533626000
C	1.949278000	-0.119407000	-2.214123000
O	1.727977000	-0.278192000	-3.301272000

A2

	Coordinates (Angstroms)		
	X	Y	Z
B	-1.774177000	0.066533000	-0.300914000
C	-3.337231000	0.397787000	-0.617367000
C	-4.406140000	-0.088951000	0.122947000
C	-3.685567000	1.275131000	-1.636562000
C	-5.725309000	0.250700000	-0.136491000
C	-4.986285000	1.636668000	-1.934509000
C	-6.018382000	1.114012000	-1.175446000
C	-1.534827000	-1.348189000	0.463835000
C	-1.158648000	-1.452143000	1.795680000
C	-1.725679000	-2.558534000	-0.184278000
C	-0.957546000	-2.668680000	2.428699000
C	-1.536270000	-3.794079000	0.404471000
C	-1.141284000	-3.846928000	1.729104000
F	-4.207589000	-0.912703000	1.161440000
F	-6.712790000	-0.247661000	0.607294000
F	-7.278619000	1.445588000	-1.438117000
F	-5.252854000	2.478953000	-2.932129000
F	-2.720655000	1.839194000	-2.392698000
F	-2.141044000	-2.565306000	-1.473830000
F	-1.730814000	-4.920241000	-0.281162000
F	-0.950217000	-5.019755000	2.326442000
F	-0.595864000	-2.713461000	3.711504000
F	-1.005507000	-0.361320000	2.555781000
C	-0.850643000	3.471979000	1.359352000
C	0.559289000	3.462171000	1.292569000

C	1.237785000	2.361996000	0.698381000
C	-0.951612000	1.319295000	0.274530000
C	-1.589751000	2.432275000	0.859424000
C	3.517910000	1.326798000	0.061621000
C	4.935170000	1.449894000	0.128470000
C	5.678334000	0.410866000	-0.399218000
C	3.695284000	-0.727010000	-1.080504000
O	3.182318000	-1.675905000	-1.654112000
H	6.756155000	0.457685000	-0.346494000
N	5.129273000	-0.657076000	-0.968630000
H	-2.672875000	2.466516000	0.923853000
H	-1.338191000	4.326638000	1.817938000
C	2.694226000	2.380420000	0.652565000
C	5.571500000	2.579774000	0.722127000
C	3.373095000	3.477552000	1.214148000
C	1.295651000	4.556399000	1.830295000
C	2.648032000	4.560800000	1.795441000
C	4.808364000	3.563381000	1.238256000
H	6.654952000	2.627503000	0.744434000
H	5.258588000	4.439138000	1.691621000
H	3.208838000	5.390166000	2.212229000
H	0.750671000	5.383114000	2.274295000
O	-1.041401000	-0.200564000	-1.753997000
H	-0.901951000	0.611101000	-2.263725000
C	0.430343000	1.305108000	0.208357000
H	0.929730000	0.456663000	-0.232423000
N	2.972753000	0.273810000	-0.532278000
C	5.982398000	-1.791572000	-1.498871000
C	5.592986000	-3.077616000	-0.761530000
C	5.771579000	-1.903984000	-3.012208000
C	7.465580000	-1.523476000	-1.238150000
H	5.742375000	-2.955032000	0.315009000
H	4.556592000	-3.348320000	-0.949966000
H	6.238807000	-3.888935000	-1.105927000
H	6.027000000	-0.959338000	-3.500978000
H	6.434431000	-2.680042000	-3.402747000
H	4.743208000	-2.163224000	-3.252821000
H	8.019925000	-2.382740000	-1.619552000
H	7.832099000	-0.638738000	-1.765361000
H	7.693198000	-1.433048000	-0.172636000
H	-1.454728000	-0.867844000	-2.322492000

A3

	Coordinates (Angstroms)		
	X	Y	Z
B	1.818629000	-0.016404000	-0.634861000
C	1.368991000	-1.284938000	0.367752000
C	1.886993000	-1.413342000	1.651327000
C	0.327644000	-2.156788000	0.102027000
C	1.430225000	-2.318655000	2.591047000
C	-0.178175000	-3.074983000	1.010359000
C	0.379173000	-3.160826000	2.269348000
C	3.464804000	0.120520000	-0.814956000
C	3.990271000	1.287212000	-1.360134000
C	4.404247000	-0.876490000	-0.598277000
C	5.335880000	1.480988000	-1.624143000
C	5.760842000	-0.729495000	-0.847772000

C	6.233363000	0.462097000	-1.361605000
F	2.892245000	-0.610302000	2.048127000
F	1.981038000	-2.384578000	3.806540000
F	-0.085202000	-4.036997000	3.161472000
F	-1.201570000	-3.869509000	0.675130000
F	-0.309468000	-2.158798000	-1.103600000
F	4.036324000	-2.086447000	-0.132871000
F	6.613413000	-1.731729000	-0.606750000
F	7.533810000	0.624546000	-1.610999000
F	5.776471000	2.635269000	-2.136560000
F	3.184433000	2.316482000	-1.674899000
C	1.093764000	3.173174000	1.617136000
C	-0.303125000	3.298540000	1.457159000
C	-1.002659000	2.348821000	0.664245000
C	1.136595000	1.257571000	0.132988000
C	1.788124000	2.164854000	0.995470000
C	-3.308249000	1.441897000	0.026792000
C	-4.709377000	1.653729000	-0.051579000
C	-5.478504000	0.643574000	-0.600555000
C	-3.597284000	-0.651230000	-0.892752000
O	-3.087117000	-1.794762000	-1.294338000
H	-6.545485000	0.773072000	-0.690368000
N	-4.959771000	-0.513000000	-1.031357000
H	2.859607000	2.085925000	1.152027000
H	1.609604000	3.898460000	2.239231000
C	-2.452422000	2.448522000	0.590642000
C	-5.306549000	2.848538000	0.448747000
C	-3.095780000	3.568220000	1.162170000
C	-1.004728000	4.381833000	2.058121000
C	-2.341931000	4.535675000	1.885468000
C	-4.513842000	3.758652000	1.053235000
H	-6.376716000	2.990048000	0.356036000
H	-4.937444000	4.661957000	1.477276000
H	-2.864392000	5.380580000	2.319578000
H	-0.441530000	5.100786000	2.644679000
O	1.212648000	-0.106955000	-1.963741000
H	-2.123426000	-1.770127000	-1.160682000
N	-2.821457000	0.258192000	-0.400638000
C	-0.233446000	1.381029000	-0.013451000
H	-0.723165000	0.690948000	-0.678253000
C	-5.844495000	-1.617879000	-1.628001000
C	-5.400058000	-1.866812000	-3.071162000
C	-5.729351000	-2.862154000	-0.743551000
C	-7.302124000	-1.161761000	-1.638183000
H	-5.443962000	-0.939591000	-3.648190000
H	-4.396604000	-2.282023000	-3.135862000
H	-6.093575000	-2.581094000	-3.519605000
H	-5.997747000	-2.623258000	0.288814000
H	-6.435649000	-3.607227000	-1.115412000
H	-4.733609000	-3.299467000	-0.760978000
H	-7.882836000	-1.976906000	-2.072106000
H	-7.693388000	-0.982166000	-0.633637000
H	-7.461292000	-0.279405000	-2.262782000
H	1.466488000	-0.923087000	-2.394659000

A4

Coordinates (Angstroms)

	X	Y	Z
B	-1.642266000	-0.021650000	0.000813000
C	-2.401878000	1.182300000	-0.798832000
C	-3.679263000	1.594746000	-0.439131000
C	-1.804138000	1.956863000	-1.779935000
C	-4.325018000	2.670338000	-1.026528000
C	-2.409494000	3.032643000	-2.401845000
C	-3.688903000	3.391819000	-2.020055000
C	-2.602953000	-1.269146000	0.365317000
C	-2.660245000	-1.895626000	1.602066000
C	-3.432557000	-1.811315000	-0.609954000
C	-3.502382000	-2.963940000	1.868318000
C	-4.279628000	-2.880899000	-0.386552000
C	-4.318055000	-3.459769000	0.869293000
F	-4.352664000	0.960666000	0.532926000
F	-5.551571000	3.016476000	-0.640053000
F	-4.297020000	4.423557000	-2.596206000
F	-1.776135000	3.720348000	-3.349161000
F	-0.538536000	1.674190000	-2.186130000
F	-3.431050000	-1.299642000	-1.851842000
F	-5.053229000	-3.357711000	-1.363033000
F	-5.125762000	-4.488475000	1.109458000
F	-3.522522000	-3.525618000	3.078216000
F	-1.881131000	-1.502389000	2.615127000
C	-0.446504000	1.926211000	3.135457000
C	0.963973000	1.866533000	3.039221000
C	1.564345000	1.180171000	1.953264000
C	-0.681208000	0.614966000	1.117370000
C	-1.247867000	1.323268000	2.205078000
C	3.753446000	0.443583000	0.801648000
C	5.175551000	0.459511000	0.841724000
C	5.838590000	-0.197379000	-0.178121000
C	3.778720000	-0.820518000	-1.186935000
O	3.183439000	-1.392418000	-2.097555000
H	6.918600000	-0.206890000	-0.181327000
N	5.210047000	-0.825685000	-1.168133000
H	-2.326596000	1.385864000	2.309717000
H	-0.884028000	2.462169000	3.971817000
C	3.014403000	1.124423000	1.862629000
C	5.896603000	1.119194000	1.881812000
C	3.773386000	1.759189000	2.864186000
C	1.776959000	2.494919000	4.023473000
C	3.128077000	2.442468000	3.937040000
C	5.211308000	1.747436000	2.857474000
H	6.981016000	1.101139000	1.866198000
H	5.728438000	2.256478000	3.662825000
H	3.748611000	2.920004000	4.687568000
H	1.294650000	3.013922000	4.845521000
O	-0.611403000	-0.712086000	-1.173836000
H	1.126824000	-1.731589000	-1.657498000
N	3.132314000	-0.182777000	-0.193464000
C	0.690874000	0.579777000	1.012987000
H	1.168905000	0.092174000	0.184273000
C	0.260325000	-1.802279000	-0.983219000
O	0.020742000	-2.631269000	-0.180685000
C	5.979807000	-1.536369000	-2.265763000
C	5.609631000	-3.022913000	-2.240973000

C	5.640921000	-0.875793000	-3.606049000
C	7.486794000	-1.413755000	-2.036282000
H	5.838934000	-3.454611000	-1.262626000
H	4.555835000	-3.178035000	-2.460242000
H	6.206732000	-3.545122000	-2.992459000
H	5.891122000	0.188621000	-3.577801000
H	6.238095000	-1.346631000	-4.390520000
H	4.587199000	-0.988739000	-3.850822000
H	7.980619000	-1.941529000	-2.853995000
H	7.832821000	-0.377156000	-2.062344000
H	7.808203000	-1.883510000	-1.103049000
H	-0.238530000	-0.069450000	-1.804340000

A5

	Coordinates (Angstroms)		
	X	Y	Z
B	-1.685689000	-0.106331000	0.071229000
C	-2.918161000	-0.794860000	0.869408000
C	-4.177757000	-0.906969000	0.299168000
C	-2.774444000	-1.381886000	2.117386000
C	-5.234644000	-1.546358000	0.925931000
C	-3.805395000	-2.020700000	2.782229000
C	-5.047689000	-2.102743000	2.178411000
C	-2.084528000	1.280747000	-0.657760000
C	-1.943912000	1.518535000	-2.017206000
C	-2.625199000	2.326830000	0.080599000
C	-2.315601000	2.715547000	-2.610787000
C	-3.001579000	3.535615000	-0.473538000
C	-2.845500000	3.729471000	-1.834994000
F	-4.414152000	-0.410162000	-0.924435000
F	-6.424582000	-1.634934000	0.332215000
F	-6.052600000	-2.717794000	2.794761000
F	-3.613604000	-2.557858000	3.986194000
F	-1.582668000	-1.337969000	2.738326000
F	-2.784295000	2.187528000	1.404451000
F	-3.508254000	4.508868000	0.282999000
F	-3.204308000	4.882238000	-2.391536000
F	-2.169466000	2.894342000	-3.924159000
F	-1.451392000	0.582737000	-2.836084000
C	-0.515276000	-3.104401000	-2.101016000
C	0.892962000	-3.019843000	-2.018184000
C	1.496522000	-1.983420000	-1.257190000
C	-0.750730000	-1.159215000	-0.691242000
C	-1.318822000	-2.208139000	-1.448686000
C	3.696114000	-0.936473000	-0.416473000
C	5.113084000	-1.036696000	-0.350227000
C	5.770252000	-0.095435000	0.419857000
C	3.726126000	0.988353000	0.928475000
O	3.128369000	1.920328000	1.476837000
H	6.844250000	-0.149306000	0.515372000
N	5.140057000	0.884282000	1.065961000
H	-2.396982000	-2.304616000	-1.528021000
H	-0.950001000	-3.906996000	-2.688386000
C	2.948687000	-1.937114000	-1.167423000
C	5.827849000	-2.064312000	-1.035933000
C	3.701167000	-2.923245000	-1.832022000
C	1.700727000	-3.981723000	-2.689069000

C	3.050969000	-3.935478000	-2.598004000
C	5.136957000	-2.967028000	-1.760468000
H	6.909228000	-2.098508000	-0.961327000
H	5.648107000	-3.758340000	-2.296961000
H	3.665622000	-4.672564000	-3.103056000
H	1.211584000	-4.757843000	-3.268803000
N	3.080574000	0.062968000	0.205717000
C	0.624043000	-1.068624000	-0.615047000
H	1.071564000	-0.269930000	-0.047848000
O	0.487869000	2.076578000	0.746832000
H	1.410817000	1.961567000	1.063349000
H	-0.625263000	0.736659000	0.895843000
H	-0.979557000	0.118176000	1.280362000
C	5.899486000	1.880181000	1.925010000
C	5.728272000	3.277985000	1.322509000
C	5.363198000	1.794858000	3.357706000
C	7.390764000	1.542490000	1.950308000
H	6.097689000	3.295486000	0.293252000
H	4.687991000	3.595340000	1.335068000
H	6.319267000	3.985441000	1.909107000
H	5.475824000	0.778515000	3.745642000
H	5.948944000	2.466487000	3.989671000
H	4.316405000	2.084664000	3.414932000
H	7.876041000	2.279917000	2.591942000
H	7.589372000	0.556042000	2.377673000
H	7.855274000	1.614383000	0.963519000
H	0.578185000	2.302418000	-0.183541000

A6

	Coordinates (Angstroms)		
	X	Y	Z
B	-1.858863000	-0.060468000	-0.499588000
C	-1.482069000	1.167726000	0.523440000
C	-1.130690000	1.025818000	1.860405000
C	-1.472868000	2.478969000	0.068090000
C	-0.778145000	2.089969000	2.676434000
C	-1.126094000	3.572377000	0.843484000
C	-0.774550000	3.374497000	2.165368000
C	-3.478490000	-0.233120000	-0.643724000
C	-4.098818000	-0.335888000	-1.881393000
C	-4.339337000	-0.310252000	0.442193000
C	-5.467273000	-0.488353000	-2.043207000
C	-5.711357000	-0.462529000	0.329530000
C	-6.281326000	-0.549442000	-0.927364000
F	-1.116369000	-0.181230000	2.445915000
F	-0.445017000	1.888387000	3.955419000
F	-0.437261000	4.408527000	2.937710000
F	-1.124123000	4.807348000	0.331425000
F	-1.800877000	2.751412000	-1.214132000
F	-3.850688000	-0.256700000	1.694020000
F	-6.487266000	-0.532765000	1.415957000
F	-7.600171000	-0.697400000	-1.061494000
F	-6.008817000	-0.576847000	-3.262405000
F	-3.376692000	-0.296613000	-3.015346000
C	-1.025824000	-3.805927000	0.400919000
C	0.380585000	-3.774826000	0.495132000
C	1.076042000	-2.563311000	0.224221000

C	-1.091051000	-1.443006000	-0.136886000
C	-1.739082000	-2.675588000	0.086843000
C	3.382271000	-1.468621000	-0.056668000
C	4.784023000	-1.551115000	0.151360000
C	5.546865000	-0.452779000	-0.199513000
C	3.656931000	0.645072000	-0.950564000
O	3.155769000	1.709466000	-1.506055000
H	6.613233000	-0.472352000	-0.039545000
N	5.024289000	0.651354000	-0.742481000
H	-2.821947000	-2.734099000	0.026895000
H	-1.534749000	-4.744498000	0.598680000
C	2.531741000	-2.571291000	0.310155000
C	5.392289000	-2.727432000	0.679735000
C	3.184694000	-3.739142000	0.762381000
C	1.093963000	-4.941099000	0.891235000
C	2.440250000	-4.919326000	1.046310000
C	4.606350000	-3.789514000	0.951313000
H	6.464801000	-2.751099000	0.833096000
H	5.034152000	-4.711121000	1.328908000
H	2.977120000	-5.801204000	1.376831000
H	0.533064000	-5.848742000	1.090333000
H	2.179361000	1.584029000	-1.718147000
N	2.888296000	-0.350006000	-0.622736000
C	0.293687000	-1.426983000	-0.070311000
H	0.798426000	-0.489288000	-0.236905000
H	-1.460429000	0.280814000	-1.601530000
O	0.714612000	1.547079000	-2.295042000
H	0.087115000	0.858066000	-2.027237000
C	5.906559000	1.844392000	-1.131154000
C	5.789362000	2.067841000	-2.641563000
C	5.463905000	3.058841000	-0.312063000
C	7.367188000	1.538710000	-0.803278000
H	6.040285000	1.151552000	-3.182358000
H	4.797246000	2.398044000	-2.939330000
H	6.508421000	2.838440000	-2.926743000
H	5.555831000	2.851567000	0.757246000
H	6.125017000	3.893112000	-0.556069000
H	4.441466000	3.358136000	-0.532434000
H	7.944067000	2.420138000	-1.087220000
H	7.534322000	1.370418000	0.263411000
H	7.756458000	0.694718000	-1.378189000
H	0.197057000	2.358832000	-2.295267000

A7

	Coordinates (Angstroms)		
	X	Y	Z
B	1.898111000	-0.032752000	-0.608415000
C	1.399304000	-1.278904000	0.338189000
C	1.854625000	-1.454383000	1.638646000
C	0.359612000	-2.119635000	-0.014436000
C	1.348254000	-2.396946000	2.514413000
C	-0.194483000	-3.071536000	0.827339000
C	0.308508000	-3.215637000	2.105087000
C	3.515433000	0.088043000	-0.831362000
C	4.036183000	1.223428000	-1.444403000
C	4.464198000	-0.886382000	-0.551926000
C	5.380251000	1.414808000	-1.715398000

C	5.820011000	-0.740121000	-0.805669000
C	6.285830000	0.422598000	-1.387716000
F	2.838847000	-0.668376000	2.109842000
F	1.836962000	-2.520508000	3.751477000
F	-0.203358000	-4.124813000	2.936288000
F	-1.213080000	-3.836658000	0.417593000
F	-0.222098000	-2.027125000	-1.243585000
F	4.106064000	-2.069405000	-0.017946000
F	6.680063000	-1.717494000	-0.499551000
F	7.586493000	0.581183000	-1.640466000
F	5.809520000	2.539119000	-2.298496000
F	3.213750000	2.216271000	-1.831201000
C	1.173408000	3.264398000	1.464733000
C	-0.229924000	3.360363000	1.347091000
C	-0.940238000	2.368020000	0.617045000
C	1.202570000	1.270351000	0.085262000
C	1.865646000	2.238832000	0.870285000
C	-3.257601000	1.446807000	0.017133000
C	-4.663750000	1.640799000	-0.008640000
C	-5.439638000	0.634288000	-0.555034000
C	-3.554924000	-0.623754000	-0.954338000
O	-3.044886000	-1.746645000	-1.411403000
H	-6.510461000	0.752847000	-0.602565000
N	-4.923066000	-0.502163000	-1.038059000
H	2.942705000	2.183705000	0.997787000
H	1.695446000	4.024260000	2.038563000
C	-2.394355000	2.454039000	0.571328000
C	-5.260295000	2.820536000	0.525534000
C	-3.033953000	3.574201000	1.148799000
C	-0.926488000	4.450919000	1.938940000
C	-2.272560000	4.571239000	1.821347000
C	-4.457706000	3.743343000	1.095610000
H	-6.335476000	2.944963000	0.476711000
H	-4.877022000	4.641858000	1.533658000
H	-2.794679000	5.415813000	2.256299000
H	-0.354329000	5.198741000	2.478861000
H	-2.078397000	-1.714474000	-1.308170000
N	-2.773854000	0.282211000	-0.463916000
C	-0.174625000	1.372133000	-0.023798000
H	-0.685036000	0.634048000	-0.618428000
H	1.412617000	-0.180615000	-1.715978000
C	-5.814165000	-1.601005000	-1.636760000
C	-5.415328000	-1.803870000	-3.100439000
C	-5.654110000	-2.867598000	-0.792377000
C	-7.276321000	-1.162371000	-1.588145000
H	-5.492846000	-0.862194000	-3.649759000
H	-4.409227000	-2.202177000	-3.211274000
H	-6.113842000	-2.516174000	-3.544159000
H	-5.885184000	-2.660375000	0.255664000
H	-6.366741000	-3.609588000	-1.158179000
H	-4.655691000	-3.294228000	-0.859504000
H	-7.861192000	-1.970934000	-2.028679000
H	-7.638086000	-1.017312000	-0.567131000
H	-7.464278000	-0.263343000	-2.180247000

A8

Coordinates (Angstroms)

	X	Y	Z
B	-1.631554000	-0.004198000	-0.113819000
C	-2.745321000	0.976400000	-0.816505000
C	-4.058330000	1.094949000	-0.383522000
C	-2.391651000	1.856829000	-1.829424000
C	-4.967044000	1.986565000	-0.931052000
C	-3.263630000	2.760905000	-2.408946000
C	-4.569431000	2.822105000	-1.957607000
C	-2.256431000	-1.363952000	0.521005000
C	-1.959856000	-1.850345000	1.786919000
C	-3.145302000	-2.143378000	-0.211532000
C	-2.516774000	-3.013577000	2.298948000
C	-3.716982000	-3.310088000	0.258533000
C	-3.398932000	-3.748492000	1.531857000
F	-4.515606000	0.346042000	0.634080000
F	-6.218096000	2.048891000	-0.472677000
F	-5.428123000	3.684101000	-2.496898000
F	-2.857669000	3.574817000	-3.384334000
F	-1.121124000	1.885569000	-2.288925000
F	-3.475243000	-1.780371000	-1.463744000
F	-4.558990000	-4.015809000	-0.498175000
F	-3.934230000	-4.868701000	2.008959000
F	-2.202361000	-3.431307000	3.526396000
F	-1.110276000	-1.213062000	2.599277000
C	-0.528807000	2.635851000	2.527327000
C	0.881989000	2.620647000	2.429192000
C	1.513521000	1.713702000	1.539087000
C	-0.705852000	0.905526000	0.844492000
C	-1.301154000	1.813570000	1.752288000
C	3.739917000	0.828815000	0.599374000
C	5.153392000	0.969521000	0.544887000
C	5.832409000	0.114385000	-0.302498000
C	3.818513000	-0.971519000	-0.903669000
O	3.235589000	-1.876484000	-1.517552000
H	6.904556000	0.202075000	-0.393768000
N	5.225756000	-0.825374000	-1.029196000
H	-2.382148000	1.860575000	1.844225000
H	-0.989329000	3.329651000	3.223821000
C	2.965120000	1.724057000	1.446302000
C	5.840247000	1.948381000	1.325646000
C	3.691096000	2.652128000	2.215867000
C	1.662214000	3.519667000	3.209642000
C	3.012971000	3.540491000	3.101111000
C	5.124749000	2.749796000	2.140381000
H	6.919651000	2.024061000	1.253670000
H	5.615025000	3.495915000	2.755553000
H	3.605145000	4.233672000	3.688490000
H	1.152330000	4.196771000	3.887504000
O	-0.715330000	-0.528350000	-1.348870000
N	3.150410000	-0.121947000	-0.117163000
C	0.669873000	0.868698000	0.773496000
H	1.150876000	0.158999000	0.125343000
C	-0.159287000	-1.889198000	-1.586094000
H	0.270844000	-1.811857000	-2.586710000
H	-1.037806000	-2.527513000	-1.588305000
O	0.716202000	-2.276151000	-0.634774000
H	1.636623000	-2.063057000	-0.916889000

C	6.010775000	-1.737644000	-1.955589000
C	5.876634000	-3.177915000	-1.451953000
C	5.471190000	-1.567203000	-3.379254000
C	7.491794000	-1.358479000	-1.955453000
H	6.240487000	-3.256782000	-0.423647000
H	4.846067000	-3.523631000	-1.493495000
H	6.491040000	-3.826120000	-2.081462000
H	5.560114000	-0.524228000	-3.696235000
H	6.071976000	-2.181004000	-4.054370000
H	4.431703000	-1.877934000	-3.456740000
H	7.996197000	-2.032069000	-2.650220000
H	7.661641000	-0.337245000	-2.306600000
H	7.959769000	-1.491213000	-0.976907000
H	-0.106110000	0.152335000	-1.665252000

TSA2-3

	Coordinates (Angstroms)		
	X	Y	Z
B	1.273260000	0.144687000	-0.088350000
C	1.748272000	-0.921234000	1.071681000
C	3.015046000	-0.932746000	1.638675000
C	0.856001000	-1.828533000	1.630342000
C	3.394952000	-1.796988000	2.653342000
C	1.194502000	-2.712846000	2.641085000
C	2.478305000	-2.701193000	3.154324000
C	2.523424000	0.585317000	-1.065516000
C	3.004310000	1.878113000	-1.217265000
C	3.197355000	-0.368185000	-1.816560000
C	4.072507000	2.199434000	-2.043027000
C	4.263011000	-0.097532000	-2.653201000
C	4.706653000	1.207888000	-2.766309000
F	3.955862000	-0.059078000	1.236260000
F	4.632267000	-1.760066000	3.154502000
F	2.823100000	-3.542221000	4.128419000
F	0.292769000	-3.567790000	3.131201000
F	-0.421371000	-1.875069000	1.217356000
F	2.806258000	-1.662902000	-1.747304000
F	4.860818000	-1.068588000	-3.345846000
F	5.730138000	1.504227000	-3.564515000
F	4.493992000	3.461930000	-2.145744000
F	2.465518000	2.907291000	-0.548918000
C	-0.015790000	2.813584000	2.552172000
C	-1.322089000	3.052956000	2.050304000
C	-1.684330000	2.421471000	0.844769000
C	0.490701000	1.359367000	0.652595000
C	0.812692000	1.910157000	1.924167000
C	-3.517968000	1.110152000	-0.184635000
C	-4.887313000	0.815933000	-0.359755000
C	-5.157207000	-0.379368000	-1.008775000
C	-2.869034000	-0.895148000	-1.151005000
O	-1.893063000	-1.663756000	-1.435783000
H	-6.179927000	-0.671048000	-1.191585000
N	-4.198399000	-1.235366000	-1.418961000
H	1.752459000	1.643061000	2.399748000
H	0.288817000	3.289703000	3.478978000
C	-3.083405000	2.261245000	0.555616000
C	-5.865669000	1.701836000	0.207231000

C	-4.054410000	2.974868000	1.260001000
C	-2.319549000	3.822266000	2.727707000
C	-3.620770000	3.840110000	2.310440000
C	-5.453080000	2.724999000	0.996677000
H	-6.920059000	1.514619000	0.038309000
H	-6.183727000	3.361612000	1.484061000
H	-4.358102000	4.430655000	2.843455000
H	-2.030360000	4.385413000	3.609825000
O	0.279465000	-0.601124000	-1.076190000
H	-0.830464000	-1.082993000	-1.124076000
N	-2.602318000	0.243075000	-0.576790000
C	-0.686777000	1.771562000	0.086160000
H	-0.905004000	1.471105000	-0.920560000
C	-4.541098000	-2.548790000	-2.108390000
C	-3.854393000	-2.562850000	-3.476892000
C	-4.070384000	-3.694875000	-1.208657000
C	-6.049262000	-2.671902000	-2.316369000
H	-4.182795000	-1.709950000	-4.077098000
H	-2.770128000	-2.540313000	-3.387135000
H	-4.139480000	-3.478855000	-3.998811000
H	-4.565951000	-3.641410000	-0.235634000
H	-4.341179000	-4.641875000	-1.680715000
H	-2.992080000	-3.678033000	-1.060194000
H	-6.224096000	-3.620106000	-2.827547000
H	-6.601791000	-2.703903000	-1.374006000
H	-6.449142000	-1.876956000	-2.951249000
H	0.795132000	-1.183084000	-1.642257000

TSA3-4

	Coordinates (Angstroms)		
	X	Y	Z
B	1.533522000	0.046643000	-0.089230000
C	2.373371000	-1.141439000	-0.874496000
C	3.639586000	-1.551465000	-0.477459000
C	1.830415000	-1.895412000	-1.903459000
C	4.330775000	-2.595942000	-1.069303000
C	2.480394000	-2.945356000	-2.528652000
C	3.749074000	-3.297449000	-2.109074000
C	2.504264000	1.274192000	0.396357000
C	2.470425000	1.879351000	1.643326000
C	3.397897000	1.844360000	-0.502748000
C	3.280792000	2.949359000	1.993591000
C	4.222654000	2.911616000	-0.195847000
C	4.165576000	3.468206000	1.069108000
F	4.266988000	-0.938200000	0.542917000
F	5.549332000	-2.934175000	-0.643027000
F	4.397819000	-4.305037000	-2.690440000
F	1.894672000	-3.620181000	-3.519488000
F	0.575832000	-1.639795000	-2.351565000
F	3.500539000	1.360447000	-1.752561000
F	5.067376000	3.410206000	-1.102967000
F	4.949946000	4.496802000	1.388629000
F	3.204554000	3.490629000	3.212901000
F	1.616181000	1.469088000	2.593204000
C	0.453130000	-2.117998000	3.033756000
C	-0.959922000	-2.100343000	2.921131000
C	-1.555983000	-1.363838000	1.869200000

C	0.680840000	-0.711173000	1.070837000
C	1.249148000	-1.442623000	2.143732000
C	-3.717499000	-0.541433000	0.782856000
C	-5.130019000	-0.619172000	0.699287000
C	-5.735571000	0.133943000	-0.291538000
C	-3.666938000	0.984844000	-0.978425000
O	-2.979337000	1.734306000	-1.740862000
H	-6.807514000	0.096027000	-0.409127000
N	-5.054802000	0.920160000	-1.135608000
H	2.327795000	-1.475610000	2.266225000
H	0.897441000	-2.683985000	3.846889000
C	-3.001619000	-1.329160000	1.761194000
C	-5.875709000	-1.437702000	1.603612000
C	-3.776840000	-2.087083000	2.657147000
C	-1.785944000	-2.824609000	3.827419000
C	-3.138211000	-2.831947000	3.691192000
C	-5.211751000	-2.129933000	2.554569000
H	-6.955281000	-1.481096000	1.515745000
H	-5.752482000	-2.746430000	3.263946000
H	-3.758554000	-3.401802000	4.374222000
H	-1.313740000	-3.384363000	4.628635000
O	0.579500000	0.734109000	-1.101870000
H	-1.776113000	1.808980000	-1.316722000
N	-3.068067000	0.274470000	-0.048816000
C	-0.688409000	-0.715413000	0.962821000
H	-1.142626000	-0.201520000	0.142252000
C	-0.618337000	2.073298000	-0.727370000
O	-0.146683000	2.853164000	-0.035899000
C	-5.761298000	1.714030000	-2.230582000
C	-5.511124000	3.204863000	-1.988583000
C	-5.226540000	1.240092000	-3.584674000
C	-7.266865000	1.458214000	-2.184296000
H	-5.862279000	3.493559000	-0.994222000
H	-4.458790000	3.462492000	-2.085248000
H	-6.077836000	3.774053000	-2.728770000
H	-5.401982000	0.168113000	-3.709095000
H	-5.766304000	1.767538000	-4.374246000
H	-4.163900000	1.443910000	-3.699110000
H	-7.715051000	2.042637000	-2.989301000
H	-7.518507000	0.409566000	-2.362690000
H	-7.719828000	1.792050000	-1.247205000
H	0.122456000	0.098287000	-1.662194000

TSA1-7

	Coordinates (Angstroms)		
	X	Y	Z
B	-1.359720000	-0.330560000	-0.352433000
C	-2.443821000	-0.992039000	0.631602000
C	-3.811726000	-0.802942000	0.495648000
C	-2.057479000	-1.850842000	1.653331000
C	-4.743360000	-1.408301000	1.323954000
C	-2.956137000	-2.468856000	2.504716000
C	-4.311395000	-2.244127000	2.337009000
C	-1.715265000	1.109051000	-0.972860000
C	-1.902199000	1.321811000	-2.331969000
C	-1.864576000	2.232326000	-0.169033000
C	-2.185518000	2.567340000	-2.869273000

C	-2.140951000	3.493024000	-0.667525000
C	-2.300957000	3.661134000	-2.031373000
F	-4.299042000	-0.021425000	-0.479156000
F	-6.049003000	-1.197073000	1.150593000
F	-5.192219000	-2.833790000	3.141038000
F	-2.533718000	-3.278851000	3.476565000
F	-0.758044000	-2.108383000	1.856512000
F	-1.740218000	2.125226000	1.161508000
F	-2.262226000	4.541121000	0.149147000
F	-2.573032000	4.864016000	-2.531072000
F	-2.354111000	2.719682000	-4.183991000
F	-1.836470000	0.303653000	-3.200294000
C	0.172320000	-3.530198000	-2.092035000
C	1.504417000	-3.071858000	-2.258663000
C	1.792001000	-1.742914000	-1.892568000
C	-0.506324000	-1.347595000	-1.240168000
C	-0.767119000	-2.720631000	-1.487792000
C	3.389370000	-0.426921000	-0.534429000
C	4.697411000	-0.178032000	-0.064761000
C	4.785719000	0.707273000	0.998458000
C	2.433384000	0.881565000	1.134962000
O	1.375751000	1.252357000	1.714962000
H	5.753224000	0.968775000	1.399517000
N	3.710684000	1.256715000	1.594044000
H	-1.736267000	-3.136772000	-1.227962000
H	-0.071853000	-4.552515000	-2.362704000
C	3.148202000	-1.397822000	-1.569611000
C	5.797453000	-0.901400000	-0.638318000
C	4.204947000	-2.229600000	-1.936307000
C	2.599419000	-3.890429000	-2.685831000
C	3.892066000	-3.459206000	-2.598014000
C	5.548492000	-1.899517000	-1.522664000
H	6.808891000	-0.679110000	-0.317152000
H	6.364627000	-2.501567000	-1.907065000
H	4.704106000	-4.099468000	-2.925335000
H	2.388657000	-4.885467000	-3.065211000
H	0.292951000	0.557720000	1.045706000
N	2.346530000	0.109029000	0.076981000
C	0.727006000	-0.868269000	-1.612224000
H	0.918335000	0.188391000	-1.542994000
H	-0.353114000	0.068544000	0.660429000
C	3.854365000	2.224302000	2.759380000
C	3.143232000	3.528064000	2.384759000
C	3.250932000	1.571868000	4.006118000
C	5.326430000	2.531873000	3.027223000
H	3.581761000	3.953839000	1.477941000
H	2.076236000	3.379032000	2.230583000
H	3.278415000	4.244401000	3.198131000
H	3.753362000	0.624662000	4.220406000
H	3.401622000	2.240481000	4.856844000
H	2.184122000	1.391591000	3.889593000
H	5.359050000	3.262756000	3.836920000
H	5.886299000	1.653534000	3.358104000
H	5.822380000	2.978554000	2.161458000

TSA5-6

Coordinates (Angstroms)

	X	Y	Z
B	-1.646241000	-0.098567000	0.076140000
C	-2.870254000	-0.798002000	0.882997000
C	-4.126681000	-0.947751000	0.314944000
C	-2.719254000	-1.352364000	2.145174000
C	-5.174705000	-1.590322000	0.953202000
C	-3.741220000	-1.995588000	2.821084000
C	-4.980730000	-2.114916000	2.218086000
C	-2.084588000	1.270718000	-0.673222000
C	-1.960909000	1.504049000	-2.035245000
C	-2.647503000	2.309515000	0.059333000
C	-2.370842000	2.685033000	-2.636100000
C	-3.063736000	3.502723000	-0.501169000
C	-2.924423000	3.689957000	-1.865233000
F	-4.370633000	-0.482372000	-0.921106000
F	-6.362348000	-1.713085000	0.359273000
F	-5.975842000	-2.735050000	2.847138000
F	-3.542961000	-2.502444000	4.038208000
F	-1.532868000	-1.275752000	2.771951000
F	-2.792369000	2.179578000	1.386614000
F	-3.591699000	4.467713000	0.252100000
F	-3.320152000	4.827692000	-2.428759000
F	-2.238110000	2.857667000	-3.952127000
F	-1.445280000	0.578006000	-2.852230000
C	-0.489092000	-3.098985000	-2.130936000
C	0.919718000	-3.026820000	-2.029818000
C	1.516226000	-1.997136000	-1.256571000
C	-0.732606000	-1.160566000	-0.707657000
C	-1.295390000	-2.197976000	-1.486506000
C	3.698534000	-0.934208000	-0.406948000
C	5.115614000	-1.012422000	-0.334337000
C	5.754111000	-0.048320000	0.423785000
C	3.692075000	0.998045000	0.925442000
O	3.065464000	1.916102000	1.473552000
H	6.828807000	-0.079330000	0.519691000
N	5.105237000	0.928966000	1.058294000
H	-2.372830000	-2.283553000	-1.586520000
H	-0.922531000	-3.894701000	-2.728778000
C	2.965836000	-1.945068000	-1.155172000
C	5.843659000	-2.039295000	-1.008711000
C	3.729167000	-2.927311000	-1.811513000
C	1.737226000	-3.987995000	-2.690113000
C	3.087426000	-3.942238000	-2.581115000
C	5.165206000	-2.955993000	-1.728565000
H	6.924988000	-2.061617000	-0.930108000
H	5.688247000	-3.745695000	-2.256034000
H	3.707819000	-4.679856000	-3.078334000
H	1.256954000	-4.763963000	-3.277553000
N	3.067145000	0.060311000	0.207292000
C	0.640601000	-1.089149000	-0.612655000
H	1.089490000	-0.309327000	-0.024796000
O	0.463247000	1.985710000	0.791777000
H	1.401935000	1.877315000	1.097463000
H	-0.468693000	0.846052000	0.883396000
H	-0.929373000	0.182673000	1.190096000
C	5.846614000	1.954798000	1.898807000
C	5.637098000	3.338186000	1.276049000

C	5.321202000	1.878011000	3.335892000
C	7.345378000	1.652643000	1.919517000
H	6.005814000	3.350769000	0.246497000
H	4.588347000	3.627009000	1.284588000
H	6.207701000	4.070680000	1.851889000
H	5.455544000	0.869181000	3.736203000
H	5.898258000	2.568800000	3.955004000
H	4.269486000	2.148415000	3.396934000
H	7.817371000	2.413095000	2.543905000
H	7.569026000	0.679221000	2.364104000
H	7.802036000	1.716173000	0.928567000
H	0.524258000	2.276971000	-0.124584000

TSA7

	Coordinates (Angstroms)		
	X	Y	Z
B	1.666829000	-0.086376000	0.040706000
C	2.703514000	-1.006739000	-0.801041000
C	4.005652000	-1.254243000	-0.392481000
C	2.307772000	-1.681586000	-1.946288000
C	4.873283000	-2.089392000	-1.076244000
C	3.142703000	-2.521731000	-2.663658000
C	4.438752000	-2.724675000	-2.225173000
C	2.327337000	1.254642000	0.680078000
C	2.096251000	1.718735000	1.970977000
C	3.167819000	2.062860000	-0.082288000
C	2.661496000	2.882327000	2.473000000
C	3.750122000	3.229469000	0.379009000
C	3.495033000	3.642081000	1.674756000
F	4.484323000	-0.684015000	0.728360000
F	6.118253000	-2.290309000	-0.637692000
F	5.258378000	-3.530442000	-2.898811000
F	2.711460000	-3.138172000	-3.766392000
F	1.056990000	-1.537439000	-2.410005000
F	3.446540000	1.724901000	-1.355376000
F	4.545190000	3.956196000	-0.406448000
F	4.039806000	4.761019000	2.143249000
F	2.406674000	3.274900000	3.721775000
F	1.302273000	1.052302000	2.817057000
C	0.471941000	-2.676223000	2.695757000
C	-0.936408000	-2.578997000	2.610652000
C	-1.524987000	-1.690586000	1.672180000
C	0.729229000	-0.989206000	0.980453000
C	1.282378000	-1.912317000	1.899220000
C	-3.707996000	-0.804269000	0.643652000
C	-5.125948000	-0.747098000	0.717074000
C	-5.765241000	0.081964000	-0.184909000
C	-3.711342000	0.646395000	-1.190050000
O	-3.092499000	1.250436000	-2.090378000
H	-6.839704000	0.176053000	-0.147292000
N	-5.114799000	0.787607000	-1.111820000
H	2.360625000	-2.006291000	1.989294000
H	0.897740000	-3.370890000	3.413369000
C	-2.975830000	-1.617338000	1.599277000
C	-5.858821000	-1.510482000	1.675831000
C	-3.744558000	-2.380976000	2.498183000
C	-1.758366000	-3.359977000	3.471289000

C	-3.108983000	-3.254704000	3.427409000
C	-5.181021000	-2.311174000	2.522704000
H	-6.940517000	-1.438701000	1.697848000
H	-5.705435000	-2.917052000	3.253099000
H	-3.732927000	-3.838567000	4.095068000
H	-1.280810000	-4.034195000	4.175127000
H	-1.636889000	1.039623000	-2.155598000
N	-3.076772000	-0.120162000	-0.308852000
C	-0.643623000	-0.908664000	0.884095000
H	-1.079456000	-0.224102000	0.179095000
H	0.841902000	0.392779000	-0.857436000
O	-0.338601000	2.272753000	-0.660806000
C	0.190659000	1.514477000	-1.612483000
H	1.149047000	1.808791000	-2.037178000
O	-0.634054000	0.954065000	-2.418557000
C	-5.854895000	1.706430000	-2.072499000
C	-5.312743000	3.128848000	-1.899761000
C	-5.666204000	1.177462000	-3.497384000
C	-7.350216000	1.721680000	-1.754058000
H	-5.441664000	3.460313000	-0.865578000
H	-4.261303000	3.198887000	-2.168817000
H	-5.885505000	3.798291000	-2.545582000
H	-6.036979000	0.151539000	-3.574351000
H	-6.248057000	1.801865000	-4.179507000
H	-4.622686000	1.204107000	-3.802989000
H	-7.819791000	2.410357000	-2.458325000
H	-7.820044000	0.744699000	-1.892254000
H	-7.559559000	2.090288000	-0.746395000
H	0.360546000	2.711528000	-0.157628000

TSA8

	Coordinates (Angstroms)		
	X	Y	Z
B	1.602374000	0.033134000	-0.201888000
C	2.766689000	-0.965256000	-0.845737000
C	4.032599000	-1.140750000	-0.305602000
C	2.495542000	-1.781074000	-1.934250000
C	4.973028000	-2.023527000	-0.811938000
C	3.400714000	-2.676915000	-2.477787000
C	4.657044000	-2.795900000	-1.913803000
C	2.262975000	1.373978000	0.485886000
C	1.927715000	1.881711000	1.731934000
C	3.172154000	2.146041000	-0.228683000
C	2.465254000	3.052963000	2.248188000
C	3.730014000	3.319359000	0.246170000
C	3.372474000	3.777636000	1.501643000
F	4.410782000	-0.453680000	0.790440000
F	6.176308000	-2.140140000	-0.243729000
F	5.545848000	-3.652446000	-2.417549000
F	3.071196000	-3.430541000	-3.530411000
F	1.275931000	-1.762423000	-2.518142000
F	3.553162000	1.771795000	-1.463101000
F	4.600602000	4.014969000	-0.491523000
F	3.894768000	4.906100000	1.981160000
F	2.107381000	3.489717000	3.459278000
F	1.042872000	1.260336000	2.525794000
C	0.573999000	-2.592168000	2.532385000

C	-0.832735000	-2.642391000	2.395034000
C	-1.477420000	-1.770722000	1.479098000
C	0.723891000	-0.899335000	0.803308000
C	1.327441000	-1.747031000	1.763074000
C	-3.724349000	-0.926559000	0.580365000
C	-5.123806000	-1.127392000	0.464693000
C	-5.818359000	-0.249318000	-0.346160000
C	-3.872459000	0.966058000	-0.755790000
O	-3.318359000	1.991499000	-1.279775000
H	-6.879122000	-0.383367000	-0.489006000
N	-5.236511000	0.777156000	-0.974934000
H	2.405526000	-1.734727000	1.893022000
H	1.045545000	-3.253644000	3.252867000
C	-2.926511000	-1.832636000	1.373690000
C	-5.784627000	-2.182641000	1.163820000
C	-3.629989000	-2.815879000	2.098934000
C	-1.592325000	-3.580484000	3.148926000
C	-2.935548000	-3.682884000	2.989815000
C	-5.052421000	-2.979278000	1.969528000
H	-6.854529000	-2.311724000	1.047492000
H	-5.524057000	-3.774508000	2.535711000
H	-3.506122000	-4.418257000	3.546171000
H	-1.069918000	-4.231730000	3.842546000
O	0.755183000	0.593309000	-1.321257000
N	-3.171928000	0.127339000	-0.032208000
C	-0.650579000	-0.927264000	0.695285000
H	-1.123291000	-0.267848000	-0.010301000
C	-0.198549000	2.338423000	-1.428140000
H	-0.470171000	2.095305000	-2.459230000
H	0.758867000	2.833165000	-1.275936000
O	-1.078286000	2.454266000	-0.552177000
H	-2.233130000	2.145441000	-0.918275000
C	-6.039960000	1.716557000	-1.873143000
C	-6.043326000	3.109599000	-1.238748000
C	-5.416208000	1.706022000	-3.271644000
C	-7.483745000	1.228230000	-1.988349000
H	-6.469979000	3.066852000	-0.233096000
H	-5.045696000	3.540029000	-1.185649000
H	-6.671287000	3.763394000	-1.848173000
H	-5.389933000	0.688316000	-3.670410000
H	-6.042166000	2.313247000	-3.929044000
H	-4.410543000	2.119504000	-3.277190000
H	-8.000357000	1.917490000	-2.657925000
H	-7.553717000	0.229844000	-2.427981000
H	-8.012186000	1.248010000	-1.032277000
H	0.243823000	-0.090755000	-1.760184000

TSA9

	Coordinates (Angstroms)		
	X	Y	Z
B	-1.723462000	-0.032897000	-0.148197000
C	-1.884084000	1.465798000	0.464090000
C	-2.704711000	1.705707000	1.557408000
C	-1.150951000	2.557826000	0.032277000
C	-2.816415000	2.935441000	2.178233000
C	-1.227028000	3.809361000	0.626490000
C	-2.066499000	3.999924000	1.706762000

C	-3.057797000	-0.718851000	-0.776143000
C	-3.020709000	-2.046251000	-1.194006000
C	-4.273452000	-0.083621000	-0.999798000
C	-4.096757000	-2.712593000	-1.753932000
C	-5.377511000	-0.713350000	-1.554166000
C	-5.291102000	-2.039298000	-1.932840000
F	-3.458527000	0.707291000	2.051808000
F	-3.631353000	3.108853000	3.222560000
F	-2.153636000	5.195441000	2.291064000
F	-0.496282000	4.829242000	0.165869000
F	-0.306155000	2.446057000	-1.007539000
F	-4.445984000	1.211006000	-0.688491000
F	-6.521209000	-0.047682000	-1.732348000
F	-6.339850000	-2.657471000	-2.472865000
F	-3.990839000	-3.989384000	-2.130074000
F	-1.884302000	-2.757556000	-1.082927000
C	-0.751427000	-2.471225000	2.786617000
C	0.657212000	-2.357149000	2.790545000
C	1.293030000	-1.553269000	1.808928000
C	-0.914545000	-0.936911000	0.918793000
C	-1.513896000	-1.802219000	1.862047000
C	3.508904000	-0.899713000	0.711470000
C	4.920884000	-0.823353000	0.820743000
C	5.595417000	-0.187553000	-0.205415000
C	3.593815000	0.132053000	-1.368454000
O	3.017540000	0.544170000	-2.424294000
H	6.667533000	-0.078468000	-0.149514000
N	4.978704000	0.309233000	-1.280943000
H	-2.592252000	-1.927302000	1.869348000
H	-1.220027000	-3.103609000	3.534573000
C	2.743627000	-1.508186000	1.775291000
C	5.614223000	-1.394558000	1.930753000
C	3.471299000	-2.133402000	2.805813000
C	1.439762000	-3.017672000	3.780765000
C	2.789454000	-2.881606000	3.809989000
C	4.903932000	-2.048863000	2.873505000
H	6.693577000	-1.309803000	1.983848000
H	5.401370000	-2.522336000	3.712379000
H	3.377594000	-3.361002000	4.584622000
H	0.931685000	-3.617751000	4.528993000
H	1.848388000	0.422824000	-2.498703000
N	2.915951000	-0.439203000	-0.399179000
C	0.460925000	-0.820363000	0.936382000
H	0.924834000	-0.147639000	0.235864000
H	-0.936441000	0.098111000	-1.148895000
C	-0.088802000	-0.486889000	-2.377641000
H	0.305463000	-1.291222000	-1.751271000
O	0.674685000	0.397018000	-2.833969000
H	-1.028927000	-0.665183000	-2.904333000
C	5.757035000	1.026528000	-2.383404000
C	5.626966000	0.220051000	-3.678316000
C	5.209400000	2.450544000	-2.516290000
C	7.237489000	1.112711000	-2.013451000
H	6.003976000	-0.795976000	-3.532649000
H	4.599213000	0.173545000	-4.031088000
H	6.236296000	0.702079000	-4.446108000
H	5.283981000	2.975775000	-1.560298000

H	5.819389000	2.985863000	-3.247250000
H	4.175491000	2.465004000	-2.852920000
H	7.737122000	1.646468000	-2.823201000
H	7.406581000	1.678601000	-1.094036000
H	7.709194000	0.130359000	-1.933215000

TSA3-4_CH_3OH

	Coordinates (Angstroms)		
	X	Y	Z
B	-1.465704000	0.132739000	-0.297364000
C	-2.935277000	0.436616000	-0.997552000
C	-3.903577000	1.086175000	-0.238220000
C	-3.372807000	0.044156000	-2.255319000
C	-5.182163000	1.372528000	-0.682493000
C	-4.644049000	0.311114000	-2.743324000
C	-5.556477000	0.984086000	-1.955063000
C	-1.793322000	-0.824386000	1.005089000
C	-1.523091000	-0.519351000	2.331446000
C	-2.483931000	-2.017717000	0.816900000
C	-1.883883000	-1.340095000	3.391830000
C	-2.862351000	-2.862438000	1.843179000
C	-2.555979000	-2.520370000	3.148174000
F	-3.625311000	1.469075000	1.022943000
F	-6.052825000	2.012045000	0.102352000
F	-6.780617000	1.247789000	-2.410498000
F	-4.996947000	-0.089109000	-3.968141000
F	-2.582003000	-0.651300000	-3.088454000
F	-2.806530000	-2.419401000	-0.422511000
F	-3.512237000	-4.002348000	1.590930000
F	-2.908497000	-3.318536000	4.154996000
F	-1.592646000	-0.991321000	4.648865000
F	-0.901839000	0.617203000	2.680135000
C	-0.339933000	3.886332000	0.380199000
C	1.044178000	3.717416000	0.593368000
C	1.606147000	2.423311000	0.454707000
C	-0.637244000	1.499686000	0.040008000
C	-1.145976000	2.817100000	0.065975000
C	3.751085000	1.094744000	0.098981000
C	5.160722000	1.024031000	0.227955000
C	5.767369000	-0.142284000	-0.203426000
C	3.706215000	-1.004090000	-0.900234000
O	3.043522000	-1.932562000	-1.462154000
H	6.836524000	-0.252569000	-0.108512000
N	5.088404000	-1.160031000	-0.743649000
H	-2.194752000	3.005709000	-0.130779000
H	-0.759688000	4.884373000	0.460385000
C	3.047970000	2.280758000	0.522902000
C	5.909883000	2.124909000	0.746156000
C	3.831349000	3.377540000	0.920933000
C	1.881188000	4.813674000	0.957933000
C	3.212344000	4.641383000	1.159685000
C	5.257867000	3.265681000	1.059848000
H	6.984682000	2.033386000	0.853222000
H	5.802012000	4.131709000	1.419789000
H	3.837845000	5.472882000	1.465209000
H	1.427968000	5.791135000	1.089149000
O	-0.557801000	-0.708052000	-1.243573000

C	0.021358000	-0.060146000	-2.378414000
H	1.030808000	0.288751000	-2.146717000
H	0.054288000	-0.772074000	-3.204556000
H	-0.584837000	0.796241000	-2.671745000
H	1.814780000	-1.836797000	-1.231403000
N	3.096722000	0.080001000	-0.475541000
C	0.717870000	1.348436000	0.257088000
H	1.129028000	0.354657000	0.254788000
C	0.569043000	-2.053087000	-0.787652000
O	0.043586000	-2.877846000	-0.189515000
C	5.790924000	-2.443694000	-1.179766000
C	5.195461000	-3.611086000	-0.387341000
C	5.613839000	-2.606147000	-2.691554000
C	7.284751000	-2.362671000	-0.869016000
H	5.313531000	-3.440264000	0.686117000
H	4.142532000	-3.766196000	-0.611416000
H	5.742069000	-4.519600000	-0.649809000
H	6.016186000	-1.735672000	-3.216707000
H	6.174007000	-3.487486000	-3.011676000
H	4.570262000	-2.740263000	-2.967129000
H	7.727963000	-3.307630000	-1.186926000
H	7.787813000	-1.565347000	-1.421462000
H	7.483205000	-2.250297000	0.199808000

EtOH

	Coordinates (Angstroms)		
	X	Y	Z
C	-1.170582000	-0.406556000	0.000000000
C	0.000000000	0.551064000	0.000000000
H	-1.143048000	-1.046003000	0.886965000
H	-2.116038000	0.142982000	0.000000000
H	-1.143048000	-1.046003000	-0.886965000
H	-0.046846000	1.198413000	-0.886881000
H	-0.046846000	1.198413000	0.886881000
O	1.197858000	-0.214177000	0.000000000
H	1.936458000	0.398571000	0.000000000

TSA3-4_EtOH

	Coordinates (Angstroms)		
	X	Y	Z
B	-1.472339000	0.131752000	-0.271966000
C	-2.975185000	0.465528000	-0.888859000
C	-3.903562000	1.062188000	-0.039503000
C	-3.475567000	0.170148000	-2.148018000
C	-5.205371000	1.367641000	-0.394868000
C	-4.772024000	0.459258000	-2.549736000
C	-5.646332000	1.063985000	-1.669169000
C	-1.754362000	-0.859057000	1.018846000
C	-1.420314000	-0.603820000	2.341025000
C	-2.471425000	-2.035478000	0.822804000
C	-1.747271000	-1.454166000	3.388969000
C	-2.818671000	-2.908162000	1.836691000
C	-2.449462000	-2.615074000	3.137196000
F	-3.557793000	1.377675000	1.223180000
F	-6.034020000	1.948768000	0.476177000
F	-6.894394000	1.347857000	-2.039573000

F	-5.184002000	0.147870000	-3.782025000
F	-2.721464000	-0.443003000	-3.074422000
F	-2.854685000	-2.390725000	-0.413551000
F	-3.498366000	-4.028664000	1.576285000
F	-2.770582000	-3.441242000	4.131986000
F	-1.394976000	-1.151897000	4.642525000
F	-0.766311000	0.510883000	2.699920000
C	-0.391998000	3.897736000	0.392830000
C	0.993173000	3.749703000	0.613630000
C	1.573998000	2.462243000	0.489120000
C	-0.653650000	1.503866000	0.068498000
C	-1.180683000	2.814235000	0.083498000
C	3.738753000	1.159941000	0.170397000
C	5.148382000	1.107588000	0.307440000
C	5.769054000	-0.059994000	-0.099700000
C	3.718129000	-0.961300000	-0.779127000
O	3.061484000	-1.911901000	-1.308051000
H	6.839256000	-0.156628000	-0.001198000
N	5.102253000	-1.096533000	-0.620205000
H	-2.230854000	2.986530000	-0.123326000
H	-0.826210000	4.890297000	0.463087000
C	3.017457000	2.342927000	0.569175000
C	5.880437000	2.227147000	0.810059000
C	3.783733000	3.454834000	0.957242000
C	1.812618000	4.861631000	0.971156000
C	3.145550000	4.712083000	1.179296000
C	5.211414000	3.363637000	1.103397000
H	6.955910000	2.152394000	0.923365000
H	5.742441000	4.242065000	1.452761000
H	3.757096000	5.556314000	1.478124000
H	1.344217000	5.833342000	1.091221000
O	-0.595023000	-0.699750000	-1.259863000
H	1.816597000	-1.798300000	-1.095077000
N	3.098085000	0.128210000	-0.385740000
C	0.701750000	1.372986000	0.293503000
H	1.124547000	0.384031000	0.300557000
C	0.557813000	-2.041129000	-0.753939000
O	-0.001524000	-2.913045000	-0.266207000
C	5.820290000	-2.378423000	-1.034240000
C	5.246317000	-3.538393000	-0.215667000
C	5.635901000	-2.573572000	-2.541317000
C	7.314587000	-2.269375000	-0.735014000
H	5.372217000	-3.347718000	0.853554000
H	4.193136000	-3.708520000	-0.427713000
H	5.800627000	-4.445246000	-0.467600000
H	6.019215000	-1.706715000	-3.086282000
H	6.209299000	-3.451159000	-2.848146000
H	4.592949000	-2.731422000	-2.806704000
H	7.770329000	-3.213596000	-1.037061000
H	7.801946000	-1.475120000	-1.305621000
H	7.517653000	-2.133389000	0.330174000
C	0.002381000	-0.022750000	-2.380194000
C	0.202401000	-0.973918000	-3.543396000
H	0.956479000	0.410670000	-2.065709000
H	-0.654804000	0.799872000	-2.666117000
H	0.543519000	-0.406560000	-4.414048000
H	0.962242000	-1.730216000	-3.329118000

H	-0.728203000	-1.479702000	-3.801356000

PhOH

	Coordinates (Angstroms)		
	X	Y	Z
O	-2.293350000	-0.113298000	0.000028000
H	-2.670894000	0.772258000	0.000125000
C	-0.939915000	-0.026934000	-0.000097000
C	-0.217573000	-1.219905000	-0.000031000
C	-0.267466000	1.194475000	0.000008000
C	1.170085000	-1.183891000	0.000027000
H	-0.755038000	-2.161479000	0.000047000
C	1.122918000	1.216045000	-0.000030000
H	-0.831731000	2.122596000	0.000125000
C	1.850105000	0.031157000	0.000017000
H	1.723954000	-2.116563000	0.000080000
H	1.637808000	2.170675000	0.000022000
H	2.933773000	0.053220000	0.000020000

TSA3-4_PhOH

	Coordinates (Angstroms)		
	X	Y	Z
B	-1.524540000	0.110212000	-0.070282000
C	-2.898418000	-0.062653000	-0.979752000
C	-3.916920000	0.869066000	-0.784717000
C	-3.237984000	-1.079943000	-1.862653000
C	-5.132146000	0.852143000	-1.445645000
C	-4.442915000	-1.135638000	-2.548854000
C	-5.397697000	-0.159979000	-2.347988000
C	-2.041917000	-0.004085000	1.488739000
C	-1.939917000	0.989277000	2.451120000
C	-2.727403000	-1.143101000	1.899096000
C	-2.462331000	0.863109000	3.731085000
C	-3.263235000	-1.308098000	3.161912000
C	-3.127799000	-0.291492000	4.090761000
F	-3.763772000	1.863212000	0.110609000
F	-6.047168000	1.795650000	-1.212845000
F	-6.558847000	-0.201243000	-2.998376000
F	-4.691742000	-2.141016000	-3.391802000
F	-2.418938000	-2.115671000	-2.090753000
F	-2.872684000	-2.179496000	1.056724000
F	-3.896966000	-2.434731000	3.497590000
F	-3.634073000	-0.426372000	5.315054000
F	-2.333520000	1.855516000	4.616071000
F	-1.335205000	2.156915000	2.192186000
C	-0.403087000	3.693044000	-1.345060000
C	0.970535000	3.690515000	-1.027184000
C	1.542739000	2.526334000	-0.453692000
C	-0.682672000	1.464689000	-0.423413000
C	-1.193972000	2.599212000	-1.092826000
C	3.719584000	1.260297000	0.009571000
C	5.136704000	1.297762000	0.088328000
C	5.783402000	0.092051000	0.288739000
C	3.730684000	-1.047552000	0.358187000
O	3.092654000	-2.120053000	0.498848000
H	6.861329000	0.070430000	0.337452000

N	5.136889000	-1.069687000	0.413035000
H	-2.228828000	2.638964000	-1.406583000
H	-0.825031000	4.577181000	-1.812959000
C	2.986341000	2.472529000	-0.290849000
C	5.859471000	2.517329000	-0.085601000
C	3.746898000	3.628148000	-0.544016000
C	1.782342000	4.834614000	-1.279085000
C	3.110670000	4.818081000	-1.008457000
C	5.176904000	3.639901000	-0.393166000
H	6.939297000	2.513880000	0.012378000
H	5.696598000	4.577126000	-0.557192000
H	3.723007000	5.695346000	-1.185636000
H	1.313889000	5.724449000	-1.687259000
O	-0.528286000	-1.119491000	-0.238799000
H	1.694023000	-1.840388000	0.652910000
N	3.089495000	0.094999000	0.162424000
C	0.661812000	1.479794000	-0.105937000
H	1.088561000	0.629447000	0.391510000
C	0.591353000	-1.711720000	1.147438000
O	0.054037000	-1.864391000	2.137552000
C	5.885121000	-2.383988000	0.595075000
C	5.490601000	-2.985315000	1.947056000
C	5.538417000	-3.300949000	-0.582049000
C	7.394859000	-2.145327000	0.594261000
H	5.727419000	-2.287609000	2.755055000
H	4.432629000	-3.233773000	1.988633000
H	6.069263000	-3.898472000	2.104065000
H	5.815417000	-2.825152000	-1.526787000
H	6.111521000	-4.225613000	-0.483278000
H	4.479707000	-3.549418000	-0.606350000
H	7.871891000	-3.119129000	0.715576000
H	7.752732000	-1.721361000	-0.347174000
H	7.720926000	-1.516737000	1.427156000
C	0.063981000	-1.527918000	-1.416192000
C	0.466690000	-2.860323000	-1.480275000
C	0.271604000	-0.687658000	-2.506409000
C	1.109727000	-3.341982000	-2.612131000
H	0.244827000	-3.527286000	-0.653751000
C	0.901783000	-1.185899000	-3.640271000
H	-0.059971000	0.339243000	-2.485239000
C	1.335089000	-2.505264000	-3.698567000
H	1.418711000	-4.380693000	-2.647034000
H	1.055588000	-0.524946000	-4.486177000
H	1.830134000	-2.881202000	-4.586529000

^{i}PrOH

	Coordinates (Angstroms)		
	X	Y	Z
O	-0.077056000	1.364786000	-0.167557000
H	-0.911854000	1.743495000	0.119006000
C	0.002190000	0.042971000	0.362465000
C	1.334058000	-0.526301000	-0.088081000
C	-1.176807000	-0.801191000	-0.102444000
H	-0.008102000	0.090388000	1.461256000
H	2.155442000	0.110371000	0.250534000
H	1.482593000	-1.529296000	0.320571000
H	1.370711000	-0.586470000	-1.180414000

H	-2.123625000	-0.358908000	0.223926000
H	-1.186521000	-0.867502000	-1.194999000
H	-1.118843000	-1.813234000	0.308935000

TSA3-4_^{i}PrOH

	Coordinates (Angstroms)		
	X	Y	Z
B	-1.547909000	0.278431000	-0.272230000
C	-3.184032000	0.448162000	-0.447301000
C	-3.916571000	0.848394000	0.665057000
C	-3.958111000	0.154843000	-1.560352000
C	-5.292073000	1.002160000	0.677867000
C	-5.337400000	0.294472000	-1.594977000
C	-6.011849000	0.726701000	-0.469558000
C	-1.373902000	-0.996386000	0.783685000
C	-0.746039000	-0.979837000	2.025605000
C	-1.958051000	-2.220896000	0.461939000
C	-0.653606000	-2.091383000	2.851552000
C	-1.888612000	-3.352564000	1.256265000
C	-1.223264000	-3.288244000	2.465258000
F	-3.287788000	1.093397000	1.830202000
F	-5.926768000	1.404109000	1.782218000
F	-7.337244000	0.867315000	-0.486903000
F	-6.022022000	0.005008000	-2.705495000
F	-3.401963000	-0.303936000	-2.693370000
F	-2.640877000	-2.364996000	-0.684640000
F	-2.460732000	-4.495667000	0.871033000
F	-1.139794000	-4.361458000	3.249093000
F	-0.023912000	-2.009962000	4.027013000
F	-0.193709000	0.133401000	2.526280000
C	-0.553548000	4.001334000	0.767502000
C	0.854027000	3.876592000	0.792476000
C	1.436806000	2.618224000	0.494262000
C	-0.811392000	1.657864000	0.199394000
C	-1.363390000	2.926958000	0.471321000
C	3.575343000	1.279823000	0.116711000
C	4.988193000	1.211078000	0.198547000
C	5.567887000	0.000769000	-0.140985000
C	3.470817000	-0.916686000	-0.653384000
O	2.769050000	-1.886352000	-1.068703000
H	6.639261000	-0.112717000	-0.078453000
N	4.861579000	-1.062780000	-0.546001000
H	-2.438011000	3.078754000	0.440945000
H	-0.990148000	4.971467000	0.985212000
C	2.882257000	2.490756000	0.489685000
C	5.753505000	2.346448000	0.609332000
C	3.673267000	3.593409000	0.850507000
C	1.698631000	4.977691000	1.122529000
C	3.049329000	4.838379000	1.165657000
C	5.108107000	3.495686000	0.908048000
H	6.833197000	2.268497000	0.666626000
H	5.664546000	4.376851000	1.207994000
H	3.681177000	5.677834000	1.434659000
H	1.239926000	5.934701000	1.350993000
O	-0.794469000	-0.200357000	-1.533735000
H	1.463234000	-1.646742000	-1.192294000
N	2.900496000	0.219415000	-0.324984000

C	0.560203000	1.553070000	0.227049000
H	0.988577000	0.593740000	0.019484000
C	0.256130000	-1.799174000	-1.611807000
O	-0.329496000	-2.594375000	-2.181828000
C	5.539192000	-2.384717000	-0.889292000
C	4.982076000	-3.466901000	0.039836000
C	5.280810000	-2.690975000	-2.366943000
C	7.047396000	-2.284097000	-0.666783000
H	5.156086000	-3.198564000	1.085376000
H	3.917778000	-3.628934000	-0.115860000
H	5.508541000	-4.402050000	-0.163896000
H	5.651146000	-1.876273000	-2.995231000
H	5.824116000	-3.600677000	-2.632370000
H	4.223522000	-2.847208000	-2.569751000
H	7.472499000	-3.258836000	-0.911245000
H	7.520484000	-1.548378000	-1.322039000
H	7.302373000	-2.067974000	0.373859000
C	-0.648009000	0.500768000	-2.793449000
C	0.826761000	0.574538000	-3.175407000
C	-1.251385000	1.899786000	-2.825043000
H	-1.176094000	-0.111083000	-3.530070000
H	1.275570000	-0.410917000	-3.328595000
H	0.925248000	1.116969000	-4.119622000
H	1.395995000	1.100287000	-2.405741000
H	-2.258623000	1.941369000	-2.413295000
H	-0.629793000	2.619040000	-2.289857000
H	-1.310001000	2.209743000	-3.872335000

^{t}BuOH

	Coordinates (Angstroms)		
	X	Y	Z
O	-0.061892000	0.000142000	1.447743000
H	-0.991785000	-0.002126000	1.691484000
C	0.006553000	0.000039000	0.017013000
C	-0.666913000	-1.259864000	-0.526838000
C	-0.674231000	1.255777000	-0.527355000
C	1.493030000	0.004190000	-0.315779000
H	-0.196015000	-2.151855000	-0.104654000
H	-1.729307000	-1.273378000	-0.260127000
H	-0.592051000	-1.309643000	-1.617141000
H	-0.208481000	2.150659000	-0.105582000
H	-0.599694000	1.305512000	-1.617681000
H	-1.736674000	1.263217000	-0.260604000
H	1.647064000	0.004263000	-1.398004000
H	1.973230000	0.892522000	0.103770000
H	1.978215000	-0.881157000	0.104350000

TSA3-4_^{t}BuOH

	Coordinates (Angstroms)		
	X	Y	Z
B	-1.503341000	0.245763000	-0.328419000
C	-3.146619000	0.460576000	-0.454360000
C	-3.802182000	1.031733000	0.634715000
C	-4.005869000	0.026422000	-1.453805000
C	-5.169172000	1.235209000	0.707489000
C	-5.380600000	0.210567000	-1.426556000

C	-5.970570000	0.827160000	-0.341704000
C	-1.389958000	-0.932302000	0.852806000
C	-0.805717000	-0.813374000	2.111074000
C	-2.045635000	-2.148409000	0.655906000
C	-0.818304000	-1.823296000	3.064261000
C	-2.080624000	-3.179784000	1.577026000
C	-1.455467000	-3.017488000	2.798587000
F	-3.106034000	1.398404000	1.726985000
F	-5.718900000	1.806553000	1.782611000
F	-7.289965000	1.010812000	-0.298986000
F	-6.141089000	-0.219034000	-2.438070000
F	-3.551774000	-0.635019000	-2.530554000
F	-2.698542000	-2.386377000	-0.490788000
F	-2.716469000	-4.321473000	1.301638000
F	-1.475255000	-3.992548000	3.705018000
F	-0.224394000	-1.640558000	4.247350000
F	-0.196194000	0.310061000	2.512645000
C	-0.461915000	4.020429000	0.425810000
C	0.933072000	3.872811000	0.593408000
C	1.511144000	2.588092000	0.433712000
C	-0.726171000	1.636683000	0.039494000
C	-1.262754000	2.940068000	0.135695000
C	3.657797000	1.236225000	0.198778000
C	5.065782000	1.165032000	0.341531000
C	5.657598000	-0.044573000	0.022557000
C	3.584144000	-0.950273000	-0.595937000
O	2.899834000	-1.903322000	-1.072145000
H	6.724880000	-0.160995000	0.132604000
N	4.967653000	-1.102368000	-0.424020000
H	-2.324497000	3.108207000	-0.016258000
H	-0.894356000	5.010830000	0.530834000
C	2.952601000	2.453576000	0.521185000
C	5.814824000	2.301832000	0.777929000
C	3.732527000	3.564559000	0.880743000
C	1.765731000	4.980955000	0.929587000
C	3.105447000	4.828562000	1.094502000
C	5.161197000	3.459145000	1.021934000
H	6.889772000	2.219687000	0.891585000
H	5.705903000	4.344073000	1.331990000
H	3.727166000	5.673525000	1.369811000
H	1.304940000	5.954458000	1.064854000
O	-0.808661000	-0.377788000	-1.573986000
H	1.569212000	-1.662914000	-1.142196000
N	2.998496000	0.179546000	-0.270543000
C	0.634102000	1.511266000	0.199495000
H	1.057394000	0.528598000	0.125544000
C	0.340839000	-1.917594000	-1.356396000
O	-0.259077000	-2.863990000	-1.560784000
C	5.656720000	-2.422416000	-0.749582000
C	5.045849000	-3.514784000	0.132491000
C	5.477571000	-2.706976000	-2.243286000
C	7.151236000	-2.332920000	-0.444312000
H	5.172434000	-3.265532000	1.189541000
H	3.988749000	-3.664639000	-0.076175000
H	5.572980000	-4.451525000	-0.061818000
H	5.880008000	-1.882705000	-2.838391000
H	6.035030000	-3.612663000	-2.492769000

H	4.432773000	-2.860404000	-2.504072000
H	7.583801000	-3.308040000	-0.673602000
H	7.663424000	-1.594752000	-1.066666000
H	7.351374000	-2.126536000	0.610262000
C	-0.538033000	0.268318000	-2.863841000
C	-1.443049000	1.473857000	-3.115231000
C	0.920197000	0.730291000	-2.935049000
C	-0.778164000	-0.785822000	-3.948455000
H	-2.487687000	1.191656000	-3.223586000
H	-1.349372000	2.224191000	-2.331522000
H	-1.127754000	1.936692000	-4.054285000
H	1.616210000	-0.083038000	-2.721659000
H	1.128202000	1.082182000	-3.949568000
H	1.119598000	1.544233000	-2.239855000
H	-0.740704000	-0.310839000	-4.932404000
H	-0.008646000	-1.562439000	-3.933970000
H	-1.751868000	-1.257095000	-3.822749000

PhOH-NH_2

	Coordinates (Angstroms)		
	X	Y	Z
O	-2.780095000	-0.092793000	0.009426000
H	-3.143877000	0.797642000	0.008699000
C	-1.417838000	-0.019603000	0.002247000
C	-0.700382000	-1.212583000	-0.000184000
C	-0.724133000	1.186387000	-0.000191000
C	0.687040000	-1.197432000	-0.006887000
H	-1.239287000	-2.153678000	0.004123000
C	0.666479000	1.198576000	-0.006751000
H	-1.269537000	2.125984000	0.004134000
C	1.397811000	0.008221000	-0.009897000
H	1.231032000	-2.137020000	-0.010796000
H	1.191609000	2.148738000	-0.010984000
N	2.796329000	0.019023000	-0.080102000
H	3.215098000	0.852949000	0.308176000
H	3.227566000	-0.806828000	0.311927000

TSA3-4_PhOH-NH_2

	Coordinates (Angstroms)		
	X	Y	Z
B	1.572115000	-0.088726000	-0.102984000
C	2.907763000	0.704304000	-0.683247000
C	3.954309000	-0.075963000	-1.172588000
C	3.189806000	2.064575000	-0.669031000
C	5.143536000	0.427364000	-1.668705000
C	4.366596000	2.613415000	-1.159326000
C	5.351417000	1.793084000	-1.670296000
C	2.150480000	-1.037166000	1.115232000
C	2.097724000	-2.422114000	1.162329000
C	2.830161000	-0.445659000	2.175202000
C	2.661632000	-3.169988000	2.186870000
C	3.406222000	-1.152574000	3.213663000
C	3.318728000	-2.533349000	3.220283000
F	3.858039000	-1.419475000	-1.169297000
F	6.088531000	-0.391955000	-2.135635000
F	6.486357000	2.305856000	-2.142157000

F	4.559702000	3.934647000	-1.122386000
F	2.339707000	2.953943000	-0.138436000
F	2.932320000	0.892255000	2.245572000
F	4.034286000	-0.521683000	4.209580000
F	3.863656000	-3.238277000	4.210532000
F	2.579435000	-4.503577000	2.177441000
F	1.502177000	-3.134904000	0.195801000
C	0.486154000	-1.957564000	-3.433326000
C	-0.876379000	-2.217039000	-3.179439000
C	-1.452785000	-1.744503000	-1.972961000
C	0.751213000	-0.891491000	-1.267920000
C	1.262941000	-1.277126000	-2.527603000
C	-3.636136000	-1.183954000	-0.760511000
C	-5.048730000	-1.313500000	-0.708818000
C	-5.710781000	-0.559054000	0.242492000
C	-3.676447000	0.299437000	1.039714000
O	-3.049835000	1.006879000	1.869472000
H	-6.787390000	-0.605815000	0.303123000
N	-5.080352000	0.251913000	1.096323000
H	2.288258000	-1.062867000	-2.798793000
H	0.911288000	-2.295210000	-4.373576000
C	-2.890951000	-1.864143000	-1.798894000
C	-5.754155000	-2.136857000	-1.638635000
C	-3.637113000	-2.590923000	-2.743605000
C	-1.673223000	-2.940778000	-4.113772000
C	-2.992300000	-3.155445000	-3.884709000
C	-5.060876000	-2.750088000	-2.620764000
H	-6.829942000	-2.237922000	-1.550252000
H	-5.569251000	-3.361148000	-3.358170000
H	-3.592200000	-3.722051000	-4.588216000
H	-1.200467000	-3.325505000	-5.011870000
O	0.552007000	0.879449000	0.615528000
H	-1.672923000	0.734989000	1.788199000
N	-3.022690000	-0.396974000	0.124012000
C	-0.582303000	-1.159909000	-1.028837000
H	-1.010104000	-0.868040000	-0.088780000
C	-0.540306000	0.328393000	2.049157000
O	0.024933000	-0.215627000	2.872941000
C	-5.848075000	1.120243000	2.083862000
C	-5.464098000	0.706001000	3.506951000
C	-5.513705000	2.586505000	1.790236000
C	-7.353254000	0.921924000	1.908052000
H	-5.680196000	-0.353915000	3.666736000
H	-4.411879000	0.890544000	3.711149000
H	-6.065017000	1.287390000	4.209979000
H	-5.754431000	2.830357000	0.751879000
H	-6.122785000	3.218138000	2.440777000
H	-4.464251000	2.808135000	1.971907000
H	-7.848266000	1.577992000	2.625803000
H	-7.699113000	1.206318000	0.910817000
H	-7.670538000	-0.100581000	2.128039000
C	-0.111722000	1.943608000	0.027822000
C	-0.538549000	2.960357000	0.876098000
C	-0.375479000	2.046029000	-1.333570000
C	-1.258307000	4.039949000	0.388469000
H	-0.285012000	2.917902000	1.930466000
C	-1.078905000	3.137469000	-1.823798000

H	-0.028303000	1.292599000	-2.024511000
C	-1.548982000	4.145033000	-0.975007000
H	-1.588067000	4.816707000	1.070882000
H	-1.271842000	3.206427000	-2.889756000
N	-2.324747000	5.194208000	-1.465361000
H	-2.289998000	6.033170000	-0.903478000
H	-2.169076000	5.403899000	-2.441345000

PhOH-OCH$_3$

	Coordinates (Angstroms)		
	X	Y	Z
O	3.178171000	0.392648000	0.000145000
H	3.662193000	-0.438796000	0.000064000
C	1.841235000	0.134039000	0.000043000
C	0.966891000	1.212726000	-0.000111000
C	1.324084000	-1.162022000	0.000091000
C	-0.410938000	1.010157000	-0.000219000
H	1.369597000	2.219398000	-0.000140000
C	-0.046074000	-1.366543000	-0.000045000
H	1.997402000	-2.014141000	0.000259000
C	-0.927491000	-0.284383000	-0.000195000
H	-1.064411000	1.873140000	-0.000278000
H	-0.453262000	-2.371296000	0.000055000
O	-2.251533000	-0.591999000	-0.000292000
C	-3.181169000	0.475106000	0.000368000
H	-3.077615000	1.099312000	0.895079000
H	-4.167661000	0.013034000	0.000737000
H	-3.078567000	1.099679000	-0.894194000

TSA3-4_PhOH-OCH$_3$

	Coordinates (Angstroms)		
	X	Y	Z
B	1.639494000	-0.141186000	-0.104338000
C	2.924531000	0.731646000	-0.682992000
C	4.006690000	0.013415000	-1.189849000
C	3.134015000	2.104520000	-0.657683000
C	5.162251000	0.584204000	-1.692191000
C	4.275215000	2.720131000	-1.152506000
C	5.297529000	1.959044000	-1.681067000
C	2.277753000	-1.073175000	1.095125000
C	2.298085000	-2.459275000	1.126008000
C	2.927028000	-0.457977000	2.160615000
C	2.902821000	-3.187741000	2.141039000
C	3.541203000	-1.145228000	3.190288000
C	3.527367000	-2.528717000	3.180755000
F	3.981722000	-1.333228000	-1.200578000
F	6.144148000	-0.179091000	-2.177315000
F	6.398400000	2.536576000	-2.158345000
F	4.397580000	4.049208000	-1.103017000
F	2.241579000	2.941860000	-0.112269000
F	2.958321000	0.882707000	2.245880000
F	4.136260000	-0.493571000	4.192886000
F	4.110753000	-3.214910000	4.162016000
F	2.890580000	-4.523523000	2.117199000
F	1.735839000	-3.189760000	0.153015000
C	0.627767000	-1.999521000	-3.462202000

C	-0.718390000	-2.332909000	-3.207264000
C	-1.311709000	-1.914233000	-1.989264000
C	0.849975000	-0.962622000	-1.277625000
C	1.373328000	-1.296621000	-2.547653000
C	-3.515704000	-1.478847000	-0.762495000
C	-4.922629000	-1.663598000	-0.719800000
C	-5.618354000	-0.945256000	0.235349000
C	-3.621691000	-0.025988000	1.060715000
O	-3.027261000	0.690691000	1.904955000
H	-6.693001000	-1.032061000	0.286603000
N	-5.024237000	-0.120601000	1.101932000
H	2.384566000	-1.024719000	-2.820049000
H	1.064121000	-2.295538000	-4.411232000
C	-2.741875000	-2.105902000	-1.813455000
C	-5.591744000	-2.500185000	-1.664524000
C	-3.456213000	-2.846418000	-2.772260000
C	-1.482480000	-3.076161000	-4.153521000
C	-2.788869000	-3.358044000	-3.925247000
C	-4.871843000	-3.069013000	-2.653976000
H	-6.662884000	-2.645170000	-1.580980000
H	-5.353058000	-3.688658000	-3.402328000
H	-3.363967000	-3.937495000	-4.638932000
H	-0.994961000	-3.419119000	-5.060583000
O	0.572349000	0.755941000	0.648205000
H	-1.627233000	0.474207000	1.818799000
N	-2.936524000	-0.687507000	0.141135000
C	-0.466269000	-1.305505000	-1.037163000
H	-0.903392000	-1.058382000	-0.088535000
C	-0.486915000	0.102374000	2.063351000
O	0.102069000	-0.455176000	2.860307000
C	-5.830442000	0.717364000	2.084882000
C	-5.451977000	0.307721000	3.510644000
C	-5.537001000	2.194772000	1.802183000
C	-7.326501000	0.474903000	1.888557000
H	-5.647091000	-0.756989000	3.665345000
H	-4.406003000	0.513966000	3.725865000
H	-6.072380000	0.874071000	4.209062000
H	-5.774441000	2.436144000	0.762403000
H	-6.170841000	2.805353000	2.449140000
H	-4.496585000	2.446517000	1.996295000
H	-7.850949000	1.111047000	2.603400000
H	-7.667227000	0.754981000	0.888436000
H	-7.615808000	-0.558077000	2.098113000
C	-0.142908000	1.807799000	0.106748000
C	-0.596697000	2.775347000	0.991648000
C	-0.438810000	1.932632000	-1.249777000
C	-1.379646000	3.839361000	0.555331000
H	-0.318745000	2.712881000	2.038608000
C	-1.201476000	2.999391000	-1.690769000
H	-0.073263000	1.212578000	-1.966423000
C	-1.694432000	3.951696000	-0.795958000
H	-1.721097000	4.569570000	1.277088000
H	-1.431305000	3.107071000	-2.744576000
O	-2.451210000	4.940101000	-1.332466000
C	-2.972159000	5.924961000	-0.457760000
H	-3.631309000	5.482098000	0.297530000
H	-3.550698000	6.604719000	-1.081917000

H	-2.171768000	6.484747000	0.038414000

PhOH-F

	Coordinates (Angstroms)		
	X	Y	Z
O	-2.734607000	0.094012000	0.000215000
H	-3.105573000	-0.794161000	0.000928000
C	-1.379630000	0.019686000	-0.000418000
C	-0.668815000	1.219251000	-0.000114000
C	-0.693317000	-1.193481000	-0.000150000
C	0.718854000	1.207490000	0.000037000
H	-1.211890000	2.156923000	0.000039000
C	0.696980000	-1.209354000	-0.000010000
H	-1.243232000	-2.129355000	0.000042000
C	1.378589000	-0.008220000	-0.000020000
H	1.285288000	2.131094000	0.000165000
H	1.244781000	-2.144037000	0.000130000
F	2.732391000	-0.020533000	0.000114000

TSA3-4_PhOH-F

	Coordinates (Angstroms)		
	X	Y	Z
B	1.570123000	-0.103364000	-0.104689000
C	2.909685000	0.670365000	-0.695975000
C	3.951578000	-0.127910000	-1.166042000
C	3.204211000	2.027970000	-0.702602000
C	5.146807000	0.356548000	-1.666151000
C	4.387659000	2.558167000	-1.196909000
C	5.366525000	1.720249000	-1.690597000
C	2.135755000	-1.016405000	1.142342000
C	2.091790000	-2.400532000	1.217852000
C	2.800602000	-0.396769000	2.195668000
C	2.650555000	-3.121636000	2.264044000
C	3.369840000	-1.076807000	3.255270000
C	3.291946000	-2.457829000	3.290382000
F	3.843983000	-1.469729000	-1.135154000
F	6.086479000	-0.478951000	-2.113501000
F	6.507402000	2.214603000	-2.166461000
F	4.592401000	3.877490000	-1.180883000
F	2.359281000	2.933193000	-0.189354000
F	2.892651000	0.943101000	2.236453000
F	3.982483000	-0.420169000	4.243612000
F	3.831313000	-3.137045000	4.300880000
F	2.578690000	-4.455378000	2.282294000
F	1.511984000	-3.136479000	0.259680000
C	0.470808000	-2.051475000	-3.380109000
C	-0.893389000	-2.296436000	-3.120764000
C	-1.469522000	-1.784161000	-1.930796000
C	0.739065000	-0.926309000	-1.245623000
C	1.250137000	-1.349503000	-2.493620000
C	-3.652878000	-1.172870000	-0.740965000
C	-5.065538000	-1.300529000	-0.685877000
C	-5.728045000	-0.521337000	0.244794000
C	-3.694651000	0.374231000	1.007415000
O	-3.070513000	1.117894000	1.803895000
H	-6.804255000	-0.571621000	0.309771000

N	-5.099038000	0.315557000	1.073590000
H	2.276968000	-1.148510000	-2.768874000
H	0.895518000	-2.419104000	-4.309167000
C	-2.908362000	-1.890838000	-1.754825000
C	-5.772065000	-2.152385000	-1.588582000
C	-3.655577000	-2.646236000	-2.676099000
C	-1.691712000	-3.046539000	-4.032808000
C	-3.011345000	-3.249730000	-3.797597000
C	-5.079650000	-2.798205000	-2.550103000
H	-6.847946000	-2.249341000	-1.496782000
H	-5.588507000	-3.432301000	-3.267376000
H	-3.612687000	-3.837447000	-4.482271000
H	-1.219504000	-3.461166000	-4.917719000
O	0.545397000	0.900650000	0.585932000
H	-1.662643000	0.816856000	1.737827000
N	-3.039684000	-0.353261000	0.114178000
C	-0.596872000	-1.178599000	-1.001787000
H	-1.027287000	-0.859348000	-0.071939000
C	-0.562363000	0.384505000	2.010173000
O	-0.022598000	-0.183890000	2.833279000
C	-5.867511000	1.195606000	2.049929000
C	-5.466113000	0.812116000	3.476941000
C	-5.553490000	2.660072000	1.727072000
C	-7.371941000	0.977522000	1.892349000
H	-5.674282000	-0.245734000	3.659417000
H	-4.412991000	1.006895000	3.666787000
H	-6.063286000	1.403671000	4.174665000
H	-5.805390000	2.881856000	0.686408000
H	-6.165966000	3.295784000	2.370375000
H	-4.505750000	2.897677000	1.897474000
H	-7.867563000	1.640893000	2.602860000
H	-7.730209000	1.240292000	0.893603000
H	-7.675804000	-0.044291000	2.133385000
C	-0.086883000	1.959787000	-0.031008000
C	-0.518456000	2.993905000	0.796408000
C	-0.309798000	2.032193000	-1.403193000
C	-1.207398000	4.077747000	0.271954000
H	-0.292906000	2.963533000	1.856664000
C	-0.981650000	3.124338000	-1.936189000
H	0.042379000	1.256265000	-2.065413000
C	-1.430604000	4.119823000	-1.090051000
H	-1.551626000	4.884087000	0.907774000
H	-1.158674000	3.199941000	-3.002173000
F	-2.096927000	5.172921000	-1.612789000

PhOH-NO_2

	Coordinates (Angstroms)		
	X	Y	Z
O	-3.412329000	0.085652000	0.000012000
H	-3.788644000	-0.801907000	-0.000564000
C	-2.070392000	0.016938000	-0.000001000
C	-1.366973000	1.225527000	0.000321000
C	-1.381328000	-1.199482000	-0.000269000
C	0.013209000	1.218311000	0.000307000
H	-1.917234000	2.158315000	0.000561000
C	0.001200000	-1.207748000	-0.000241000
H	-1.930490000	-2.135078000	-0.000486000

C	0.685673000	0.000640000	0.000021000
H	0.571055000	2.144875000	0.000511000
H	0.548423000	-2.140635000	-0.000423000
N	2.144570000	-0.007928000	-0.000019000
O	2.713572000	-1.085453000	0.000732000
O	2.725829000	1.062901000	-0.000782000

TSA3-4_PhOH-NO_2

	Coordinates (Angstroms)		
	X	Y	Z
B	-1.687112000	-0.160101000	0.119770000
C	-2.960347000	0.833462000	0.464779000
C	-4.038091000	0.273064000	1.148195000
C	-3.169340000	2.150103000	0.075456000
C	-5.192207000	0.957445000	1.484042000
C	-4.309458000	2.874832000	0.390908000
C	-5.328332000	2.279820000	1.107087000
C	-2.314817000	-1.349964000	-0.822006000
C	-2.311537000	-2.703405000	-0.517076000
C	-3.009484000	-1.016126000	-1.980853000
C	-2.939174000	-3.659773000	-1.303701000
C	-3.647577000	-1.935909000	-2.790103000
C	-3.611731000	-3.275926000	-2.446262000
F	-4.010134000	-1.023585000	1.508447000
F	-6.172388000	0.351047000	2.155747000
F	-6.428529000	2.962273000	1.415352000
F	-4.434549000	4.141592000	-0.010204000
F	-2.276095000	2.808429000	-0.678371000
F	-3.055783000	0.262913000	-2.388426000
F	-4.284391000	-1.550675000	-3.898141000
F	-4.217322000	-4.182402000	-3.209770000
F	-2.903733000	-4.949130000	-0.958638000
F	-1.705198000	-3.173619000	0.580679000
C	-0.624561000	-1.117374000	3.808481000
C	0.716801000	-1.512165000	3.628149000
C	1.295697000	-1.418422000	2.336813000
C	-0.872274000	-0.666479000	1.436945000
C	-1.380625000	-0.665493000	2.755438000
C	3.484223000	-1.338799000	1.008391000
C	4.883775000	-1.576429000	0.989264000
C	5.571488000	-1.169441000	-0.139126000
C	3.581210000	-0.404104000	-1.131383000
O	2.989651000	0.103344000	-2.111437000
H	6.639826000	-1.312957000	-0.190731000
N	4.978418000	-0.584259000	-1.181594000
H	-2.387527000	-0.327067000	2.960645000
H	-1.049157000	-1.163392000	4.806477000
C	2.721003000	-1.665820000	2.195929000
C	5.556825000	-2.159107000	2.105755000
C	3.441560000	-2.154531000	3.300339000
C	1.489563000	-1.999164000	4.722622000
C	2.788735000	-2.346877000	4.554891000
C	4.850017000	-2.431939000	3.222196000
H	6.622251000	-2.349894000	2.043063000
H	5.334735000	-2.849732000	4.097295000
H	3.369724000	-2.735522000	5.383870000
H	1.013413000	-2.096261000	5.692908000

O	-0.607742000	0.577805000	-0.833333000
H	1.508047000	-0.032466000	-1.922362000
N	2.903706000	-0.774262000	-0.051044000
C	0.439465000	-1.065522000	1.271164000
H	0.865408000	-1.075406000	0.285134000
C	0.380105000	-0.367269000	-2.085252000
O	-0.245721000	-1.027940000	-2.765745000
C	5.777937000	-0.115827000	-2.389771000
C	5.313258000	-0.905511000	-3.616461000
C	5.572398000	1.393642000	-2.551112000
C	7.269098000	-0.379161000	-2.178566000
H	5.454701000	-1.977432000	-3.452951000
H	4.268068000	-0.713605000	-3.848392000
H	5.921906000	-0.607452000	-4.473357000
H	5.882214000	1.918333000	-1.643119000
H	6.197800000	1.741663000	-3.376246000
H	4.536340000	1.641594000	-2.770056000
H	7.787167000	-0.010564000	-3.065290000
H	7.670711000	0.158985000	-1.316125000
H	7.498100000	-1.443960000	-2.086181000
C	0.106451000	1.699883000	-0.506979000
C	0.678762000	2.410860000	-1.564198000
C	0.278896000	2.143848000	0.804920000
C	1.472961000	3.514717000	-1.317317000
H	0.482045000	2.117490000	-2.588661000
C	1.056179000	3.259726000	1.053861000
H	-0.190425000	1.634416000	1.631605000
C	1.661565000	3.920319000	-0.005403000
H	1.929300000	4.060861000	-2.131308000
H	1.198680000	3.611413000	2.066447000
N	2.505384000	5.084143000	0.266917000
O	2.613550000	5.457015000	1.420581000
O	3.061795000	5.620205000	-0.674050000

PhOH-CH_3

	Coordinates (Angstroms)		
	X	Y	Z
O	2.794887000	0.096098000	0.002444000
H	3.163992000	-0.792769000	0.002525000
C	1.438680000	0.019679000	0.001195000
C	0.722498000	1.216272000	-0.000247000
C	0.751545000	-1.190158000	-0.000306000
C	-0.663594000	1.190448000	-0.003075000
H	1.263665000	2.155914000	-0.000843000
C	-0.640641000	-1.196377000	-0.003036000
H	1.301728000	-2.126804000	-0.000970000
C	-1.374806000	-0.013155000	-0.004016000
H	-1.207331000	2.130652000	-0.005677000
H	-1.161289000	-2.149124000	-0.005489000
C	-2.881626000	-0.017224000	0.004061000
H	-3.283097000	0.610850000	-0.796607000
H	-3.276149000	-1.026948000	-0.129357000
H	-3.272954000	0.372536000	0.949408000

TSA3-4_PhOH-CH_3

Coordinates (Angstroms)

	X	Y	Z
B	1.577923000	-0.090104000	-0.106966000
C	2.916071000	0.706431000	-0.672569000
C	3.959786000	-0.071533000	-1.171450000
C	3.203870000	2.064993000	-0.636659000
C	5.151945000	0.434270000	-1.657783000
C	4.383770000	2.616269000	-1.116374000
C	5.365642000	1.799043000	-1.638123000
C	2.147652000	-1.051363000	1.103638000
C	2.091326000	-2.436585000	1.135980000
C	2.824140000	-0.471880000	2.172300000
C	2.649621000	-3.196091000	2.155013000
C	3.394509000	-1.190599000	3.205627000
C	3.304061000	-2.571283000	3.197381000
F	3.856778000	-1.414299000	-1.187934000
F	6.094215000	-0.381729000	-2.135371000
F	6.503598000	2.313686000	-2.100093000
F	4.582683000	3.935554000	-1.058595000
F	2.356014000	2.948851000	-0.092989000
F	2.927370000	0.865073000	2.256829000
F	4.019639000	-0.571497000	4.210459000
F	3.843288000	-3.287474000	4.182425000
F	2.564304000	-4.529105000	2.131871000
F	1.496915000	-3.137200000	0.160263000
C	0.487926000	-1.910309000	-3.459392000
C	-0.873954000	-2.175169000	-3.207557000
C	-1.450903000	-1.717463000	-1.995705000
C	0.752810000	-0.872323000	-1.280791000
C	1.264616000	-1.241043000	-2.545593000
C	-3.635194000	-1.178381000	-0.774512000
C	-5.046794000	-1.319265000	-0.720747000
C	-5.712738000	-0.578032000	0.238112000
C	-3.682831000	0.294617000	1.035662000
O	-3.061143000	1.003996000	1.865092000
H	-6.788683000	-0.635521000	0.302078000
N	-5.087154000	0.232739000	1.095360000
H	2.289763000	-1.023026000	-2.814157000
H	0.912885000	-2.235624000	-4.404017000
C	-2.888130000	-1.845404000	-1.820510000
C	-5.748604000	-2.139504000	-1.656029000
C	-3.631811000	-2.567209000	-2.771014000
C	-1.669006000	-2.891526000	-4.149036000
C	-2.986394000	-3.115472000	-3.919675000
C	-5.054063000	-2.737722000	-2.646355000
H	-6.823341000	-2.249338000	-1.565305000
H	-5.560076000	-3.344943000	-3.388520000
H	-3.584749000	-3.677469000	-4.628169000
H	-1.195867000	-3.263946000	-5.052079000
O	0.556160000	0.879087000	0.623135000
H	-1.658551000	0.725959000	1.780531000
N	-3.025418000	-0.392753000	0.113772000
C	-0.580891000	-1.142330000	-1.045173000
H	-1.009367000	-0.865017000	-0.100899000
C	-0.545523000	0.305824000	2.039512000
O	0.008336000	-0.269061000	2.848734000
C	-5.859997000	1.087315000	2.090419000
C	-5.466502000	0.669609000	3.509856000

C	-5.542116000	2.558520000	1.803115000
C	-7.363834000	0.874707000	1.919555000
H	-5.674428000	-0.392534000	3.665673000
H	-4.414726000	0.860657000	3.710163000
H	-6.068335000	1.243344000	4.218374000
H	-5.791700000	2.804833000	0.767325000
H	-6.153833000	3.181006000	2.459926000
H	-4.493963000	2.789591000	1.980507000
H	-7.862558000	1.520733000	2.643856000
H	-7.717089000	1.162215000	0.925804000
H	-7.669682000	-0.152459000	2.133899000
C	-0.094238000	1.953814000	0.050006000
C	-0.535445000	2.949079000	0.918274000
C	-0.329927000	2.082693000	-1.313789000
C	-1.246723000	4.036474000	0.434459000
H	-0.296691000	2.881582000	1.974632000
C	-1.028635000	3.188604000	-1.784140000
H	0.031711000	1.343568000	-2.012521000
C	-1.514725000	4.174254000	-0.927014000
H	-1.585551000	4.797614000	1.130279000
H	-1.200000000	3.279585000	-2.852226000
H	-3.386557000	5.169111000	-1.303882000
H	-2.058589000	6.262277000	-0.914984000
C	-2.314002000	5.340644000	-1.444256000
H	-2.140290000	5.499457000	-2.510820000

CO_toluene

	Coordinates (Angstroms)		
	X	Y	Z
C	0.000000000	0.000000000	-0.642706000
O	0.000000000	0.000000000	0.482029000

H_2O_toluene

	Coordinates (Angstroms)		
	X	Y	Z
O	0.000000000	0.000000000	0.118201000
H	0.000000000	0.754214000	-0.472804000
H	0.000000000	-0.754214000	-0.472804000

HCOOH_toluene

	Coordinates (Angstroms)		
	X	Y	Z
C	-0.131717000	0.398170000	0.000024000
O	-1.129069000	-0.262846000	-0.000010000
O	1.109690000	-0.090754000	-0.000008000
H	-0.100090000	1.495894000	0.000035000
H	1.045431000	-1.056121000	-0.000038000

A1_toluene

	Coordinates (Angstroms)		
	X	Y	Z
B	-2.065271000	0.258768000	0.230929000
C	-3.630348000	0.167268000	0.053155000
C	-4.407037000	-0.610870000	0.902342000
C	-4.301477000	0.817849000	-0.974877000

C	-5.777933000	-0.733115000	0.759000000
C	-5.669202000	0.706845000	-1.157235000
C	-6.409018000	-0.071168000	-0.281770000
C	-1.240948000	-1.084898000	0.313853000
C	-1.312489000	-2.025390000	-0.704212000
C	-0.342785000	-1.355674000	1.340128000
C	-0.519417000	-3.159021000	-0.731898000
C	0.455551000	-2.482961000	1.349369000
C	0.370602000	-3.383445000	0.302424000
F	-3.832832000	-1.258450000	1.921175000
F	-6.491197000	-1.473612000	1.602768000
F	-7.721343000	-0.181454000	-0.439121000
F	-6.277193000	1.333456000	-2.160750000
F	-3.622964000	1.557755000	-1.855199000
F	-0.231191000	-0.511272000	2.371338000
F	1.328279000	-2.695170000	2.332530000
F	1.158048000	-4.449869000	0.282827000
F	-0.592220000	-4.019523000	-1.745201000
F	-2.144277000	-1.826025000	-1.735904000
C	-1.239681000	4.008974000	0.527391000
C	0.167085000	3.993126000	0.363875000
C	0.848013000	2.765067000	0.155140000
C	-1.321360000	1.609505000	0.286237000
C	-1.968873000	2.854128000	0.491234000
C	3.103423000	1.558846000	-0.167120000
C	4.522560000	1.688894000	-0.193106000
C	5.248262000	0.516478000	-0.282474000
C	3.246458000	-0.767862000	-0.491126000
O	2.724727000	-1.846413000	-0.712708000
H	6.327568000	0.560389000	-0.254548000
N	4.681541000	-0.682102000	-0.390785000
C	5.505265000	-1.952030000	-0.426998000
H	-3.043891000	2.892422000	0.632743000
H	-1.727929000	4.964489000	0.688052000
C	2.294953000	2.763152000	0.027598000
C	5.165068000	2.959707000	-0.094119000
C	2.977614000	3.985897000	0.115301000
C	0.904427000	5.209269000	0.419566000
C	2.252987000	5.201665000	0.298998000
C	4.411909000	4.068804000	0.040680000
H	6.247992000	3.012410000	-0.132662000
H	4.871430000	5.047608000	0.114108000
H	2.812893000	6.129306000	0.349431000
H	0.368830000	6.140980000	0.567471000
N	2.542674000	0.371002000	-0.316403000
C	0.054895000	1.597363000	0.116178000
H	0.561918000	0.663920000	-0.060491000
C	5.041792000	-2.863748000	0.715690000
C	5.333918000	-2.613808000	-1.798192000
C	6.988716000	-1.640639000	-0.219423000
H	5.970498000	-3.500905000	-1.846196000
H	4.302424000	-2.912298000	-1.968153000
H	5.643904000	-1.928604000	-2.592589000
H	5.150312000	-2.355033000	1.677880000
H	4.006096000	-3.170100000	0.591893000
H	5.670607000	-3.757216000	0.730456000
H	7.523586000	-2.591712000	-0.214673000

H	7.412445000	-1.039459000	-1.028736000
H	7.184786000	-1.152785000	0.739535000

A2_toluene

	Coordinates (Angstroms)		
	X	Y	Z
B	-1.472618000	0.017380000	0.135846000
C	-2.187665000	0.823128000	-1.088093000
C	-3.516429000	1.222438000	-1.028141000
C	-1.496636000	1.251301000	-2.214396000
C	-4.134510000	1.965783000	-2.021205000
C	-2.076567000	1.988609000	-3.232743000
C	-3.409919000	2.345616000	-3.136444000
C	-2.441119000	-1.081968000	0.835274000
C	-2.892906000	-1.017330000	2.144979000
C	-2.899061000	-2.169259000	0.106468000
C	-3.741899000	-1.967921000	2.693347000
C	-3.741007000	-3.141827000	0.610761000
C	-4.166087000	-3.036344000	1.924201000
F	-4.268993000	0.920268000	0.040744000
F	-5.413259000	2.321147000	-1.908867000
F	-3.985074000	3.056432000	-4.101095000
F	-1.365542000	2.360455000	-4.295792000
F	-0.191214000	0.962095000	-2.362018000
F	-2.515418000	-2.309499000	-1.184966000
F	-4.142391000	-4.164934000	-0.141415000
F	-4.975924000	-3.954956000	2.440619000
F	-4.153939000	-1.860756000	3.955528000
F	-2.545063000	-0.004814000	2.943937000
C	-0.262279000	3.191721000	2.103284000
C	1.115875000	2.902380000	2.261074000
C	1.605679000	1.672760000	1.770140000
C	-0.636904000	1.035596000	1.053723000
C	-1.092932000	2.319273000	1.445849000
C	3.595592000	0.453534000	0.785779000
C	5.009838000	0.301735000	0.756942000
C	5.503561000	-0.670097000	-0.089456000
C	3.313307000	-1.173928000	-0.891440000
O	2.583870000	-1.785110000	-1.662220000
H	6.568490000	-0.844259000	-0.133617000
N	4.726706000	-1.418487000	-0.876889000
H	-2.123830000	2.603474000	1.258342000
H	-0.642716000	4.139152000	2.471881000
C	3.024374000	1.458688000	1.668038000
C	5.861738000	1.149547000	1.535989000
C	3.895948000	2.355914000	2.298635000
C	2.032737000	3.802318000	2.876795000
C	3.360378000	3.511153000	2.943182000
C	5.321273000	2.153604000	2.258067000
H	6.934532000	0.991490000	1.506371000
H	5.952612000	2.833987000	2.818420000
H	4.046212000	4.192130000	3.435432000
H	1.653860000	4.726717000	3.300979000
O	-0.311060000	-0.957349000	-0.530954000
H	0.577779000	-0.594162000	-0.714594000
C	0.669874000	0.719404000	1.329058000
H	1.032766000	-0.274305000	1.148267000

N	2.828327000	-0.259848000	-0.029563000
C	5.309535000	-2.490599000	-1.774929000
C	4.663248000	-3.831887000	-1.411078000
C	5.049545000	-2.102756000	-3.234225000
C	6.820062000	-2.614545000	-1.565259000
H	4.840456000	-4.065953000	-0.357417000
H	3.592105000	-3.822705000	-1.598665000
H	5.118634000	-4.619669000	-2.015808000
H	5.505640000	-1.133949000	-3.457158000
H	5.503308000	-2.850872000	-3.888804000
H	3.984194000	-2.050896000	-3.446519000
H	7.173457000	-3.418728000	-2.212672000
H	7.362677000	-1.709954000	-1.852910000
H	7.080386000	-2.886078000	-0.538520000
H	-0.593726000	-1.499494000	-1.276818000

A3_toluene

	Coordinates (Angstroms)		
	X	Y	Z
B	1.788788000	-0.009193000	-0.622305000
C	1.402127000	-1.271792000	0.416860000
C	1.993625000	-1.394601000	1.669170000
C	0.346402000	-2.142253000	0.217866000
C	1.588793000	-2.294258000	2.638711000
C	-0.110315000	-3.054015000	1.157161000
C	0.518556000	-3.135699000	2.382573000
C	3.420933000	0.158346000	-0.856956000
C	3.923301000	1.351032000	-1.365936000
C	4.365728000	-0.848070000	-0.721634000
C	5.259295000	1.556912000	-1.670616000
C	5.712006000	-0.689247000	-1.015385000
C	6.164747000	0.526664000	-1.490795000
F	3.019153000	-0.592021000	2.002460000
F	2.206009000	-2.355668000	3.820440000
F	0.102838000	-4.005181000	3.304251000
F	-1.157696000	-3.843534000	0.885102000
F	-0.360076000	-2.150197000	-0.952576000
F	4.009858000	-2.075845000	-0.298152000
F	6.572174000	-1.699886000	-0.855040000
F	7.454379000	0.701856000	-1.782076000
F	5.682601000	2.733353000	-2.143918000
F	3.106791000	2.390529000	-1.598110000
C	1.040403000	3.112320000	1.708697000
C	-0.355266000	3.239878000	1.539997000
C	-1.043687000	2.309964000	0.712969000
C	1.097740000	1.246746000	0.163130000
C	1.739480000	2.124047000	1.063690000
C	-3.332035000	1.397296000	0.062002000
C	-4.735286000	1.609220000	-0.055716000
C	-5.478707000	0.612000000	-0.651616000
C	-3.593038000	-0.698758000	-0.861654000
O	-3.078713000	-1.853792000	-1.217624000
H	-6.539500000	0.745395000	-0.792373000
N	-4.945022000	-0.544185000	-1.079102000
H	2.808486000	2.035067000	1.227926000
H	1.551584000	3.819272000	2.354955000
C	-2.490169000	2.404327000	0.635785000

C	-5.342997000	2.800030000	0.446291000
C	-3.146547000	3.512313000	1.219207000
C	-1.067960000	4.302443000	2.155855000
C	-2.405854000	4.462214000	1.969960000
C	-4.564364000	3.698123000	1.084147000
H	-6.410056000	2.948978000	0.330574000
H	-4.999698000	4.594031000	1.512277000
H	-2.931066000	5.299119000	2.415920000
H	-0.515065000	5.009143000	2.766714000
O	1.133210000	-0.129484000	-1.923325000
H	-2.118989000	-1.841652000	-1.040821000
N	-2.837505000	0.208226000	-0.336744000
C	-0.269891000	1.371683000	0.005382000
H	-0.744491000	0.706701000	-0.694851000
C	-5.798496000	-1.625576000	-1.753233000
C	-5.236235000	-1.880132000	-3.154575000
C	-5.787600000	-2.874860000	-0.868531000
C	-7.240327000	-1.137741000	-1.892769000
H	-5.206600000	-0.951400000	-3.730421000
H	-4.240802000	-2.316802000	-3.132857000
H	-5.900073000	-2.576674000	-3.670329000
H	-6.162445000	-2.639944000	0.131237000
H	-6.454024000	-3.617613000	-1.312091000
H	-4.798349000	-3.317958000	-0.780799000
H	-7.796472000	-1.928694000	-2.397761000
H	-7.727662000	-0.970679000	-0.928842000
H	-7.322218000	-0.240463000	-2.511233000
H	1.434782000	-0.913928000	-2.378824000

A4_toluene

	Coordinates (Angstroms)		
	X	Y	Z
B	-1.636220000	-0.050406000	0.022022000
C	-2.241681000	1.250863000	-0.757363000
C	-3.469880000	1.781670000	-0.382370000
C	-1.563382000	1.993281000	-1.709641000
C	-3.998298000	2.940401000	-0.926745000
C	-2.049279000	3.151282000	-2.288797000
C	-3.285253000	3.629235000	-1.891965000
C	-2.709756000	-1.228618000	0.267978000
C	-2.845924000	-1.941607000	1.451659000
C	-3.558908000	-1.618577000	-0.761790000
C	-3.784036000	-2.948868000	1.618618000
C	-4.501122000	-2.623112000	-0.636935000
C	-4.617039000	-3.290696000	0.569669000
F	-4.205376000	1.182161000	0.565537000
F	-5.181827000	3.397752000	-0.527735000
F	-3.777555000	4.740869000	-2.426652000
F	-1.343385000	3.803454000	-3.208209000
F	-0.329627000	1.597503000	-2.129984000
F	-3.481309000	-1.017654000	-1.959641000
F	-5.290476000	-2.953359000	-1.658646000
F	-5.516038000	-4.258519000	0.714751000
F	-3.881823000	-3.598924000	2.777793000
F	-2.057913000	-1.696953000	2.500639000
C	-0.454513000	1.625769000	3.322418000
C	0.955731000	1.557664000	3.228492000

C	1.550513000	0.942772000	2.098694000
C	-0.695322000	0.468773000	1.213463000
C	-1.258793000	1.102778000	2.347577000
C	3.724453000	0.269387000	0.892184000
C	5.149507000	0.294946000	0.907966000
C	5.793215000	-0.272315000	-0.172021000
C	3.720349000	-0.871811000	-1.170043000
O	3.108583000	-1.411339000	-2.088604000
H	6.872424000	-0.264139000	-0.205182000
N	5.150569000	-0.835004000	-1.196189000
H	-2.336682000	1.172598000	2.452209000
H	-0.891426000	2.104593000	4.193004000
C	2.997437000	0.882420000	2.000737000
C	5.880649000	0.886075000	1.983927000
C	3.765315000	1.445256000	3.036018000
C	1.776911000	2.106501000	4.250836000
C	3.128229000	2.053195000	4.155876000
C	5.204473000	1.436029000	3.010121000
H	6.965063000	0.880915000	1.955750000
H	5.730563000	1.889745000	3.842247000
H	3.752408000	2.474700000	4.936178000
H	1.302946000	2.571065000	5.109465000
O	-0.585750000	-0.766317000	-1.130865000
H	1.140423000	-1.847533000	-1.519508000
N	3.092956000	-0.304356000	-0.124334000
C	0.675523000	0.423045000	1.116233000
H	1.152963000	-0.011351000	0.259567000
C	0.214529000	-1.913404000	-0.925924000
O	-0.139692000	-2.772632000	-0.204605000
C	5.900727000	-1.431438000	-2.370747000
C	5.593041000	-2.930627000	-2.438697000
C	5.474931000	-0.694839000	-3.645412000
C	7.410530000	-1.254416000	-2.197369000
H	5.884761000	-3.422795000	-1.506559000
H	4.536361000	-3.111907000	-2.620389000
H	6.170571000	-3.374692000	-3.253080000
H	5.679955000	0.375573000	-3.552894000
H	6.053672000	-1.080895000	-4.487869000
H	4.417300000	-0.838003000	-3.852964000
H	7.891281000	-1.691571000	-3.073944000
H	7.709175000	-0.203271000	-2.156336000
H	7.800890000	-1.779608000	-1.321571000
H	-0.130648000	-0.106616000	-1.682547000

TSA2-3_toluene

	Coordinates (Angstroms)		
	X	Y	Z
B	-1.287037000	-0.147012000	-0.073898000
C	-1.712092000	1.001408000	1.025617000
C	-2.984928000	1.166966000	1.552612000
C	-0.748574000	1.851665000	1.556965000
C	-3.301163000	2.122645000	2.507263000
C	-1.020977000	2.821535000	2.506806000
C	-2.312211000	2.962002000	2.983890000
C	-2.566763000	-0.643942000	-0.985767000
C	-3.122963000	-1.915628000	-0.953371000
C	-3.191150000	0.229468000	-1.865081000

C	-4.209739000	-2.289668000	-1.732217000
C	-4.271931000	-0.094677000	-2.662468000
C	-4.788079000	-1.376558000	-2.593211000
F	-3.998100000	0.372488000	1.168365000
F	-4.546978000	2.235881000	2.971570000
F	-2.595304000	3.887220000	3.898787000
F	-0.052730000	3.614499000	2.972360000
F	0.533688000	1.752169000	1.171776000
F	-2.736997000	1.504183000	-1.970177000
F	-4.816130000	0.801887000	-3.485955000
F	-5.827019000	-1.724982000	-3.348227000
F	-4.702677000	-3.526683000	-1.657343000
F	-2.646728000	-2.864446000	-0.139006000
C	0.040555000	-2.659997000	2.690914000
C	1.322867000	-2.961684000	2.163991000
C	1.658762000	-2.421513000	0.905783000
C	-0.496637000	-1.324907000	0.716780000
C	-0.786365000	-1.780600000	2.031853000
C	3.487292000	-1.196019000	-0.231663000
C	4.859877000	-0.898402000	-0.410509000
C	5.126758000	0.281745000	-1.080551000
C	2.838936000	0.777266000	-1.261170000
O	1.864629000	1.543051000	-1.563925000
H	6.148520000	0.578474000	-1.260271000
N	4.166716000	1.131209000	-1.510733000
H	-1.703238000	-1.457215000	2.516491000
H	-0.243481000	-3.067821000	3.655807000
C	3.053303000	-2.300682000	0.575940000
C	5.841399000	-1.761827000	0.186724000
C	4.031817000	-2.975899000	1.310334000
C	2.323150000	-3.702099000	2.862808000
C	3.614483000	-3.767724000	2.419009000
C	5.429078000	-2.747921000	1.018909000
H	6.895988000	-1.585009000	0.007720000
H	6.160970000	-3.365737000	1.528182000
H	4.355661000	-4.332813000	2.973583000
H	2.048098000	-4.202208000	3.786394000
O	-0.310825000	0.527838000	-1.119088000
H	0.832714000	1.007043000	-1.224104000
N	2.573487000	-0.357409000	-0.682887000
C	0.649521000	-1.802394000	0.137782000
H	0.835713000	-1.572612000	-0.893538000
C	4.507591000	2.450336000	-2.187274000
C	3.837570000	2.472650000	-3.564071000
C	4.018082000	3.586899000	-1.284768000
C	6.017994000	2.585891000	-2.376256000
H	4.177150000	1.628522000	-4.170513000
H	2.752980000	2.441201000	-3.486628000
H	4.119259000	3.394854000	-4.076794000
H	4.501494000	3.532782000	-0.305672000
H	4.283713000	4.541317000	-1.744707000
H	2.938938000	3.559650000	-1.147336000
H	6.195557000	3.537068000	-2.880627000
H	6.561210000	2.620021000	-1.428276000
H	6.434163000	1.799318000	-3.011445000
H	-0.844067000	1.094561000	-1.682303000

TSA3-4_toluene

	Coordinates (Angstroms)		
	X	Y	Z
B	-1.536651000	-0.055488000	-0.100160000
C	-2.339577000	1.177648000	-0.854117000
C	-3.591252000	1.614569000	-0.439932000
C	-1.779365000	1.940346000	-1.866549000
C	-4.254408000	2.693161000	-1.002343000
C	-2.399906000	3.024449000	-2.462943000
C	-3.656208000	3.402957000	-2.028011000
C	-2.534865000	-1.275374000	0.342297000
C	-2.501310000	-1.930854000	1.563792000
C	-3.449205000	-1.788117000	-0.570635000
C	-3.331546000	-2.996753000	1.879308000
C	-4.294112000	-2.849592000	-0.298108000
C	-4.236152000	-3.458557000	0.943150000
F	-4.228391000	0.994186000	0.568382000
F	-5.459381000	3.056665000	-0.562404000
F	-4.276972000	4.442981000	-2.580972000
F	-1.798517000	3.705918000	-3.439020000
F	-0.532896000	1.659252000	-2.328157000
F	-3.552146000	-1.250511000	-1.796617000
F	-5.158171000	-3.292583000	-1.214661000
F	-5.038065000	-4.483249000	1.228156000
F	-3.255353000	-3.589065000	3.073792000
F	-1.628757000	-1.577053000	2.517673000
C	-0.466814000	1.957736000	3.125952000
C	0.946046000	1.944138000	3.017747000
C	1.545514000	1.260184000	1.931852000
C	-0.687089000	0.650630000	1.095942000
C	-1.258631000	1.326738000	2.202470000
C	3.710008000	0.479582000	0.829508000
C	5.126721000	0.561677000	0.749882000
C	5.729224000	-0.135206000	-0.278405000
C	3.664724000	-0.970411000	-0.995320000
O	2.980212000	-1.700007000	-1.781122000
H	6.800103000	-0.086048000	-0.400502000
N	5.049144000	-0.881934000	-1.163721000
H	-2.337324000	1.349741000	2.323424000
H	-0.914798000	2.481318000	3.964883000
C	2.990557000	1.225705000	1.834437000
C	5.869019000	1.335574000	1.697367000
C	3.763697000	1.936515000	2.771272000
C	1.768788000	2.617912000	3.961053000
C	3.123158000	2.630403000	3.834519000
C	5.200200000	1.979113000	2.676553000
H	6.949102000	1.385624000	1.619127000
H	5.738896000	2.558698000	3.417904000
H	3.738267000	3.164818000	4.549980000
H	1.294396000	3.137784000	4.787463000
O	-0.584491000	-0.726001000	-1.119241000
H	1.799324000	-1.803899000	-1.351984000
N	3.066678000	-0.305375000	-0.034164000
C	0.681736000	0.662541000	0.989728000
H	1.132478000	0.190645000	0.142985000
C	0.636693000	-2.094424000	-0.756162000
O	0.160325000	-2.878692000	-0.077937000

C	5.750336000	-1.605176000	-2.307726000
C	5.533852000	-3.111401000	-2.137791000
C	5.181771000	-1.077427000	-3.628181000
C	7.252066000	-1.320640000	-2.278280000
H	5.913799000	-3.447303000	-1.169081000
H	4.484227000	-3.382973000	-2.222577000
H	6.089676000	-3.634131000	-2.919411000
H	5.336940000	0.001964000	-3.708528000
H	5.711228000	-1.557654000	-4.453965000
H	4.120740000	-1.293080000	-3.731594000
H	7.696387000	-1.845879000	-3.125088000
H	7.482463000	-0.259169000	-2.402204000
H	7.736650000	-1.701117000	-1.375350000
H	-0.152559000	-0.077869000	-1.682591000

CO_ethanol

	Coordinates (Angstroms)		
	X	Y	Z
C	0.000000000	0.000000000	-0.642706000
O	0.000000000	0.000000000	0.482029000

H_2O_ethanol

	Coordinates (Angstroms)		
	X	Y	Z
O	0.000000000	0.000000000	0.118922000
H	0.000000000	0.753616000	-0.475689000
H	0.000000000	-0.753616000	-0.475689000

HCOOH_ethanol

	Coordinates (Angstroms)		
	X	Y	Z
C	-0.127037000	0.398889000	0.000038000
O	-1.131792000	-0.263315000	-0.000016000
O	1.106631000	-0.090429000	-0.000010000
H	-0.101549000	1.495402000	0.000025000
H	1.065065000	-1.058782000	-0.000044000

A1_ethanol

	Coordinates (Angstroms)		
	X	Y	Z
B	-2.070711000	0.263934000	0.232567000
C	-3.632509000	0.174170000	0.045390000
C	-4.414423000	-0.611484000	0.882491000
C	-4.299722000	0.835830000	-0.977641000
C	-5.784145000	-0.728850000	0.732985000
C	-5.665857000	0.729029000	-1.166104000
C	-6.410288000	-0.056035000	-0.302486000
C	-1.251205000	-1.083475000	0.333786000
C	-1.330879000	-2.040806000	-0.667455000
C	-0.351477000	-1.342951000	1.360780000
C	-0.544145000	-3.178928000	-0.677964000
C	0.442199000	-2.473552000	1.386681000
C	0.348101000	-3.391515000	0.356303000
F	-3.846903000	-1.274415000	1.897161000
F	-6.502162000	-1.478062000	1.567393000

F	-7.722872000	-0.163728000	-0.467040000
F	-6.269067000	1.367006000	-2.167350000
F	-3.617533000	1.583717000	-1.851784000
F	-0.229639000	-0.483197000	2.379681000
F	1.316529000	-2.674526000	2.371988000
F	1.132863000	-4.462502000	0.350017000
F	-0.625189000	-4.056821000	-1.676990000
F	-2.163054000	-1.858213000	-1.701738000
C	-1.229435000	4.010105000	0.524137000
C	0.177094000	3.989702000	0.361022000
C	0.855004000	2.760427000	0.146439000
C	-1.318778000	1.611170000	0.278871000
C	-1.963518000	2.856789000	0.486551000
C	3.111613000	1.556584000	-0.182142000
C	4.526437000	1.679957000	-0.173965000
C	5.255179000	0.506316000	-0.272894000
C	3.263080000	-0.760774000	-0.552140000
O	2.732495000	-1.838485000	-0.807462000
H	6.333180000	0.550408000	-0.219809000
N	4.687664000	-0.685782000	-0.420238000
C	5.517515000	-1.955626000	-0.463672000
H	-3.037970000	2.898521000	0.632462000
H	-1.713045000	4.967320000	0.689592000
C	2.304456000	2.754944000	0.024890000
C	5.174445000	2.942975000	-0.040817000
C	2.989846000	3.976212000	0.138059000
C	0.916832000	5.205365000	0.429623000
C	2.266321000	5.194196000	0.324180000
C	4.423134000	4.055983000	0.091000000
H	6.258191000	2.985764000	-0.053283000
H	4.884011000	5.032497000	0.185890000
H	2.830016000	6.118634000	0.389312000
H	0.380787000	6.136564000	0.579513000
N	2.551145000	0.367578000	-0.379385000
C	0.058281000	1.595722000	0.107299000
H	0.561537000	0.660185000	-0.069789000
C	5.042951000	-2.880321000	0.662849000
C	5.371360000	-2.595982000	-1.847250000
C	6.995467000	-1.640646000	-0.231534000
H	6.018205000	-3.475504000	-1.895714000
H	4.346974000	-2.903312000	-2.044365000
H	5.690183000	-1.894271000	-2.623249000
H	5.150985000	-2.383037000	1.631049000
H	4.006606000	-3.184389000	0.534493000
H	5.670302000	-3.774888000	0.666906000
H	7.531788000	-2.591196000	-0.238123000
H	7.421827000	-1.020303000	-1.024337000
H	7.173983000	-1.167881000	0.737998000

A2_ethanol

	Coordinates (Angstroms)		
	X	Y	Z
B	1.758262000	-0.064551000	-0.330160000
C	3.317725000	-0.422564000	-0.643625000
C	4.382261000	0.002684000	0.139128000
C	3.667698000	-1.260665000	-1.694377000
C	5.698841000	-0.351845000	-0.110128000

C	4.967218000	-1.634744000	-1.983380000
C	5.995072000	-1.170460000	-1.183112000
C	1.562658000	1.350826000	0.454689000
C	1.158035000	1.462492000	1.777325000
C	1.826265000	2.557354000	-0.176122000
C	0.995458000	2.681434000	2.416791000
C	1.677371000	3.794900000	0.420260000
C	1.251062000	3.855571000	1.734401000
F	4.180213000	0.777300000	1.216017000
F	6.681297000	0.088168000	0.677633000
F	7.253942000	-1.516769000	-1.437579000
F	5.235745000	-2.437504000	-3.013830000
F	2.710287000	-1.775782000	-2.491734000
F	2.276215000	2.558157000	-1.451953000
F	1.940607000	4.917490000	-0.249449000
F	1.098123000	5.031371000	2.337986000
F	0.601319000	2.731004000	3.690677000
F	0.935738000	0.375274000	2.527889000
C	0.825053000	-3.447060000	1.376278000
C	-0.584792000	-3.433747000	1.314095000
C	-1.261793000	-2.338118000	0.709484000
C	0.930211000	-1.308059000	0.264003000
C	1.566122000	-2.415534000	0.860963000
C	-3.543637000	-1.305675000	0.075183000
C	-4.958813000	-1.428898000	0.138910000
C	-5.703516000	-0.394712000	-0.397382000
C	-3.727966000	0.741966000	-1.070994000
O	-3.205092000	1.694717000	-1.645296000
H	-6.781030000	-0.445450000	-0.348091000
N	-5.154941000	0.671950000	-0.970265000
H	2.649514000	-2.453705000	0.922214000
H	1.311489000	-4.298372000	1.842540000
C	-2.719297000	-2.353816000	0.668634000
C	-5.596618000	-2.554192000	0.738359000
C	-3.398630000	-3.446507000	1.240256000
C	-1.321973000	-4.521095000	1.865323000
C	-2.674222000	-4.524977000	1.831583000
C	-4.832933000	-3.532718000	1.264084000
H	-6.680048000	-2.601879000	0.757717000
H	-5.283149000	-4.405356000	1.723607000
H	-3.235696000	-5.349765000	2.256633000
H	-0.777249000	-5.343870000	2.317164000
O	1.021293000	0.228993000	-1.752774000
H	0.848907000	-0.563779000	-2.278808000
C	-0.452054000	-1.291590000	0.202600000
H	-0.947355000	-0.446522000	-0.247798000
N	-3.000888000	-0.249105000	-0.522341000
C	-6.013185000	1.800417000	-1.510902000
C	-5.637432000	3.092460000	-0.777891000
C	-5.799609000	1.902788000	-3.023879000
C	-7.494689000	1.523311000	-1.252876000
H	-5.783268000	2.970818000	0.299197000
H	-4.606607000	3.382972000	-0.967003000
H	-6.295960000	3.893183000	-1.122937000
H	-6.051370000	0.954106000	-3.506484000
H	-6.464846000	2.673897000	-3.420017000
H	-4.773171000	2.165022000	-3.270250000

H	-8.053005000	2.376291000	-1.642634000
H	-7.853058000	0.632385000	-1.774985000
H	-7.724547000	1.438976000	-0.187375000
H	1.449592000	0.880479000	-2.326095000

A3_ethanol

	Coordinates (Angstroms)		
	X	Y	Z
B	1.869049000	-0.033448000	-0.691767000
C	1.294097000	-1.255342000	0.296339000
C	1.650209000	-1.303582000	1.638993000
C	0.305226000	-2.158775000	-0.051893000
C	1.088345000	-2.161252000	2.564842000
C	-0.301396000	-3.032197000	0.840200000
C	0.093910000	-3.036616000	2.161668000
C	3.528482000	0.020856000	-0.754121000
C	4.142445000	1.116695000	-1.351314000
C	4.408955000	-0.978165000	-0.366222000
C	5.510935000	1.247151000	-1.513213000
C	5.786290000	-0.893181000	-0.508627000
C	6.344305000	0.230941000	-1.083777000
F	2.596680000	-0.466388000	2.106395000
F	1.484048000	-2.152528000	3.841451000
F	-0.470063000	-3.870054000	3.037318000
F	-1.265167000	-3.864939000	0.430556000
F	-0.167449000	-2.236110000	-1.323377000
F	3.962411000	-2.129503000	0.172248000
F	6.577736000	-1.893024000	-0.105093000
F	7.665864000	0.331532000	-1.232212000
F	6.034673000	2.336792000	-2.084421000
F	3.402230000	2.138096000	-1.821820000
C	1.194061000	3.334402000	1.304776000
C	-0.207283000	3.443117000	1.179052000
C	-0.928879000	2.433998000	0.485179000
C	1.202538000	1.298667000	-0.008196000
C	1.875616000	2.279854000	0.749080000
C	-3.258016000	1.497595000	-0.052147000
C	-4.663281000	1.684812000	-0.034867000
C	-5.454522000	0.643777000	-0.489191000
C	-3.575394000	-0.606546000	-0.941344000
O	-3.081516000	-1.731932000	-1.405994000
H	-6.527840000	0.747508000	-0.477818000
N	-4.949193000	-0.509129000	-0.938710000
H	2.950642000	2.215705000	0.887575000
H	1.723410000	4.107261000	1.854200000
C	-2.383585000	2.523708000	0.451577000
C	-5.249355000	2.883090000	0.466882000
C	-3.011011000	3.664750000	0.998548000
C	-0.891388000	4.562149000	1.734404000
C	-2.236420000	4.685029000	1.622810000
C	-4.434759000	3.833215000	0.971857000
H	-6.326586000	2.998794000	0.448134000
H	-4.844658000	4.748372000	1.383718000
H	-2.751295000	5.547437000	2.030980000
H	-0.310195000	5.325454000	2.242189000
O	1.355606000	-0.128603000	-2.067820000
H	-2.112239000	-1.693043000	-1.375222000

N	-2.781768000	0.325034000	-0.521961000
C	-0.173092000	1.407499000	-0.120095000
H	-0.691565000	0.654390000	-0.687921000
C	-5.858221000	-1.656062000	-1.406757000
C	-5.581176000	-1.919940000	-2.888176000
C	-5.592355000	-2.875340000	-0.520484000
C	-7.321529000	-1.250546000	-1.246221000
H	-5.727782000	-1.006480000	-3.470229000
H	-4.577642000	-2.300512000	-3.066488000
H	-6.296808000	-2.666681000	-3.238529000
H	-5.762929000	-2.627612000	0.530387000
H	-6.299328000	-3.657488000	-0.804987000
H	-4.585221000	-3.270416000	-0.634747000
H	-7.920954000	-2.099380000	-1.578464000
H	-7.586508000	-1.049625000	-0.205219000
H	-7.591935000	-0.395459000	-1.870455000
H	1.607007000	-0.965467000	-2.459800000

A4_ethanol

	Coordinates (Angstroms)		
	X	Y	Z
B	-1.687105000	0.094891000	-0.083376000
C	-3.014219000	0.730576000	-0.773175000
C	-4.271787000	0.625105000	-0.192108000
C	-2.968680000	1.523811000	-1.910009000
C	-5.401931000	1.234877000	-0.712551000
C	-4.068962000	2.143391000	-2.470412000
C	-5.302640000	1.993328000	-1.863476000
C	-1.951659000	-1.289789000	0.699315000
C	-1.631559000	-1.505354000	2.031289000
C	-2.553685000	-2.363545000	0.053278000
C	-1.877959000	-2.706140000	2.679419000
C	-2.810417000	-3.575966000	0.662213000
C	-2.467706000	-3.748366000	1.991904000
F	-4.447955000	-0.061191000	0.945819000
F	-6.581952000	1.100004000	-0.108752000
F	-6.378471000	2.579950000	-2.377537000
F	-3.951942000	2.879629000	-3.573649000
F	-1.786291000	1.728227000	-2.537548000
F	-2.901843000	-2.254672000	-1.239890000
F	-3.380737000	-4.575201000	-0.012487000
F	-2.704916000	-4.906685000	2.600651000
F	-1.550839000	-2.861593000	3.963557000
F	-1.070372000	-0.545843000	2.777200000
C	-0.576884000	3.217748000	1.926965000
C	0.831266000	3.115140000	1.922212000
C	1.458150000	2.067773000	1.197079000
C	-0.763183000	1.206217000	0.598079000
C	-1.354245000	2.302244000	1.266524000
C	3.676694000	1.081648000	0.342826000
C	5.086020000	1.024030000	0.513803000
C	5.766825000	0.079754000	-0.232586000
C	3.771329000	-0.516904000	-1.376824000
O	3.235155000	-1.132464000	-2.296954000
H	6.831212000	-0.035720000	-0.092151000
N	5.167257000	-0.707304000	-1.120795000
H	-2.434390000	2.411666000	1.284218000

H	-1.034172000	4.038444000	2.470828000
C	2.909861000	1.999027000	1.176404000
C	5.775012000	1.893246000	1.410882000
C	3.639796000	2.888254000	1.985258000
C	1.619687000	4.037627000	2.669570000
C	2.966386000	3.907582000	2.724815000
C	5.070503000	2.814673000	2.099517000
H	6.851890000	1.808077000	1.507961000
H	5.565971000	3.511308000	2.766315000
H	3.564061000	4.587115000	3.322813000
H	1.117282000	4.830820000	3.213991000
O	-0.746158000	-0.446169000	-1.474353000
H	0.613522000	-1.414426000	-2.648040000
N	3.089322000	0.333177000	-0.587710000
C	0.613579000	1.114878000	0.580124000
H	1.090303000	0.286557000	0.087069000
C	0.201271000	-1.435971000	-1.636375000
O	0.479894000	-2.181465000	-0.759540000
C	5.942540000	-1.768760000	-1.878738000
C	5.278236000	-3.126321000	-1.626768000
C	5.970088000	-1.397638000	-3.364322000
C	7.384611000	-1.845197000	-1.375036000
H	5.264621000	-3.346881000	-0.555586000
H	4.260259000	-3.160081000	-2.008602000
H	5.864571000	-3.900297000	-2.127587000
H	6.420812000	-0.410193000	-3.499206000
H	6.584813000	-2.127393000	-3.897153000
H	4.973713000	-1.395106000	-3.800519000
H	7.873408000	-2.656051000	-1.918130000
H	7.949251000	-0.931281000	-1.577267000
H	7.443374000	-2.080801000	-0.308953000
H	-0.792297000	0.139958000	-2.247682000

TSA2-3_ethanol

	Coordinates (Angstroms)		
	X	Y	Z
B	-1.278919000	-0.174990000	-0.143266000
C	-1.661321000	0.697072000	1.193774000
C	-2.868294000	0.539182000	1.861020000
C	-0.774401000	1.570956000	1.805094000
C	-3.199995000	1.219271000	3.020865000
C	-1.064884000	2.275318000	2.961943000
C	-2.292007000	2.102577000	3.573196000
C	-2.579618000	-0.383687000	-1.120888000
C	-3.021829000	-1.604757000	-1.608237000
C	-3.319075000	0.715547000	-1.545159000
C	-4.121238000	-1.737483000	-2.443677000
C	-4.419475000	0.627182000	-2.378165000
C	-4.824970000	-0.614791000	-2.832398000
F	-3.792704000	-0.327889000	1.406216000
F	-4.381646000	1.025808000	3.612594000
F	-2.590143000	2.768100000	4.688639000
F	-0.169094000	3.109845000	3.496200000
F	0.454590000	1.764621000	1.295806000
F	-2.986315000	1.951787000	-1.135708000
F	-5.091995000	1.720522000	-2.746789000
F	-5.880867000	-0.726071000	-3.636324000

F	-4.502734000	-2.941222000	-2.879656000
F	-2.400850000	-2.749547000	-1.285506000
C	-0.007201000	-3.261212000	2.033541000
C	1.318078000	-3.373512000	1.537290000
C	1.682099000	-2.543812000	0.459310000
C	-0.524618000	-1.525068000	0.388069000
C	-0.856178000	-2.294270000	1.538577000
C	3.497230000	-1.048280000	-0.338807000
C	4.861477000	-0.719140000	-0.473087000
C	5.118285000	0.554680000	-0.960223000
C	2.825096000	1.059071000	-1.035239000
O	1.840802000	1.849592000	-1.234579000
H	6.138115000	0.876717000	-1.104708000
N	4.150339000	1.444332000	-1.257808000
H	-1.814242000	-2.142490000	2.028522000
H	-0.315102000	-3.892548000	2.861571000
C	3.079277000	-2.303201000	0.220546000
C	5.851418000	-1.661419000	-0.034287000
C	4.058870000	-3.101026000	0.814314000
C	2.328113000	-4.214398000	2.102626000
C	3.634276000	-4.130711000	1.710129000
C	5.453514000	-2.793329000	0.600084000
H	6.902908000	-1.437180000	-0.173879000
H	6.192995000	-3.483092000	0.992808000
H	4.380767000	-4.782232000	2.151706000
H	2.043175000	-4.921552000	2.875696000
O	-0.310034000	0.700523000	-1.034729000
H	0.777319000	1.241307000	-1.015473000
N	2.570248000	-0.142238000	-0.603341000
C	0.679174000	-1.808008000	-0.204202000
H	0.916291000	-1.336437000	-1.137266000
C	4.481117000	2.835286000	-1.785034000
C	3.808661000	2.999834000	-3.150510000
C	3.988941000	3.862775000	-0.762690000
C	5.988838000	2.997969000	-1.961232000
H	4.160961000	2.231177000	-3.843744000
H	2.723699000	2.947340000	-3.081775000
H	4.081942000	3.976537000	-3.555779000
H	4.481342000	3.705210000	0.200723000
H	4.249314000	4.861033000	-1.121898000
H	2.910549000	3.817192000	-0.621705000
H	6.157533000	4.000578000	-2.357818000
H	6.531327000	2.924002000	-1.015656000
H	6.402577000	2.285910000	-2.679795000
H	-0.457449000	0.492634000	-1.961202000

TSA3-4_ethanol

	Coordinates (Angstroms)		
	X	Y	Z
B	-1.536649000	-0.047812000	-0.104827000
C	-2.370039000	1.165962000	-0.855613000
C	-3.645951000	1.557131000	-0.471498000
C	-1.802322000	1.967209000	-1.834735000
C	-4.324683000	2.625813000	-1.033375000
C	-2.439661000	3.042820000	-2.428086000
C	-3.719170000	3.373970000	-2.025465000
C	-2.509368000	-1.285343000	0.354990000

C	-2.454097000	-1.931499000	1.580280000
C	-3.421490000	-1.825055000	-0.543870000
C	-3.263337000	-3.007482000	1.913100000
C	-4.246565000	-2.896778000	-0.254723000
C	-4.168988000	-3.492569000	0.990894000
F	-4.298391000	0.899574000	0.505205000
F	-5.554763000	2.942707000	-0.622811000
F	-4.355635000	4.405918000	-2.577285000
F	-1.830654000	3.763227000	-3.372008000
F	-0.536059000	1.735695000	-2.258795000
F	-3.543250000	-1.303577000	-1.777757000
F	-5.111023000	-3.362069000	-1.160971000
F	-4.952306000	-4.527053000	1.293319000
F	-3.164855000	-3.588170000	3.112582000
F	-1.574125000	-1.560983000	2.524085000
C	-0.463635000	2.011204000	3.090472000
C	0.949565000	2.002325000	2.977705000
C	1.548021000	1.307465000	1.899354000
C	-0.685988000	0.676981000	1.076766000
C	-1.257219000	1.364495000	2.176640000
C	3.710309000	0.515913000	0.794499000
C	5.120945000	0.597440000	0.713103000
C	5.727571000	-0.127536000	-0.299111000
C	3.659248000	-0.959835000	-1.007350000
O	2.967475000	-1.686615000	-1.791097000
H	6.799709000	-0.084410000	-0.413793000
N	5.046464000	-0.889762000	-1.163791000
H	-2.336202000	1.387459000	2.301109000
H	-0.910052000	2.545583000	3.923774000
C	2.993876000	1.275319000	1.793924000
C	5.865753000	1.387885000	1.641890000
C	3.767425000	2.002649000	2.715695000
C	1.774080000	2.694902000	3.910836000
C	3.126102000	2.710453000	3.774778000
C	5.201880000	2.049271000	2.615435000
H	6.945315000	1.434240000	1.554631000
H	5.742746000	2.642110000	3.344739000
H	3.744977000	3.256809000	4.478248000
H	1.300689000	3.223804000	4.732189000
O	-0.588901000	-0.715291000	-1.135184000
H	1.771579000	-1.775609000	-1.360965000
N	3.060022000	-0.278933000	-0.057331000
C	0.682876000	0.694737000	0.965858000
H	1.137368000	0.219453000	0.122204000
C	0.615387000	-2.047187000	-0.760732000
O	0.148073000	-2.825665000	-0.062637000
C	5.754472000	-1.656016000	-2.277814000
C	5.509373000	-3.152361000	-2.068594000
C	5.220523000	-1.152243000	-3.621033000
C	7.259071000	-1.398072000	-2.226216000
H	5.867361000	-3.462343000	-1.083073000
H	4.457905000	-3.414416000	-2.165163000
H	6.073703000	-3.702344000	-2.825077000
H	5.392661000	-0.076991000	-3.719407000
H	5.765302000	-1.659131000	-4.420726000
H	4.159170000	-1.356122000	-3.746750000
H	7.706808000	-1.961485000	-3.046604000

H	7.508747000	-0.344935000	-2.378619000
H	7.713438000	-1.754779000	-1.298367000
H	-0.110809000	-0.075627000	-1.673097000

CO_methanol

	Coordinates (Angstroms)		
	X	Y	Z
C	0.000000000	0.000000000	-0.642605000
O	0.000000000	0.000000000	0.481954000

H_2O_methanol

	Coordinates (Angstroms)		
	X	Y	Z
O	0.000000000	0.000000000	0.118655000
H	0.000000000	0.754714000	-0.474621000
H	0.000000000	-0.754714000	-0.474621000

HCOOH_methanol

	Coordinates (Angstroms)		
	X	Y	Z
C	-0.126311000	0.399581000	0.000015000
O	-1.131534000	-0.263587000	-0.000006000
O	1.105987000	-0.090695000	-0.000006000
H	-0.102469000	1.495737000	0.000039000
H	1.064710000	-1.058976000	-0.000036000

A1_methanol

	Coordinates (Angstroms)		
	X	Y	Z
B	-2.067131000	0.268062000	0.233271000
C	-3.628033000	0.180178000	0.039904000
C	-4.414343000	-0.603784000	0.874443000
C	-4.290201000	0.842231000	-0.986149000
C	-5.783600000	-0.719193000	0.719569000
C	-5.655658000	0.737116000	-1.180054000
C	-6.404603000	-0.046317000	-0.318912000
C	-1.251075000	-1.081221000	0.341163000
C	-1.328542000	-2.040484000	-0.658404000
C	-0.361992000	-1.343490000	1.376434000
C	-0.550475000	-3.184484000	-0.658664000
C	0.422638000	-2.480468000	1.413322000
C	0.329201000	-3.401983000	0.385572000
F	-3.851899000	-1.266780000	1.891930000
F	-6.506071000	-1.466694000	1.551710000
F	-7.716675000	-0.152276000	-0.488666000
F	-6.253999000	1.375242000	-2.184178000
F	-3.603381000	1.588849000	-1.857856000
F	-0.243078000	-0.481633000	2.393758000
F	1.284090000	-2.686813000	2.408722000
F	1.103364000	-4.480944000	0.390890000
F	-0.629599000	-4.065055000	-1.655189000
F	-2.150393000	-1.854670000	-1.700137000
C	-1.221224000	4.012445000	0.529502000
C	0.185547000	3.990043000	0.369081000
C	0.861688000	2.760339000	0.151733000

C	-1.313484000	1.614026000	0.281016000
C	-1.957043000	2.860308000	0.488129000
C	3.115876000	1.556146000	-0.186453000
C	4.530486000	1.675385000	-0.173235000
C	5.256156000	0.500349000	-0.277875000
C	3.263673000	-0.755929000	-0.581950000
O	2.729009000	-1.829365000	-0.857339000
H	6.333897000	0.540150000	-0.218181000
N	4.685456000	-0.688922000	-0.437881000
C	5.512725000	-1.961244000	-0.481743000
H	-3.031762000	2.903318000	0.631749000
H	-1.703564000	4.970062000	0.696322000
C	2.311139000	2.753351000	0.030694000
C	5.181458000	2.935529000	-0.028054000
C	2.998799000	3.972588000	0.152628000
C	0.927371000	5.204149000	0.444360000
C	2.277185000	5.190981000	0.343427000
C	4.432240000	4.049314000	0.109551000
H	6.265310000	2.975500000	-0.036697000
H	4.895128000	5.024087000	0.212386000
H	2.842403000	6.114000000	0.414933000
H	0.392536000	6.135663000	0.596595000
N	2.553208000	0.369997000	-0.399394000
C	0.063788000	1.596539000	0.111833000
H	0.565392000	0.659966000	-0.064584000
C	5.019832000	-2.893990000	0.630155000
C	5.386004000	-2.588015000	-1.873452000
C	6.987880000	-1.652138000	-0.225259000
H	6.033829000	-3.466823000	-1.921001000
H	4.365262000	-2.894508000	-2.090206000
H	5.715869000	-1.878729000	-2.637773000
H	5.125148000	-2.408384000	1.604530000
H	3.981455000	-3.186390000	0.491064000
H	5.638200000	-3.794716000	0.628886000
H	7.520780000	-2.604640000	-0.228177000
H	7.428502000	-1.029593000	-1.008419000
H	7.152838000	-1.184570000	0.749132000

A2_methanol

	Coordinates (Angstroms)		
	X	Y	Z
B	-1.649055000	-0.075239000	-0.231499000
C	-2.350500000	1.407009000	-0.225559000
C	-3.588426000	1.646294000	0.356501000
C	-1.693412000	2.546870000	-0.664992000
C	-4.153219000	2.904744000	0.468845000
C	-2.221036000	3.823989000	-0.580873000
C	-3.466052000	4.004982000	-0.009703000
C	-2.668334000	-1.324004000	-0.485445000
C	-2.498974000	-2.575250000	0.095796000
C	-3.744600000	-1.232930000	-1.362163000
C	-3.352074000	-3.643613000	-0.136049000
C	-4.616602000	-2.274401000	-1.621544000
C	-4.419415000	-3.492936000	-0.999196000
F	-4.311587000	0.630702000	0.861797000
F	-5.350647000	3.065227000	1.034101000
F	-3.992918000	5.223353000	0.087086000

F	-1.536723000	4.875527000	-1.033067000
F	-0.453174000	2.459945000	-1.195322000
F	-3.989113000	-0.086758000	-2.018923000
F	-5.636917000	-2.117721000	-2.466941000
F	-5.243433000	-4.509682000	-1.235634000
F	-3.142265000	-4.819881000	0.458452000
F	-1.474693000	-2.825784000	0.921875000
C	-0.536760000	-0.128766000	3.489690000
C	0.873148000	-0.206623000	3.408485000
C	1.505011000	-0.230615000	2.137524000
C	-0.715337000	-0.160114000	1.077810000
C	-1.308807000	-0.090138000	2.360714000
C	3.738386000	-0.207221000	0.848056000
C	5.153680000	-0.307144000	0.925095000
C	5.851390000	-0.247213000	-0.266945000
C	3.837109000	0.044649000	-1.489117000
O	3.267626000	0.222853000	-2.567988000
H	6.927526000	-0.332470000	-0.250328000
N	5.259343000	-0.086977000	-1.447913000
H	-2.388482000	-0.024045000	2.457161000
H	-0.995883000	-0.100978000	4.473113000
C	2.958482000	-0.277098000	2.078758000
C	5.835117000	-0.449643000	2.170392000
C	3.680932000	-0.372023000	3.283732000
C	1.651151000	-0.271848000	4.598613000
C	3.000827000	-0.369992000	4.537170000
C	5.115317000	-0.468442000	3.310207000
H	6.916779000	-0.530132000	2.175957000
H	5.601625000	-0.560786000	4.274797000
H	3.592642000	-0.436203000	5.443695000
H	1.140610000	-0.253279000	5.556200000
O	-0.669210000	-0.195175000	-1.506707000
H	0.007851000	0.491684000	-1.594046000
C	0.659184000	-0.230098000	0.997807000
H	1.143020000	-0.290535000	0.038776000
N	3.154605000	-0.033381000	-0.334540000
C	6.071061000	-0.033498000	-2.730208000
C	5.625020000	-1.182406000	-3.640097000
C	5.875665000	1.340411000	-3.378960000
C	7.560654000	-0.213398000	-2.434427000
H	5.764117000	-2.141750000	-3.133730000
H	4.583579000	-1.086288000	-3.938503000
H	6.246639000	-1.177804000	-4.538759000
H	6.190929000	2.130103000	-2.691123000
H	6.501096000	1.396531000	-4.273217000
H	4.841130000	1.512986000	-3.667157000
H	8.084096000	-0.177092000	-3.391457000
H	7.963869000	0.588331000	-1.810198000
H	7.781383000	-1.180363000	-1.974851000
H	-1.110513000	-0.262174000	-2.364417000

A3_methanol

	Coordinates (Angstroms)		
	X	Y	Z
B	1.866835000	-0.030506000	-0.688008000
C	1.295889000	-1.254625000	0.298044000
C	1.656521000	-1.304664000	1.639407000

C	0.307248000	-2.159127000	-0.048597000
C	1.099056000	-2.164788000	2.565589000
C	-0.294993000	-3.034928000	0.844075000
C	0.104635000	-3.040949000	2.164227000
C	3.525417000	0.025784000	-0.759053000
C	4.141352000	1.125182000	-1.348058000
C	4.404760000	-0.977741000	-0.379445000
C	5.510122000	1.254317000	-1.509111000
C	5.782092000	-0.893967000	-0.521304000
C	6.341912000	0.233661000	-1.087682000
F	2.603643000	-0.466990000	2.104854000
F	1.499153000	-2.157560000	3.840796000
F	-0.455101000	-3.876652000	3.040279000
F	-1.258588000	-3.868413000	0.436053000
F	-0.169391000	-2.235635000	-1.318545000
F	3.956603000	-2.132385000	0.150134000
F	6.571721000	-1.898221000	-0.125688000
F	7.663474000	0.333285000	-1.235325000
F	6.035330000	2.347439000	-2.071970000
F	3.404366000	2.152834000	-1.811409000
C	1.189638000	3.335149000	1.310220000
C	-0.211643000	3.443464000	1.182641000
C	-0.932141000	2.433923000	0.488517000
C	1.200346000	1.300039000	-0.003214000
C	1.872451000	2.281069000	0.755114000
C	-3.260374000	1.496026000	-0.050982000
C	-4.665349000	1.683527000	-0.036972000
C	-5.455879000	0.641896000	-0.491650000
C	-3.575848000	-0.609446000	-0.937682000
O	-3.079957000	-1.734789000	-1.399668000
H	-6.529196000	0.746143000	-0.483166000
N	-4.949592000	-0.511563000	-0.938360000
H	2.947402000	2.217568000	0.894532000
H	1.717995000	4.108206000	1.860321000
C	-2.386855000	2.523132000	0.452751000
C	-5.252312000	2.882603000	0.461695000
C	-3.015136000	3.664739000	0.997108000
C	-0.896747000	4.562991000	1.736247000
C	-2.241465000	4.685690000	1.621889000
C	-4.438761000	3.833416000	0.967242000
H	-6.329508000	2.998248000	0.440145000
H	-4.849418000	4.749176000	1.377012000
H	-2.757177000	5.548484000	2.028219000
H	-0.316445000	5.326671000	2.244484000
O	1.344390000	-0.124439000	-2.063803000
H	-2.111293000	-1.694546000	-1.364251000
N	-2.782890000	0.322801000	-0.517613000
C	-0.175150000	1.407626000	-0.116037000
H	-0.693927000	0.654212000	-0.683246000
C	-5.857409000	-1.658693000	-1.407966000
C	-5.577202000	-1.922459000	-2.888762000
C	-5.593413000	-2.877809000	-0.520873000
C	-7.321060000	-1.253420000	-1.250708000
H	-5.724350000	-1.009272000	-3.471087000
H	-4.572696000	-2.301254000	-3.065334000
H	-6.291038000	-2.670479000	-3.240079000
H	-5.766095000	-2.629612000	0.529530000

H	-6.300064000	-3.659769000	-0.806724000
H	-4.586154000	-3.273343000	-0.632792000
H	-7.919479000	-2.102451000	-1.584344000
H	-7.588143000	-1.052325000	-0.210291000
H	-7.589961000	-0.398327000	-1.875542000
H	1.598187000	-0.958710000	-2.460409000

A4_methanol

	Coordinates (Angstroms)		
	X	Y	Z
B	-1.685116000	0.092635000	-0.099953000
C	-3.011186000	0.729535000	-0.792051000
C	-4.258245000	0.662813000	-0.182855000
C	-2.978592000	1.481487000	-1.956667000
C	-5.390158000	1.272126000	-0.699503000
C	-4.081351000	2.100041000	-2.513749000
C	-5.303768000	1.990966000	-1.876446000
C	-1.965368000	-1.283444000	0.695398000
C	-1.646469000	-1.493405000	2.028835000
C	-2.581236000	-2.354658000	0.058290000
C	-1.908979000	-2.685111000	2.686653000
C	-2.854071000	-3.558868000	0.677245000
C	-2.513336000	-3.724968000	2.007913000
F	-4.419752000	0.018370000	0.981757000
F	-6.558694000	1.175464000	-0.066666000
F	-6.380862000	2.578504000	-2.386989000
F	-3.977273000	2.795277000	-3.644647000
F	-1.809215000	1.640949000	-2.620071000
F	-2.927855000	-2.252632000	-1.236091000
F	-3.437730000	-4.555627000	0.010525000
F	-2.765513000	-4.874982000	2.626322000
F	-1.582975000	-2.834919000	3.971722000
F	-1.070464000	-0.536092000	2.766181000
C	-0.573189000	3.212656000	1.910266000
C	0.834636000	3.108208000	1.910877000
C	1.463778000	2.060978000	1.187050000
C	-0.757196000	1.203119000	0.577879000
C	-1.349154000	2.298251000	1.247151000
C	3.689657000	1.070092000	0.352835000
C	5.098363000	1.017801000	0.528016000
C	5.786879000	0.080409000	-0.219823000
C	3.796140000	-0.539062000	-1.355614000
O	3.257952000	-1.171498000	-2.265990000
H	6.852357000	-0.025100000	-0.080339000
N	5.194338000	-0.710643000	-1.109553000
H	-2.429118000	2.408803000	1.263756000
H	-1.031406000	4.033446000	2.453207000
C	2.916376000	1.990257000	1.176858000
C	5.781850000	1.889413000	1.426725000
C	3.641745000	2.880616000	1.989270000
C	1.618974000	4.030226000	2.662977000
C	2.965162000	3.900487000	2.724921000
C	5.071714000	2.809013000	2.111732000
H	6.858537000	1.807147000	1.527898000
H	5.562201000	3.507145000	2.780641000
H	3.560051000	4.580041000	3.325624000
H	1.113486000	4.822991000	3.205160000

O	-0.750648000	-0.475274000	-1.478859000
H	0.499544000	-1.575115000	-2.655507000
N	3.108535000	0.311961000	-0.574543000
C	0.620105000	1.111618000	0.562049000
H	1.097019000	0.285579000	0.065339000
C	0.168345000	-1.489469000	-1.617188000
O	0.508112000	-2.151484000	-0.694630000
C	5.983807000	-1.755018000	-1.878239000
C	5.349038000	-3.127027000	-1.629026000
C	5.995811000	-1.374146000	-3.361649000
C	7.430696000	-1.806615000	-1.385124000
H	5.337565000	-3.348600000	-0.558062000
H	4.333515000	-3.186395000	-2.013775000
H	5.954968000	-3.886684000	-2.128339000
H	6.432903000	-0.380168000	-3.492933000
H	6.616736000	-2.092390000	-3.902795000
H	4.997869000	-1.381860000	-3.794275000
H	7.931412000	-2.602845000	-1.938848000
H	7.975671000	-0.880086000	-1.583375000
H	7.501773000	-2.050250000	-0.321592000
H	-0.854607000	0.039256000	-2.295925000

TSA2-3_methanol

	Coordinates (Angstroms)		
	X	Y	Z
B	-1.276933000	-0.176311000	-0.148839000
C	-1.653358000	0.703949000	1.184785000
C	-2.850641000	0.530741000	1.865921000
C	-0.777253000	1.600309000	1.778540000
C	-3.183628000	1.215271000	3.022495000
C	-1.069369000	2.309771000	2.932048000
C	-2.286655000	2.120302000	3.557472000
C	-2.584046000	-0.386788000	-1.117702000
C	-3.019485000	-1.607351000	-1.612231000
C	-3.334066000	0.709869000	-1.529733000
C	-4.122505000	-1.742253000	-2.442478000
C	-4.438666000	0.619346000	-2.356855000
C	-4.837269000	-0.622212000	-2.818294000
F	-3.763382000	-0.357325000	1.427599000
F	-4.355830000	1.006062000	3.627406000
F	-2.585824000	2.790633000	4.669725000
F	-0.184073000	3.166125000	3.448861000
F	0.442420000	1.814592000	1.254827000
F	-3.007794000	1.945987000	-1.113900000
F	-5.121617000	1.710262000	-2.713389000
F	-5.897011000	-0.735619000	-3.616886000
F	-4.496682000	-2.945523000	-2.886201000
F	-2.386789000	-2.749679000	-1.303043000
C	-0.011034000	-3.256592000	2.041295000
C	1.315512000	-3.370188000	1.548838000
C	1.681864000	-2.544224000	0.468851000
C	-0.524987000	-1.525783000	0.388800000
C	-0.859124000	-2.291491000	1.541073000
C	3.498931000	-1.052491000	-0.332099000
C	4.863081000	-0.723739000	-0.464902000
C	5.120851000	0.547986000	-0.957443000
C	2.828106000	1.049844000	-1.043861000

O	1.842209000	1.836997000	-1.254485000
H	6.141065000	0.869612000	-1.100036000
N	4.153415000	1.435494000	-1.262924000
H	-1.818332000	-2.139020000	2.028458000
H	-0.320844000	-3.885198000	2.870701000
C	3.079658000	-2.304386000	0.232393000
C	5.852174000	-1.663509000	-0.019225000
C	4.058016000	-3.099686000	0.831636000
C	2.324371000	-4.208799000	2.119851000
C	3.631452000	-4.126097000	1.730402000
C	5.453002000	-2.792658000	0.619448000
H	6.903878000	-1.439438000	-0.157392000
H	6.191701000	-3.480442000	1.017092000
H	4.377085000	-4.775651000	2.176312000
H	2.037592000	-4.913249000	2.894722000
O	-0.310963000	0.690890000	-1.050615000
H	0.778984000	1.230254000	-1.035565000
N	2.572388000	-0.147887000	-0.604134000
C	0.680237000	-1.811088000	-0.199707000
H	0.919365000	-1.343033000	-1.133966000
C	4.485867000	2.825339000	-1.792800000
C	3.820280000	2.986356000	-3.161994000
C	3.988670000	3.855078000	-0.775120000
C	5.994327000	2.988321000	-1.961955000
H	4.174677000	2.214804000	-3.850880000
H	2.734818000	2.936391000	-3.099288000
H	4.097715000	3.961236000	-3.568818000
H	4.476875000	3.699847000	0.190788000
H	4.250574000	4.852433000	-1.135672000
H	2.909632000	3.810471000	-0.638568000
H	6.164274000	3.990317000	-2.359573000
H	6.532110000	2.916287000	-1.013547000
H	6.411762000	2.275009000	-2.677130000
H	-0.445486000	0.456078000	-1.972904000

TSA3-4_methanol

	Coordinates (Angstroms)		
	X	Y	Z
B	-1.536166000	-0.045261000	-0.104933000
C	-2.361669000	1.180081000	-0.845018000
C	-3.639236000	1.570006000	-0.465250000
C	-1.784570000	1.991876000	-1.809982000
C	-4.311434000	2.646666000	-1.019703000
C	-2.415301000	3.075593000	-2.395556000
C	-3.697070000	3.404866000	-1.998603000
C	-2.515375000	-1.283322000	0.340419000
C	-2.466310000	-1.942230000	1.559139000
C	-3.426888000	-1.810631000	-0.566299000
C	-3.281412000	-3.017637000	1.879059000
C	-4.257769000	-2.881298000	-0.290256000
C	-4.186677000	-3.489557000	0.949727000
F	-4.300592000	0.902645000	0.498714000
F	-5.543923000	2.961356000	-0.614609000
F	-4.327152000	4.444608000	-2.543060000
F	-1.797654000	3.805756000	-3.326281000
F	-0.515498000	1.762924000	-2.226623000
F	-3.541697000	-1.276614000	-1.795686000

F	-5.121593000	-3.333522000	-1.203655000
F	-4.975341000	-4.523499000	1.239735000
F	-3.188625000	-3.610708000	3.072914000
F	-1.586566000	-1.586179000	2.508865000
C	-0.461743000	1.966884000	3.118991000
C	0.951710000	1.952831000	3.009723000
C	1.549738000	1.271439000	1.922599000
C	-0.684912000	0.662252000	1.086137000
C	-1.255921000	1.336603000	2.194210000
C	3.710785000	0.486923000	0.811241000
C	5.121190000	0.568397000	0.729814000
C	5.725635000	-0.134715000	-0.299248000
C	3.656654000	-0.960358000	-1.012784000
O	2.963128000	-1.680708000	-1.801690000
H	6.797271000	-0.085656000	-0.416748000
N	5.042133000	-0.879158000	-1.177523000
H	-2.335115000	1.362326000	2.316488000
H	-0.907793000	2.491466000	3.958699000
C	2.995673000	1.234098000	1.820481000
C	5.867544000	1.340577000	1.672526000
C	3.770302000	1.943956000	2.754752000
C	1.777133000	2.627010000	3.955636000
C	3.129549000	2.638218000	3.823221000
C	5.205029000	1.986740000	2.657306000
H	6.947219000	1.385809000	1.586193000
H	5.747067000	2.565063000	3.397325000
H	3.749133000	3.170644000	4.536701000
H	1.304123000	3.145551000	4.783792000
O	-0.590358000	-0.708377000	-1.140772000
H	1.767643000	-1.772138000	-1.367686000
N	3.059076000	-0.296950000	-0.049700000
C	0.684139000	0.676931000	0.977672000
H	1.137593000	0.213296000	0.126938000
C	0.613178000	-2.042662000	-0.766143000
O	0.144326000	-2.817767000	-0.065357000
C	5.745057000	-1.614744000	-2.315104000
C	5.518102000	-3.117725000	-2.134947000
C	5.191334000	-1.087938000	-3.641558000
C	7.247307000	-1.341729000	-2.275809000
H	5.892559000	-3.444848000	-1.161180000
H	4.468464000	-3.390005000	-2.223461000
H	6.078009000	-3.645086000	-2.910621000
H	5.357536000	-0.010199000	-3.721073000
H	5.728603000	-1.576917000	-4.457301000
H	4.129374000	-1.293992000	-3.758938000
H	7.689994000	-1.877797000	-3.117045000
H	7.483496000	-0.282167000	-2.403019000
H	7.717966000	-1.718533000	-1.364327000
H	-0.112368000	-0.068251000	-1.678140000

B1(B(C_6F_5)$_3$)

	Coordinates (Angstroms)		
	X	Y	Z
B	0.002446000	-0.002949000	0.002781000
C	1.542451000	-0.285786000	0.001187000
C	2.106690000	-1.300693000	0.770109000
C	2.426820000	0.464051000	-0.770015000

C	3.465080000	-1.556164000	0.786660000
C	3.787538000	0.221910000	-0.791315000
C	4.307077000	-0.791593000	-0.003572000
C	-1.015617000	-1.192980000	0.005205000
C	-2.176318000	-1.168141000	0.774444000
C	-0.814743000	-2.335540000	-0.765445000
C	-3.082683000	-2.211753000	0.790737000
C	-1.710670000	-3.388141000	-0.786451000
C	-2.848503000	-3.324654000	0.000496000
C	-0.521403000	1.473294000	0.000526000
C	0.064055000	2.463263000	0.785075000
C	-1.600053000	1.870101000	-0.785281000
C	-0.392972000	3.767708000	0.803485000
C	-2.069216000	3.170117000	-0.805159000
C	-1.463269000	4.121221000	-0.001333000
F	1.336204000	-2.058427000	1.554708000
F	3.968575000	-2.523020000	1.548435000
F	5.609822000	-1.030106000	-0.006316000
F	4.598212000	0.947903000	-1.555570000
F	1.972468000	1.444914000	-1.553943000
F	-2.441155000	-0.121513000	1.560564000
F	-4.171709000	-2.157253000	1.552122000
F	-3.712717000	-4.328167000	-0.002281000
F	-1.492476000	-4.454856000	-1.550294000
F	0.261907000	-2.438873000	-1.548975000
F	-2.209054000	0.991362000	-1.585863000
F	-3.090899000	3.514698000	-1.583722000
F	-1.907221000	5.369109000	-0.002437000
F	0.181402000	4.680799000	1.581129000
F	1.092436000	2.167590000	1.584663000

B1(TMP)

	Coordinates (Angstroms)		
	X	Y	Z
C	-1.276665000	-0.278416000	0.008869000
C	-1.109722000	1.077205000	-0.696212000
C	-0.003877000	1.949155000	-0.076061000
C	1.085806000	1.123304000	0.628876000
C	1.281419000	-0.274040000	0.031851000
N	0.003978000	-1.015643000	0.094478000
H	0.443861000	2.563153000	-0.862891000
H	-0.885819000	0.883259000	-1.749855000
H	2.042248000	1.653307000	0.594965000
H	0.839283000	1.003022000	1.688238000
H	-0.435356000	2.647609000	0.646646000
H	-2.065556000	1.610606000	-0.679460000
H	0.021197000	-1.733107000	-0.619886000
C	-2.253395000	-1.146367000	-0.785749000
H	-2.369557000	-2.126674000	-0.311884000
H	-3.238901000	-0.675333000	-0.838112000
H	-1.892955000	-1.298787000	-1.808576000
C	-1.848309000	-0.075014000	1.421358000
H	-2.827794000	0.413120000	1.377183000
H	-1.964305000	-1.042899000	1.917174000
H	-1.194873000	0.541224000	2.042803000
C	1.818739000	-0.176056000	-1.407222000
H	2.797590000	0.314211000	-1.425130000

H	1.939757000	-1.178406000	-1.832952000
H	1.149069000	0.385918000	-2.061572000
C	2.299139000	-1.049591000	0.874762000
H	2.428610000	-2.062767000	0.479510000
H	3.277241000	-0.556739000	0.872148000
H	1.949602000	-1.132295000	1.907466000

B1_CO

	Coordinates (Angstroms)		
	X	Y	Z
B	0.016678000	-0.009050000	0.618236000
C	1.592157000	0.115637000	0.232810000
C	2.545413000	-0.659133000	0.878312000
C	2.074088000	0.938702000	-0.775708000
C	3.892227000	-0.631979000	0.572387000
C	3.416854000	0.995297000	-1.113156000
C	4.330685000	0.209638000	-0.434869000
C	-0.680186000	-1.431291000	0.241872000
C	-1.851275000	-1.823058000	0.874677000
C	-0.218766000	-2.288867000	-0.747109000
C	-2.537579000	-2.982412000	0.570842000
C	-0.877128000	-3.461863000	-1.081128000
C	-2.039937000	-3.811684000	-0.418833000
C	-0.880486000	1.295746000	0.242726000
C	-0.695910000	2.499699000	0.906208000
C	-1.819100000	1.314330000	-0.779663000
C	-1.388767000	3.656332000	0.605154000
C	-2.535170000	2.452372000	-1.113144000
C	-2.321704000	3.628034000	-0.416415000
F	2.151007000	-1.500345000	1.852883000
F	4.759606000	-1.398821000	1.227903000
F	5.618735000	0.258304000	-0.751397000
F	3.833000000	1.797221000	-2.090716000
F	1.248910000	1.703664000	-1.497507000
F	-2.369624000	-1.033202000	1.833936000
F	-3.657198000	-3.304681000	1.212994000
F	-2.676934000	-4.932640000	-0.733926000
F	-0.402079000	-4.250696000	-2.042353000
F	0.877524000	-2.000376000	-1.455563000
F	-2.053069000	0.226555000	-1.521211000
F	-3.422722000	2.425170000	-2.104808000
F	-3.003690000	4.723146000	-0.728409000
F	-1.170001000	4.781654000	1.280224000
F	0.214231000	2.565393000	1.896397000
C	0.017191000	-0.021866000	2.254331000
O	0.013061000	-0.044953000	3.370391000

B1-2

	Coordinates (Angstroms)		
	X	Y	Z
B	-0.102679000	-0.000347000	0.290775000
C	-1.511845000	-0.219750000	-0.561322000
C	-2.448943000	0.799703000	-0.712162000
C	-1.908554000	-1.396439000	-1.200009000
C	-3.680301000	0.668682000	-1.326737000
C	-3.129220000	-1.576882000	-1.831798000

C	-4.035948000	-0.539864000	-1.889694000
C	0.793756000	1.292449000	-0.243335000
C	2.000665000	1.587524000	0.385714000
C	0.601504000	1.998881000	-1.425985000
C	2.912368000	2.527951000	-0.046210000
C	1.491084000	2.954374000	-1.901295000
C	2.651703000	3.228880000	-1.209607000
C	1.037736000	-1.191169000	-0.005727000
C	1.872320000	-1.838782000	0.895106000
C	1.371367000	-1.447911000	-1.336457000
C	2.889069000	-2.711304000	0.539082000
C	2.373469000	-2.307509000	-1.742642000
C	3.142429000	-2.955715000	-0.793767000
F	-2.196630000	2.038019000	-0.259165000
F	-4.517241000	1.704923000	-1.381526000
F	-5.211527000	-0.693942000	-2.488502000
F	-3.426557000	-2.750942000	-2.389281000
F	-1.122870000	-2.487430000	-1.240397000
F	2.325085000	0.944744000	1.525435000
F	4.026719000	2.766435000	0.645568000
F	3.508701000	4.142740000	-1.654888000
F	1.232935000	3.599630000	-3.039672000
F	-0.457491000	1.791976000	-2.219044000
F	0.703588000	-0.835406000	-2.326596000
F	2.608569000	-2.509276000	-3.039262000
F	4.112245000	-3.788652000	-1.159980000
F	3.625801000	-3.304056000	1.479081000
F	1.752948000	-1.647907000	2.230211000
C	-0.670414000	1.387121000	2.827954000
C	-2.112750000	1.450883000	3.390240000
C	-3.156037000	0.761218000	2.529817000
C	-2.838836000	-0.721545000	2.325913000
C	-1.359719000	-1.098767000	2.526405000
N	-0.437665000	0.047531000	1.991283000
H	-4.138110000	0.871738000	2.995235000
H	-2.141272000	1.043885000	4.401465000
H	-3.398956000	-1.327370000	3.043614000
H	-3.167341000	-1.041992000	1.335474000
H	-3.218453000	1.274160000	1.572136000
H	-2.370209000	2.506200000	3.491477000
H	0.476065000	-0.230470000	2.339136000
C	0.343924000	1.403509000	3.982552000
H	1.367229000	1.409609000	3.602629000
H	0.195264000	2.330426000	4.542038000
H	0.234451000	0.578309000	4.680681000
C	-0.408346000	2.679975000	2.049087000
H	-0.709904000	3.499933000	2.704201000
H	0.645908000	2.831385000	1.837112000
H	-0.975597000	2.763260000	1.131974000
C	-1.062133000	-1.388616000	4.011893000
H	-1.582572000	-2.314326000	4.265945000
H	0.004447000	-1.561712000	4.171806000
H	-1.407985000	-0.633033000	4.709103000
C	-1.108162000	-2.450368000	1.850167000
H	-0.177209000	-2.906783000	2.183733000
H	-1.919999000	-3.110372000	2.165380000
H	-1.118304000	-2.430716000	0.773053000

B3

	Coordinates (Angstroms)		
	X	Y	Z
B	0.516150000	-0.063774000	-0.165629000
H	-2.046063000	0.709313000	-1.386005000
C	-0.310603000	-0.958863000	0.943115000
C	-0.771232000	-2.213514000	0.557519000
C	-0.706489000	-0.573033000	2.214522000
C	-1.568632000	-3.021301000	1.346545000
C	-1.496538000	-1.355747000	3.045439000
C	-1.933780000	-2.590192000	2.609356000
C	1.886416000	-0.831471000	-0.716032000
C	2.481935000	-0.381975000	-1.885916000
C	2.555755000	-1.880718000	-0.102411000
C	3.630193000	-0.920471000	-2.437395000
C	3.708775000	-2.455504000	-0.616021000
C	4.250523000	-1.974785000	-1.793081000
C	1.044931000	1.400261000	0.403397000
C	0.673587000	2.634509000	-0.106482000
C	1.990109000	1.460373000	1.419622000
C	1.173124000	3.840727000	0.364791000
C	2.519993000	2.635016000	1.917871000
C	2.104710000	3.842708000	1.382954000
F	-0.455344000	-2.711089000	-0.650205000
F	-2.000877000	-4.202763000	0.897731000
F	-2.706079000	-3.348945000	3.385095000
F	-1.855154000	-0.912438000	4.253397000
F	-0.376476000	0.629863000	2.717878000
F	1.940047000	0.662691000	-2.556974000
F	4.142889000	-0.435790000	-3.570774000
F	5.358054000	-2.516634000	-2.297827000
F	4.303866000	-3.468592000	0.018648000
F	2.115844000	-2.404207000	1.052999000
F	2.417190000	0.321926000	1.993196000
F	3.418805000	2.621008000	2.905064000
F	2.598083000	4.990627000	1.846148000
F	0.763138000	4.998002000	-0.163452000
F	-0.217882000	2.745987000	-1.114439000
C	-3.624203000	0.399707000	-2.752710000
C	-4.436922000	-0.786102000	-2.205386000
C	-3.826886000	-1.321604000	-0.914799000
C	-3.937636000	-0.296286000	0.219531000
C	-3.716650000	1.165404000	-0.237561000
N	-2.990195000	1.131480000	-1.569639000
H	-4.319387000	-2.248975000	-0.616227000
H	-5.473921000	-0.485157000	-2.029704000
H	-4.929741000	-0.356075000	0.674022000
H	-3.216226000	-0.539610000	0.999861000
H	-2.776385000	-1.571359000	-1.090775000
H	-4.456444000	-1.560062000	-2.975635000
C	-4.482987000	1.368406000	-3.558588000
H	-3.880925000	2.197529000	-3.942800000
H	-4.899818000	0.832565000	-4.414438000
H	-5.313336000	1.774536000	-2.982287000
C	-2.466298000	-0.091538000	-3.623256000
H	-2.873686000	-0.530212000	-4.535997000

H	-1.811482000	0.737611000	-3.909389000
H	-1.868240000	-0.846031000	-3.109634000
C	-5.031696000	1.929888000	-0.366230000
H	-5.428103000	2.092455000	0.638573000
H	-4.877632000	2.907985000	-0.831043000
H	-5.786218000	1.381894000	-0.930464000
C	-2.794486000	1.913507000	0.722683000
H	-2.672349000	2.958455000	0.423516000
H	-3.231184000	1.891058000	1.723432000
H	-1.811728000	1.446015000	0.773721000
H	-2.800042000	2.094144000	-1.854314000
O	-0.455503000	0.084831000	-1.275557000
H	-0.037457000	0.482548000	-2.041560000

B4

	Coordinates (Angstroms)		
	X	Y	Z
B	-0.999086000	-0.089780000	0.279141000
C	-0.294804000	-1.412963000	-0.335072000
C	-0.394235000	-2.614270000	0.363087000
C	0.427898000	-1.488605000	-1.519378000
C	0.179733000	-3.799551000	-0.052099000
C	1.021531000	-2.658273000	-1.969844000
C	0.901249000	-3.819068000	-1.232992000
C	-2.599557000	-0.181606000	0.542474000
C	-3.230078000	0.776445000	1.331049000
C	-3.449909000	-1.100995000	-0.060423000
C	-4.596568000	0.821307000	1.537979000
C	-4.823879000	-1.085569000	0.117426000
C	-5.400616000	-0.121609000	0.923667000
C	-0.651570000	1.332114000	-0.415360000
C	0.073305000	2.386406000	0.115398000
C	-1.200998000	1.572251000	-1.669844000
C	0.277086000	3.582127000	-0.556053000
C	-1.020905000	2.746324000	-2.374262000
C	-0.267297000	3.762467000	-1.811980000
F	-1.089291000	-2.656875000	1.512935000
F	0.054534000	-4.910062000	0.670938000
F	1.491874000	-4.936090000	-1.639131000
F	1.736390000	-2.658845000	-3.094057000
F	0.609063000	-0.418980000	-2.300424000
F	-2.497114000	1.720149000	1.945124000
F	-5.139592000	1.757134000	2.313563000
F	-6.715399000	-0.099707000	1.104636000
F	-5.593280000	-1.989621000	-0.485709000
F	-2.975962000	-2.048834000	-0.876152000
F	-1.938291000	0.618102000	-2.258022000
F	-1.560977000	2.908117000	-3.580896000
F	-0.077104000	4.900495000	-2.470837000
F	0.999089000	4.553328000	0.002386000
F	0.631866000	2.306313000	1.335179000
C	4.305711000	-1.006366000	0.446192000
C	4.099520000	-0.380905000	-0.951684000
C	3.133278000	0.797875000	-0.892573000
C	3.720159000	1.931068000	-0.050578000
C	4.416022000	1.417476000	1.241183000
N	4.002199000	0.021209000	1.462245000

H	2.907153000	1.159544000	-1.899106000
H	5.058604000	-0.031701000	-1.351281000
H	4.448519000	2.484051000	-0.652210000
H	2.935567000	2.644044000	0.211629000
H	2.181078000	0.467646000	-0.465473000
H	3.734177000	-1.149819000	-1.639310000
C	5.711323000	-1.613204000	0.573512000
H	5.842149000	-2.068224000	1.561559000
H	5.851328000	-2.398388000	-0.177067000
H	6.497932000	-0.872074000	0.431987000
C	3.292608000	-2.134292000	0.669212000
H	3.492542000	-2.979354000	0.003842000
H	3.337655000	-2.494249000	1.702492000
H	2.279582000	-1.780069000	0.478333000
C	5.939035000	1.618394000	1.132316000
H	6.173544000	2.687947000	1.127953000
H	6.453379000	1.160341000	1.983674000
H	6.343754000	1.192164000	0.212262000
C	3.923039000	2.206497000	2.456990000
H	4.425761000	1.869846000	3.370547000
H	4.129651000	3.273037000	2.330191000
H	2.846785000	2.080163000	2.591519000
H	4.334074000	-0.292711000	2.368532000
H	1.671207000	0.012333000	1.598439000
O	-0.404289000	-0.058935000	1.863188000
C	0.904226000	-0.173220000	2.358715000
O	1.065242000	-0.434118000	3.499406000
H	-1.046103000	-0.282807000	2.561145000

B7

	Coordinates (Angstroms)		
	X	Y	Z
H	0.202363000	0.074138000	1.009536000
B	-0.518423000	-0.052449000	0.041240000
H	1.791966000	0.554886000	1.351947000
C	0.404676000	-0.784009000	-1.092632000
C	0.958495000	-2.022452000	-0.790508000
C	0.786796000	-0.280927000	-2.327364000
C	1.818687000	-2.717458000	-1.619185000
C	1.645309000	-0.941409000	-3.195378000
C	2.165402000	-2.171204000	-2.842171000
C	-1.778921000	-0.961617000	0.555267000
C	-2.302906000	-0.727535000	1.819989000
C	-2.426109000	-1.956171000	-0.163218000
C	-3.373000000	-1.424003000	2.354109000
C	-3.501414000	-2.679052000	0.330949000
C	-3.979785000	-2.411928000	1.599858000
C	-1.089273000	1.426193000	-0.345362000
C	-0.729669000	2.577965000	0.333206000
C	-2.039609000	1.612360000	-1.339720000
C	-1.238336000	3.834210000	0.040047000
C	-2.581318000	2.842009000	-1.665127000
C	-2.173972000	3.966865000	-0.967341000
F	0.683863000	-2.602689000	0.395258000
F	2.335953000	-3.889305000	-1.241690000
F	2.997573000	-2.815888000	-3.659362000
F	1.979541000	-0.393050000	-4.366519000

F	0.362960000	0.924649000	-2.748834000
F	-1.767390000	0.228280000	2.603049000
F	-3.825868000	-1.152545000	3.581874000
F	-5.013369000	-3.096816000	2.090497000
F	-4.080942000	-3.630190000	-0.407436000
F	-2.027226000	-2.277706000	-1.405494000
F	-2.462942000	0.558165000	-2.055884000
F	-3.485146000	2.960027000	-2.641040000
F	-2.679372000	5.163480000	-1.265964000
F	-0.835306000	4.910942000	0.721712000
F	0.163195000	2.534988000	1.345360000
C	3.111699000	0.103102000	2.915382000
C	4.197500000	-0.852423000	2.398155000
C	3.936936000	-1.268095000	0.955928000
C	4.007763000	-0.081473000	-0.017366000
C	3.574486000	1.270465000	0.590721000
N	2.639069000	0.983007000	1.752302000
H	4.652967000	-2.032350000	0.649076000
H	5.183166000	-0.388280000	2.486023000
H	5.028865000	0.042802000	-0.385379000
H	3.382488000	-0.296433000	-0.886679000
H	2.951123000	-1.735644000	0.899033000
H	4.201846000	-1.727024000	3.052705000
C	3.622154000	1.001709000	4.035033000
H	2.825829000	1.654748000	4.404661000
H	3.953918000	0.371811000	4.863449000
H	4.466198000	1.616457000	3.720518000
C	1.874922000	-0.662628000	3.383707000
H	2.127858000	-1.235180000	4.277953000
H	1.062122000	0.024201000	3.635098000
H	1.514817000	-1.355478000	2.619046000
C	4.758453000	2.103830000	1.076010000
H	5.318179000	2.436606000	0.199287000
H	4.417391000	2.992612000	1.614700000
H	5.442711000	1.543496000	1.712266000
C	2.761573000	2.086393000	-0.410978000
H	2.499261000	3.067200000	-0.005698000
H	3.358018000	2.234817000	-1.313572000
H	1.845726000	1.566020000	-0.694286000
H	2.318866000	1.874431000	2.136167000

B8

	Coordinates (Angstroms)		
	X	Y	Z
B	0.980626000	0.099997000	-0.078324000
C	0.420847000	1.404247000	0.733299000
C	-0.694210000	1.273247000	1.555269000
C	0.870732000	2.707494000	0.578120000
C	-1.318669000	2.327008000	2.191541000
C	0.281612000	3.795608000	1.207582000
C	-0.820874000	3.606774000	2.016895000
C	1.213705000	-1.250273000	0.816024000
C	1.329119000	-2.487677000	0.190910000
C	1.429349000	-1.269795000	2.188906000
C	1.601795000	-3.667135000	0.861222000
C	1.710945000	-2.427399000	2.896034000
C	1.794882000	-3.635714000	2.229803000

C	2.343018000	0.335398000	-0.943931000
C	2.506048000	0.188577000	-2.313067000
C	3.509127000	0.624194000	-0.244436000
C	3.733227000	0.329470000	-2.947003000
C	4.747831000	0.772841000	-0.835705000
C	4.861043000	0.622565000	-2.207131000
F	-1.246867000	0.060180000	1.735704000
F	-2.396252000	2.128029000	2.949936000
F	-1.402403000	4.640321000	2.615805000
F	0.763734000	5.024219000	1.022083000
F	1.903774000	2.998135000	-0.224221000
F	1.175973000	-2.582572000	-1.140941000
F	1.687821000	-4.822190000	0.201966000
F	2.060312000	-4.754132000	2.896974000
F	1.903170000	-2.384796000	4.214454000
F	1.384376000	-0.144150000	2.912196000
F	3.451611000	0.789229000	1.087621000
F	5.826582000	1.057461000	-0.105548000
F	6.042451000	0.759283000	-2.803447000
F	3.828920000	0.181771000	-4.269567000
F	1.472360000	-0.097856000	-3.118940000
C	-5.052571000	0.643196000	-0.911092000
C	-6.258600000	0.036947000	-0.157958000
C	-6.781374000	-1.202747000	-0.873773000
C	-5.733964000	-2.321933000	-0.862753000
C	-4.279790000	-1.793026000	-1.032817000
N	-4.269626000	-0.420594000	-1.611308000
H	-7.705516000	-1.555198000	-0.408932000
H	-5.971243000	-0.226757000	0.865614000
H	-5.798192000	-2.872706000	0.081064000
H	-5.960395000	-3.038514000	-1.657790000
H	-7.035764000	-0.942584000	-1.907708000
H	-7.037962000	0.800111000	-0.074980000
C	-4.163074000	1.433612000	0.053136000
H	-3.448013000	2.051552000	-0.497503000
H	-4.780327000	2.104870000	0.657870000
H	-3.612740000	0.783822000	0.733666000
C	-5.544178000	1.596819000	-2.003560000
H	-6.013588000	2.476407000	-1.556125000
H	-4.705202000	1.933524000	-2.620787000
H	-6.278209000	1.113484000	-2.656281000
C	-3.543981000	-1.819739000	0.308157000
H	-3.388148000	-2.857376000	0.614941000
H	-2.567361000	-1.338824000	0.228274000
H	-4.094999000	-1.320814000	1.106442000
C	-3.515920000	-2.678685000	-2.020269000
H	-2.468177000	-2.375027000	-2.101341000
H	-3.549066000	-3.720949000	-1.691997000
H	-3.964345000	-2.625074000	-3.017839000
O	-0.193073000	-0.267750000	-1.083893000
H	-0.767124000	-0.985132000	-0.780944000
C	-1.026323000	0.793586000	-1.766163000
H	-1.521839000	1.338023000	-0.962300000
H	-0.274276000	1.401966000	-2.264799000
O	-1.864031000	0.206485000	-2.630040000
H	-2.748376000	0.006852000	-2.181744000
H	-4.686608000	-0.518527000	-2.534681000

TSB3-4

	Coordinates (Angstroms)		
	X	Y	Z
B	-0.887185000	-0.079668000	0.376089000
H	2.262352000	0.432500000	1.680360000
C	0.104879000	-1.086086000	-0.439652000
C	0.257226000	-2.385170000	0.038793000
C	0.796371000	-0.820768000	-1.612412000
C	1.011892000	-3.356525000	-0.589812000
C	1.563664000	-1.767766000	-2.276215000
C	1.675235000	-3.044259000	-1.762819000
C	-2.412544000	-0.681677000	0.539247000
C	-3.263279000	-0.173497000	1.511718000
C	-2.998991000	-1.619881000	-0.301429000
C	-4.580480000	-0.557066000	1.678854000
C	-4.316778000	-2.032208000	-0.176155000
C	-5.113430000	-1.501385000	0.820383000
C	-1.052624000	1.437434000	-0.213821000
C	-0.745615000	2.612805000	0.453696000
C	-1.626334000	1.617304000	-1.466670000
C	-0.936175000	3.872935000	-0.092742000
C	-1.840961000	2.852211000	-2.047573000
C	-1.487295000	3.995900000	-1.352539000
F	-0.362282000	-2.760281000	1.168475000
F	1.128829000	-4.576798000	-0.065324000
F	2.430467000	-3.952715000	-2.372308000
F	2.223600000	-1.440241000	-3.388501000
F	0.774813000	0.393888000	-2.180990000
F	-2.801032000	0.767211000	2.369715000
F	-5.332353000	-0.028964000	2.643383000
F	-6.377551000	-1.891533000	0.951689000
F	-4.823114000	-2.936950000	-1.013842000
F	-2.314681000	-2.177930000	-1.308718000
F	-1.987506000	0.543875000	-2.187497000
F	-2.380956000	2.952371000	-3.262567000
F	-1.679537000	5.197403000	-1.890486000
F	-0.587941000	4.963988000	0.591658000
F	-0.215951000	2.598280000	1.690394000
C	4.357404000	-0.605052000	1.539911000
C	5.649289000	-0.538836000	0.722029000
C	5.411723000	-0.205943000	-0.759223000
C	4.178034000	0.684790000	-0.985000000
C	3.811064000	1.560459000	0.213394000
N	3.588060000	0.685838000	1.418521000
H	6.306124000	0.279823000	-1.157017000
H	6.308409000	0.203782000	1.179411000
H	4.331018000	1.339130000	-1.846688000
H	3.311364000	0.066645000	-1.225926000
H	5.281274000	-1.128421000	-1.331496000
H	6.160141000	-1.501032000	0.816170000
H	3.822590000	1.254168000	2.230240000
C	4.674232000	-0.800513000	3.020774000
H	3.758262000	-0.789334000	3.619274000
H	5.167842000	-1.763222000	3.173968000
H	5.339715000	-0.012281000	3.386940000
C	3.475154000	-1.757748000	1.053694000

H	4.044837000	-2.690515000	1.056874000
H	2.605851000	-1.895449000	1.701538000
H	3.120392000	-1.581337000	0.039804000
C	4.900203000	2.600741000	0.500103000
H	4.989255000	3.279411000	-0.352364000
H	4.632873000	3.199697000	1.376994000
H	5.879176000	2.153931000	0.677950000
C	2.496561000	2.290056000	-0.055378000
H	2.205581000	2.901089000	0.803948000
H	2.598538000	2.947208000	-0.922732000
H	1.696366000	1.579601000	-0.262551000
O	-0.287934000	-0.049764000	1.821448000
H	-0.923781000	0.150829000	2.525138000
C	1.144326000	0.294452000	2.404204000
O	1.084380000	0.424588000	3.568825000

TSB1-7

	Coordinates (Angstroms)		
	X	Y	Z
C	-2.811437000	1.539497000	-2.264313000
N	-2.738108000	0.430656000	-1.281565000
C	-3.541266000	-0.794975000	-1.500661000
C	-4.989112000	-0.442620000	-1.892967000
C	-5.059401000	0.593938000	-3.013154000
C	-4.275279000	1.848487000	-2.630842000
C	-2.893351000	-1.703049000	-2.553388000
C	-3.542197000	-1.543676000	-0.166297000
C	-1.988263000	1.232952000	-3.523106000
C	-2.179554000	2.755499000	-1.581491000
F	-0.062601000	-2.563512000	-0.686425000
C	1.259987000	-2.377527000	-0.744487000
C	1.829047000	-1.241798000	-0.175352000
C	3.213661000	-1.149252000	-0.273161000
C	3.986287000	-2.108543000	-0.905629000
C	3.372890000	-3.208580000	-1.476610000
C	1.996899000	-3.344968000	-1.398730000
B	0.957568000	-0.123478000	0.530279000
C	1.450562000	1.379970000	0.443933000
C	1.801921000	1.974214000	-0.763397000
C	2.215455000	3.289737000	-0.863616000
C	2.323256000	4.053964000	0.285283000
C	2.007980000	3.496623000	1.512046000
C	1.579830000	2.182692000	1.571280000
F	1.402180000	-4.402973000	-1.944356000
F	4.099216000	-4.132084000	-2.091349000
F	5.309897000	-1.981722000	-0.966731000
F	3.869190000	-0.118105000	0.269374000
C	-0.175685000	-0.515050000	1.558798000
C	-1.241330000	0.343758000	1.818734000
C	-2.293629000	0.016732000	2.649905000
C	-2.298009000	-1.210782000	3.287105000
C	-1.246181000	-2.086531000	3.087520000
C	-0.212703000	-1.729760000	2.238093000
F	-1.302371000	1.542768000	1.232121000
F	0.777384000	-2.616799000	2.107522000
F	-1.235085000	-3.261878000	3.711623000
F	-3.306095000	-1.546796000	4.079088000

F	-3.316636000	0.854291000	2.810225000
H	-5.494767000	-0.036666000	-1.007191000
H	-5.521848000	-1.356845000	-2.175987000
H	-4.667594000	0.171237000	-3.944644000
H	-6.103699000	0.854548000	-3.208908000
H	-4.756065000	2.313739000	-1.760380000
H	-4.299388000	2.583851000	-3.442174000
H	-0.844011000	0.100220000	-1.026342000
H	-1.848897000	2.155807000	-4.093645000
H	-1.001668000	0.847463000	-3.255054000
H	-2.468845000	0.513096000	-4.185375000
H	-1.123212000	2.570955000	-1.363538000
H	-2.248132000	3.631494000	-2.232310000
H	-2.688485000	2.989926000	-0.641363000
H	-1.827190000	-1.824983000	-2.351099000
H	-3.362396000	-2.690584000	-2.518266000
H	-3.008802000	-1.325415000	-3.569183000
H	-4.001216000	-0.936795000	0.620883000
H	-4.113774000	-2.471928000	-0.249296000
H	-2.520893000	-1.793134000	0.135782000
H	-3.037495000	0.818711000	-0.391637000
H	-0.072326000	0.013037000	-1.053356000
F	1.297716000	1.694572000	2.784591000
F	2.121845000	4.226030000	2.619862000
F	2.728926000	5.314969000	0.210987000
F	2.515033000	3.821524000	-2.046260000
F	1.728884000	1.277423000	-1.903642000

TSB7

	Coordinates (Angstroms)		
	X	Y	Z
H	0.256975000	-0.077335000	-1.290890000
B	1.056675000	-0.102993000	0.067660000
H	-2.172634000	-0.535206000	-1.798367000
C	-0.091195000	-0.796350000	0.918615000
C	-0.410581000	-2.142326000	0.734433000
C	-0.865807000	-0.135165000	1.870128000
C	-1.397008000	-2.796214000	1.444915000
C	-1.854718000	-0.762390000	2.608421000
C	-2.124122000	-2.100128000	2.393287000
C	2.353501000	-0.941623000	-0.357390000
C	3.050732000	-0.647412000	-1.522108000
C	2.934128000	-1.908563000	0.455336000
C	4.220564000	-1.283502000	-1.894312000
C	4.110901000	-2.558399000	0.125615000
C	4.754742000	-2.248505000	-1.059004000
C	1.421410000	1.443166000	0.314758000
C	1.280222000	2.509172000	-0.555204000
C	2.042615000	1.738363000	1.522516000
C	1.688908000	3.796776000	-0.241550000
C	2.467990000	3.004400000	1.872787000
C	2.285591000	4.046259000	0.978859000
F	0.242532000	-2.871668000	-0.174511000
F	-1.684155000	-4.072275000	1.195628000
F	-3.104496000	-2.699149000	3.055521000
F	-2.579268000	-0.074144000	3.490066000
F	-0.701911000	1.168474000	2.115769000

F	2.582197000	0.297383000	-2.355683000
F	4.834543000	-0.972855000	-3.033842000
F	5.879995000	-2.869756000	-1.390441000
F	4.628876000	-3.476800000	0.938238000
F	2.375326000	-2.245328000	1.622632000
F	2.234184000	0.753610000	2.414315000
F	3.045086000	3.230898000	3.051277000
F	2.685156000	5.274411000	1.292352000
F	1.515536000	4.789615000	-1.112904000
F	0.733107000	2.343313000	-1.765856000
C	-4.683231000	-0.750642000	-1.400271000
C	-5.941042000	-0.082600000	-0.831977000
C	-5.653521000	0.767889000	0.416954000
C	-4.227499000	1.344273000	0.434717000
C	-3.625526000	1.554956000	-0.956382000
N	-3.632042000	0.266122000	-1.716788000
H	-6.386652000	1.577397000	0.467949000
H	-6.383003000	0.536662000	-1.618336000
H	-4.212660000	2.303570000	0.959169000
H	-3.563020000	0.685377000	0.998670000
H	-5.796836000	0.167944000	1.319896000
H	-6.678058000	-0.856923000	-0.599261000
H	-3.731345000	0.509676000	-2.697888000
C	-5.013868000	-1.470289000	-2.706654000
H	-4.110566000	-1.908449000	-3.142307000
H	-5.731283000	-2.275611000	-2.528621000
H	-5.449943000	-0.778678000	-3.435060000
C	-4.131008000	-1.775039000	-0.399747000
H	-4.908185000	-2.495447000	-0.128900000
H	-3.288351000	-2.321778000	-0.827646000
H	-3.786214000	-1.293124000	0.514267000
C	-4.395293000	2.642754000	-1.719670000
H	-4.321099000	3.598000000	-1.191678000
H	-3.964892000	2.779373000	-2.717567000
H	-5.453778000	2.404101000	-1.838349000
C	-2.168060000	1.996061000	-0.833339000
H	-1.726877000	2.130495000	-1.822344000
H	-2.085764000	2.938384000	-0.283578000
H	-1.582469000	1.239894000	-0.307197000
C	-0.249224000	-0.636268000	-2.244375000
H	0.492616000	-1.399945000	-2.499492000
O	-1.399435000	-1.196641000	-1.876718000
O	-0.421126000	0.318643000	-3.214396000
H	0.431473000	0.714232000	-3.423540000

TSB8

	Coordinates (Angstroms)		
	X	Y	Z
B	1.051515000	-0.034782000	-0.171232000
H	-2.823643000	-0.642939000	-1.549061000
C	-0.042431000	-0.233833000	1.033866000
C	-0.638319000	-1.467772000	1.277175000
C	-0.609027000	0.825283000	1.729534000
C	-1.705756000	-1.648119000	2.134323000
C	-1.690179000	0.689785000	2.587958000
C	-2.247307000	-0.555885000	2.786974000
C	2.177034000	-1.212065000	-0.353438000

C	2.847227000	-1.353523000	-1.564886000
C	2.618789000	-2.060726000	0.654742000
C	3.839399000	-2.294488000	-1.787066000
C	3.610926000	-3.009912000	0.473630000
C	4.222475000	-3.134280000	-0.759330000
C	1.861640000	1.394677000	-0.092416000
C	1.719678000	2.478919000	-0.943112000
C	2.812959000	1.574242000	0.904580000
C	2.461570000	3.644256000	-0.845580000
C	3.579874000	2.717144000	1.042573000
C	3.405433000	3.764027000	0.155415000
F	-0.211607000	-2.566444000	0.641293000
F	-2.258126000	-2.852934000	2.293446000
F	-3.317819000	-0.700118000	3.565546000
F	-2.226131000	1.760492000	3.179565000
F	-0.178676000	2.087107000	1.555563000
F	2.568658000	-0.548478000	-2.602552000
F	4.434574000	-2.387489000	-2.978437000
F	5.176125000	-4.042680000	-0.950395000
F	3.984417000	-3.802409000	1.480252000
F	2.102776000	-1.995684000	1.889350000
F	3.009233000	0.612901000	1.819223000
F	4.476665000	2.820385000	2.023272000
F	4.129346000	4.874115000	0.271654000
F	2.265378000	4.648073000	-1.700815000
F	0.791190000	2.471094000	-1.930930000
C	-4.990351000	-1.083549000	-1.205374000
C	-6.292166000	-0.341905000	-0.898811000
C	-6.117336000	0.766074000	0.153457000
C	-4.705174000	1.375493000	0.154937000
C	-4.008123000	1.326704000	-1.206062000
N	-3.952235000	-0.101207000	-1.704390000
H	-6.861632000	1.545165000	-0.029408000
H	-6.678745000	0.075552000	-1.833407000
H	-4.745665000	2.421699000	0.468528000
H	-4.069653000	0.867642000	0.885276000
H	-6.324225000	0.369411000	1.150442000
H	-7.030831000	-1.072785000	-0.559227000
H	-4.062330000	-0.052217000	-2.715162000
C	-5.195791000	-2.096003000	-2.330383000
H	-4.252307000	-2.591181000	-2.577878000
H	-5.910976000	-2.860324000	-2.018705000
H	-5.585922000	-1.611102000	-3.231304000
C	-4.479894000	-1.822115000	0.037244000
H	-5.227107000	-2.561187000	0.337409000
H	-3.542665000	-2.337946000	-0.171898000
H	-4.319643000	-1.156086000	0.884671000
C	-4.739275000	2.198943000	-2.231107000
H	-4.709142000	3.243389000	-1.910549000
H	-4.247789000	2.133831000	-3.207487000
H	-5.785047000	1.914297000	-2.356404000
C	-2.570896000	1.822903000	-1.069105000
H	-2.075593000	1.832873000	-2.045895000
H	-2.556724000	2.841373000	-0.674591000
H	-2.012872000	1.179653000	-0.388717000
C	-0.741885000	-1.164383000	-2.048368000
H	-0.871471000	-0.781283000	-3.069779000

H	-0.052038000	-2.012510000	-2.019766000
O	-1.817852000	-1.310511000	-1.331548000
O	0.227119000	-0.027145000	-1.495294000
H	-0.132639000	0.837555000	-1.717986000

TSB9

	Coordinates (Angstroms)		
	X	Y	Z
H	0.311994000	-0.039795000	-1.270650000
B	1.024458000	-0.091276000	-0.070643000
H	-2.452495000	-0.319791000	-1.785027000
C	-0.095343000	-0.792551000	0.831073000
C	-0.428446000	-2.127740000	0.613486000
C	-0.855201000	-0.154996000	1.806211000
C	-1.405920000	-2.798684000	1.322569000
C	-1.840812000	-0.794856000	2.539526000
C	-2.119038000	-2.125772000	2.296551000
C	2.348813000	-0.935355000	-0.437234000
C	3.049384000	-0.677964000	-1.608039000
C	2.927105000	-1.875898000	0.405028000
C	4.226347000	-1.319078000	-1.951025000
C	4.107264000	-2.534224000	0.102315000
C	4.759467000	-2.256883000	-1.085181000
C	1.411375000	1.451698000	0.213358000
C	1.270337000	2.517896000	-0.658617000
C	2.034827000	1.751112000	1.418123000
C	1.678509000	3.806840000	-0.348667000
C	2.460108000	3.018642000	1.765385000
C	2.276656000	4.059121000	0.870349000
F	0.213588000	-2.836183000	-0.322224000
F	-1.702622000	-4.067892000	1.041844000
F	-3.097005000	-2.738032000	2.955888000
F	-2.553531000	-0.123590000	3.446380000
F	-0.683469000	1.145081000	2.082685000
F	2.582060000	0.234838000	-2.475043000
F	4.848735000	-1.040183000	-3.096553000
F	5.891344000	-2.884328000	-1.390794000
F	4.620303000	-3.431134000	0.944666000
F	2.357246000	-2.189669000	1.575897000
F	2.231570000	0.771800000	2.314885000
F	3.039213000	3.249006000	2.943642000
F	2.676217000	5.289311000	1.181637000
F	1.503544000	4.799681000	-1.221866000
F	0.723627000	2.353781000	-1.872582000
C	-4.661665000	-0.756290000	-1.494300000
C	-5.928706000	-0.095183000	-0.947806000
C	-5.676347000	0.728571000	0.325520000
C	-4.260067000	1.325767000	0.385627000
C	-3.632853000	1.575094000	-0.986847000
N	-3.612603000	0.292183000	-1.783264000
H	-6.422391000	1.525327000	0.380433000
H	-6.353243000	0.536518000	-1.733399000
H	-4.272969000	2.277898000	0.922026000
H	-3.595185000	0.671686000	0.954453000
H	-5.827158000	0.104345000	1.210027000
H	-6.666580000	-0.877692000	-0.751080000
H	-3.729756000	0.563008000	-2.757266000

C	-4.951447000	-1.458410000	-2.818838000
H	-4.035252000	-1.885459000	-3.237291000
H	-5.666223000	-2.270286000	-2.664866000
H	-5.377841000	-0.761247000	-3.547455000
C	-4.112402000	-1.778123000	-0.493250000
H	-4.893888000	-2.500652000	-0.244842000
H	-3.260281000	-2.315874000	-0.910879000
H	-3.787347000	-1.303022000	0.431291000
C	-4.403724000	2.654247000	-1.755683000
H	-4.348543000	3.601039000	-1.212131000
H	-3.956961000	2.809951000	-2.743195000
H	-5.457180000	2.406604000	-1.894074000
C	-2.185550000	2.032921000	-0.828276000
H	-1.734024000	2.227893000	-1.803370000
H	-2.140052000	2.954783000	-0.242886000
H	-1.590053000	1.275288000	-0.318470000
C	-0.383959000	-0.476226000	-2.251827000
H	-0.439787000	0.482399000	-2.779199000
H	0.345880000	-1.169784000	-2.678392000
O	-1.499300000	-1.019561000	-1.855130000

C1

	Coordinates (Angstroms)		
	X	Y	Z
B	0.715276000	-0.183547000	0.744019000
C	2.184664000	-0.185460000	0.141249000
C	2.988725000	0.955116000	0.185711000
C	2.773989000	-1.301380000	-0.452762000
C	4.270542000	1.000545000	-0.331581000
C	4.046594000	-1.284095000	-0.996740000
C	4.799922000	-0.126160000	-0.934430000
C	-0.073211000	1.171833000	0.569466000
C	-0.199861000	1.786998000	-0.667918000
C	-0.540173000	1.887240000	1.661987000
C	-0.761905000	3.038634000	-0.827688000
C	-1.086440000	3.154502000	1.544067000
C	-1.200138000	3.729967000	0.290501000
F	2.556084000	2.074251000	0.776410000
F	4.998331000	2.112018000	-0.248499000
F	6.024728000	-0.098641000	-1.440293000
F	4.553024000	-2.373137000	-1.570717000
F	2.120116000	-2.459716000	-0.539801000
F	0.223009000	1.157064000	-1.770496000
F	-0.905619000	3.574108000	-2.038264000
F	-1.745383000	4.932713000	0.157034000
F	-1.514226000	3.815348000	2.617834000
F	-0.445627000	1.373260000	2.893759000
C	0.222053000	-1.380709000	1.599775000
C	-1.108255000	-1.857846000	1.706860000
C	1.218324000	-2.019241000	2.358493000
C	-1.361910000	-2.933484000	2.555945000
C	0.941197000	-3.070755000	3.219749000
H	2.242737000	-1.669226000	2.285891000
C	-0.361151000	-3.534714000	3.311171000
H	-2.375635000	-3.312698000	2.633189000
H	1.735594000	-3.526590000	3.799602000
H	-0.605638000	-4.365023000	3.964871000

C	-2.326079000	-1.232805000	1.038783000
H	-2.404971000	-0.236476000	1.472652000
H	-3.199506000	-1.788647000	1.411199000
C	-3.522292000	-0.211996000	-0.842363000
C	-2.208653000	-2.338383000	-1.211848000
C	-3.228234000	0.295971000	-2.276864000
C	-2.669410000	-2.102240000	-2.671556000
C	-2.397731000	-0.676281000	-3.114581000
H	-4.187380000	0.497545000	-2.766103000
H	-3.732422000	-2.327507000	-2.789862000
H	-2.621731000	-0.551010000	-4.177689000
N	-2.370421000	-1.072943000	-0.416037000
H	-2.137983000	-2.814494000	-3.308094000
H	-1.334264000	-0.458244000	-2.988857000
H	-2.703081000	1.253768000	-2.211092000
C	-2.963715000	-3.552491000	-0.642453000
H	-2.873334000	-4.386718000	-1.345469000
H	-2.541101000	-3.883375000	0.308828000
H	-4.026282000	-3.353987000	-0.497116000
C	-0.718747000	-2.700380000	-1.276977000
H	-0.570536000	-3.532699000	-1.971713000
H	-0.130192000	-1.849832000	-1.629128000
H	-0.322477000	-3.009111000	-0.313615000
C	-4.902923000	-0.906812000	-0.763102000
H	-5.685542000	-0.161796000	-0.936403000
H	-5.042393000	-1.697148000	-1.498397000
H	-5.070232000	-1.331031000	0.231195000
C	-3.658322000	1.038415000	0.037755000
H	-4.111007000	0.833318000	1.010830000
H	-2.697722000	1.520487000	0.188935000
H	-4.309440000	1.749475000	-0.478091000

C1_CO

	Coordinates (Angstroms)		
	X	Y	Z
B	0.295521000	-0.197417000	0.322558000
C	1.721446000	-0.926419000	-0.061374000
C	2.944834000	-0.328854000	0.231715000
C	1.837357000	-2.206274000	-0.589221000
C	4.174705000	-0.911049000	-0.028617000
C	3.045192000	-2.825559000	-0.868718000
C	4.228766000	-2.169561000	-0.594813000
C	0.458670000	1.441458000	0.127123000
C	0.871208000	1.957733000	-1.096730000
C	0.252971000	2.404721000	1.106728000
C	1.043101000	3.304930000	-1.356314000
C	0.409552000	3.765687000	0.889670000
C	0.806519000	4.223510000	-0.351122000
F	3.002024000	0.870842000	0.835796000
F	5.306332000	-0.270998000	0.275825000
F	5.400652000	-2.747698000	-0.851289000
F	3.072082000	-4.055483000	-1.388489000
F	0.747538000	-2.949939000	-0.832334000
F	1.111154000	1.130581000	-2.128243000
F	1.422442000	3.723644000	-2.565456000
F	0.962191000	5.526486000	-0.573941000
F	0.189204000	4.636800000	1.877311000

F	-0.115303000	2.070635000	2.352873000
C	-0.189275000	-0.642712000	1.791019000
C	-1.536183000	-0.463810000	2.118526000
C	0.652397000	-1.183557000	2.762793000
C	-2.007038000	-0.757195000	3.394695000
C	0.184882000	-1.495879000	4.035638000
H	1.699794000	-1.353091000	2.533486000
C	-1.147064000	-1.270082000	4.358773000
H	-3.051076000	-0.584488000	3.638484000
H	0.863199000	-1.905427000	4.776802000
H	-1.518562000	-1.496225000	5.352264000
C	-2.458392000	0.170363000	1.109408000
H	-2.142650000	1.197798000	0.945668000
H	-3.467283000	0.214860000	1.503198000
C	-3.216662000	0.461365000	-1.268919000
C	-3.194326000	-1.925774000	-0.150460000
C	-3.370010000	-0.203108000	-2.666211000
C	-4.060025000	-2.227560000	-1.390362000
C	-3.486907000	-1.724382000	-2.694387000
H	-4.272174000	0.239720000	-3.096169000
H	-5.064276000	-1.819841000	-1.251201000
H	-4.131068000	-2.031233000	-3.521759000
N	-2.495911000	-0.491289000	-0.246373000
H	-4.174000000	-3.314520000	-1.414663000
H	-2.507761000	-2.172170000	-2.866532000
H	-2.540339000	0.107788000	-3.300978000
C	-4.122014000	-2.044959000	1.065484000
H	-4.650946000	-2.993373000	0.946526000
H	-3.583133000	-2.095082000	2.008767000
H	-4.877695000	-1.261357000	1.117720000
C	-2.116613000	-3.005181000	-0.023139000
H	-2.616471000	-3.936011000	0.253046000
H	-1.591781000	-3.175385000	-0.960406000
H	-1.390259000	-2.777217000	0.757258000
C	-4.588550000	0.878035000	-0.714071000
H	-5.070744000	1.497476000	-1.472338000
H	-5.254343000	0.042528000	-0.508792000
H	-4.500422000	1.486520000	0.186517000
C	-2.417502000	1.751621000	-1.493133000
H	-2.261839000	2.343456000	-0.590655000
H	-1.460309000	1.564949000	-1.974442000
H	-3.003621000	2.363710000	-2.182518000
C	-0.894293000	-0.623610000	-0.762113000
O	-0.785475000	-0.959313000	-1.884645000

C3

	Coordinates (Angstroms)		
	X	Y	Z
B	0.494988000	-0.222137000	0.120673000
C	2.033019000	-0.778423000	-0.163608000
C	3.205310000	-0.077620000	0.083517000
C	2.229098000	-2.057354000	-0.676287000
C	4.472526000	-0.572753000	-0.186677000
C	3.475096000	-2.591272000	-0.960810000
C	4.609808000	-1.838851000	-0.720357000
C	0.498512000	1.446798000	0.158486000
C	0.727568000	2.143613000	-1.020532000

C	0.251764000	2.258409000	1.254994000
C	0.686367000	3.519820000	-1.140183000
C	0.194177000	3.643525000	1.186939000
C	0.410005000	4.281129000	-0.018891000
F	3.177413000	1.147678000	0.638986000
F	5.560121000	0.160982000	0.067458000
F	5.818300000	-2.333175000	-0.988048000
F	3.593469000	-3.827547000	-1.455072000
F	1.187993000	-2.877687000	-0.893784000
F	1.007019000	1.466268000	-2.159555000
F	0.901772000	4.117336000	-2.314795000
F	0.358298000	5.610281000	-0.100835000
F	-0.064428000	4.367894000	2.279397000
F	0.047826000	1.737218000	2.476897000
C	-0.109026000	-0.899250000	1.487958000
C	-1.467787000	-0.857853000	1.880584000
C	0.763138000	-1.556185000	2.363592000
C	-1.872168000	-1.406096000	3.097933000
C	0.354564000	-2.124448000	3.566016000
H	1.815862000	-1.619475000	2.110669000
C	-0.975165000	-2.042739000	3.945226000
H	-2.913377000	-1.327567000	3.394935000
H	1.079283000	-2.617983000	4.205340000
H	-1.315391000	-2.463370000	4.885145000
C	-2.533987000	-0.116624000	1.099461000
H	-2.181244000	0.896914000	0.938989000
H	-3.451439000	-0.055402000	1.680699000
C	-3.627788000	0.400526000	-1.124266000
C	-3.484101000	-2.078619000	-0.284884000
C	-3.585471000	-0.083710000	-2.596571000
C	-4.181143000	-2.314030000	-1.640371000
C	-3.471887000	-1.597742000	-2.774008000
H	-4.497874000	0.283938000	-3.073721000
H	-5.226112000	-1.997582000	-1.597259000
H	-3.895748000	-1.890399000	-3.736594000
N	-2.883176000	-0.647281000	-0.267086000
H	-4.192986000	-3.393033000	-1.807951000
H	-2.418754000	-1.896266000	-2.794857000
H	-2.748114000	0.402160000	-3.103495000
C	-4.478044000	-2.302079000	0.851082000
H	-4.963716000	-3.265178000	0.674603000
H	-3.988220000	-2.359775000	1.822336000
H	-5.257041000	-1.540882000	0.889113000
C	-2.328440000	-3.080803000	-0.162704000
H	-2.749932000	-4.085824000	-0.236464000
H	-1.594704000	-2.951048000	-0.959506000
H	-1.808339000	-3.003195000	0.789181000
C	-5.054645000	0.625387000	-0.621344000
H	-5.465584000	1.479669000	-1.164467000
H	-5.718671000	-0.219128000	-0.794772000
H	-5.071951000	0.876385000	0.441647000
C	-2.892645000	1.745409000	-1.075736000
H	-3.058766000	2.295853000	-0.148140000
H	-1.822397000	1.618683000	-1.238750000
H	-3.285823000	2.358280000	-1.890034000
O	-0.372512000	-0.635212000	-1.028847000
H	-0.024785000	-0.332777000	-1.868524000

H	-1.938755000	-0.721894000	-0.728300000

C4

	Coordinates (Angstroms)		
	X	Y	Z
B	0.751753000	-0.243315000	0.217566000
H	-1.051013000	-2.331463000	-0.549498000
C	2.273615000	-0.759946000	-0.064001000
C	3.399469000	-0.026358000	0.292978000
C	2.548366000	-1.991861000	-0.647244000
C	4.696009000	-0.456277000	0.058239000
C	3.825505000	-2.456323000	-0.905397000
C	4.911779000	-1.676385000	-0.553361000
C	0.653154000	1.383598000	0.160435000
C	0.892600000	2.080268000	-1.014536000
C	0.394542000	2.188670000	1.262577000
C	0.831860000	3.454381000	-1.137905000
C	0.327371000	3.571229000	1.190884000
C	0.542535000	4.209803000	-0.016276000
F	3.283990000	1.151335000	0.922415000
F	5.734405000	0.295676000	0.421055000
F	6.148202000	-2.102248000	-0.788092000
F	4.016522000	-3.644993000	-1.475462000
F	1.545088000	-2.827694000	-0.972431000
F	1.189258000	1.396678000	-2.148485000
F	1.046109000	4.048468000	-2.309553000
F	0.480293000	5.534324000	-0.096389000
F	0.065826000	4.291412000	2.280524000
F	0.203454000	1.658485000	2.475277000
C	-0.012560000	-0.996149000	1.418029000
C	-1.360537000	-0.791230000	1.809936000
C	0.781099000	-1.810254000	2.238851000
C	-1.784721000	-1.307455000	3.034935000
C	0.319258000	-2.372892000	3.421790000
H	1.814811000	-1.991346000	1.968538000
C	-0.971439000	-2.095862000	3.837352000
H	-2.784109000	-1.079845000	3.383805000
H	0.975484000	-2.993086000	4.022489000
H	-1.348638000	-2.482835000	4.777757000
C	-2.354868000	0.078085000	1.039165000
H	-1.801637000	0.939687000	0.683144000
H	-3.072391000	0.468241000	1.772821000
C	-3.546882000	0.588118000	-1.062339000
C	-4.075642000	-1.548565000	0.191926000
C	-3.929461000	-0.058600000	-2.420641000
C	-5.077746000	-1.724737000	-0.973662000
C	-4.431774000	-1.497844000	-2.323555000
H	-4.690409000	0.579180000	-2.882665000
H	-5.925629000	-1.044877000	-0.855494000
H	-5.142295000	-1.707414000	-3.128131000
N	-3.048490000	-0.488967000	-0.137412000
H	-5.488650000	-2.735859000	-0.900015000
H	-3.598302000	-2.192069000	-2.452383000
H	-3.057900000	-0.031371000	-3.081000000
C	-4.914272000	-1.268937000	1.454378000
H	-5.758695000	-1.964708000	1.467065000

H	-4.361544000	-1.430576000	2.377817000
H	-5.319848000	-0.256578000	1.468617000
C	-3.371379000	-2.904764000	0.371313000
H	-4.088345000	-3.636583000	0.755816000
H	-2.998735000	-3.286130000	-0.582982000
H	-2.542173000	-2.858156000	1.076887000
C	-4.718256000	1.415098000	-0.483581000
H	-4.923312000	2.260348000	-1.147509000
H	-5.645451000	0.852938000	-0.383311000
H	-4.458385000	1.824796000	0.497074000
C	-2.455240000	1.615441000	-1.400073000
H	-2.233876000	2.309154000	-0.585436000
H	-1.534808000	1.128628000	-1.712410000
H	-2.808543000	2.217948000	-2.241205000
C	-0.840808000	-1.802059000	-1.473151000
O	-1.131318000	-2.079772000	-2.587604000
O	-0.060591000	-0.682757000	-1.218798000
H	0.232546000	-0.260588000	-2.046657000

C7

	Coordinates (Angstroms)		
	X	Y	Z
B	0.379772000	-0.200045000	0.246258000
C	1.779106000	-0.979491000	-0.085928000
C	3.003056000	-0.607113000	0.451365000
C	1.831216000	-2.063264000	-0.949886000
C	4.197801000	-1.243896000	0.159639000
C	3.001407000	-2.732133000	-1.271947000
C	4.196199000	-2.316072000	-0.713952000
C	0.611445000	1.416856000	0.084661000
C	0.861528000	1.933421000	-1.181374000
C	0.583844000	2.367161000	1.094544000
C	1.044862000	3.277858000	-1.450518000
C	0.758934000	3.725513000	0.872499000
C	0.991712000	4.186670000	-0.409145000
F	3.070264000	0.413263000	1.326428000
F	5.345410000	-0.841740000	0.713315000
F	5.333860000	-2.945492000	-1.010911000
F	2.990491000	-3.767808000	-2.116823000
F	0.710465000	-2.527258000	-1.536725000
F	0.898310000	1.106924000	-2.244704000
F	1.258679000	3.707343000	-2.697948000
F	1.163079000	5.488980000	-0.639136000
F	0.713988000	4.593363000	1.888359000
F	0.382765000	2.011776000	2.374330000
C	-0.271932000	-0.663965000	1.668411000
C	-1.621176000	-0.432624000	2.014757000
C	0.501524000	-1.332316000	2.623775000
C	-2.111190000	-0.786467000	3.270888000
C	0.007237000	-1.715387000	3.866709000
H	1.539198000	-1.557218000	2.402019000
C	-1.305959000	-1.425563000	4.204676000
H	-3.144364000	-0.564718000	3.520760000
H	0.654267000	-2.227436000	4.571593000
H	-1.704583000	-1.696548000	5.176166000
C	-2.579547000	0.265678000	1.077248000
H	-2.152568000	1.225316000	0.802619000

H	-3.534734000	0.450091000	1.563718000
C	-3.477697000	0.492751000	-1.293344000
C	-3.529049000	-1.848117000	-0.092413000
C	-3.336659000	-0.207646000	-2.668406000
C	-4.135302000	-2.241416000	-1.454141000
C	-3.295858000	-1.735091000	-2.612511000
H	-4.182301000	0.124517000	-3.276378000
H	-5.158962000	-1.870865000	-1.543891000
H	-3.655285000	-2.150077000	-3.555884000
N	-2.858333000	-0.446430000	-0.226929000
H	-4.201660000	-3.331181000	-1.466843000
H	-2.263253000	-2.079481000	-2.496070000
H	-2.430856000	0.159742000	-3.156432000
C	-4.608126000	-1.863063000	0.984420000
H	-5.124500000	-2.823236000	0.909481000
H	-4.190623000	-1.795701000	1.988346000
H	-5.351737000	-1.077000000	0.852262000
C	-2.434522000	-2.865112000	0.253631000
H	-2.890400000	-3.857261000	0.251682000
H	-1.628761000	-2.861187000	-0.483920000
H	-2.001859000	-2.697439000	1.236569000
C	-4.925446000	0.841050000	-0.949137000
H	-5.252813000	1.606822000	-1.656035000
H	-5.613645000	0.003011000	-1.037981000
H	-5.011449000	1.265727000	0.053417000
C	-2.679759000	1.797115000	-1.370990000
H	-2.883045000	2.482361000	-0.546764000
H	-1.609062000	1.603662000	-1.432014000
H	-2.977111000	2.301642000	-2.293057000
H	-0.358477000	-0.497648000	-0.678771000
H	-1.913633000	-0.636815000	-0.594245000

C8

	Coordinates (Angstroms)		
	X	Y	Z
B	0.779280000	-0.430380000	-0.061613000
C	2.380927000	-0.772408000	-0.243248000
C	3.266967000	-0.414060000	0.768199000
C	2.979587000	-1.370597000	-1.341794000
C	4.632065000	-0.625555000	0.711054000
C	4.343861000	-1.600463000	-1.443829000
C	5.178415000	-1.223923000	-0.410625000
C	0.687075000	1.202251000	0.135083000
C	0.947319000	2.051234000	-0.930760000
C	0.430744000	1.854444000	1.334022000
C	0.911782000	3.430681000	-0.862791000
C	0.384906000	3.235011000	1.454576000
C	0.623889000	4.030405000	0.349353000
F	2.800270000	0.148652000	1.893699000
F	5.422432000	-0.264381000	1.722197000
F	6.489155000	-1.437510000	-0.489252000
F	4.852904000	-2.180922000	-2.531413000
F	2.250187000	-1.774648000	-2.400486000
F	1.243632000	1.526624000	-2.145915000
F	1.152239000	4.178096000	-1.939228000
F	0.584244000	5.355514000	0.451468000
F	0.116388000	3.802048000	2.630689000

F	0.190089000	1.168500000	2.457656000
C	0.027888000	-1.370042000	1.030111000
C	-1.297977000	-1.214222000	1.511941000
C	0.792641000	-2.403699000	1.592396000
C	-1.699625000	-1.981863000	2.608623000
C	0.344529000	-3.212683000	2.626829000
H	1.795046000	-2.579954000	1.220816000
C	-0.907477000	-2.973966000	3.166770000
H	-2.667624000	-1.796617000	3.053349000
H	0.984303000	-3.993304000	3.024083000
H	-1.270615000	-3.546868000	4.013036000
C	-2.278483000	-0.147745000	1.010844000
H	-1.684451000	0.697971000	0.692200000
H	-2.848049000	0.195629000	1.880841000
C	-3.640710000	0.835423000	-0.816280000
C	-4.348355000	-1.384093000	0.225990000
C	-4.340225000	0.473031000	-2.157788000
C	-5.511526000	-1.237812000	-0.785302000
C	-5.037250000	-0.886240000	-2.178410000
H	-5.061743000	1.268671000	-2.367825000
H	-6.218515000	-0.477335000	-0.441668000
H	-5.878743000	-0.867351000	-2.876034000
N	-3.206343000	-0.442938000	-0.122767000
H	-6.059143000	-2.184549000	-0.784536000
H	-4.349763000	-1.654328000	-2.541635000
H	-3.598704000	0.502410000	-2.959767000
C	-4.965357000	-1.173494000	1.619710000
H	-5.899466000	-1.740735000	1.665589000
H	-4.339434000	-1.534625000	2.431873000
H	-5.204948000	-0.126881000	1.811561000
C	-3.827820000	-2.826776000	0.110940000
H	-4.593305000	-3.521700000	0.468701000
H	-3.602740000	-3.078028000	-0.928351000
H	-2.926644000	-2.990363000	0.699246000
C	-4.541766000	1.729295000	0.056979000
H	-4.678629000	2.691740000	-0.444574000
H	-5.532276000	1.309663000	0.228565000
H	-4.077624000	1.928332000	1.026878000
C	-2.429549000	1.696333000	-1.211908000
H	-1.995825000	2.248416000	-0.375773000
H	-1.656361000	1.092539000	-1.686398000
H	-2.767680000	2.439172000	-1.939010000
O	0.053076000	-0.702032000	-1.451107000
H	0.384764000	-0.211355000	-2.215292000
C	-0.682583000	-1.922527000	-1.866043000
H	-0.631834000	-2.565574000	-0.987176000
H	-0.134833000	-2.347574000	-2.704368000
O	-1.926637000	-1.568892000	-2.247744000
H	-2.405877000	-1.210945000	-1.438642000

TSC3-4

	Coordinates (Angstroms)		
	X	Y	Z
B	0.672214000	-0.401471000	-0.088809000
H	-2.277775000	-0.989375000	-0.882016000
C	2.267879000	-0.808841000	-0.246757000
C	3.149229000	-0.471765000	0.777112000

C	2.869024000	-1.450441000	-1.318306000
C	4.505733000	-0.738868000	0.748971000
C	4.223261000	-1.736539000	-1.395281000
C	5.051532000	-1.375602000	-0.351612000
C	0.664943000	1.238599000	0.097983000
C	0.983005000	2.046889000	-0.987897000
C	0.436296000	1.933072000	1.278153000
C	1.012988000	3.428925000	-0.944371000
C	0.457281000	3.316670000	1.369811000
C	0.742518000	4.072493000	0.249154000
F	2.688223000	0.121616000	1.888175000
F	5.289442000	-0.393563000	1.770544000
F	6.354075000	-1.640812000	-0.399859000
F	4.726652000	-2.354284000	-2.463748000
F	2.137382000	-1.850218000	-2.386072000
F	1.283958000	1.493407000	-2.178633000
F	1.306995000	4.141836000	-2.032974000
F	0.768362000	5.401156000	0.319166000
F	0.208789000	3.922678000	2.532370000
F	0.156794000	1.288424000	2.419738000
C	-0.077223000	-1.308115000	1.048138000
C	-1.341878000	-1.066540000	1.639044000
C	0.645241000	-2.397646000	1.557523000
C	-1.709177000	-1.772475000	2.786009000
C	0.226832000	-3.156157000	2.643420000
H	1.596615000	-2.657547000	1.108789000
C	-0.942367000	-2.808466000	3.298282000
H	-2.628298000	-1.506562000	3.293647000
H	0.835214000	-3.982993000	2.993872000
H	-1.263926000	-3.337061000	4.188914000
C	-2.326156000	-0.017657000	1.136644000
H	-1.767798000	0.863973000	0.850679000
H	-2.979958000	0.278448000	1.958124000
C	-3.616951000	0.842939000	-0.830924000
C	-4.251560000	-1.422634000	0.222359000
C	-4.169438000	0.383976000	-2.208293000
C	-5.340978000	-1.360830000	-0.871387000
C	-4.771555000	-1.019682000	-2.232148000
H	-4.919969000	1.121058000	-2.507687000
H	-6.113249000	-0.635536000	-0.603312000
H	-5.547130000	-1.081522000	-2.998985000
N	-3.153442000	-0.387896000	-0.056489000
H	-5.830721000	-2.337862000	-0.886453000
H	-4.008784000	-1.753868000	-2.507480000
H	-3.367285000	0.442695000	-2.949238000
C	-4.945521000	-1.233622000	1.576081000
H	-5.840346000	-1.862114000	1.582500000
H	-4.330633000	-1.547596000	2.416716000
H	-5.263426000	-0.203413000	1.741344000
C	-3.628240000	-2.828476000	0.158872000
H	-4.370997000	-3.552321000	0.503744000
H	-3.352859000	-3.100651000	-0.862573000
H	-2.745929000	-2.930118000	0.787762000
C	-4.643630000	1.671369000	-0.045130000
H	-4.824727000	2.603282000	-0.587476000
H	-5.604304000	1.174777000	0.079255000
H	-4.259301000	1.937267000	0.943169000

C	-2.441778000	1.781109000	-1.147505000
H	-2.110634000	2.369113000	-0.289473000
H	-1.588795000	1.245435000	-1.563782000
H	-2.785657000	2.490194000	-1.904495000
C	-1.373469000	-1.469577000	-1.750610000
O	-1.312949000	-2.118783000	-2.723069000
O	-0.027863000	-0.672276000	-1.477030000
H	0.569104000	-0.801415000	-2.227681000

TSC1-7

	Coordinates (Angstroms)		
	X	Y	Z
C	0.646285000	2.105081000	-0.898089000
C	0.415789000	1.449516000	0.306538000
C	-0.027008000	2.257238000	1.347355000
C	-0.263473000	3.613771000	1.197870000
C	-0.045449000	4.214421000	-0.028472000
C	0.412226000	3.452955000	-1.089475000
B	0.701405000	-0.108524000	0.476699000
C	2.127104000	-0.624554000	-0.038171000
C	2.313277000	-1.887092000	-0.590568000
C	3.545416000	-2.360085000	-1.004767000
C	4.664433000	-1.561197000	-0.850009000
C	4.532568000	-0.304909000	-0.287411000
C	3.280931000	0.138814000	0.104673000
F	1.271344000	-2.708542000	-0.758201000
F	3.664547000	-3.572531000	-1.542471000
F	5.857331000	-2.000273000	-1.232873000
F	5.608441000	0.462659000	-0.121374000
F	3.231025000	1.352404000	0.668061000
F	-0.244035000	1.753396000	2.566363000
F	-0.690214000	4.344706000	2.226584000
F	-0.269969000	5.513154000	-0.185884000
F	0.614810000	4.019278000	-2.277422000
F	1.074251000	1.414357000	-1.963946000
C	-0.033546000	-0.965781000	1.574968000
C	0.817891000	-1.659191000	2.447538000
C	0.353643000	-2.304906000	3.587131000
C	-1.000459000	-2.272192000	3.876847000
C	-1.868585000	-1.624425000	3.006221000
C	-1.419699000	-0.988745000	1.850773000
C	-2.477420000	-0.319490000	0.989887000
N	-2.669288000	-0.806832000	-0.388552000
C	-3.125785000	-2.239869000	-0.483284000
C	-3.400820000	-2.614432000	-1.952105000
C	-4.317303000	-1.652679000	-2.678887000
C	-3.665634000	-0.287153000	-2.628125000
C	-3.415035000	0.224909000	-1.199921000
C	-4.369654000	-2.569377000	0.363821000
C	-1.996464000	-3.191961000	-0.047055000
C	-2.543782000	1.478868000	-1.380389000
C	-4.751224000	0.672650000	-0.573792000
H	-0.000280000	-0.602802000	-1.206102000
H	1.884397000	-1.674751000	2.249547000
H	1.047978000	-2.819285000	4.242107000
H	-1.387123000	-2.755577000	4.767451000
H	-2.932125000	-1.627707000	3.222724000

H	-2.214121000	0.734093000	0.921166000
H	-3.415683000	-0.350738000	1.556005000
H	-0.721341000	-0.685753000	-0.944087000
H	-2.441359000	-2.649737000	-2.483014000
H	-3.806049000	-3.630471000	-1.959628000
H	-5.311052000	-1.631607000	-2.219734000
H	-4.454697000	-1.970154000	-3.716772000
H	-4.267526000	0.462888000	-3.149473000
H	-2.701776000	-0.343160000	-3.148815000
H	-1.587481000	1.214005000	-1.831329000
H	-2.361941000	2.029850000	-0.455926000
H	-3.054160000	2.164209000	-2.062737000
H	-4.609153000	1.108124000	0.417877000
H	-5.472799000	-0.139637000	-0.486766000
H	-5.199374000	1.445441000	-1.204906000
H	-4.224393000	-2.318699000	1.416420000
H	-4.555963000	-3.646217000	0.312599000
H	-5.270040000	-2.063416000	0.014286000
H	-1.046460000	-2.893370000	-0.489606000
H	-2.234795000	-4.198278000	-0.404845000
H	-1.871822000	-3.255413000	1.031777000

TSC7

	Coordinates (Angstroms)		
	X	Y	Z
H	0.265766000	-0.421914000	-0.776897000
B	0.835922000	-0.095609000	0.452414000
H	-1.942180000	-1.102746000	-1.086654000
O	-1.148645000	-1.512041000	-1.784065000
C	2.329423000	-0.609630000	0.121768000
C	3.480511000	0.154294000	0.262562000
C	2.534868000	-1.909580000	-0.334487000
C	4.745453000	-0.314938000	-0.053021000
C	3.780501000	-2.412520000	-0.664441000
C	4.895591000	-1.605085000	-0.525842000
C	0.616881000	1.504133000	0.391449000
C	0.978658000	2.229271000	-0.738995000
C	0.044193000	2.264015000	1.403602000
C	0.744987000	3.580847000	-0.902188000
C	-0.209823000	3.621765000	1.283684000
C	0.135411000	4.284580000	0.121558000
F	3.420368000	1.406548000	0.740352000
F	5.818418000	0.463191000	0.101017000
F	6.103034000	-2.069676000	-0.834722000
F	3.918671000	-3.663872000	-1.104339000
F	1.501311000	-2.749488000	-0.448132000
F	1.600054000	1.610266000	-1.763918000
F	1.102632000	4.207541000	-2.022003000
F	-0.104438000	5.585227000	-0.007856000
F	-0.786241000	4.292344000	2.280797000
F	-0.308853000	1.707584000	2.569359000
C	0.039826000	-0.972126000	1.541256000
C	-1.352582000	-0.995402000	1.803166000
C	0.860759000	-1.703398000	2.416956000
C	-1.814853000	-1.651300000	2.946757000
C	0.378704000	-2.394901000	3.518715000
H	1.932200000	-1.708180000	2.255180000

C	-0.976791000	-2.355714000	3.797386000
H	-2.865978000	-1.589981000	3.197681000
H	1.063418000	-2.935120000	4.163408000
H	-1.382475000	-2.851312000	4.672502000
C	-2.383461000	-0.179056000	1.030478000
H	-1.909558000	0.761979000	0.782784000
H	-3.197502000	0.058567000	1.719311000
C	-3.631747000	0.457854000	-1.018223000
C	-3.844201000	-1.933955000	-0.121964000
C	-3.837910000	-0.014972000	-2.479208000
C	-4.752341000	-2.074681000	-1.368609000
C	-4.131520000	-1.511246000	-2.633520000
H	-4.655373000	0.581689000	-2.894161000
H	-5.718172000	-1.596281000	-1.190132000
H	-4.800274000	-1.677609000	-3.481579000
N	-2.964879000	-0.683946000	-0.252734000
H	-4.962054000	-3.140649000	-1.490894000
H	-3.203887000	-2.043983000	-2.852181000
H	-2.943967000	0.235259000	-3.057877000
C	-4.749931000	-1.939662000	1.116973000
H	-5.503239000	-2.719886000	0.976687000
H	-4.208208000	-2.185662000	2.027183000
H	-5.275242000	-0.997080000	1.266526000
C	-2.933131000	-3.171484000	-0.065357000
H	-3.551174000	-4.044967000	0.159530000
H	-2.428092000	-3.343515000	-1.016107000
H	-2.172123000	-3.092036000	0.710124000
C	-4.954099000	0.935146000	-0.391434000
H	-5.269052000	1.840024000	-0.917967000
H	-5.765933000	0.215557000	-0.470956000
H	-4.824173000	1.199420000	0.661030000
C	-2.731340000	1.704629000	-1.071077000
H	-2.782739000	2.300105000	-0.157023000
H	-1.686473000	1.467498000	-1.266109000
H	-3.085105000	2.337988000	-1.888249000
C	-0.182082000	-0.657196000	-1.944385000
H	-0.449846000	0.371331000	-2.223292000
O	0.847261000	-1.151590000	-2.674803000
H	1.442726000	-0.428906000	-2.900224000

TSC8

	Coordinates (Angstroms)		
	X	Y	Z
B	0.822720000	-0.257203000	-0.046088000
H	-2.329586000	-1.413052000	-0.702052000
C	2.394006000	-0.741552000	-0.199862000
C	3.516325000	0.055776000	-0.013512000
C	2.681639000	-2.055510000	-0.558654000
C	4.816328000	-0.400198000	-0.183421000
C	3.959571000	-2.551421000	-0.740599000
C	5.041771000	-1.710457000	-0.554424000
C	0.716891000	1.398636000	0.021664000
C	0.686234000	2.213778000	-1.100772000
C	0.664494000	2.099754000	1.223441000
C	0.527926000	3.588620000	-1.070597000
C	0.509230000	3.473430000	1.305729000
C	0.428337000	4.226878000	0.149265000

F	3.411665000	1.340174000	0.362248000
F	5.851872000	0.420067000	0.006782000
F	6.282534000	-2.160132000	-0.724307000
F	4.157854000	-3.826013000	-1.081380000
F	1.684081000	-2.942590000	-0.709024000
F	0.825413000	1.686407000	-2.342517000
F	0.473025000	4.293595000	-2.201665000
F	0.276250000	5.547498000	0.211855000
F	0.451947000	4.079501000	2.493080000
F	0.799197000	1.469125000	2.398128000
C	0.056782000	-0.938008000	1.227323000
C	-1.261552000	-0.641092000	1.658642000
C	0.842095000	-1.699329000	2.103586000
C	-1.649583000	-0.988633000	2.953571000
C	0.413358000	-2.110438000	3.359129000
H	1.858939000	-1.948746000	1.826770000
C	-0.836594000	-1.720261000	3.806656000
H	-2.621347000	-0.676726000	3.314864000
H	1.071743000	-2.695778000	3.992305000
H	-1.180120000	-1.974166000	4.803562000
C	-2.261004000	0.164974000	0.834929000
H	-1.698123000	0.887040000	0.255884000
H	-2.900245000	0.734294000	1.512147000
C	-3.629714000	0.367396000	-1.258134000
C	-4.236338000	-1.437216000	0.466191000
C	-4.146801000	-0.514267000	-2.424092000
C	-5.303750000	-1.765537000	-0.605664000
C	-4.706729000	-1.870662000	-1.995313000
H	-4.914503000	0.064513000	-2.946579000
H	-6.099208000	-1.016817000	-0.602626000
H	-5.458576000	-2.210674000	-2.711446000
N	-3.126214000	-0.567037000	-0.154582000
H	-5.771264000	-2.708202000	-0.310433000
H	-3.908484000	-2.617515000	-1.993765000
H	-3.333883000	-0.668335000	-3.139417000
C	-4.942239000	-0.782101000	1.659293000
H	-5.845204000	-1.361441000	1.870804000
H	-4.340059000	-0.794230000	2.565383000
H	-5.248258000	0.244434000	1.463378000
C	-3.635546000	-2.778312000	0.919786000
H	-4.405738000	-3.324133000	1.471314000
H	-3.324579000	-3.385006000	0.069200000
H	-2.774689000	-2.656415000	1.572294000
C	-4.700306000	1.355027000	-0.765887000
H	-4.873258000	2.088088000	-1.558327000
H	-5.659735000	0.895372000	-0.539355000
H	-4.356582000	1.902599000	0.115485000
C	-2.500888000	1.233424000	-1.834718000
H	-2.246283000	2.082968000	-1.197510000
H	-1.598461000	0.661528000	-2.033370000
H	-2.855738000	1.643041000	-2.783673000
O	0.153431000	-0.635218000	-1.420739000
C	-0.721464000	-1.863102000	-1.976559000
H	0.036387000	-2.596901000	-2.254613000
H	-1.142052000	-1.354384000	-2.857409000
O	-1.551300000	-2.258341000	-1.065301000
H	0.612318000	-0.223873000	-2.162774000

TSC9

	Coordinates (Angstroms)		
	X	Y	Z
H	0.268623000	-0.524325000	-0.979416000
B	0.865114000	-0.233877000	0.297546000
H	-2.175013000	-1.075145000	-1.011795000
O	-1.313478000	-1.641233000	-1.743455000
C	2.379340000	-0.688895000	-0.040531000
C	3.495489000	0.084281000	0.255781000
C	2.657712000	-1.915134000	-0.636784000
C	4.791273000	-0.305335000	-0.043731000
C	3.935387000	-2.337804000	-0.956478000
C	5.012661000	-1.521716000	-0.660844000
C	0.652237000	1.364770000	0.298880000
C	0.963272000	2.122943000	-0.824460000
C	0.193805000	2.094327000	1.387318000
C	0.779374000	3.490393000	-0.902336000
C	-0.001631000	3.466013000	1.353573000
C	0.287730000	4.168612000	0.199239000
F	3.370386000	1.262592000	0.883936000
F	5.824591000	0.479290000	0.264102000
F	6.249509000	-1.910003000	-0.956193000
F	4.138241000	-3.521487000	-1.535311000
F	1.662283000	-2.768091000	-0.913759000
F	1.462898000	1.523876000	-1.917956000
F	1.074020000	4.158519000	-2.017182000
F	0.103203000	5.484123000	0.149355000
F	-0.463209000	4.111895000	2.424686000
F	-0.095037000	1.490136000	2.548043000
C	0.056882000	-1.164921000	1.321306000
C	-1.321187000	-1.112221000	1.654323000
C	0.856070000	-2.036387000	2.081772000
C	-1.776447000	-1.828551000	2.762912000
C	0.374337000	-2.786341000	3.144796000
H	1.914722000	-2.108594000	1.864374000
C	-0.956778000	-2.665269000	3.504381000
H	-2.807627000	-1.716948000	3.071170000
H	1.044020000	-3.432550000	3.701539000
H	-1.357503000	-3.202909000	4.356545000
C	-2.329866000	-0.180048000	0.992631000
H	-1.805604000	0.729480000	0.733556000
H	-3.082058000	0.090706000	1.734489000
C	-3.588605000	0.610545000	-1.021238000
C	-4.050817000	-1.751199000	-0.099889000
C	-3.903479000	0.154862000	-2.467914000
C	-5.004727000	-1.781390000	-1.319077000
C	-4.345239000	-1.304784000	-2.598972000
H	-4.677432000	0.827790000	-2.848472000
H	-5.898792000	-1.185997000	-1.119580000
H	-5.034160000	-1.414921000	-3.439840000
N	-3.031597000	-0.605022000	-0.266091000
H	-5.347659000	-2.813594000	-1.426395000
H	-3.473098000	-1.926642000	-2.812689000
H	-3.015130000	0.314733000	-3.084833000
C	-4.905853000	-1.645098000	1.167897000
H	-5.727159000	-2.359758000	1.067730000

H	-4.360886000	-1.917899000	2.068728000
H	-5.344016000	-0.658287000	1.310830000
C	-3.290186000	-3.086282000	-0.075283000
H	-4.011950000	-3.881850000	0.127479000
H	-2.804876000	-3.287166000	-1.029970000
H	-2.527250000	-3.119497000	0.699887000
C	-4.811720000	1.235945000	-0.335228000
H	-5.032656000	2.178658000	-0.842344000
H	-5.710023000	0.625069000	-0.385524000
H	-4.604524000	1.470280000	0.711840000
C	-2.546739000	1.733345000	-1.129025000
H	-2.450055000	2.318516000	-0.211797000
H	-1.565767000	1.360711000	-1.414434000
H	-2.881492000	2.416793000	-1.912926000
C	-0.289678000	-0.870916000	-2.012852000
H	0.547063000	-1.353487000	-2.531384000
H	-0.493937000	0.121122000	-2.442725000

D1(B(C_6F_5)$_3$)

	Coordinates (Angstroms)		
	X	Y	Z
B	0.002446000	-0.002949000	0.002781000
C	1.542451000	-0.285786000	0.001187000
C	2.106690000	-1.300693000	0.770109000
C	2.426820000	0.464051000	-0.770015000
C	3.465080000	-1.556164000	0.786660000
C	3.787538000	0.221910000	-0.791315000
C	4.307077000	-0.791593000	-0.003572000
C	-1.015617000	-1.192980000	0.005205000
C	-2.176318000	-1.168141000	0.774444000
C	-0.814743000	-2.335540000	-0.765445000
C	-3.082683000	-2.211753000	0.790737000
C	-1.710670000	-3.388141000	-0.786451000
C	-2.848503000	-3.324654000	0.000496000
C	-0.521403000	1.473294000	0.000526000
C	0.064055000	2.463263000	0.785075000
C	-1.600053000	1.870101000	-0.785281000
C	-0.392972000	3.767708000	0.803485000
C	-2.069216000	3.170117000	-0.805159000
C	-1.463269000	4.121221000	-0.001333000
F	1.336204000	-2.058427000	1.554708000
F	3.968575000	-2.523020000	1.548435000
F	5.609822000	-1.030106000	-0.006316000
F	4.598212000	0.947903000	-1.555570000
F	1.972468000	1.444914000	-1.553943000
F	-2.441155000	-0.121513000	1.560564000
F	-4.171709000	-2.157253000	1.552122000
F	-3.712717000	-4.328167000	-0.002281000
F	-1.492476000	-4.454856000	-1.550294000
F	0.261907000	-2.438873000	-1.548975000
F	-2.209054000	0.991362000	-1.585863000
F	-3.090899000	3.514698000	-1.583722000
F	-1.907221000	5.369109000	-0.002437000
F	0.181402000	4.680799000	1.581129000
F	1.092436000	2.167590000	1.584663000

D1(^{t}Bu$_3$P)

	Coordinates (Angstroms)		
	X	Y	Z
P	0.000206000	-0.000323000	-0.701783000
C	-1.484035000	-1.001290000	-0.008470000
C	-1.233081000	-2.503129000	-0.227577000
C	-1.843101000	-0.784939000	1.467601000
C	-2.711520000	-0.665106000	-0.879674000
H	-0.942731000	-2.723188000	-1.258785000
H	-0.477628000	-2.910638000	0.444025000
H	-2.166711000	-3.040906000	-0.027565000
H	-2.185271000	0.231277000	1.667723000
H	-2.663124000	-1.461527000	1.738388000
H	-1.006991000	-0.998656000	2.134838000
H	-3.539042000	-1.323829000	-0.590786000
H	-3.058903000	0.359572000	-0.764050000
H	-2.504117000	-0.836283000	-1.939998000
C	1.609677000	-0.784294000	-0.008393000
C	2.783635000	0.185889000	-0.224744000
C	1.600629000	-1.206008000	1.466761000
C	1.934374000	-2.013872000	-0.881524000
H	2.828128000	0.550962000	-1.254746000
H	2.758198000	1.041986000	0.449525000
H	3.717466000	-0.352514000	-0.027292000
H	0.886959000	-2.006351000	1.665945000
H	2.594307000	-1.585054000	1.736082000
H	1.372778000	-0.374974000	2.135771000
H	2.914681000	-2.406723000	-0.586930000
H	1.215626000	-2.823816000	-0.774587000
H	1.987228000	-1.744965000	-1.940608000
C	-0.125501000	1.785291000	-0.008111000
C	-1.552042000	2.317631000	-0.227180000
C	0.242667000	1.990822000	1.467302000
C	0.778283000	2.679313000	-0.881614000
H	-1.886838000	2.178328000	-1.259027000
H	-2.283654000	1.864994000	0.442230000
H	-1.553151000	3.394680000	-0.024909000
H	1.292970000	1.775213000	1.667468000
H	0.072075000	3.041108000	1.734410000
H	-0.362902000	1.378537000	2.136978000
H	0.620915000	3.726303000	-0.596812000
H	1.839442000	2.468637000	-0.765010000
H	0.527284000	2.580827000	-1.941801000

D1_CO

	Coordinates (Angstroms)		
	X	Y	Z
B	0.016678000	-0.009050000	0.618236000
C	1.592157000	0.115637000	0.232810000
C	2.545413000	-0.659133000	0.878312000
C	2.074088000	0.938702000	-0.775708000
C	3.892227000	-0.631979000	0.572387000
C	3.416854000	0.995297000	-1.113156000
C	4.330685000	0.209638000	-0.434869000
C	-0.680186000	-1.431291000	0.241872000
C	-1.851275000	-1.823058000	0.874677000

C	-0.218766000	-2.288867000	-0.747109000
C	-2.537579000	-2.982412000	0.570842000
C	-0.877128000	-3.461863000	-1.081128000
C	-2.039937000	-3.811684000	-0.418833000
C	-0.880486000	1.295746000	0.242726000
C	-0.695910000	2.499699000	0.906208000
C	-1.819100000	1.314330000	-0.779663000
C	-1.388767000	3.656332000	0.605154000
C	-2.535170000	2.452372000	-1.113144000
C	-2.321704000	3.628034000	-0.416415000
F	2.151007000	-1.500345000	1.852883000
F	4.759606000	-1.398821000	1.227903000
F	5.618735000	0.258304000	-0.751397000
F	3.833000000	1.797221000	-2.090716000
F	1.248910000	1.703664000	-1.497507000
F	-2.369624000	-1.033202000	1.833936000
F	-3.657198000	-3.304681000	1.212994000
F	-2.676934000	-4.932640000	-0.733926000
F	-0.402079000	-4.250696000	-2.042353000
F	0.877524000	-2.000376000	-1.455563000
F	-2.053069000	0.226555000	-1.521211000
F	-3.422722000	2.425170000	-2.104808000
F	-3.003690000	4.723146000	-0.728409000
F	-1.170001000	4.781654000	1.280224000
F	0.214231000	2.565393000	1.896397000
C	0.017191000	-0.021866000	2.254331000
O	0.013061000	-0.044953000	3.370391000

D1-2

	Coordinates (Angstroms)		
	X	Y	Z
B	-1.301936000	0.002392000	-0.915447000
P	2.251042000	0.013902000	1.484864000
C	-1.928560000	-1.238448000	-0.183228000
C	-2.142059000	-1.253747000	1.192288000
C	-2.276925000	-2.403785000	-0.860982000
C	-2.620837000	-2.361696000	1.865589000
C	-2.784423000	-3.520106000	-0.223891000
C	-2.945813000	-3.501394000	1.151463000
C	-1.739675000	1.446560000	-0.485555000
C	-0.855082000	2.523253000	-0.475754000
C	-3.031679000	1.727878000	-0.042354000
C	-1.200657000	3.778892000	-0.013856000
C	-3.418962000	2.978155000	0.401716000
C	-2.492251000	4.006425000	0.428216000
C	-0.247371000	-0.218715000	-2.056944000
C	0.660043000	-1.277819000	-2.036140000
C	-0.148935000	0.633985000	-3.155855000
C	1.618463000	-1.464723000	-3.014333000
C	0.797927000	0.476298000	-4.150013000
C	1.694099000	-0.575849000	-4.072014000
F	-1.850559000	-0.184440000	1.936429000
F	-2.754730000	-2.344194000	3.189245000
F	-3.412302000	-4.570208000	1.780128000
F	-3.111738000	-4.608859000	-0.915061000
F	-2.138053000	-2.478008000	-2.188054000
F	0.398598000	2.373141000	-0.903147000

F	-0.306821000	4.763838000	0.008400000
F	-2.843612000	5.206012000	0.864778000
F	-4.666206000	3.200586000	0.807286000
F	-3.970480000	0.778227000	-0.045017000
F	-0.998308000	1.654249000	-3.297833000
F	0.852845000	1.318689000	-5.178048000
F	2.609357000	-0.740893000	-5.014113000
F	2.471302000	-2.484227000	-2.946557000
F	0.650252000	-2.166801000	-1.040960000
C	2.206713000	1.446079000	2.775322000
C	3.325355000	1.584058000	3.824128000
C	2.150680000	2.773834000	1.999401000
C	0.873797000	1.325012000	3.534478000
H	3.296449000	0.801102000	4.577918000
H	4.323234000	1.600967000	3.391062000
H	3.182714000	2.537332000	4.348102000
H	1.406501000	2.756952000	1.203085000
H	1.876159000	3.574226000	2.695684000
H	3.115625000	3.041166000	1.565400000
H	0.723395000	2.232534000	4.130734000
H	0.024657000	1.225608000	2.853793000
H	0.867080000	0.480777000	4.226428000
C	2.304647000	-1.634905000	2.483575000
C	2.849982000	-2.811165000	1.658952000
C	3.070649000	-1.618499000	3.810182000
C	0.825924000	-1.982427000	2.752301000
H	2.435479000	-2.850607000	0.650526000
H	3.937583000	-2.805101000	1.596564000
H	2.561740000	-3.738477000	2.167533000
H	2.563436000	-1.020186000	4.567930000
H	3.134034000	-2.641967000	4.199481000
H	4.090156000	-1.242821000	3.691237000
H	0.776929000	-2.888287000	3.368598000
H	0.276767000	-1.198442000	3.271545000
H	0.311901000	-2.190118000	1.810542000
C	3.822021000	0.278869000	0.400026000
C	3.393793000	1.280788000	-0.693103000
C	5.077094000	0.797603000	1.108219000
C	4.218488000	-0.994070000	-0.364286000
H	2.596539000	0.859680000	-1.312504000
H	3.042927000	2.233474000	-0.303305000
H	4.254083000	1.484285000	-1.341932000
H	5.370834000	0.150815000	1.939693000
H	5.908797000	0.817762000	0.393130000
H	4.952875000	1.813855000	1.485400000
H	4.852666000	-0.700471000	-1.208618000
H	4.797813000	-1.685054000	0.246019000
H	3.359076000	-1.525565000	-0.772344000

D2

	Coordinates (Angstroms)		
	X	Y	Z
B	1.095440000	0.104146000	-0.015678000
P	-3.037808000	-0.112351000	-0.961928000
C	0.566646000	-0.818613000	1.225681000
C	0.115984000	-2.107313000	0.966394000
C	0.535429000	-0.448749000	2.562828000

C	-0.389556000	-2.953663000	1.937254000
C	0.041027000	-1.266806000	3.565730000
C	-0.436175000	-2.525212000	3.250199000
C	2.417103000	-0.588094000	-0.682170000
C	2.616363000	-0.776826000	-2.039593000
C	3.450953000	-1.041729000	0.126100000
C	3.731423000	-1.401919000	-2.572919000
C	4.583599000	-1.669150000	-0.360492000
C	4.721921000	-1.857618000	-1.724189000
C	1.269428000	1.700430000	0.291888000
C	0.173176000	2.411732000	0.764663000
C	2.392239000	2.474009000	0.036528000
C	0.153738000	3.777906000	0.957523000
C	2.423560000	3.849295000	0.227890000
C	1.298632000	4.507126000	0.685502000
F	0.147838000	-2.597316000	-0.284207000
F	-0.849009000	-4.164600000	1.616041000
F	-0.930326000	-3.315959000	4.198891000
F	0.019868000	-0.850105000	4.832753000
F	1.002070000	0.743922000	2.958594000
F	1.714985000	-0.323132000	-2.938358000
F	3.859142000	-1.559662000	-3.891012000
F	5.803049000	-2.459591000	-2.213387000
F	5.542979000	-2.084855000	0.467131000
F	3.397928000	-0.849629000	1.453894000
F	3.528512000	1.930442000	-0.422821000
F	3.531638000	4.542924000	-0.037596000
F	1.314643000	5.824756000	0.864404000
F	-0.945035000	4.392347000	1.398619000
F	-0.954857000	1.749613000	1.070108000
H	-1.128412000	-0.004113000	-0.833901000
O	-0.068669000	0.006223000	-1.054146000
C	-3.368068000	-1.727139000	-1.921937000
C	-3.273399000	-2.927859000	-0.965247000
C	-4.709397000	-1.789046000	-2.662904000
C	-2.218459000	-1.917169000	-2.933371000
H	-2.331854000	-2.935576000	-0.413912000
H	-4.096940000	-2.981284000	-0.254778000
H	-3.303575000	-3.842555000	-1.566811000
H	-4.772782000	-1.049850000	-3.462251000
H	-4.811781000	-2.777624000	-3.124966000
H	-5.561181000	-1.645104000	-1.997029000
H	-2.346992000	-2.890449000	-3.419300000
H	-2.202287000	-1.164051000	-3.718131000
H	-1.245419000	-1.922825000	-2.437017000
C	-3.531586000	1.386743000	-2.029610000
C	-5.039216000	1.648850000	-2.139339000
C	-2.824081000	2.624500000	-1.440949000
C	-2.965487000	1.227046000	-3.450913000
H	-5.577492000	0.791352000	-2.545659000
H	-5.491451000	1.911092000	-1.183066000
H	-5.201081000	2.493676000	-2.818727000
H	-1.737755000	2.496099000	-1.449041000
H	-3.060237000	3.489382000	-2.070579000
H	-3.127542000	2.865262000	-0.425137000
H	-3.093843000	2.180645000	-3.973965000
H	-1.895669000	0.999310000	-3.448960000

H	-3.484175000	0.466108000	-4.033869000
C	-4.051906000	-0.112761000	0.653973000
C	-4.148160000	1.300198000	1.254793000
C	-5.481503000	-0.654168000	0.513547000
C	-3.266613000	-0.951033000	1.680950000
H	-3.171179000	1.758794000	1.401179000
H	-4.769760000	1.972496000	0.664659000
H	-4.617671000	1.207835000	2.239925000
H	-5.512563000	-1.704883000	0.226761000
H	-5.981450000	-0.568599000	1.485023000
H	-6.067777000	-0.083298000	-0.209061000
H	-3.803320000	-0.928395000	2.635880000
H	-3.149616000	-1.995124000	1.397565000
H	-2.273629000	-0.526835000	1.846964000
H	0.080167000	0.453041000	-1.895660000

D3

	Coordinates (Angstroms)		
	X	Y	Z
B	1.112775000	0.126265000	-0.210406000
P	-3.291727000	-0.178675000	-0.898299000
C	0.641529000	-0.870490000	1.026730000
C	0.222548000	-2.160499000	0.721417000
C	0.626309000	-0.571602000	2.382067000
C	-0.239873000	-3.064594000	1.663692000
C	0.171688000	-1.445754000	3.357556000
C	-0.276766000	-2.701249000	2.995855000
C	2.575517000	-0.461556000	-0.717068000
C	2.851036000	-0.917556000	-1.995561000
C	3.626643000	-0.591028000	0.180582000
C	4.065535000	-1.480858000	-2.362017000
C	4.853435000	-1.142908000	-0.137324000
C	5.073481000	-1.598449000	-1.425641000
C	1.182023000	1.731377000	0.205620000
C	0.050736000	2.317789000	0.759952000
C	2.186629000	2.631137000	-0.112035000
C	-0.097760000	3.671102000	0.987760000
C	2.089020000	4.000238000	0.105759000
C	0.938425000	4.527281000	0.656284000
F	0.252647000	-2.613147000	-0.543794000
F	-0.667562000	-4.277464000	1.295409000
F	-0.734432000	-3.549765000	3.916396000
F	0.156898000	-1.082916000	4.643705000
F	1.054124000	0.618256000	2.834630000
F	1.939339000	-0.822680000	-2.984759000
F	4.270248000	-1.906193000	-3.612631000
F	6.247768000	-2.134524000	-1.759924000
F	5.824875000	-1.236070000	0.775097000
F	3.482419000	-0.142480000	1.441024000
F	3.335587000	2.224355000	-0.681543000
F	3.095577000	4.813953000	-0.226417000
F	0.823957000	5.838948000	0.863242000
F	-1.226479000	4.159769000	1.512949000
F	-0.998578000	1.548829000	1.111747000
H	-1.909730000	-0.056783000	-0.658433000
O	0.052073000	0.016923000	-1.225623000
C	-3.436437000	-1.773657000	-1.887298000

C	-3.250791000	-2.990581000	-0.967246000
C	-4.786121000	-1.882641000	-2.607442000
C	-2.274692000	-1.816084000	-2.900848000
H	-2.294225000	-2.963258000	-0.445693000
H	-4.054037000	-3.114296000	-0.242542000
H	-3.247087000	-3.879977000	-1.603980000
H	-4.932145000	-1.087551000	-3.339715000
H	-4.805768000	-2.834903000	-3.146394000
H	-5.629301000	-1.876018000	-1.914345000
H	-2.247516000	-2.823104000	-3.327809000
H	-2.401197000	-1.118778000	-3.725544000
H	-1.312151000	-1.624216000	-2.420898000
C	-3.707876000	1.369541000	-1.882430000
C	-5.220181000	1.619953000	-1.937148000
C	-2.970235000	2.556645000	-1.233908000
C	-3.166641000	1.260636000	-3.316563000
H	-5.753788000	0.792304000	-2.409056000
H	-5.655634000	1.793555000	-0.952913000
H	-5.395589000	2.516778000	-2.539034000
H	-1.889610000	2.394844000	-1.244506000
H	-3.183327000	3.445520000	-1.835404000
H	-3.280996000	2.767188000	-0.214597000
H	-3.319309000	2.233176000	-3.794096000
H	-2.095695000	1.049248000	-3.336767000
H	-3.693148000	0.518218000	-3.915410000
C	-4.115772000	-0.256068000	0.793640000
C	-4.214510000	1.136425000	1.438146000
C	-5.528725000	-0.848974000	0.703274000
C	-3.216264000	-1.103508000	1.713734000
H	-3.239191000	1.606005000	1.559502000
H	-4.876463000	1.811651000	0.897680000
H	-4.639459000	0.995614000	2.436398000
H	-5.534642000	-1.884045000	0.363175000
H	-5.966955000	-0.830859000	1.705696000
H	-6.176932000	-0.262385000	0.048468000
H	-3.649188000	-1.077677000	2.718282000
H	-3.142068000	-2.146913000	1.416954000
H	-2.210162000	-0.682877000	1.774023000
H	0.311806000	0.438700000	-2.044956000

D4

	Coordinates (Angstroms)		
	X	Y	Z
B	-1.455171000	0.047406000	-0.341014000
P	3.400091000	-0.109929000	-0.435074000
C	-0.873916000	-1.320074000	0.310914000
C	-0.085247000	-2.286982000	-0.295120000
C	-1.285351000	-1.629223000	1.602812000
C	0.290057000	-3.462865000	0.336718000
C	-0.911828000	-2.775221000	2.276931000
C	-0.106762000	-3.701813000	1.636863000
C	-0.915622000	1.463644000	0.241715000
C	-1.211571000	2.632200000	-0.456308000
C	-0.197013000	1.657274000	1.413691000
C	-0.825399000	3.894842000	-0.052155000
C	0.174564000	2.911235000	1.876009000
C	-0.121818000	4.034784000	1.131052000

C	-3.067314000	-0.072238000	-0.504706000
C	-3.618712000	-1.067787000	-1.305009000
C	-3.987860000	0.699933000	0.194056000
C	-4.978593000	-1.279201000	-1.438583000
C	-5.357135000	0.514654000	0.092434000
C	-5.856089000	-0.476812000	-0.731809000
F	0.355260000	-2.145943000	-1.556170000
F	1.042758000	-4.359229000	-0.302167000
F	0.265980000	-4.814046000	2.260369000
F	-1.322646000	-2.998258000	3.524046000
F	-2.090948000	-0.778018000	2.256223000
F	-1.931911000	2.563437000	-1.590956000
F	-1.134035000	4.968537000	-0.775565000
F	0.260581000	5.235908000	1.546331000
F	0.843991000	3.036634000	3.020358000
F	0.188321000	0.628398000	2.176559000
F	-3.588733000	1.663141000	1.031815000
F	-6.195778000	1.283042000	0.784885000
F	-7.166347000	-0.659906000	-0.841032000
F	-5.445644000	-2.240979000	-2.231789000
F	-2.810605000	-1.880830000	-2.005590000
H	1.088814000	0.304759000	-1.566611000
O	-0.937402000	0.101678000	-1.931880000
C	0.348374000	0.446374000	-2.368088000
O	0.506887000	0.811618000	-3.480398000
H	-1.604355000	0.290076000	-2.617197000
C	4.036646000	-1.301016000	0.923215000
C	4.017570000	-2.739157000	0.379914000
C	5.432946000	-1.021028000	1.493481000
C	3.003994000	-1.283156000	2.069247000
H	3.058756000	-2.988677000	-0.078157000
H	4.808387000	-2.931335000	-0.345244000
H	4.170365000	-3.429018000	1.217323000
H	5.492494000	-0.061085000	2.007435000
H	5.679198000	-1.797175000	2.228495000
H	6.205809000	-1.040489000	0.724082000
H	3.255073000	-2.077682000	2.781415000
H	2.986602000	-0.345951000	2.621752000
H	1.994311000	-1.474962000	1.695222000
C	4.340158000	-0.503204000	-2.059858000
C	4.145030000	0.643245000	-3.067994000
C	5.843586000	-0.777999000	-1.930144000
C	3.653704000	-1.723125000	-2.708428000
H	3.089667000	0.881983000	-3.219955000
H	4.675069000	1.554890000	-2.794631000
H	4.544103000	0.316663000	-4.034983000
H	6.052429000	-1.682255000	-1.357174000
H	6.268724000	-0.924111000	-2.930716000
H	6.376784000	0.051207000	-1.463220000
H	4.123082000	-1.912280000	-3.680946000
H	3.737225000	-2.637243000	-2.124414000
H	2.592287000	-1.532414000	-2.886084000
C	3.844845000	1.680513000	0.075920000
C	3.472982000	1.902441000	1.549458000
C	5.302926000	2.119604000	-0.108060000
C	2.912130000	2.610018000	-0.729400000
H	2.476601000	1.528083000	1.779341000

H	4.178660000	1.438816000	2.238673000
H	3.477981000	2.979102000	1.752021000
H	5.609748000	2.145950000	-1.153675000
H	5.422014000	3.134662000	0.290536000
H	5.994158000	1.471139000	0.433274000
H	3.089445000	3.647545000	-0.422318000
H	3.066557000	2.557700000	-1.805911000
H	1.860751000	2.381507000	-0.527396000

D7

	Coordinates (Angstroms)		
	X	Y	Z
H	0.193417000	-0.038914000	-0.844987000
B	1.094347000	0.094387000	-0.040990000
P	-3.132244000	-0.213713000	-0.996769000
H	-1.793269000	-0.226499000	-0.577971000
C	0.607580000	-0.697289000	1.310172000
C	0.019156000	-1.946601000	1.169079000
C	0.697706000	-0.253153000	2.621375000
C	-0.492297000	-2.688069000	2.219554000
C	0.193240000	-0.956425000	3.704820000
C	-0.413900000	-2.182104000	3.503410000
C	2.401956000	-0.669002000	-0.641640000
C	2.430233000	-1.219211000	-1.912420000
C	3.551733000	-0.866894000	0.108203000
C	3.514471000	-1.923529000	-2.414266000
C	4.658145000	-1.558673000	-0.349914000
C	4.636573000	-2.094930000	-1.626198000
C	1.248901000	1.712717000	0.130862000
C	0.188663000	2.437340000	0.659872000
C	2.296222000	2.499494000	-0.325015000
C	0.136393000	3.814852000	0.734592000
C	2.296007000	3.887187000	-0.263957000
C	1.208857000	4.553409000	0.266431000
F	-0.097872000	-2.496083000	-0.055904000
F	-1.082116000	-3.869366000	2.004945000
F	-0.910485000	-2.869365000	4.532447000
F	0.292784000	-0.464382000	4.942994000
F	1.286376000	0.919427000	2.910480000
F	1.377633000	-1.090802000	-2.742662000
F	3.486995000	-2.436553000	-3.648687000
F	5.690382000	-2.767363000	-2.091464000
F	5.742221000	-1.713591000	0.416038000
F	3.632401000	-0.350191000	1.347464000
F	3.391558000	1.948814000	-0.877768000
F	3.338479000	4.585807000	-0.723025000
F	1.189693000	5.885570000	0.321943000
F	-0.937706000	4.434629000	1.234553000
F	-0.892888000	1.779844000	1.126949000
C	-4.117585000	0.063051000	0.584008000
C	-4.121194000	1.540205000	1.010609000
C	-5.574836000	-0.389306000	0.414545000
C	-3.411223000	-0.716259000	1.710985000
H	-3.117755000	1.930581000	1.170687000
H	-4.654441000	2.186519000	0.314540000
H	-4.649262000	1.589218000	1.967541000
H	-5.674220000	-1.455557000	0.212360000

H	-6.101631000	-0.181634000	1.350465000
H	-6.079327000	0.163493000	-0.381458000
H	-3.953389000	-0.521433000	2.640970000
H	-3.394960000	-1.793314000	1.560082000
H	-2.384553000	-0.366189000	1.844909000
C	-3.378870000	-1.914779000	-1.764928000
C	-4.661870000	-1.975866000	-2.603908000
C	-2.151771000	-2.240164000	-2.641013000
C	-3.433973000	-2.989600000	-0.667312000
H	-5.557595000	-1.774061000	-2.013806000
H	-4.638356000	-1.284307000	-3.446896000
H	-4.750818000	-2.987923000	-3.010125000
H	-1.211008000	-2.052112000	-2.121162000
H	-2.191803000	-3.308069000	-2.874390000
H	-2.145895000	-1.701469000	-3.584980000
H	-3.501557000	-3.958611000	-1.169776000
H	-2.527904000	-2.999672000	-0.061178000
H	-4.302751000	-2.902198000	-0.016817000
C	-3.212222000	1.204153000	-2.224998000
C	-2.529255000	0.811637000	-3.544880000
C	-4.663610000	1.612700000	-2.510175000
C	-2.403679000	2.384574000	-1.656296000
H	-1.507213000	0.458370000	-3.391383000
H	-3.091129000	0.065540000	-4.105995000
H	-2.478692000	1.713923000	-4.161285000
H	-5.177909000	1.987574000	-1.625284000
H	-4.648093000	2.417481000	-3.251142000
H	-5.248729000	0.790443000	-2.925968000
H	-2.430773000	3.186642000	-2.399763000
H	-2.795239000	2.786095000	-0.725681000
H	-1.357626000	2.105481000	-1.509342000

D8

	Coordinates (Angstroms)		
	X	Y	Z
P	3.604829000	-0.117476000	-0.411859000
C	-0.887075000	-1.156199000	0.313992000
C	-0.137479000	-2.159835000	-0.278162000
C	-1.222229000	-1.392942000	1.641951000
C	0.242677000	-3.319735000	0.378341000
C	-0.839450000	-2.518519000	2.345058000
C	-0.098550000	-3.496285000	1.703427000
C	-1.152442000	1.610999000	0.118177000
C	-1.721349000	2.738158000	-0.459415000
C	-0.190519000	1.873399000	1.081608000
C	-1.379185000	4.033501000	-0.115607000
C	0.177374000	3.152540000	1.461024000
C	-0.418032000	4.243011000	0.856283000
C	-3.159209000	-0.161231000	-0.517005000
C	-3.636026000	-1.182965000	-1.325978000
C	-4.131994000	0.469975000	0.247278000
C	-4.963173000	-1.556738000	-1.413476000
C	-5.474832000	0.127714000	0.196225000
C	-5.895541000	-0.888683000	-0.640622000
F	0.263449000	-2.062463000	-1.564810000
F	0.968333000	-4.248441000	-0.246411000
F	0.295652000	-4.583057000	2.360122000

F	-1.178699000	-2.674643000	3.624001000
F	-1.962124000	-0.489355000	2.302622000
F	-2.645712000	2.602560000	-1.425451000
F	-1.961185000	5.074780000	-0.712341000
F	-0.068473000	5.478516000	1.202517000
F	1.122249000	3.341003000	2.384588000
F	0.485216000	0.878520000	1.673416000
F	-3.815609000	1.456940000	1.094581000
F	-6.364664000	0.772157000	0.950178000
F	-7.180513000	-1.223278000	-0.700406000
F	-5.346666000	-2.544545000	-2.220511000
F	-2.765471000	-1.891328000	-2.086300000
O	-1.079491000	0.157216000	-1.966006000
C	0.147652000	0.812757000	-2.559790000
O	1.015520000	1.214397000	-1.617988000
H	-0.252941000	1.678958000	-3.080792000
H	0.531744000	0.061447000	-3.252888000
H	1.625688000	0.511965000	-1.283810000
B	-1.546939000	0.139323000	-0.437981000
H	-1.246761000	-0.681111000	-2.416829000
C	4.336963000	-1.335398000	-1.691338000
C	3.628436000	-1.060516000	-3.033801000
C	5.855807000	-1.283386000	-1.898058000
C	3.940988000	-2.773467000	-1.318491000
H	2.545872000	-1.182825000	-2.934801000
H	3.825836000	-0.070453000	-3.439492000
H	3.976207000	-1.792796000	-3.771492000
H	6.405480000	-1.455903000	-0.971686000
H	6.146261000	-2.067501000	-2.607587000
H	6.185544000	-0.331776000	-2.316748000
H	4.201231000	-3.429222000	-2.156971000
H	4.463628000	-3.152195000	-0.440796000
H	2.866096000	-2.868534000	-1.153290000
C	4.007847000	-0.762778000	1.345084000
C	5.413247000	-1.347268000	1.539066000
C	3.808810000	0.351992000	2.387022000
C	2.948561000	-1.826976000	1.687577000
H	5.583194000	-2.245002000	0.944209000
H	6.196480000	-0.626708000	1.297860000
H	5.536259000	-1.628048000	2.591923000
H	2.832697000	0.830953000	2.295371000
H	3.858037000	-0.104902000	3.381650000
H	4.583096000	1.117554000	2.347254000
H	3.116678000	-2.179954000	2.711666000
H	1.946855000	-1.393377000	1.644099000
H	2.973470000	-2.697982000	1.035323000
C	4.455965000	1.584237000	-0.631063000
C	5.894006000	1.694697000	-0.109389000
C	4.451270000	1.979836000	-2.117524000
C	3.570537000	2.634201000	0.073602000
H	5.959191000	1.560794000	0.970642000
H	6.561387000	0.971706000	-0.580551000
H	6.278623000	2.696554000	-0.335351000
H	3.452680000	1.904646000	-2.555040000
H	4.763774000	3.027247000	-2.193446000
H	5.145525000	1.392197000	-2.718447000
H	3.997848000	3.627403000	-0.107580000

H	2.555345000	2.628831000	-0.331146000
H	3.508642000	2.499792000	1.150377000

TSD2-3

	Coordinates (Angstroms)		
	X	Y	Z
B	1.074325000	0.113167000	-0.039330000
P	-3.034012000	-0.138356000	-0.946883000
C	0.579695000	-0.807817000	1.220359000
C	0.136460000	-2.101961000	0.975445000
C	0.571055000	-0.433320000	2.556475000
C	-0.346424000	-2.947348000	1.958607000
C	0.099605000	-1.250021000	3.571626000
C	-0.374534000	-2.512971000	3.270117000
C	2.405755000	-0.574764000	-0.695407000
C	2.581356000	-0.879016000	-2.034938000
C	3.473771000	-0.917577000	0.123501000
C	3.711253000	-1.505844000	-2.536484000
C	4.620527000	-1.541531000	-0.331486000
C	4.737746000	-1.844978000	-1.676509000
C	1.235349000	1.716081000	0.255349000
C	0.143489000	2.412360000	0.759683000
C	2.330300000	2.509780000	-0.053415000
C	0.104732000	3.779926000	0.939629000
C	2.342562000	3.887458000	0.123284000
C	1.224064000	4.528420000	0.618349000
F	0.152083000	-2.599232000	-0.272753000
F	-0.801947000	-4.163470000	1.650588000
F	-0.846529000	-3.303061000	4.230901000
F	0.098057000	-0.827905000	4.837213000
F	1.035611000	0.764546000	2.938908000
F	1.643256000	-0.551057000	-2.948971000
F	3.816824000	-1.777780000	-3.838330000
F	5.831942000	-2.446624000	-2.137321000
F	5.612317000	-1.846105000	0.506524000
F	3.435988000	-0.612469000	1.431023000
F	3.456377000	1.985120000	-0.558644000
F	3.425572000	4.599275000	-0.193348000
F	1.221002000	5.848154000	0.783461000
F	-0.989760000	4.378232000	1.413097000
F	-0.962832000	1.734769000	1.108007000
H	-1.193184000	-0.025453000	-0.829983000
O	-0.095756000	-0.001838000	-1.052100000
H	0.050066000	0.424462000	-1.904171000
C	-4.033658000	-0.139807000	0.676198000
C	-4.139391000	1.273667000	1.274085000
C	-5.459903000	-0.691531000	0.539671000
C	-3.238327000	-0.971220000	1.700787000
H	-3.165935000	1.739289000	1.419427000
H	-4.766700000	1.940131000	0.683510000
H	-4.607623000	1.178614000	2.259441000
H	-5.484928000	-1.742875000	0.254949000
H	-5.957207000	-0.607233000	1.512434000
H	-6.051777000	-0.125340000	-0.182121000
H	-3.771940000	-0.949754000	2.657322000
H	-3.116674000	-2.015123000	1.418920000
H	-2.247544000	-0.540622000	1.862709000

C	-3.528071000	1.358852000	-2.013567000
C	-2.964550000	1.199795000	-3.435971000
C	-5.036897000	1.614771000	-2.119820000
C	-2.822277000	2.597252000	-1.424209000
H	-1.893760000	0.976933000	-3.437242000
H	-3.481818000	0.436074000	-4.016483000
H	-3.098661000	2.152466000	-3.959015000
H	-5.489067000	1.871574000	-1.162092000
H	-5.202603000	2.461188000	-2.796060000
H	-5.571571000	0.756229000	-2.528846000
H	-3.056649000	3.460648000	-2.056291000
H	-3.130030000	2.839660000	-0.410128000
H	-1.735933000	2.469183000	-1.428457000
C	-3.349586000	-1.757712000	-1.900620000
C	-3.244675000	-2.954957000	-0.940626000
C	-4.692326000	-1.828628000	-2.638114000
C	-2.200271000	-1.940391000	-2.913669000
H	-2.301891000	-2.955339000	-0.391603000
H	-4.066488000	-3.012292000	-0.228545000
H	-3.270094000	-3.870825000	-1.540442000
H	-4.761918000	-1.091046000	-3.438478000
H	-4.788886000	-2.818519000	-3.098294000
H	-5.543602000	-1.689386000	-1.970569000
H	-2.322438000	-2.916138000	-3.395991000
H	-2.192856000	-1.189663000	-3.700806000
H	-1.226312000	-1.935990000	-2.419574000

TSD3-4

	Coordinates (Angstroms)		
	X	Y	Z
B	-1.400140000	0.083148000	-0.382497000
P	3.451573000	-0.156483000	-0.530084000
C	-1.076499000	-1.357255000	0.321946000
C	-0.263183000	-2.352767000	-0.196535000
C	-1.717447000	-1.692579000	1.508954000
C	-0.089240000	-3.587723000	0.410642000
C	-1.565607000	-2.905941000	2.151794000
C	-0.737639000	-3.866372000	1.596082000
C	-0.768521000	1.399981000	0.377316000
C	-0.837802000	2.628598000	-0.272980000
C	-0.125355000	1.440899000	1.604548000
C	-0.308488000	3.804621000	0.221627000
C	0.415945000	2.599793000	2.144136000
C	0.335739000	3.787110000	1.445711000
C	-3.026461000	0.167491000	-0.610787000
C	-3.640101000	-0.743426000	-1.465369000
C	-3.894825000	1.046347000	0.023034000
C	-5.003507000	-0.777385000	-1.701481000
C	-5.265189000	1.044385000	-0.184801000
C	-5.824190000	0.128724000	-1.055926000
F	0.449274000	-2.166208000	-1.321028000
F	0.734001000	-4.493864000	-0.124116000
F	-0.565998000	-5.039281000	2.201119000
F	-2.201574000	-3.159385000	3.296793000
F	-2.525164000	-0.798371000	2.101903000
F	-1.473029000	2.717688000	-1.458413000
F	-0.403552000	4.942521000	-0.465533000

F	0.884565000	4.894710000	1.935587000
F	1.048074000	2.567749000	3.319032000
F	0.020370000	0.337447000	2.355152000
F	-3.444624000	1.949976000	0.905651000
F	-6.049622000	1.914883000	0.453101000
F	-7.137680000	0.115426000	-1.266535000
F	-5.530129000	-1.673036000	-2.538174000
F	-2.905383000	-1.663895000	-2.110716000
H	1.829204000	0.146666000	-1.190261000
O	-0.781760000	0.083610000	-1.790978000
C	0.866785000	0.573103000	-2.092464000
O	0.920012000	1.128857000	-3.104944000
C	3.675591000	-0.776563000	1.253661000
C	2.604676000	-1.849198000	1.517700000
C	5.075264000	-1.336901000	1.541590000
C	3.398877000	0.350114000	2.259249000
H	1.604579000	-1.417862000	1.449507000
H	2.661297000	-2.702059000	0.845128000
H	2.734562000	-2.224517000	2.538385000
H	5.861070000	-0.608650000	1.334524000
H	5.129947000	-1.589004000	2.606363000
H	5.294138000	-2.245707000	0.982190000
H	3.354981000	-0.095168000	3.258171000
H	4.176965000	1.112060000	2.274471000
H	2.438954000	0.828467000	2.078280000
C	4.228890000	1.568993000	-0.725936000
C	5.619175000	1.730209000	-0.099875000
C	4.323043000	1.942352000	-2.215291000
C	3.247375000	2.579383000	-0.093902000
H	5.614333000	1.580151000	0.979727000
H	6.348886000	1.046463000	-0.536970000
H	5.967868000	2.752118000	-0.285828000
H	3.361408000	1.864881000	-2.726432000
H	4.640935000	2.988125000	-2.278108000
H	5.058308000	1.348449000	-2.757348000
H	3.585429000	3.591796000	-0.338565000
H	2.235385000	2.468264000	-0.494979000
H	3.194368000	2.509546000	0.990249000
C	4.223838000	-1.394976000	-1.750687000
C	3.588300000	-1.149314000	-3.133335000
C	5.751262000	-1.306053000	-1.858710000
C	3.836798000	-2.835395000	-1.373505000
H	2.512149000	-1.337603000	-3.111758000
H	3.757691000	-0.149521000	-3.526977000
H	4.027365000	-1.859731000	-3.841675000
H	6.245353000	-1.457550000	-0.898150000
H	6.097364000	-2.092140000	-2.538964000
H	6.085039000	-0.352679000	-2.269471000
H	4.169611000	-3.490826000	-2.185144000
H	4.314266000	-3.188024000	-0.460154000
H	2.756755000	-2.958007000	-1.278643000
H	-1.317893000	0.585047000	-2.419296000

TSD1-7

	Coordinates (Angstroms)		
	X	Y	Z
H	3.558238000	-3.659514000	-1.685330000

H	4.579234000	-2.501034000	-3.557490000
H	2.145277000	-3.000949000	-3.351879000
F	1.571641000	-3.814210000	1.664937000
C	3.499314000	-2.713666000	-1.135532000
H	4.437766000	-2.596999000	-0.594157000
H	2.688145000	-2.808633000	-0.411328000
F	-5.191361000	-2.989196000	-2.561970000
C	4.418765000	-1.509241000	-3.117195000
H	5.345972000	-1.212602000	-2.625235000
F	-4.671061000	-3.680479000	0.009188000
C	1.971170000	-2.010167000	-2.916512000
H	5.727883000	-1.082042000	-0.436235000
C	3.235969000	-1.582551000	-2.143381000
F	0.001068000	-2.489477000	-0.039765000
H	4.235533000	-0.817813000	-3.940980000
C	0.973129000	-2.689549000	2.052826000
H	1.106783000	-2.086389000	-2.252698000
F	1.966391000	-2.868376000	4.172641000
C	-4.306833000	-2.298153000	-1.854714000
C	-4.036363000	-2.649878000	-0.544982000
H	3.679574000	-1.647048000	1.166990000
H	1.715613000	-1.338876000	-3.733472000
C	0.164430000	-1.979588000	1.185895000
H	6.227282000	0.224207000	0.631124000
C	1.177684000	-2.209971000	3.334010000
F	-3.910951000	-0.874761000	-3.683926000
C	5.570754000	-0.027114000	-0.210807000
C	-3.652960000	-1.218546000	-2.424359000
F	-2.902841000	-2.299334000	1.444461000
C	-3.107583000	-1.921129000	0.177787000
H	5.900435000	0.557429000	-1.071678000
C	3.693933000	-0.578958000	1.378234000
H	4.396876000	-0.416492000	2.203641000
H	2.623015000	0.431407000	-4.320094000
C	-2.744752000	-0.504326000	-1.666661000
C	-2.426345000	-0.832423000	-0.353001000
P	2.802898000	0.018194000	-1.186781000
C	4.120664000	0.289218000	0.177424000
C	-0.461624000	-0.785611000	1.533648000
H	2.697804000	-0.288373000	1.724041000
C	0.560650000	-1.038170000	3.732231000
H	4.871167000	1.166057000	-3.267676000
H	0.479924000	0.045591000	-0.829203000
H	-0.287043000	0.125531000	-0.934341000
F	-2.151138000	0.541942000	-2.256615000
C	-0.242575000	-0.353293000	2.837281000
B	-1.379014000	-0.003096000	0.506107000
F	0.743481000	-0.580169000	4.968763000
C	2.100170000	1.145511000	-3.683858000
H	1.100971000	0.758315000	-3.465760000
C	4.282633000	1.963807000	-2.812200000
C	2.881393000	1.490299000	-2.404200000
H	1.974998000	2.065926000	-4.265086000
H	4.850320000	2.365231000	-1.972476000
C	4.077410000	1.738038000	0.694315000
H	4.186404000	2.767358000	-3.552690000
F	-0.827324000	0.759405000	3.293297000

H	4.713832000	1.797051000	1.584536000
H	4.462024000	2.461612000	-0.024023000
H	3.072483000	2.039835000	0.992302000
C	-1.545135000	1.569457000	0.612066000
F	0.746343000	1.856553000	1.097681000
F	-3.883801000	1.537125000	0.212943000
H	1.067263000	2.377237000	-1.567392000
C	2.106019000	2.658943000	-1.761907000
H	2.540823000	3.018932000	-0.832650000
C	-0.454601000	2.395354000	0.871468000
C	-2.757922000	2.224841000	0.426140000
H	2.093984000	3.498091000	-2.467162000
C	-0.532275000	3.772926000	0.895488000
C	-2.879320000	3.604209000	0.463534000
F	0.553243000	4.512125000	1.113330000
F	-4.063303000	4.185123000	0.283527000
C	-1.758650000	4.382371000	0.690506000
F	-1.858598000	5.704140000	0.717764000

TSD7

	Coordinates (Angstroms)		
	X	Y	Z
H	0.635930000	0.015537000	-1.056194000
B	1.463746000	-0.114320000	-0.028111000
P	-3.663147000	0.150867000	-0.736099000
H	-1.912639000	-0.658860000	-1.344344000
C	0.623916000	-1.133934000	0.895038000
C	0.435072000	-2.450537000	0.482970000
C	-0.041177000	-0.796911000	2.067328000
C	-0.346442000	-3.367037000	1.158203000
C	-0.823592000	-1.690808000	2.783271000
C	-0.986367000	-2.981214000	2.321376000
C	2.836527000	-0.663244000	-0.690583000
C	3.302612000	-0.118332000	-1.878319000
C	3.674409000	-1.605607000	-0.109723000
C	4.495932000	-0.478300000	-2.477823000
C	4.880310000	-1.992227000	-0.671062000
C	5.293692000	-1.427093000	-1.864080000
C	1.713905000	1.384985000	0.533215000
C	1.275814000	2.556261000	-0.057789000
C	2.492618000	1.554238000	1.670724000
C	1.540045000	3.814854000	0.460367000
C	2.787267000	2.787869000	2.218449000
C	2.302077000	3.931642000	1.606277000
F	1.013502000	-2.886567000	-0.646689000
F	-0.517310000	-4.601667000	0.684404000
F	-1.780122000	-3.830699000	2.965903000
F	-1.460267000	-1.299631000	3.888885000
F	0.017518000	0.445389000	2.568537000
F	2.569956000	0.821892000	-2.512284000
F	4.881678000	0.077396000	-3.626376000
F	6.448105000	-1.791224000	-2.414977000
F	5.646792000	-2.903471000	-0.071926000
F	3.344543000	-2.192739000	1.047973000
F	2.975297000	0.473279000	2.303224000
F	3.526995000	2.889775000	3.323247000
F	2.571194000	5.130860000	2.116615000

F	1.068581000	4.908064000	-0.141683000
F	0.546641000	2.524320000	-1.184808000
C	-0.136260000	-0.488505000	-2.164166000
O	-1.085734000	-1.205643000	-1.663759000
O	-0.511267000	0.630534000	-2.776937000
H	0.687048000	-1.053543000	-2.600544000
H	0.259725000	1.092742000	-3.131718000
C	-3.507053000	1.468899000	0.633907000
C	-2.195820000	2.229549000	0.359059000
C	-4.680392000	2.447820000	0.755331000
C	-3.309556000	0.793984000	1.998145000
H	-1.350338000	1.536925000	0.334532000
H	-2.201217000	2.790608000	-0.572644000
H	-2.018616000	2.940726000	1.173869000
H	-5.622820000	1.933051000	0.952394000
H	-4.488488000	3.122694000	1.597635000
H	-4.809476000	3.067198000	-0.131887000
H	-3.014603000	1.560211000	2.722269000
H	-4.212230000	0.316392000	2.377637000
H	-2.511492000	0.054665000	1.965343000
C	-4.807112000	-1.253463000	-0.141532000
C	-6.092930000	-0.808062000	0.565481000
C	-5.190699000	-2.144450000	-1.334439000
C	-3.980123000	-2.156008000	0.797405000
H	-5.896900000	-0.271357000	1.494116000
H	-6.712632000	-0.172604000	-0.069046000
H	-6.681866000	-1.696543000	0.822263000
H	-4.313403000	-2.475309000	-1.897412000
H	-5.686089000	-3.039831000	-0.944008000
H	-5.888232000	-1.661987000	-2.018819000
H	-4.584746000	-3.029430000	1.065991000
H	-3.077411000	-2.521494000	0.299338000
H	-3.686295000	-1.668846000	1.724831000
C	-4.398057000	0.959638000	-2.300444000
C	-5.906311000	1.232441000	-2.264082000
C	-3.661185000	2.280153000	-2.588915000
C	-4.072099000	0.043135000	-3.497361000
H	-6.491027000	0.312716000	-2.216155000
H	-6.194567000	1.865286000	-1.423554000
H	-6.194186000	1.752662000	-3.185276000
H	-2.578433000	2.140514000	-2.618956000
H	-3.975817000	2.633995000	-3.576885000
H	-3.896305000	3.069454000	-1.875839000
H	-4.389380000	0.548487000	-4.416417000
H	-2.997618000	-0.141361000	-3.578029000
H	-4.583459000	-0.916425000	-3.462653000

TSD8

	Coordinates (Angstroms)		
	X	Y	Z
B	-1.524827000	0.123119000	-0.498695000
P	3.714047000	-0.137692000	-0.450742000
C	-1.038593000	-1.249286000	0.251289000
C	-0.376073000	-2.319453000	-0.331356000
C	-1.436093000	-1.464997000	1.566162000
C	-0.106707000	-3.505193000	0.337583000
C	-1.163402000	-2.616770000	2.278628000

C	-0.486102000	-3.650263000	1.655555000
C	-0.993576000	1.539742000	0.150511000
C	-1.320197000	2.715026000	-0.512261000
C	-0.154628000	1.715944000	1.236951000
C	-0.867428000	3.968700000	-0.152684000
C	0.316755000	2.954540000	1.646532000
C	-0.032931000	4.089827000	0.943556000
C	-3.171080000	0.020511000	-0.576521000
C	-3.760885000	-0.967929000	-1.359531000
C	-4.068364000	0.799034000	0.144117000
C	-5.128355000	-1.160826000	-1.454623000
C	-5.443580000	0.636146000	0.078367000
C	-5.979361000	-0.347714000	-0.729848000
F	0.065713000	-2.271818000	-1.599205000
F	0.543206000	-4.497435000	-0.276233000
F	-0.199094000	-4.767049000	2.321745000
F	-1.551072000	-2.746062000	3.548781000
F	-2.129437000	-0.512875000	2.211341000
F	-2.125195000	2.665259000	-1.597321000
F	-1.221104000	5.053104000	-0.845798000
F	0.427724000	5.282960000	1.315135000
F	1.126125000	3.060282000	2.703900000
F	0.293017000	0.668572000	1.951144000
F	-3.644260000	1.764893000	0.971806000
F	-6.253981000	1.420773000	0.791589000
F	-7.297402000	-0.515111000	-0.805001000
F	-5.629906000	-2.124281000	-2.230132000
F	-2.999234000	-1.814595000	-2.070342000
H	2.226936000	0.309025000	-0.994502000
O	-1.043350000	0.113817000	-1.957667000
C	0.568310000	0.515095000	-2.472512000
O	1.269945000	1.002703000	-1.523624000
H	0.252412000	1.214631000	-3.253946000
H	0.807321000	-0.483166000	-2.843880000
H	-1.636636000	0.653219000	-2.491130000
C	3.738576000	-1.742882000	0.553319000
C	3.445882000	-2.921913000	-0.387671000
C	5.059329000	-2.009900000	1.286455000
C	2.583769000	-1.699438000	1.574964000
H	2.536956000	-2.763782000	-0.971767000
H	4.272152000	-3.134395000	-1.066235000
H	3.291961000	-3.814469000	0.225836000
H	5.283889000	-1.246532000	2.032462000
H	4.971347000	-2.966831000	1.812666000
H	5.906691000	-2.083890000	0.603692000
H	2.413607000	-2.717187000	1.940408000
H	2.804435000	-1.079068000	2.439779000
H	1.653888000	-1.343692000	1.129251000
C	4.728081000	-0.296688000	-2.045800000
C	4.897319000	1.095294000	-2.678464000
C	6.105876000	-0.940892000	-1.853479000
C	3.916776000	-1.116732000	-3.068001000
H	3.936686000	1.599608000	-2.810644000
H	5.563362000	1.745938000	-2.114070000
H	5.339209000	0.960486000	-3.670917000
H	6.028054000	-1.988484000	-1.558042000
H	6.642058000	-0.908898000	-2.808144000

H	6.712679000	-0.417866000	-1.112987000
H	4.484071000	-1.146060000	-4.004270000
H	3.735012000	-2.145445000	-2.766908000
H	2.956536000	-0.644104000	-3.284528000
C	4.320981000	1.329331000	0.592167000
C	3.758295000	1.225079000	2.017606000
C	5.847760000	1.439359000	0.690217000
C	3.729362000	2.619755000	-0.010261000
H	2.678500000	1.080504000	2.028669000
H	4.228904000	0.430606000	2.595942000
H	3.969519000	2.168976000	2.529114000
H	6.321619000	1.645816000	-0.269479000
H	6.091470000	2.270853000	1.360547000
H	6.296792000	0.535792000	1.107651000
H	3.960992000	3.449221000	0.665985000
H	4.142956000	2.866823000	-0.986159000
H	2.643093000	2.555389000	-0.107928000

TSD9

	Coordinates (Angstroms)		
	X	Y	Z
H	0.614031000	-0.025882000	-1.071604000
B	1.459066000	-0.109750000	-0.045805000
P	-3.648195000	0.157537000	-0.797228000
H	-2.223891000	-0.572791000	-1.134837000
C	0.626276000	-1.116432000	0.888124000
C	0.404629000	-2.420385000	0.450428000
C	-0.025387000	-0.786236000	2.069695000
C	-0.400706000	-3.328404000	1.111073000
C	-0.824461000	-1.674421000	2.772921000
C	-1.027408000	-2.949308000	2.282300000
C	2.832343000	-0.663725000	-0.695448000
C	3.290668000	-0.158354000	-1.904137000
C	3.670410000	-1.586106000	-0.084980000
C	4.481314000	-0.545147000	-2.493541000
C	4.872350000	-1.996693000	-0.637565000
C	5.279830000	-1.474760000	-1.851609000
C	1.667659000	1.417381000	0.450986000
C	1.197169000	2.548212000	-0.194552000
C	2.435264000	1.660785000	1.582119000
C	1.416038000	3.835030000	0.272717000
C	2.685947000	2.925647000	2.078726000
C	2.164749000	4.025516000	1.417816000
F	0.986395000	-2.857516000	-0.674114000
F	-0.607638000	-4.548199000	0.612048000
F	-1.843009000	-3.790175000	2.912845000
F	-1.441585000	-1.291616000	3.893928000
F	0.064314000	0.445403000	2.594273000
F	2.559186000	0.757939000	-2.562125000
F	4.865938000	-0.031254000	-3.663018000
F	6.431002000	-1.861134000	-2.396407000
F	5.640242000	-2.888996000	-0.010031000
F	3.344064000	-2.130573000	1.095648000
F	2.954244000	0.624319000	2.258883000
F	3.417327000	3.098855000	3.180553000
F	2.388086000	5.253636000	1.879955000
F	0.910253000	4.886069000	-0.375928000

F	0.478108000	2.450221000	-1.325329000
C	-0.295263000	-0.438747000	-2.019921000
O	-1.177379000	-1.223410000	-1.530419000
H	0.451572000	-0.874648000	-2.689818000
H	-0.581627000	0.593635000	-2.257992000
C	-4.171986000	1.039395000	-2.390547000
C	-3.797468000	0.140131000	-3.585547000
C	-5.665483000	1.382412000	-2.446147000
C	-3.353295000	2.329508000	-2.564938000
H	-2.737791000	-0.127045000	-3.574037000
H	-4.379356000	-0.776713000	-3.638866000
H	-3.988112000	0.701342000	-4.506073000
H	-5.975595000	2.017793000	-1.614941000
H	-5.864424000	1.927058000	-3.375503000
H	-6.293757000	0.490428000	-2.450020000
H	-3.573547000	2.733066000	-3.558192000
H	-3.607213000	3.100867000	-1.839557000
H	-2.277307000	2.142801000	-2.519669000
C	-3.459570000	1.385195000	0.632791000
C	-4.586601000	2.419537000	0.731113000
C	-3.375753000	0.634111000	1.968692000
C	-2.094252000	2.075286000	0.454102000
H	-4.628100000	3.086328000	-0.129947000
H	-5.563898000	1.947104000	0.849735000
H	-4.407373000	3.039517000	1.616101000
H	-2.613720000	-0.141762000	1.940355000
H	-3.085491000	1.351557000	2.741883000
H	-4.321954000	0.187648000	2.272411000
H	-1.904183000	2.701743000	1.331729000
H	-1.293064000	1.334176000	0.399945000
H	-2.035823000	2.713718000	-0.423942000
C	-4.822982000	-1.260823000	-0.353953000
C	-4.064069000	-2.220351000	0.586534000
C	-6.138264000	-0.807663000	0.290787000
C	-5.147143000	-2.076857000	-1.616113000
H	-3.118450000	-2.546915000	0.144390000
H	-3.861690000	-1.799077000	1.568414000
H	-4.682158000	-3.111372000	0.736361000
H	-6.713230000	-0.153543000	-0.367012000
H	-6.748977000	-1.693915000	0.495028000
H	-5.987423000	-0.290389000	1.238642000
H	-5.691311000	-2.973627000	-1.303182000
H	-5.785760000	-1.537656000	-2.315596000
H	-4.243436000	-2.405680000	-2.135579000

E1(B(C_6F_5)$_3$)

	Coordinates (Angstroms)		
	X	Y	Z
B	0.002446000	-0.002949000	0.002781000
C	1.542451000	-0.285786000	0.001187000
C	2.106690000	-1.300693000	0.770109000
C	2.426820000	0.464051000	-0.770015000
C	3.465080000	-1.556164000	0.786660000
C	3.787538000	0.221910000	-0.791315000
C	4.307077000	-0.791593000	-0.003572000
C	-1.015617000	-1.192980000	0.005205000
C	-2.176318000	-1.168141000	0.774444000

C	-0.814743000	-2.335540000	-0.765445000
C	-3.082683000	-2.211753000	0.790737000
C	-1.710670000	-3.388141000	-0.786451000
C	-2.848503000	-3.324654000	0.000496000
C	-0.521403000	1.473294000	0.000526000
C	0.064055000	2.463263000	0.785075000
C	-1.600053000	1.870101000	-0.785281000
C	-0.392972000	3.767708000	0.803485000
C	-2.069216000	3.170117000	-0.805159000
C	-1.463269000	4.121221000	-0.001333000
F	1.336204000	-2.058427000	1.554708000
F	3.968575000	-2.523020000	1.548435000
F	5.609822000	-1.030106000	-0.006316000
F	4.598212000	0.947903000	-1.555570000
F	1.972468000	1.444914000	-1.553943000
F	-2.441155000	-0.121513000	1.560564000
F	-4.171709000	-2.157253000	1.552122000
F	-3.712717000	-4.328167000	-0.002281000
F	-1.492476000	-4.454856000	-1.550294000
F	0.261907000	-2.438873000	-1.548975000
F	-2.209054000	0.991362000	-1.585863000
F	-3.090899000	3.514698000	-1.583722000
F	-1.907221000	5.369109000	-0.002437000
F	0.181402000	4.680799000	1.581129000
F	1.092436000	2.167590000	1.584663000

E1(PPh$_3$)

	Coordinates (Angstroms)		
	X	Y	Z
P	-0.012766000	0.011987000	-1.241735000
C	0.464220000	-1.579085000	-0.446125000
C	1.560609000	-2.248798000	-0.998539000
C	-0.184547000	-2.140323000	0.654840000
C	2.013503000	-3.441208000	-0.449370000
H	2.067322000	-1.830268000	-1.863192000
C	0.261862000	-3.342014000	1.196558000
H	-1.039560000	-1.640599000	1.096759000
C	1.362475000	-3.991813000	0.650014000
H	2.869915000	-3.944280000	-0.884722000
H	-0.250830000	-3.768222000	2.052076000
H	1.710460000	-4.925826000	1.077322000
C	-1.620564000	0.389479000	-0.427727000
C	-1.758368000	1.217500000	0.687319000
C	-2.765102000	-0.191526000	-0.983167000
C	-3.013337000	1.450937000	1.240557000
H	-0.885489000	1.683604000	1.130399000
C	-4.016376000	0.031623000	-0.422520000
H	-2.676375000	-0.827082000	-1.859372000
C	-4.143102000	0.856831000	0.690545000
H	-3.105672000	2.099083000	2.105299000
H	-4.893819000	-0.430405000	-0.861506000
H	-5.119942000	1.038294000	1.125147000
C	1.129769000	1.198559000	-0.417859000
C	1.300341000	2.444625000	-1.027650000
C	1.818289000	0.926872000	0.766474000
C	2.128582000	3.404824000	-0.458466000
H	0.781808000	2.665325000	-1.955940000

C	2.654047000	1.884390000	1.330673000
H	1.703699000	-0.034992000	1.253909000
C	2.808933000	3.125097000	0.721885000
H	2.249961000	4.367807000	-0.942151000
H	3.183415000	1.660110000	2.250428000
H	3.462672000	3.869732000	1.162682000

E1_CO

	Coordinates (Angstroms)		
	X	Y	Z
B	0.016678000	-0.009050000	0.618236000
C	1.592157000	0.115637000	0.232810000
C	2.545413000	-0.659133000	0.878312000
C	2.074088000	0.938702000	-0.775708000
C	3.892227000	-0.631979000	0.572387000
C	3.416854000	0.995297000	-1.113156000
C	4.330685000	0.209638000	-0.434869000
C	-0.680186000	-1.431291000	0.241872000
C	-1.851275000	-1.823058000	0.874677000
C	-0.218766000	-2.288867000	-0.747109000
C	-2.537579000	-2.982412000	0.570842000
C	-0.877128000	-3.461863000	-1.081128000
C	-2.039937000	-3.811684000	-0.418833000
C	-0.880486000	1.295746000	0.242726000
C	-0.695910000	2.499699000	0.906208000
C	-1.819100000	1.314330000	-0.779663000
C	-1.388767000	3.656332000	0.605154000
C	-2.535170000	2.452372000	-1.113144000
C	-2.321704000	3.628034000	-0.416415000
F	2.151007000	-1.500345000	1.852883000
F	4.759606000	-1.398821000	1.227903000
F	5.618735000	0.258304000	-0.751397000
F	3.833000000	1.797221000	-2.090716000
F	1.248910000	1.703664000	-1.497507000
F	-2.369624000	-1.033202000	1.833936000
F	-3.657198000	-3.304681000	1.212994000
F	-2.676934000	-4.932640000	-0.733926000
F	-0.402079000	-4.250696000	-2.042353000
F	0.877524000	-2.000376000	-1.455563000
F	-2.053069000	0.226555000	-1.521211000
F	-3.422722000	2.425170000	-2.104808000
F	-3.003690000	4.723146000	-0.728409000
F	-1.170001000	4.781654000	1.280224000
F	0.214231000	2.565393000	1.896397000
C	0.017191000	-0.021866000	2.254331000
O	0.013061000	-0.044953000	3.370391000

E1-2

	Coordinates (Angstroms)		
	X	Y	Z
B	-0.259415000	0.090517000	-0.488016000
P	0.646233000	-0.011127000	1.445380000
C	0.403097000	-1.702887000	2.111288000
C	-0.872463000	-2.036603000	2.577763000
C	1.415138000	-2.658993000	2.166911000
C	-1.135475000	-3.311677000	3.054881000

H	-1.661085000	-1.296715000	2.606812000
C	1.148129000	-3.935606000	2.648305000
H	2.420749000	-2.426789000	1.846075000
C	-0.127361000	-4.268939000	3.082416000
H	-2.129898000	-3.552319000	3.412934000
H	1.945409000	-4.669142000	2.682647000
H	-0.333051000	-5.266424000	3.454028000
C	0.082566000	1.032627000	2.845674000
C	-0.609998000	2.230278000	2.678957000
C	0.436333000	0.622229000	4.135117000
C	-0.940467000	3.006748000	3.782344000
H	-0.928304000	2.556224000	1.699649000
C	0.104521000	1.400684000	5.234858000
H	0.975734000	-0.305845000	4.286888000
C	-0.582065000	2.596863000	5.060257000
H	-1.483059000	3.933823000	3.637702000
H	0.384157000	1.070090000	6.228596000
H	-0.842597000	3.204607000	5.919599000
C	2.461720000	0.287913000	1.400131000
C	3.323839000	-0.511434000	0.643562000
C	3.003448000	1.357537000	2.118972000
C	4.686583000	-0.246846000	0.604619000
H	2.952491000	-1.359616000	0.089227000
C	4.366058000	1.619888000	2.077233000
H	2.366526000	2.004168000	2.707609000
C	5.212351000	0.821514000	1.318228000
H	5.332498000	-0.881701000	0.008936000
H	4.762754000	2.458259000	2.638158000
H	6.275657000	1.030237000	1.283627000
C	0.870711000	-0.711287000	-1.363706000
C	1.002799000	-2.100102000	-1.324385000
C	1.816661000	-0.075547000	-2.162909000
C	1.972123000	-2.806475000	-2.011675000
C	2.802181000	-0.748324000	-2.870472000
C	2.885214000	-2.123427000	-2.794736000
C	-1.722201000	-0.666881000	-0.442714000
C	-2.690886000	-0.332586000	0.491073000
C	-2.172934000	-1.575038000	-1.400568000
C	-3.950765000	-0.901561000	0.566230000
C	-3.422689000	-2.168254000	-1.371521000
C	-4.321109000	-1.840664000	-0.373233000
C	-0.502051000	1.634578000	-0.995529000
C	0.180363000	2.753485000	-0.538181000
C	-1.414026000	1.915947000	-2.009923000
C	-0.052693000	4.047039000	-0.966448000
C	-1.676835000	3.194340000	-2.477845000
C	-0.999054000	4.273607000	-1.947100000
F	0.165356000	-2.844707000	-0.589748000
F	2.033178000	-4.135029000	-1.924778000
F	3.829195000	-2.781453000	-3.458932000
F	3.682759000	-0.071117000	-3.607007000
F	1.856345000	1.257675000	-2.277539000
F	-2.423766000	0.593909000	1.427095000
F	-4.795335000	-0.548105000	1.536009000
F	-5.523536000	-2.406227000	-0.332093000
F	-3.777391000	-3.037557000	-2.319253000
F	-1.425847000	-1.893025000	-2.466077000

F	-2.083488000	0.936683000	-2.634089000
F	-2.572222000	3.385099000	-3.447246000
F	-1.240666000	5.506058000	-2.382841000
F	0.635929000	5.066067000	-0.452216000
F	1.159600000	2.614237000	0.369783000

E2

	Coordinates (Angstroms)		
	X	Y	Z
B	1.446593000	-0.068452000	-0.250268000
P	-2.547830000	0.097372000	-0.874456000
C	-3.569883000	-1.333574000	-1.375926000
C	-2.931997000	-2.560785000	-1.560895000
C	-4.953505000	-1.247855000	-1.547861000
C	-3.668331000	-3.693476000	-1.890779000
H	-1.856839000	-2.636020000	-1.449141000
C	-5.685626000	-2.377723000	-1.887710000
H	-5.461234000	-0.298760000	-1.415557000
C	-5.045128000	-3.602241000	-2.054749000
H	-3.163114000	-4.643244000	-2.024727000
H	-6.758818000	-2.301902000	-2.022555000
H	-5.619756000	-4.483577000	-2.317590000
C	-3.416994000	1.537519000	-1.596447000
C	-3.079427000	1.896215000	-2.903643000
C	-4.397219000	2.264542000	-0.919256000
C	-3.720921000	2.957629000	-3.529413000
H	-2.311468000	1.343084000	-3.435938000
C	-5.031541000	3.331905000	-1.544543000
H	-4.665377000	2.002683000	0.098130000
C	-4.697282000	3.677639000	-2.849139000
H	-3.454226000	3.225727000	-4.545574000
H	-5.790535000	3.892720000	-1.010424000
H	-5.193091000	4.511482000	-3.333612000
C	-2.840395000	0.253258000	0.925138000
C	-3.548667000	-0.693118000	1.663500000
C	-2.270052000	1.347956000	1.583382000
C	-3.699092000	-0.537300000	3.038404000
H	-3.985537000	-1.556292000	1.174350000
C	-2.431257000	1.505827000	2.951804000
H	-1.705336000	2.085280000	1.022268000
C	-3.148841000	0.563060000	3.681889000
H	-4.246178000	-1.282409000	3.604819000
H	-1.984413000	2.358377000	3.450041000
H	-3.266378000	0.681362000	4.752924000
C	0.645253000	-0.973843000	0.840469000
C	0.163610000	-2.224584000	0.467092000
C	0.365594000	-0.612952000	2.151619000
C	-0.588010000	-3.039518000	1.292051000
C	-0.345927000	-1.421135000	3.025526000
C	-0.846733000	-2.630418000	2.587985000
C	2.815786000	-0.744772000	-0.830510000
C	3.349880000	-0.287367000	-2.025853000
C	3.546449000	-1.746504000	-0.208575000
C	4.508761000	-0.780061000	-2.596037000
C	4.713342000	-2.270810000	-0.741760000
C	5.196033000	-1.788358000	-1.944431000
C	1.731797000	1.472377000	0.212883000

C	0.965266000	2.572665000	-0.137390000
C	2.813007000	1.760018000	1.037955000
C	1.230890000	3.863439000	0.291203000
C	3.120860000	3.031740000	1.483380000
C	2.322088000	4.096785000	1.104563000
F	0.420578000	-2.700010000	-0.763773000
F	-1.055298000	-4.209813000	0.855908000
F	-1.571149000	-3.391665000	3.401725000
F	-0.560878000	-1.031867000	4.281534000
F	0.777613000	0.556502000	2.658687000
F	2.724596000	0.708363000	-2.695153000
F	4.965018000	-0.298183000	-3.751559000
F	6.313713000	-2.284287000	-2.467005000
F	5.377860000	-3.233475000	-0.102424000
F	3.161953000	-2.248240000	0.972419000
F	3.595058000	0.765997000	1.484998000
F	4.167710000	3.240000000	2.281566000
F	2.597648000	5.327125000	1.526600000
F	0.440624000	4.873188000	-0.073081000
F	-0.121636000	2.438750000	-0.925470000
H	-0.529086000	0.002693000	-1.301027000
O	0.468242000	-0.072583000	-1.500594000
H	0.698507000	0.552595000	-2.201767000

E3

	Coordinates (Angstroms)		
	X	Y	Z
B	1.402984000	0.159192000	-0.327799000
P	-2.723928000	-0.107419000	-0.766093000
C	-3.188903000	-1.257656000	-2.064795000
C	-2.166659000	-1.933585000	-2.733137000
C	-4.528486000	-1.484887000	-2.386247000
C	-2.494890000	-2.848747000	-3.726584000
H	-1.128658000	-1.743312000	-2.480506000
C	-4.842935000	-2.400600000	-3.378833000
H	-5.317546000	-0.951320000	-1.867136000
C	-3.827136000	-3.082028000	-4.045336000
H	-1.706572000	-3.377182000	-4.249660000
H	-5.880307000	-2.583258000	-3.633386000
H	-4.078766000	-3.797472000	-4.820030000
C	-3.510514000	1.495568000	-0.970947000
C	-2.793127000	2.510668000	-1.605558000
C	-4.812987000	1.717331000	-0.522309000
C	-3.385478000	3.752466000	-1.790030000
H	-1.776979000	2.335858000	-1.941781000
C	-5.397464000	2.961254000	-0.715335000
H	-5.361136000	0.930882000	-0.015188000
C	-4.684442000	3.975677000	-1.346578000
H	-2.829240000	4.547612000	-2.272087000
H	-6.407633000	3.140576000	-0.366511000
H	-5.142657000	4.947941000	-1.488123000
C	-3.136396000	-0.783644000	0.854091000
C	-3.511805000	-2.120111000	0.990253000
C	-3.047437000	0.044761000	1.975994000
C	-3.805633000	-2.623692000	2.250741000
H	-3.566853000	-2.769034000	0.123877000
C	-3.341618000	-0.469583000	3.230427000

H	-2.753568000	1.083077000	1.874884000
C	-3.724096000	-1.799908000	3.367078000
H	-4.087537000	-3.663985000	2.359718000
H	-3.266864000	0.169689000	4.101991000
H	-3.946972000	-2.199176000	4.349597000
C	0.736158000	-0.696053000	0.925882000
C	0.258893000	-1.979914000	0.686291000
C	0.636087000	-0.287605000	2.248850000
C	-0.305138000	-2.788821000	1.658673000
C	0.077461000	-1.063679000	3.251638000
C	-0.393697000	-2.327698000	2.957100000
C	2.836291000	-0.598515000	-0.657151000
C	3.173062000	-1.168801000	-1.873760000
C	3.790172000	-0.758181000	0.338965000
C	4.355077000	-1.863136000	-2.091104000
C	4.981468000	-1.437974000	0.169333000
C	5.265081000	-2.002655000	-1.062215000
C	1.591533000	1.779146000	-0.023675000
C	0.480695000	2.520378000	0.363290000
C	2.719100000	2.543917000	-0.279387000
C	0.462775000	3.894826000	0.494535000
C	2.754437000	3.927235000	-0.153094000
C	1.619434000	4.609659000	0.234908000
F	0.330093000	-2.522234000	-0.542510000
F	-0.774913000	-4.001791000	1.351396000
F	-0.931869000	-3.087985000	3.910133000
F	-0.021258000	-0.597754000	4.500412000
F	1.090379000	0.914592000	2.640168000
F	2.362460000	-1.064009000	-2.945276000
F	4.622129000	-2.394571000	-3.288359000
F	6.406188000	-2.665106000	-1.253409000
F	5.858952000	-1.551511000	1.170275000
F	3.584656000	-0.205972000	1.548904000
F	3.869111000	1.980532000	-0.690314000
F	3.875725000	4.604250000	-0.415840000
F	1.633603000	5.936769000	0.354675000
F	-0.649042000	4.535446000	0.867020000
F	-0.680871000	1.895245000	0.645403000
H	-1.328365000	0.080974000	-0.927236000
O	0.430537000	0.060993000	-1.435679000
H	0.767952000	0.459933000	-2.238546000

E4

	Coordinates (Angstroms)		
	X	Y	Z
B	1.723872000	0.052248000	0.495191000
P	-2.629133000	0.021582000	0.342587000
C	-3.298459000	1.552331000	-0.424151000
C	-2.894203000	2.754398000	0.164927000
C	-4.161140000	1.596831000	-1.520005000
C	-3.340949000	3.973576000	-0.327905000
H	-2.217251000	2.737805000	1.014576000
C	-4.600602000	2.817977000	-2.020281000
H	-4.492834000	0.677947000	-1.989900000
C	-4.191956000	4.006981000	-1.427256000
H	-3.012538000	4.896337000	0.136889000
H	-5.267474000	2.838092000	-2.875361000

H	-4.532778000	4.957114000	-1.823268000
C	-3.864953000	-0.313400000	1.664004000
C	-3.400942000	-0.872654000	2.855629000
C	-5.230772000	-0.055030000	1.521952000
C	-4.285518000	-1.177143000	3.886166000
H	-2.343198000	-1.079039000	2.978477000
C	-6.112781000	-0.354230000	2.551606000
H	-5.608410000	0.382030000	0.603872000
C	-5.641559000	-0.917272000	3.734981000
H	-3.911494000	-1.613327000	4.805768000
H	-7.171020000	-0.150175000	2.430377000
H	-6.333116000	-1.150877000	4.537064000
C	-2.987737000	-1.310120000	-0.874849000
C	-2.525341000	-1.188215000	-2.190261000
C	-3.570927000	-2.516056000	-0.480552000
C	-2.646886000	-2.242248000	-3.085416000
H	-2.070269000	-0.263716000	-2.521422000
C	-3.686982000	-3.572876000	-1.377795000
H	-3.931635000	-2.642731000	0.533936000
C	-3.225420000	-3.440933000	-2.680833000
H	-2.277752000	-2.128275000	-4.098787000
H	-4.134699000	-4.504345000	-1.049759000
H	-3.308193000	-4.268023000	-3.376831000
C	1.042224000	-1.295780000	-0.108556000
C	0.224711000	-2.198003000	0.556103000
C	1.340396000	-1.645255000	-1.421640000
C	-0.271495000	-3.354193000	-0.026165000
C	0.852612000	-2.776431000	-2.046381000
C	0.043834000	-3.647342000	-1.336996000
C	1.147179000	1.488387000	0.015512000
C	1.533216000	2.650657000	0.679629000
C	0.305590000	1.693022000	-1.068571000
C	1.100119000	3.915198000	0.326665000
C	-0.137118000	2.943566000	-1.467023000
C	0.255755000	4.062296000	-0.760206000
C	3.346235000	-0.068990000	0.455297000
C	4.017449000	-0.991016000	1.245621000
C	4.155460000	0.654053000	-0.412359000
C	5.383398000	-1.192853000	1.217570000
C	5.528942000	0.481071000	-0.480311000
C	6.147645000	-0.443936000	0.340206000
F	-0.142929000	-2.002981000	1.836977000
F	-1.046737000	-4.179545000	0.675546000
F	-0.415946000	-4.753357000	-1.911026000
F	1.151179000	-3.036234000	-3.318544000
F	2.127300000	-0.842159000	-2.153847000
F	2.369848000	2.574750000	1.727028000
F	1.492569000	4.986560000	1.013677000
F	-0.184128000	5.265433000	-1.109266000
F	-0.954842000	3.066341000	-2.509105000
F	-0.128655000	0.668663000	-1.807960000
F	3.634860000	1.551973000	-1.255076000
F	6.257370000	1.198456000	-1.332601000
F	7.462944000	-0.614723000	0.286109000
F	5.961877000	-2.087133000	2.014448000
F	3.311434000	-1.747732000	2.120780000
H	-0.454261000	0.943468000	2.108609000

O	1.401647000	0.068410000	2.139968000
C	0.243804000	0.474605000	2.805976000
O	0.135814000	0.292399000	3.966872000
H	1.962625000	-0.484350000	2.716037000

E7

	Coordinates (Angstroms)		
	X	Y	Z
H	0.630524000	0.042842000	-1.144155000
B	1.392979000	0.171150000	-0.206284000
P	-2.603031000	-0.141722000	-0.818732000
H	-1.212182000	0.019401000	-0.890900000
C	-3.047451000	-1.112957000	-2.260552000
C	-2.037736000	-1.784922000	-2.950517000
C	-4.380028000	-1.208829000	-2.666910000
C	-2.369682000	-2.561289000	-4.053836000
H	-1.004693000	-1.702885000	-2.631889000
C	-4.698777000	-1.987779000	-3.768929000
H	-5.159580000	-0.677794000	-2.131066000
C	-3.695045000	-2.662853000	-4.459114000
H	-1.589861000	-3.083615000	-4.595208000
H	-5.729980000	-2.067213000	-4.092121000
H	-3.949900000	-3.268428000	-5.321537000
C	-3.326660000	1.500011000	-0.854780000
C	-2.589642000	2.540522000	-1.423103000
C	-4.606410000	1.725447000	-0.347272000
C	-3.140158000	3.812606000	-1.481313000
H	-1.590343000	2.363014000	-1.805715000
C	-5.148868000	3.001621000	-0.415125000
H	-5.170125000	0.918617000	0.107777000
C	-4.417190000	4.041109000	-0.979210000
H	-2.567891000	4.626805000	-1.909465000
H	-6.140567000	3.184538000	-0.018760000
H	-4.842804000	5.037114000	-1.022921000
C	-3.050183000	-1.010104000	0.693445000
C	-3.421685000	-2.353060000	0.638899000
C	-2.980264000	-0.339567000	1.917955000
C	-3.729017000	-3.024586000	1.815265000
H	-3.467518000	-2.875641000	-0.309305000
C	-3.284528000	-1.022761000	3.085462000
H	-2.692414000	0.703548000	1.963783000
C	-3.660350000	-2.361962000	3.033951000
H	-4.011358000	-4.069616000	1.776243000
H	-3.219886000	-0.510408000	4.037930000
H	-3.892459000	-2.891404000	3.950648000
C	0.796389000	-0.741745000	1.019821000
C	0.288735000	-2.001953000	0.731449000
C	0.778455000	-0.414233000	2.368567000
C	-0.217548000	-2.873308000	1.680126000
C	0.267628000	-1.246937000	3.351191000
C	-0.230200000	-2.489440000	3.006879000
C	2.815103000	-0.495666000	-0.646149000
C	3.033074000	-1.043565000	-1.899587000
C	3.872890000	-0.623215000	0.242031000
C	4.212252000	-1.680769000	-2.257294000
C	5.066949000	-1.246638000	-0.070403000
C	5.236377000	-1.783321000	-1.335251000

C	1.430489000	1.784321000	0.068529000
C	0.314350000	2.434163000	0.580598000
C	2.457103000	2.642409000	-0.300404000
C	0.196576000	3.803037000	0.725099000
C	2.391260000	4.023111000	-0.167929000
C	1.253171000	4.611783000	0.346377000
F	0.257774000	-2.442260000	-0.542777000
F	-0.698527000	-4.068495000	1.325583000
F	-0.729047000	-3.299433000	3.941246000
F	0.242199000	-0.859533000	4.630375000
F	1.259664000	0.765423000	2.794715000
F	2.086499000	-0.978951000	-2.854655000
F	4.369133000	-2.195936000	-3.481012000
F	6.378671000	-2.390098000	-1.660909000
F	6.054712000	-1.335564000	0.825248000
F	3.765546000	-0.106772000	1.478968000
F	3.596735000	2.171916000	-0.837072000
F	3.419243000	4.789281000	-0.543620000
F	1.170503000	5.936722000	0.471270000
F	-0.920782000	4.347768000	1.214523000
F	-0.761732000	1.713615000	0.964923000

E8

	Coordinates (Angstroms)		
	X	Y	Z
P	3.401584000	0.134224000	-0.678110000
C	4.441527000	1.639970000	-0.541210000
C	4.479862000	2.491145000	-1.648634000
C	5.181470000	1.969410000	0.595844000
C	5.256350000	3.643261000	-1.625927000
H	3.897646000	2.252088000	-2.533655000
C	5.950564000	3.127429000	0.620821000
H	5.157921000	1.321454000	1.465228000
C	5.991049000	3.964136000	-0.489327000
H	5.278995000	4.296268000	-2.491437000
H	6.518515000	3.375866000	1.510711000
H	6.589557000	4.868244000	-0.466952000
C	4.556346000	-1.120201000	-1.353527000
C	3.998025000	-2.286132000	-1.885057000
C	5.943578000	-0.966217000	-1.359925000
C	4.814097000	-3.287448000	-2.396929000
H	2.921200000	-2.415299000	-1.889805000
C	6.757768000	-1.964696000	-1.882804000
H	6.394269000	-0.066259000	-0.956744000
C	6.195693000	-3.127085000	-2.398759000
H	4.369330000	-4.189753000	-2.801747000
H	7.834198000	-1.832721000	-1.885036000
H	6.833286000	-3.904908000	-2.804432000
C	3.179699000	-0.370255000	1.070268000
C	2.237731000	0.351982000	1.808218000
C	3.847741000	-1.433762000	1.675045000
C	1.980153000	0.029074000	3.132852000
H	1.694654000	1.167078000	1.342973000
C	3.578466000	-1.764719000	2.999638000
H	4.570247000	-2.015280000	1.113547000
C	2.649557000	-1.034274000	3.730955000
H	1.242356000	0.594549000	3.690899000

H	4.091692000	-2.602900000	3.457567000
H	2.435456000	-1.301296000	4.759732000
C	-0.992356000	-0.873995000	0.365928000
C	0.047413000	-1.676431000	-0.081052000
C	-1.352986000	-1.081970000	1.692488000
C	0.685821000	-2.612444000	0.720002000
C	-0.727341000	-1.983210000	2.530418000
C	0.300602000	-2.765835000	2.035151000
C	-2.057346000	1.667731000	-0.128086000
C	-2.825417000	2.506660000	-0.925977000
C	-1.416152000	2.301646000	0.924432000
C	-2.978932000	3.861746000	-0.705487000
C	-1.542439000	3.656935000	1.186154000
C	-2.331160000	4.443697000	0.369449000
C	-3.373656000	-0.675475000	-0.691023000
C	-3.460574000	-1.884213000	-1.374669000
C	-4.549038000	-0.278395000	-0.063220000
C	-4.617181000	-2.637914000	-1.465314000
C	-5.726055000	-1.007834000	-0.124263000
C	-5.763594000	-2.193271000	-0.833655000
F	0.499160000	-1.605918000	-1.346346000
F	1.671923000	-3.363386000	0.226093000
F	0.907264000	-3.651987000	2.819952000
F	-1.100481000	-2.108778000	3.804748000
F	-2.377392000	-0.385215000	2.212204000
F	-3.466897000	1.998195000	-1.997618000
F	-3.734092000	4.606686000	-1.513460000
F	-2.459019000	5.745824000	0.606953000
F	-0.898206000	4.207755000	2.215865000
F	-0.607113000	1.629143000	1.752650000
F	-4.604580000	0.846257000	0.661229000
F	-6.822390000	-0.573963000	0.497172000
F	-6.887178000	-2.899488000	-0.905502000
F	-4.633062000	-3.783047000	-2.147186000
F	-2.377732000	-2.383754000	-1.995834000
O	-1.338250000	0.115539000	-2.028724000
C	-0.279127000	1.070121000	-2.507807000
O	0.545995000	1.383098000	-1.490599000
H	-0.811303000	1.958129000	-2.847503000
H	0.193720000	0.537967000	-3.334366000
H	1.305007000	0.768114000	-1.428417000
B	-1.933197000	0.097915000	-0.545442000
H	-2.009267000	-0.003254000	-2.715459000

TSE2-3

	Coordinates (Angstroms)		
	X	Y	Z
B	1.377353000	0.150500000	-0.237325000
P	-2.555768000	-0.094429000	-0.816394000
C	-3.202435000	-1.221082000	-2.083035000
C	-2.306137000	-2.122775000	-2.660654000
C	-4.546038000	-1.231718000	-2.462091000
C	-2.756839000	-3.038130000	-3.604951000
H	-1.260008000	-2.111633000	-2.374496000
C	-4.988418000	-2.144918000	-3.409060000
H	-5.245944000	-0.529034000	-2.023542000
C	-4.095617000	-3.049602000	-3.977130000

H	-2.059488000	-3.738983000	-4.049195000
H	-6.031542000	-2.151884000	-3.703773000
H	-4.445709000	-3.762851000	-4.714911000
C	-3.395992000	1.505107000	-0.976769000
C	-2.743279000	2.524201000	-1.672676000
C	-4.657840000	1.736905000	-0.425951000
C	-3.352234000	3.763485000	-1.823029000
H	-1.756718000	2.356569000	-2.090964000
C	-5.262303000	2.977566000	-0.580143000
H	-5.164444000	0.955600000	0.129827000
C	-4.610614000	3.989836000	-1.276837000
H	-2.838795000	4.554959000	-2.356569000
H	-6.240958000	3.154981000	-0.149195000
H	-5.082740000	4.959287000	-1.389713000
C	-3.038897000	-0.781086000	0.796954000
C	-3.483150000	-2.097122000	0.917517000
C	-2.909989000	0.016545000	1.937775000
C	-3.806982000	-2.607406000	2.169431000
H	-3.573052000	-2.728731000	0.041254000
C	-3.239950000	-0.498523000	3.183225000
H	-2.557659000	1.038247000	1.856154000
C	-3.691247000	-1.809479000	3.300620000
H	-4.146223000	-3.632844000	2.258226000
H	-3.134673000	0.122819000	4.065156000
H	-3.940318000	-2.211799000	4.275672000
C	0.747346000	-0.635423000	1.064373000
C	0.236420000	-1.918108000	0.903353000
C	0.717356000	-0.162848000	2.369232000
C	-0.293718000	-2.673193000	1.935356000
C	0.193614000	-0.883880000	3.429376000
C	-0.314165000	-2.150224000	3.213042000
C	2.725559000	-0.670445000	-0.687991000
C	2.955683000	-1.209863000	-1.942946000
C	3.728041000	-0.912650000	0.242248000
C	4.079969000	-1.959578000	-2.256890000
C	4.865120000	-1.650694000	-0.026228000
C	5.040148000	-2.185877000	-1.290653000
C	1.618373000	1.764894000	-0.035284000
C	0.542046000	2.561049000	0.341170000
C	2.776902000	2.470914000	-0.323736000
C	0.582463000	3.938260000	0.428729000
C	2.868915000	3.854314000	-0.239467000
C	1.765921000	4.594184000	0.136879000
F	0.230822000	-2.502756000	-0.308044000
F	-0.789832000	-3.890415000	1.704284000
F	-0.826820000	-2.851241000	4.221867000
F	0.160442000	-0.361459000	4.657766000
F	1.213246000	1.045243000	2.678549000
F	2.088215000	-1.016012000	-2.955739000
F	4.243151000	-2.458301000	-3.484642000
F	6.126404000	-2.902787000	-1.573215000
F	5.793374000	-1.847970000	0.912148000
F	3.629244000	-0.389753000	1.476515000
F	3.897106000	1.846776000	-0.720658000
F	4.013757000	4.474876000	-0.531355000
F	1.836920000	5.920833000	0.215447000
F	-0.495792000	4.634961000	0.792396000

F	-0.633395000	1.985940000	0.656558000
H	-0.931326000	0.038068000	-1.053527000
O	0.307605000	0.026097000	-1.312801000
H	0.528740000	0.455147000	-2.144919000

TSE3-4

	Coordinates (Angstroms)		
	X	Y	Z
B	1.634350000	0.084741000	0.621202000
P	-2.780405000	-0.083681000	0.436372000
C	-3.267003000	1.513164000	-0.272789000
C	-3.004917000	2.639575000	0.513543000
C	-3.878823000	1.663527000	-1.517272000
C	-3.353987000	3.903415000	0.058280000
H	-2.524372000	2.531486000	1.481195000
C	-4.213024000	2.932982000	-1.973560000
H	-4.092920000	0.797364000	-2.132464000
C	-3.952009000	4.050262000	-1.188421000
H	-3.138922000	4.773943000	0.666801000
H	-4.679357000	3.048331000	-2.945148000
H	-4.206579000	5.038922000	-1.552691000
C	-4.123376000	-0.599424000	1.545693000
C	-3.782454000	-1.346735000	2.674936000
C	-5.459791000	-0.283697000	1.295562000
C	-4.774988000	-1.782358000	3.545077000
H	-2.744665000	-1.595312000	2.871569000
C	-6.446474000	-0.719188000	2.169679000
H	-5.729189000	0.301590000	0.422961000
C	-6.104995000	-1.468100000	3.292306000
H	-4.507195000	-2.362197000	4.420891000
H	-7.484569000	-0.474640000	1.975493000
H	-6.878494000	-1.804371000	3.973661000
C	-2.702802000	-1.323913000	-0.890705000
C	-2.024589000	-1.025111000	-2.074675000
C	-3.222611000	-2.604204000	-0.699384000
C	-1.856208000	-1.998913000	-3.047719000
H	-1.629807000	-0.033005000	-2.243551000
C	-3.056081000	-3.574529000	-1.680752000
H	-3.748666000	-2.853165000	0.214927000
C	-2.368947000	-3.277292000	-2.850679000
H	-1.321480000	-1.757597000	-3.959245000
H	-3.457507000	-4.568554000	-1.522060000
H	-2.231048000	-4.040303000	-3.608231000
C	1.240632000	-1.356730000	-0.074318000
C	0.330468000	-2.272148000	0.436751000
C	1.882219000	-1.783101000	-1.232746000
C	0.064229000	-3.502869000	-0.144476000
C	1.627267000	-2.987774000	-1.861690000
C	0.705134000	-3.859441000	-1.312256000
C	0.924856000	1.391201000	-0.112082000
C	0.774260000	2.585069000	0.587882000
C	0.463524000	1.451031000	-1.419715000
C	0.188817000	3.725395000	0.068977000
C	-0.143703000	2.565700000	-1.978882000
C	-0.278915000	3.717293000	-1.231794000
C	3.274786000	0.208742000	0.700440000
C	3.989978000	-0.749150000	1.413392000

C	4.055227000	1.185924000	0.097879000
C	5.368830000	-0.745888000	1.535586000
C	5.437482000	1.225816000	0.197073000
C	6.100358000	0.253767000	0.921315000
F	-0.377247000	-2.020760000	1.555774000
F	-0.810782000	-4.339892000	0.417728000
F	0.436931000	-5.022126000	-1.903371000
F	2.260269000	-3.316505000	-2.990363000
F	2.803507000	-1.002332000	-1.820073000
F	1.249998000	2.701349000	1.845858000
F	0.059247000	4.826553000	0.811497000
F	-0.850688000	4.800463000	-1.749111000
F	-0.594232000	2.528021000	-3.234541000
F	0.608543000	0.412131000	-2.256591000
F	3.504072000	2.155288000	-0.648965000
F	6.133928000	2.191607000	-0.406220000
F	7.427044000	0.277956000	1.024880000
F	5.996047000	-1.694417000	2.234273000
F	3.347252000	-1.757102000	2.024696000
H	-1.436063000	0.205259000	1.495223000
O	1.151758000	0.042945000	2.061029000
C	-0.616974000	0.501595000	2.556853000
O	-0.637494000	0.814222000	3.654864000
H	1.713684000	0.580957000	2.628848000

TSE1-7

	Coordinates (Angstroms)		
	X	Y	Z
H	0.671368000	0.097478000	-1.235303000
B	1.357842000	-0.142467000	-0.034314000
P	-2.267583000	0.131699000	-0.884834000
H	-0.062583000	-0.006024000	-0.882239000
C	-3.472731000	-1.154361000	-1.392158000
C	-3.035593000	-2.474163000	-1.507385000
C	-4.816276000	-0.856905000	-1.638464000
C	-3.929029000	-3.484945000	-1.848217000
H	-1.995090000	-2.718210000	-1.335346000
C	-5.705082000	-1.865465000	-1.983448000
H	-5.172271000	0.164412000	-1.559104000
C	-5.263244000	-3.182046000	-2.085817000
H	-3.576693000	-4.506915000	-1.932021000
H	-6.745518000	-1.625496000	-2.172254000
H	-5.960005000	-3.967990000	-2.355388000
C	-2.789957000	1.600347000	-1.848874000
C	-2.201135000	1.776192000	-3.103437000
C	-3.734474000	2.525955000	-1.402763000
C	-2.559751000	2.853346000	-3.904237000
H	-1.455344000	1.069747000	-3.455057000
C	-4.086374000	3.607327000	-2.202434000
H	-4.197334000	2.406929000	-0.429584000
C	-3.501527000	3.771849000	-3.453197000
H	-2.095554000	2.980773000	-4.875778000
H	-4.819794000	4.322403000	-1.846578000
H	-3.776187000	4.617426000	-4.074135000
C	-2.751962000	0.527229000	0.836767000
C	-3.573635000	-0.304428000	1.596195000
C	-2.173698000	1.649009000	1.441426000

C	-3.820117000	-0.014942000	2.935286000
H	-4.021488000	-1.185642000	1.150782000
C	-2.427465000	1.939207000	2.773584000
H	-1.523413000	2.299677000	0.866199000
C	-3.251401000	1.106014000	3.524915000
H	-4.453629000	-0.674092000	3.518045000
H	-1.970396000	2.809924000	3.229625000
H	-3.438000000	1.324522000	4.570101000
C	0.476996000	-0.925315000	1.061780000
C	-0.103113000	-2.147166000	0.739822000
C	0.207619000	-0.462690000	2.342933000
C	-0.932622000	-2.850908000	1.590759000
C	-0.588420000	-1.157001000	3.239737000
C	-1.177383000	-2.345630000	2.855874000
C	2.513667000	-0.997868000	-0.775426000
C	2.903234000	-0.690045000	-2.071212000
C	3.224425000	-2.020682000	-0.163064000
C	3.917002000	-1.349362000	-2.741290000
C	4.250666000	-2.701623000	-0.797324000
C	4.597084000	-2.365761000	-2.093801000
C	1.835543000	1.359106000	0.308633000
C	1.245928000	2.513391000	-0.176750000
C	2.935725000	1.550896000	1.132822000
C	1.692333000	3.785173000	0.143753000
C	3.417586000	2.800268000	1.473416000
C	2.787813000	3.928235000	0.973842000
F	0.121052000	-2.683588000	-0.471420000
F	-1.494623000	-3.996308000	1.208382000
F	-1.980465000	-2.993554000	3.691230000
F	-0.806038000	-0.676262000	4.461918000
F	0.707619000	0.696574000	2.782880000
F	2.268503000	0.293239000	-2.733330000
F	4.243290000	-1.017115000	-3.988845000
F	5.578824000	-3.012444000	-2.712683000
F	4.907407000	-3.675240000	-0.168650000
F	2.950711000	-2.386783000	1.094199000
F	3.557878000	0.487586000	1.661492000
F	4.470820000	2.931216000	2.278210000
F	3.233883000	5.138751000	1.293422000
F	1.074995000	4.862256000	-0.339063000
F	0.183108000	2.439947000	-0.994563000

TSE7

	Coordinates (Angstroms)		
	X	Y	Z
H	1.312206000	-0.016050000	-1.263447000
B	1.932342000	-0.016565000	-0.174251000
P	-3.042780000	0.045836000	-0.776041000
H	-1.308857000	-0.081612000	-1.918518000
C	-3.983847000	-1.434651000	-1.297470000
C	-3.261361000	-2.621812000	-1.446265000
C	-5.359154000	-1.437175000	-1.531231000
C	-3.908634000	-3.799650000	-1.797616000
H	-2.189671000	-2.629754000	-1.275326000
C	-6.002388000	-2.613785000	-1.897443000
H	-5.930963000	-0.521656000	-1.428160000
C	-5.280705000	-3.795819000	-2.025610000

H	-3.340384000	-4.717436000	-1.898496000
H	-7.071402000	-2.607147000	-2.079397000
H	-5.786867000	-4.712945000	-2.305992000
C	-4.132930000	1.455763000	-1.181933000
C	-3.944788000	2.072966000	-2.420623000
C	-5.138300000	1.918923000	-0.330906000
C	-4.759693000	3.129436000	-2.809638000
H	-3.156891000	1.728722000	-3.083644000
C	-5.946343000	2.980888000	-0.718614000
H	-5.289601000	1.452377000	0.636236000
C	-5.759901000	3.584863000	-1.957874000
H	-4.607381000	3.602161000	-3.773417000
H	-6.722949000	3.336736000	-0.050802000
H	-6.391700000	4.413759000	-2.257464000
C	-3.091160000	-0.040502000	1.055581000
C	-3.619267000	-1.132753000	1.743280000
C	-2.562064000	1.030664000	1.783715000
C	-3.626987000	-1.149278000	3.134961000
H	-4.031442000	-1.976559000	1.202323000
C	-2.572694000	1.010834000	3.170830000
H	-2.147559000	1.888068000	1.267992000
C	-3.109008000	-0.078568000	3.850158000
H	-4.039115000	-2.005690000	3.656074000
H	-2.155202000	1.846277000	3.721253000
H	-3.111185000	-0.096189000	4.934049000
C	1.029472000	-0.981732000	0.764728000
C	0.523380000	-2.170309000	0.254745000
C	0.698547000	-0.726767000	2.087830000
C	-0.285203000	-3.031363000	0.975271000
C	-0.080025000	-1.578130000	2.855705000
C	-0.592303000	-2.730146000	2.289876000
C	3.385932000	-0.605039000	-0.582683000
C	4.009659000	-0.150384000	-1.734710000
C	4.100470000	-1.553329000	0.133050000
C	5.242611000	-0.594482000	-2.174072000
C	5.339716000	-2.028971000	-0.267671000
C	5.913911000	-1.549078000	-1.430397000
C	1.942208000	1.541216000	0.275702000
C	0.768846000	2.277561000	0.205672000
C	3.038718000	2.240168000	0.758724000
C	0.662112000	3.605552000	0.573237000
C	2.981075000	3.573474000	1.135465000
C	1.785536000	4.262400000	1.042656000
F	0.831959000	-2.545501000	-0.999134000
F	-0.763149000	-4.148815000	0.419859000
F	-1.358902000	-3.550754000	3.005292000
F	-0.325795000	-1.302535000	4.137354000
F	1.159260000	0.369084000	2.712446000
F	3.408757000	0.796762000	-2.492043000
F	5.787581000	-0.117708000	-3.294687000
F	7.102791000	-1.996948000	-1.828511000
F	5.986524000	-2.940761000	0.460504000
F	3.620799000	-2.045193000	1.285581000
F	4.226954000	1.635701000	0.913396000
F	4.067156000	4.196640000	1.597305000
F	1.715991000	5.541587000	1.404730000
F	-0.504549000	4.248824000	0.489172000

F	-0.358330000	1.679951000	-0.218697000
C	0.482541000	0.109488000	-2.706739000
O	-0.578130000	-0.548354000	-2.462346000
O	0.412218000	1.402431000	-2.831633000
H	1.293782000	-0.437790000	-3.179012000
H	1.281171000	1.771565000	-3.051775000

TSE8

	Coordinates (Angstroms)		
	X	Y	Z
B	1.777012000	0.124325000	0.687147000
P	-2.964990000	-0.036264000	0.379892000
C	-3.537604000	1.455073000	-0.477641000
C	-3.073615000	2.685618000	-0.004899000
C	-4.414493000	1.411227000	-1.563523000
C	-3.485128000	3.862599000	-0.616831000
H	-2.378983000	2.724751000	0.826994000
C	-4.822233000	2.592453000	-2.168997000
H	-4.772065000	0.460193000	-1.942591000
C	-4.357724000	3.816232000	-1.697227000
H	-3.109789000	4.813158000	-0.256775000
H	-5.501202000	2.556993000	-3.013278000
H	-4.671631000	4.735220000	-2.179265000
C	-4.203459000	-0.506345000	1.618113000
C	-3.746286000	-1.113217000	2.789529000
C	-5.570140000	-0.304565000	1.423436000
C	-4.654845000	-1.526562000	3.756444000
H	-2.683424000	-1.273201000	2.942210000
C	-6.472497000	-0.714603000	2.395623000
H	-5.931620000	0.173915000	0.520074000
C	-6.016168000	-1.326365000	3.559390000
H	-4.297413000	-2.000195000	4.663422000
H	-7.533947000	-0.554908000	2.244797000
H	-6.724931000	-1.645323000	4.315255000
C	-2.900945000	-1.387312000	-0.835334000
C	-2.338200000	-1.159710000	-2.094041000
C	-3.389078000	-2.653773000	-0.513528000
C	-2.267104000	-2.191722000	-3.018208000
H	-1.964033000	-0.179313000	-2.356780000
C	-3.315004000	-3.681916000	-1.445653000
H	-3.828303000	-2.844963000	0.458507000
C	-2.757028000	-3.453450000	-2.696587000
H	-1.822816000	-2.010028000	-3.990049000
H	-3.692212000	-4.664645000	-1.188171000
H	-2.697731000	-4.258091000	-3.420230000
C	1.139352000	-1.200351000	-0.055786000
C	0.321967000	-2.155985000	0.526142000
C	1.534146000	-1.506524000	-1.354449000
C	-0.049150000	-3.338048000	-0.100366000
C	1.167385000	-2.655600000	-2.027326000
C	0.376810000	-3.593149000	-1.386209000
C	1.239958000	1.571171000	0.090991000
C	1.567751000	2.732584000	0.778735000
C	0.407751000	1.778197000	-0.994224000
C	1.104253000	3.991446000	0.451606000
C	-0.075656000	3.021672000	-1.374406000
C	0.265168000	4.138033000	-0.640015000

C	3.421588000	-0.038552000	0.588546000
C	4.035374000	-1.098775000	1.249521000
C	4.288210000	0.769036000	-0.135870000
C	5.399040000	-1.337226000	1.221631000
C	5.658648000	0.565576000	-0.188700000
C	6.220487000	-0.494035000	0.496808000
F	-0.177329000	-1.999214000	1.765744000
F	-0.815888000	-4.226794000	0.537566000
F	0.027515000	-4.718271000	-2.007344000
F	1.565406000	-2.873010000	-3.283526000
F	2.318398000	-0.649132000	-2.030018000
F	2.389648000	2.664253000	1.849212000
F	1.453359000	5.061684000	1.169985000
F	-0.209680000	5.338393000	-0.971749000
F	-0.884025000	3.138420000	-2.430421000
F	-0.013595000	0.753544000	-1.755111000
F	3.834267000	1.804648000	-0.858589000
F	6.439527000	1.381685000	-0.900802000
F	7.534723000	-0.703721000	0.456155000
F	5.925793000	-2.372147000	1.880970000
F	3.302815000	-1.975559000	1.954865000
H	-1.695325000	0.450675000	1.232697000
O	1.445716000	0.085159000	2.163191000
C	-0.250504000	0.677596000	2.901998000
O	-0.936130000	1.222418000	2.007974000
H	0.247034000	1.325686000	3.627830000
H	-0.465203000	-0.340135000	3.224434000
H	2.112127000	0.584548000	2.642879000

TSE9

	Coordinates (Angstroms)		
	X	Y	Z
H	1.222618000	-0.069418000	-1.330521000
B	1.871508000	-0.042230000	-0.210729000
P	-3.017021000	-0.024593000	-0.780355000
H	-1.645622000	-0.333793000	-1.481579000
C	-4.043235000	-1.462195000	-1.179164000
C	-3.400461000	-2.699808000	-1.267020000
C	-5.422350000	-1.373835000	-1.366743000
C	-4.141336000	-3.845727000	-1.526668000
H	-2.325334000	-2.769511000	-1.138595000
C	-6.155114000	-2.522815000	-1.634700000
H	-5.923802000	-0.414204000	-1.309393000
C	-5.516807000	-3.756710000	-1.710563000
H	-3.641113000	-4.804910000	-1.591954000
H	-7.226211000	-2.453262000	-1.785886000
H	-6.092393000	-4.651467000	-1.919829000
C	-3.772585000	1.468410000	-1.467287000
C	-3.291801000	1.950884000	-2.685787000
C	-4.813688000	2.130975000	-0.813759000
C	-3.856223000	3.087954000	-3.249991000
H	-2.475711000	1.445669000	-3.190855000
C	-5.374517000	3.265306000	-1.384738000
H	-5.180844000	1.767421000	0.139838000
C	-4.896302000	3.743292000	-2.600869000
H	-3.477407000	3.465497000	-4.192660000
H	-6.183309000	3.778745000	-0.877697000

H	-5.332937000	4.632786000	-3.040876000
C	-3.022485000	0.156350000	1.025851000
C	-3.569727000	-0.827487000	1.849290000
C	-2.464144000	1.306234000	1.590517000
C	-3.571469000	-0.652311000	3.227920000
H	-3.999949000	-1.726460000	1.423064000
C	-2.464995000	1.470616000	2.967769000
H	-2.042543000	2.078311000	0.958473000
C	-3.025283000	0.495893000	3.786427000
H	-3.994262000	-1.420453000	3.864677000
H	-2.026605000	2.361917000	3.400998000
H	-3.022611000	0.626755000	4.862355000
C	0.978738000	-1.010966000	0.719701000
C	0.585075000	-2.260258000	0.251748000
C	0.534334000	-0.697763000	1.998188000
C	-0.252907000	-3.108392000	0.954773000
C	-0.248105000	-1.549504000	2.760377000
C	-0.678559000	-2.746067000	2.219816000
C	3.325562000	-0.592520000	-0.646164000
C	3.920857000	-0.115429000	-1.805282000
C	4.069319000	-1.524506000	0.061234000
C	5.159961000	-0.532612000	-2.256717000
C	5.314623000	-1.968736000	-0.353760000
C	5.862531000	-1.471367000	-1.522258000
C	1.845943000	1.521718000	0.206103000
C	0.740544000	2.319528000	-0.042826000
C	2.881257000	2.153107000	0.880265000
C	0.642093000	3.643940000	0.342338000
C	2.830677000	3.478991000	1.279134000
C	1.701579000	4.231282000	1.009264000
F	1.056215000	-2.716001000	-0.916957000
F	-0.637918000	-4.276387000	0.432489000
F	-1.476573000	-3.550884000	2.918373000
F	-0.582810000	-1.230421000	4.011040000
F	0.885588000	0.454622000	2.590104000
F	3.282236000	0.804553000	-2.551265000
F	5.681477000	-0.043477000	-3.383443000
F	7.057393000	-1.889280000	-1.934988000
F	5.992649000	-2.865893000	0.364846000
F	3.611770000	-2.028058000	1.217695000
F	3.986454000	1.472376000	1.219203000
F	3.852872000	4.033200000	1.932932000
F	1.633557000	5.504152000	1.391870000
F	-0.462641000	4.349027000	0.088380000
F	-0.333561000	1.804499000	-0.673281000
C	0.405294000	-0.312440000	-2.494412000
O	-0.638052000	-0.948781000	-2.174862000
H	1.199274000	-0.860195000	-3.014246000
H	0.352797000	0.760408000	-2.712471000

F1

	Coordinates (Angstroms)		
	X	Y	Z
B	1.566762000	-0.264706000	1.086244000
P	-1.408730000	0.251029000	0.330425000
C	1.550055000	-1.729912000	0.496899000
C	1.719140000	-1.960382000	-0.865463000

C	1.375477000	-2.862769000	1.283748000
C	1.712890000	-3.225193000	-1.421740000
C	1.380966000	-4.146055000	0.763449000
C	1.548008000	-4.326040000	-0.598183000
C	2.676206000	0.723966000	0.550155000
C	2.396741000	2.052674000	0.249055000
C	3.996371000	0.325464000	0.364426000
C	3.353110000	2.933338000	-0.223930000
C	4.982446000	1.182831000	-0.088156000
C	4.655180000	2.494177000	-0.388268000
F	1.846138000	-0.931311000	-1.710414000
F	1.837098000	-3.393442000	-2.736450000
F	1.537274000	-5.548081000	-1.114894000
F	1.226125000	-5.203536000	1.557636000
F	1.216083000	-2.752236000	2.608008000
F	1.154152000	2.526772000	0.375863000
F	3.035062000	4.191527000	-0.519395000
F	5.587874000	3.327419000	-0.830612000
F	6.237243000	0.762703000	-0.236492000
F	4.367670000	-0.928770000	0.647795000
C	0.616746000	0.181171000	2.256568000
H	0.988004000	-0.331385000	3.156405000
H	0.746584000	1.244637000	2.456805000
C	-0.866246000	-0.164348000	2.057030000
H	-0.988470000	-1.253114000	2.065969000
H	-1.491377000	0.223848000	2.859989000
C	-2.984034000	-0.719358000	0.145753000
C	-4.223715000	-0.356177000	0.712524000
C	-2.922493000	-1.876559000	-0.660927000
C	-5.358140000	-1.115629000	0.425505000
C	-4.081160000	-2.606459000	-0.918721000
C	-5.314381000	-2.236367000	-0.395025000
H	-6.306636000	-0.819734000	0.865444000
H	-4.016111000	-3.488619000	-1.549565000
C	-1.945457000	2.005066000	0.072535000
C	-2.280433000	2.283898000	-1.274482000
C	-1.971582000	3.064983000	1.004572000
C	-2.668696000	3.570803000	-1.643409000
C	-2.354249000	4.337695000	0.581053000
C	-2.717656000	4.615489000	-0.731215000
H	-2.923868000	3.759630000	-2.682397000
H	-2.368897000	5.141775000	1.311568000
C	-1.628243000	-2.385979000	-1.242191000
C	-4.398865000	0.816633000	1.639995000
C	-6.551573000	-3.046746000	-0.672635000
C	-1.610139000	2.928606000	2.461573000
C	-2.234536000	1.238406000	-2.362829000
C	-3.160200000	5.994019000	-1.141538000
H	-2.990642000	6.163756000	-2.207314000
H	-4.230240000	6.129836000	-0.951323000
H	-2.627114000	6.765600000	-0.580511000
H	-0.568770000	2.635670000	2.595566000
H	-1.743344000	3.886158000	2.967971000
H	-2.233099000	2.198398000	2.978763000
H	-1.283541000	0.697577000	-2.359215000
H	-3.028284000	0.494490000	-2.247678000
H	-2.356373000	1.707107000	-3.341050000

H	-3.620744000	0.838850000	2.403093000
H	-4.360353000	1.767205000	1.103390000
H	-5.363269000	0.754709000	2.147522000
H	-0.950368000	-2.690724000	-0.441666000
H	-1.807881000	-3.257518000	-1.875024000
H	-1.111457000	-1.628226000	-1.835454000
H	-6.686406000	-3.817716000	0.093635000
H	-7.446413000	-2.419851000	-0.669333000
H	-6.485459000	-3.551138000	-1.639359000

F1_CO

	Coordinates (Angstroms)		
	X	Y	Z
B	-1.286124000	0.002513000	-0.543528000
P	1.661401000	0.250919000	-0.405230000
C	-1.645168000	-1.585546000	-0.290747000
C	-2.149286000	-1.995475000	0.942065000
C	-1.446420000	-2.621762000	-1.193643000
C	-2.401452000	-3.312011000	1.278941000
C	-1.679010000	-3.957512000	-0.896008000
C	-2.154031000	-4.308262000	0.351329000
C	-2.547380000	1.005997000	-0.278121000
C	-2.324462000	2.348102000	0.005053000
C	-3.885339000	0.656365000	-0.392591000
C	-3.331809000	3.276498000	0.197421000
C	-4.927353000	1.551764000	-0.206289000
C	-4.650407000	2.871117000	0.097050000
F	-2.423634000	-1.084861000	1.888215000
F	-2.878315000	-3.631089000	2.483673000
F	-2.384455000	-5.583820000	0.653766000
F	-1.451666000	-4.904016000	-1.809515000
F	-1.000556000	-2.393703000	-2.441869000
F	-1.063352000	2.816446000	0.082163000
F	-3.046766000	4.551766000	0.470602000
F	-5.640657000	3.744112000	0.277526000
F	-6.196385000	1.152433000	-0.324378000
F	-4.244374000	-0.598909000	-0.712259000
C	-0.586726000	0.364952000	-1.970187000
H	-1.112823000	-0.072947000	-2.819812000
H	-0.640904000	1.448068000	-2.111973000
C	0.884636000	-0.079151000	-2.019215000
H	0.953647000	-1.168064000	-2.094330000
H	1.447632000	0.330689000	-2.854579000
C	2.372609000	1.904703000	-0.085112000
C	2.389248000	2.973172000	-1.006834000
C	2.904230000	2.089396000	1.211859000
C	2.920591000	4.195061000	-0.599771000
C	3.429802000	3.330555000	1.557022000
C	3.444871000	4.399402000	0.670444000
H	2.923752000	5.016292000	-1.309866000
H	3.833234000	3.462855000	2.556341000
C	2.956330000	-1.018224000	-0.147032000
C	4.196041000	-0.869211000	-0.806504000
C	2.723574000	-2.136037000	0.676916000
C	5.183053000	-1.827584000	-0.595034000
C	3.746910000	-3.065182000	0.849662000
C	4.985055000	-2.926216000	0.234724000

H	6.136444000	-1.712102000	-1.102068000
H	3.563932000	-3.926224000	1.484951000
C	-0.116693000	0.297958000	0.595840000
O	-0.137947000	0.589329000	1.742437000
C	1.417786000	-2.393349000	1.380013000
C	4.517939000	0.272506000	-1.736757000
C	6.083771000	-3.927374000	0.460025000
C	2.979154000	0.997244000	2.249769000
C	1.902255000	2.889151000	-2.428951000
C	4.033170000	5.725414000	1.064244000
H	1.186129000	-1.620836000	2.116607000
H	0.588320000	-2.442017000	0.672948000
H	1.455128000	-3.348305000	1.905417000
H	6.819138000	-3.533757000	1.169330000
H	5.692462000	-4.861207000	0.868202000
H	6.611915000	-4.147876000	-0.470836000
H	5.436576000	0.057707000	-2.284294000
H	3.730304000	0.439563000	-2.474748000
H	4.664860000	1.207118000	-1.190256000
H	3.134492000	1.436269000	3.236361000
H	2.064468000	0.407470000	2.302767000
H	3.814145000	0.320928000	2.046749000
H	5.108283000	5.742801000	0.856706000
H	3.574493000	6.544516000	0.506291000
H	3.900164000	5.914162000	2.131734000
H	2.484514000	2.170147000	-3.008986000
H	0.851042000	2.609183000	-2.492355000
H	2.010353000	3.860590000	-2.912510000

F2

	Coordinates (Angstroms)		
	X	Y	Z
B	-1.547002000	0.591628000	-0.112309000
P	1.689139000	0.813574000	-0.157596000
C	-1.194594000	-1.015326000	-0.048568000
C	-0.835227000	-1.711820000	1.095581000
C	-1.184560000	-1.787220000	-1.203110000
C	-0.437034000	-3.037926000	1.110442000
C	-0.799744000	-3.114668000	-1.241232000
C	-0.405900000	-3.743925000	-0.074610000
C	-3.137270000	0.950973000	-0.014859000
C	-3.547941000	2.274307000	0.131746000
C	-4.175433000	0.031430000	-0.099989000
C	-4.874424000	2.665367000	0.189608000
C	-5.516485000	0.382847000	-0.050692000
C	-5.869621000	1.709789000	0.094710000
F	-0.849334000	-1.105055000	2.308684000
F	-0.073115000	-3.628078000	2.249360000
F	0.007011000	-5.008579000	-0.097514000
F	-0.766117000	-3.781975000	-2.396909000
F	-1.559569000	-1.247101000	-2.375670000
F	-2.645436000	3.263637000	0.228906000
F	-5.201792000	3.950994000	0.333563000
F	-7.150694000	2.065778000	0.145690000
F	-6.466211000	-0.550419000	-0.137547000
F	-3.934571000	-1.281511000	-0.239634000
C	-0.798520000	1.376091000	-1.330901000

H	-1.402934000	1.295262000	-2.240346000
H	-0.788971000	2.441762000	-1.079339000
C	0.636092000	0.929574000	-1.685677000
H	0.596428000	-0.041725000	-2.167057000
H	1.104434000	1.616080000	-2.396143000
C	2.387276000	-0.876874000	0.056989000
C	2.696638000	-1.191043000	1.404654000
C	2.476561000	-1.893596000	-0.914037000
C	3.021889000	-2.495315000	1.753300000
C	2.792827000	-3.193137000	-0.507022000
C	3.045985000	-3.523143000	0.814520000
H	3.242124000	-2.719503000	2.792905000
H	2.836382000	-3.971934000	-1.263267000
C	3.025482000	2.081410000	-0.242289000
C	2.779860000	3.278216000	0.472220000
C	4.253922000	1.931344000	-0.917471000
C	3.755892000	4.270308000	0.506429000
C	5.207177000	2.947307000	-0.837267000
C	4.983355000	4.121154000	-0.130343000
H	3.551298000	5.187271000	1.051723000
H	6.152245000	2.814361000	-1.355857000
C	2.664680000	-0.155977000	2.502956000
C	2.274387000	-1.714322000	-2.399475000
C	3.303850000	-4.943966000	1.232289000
C	1.493704000	3.545885000	1.216924000
C	4.590081000	0.736566000	-1.767111000
C	6.035212000	5.192157000	-0.035512000
H	2.410802000	-5.365004000	1.704773000
H	3.555775000	-5.572128000	0.375368000
H	4.117799000	-5.003596000	1.959416000
H	1.638472000	0.150241000	2.725590000
H	3.094912000	-0.566697000	3.418065000
H	3.227463000	0.744425000	2.243134000
H	2.444738000	-0.698213000	-2.741148000
H	2.965867000	-2.364802000	-2.939850000
H	1.263776000	-2.003731000	-2.703325000
H	0.616374000	3.473569000	0.569888000
H	1.355946000	2.840317000	2.041549000
H	1.509701000	4.551573000	1.640760000
H	5.594425000	6.187257000	-0.135947000
H	6.536140000	5.152101000	0.937257000
H	6.797297000	5.071977000	-0.808502000
H	3.898040000	0.659182000	-2.608727000
H	5.596322000	0.834228000	-2.177684000
H	4.540131000	-0.198747000	-1.207978000
O	-0.897248000	1.259836000	1.194933000
H	-1.129205000	0.819468000	2.023355000
H	0.119899000	1.168524000	1.043821000

F3

	Coordinates (Angstroms)		
	X	Y	Z
B	-1.542169000	0.761017000	0.187105000
P	1.760048000	0.758049000	-0.251868000
C	-1.116801000	-0.858944000	0.165224000
C	-0.616204000	-1.566423000	1.248573000
C	-1.212952000	-1.612257000	-0.996618000

C	-0.199920000	-2.888312000	1.189152000
C	-0.811532000	-2.930191000	-1.109622000
C	-0.287758000	-3.574913000	-0.004052000
C	-3.203047000	0.923445000	0.073399000
C	-3.757867000	2.183099000	-0.133016000
C	-4.145749000	-0.080732000	0.249091000
C	-5.117875000	2.436154000	-0.188876000
C	-5.517747000	0.124067000	0.199580000
C	-6.011235000	1.393389000	-0.024214000
F	-0.499350000	-0.993575000	2.466691000
F	0.294440000	-3.497973000	2.270725000
F	0.136669000	-4.835534000	-0.095542000
F	-0.889173000	-3.576959000	-2.277737000
F	-1.722820000	-1.053247000	-2.114687000
F	-2.967588000	3.261741000	-0.288277000
F	-5.577548000	3.674654000	-0.397063000
F	-7.325579000	1.612417000	-0.074801000
F	-6.365462000	-0.895574000	0.373932000
F	-3.777618000	-1.352520000	0.500354000
C	-0.796183000	1.570578000	-1.050573000
H	-1.442852000	1.640177000	-1.931637000
H	-0.644778000	2.598608000	-0.707394000
C	0.541235000	1.022203000	-1.581850000
H	0.382636000	0.069441000	-2.075749000
H	1.005013000	1.691782000	-2.311447000
C	2.459546000	-0.917879000	-0.149882000
C	2.882878000	-1.311115000	1.143550000
C	2.476590000	-1.843697000	-1.210236000
C	3.264746000	-2.627446000	1.353419000
C	2.865872000	-3.155614000	-0.934678000
C	3.238965000	-3.574244000	0.332914000
H	3.569378000	-2.928392000	2.350848000
H	2.858543000	-3.874719000	-1.748035000
C	2.979136000	2.102310000	-0.149320000
C	2.796355000	3.108507000	0.827379000
C	4.078764000	2.153089000	-1.026753000
C	3.735893000	4.131678000	0.902302000
C	4.988772000	3.199527000	-0.902969000
C	4.838271000	4.194875000	0.054829000
H	3.600494000	4.906369000	1.651093000
H	5.835955000	3.235689000	-1.580736000
C	2.900343000	-0.373729000	2.323800000
C	2.099713000	-1.560307000	-2.644314000
C	3.578048000	-5.009339000	0.617044000
C	1.652388000	3.129593000	1.808621000
C	4.306016000	1.134054000	-2.108707000
C	5.833140000	5.314431000	0.182614000
H	2.849486000	-5.434420000	1.313681000
H	3.568544000	-5.609251000	-0.294479000
H	4.564102000	-5.096890000	1.081672000
H	1.884466000	-0.138278000	2.654517000
H	3.415236000	-0.842159000	3.163061000
H	3.412763000	0.565690000	2.103061000
H	2.248283000	-0.527400000	-2.945902000
H	2.706158000	-2.183879000	-3.303576000
H	1.053574000	-1.818535000	-2.832618000
H	0.674966000	3.004625000	1.340152000

H	1.751641000	2.326010000	2.545521000
H	1.650264000	4.075581000	2.351626000
H	5.338151000	6.285157000	0.089289000
H	6.317453000	5.288510000	1.163221000
H	6.607983000	5.248686000	-0.583118000
H	3.486672000	1.140601000	-2.832483000
H	5.223767000	1.356356000	-2.654088000
H	4.395054000	0.123151000	-1.704518000
O	-1.082250000	1.418243000	1.426050000
H	-1.438968000	0.991985000	2.205236000
H	1.009597000	0.877728000	0.919684000

F4

	Coordinates (Angstroms)		
	X	Y	Z
B	-1.739665000	0.062242000	0.236226000
P	2.043408000	0.353968000	0.358063000
C	-1.631190000	-1.555871000	0.165327000
C	-1.438202000	-2.461288000	1.194936000
C	-1.666044000	-2.124922000	-1.104057000
C	-1.265545000	-3.823709000	0.992312000
C	-1.502556000	-3.472875000	-1.352883000
C	-1.295517000	-4.335151000	-0.289129000
C	-3.224397000	0.685907000	0.000251000
C	-3.420140000	2.066387000	-0.005122000
C	-4.376083000	-0.063063000	-0.221128000
C	-4.643913000	2.668271000	-0.235656000
C	-5.618104000	0.503238000	-0.464309000
C	-5.753902000	1.877595000	-0.474445000
F	-1.410166000	-2.065438000	2.481890000
F	-1.078012000	-4.639659000	2.029244000
F	-1.132244000	-5.637489000	-0.500552000
F	-1.537176000	-3.947835000	-2.597219000
F	-1.866011000	-1.334148000	-2.172236000
F	-2.397316000	2.896981000	0.245195000
F	-4.763779000	3.995099000	-0.225669000
F	-6.938099000	2.434296000	-0.699848000
F	-6.682731000	-0.268980000	-0.679062000
F	-4.349119000	-1.401751000	-0.207406000
C	-0.564748000	0.825843000	-0.574347000
H	-1.024788000	1.111872000	-1.527009000
H	-0.334558000	1.781889000	-0.098018000
C	0.730361000	0.058998000	-0.919310000
H	0.534453000	-1.007642000	-0.951470000
H	1.079359000	0.351815000	-1.910590000
C	3.537659000	-0.714377000	0.087527000
C	4.645257000	-0.380077000	0.899613000
C	3.634984000	-1.855213000	-0.742715000
C	5.822234000	-1.125824000	0.817537000
C	4.829529000	-2.571161000	-0.781320000
C	5.943149000	-2.220130000	-0.024776000
H	6.662660000	-0.839886000	1.443973000
H	4.889940000	-3.439907000	-1.431334000
C	2.591566000	2.052650000	-0.155111000
C	2.229731000	3.136510000	0.676037000
C	3.323401000	2.311957000	-1.331768000
C	2.589614000	4.432449000	0.315194000

C	3.662391000	3.628718000	-1.650248000
C	3.306818000	4.702725000	-0.845666000
H	2.301891000	5.256049000	0.962952000
H	4.221631000	3.814807000	-2.563040000
C	4.633079000	0.772342000	1.874040000
C	2.516952000	-2.378996000	-1.611226000
C	7.219970000	-3.011262000	-0.117265000
C	1.453002000	2.967251000	1.960274000
C	3.809955000	1.239617000	-2.275256000
C	3.689326000	6.114059000	-1.201331000
H	7.035177000	-4.077542000	0.041611000
H	7.673823000	-2.903581000	-1.107558000
H	7.948713000	-2.677052000	0.624183000
H	3.694325000	0.822238000	2.433095000
H	5.446431000	0.664898000	2.594253000
H	4.758763000	1.733327000	1.367827000
H	2.168266000	-1.637561000	-2.331826000
H	2.861500000	-3.248778000	-2.173073000
H	1.658105000	-2.698906000	-1.014463000
H	0.402308000	2.736297000	1.759407000
H	1.855334000	2.167191000	2.584330000
H	1.473301000	3.893028000	2.538621000
H	2.814397000	6.770465000	-1.199337000
H	4.400487000	6.517668000	-0.473671000
H	4.152647000	6.164711000	-2.188822000
H	3.073288000	0.455437000	-2.447062000
H	4.063208000	1.680130000	-3.241674000
H	4.708006000	0.755010000	-1.881932000
O	-1.594986000	0.507912000	1.930863000
H	-2.433501000	0.663415000	2.400653000
C	-0.539836000	0.406308000	2.826335000
H	0.383720000	0.148134000	2.291995000
O	-0.705344000	0.617167000	3.979512000

F7

	Coordinates (Angstroms)		
	X	Y	Z
B	-1.652914000	0.494299000	-0.005514000
P	1.588976000	0.844502000	-0.365356000
C	-1.100485000	-1.047282000	0.145534000
C	-0.622284000	-1.526481000	1.358227000
C	-0.946265000	-1.935324000	-0.910811000
C	0.012930000	-2.748934000	1.514430000
C	-0.324269000	-3.166416000	-0.805418000
C	0.170144000	-3.575230000	0.419887000
C	-3.283534000	0.646531000	-0.013039000
C	-3.840342000	1.893941000	0.249112000
C	-4.215822000	-0.349674000	-0.264185000
C	-5.200402000	2.148209000	0.273409000
C	-5.587997000	-0.142874000	-0.248319000
C	-6.087072000	1.116156000	0.022671000
F	-0.719806000	-0.781575000	2.477057000
F	0.496680000	-3.120773000	2.703583000
F	0.794830000	-4.746910000	0.538188000
F	-0.161142000	-3.949336000	-1.877581000
F	-1.394675000	-1.604063000	-2.138520000
F	-3.040093000	2.947792000	0.505709000

F	-5.666744000	3.373708000	0.536708000
F	-7.403265000	1.335226000	0.040174000
F	-6.432081000	-1.150312000	-0.495999000
F	-3.827400000	-1.607975000	-0.543677000
C	-1.022454000	1.279586000	-1.317334000
H	-1.638826000	1.112336000	-2.208169000
H	-1.063832000	2.358041000	-1.128919000
C	0.406184000	0.897131000	-1.752897000
H	0.391094000	-0.091615000	-2.199337000
H	0.820762000	1.582607000	-2.497352000
H	-1.280406000	1.068229000	1.002509000
H	0.790532000	0.904404000	0.771044000
C	2.492728000	-0.715651000	-0.142393000
C	2.883754000	-1.000777000	1.185548000
C	2.717160000	-1.659154000	-1.167018000
C	3.467795000	-2.232613000	1.462609000
C	3.299197000	-2.876032000	-0.825974000
C	3.672054000	-3.188697000	0.476153000
H	3.752873000	-2.452778000	2.485956000
H	3.457318000	-3.609308000	-1.610998000
C	2.603920000	2.342919000	-0.248635000
C	2.160489000	3.374874000	0.609233000
C	3.794762000	2.487128000	-0.984416000
C	2.941387000	4.520195000	0.720971000
C	4.538591000	3.653369000	-0.827670000
C	4.134814000	4.676777000	0.021800000
H	2.604828000	5.316563000	1.377583000
H	5.458493000	3.763774000	-1.393052000
C	2.659585000	-0.053664000	2.337619000
C	2.360193000	-1.473995000	-2.620657000
C	4.276135000	-4.527019000	0.792575000
C	0.886332000	3.302824000	1.413204000
C	4.284384000	1.449620000	-1.957202000
C	4.964205000	5.917926000	0.196154000
H	3.627414000	-5.332388000	0.438814000
H	5.243405000	-4.642640000	0.294414000
H	4.427019000	-4.652987000	1.865984000
H	1.596904000	0.015356000	2.588870000
H	3.178624000	-0.418232000	3.224423000
H	3.026904000	0.954931000	2.131167000
H	2.412761000	-0.440967000	-2.958224000
H	3.044142000	-2.057399000	-3.239171000
H	1.350929000	-1.845470000	-2.821173000
H	0.010684000	3.105776000	0.790196000
H	0.932725000	2.515924000	2.172588000
H	0.719322000	4.248977000	1.928796000
H	4.338403000	6.813795000	0.181782000
H	5.481503000	5.896085000	1.160676000
H	5.718508000	6.007377000	-0.587818000
H	3.548514000	1.282809000	-2.748653000
H	5.208045000	1.780183000	-2.433308000
H	4.479653000	0.491366000	-1.470500000

F8

	Coordinates (Angstroms)		
	X	Y	Z
B	-1.477125000	-0.129626000	0.241603000

P	2.342866000	-0.056188000	0.544093000
C	-2.212224000	-1.564071000	-0.008685000
C	-3.266522000	-1.951483000	0.810893000
C	-1.944138000	-2.436411000	-1.054927000
C	-3.982006000	-3.124410000	0.651048000
C	-2.636376000	-3.620617000	-1.254891000
C	-3.659924000	-3.970096000	-0.394876000
C	-2.582683000	1.082993000	0.131794000
C	-2.810041000	2.099488000	1.043392000
C	-3.335776000	1.186249000	-1.032677000
C	-3.713161000	3.131667000	0.857048000
C	-4.252129000	2.196656000	-1.268627000
C	-4.445525000	3.178752000	-0.313602000
F	-3.637586000	-1.163779000	1.838892000
F	-4.971540000	-3.443359000	1.486251000
F	-4.332622000	-5.103325000	-0.573968000
F	-2.330381000	-4.420082000	-2.277635000
F	-1.000771000	-2.161670000	-1.966341000
F	-2.121513000	2.136611000	2.218406000
F	-3.876365000	4.071800000	1.786546000
F	-5.318288000	4.160162000	-0.523346000
F	-4.942668000	2.236110000	-2.407276000
F	-3.185182000	0.281891000	-2.013776000
C	-0.199603000	0.278997000	-0.648130000
H	-0.559590000	0.364296000	-1.679236000
H	0.074899000	1.305832000	-0.380001000
C	1.052062000	-0.626435000	-0.668769000
H	0.784263000	-1.643937000	-0.396326000
H	1.461518000	-0.654224000	-1.677869000
C	3.936350000	-0.985874000	0.329330000
C	5.054321000	-0.418965000	0.982211000
C	4.097275000	-2.234704000	-0.312607000
C	6.298770000	-1.045211000	0.902592000
C	5.359835000	-2.822622000	-0.356921000
C	6.481687000	-2.240191000	0.223268000
H	7.145775000	-0.582160000	1.401365000
H	5.467246000	-3.778997000	-0.861337000
C	2.696181000	1.625163000	-0.150023000
C	2.207626000	2.735535000	0.573102000
C	3.375601000	1.842206000	-1.363441000
C	2.399130000	4.020310000	0.074521000
C	3.547665000	3.150862000	-1.821798000
C	3.068740000	4.251189000	-1.124254000
H	2.011453000	4.866047000	0.635872000
H	4.068767000	3.307314000	-2.762287000
C	4.985763000	0.860182000	1.780924000
C	2.974193000	-3.010610000	-0.957588000
C	7.834287000	-2.890806000	0.116295000
C	1.482160000	2.586706000	1.888295000
C	3.980321000	0.742404000	-2.201424000
C	3.256101000	5.651949000	-1.641504000
H	7.765197000	-3.971458000	0.266628000
H	8.266605000	-2.726400000	-0.876292000
H	8.530789000	-2.485322000	0.853600000
H	4.080016000	0.913537000	2.391673000
H	5.845235000	0.929033000	2.450686000
H	4.991438000	1.741717000	1.134259000

H	2.546545000	-2.488417000	-1.814942000
H	3.347146000	-3.972810000	-1.312801000
H	2.166015000	-3.213416000	-0.251044000
H	0.637791000	1.900801000	1.808235000
H	2.143822000	2.188929000	2.663891000
H	1.103469000	3.552929000	2.227629000
H	2.292064000	6.154506000	-1.763793000
H	3.848832000	6.250773000	-0.943256000
H	3.765852000	5.654789000	-2.607117000
H	3.356934000	-0.149278000	-2.253175000
H	4.147519000	1.097559000	-3.220300000
H	4.946176000	0.431795000	-1.792080000
O	-0.996523000	-0.172368000	1.807673000
H	-1.260797000	0.592763000	2.336253000
H	1.169774000	-1.402200000	2.144585000
C	-0.661460000	-1.317816000	2.665807000
H	-0.477471000	-0.862655000	3.642488000
H	-1.516868000	-1.987764000	2.702583000
O	0.399705000	-1.997356000	2.157111000

TSF2-3

	Coordinates (Angstroms)		
	X	Y	Z
B	-1.492618000	-0.590154000	0.145013000
P	1.685453000	-0.795443000	0.257634000
C	-1.191251000	1.037736000	0.089899000
C	-0.859570000	1.731146000	-1.065446000
C	-1.182689000	1.823099000	1.234765000
C	-0.485499000	3.063903000	-1.098016000
C	-0.817940000	3.157270000	1.256454000
C	-0.447413000	3.781503000	0.080099000
C	-3.083583000	-0.977434000	0.035585000
C	-3.463301000	-2.307483000	-0.130720000
C	-4.144389000	-0.084415000	0.114724000
C	-4.779587000	-2.727591000	-0.218033000
C	-5.476667000	-0.463811000	0.034804000
C	-5.797907000	-1.795890000	-0.133855000
F	-0.880884000	1.116025000	-2.273328000
F	-0.148860000	3.651133000	-2.247866000
F	-0.048975000	5.051914000	0.085676000
F	-0.778842000	3.835517000	2.406693000
F	-1.534205000	1.293049000	2.420432000
F	-2.539558000	-3.279716000	-0.213828000
F	-5.075865000	-4.019676000	-0.379288000
F	-7.070626000	-2.179814000	-0.213591000
F	-6.449027000	0.447804000	0.115560000
F	-3.936678000	1.232918000	0.278653000
C	-0.785055000	-1.329327000	1.425625000
H	-1.398389000	-1.210454000	2.323827000
H	-0.776428000	-2.402565000	1.207848000
C	0.649068000	-0.883320000	1.786925000
H	0.616921000	0.097121000	2.250692000
H	1.123836000	-1.561172000	2.501157000
C	2.349614000	0.883934000	-0.065695000
C	2.604263000	1.144264000	-1.435292000
C	2.470857000	1.931772000	0.867453000
C	2.922647000	2.432946000	-1.841040000

C	2.783224000	3.212056000	0.401619000
C	2.991305000	3.491573000	-0.939227000
H	3.101301000	2.620491000	-2.895515000
H	2.855038000	4.017599000	1.126707000
C	3.002711000	-2.071098000	0.265412000
C	2.789019000	-3.219527000	-0.531665000
C	4.201035000	-1.949904000	0.996068000
C	3.780817000	-4.194120000	-0.594388000
C	5.167673000	-2.948688000	0.889228000
C	4.982407000	-4.074052000	0.096397000
H	3.608217000	-5.075491000	-1.205057000
H	6.090913000	-2.841604000	1.450870000
C	2.511385000	0.073686000	-2.494697000
C	2.290133000	1.813688000	2.361587000
C	3.241596000	4.894137000	-1.417219000
C	1.531669000	-3.451739000	-1.332469000
C	4.479327000	-0.799851000	1.923070000
C	6.047147000	-5.128596000	-0.028461000
H	2.337607000	5.297158000	-1.884314000
H	3.514500000	5.554536000	-0.591768000
H	4.037773000	4.923073000	-2.165443000
H	1.472169000	-0.221264000	-2.666356000
H	2.906222000	0.447787000	-3.440501000
H	3.073962000	-0.825253000	-2.230569000
H	2.485331000	0.819374000	2.750203000
H	2.971523000	2.503895000	2.863414000
H	1.275770000	2.094634000	2.659743000
H	0.632134000	-3.420048000	-0.714608000
H	1.412981000	-2.700871000	-2.118648000
H	1.572463000	-4.430456000	-1.812977000
H	5.618392000	-6.130857000	0.051687000
H	6.540152000	-5.060086000	-1.003492000
H	6.811860000	-5.016450000	0.742800000
H	3.739400000	-0.767643000	2.726806000
H	5.461792000	-0.910340000	2.384024000
H	4.455013000	0.160189000	1.403927000
O	-0.783080000	-1.237425000	-1.075916000
H	-1.002479000	-0.824302000	-1.917292000
H	0.393373000	-1.092777000	-0.792391000

TSF3-4

	Coordinates (Angstroms)		
	X	Y	Z
B	-1.627515000	0.061106000	0.245515000
P	2.062596000	0.316147000	0.101955000
C	-1.769032000	-1.575342000	0.053712000
C	-1.941564000	-2.471357000	1.099185000
C	-1.716916000	-2.176990000	-1.199174000
C	-2.000929000	-3.847868000	0.940823000
C	-1.768496000	-3.543284000	-1.407980000
C	-1.904491000	-4.391969000	-0.324134000
C	-3.063979000	0.859365000	0.076951000
C	-3.130344000	2.225055000	0.345170000
C	-4.261999000	0.304752000	-0.355053000
C	-4.277594000	2.986002000	0.202246000
C	-5.432993000	1.031798000	-0.515854000
C	-5.442893000	2.383532000	-0.235434000

F	-2.097258000	-2.037834000	2.367964000
F	-2.160982000	-4.647650000	1.997812000
F	-1.956839000	-5.710776000	-0.500585000
F	-1.694591000	-4.049259000	-2.641239000
F	-1.631000000	-1.424355000	-2.311089000
F	-2.043021000	2.893580000	0.768229000
F	-4.270188000	4.292509000	0.478188000
F	-6.557141000	3.097408000	-0.383795000
F	-6.550505000	0.434381000	-0.936962000
F	-4.361355000	-1.002090000	-0.651323000
C	-0.543127000	0.721502000	-0.785305000
H	-1.061435000	0.875237000	-1.735726000
H	-0.297210000	1.731631000	-0.441796000
C	0.750200000	-0.052445000	-1.120334000
H	0.580315000	-1.127590000	-1.082967000
H	1.093118000	0.190449000	-2.126461000
C	3.506175000	-0.819778000	0.065849000
C	4.440536000	-0.603075000	1.104298000
C	3.720533000	-1.880287000	-0.841829000
C	5.580323000	-1.400008000	1.183088000
C	4.873376000	-2.652305000	-0.710585000
C	5.822403000	-2.426121000	0.279985000
H	6.292559000	-1.213503000	1.981235000
H	5.034500000	-3.461907000	-1.416314000
C	2.632719000	2.017056000	-0.282645000
C	2.220598000	3.065109000	0.568823000
C	3.434344000	2.304404000	-1.404135000
C	2.608259000	4.368691000	0.275081000
C	3.799785000	3.627382000	-1.652166000
C	3.398600000	4.672460000	-0.829852000
H	2.283064000	5.171189000	0.930868000
H	4.414237000	3.843418000	-2.521207000
C	4.257492000	0.463398000	2.155751000
C	2.777142000	-2.270156000	-1.952199000
C	7.071791000	-3.259708000	0.356463000
C	1.384999000	2.835625000	1.803888000
C	3.939832000	1.249041000	-2.352560000
C	3.810627000	6.091730000	-1.109097000
H	6.855452000	-4.315413000	0.174784000
H	7.793427000	-2.938122000	-0.401552000
H	7.551439000	-3.167282000	1.333135000
H	3.291647000	0.364235000	2.660191000
H	5.035689000	0.381334000	2.915938000
H	4.310549000	1.469984000	1.732354000
H	2.476455000	-1.425043000	-2.569617000
H	3.256488000	-3.001641000	-2.604505000
H	1.869229000	-2.734048000	-1.556560000
H	0.422941000	2.372812000	1.573121000
H	1.907476000	2.196727000	2.522250000
H	1.178031000	3.785001000	2.300512000
H	2.947803000	6.762852000	-1.083020000
H	4.519427000	6.442932000	-0.352529000
H	4.288390000	6.183476000	-2.086456000
H	3.148097000	0.571868000	-2.675626000
H	4.362743000	1.713886000	-3.244571000
H	4.720465000	0.641109000	-1.887778000
O	-1.199126000	0.353994000	1.709213000

H	-1.918851000	0.192880000	2.330825000
H	1.189565000	0.078197000	1.577222000
C	0.295750000	-0.112141000	2.580753000
O	0.090650000	-0.302750000	3.694558000

TSF1-7

	Coordinates (Angstroms)		
	X	Y	Z
C	-1.493102000	-1.603571000	1.134443000
C	-1.270586000	-1.062100000	-0.124052000
C	-0.886731000	-1.974687000	-1.095429000
C	-0.679842000	-3.318477000	-0.831791000
C	-0.879890000	-3.800474000	0.447658000
C	-1.295411000	-2.935942000	1.443051000
B	-1.413295000	0.534818000	-0.349244000
C	-2.912769000	1.114318000	-0.167110000
C	-3.221612000	2.233966000	0.589369000
C	-4.502820000	2.746002000	0.700795000
C	-5.538031000	2.129189000	0.020960000
C	-5.274565000	1.016173000	-0.757723000
C	-3.978702000	0.534675000	-0.838973000
F	-2.250948000	2.873768000	1.266207000
F	-4.746697000	3.821896000	1.449391000
F	-6.776652000	2.607229000	0.109852000
F	-6.265992000	0.424651000	-1.424880000
F	-3.775894000	-0.534318000	-1.624912000
F	-0.691058000	-1.584858000	-2.364024000
F	-0.268602000	-4.146075000	-1.793769000
F	-0.642764000	-5.078952000	0.728053000
F	-1.457801000	-3.383640000	2.688806000
F	-1.881006000	-0.800326000	2.141804000
C	-0.623730000	1.257681000	-1.548875000
C	0.882173000	0.950453000	-1.679125000
P	1.701801000	0.819216000	-0.012929000
C	2.256071000	-0.930400000	0.049120000
C	1.970134000	-1.647876000	1.227079000
C	2.165834000	-3.026796000	1.247308000
C	2.633612000	-3.718020000	0.135031000
C	2.978401000	-2.982446000	-0.993985000
C	2.818204000	-1.600812000	-1.053708000
C	3.103565000	2.018549000	0.029896000
C	2.751221000	3.376789000	-0.156793000
C	3.737435000	4.357107000	-0.129680000
C	5.073423000	4.052110000	0.103775000
C	5.393983000	2.724361000	0.349677000
C	4.444408000	1.699873000	0.334226000
H	1.396212000	1.687321000	-2.299158000
H	1.000173000	-0.011867000	-2.168685000
H	-0.780859000	2.334959000	-1.435064000
H	-1.113025000	0.986892000	-2.490389000
H	3.446470000	5.393068000	-0.280532000
H	6.424753000	2.467583000	0.576041000
H	1.926965000	-3.577511000	2.152605000
H	3.395076000	-3.496655000	-1.855971000
H	-0.984527000	1.009608000	0.891857000
H	-0.157382000	0.983226000	0.665954000
C	1.329018000	3.832051000	-0.370130000

C	4.945600000	0.319092000	0.683072000
C	6.121162000	5.131511000	0.115365000
C	1.500830000	-0.971392000	2.490826000
C	3.319121000	-0.897115000	-2.292036000
C	2.744784000	-5.217415000	0.136783000
H	0.557897000	-0.439514000	2.354793000
H	1.359817000	-1.706328000	3.285559000
H	2.233597000	-0.236066000	2.838106000
H	1.960331000	-5.652765000	-0.489731000
H	3.705729000	-5.545389000	-0.268599000
H	2.634429000	-5.625347000	1.143376000
H	3.455145000	0.174426000	-2.141551000
H	4.284067000	-1.318639000	-2.584234000
H	2.637811000	-1.036956000	-3.136555000
H	4.965964000	-0.344132000	-0.183445000
H	5.964111000	0.388703000	1.069158000
H	4.332854000	-0.161044000	1.447051000
H	0.619635000	3.268471000	0.242334000
H	1.229165000	4.886381000	-0.106551000
H	1.022929000	3.725336000	-1.414024000
H	5.844624000	5.937773000	0.800783000
H	7.092445000	4.738926000	0.423076000
H	6.234680000	5.574588000	-0.878942000

TSF7

	Coordinates (Angstroms)		
	X	Y	Z
B	1.554215000	-0.585052000	-0.223931000
P	-1.877170000	-0.792759000	0.108354000
C	1.208812000	0.994283000	-0.350061000
C	0.916742000	1.846737000	0.704138000
C	1.171147000	1.583733000	-1.605108000
C	0.626978000	3.189598000	0.539284000
C	0.844874000	2.911227000	-1.819899000
C	0.573857000	3.722615000	-0.734524000
C	3.137359000	-0.919264000	-0.153046000
C	3.745851000	-2.000740000	-0.782604000
C	4.006366000	-0.137574000	0.604393000
C	5.102541000	-2.271883000	-0.700485000
C	5.365084000	-0.375464000	0.716116000
C	5.920977000	-1.453481000	0.053569000
F	0.895823000	1.389519000	1.965477000
F	0.374693000	3.967244000	1.593433000
F	0.237809000	4.998028000	-0.915917000
F	0.767432000	3.405432000	-3.057821000
F	1.436958000	0.845884000	-2.699345000
F	3.037122000	-2.867272000	-1.522071000
F	5.623547000	-3.322323000	-1.338138000
F	7.223923000	-1.703410000	0.146486000
F	6.137070000	0.417275000	1.461307000
F	3.542224000	0.906010000	1.307881000
C	0.614327000	-1.566806000	-1.084870000
H	1.082597000	-1.716724000	-2.062152000
H	0.606621000	-2.560093000	-0.626720000
C	-0.816931000	-1.099715000	-1.376269000
H	-0.774571000	-0.176667000	-1.944323000
H	-1.347256000	-1.819264000	-2.004784000

H	1.191112000	-0.865157000	1.072410000
H	-0.988095000	-1.265625000	1.391150000
C	-2.280226000	0.987323000	0.209101000
C	-2.372980000	1.545332000	1.504763000
C	-2.478423000	1.805374000	-0.916600000
C	-2.571952000	2.912367000	1.638219000
C	-2.660229000	3.175663000	-0.727485000
C	-2.687257000	3.752911000	0.532997000
H	-2.633764000	3.337610000	2.635272000
H	-2.794926000	3.804833000	-1.602579000
C	-3.379990000	-1.854759000	-0.038864000
C	-3.136660000	-3.250430000	-0.042606000
C	-4.713373000	-1.391834000	-0.101710000
C	-4.202901000	-4.136120000	-0.149642000
C	-5.743411000	-2.329840000	-0.207923000
C	-5.518165000	-3.697965000	-0.243954000
H	-3.996281000	-5.202489000	-0.151015000
H	-6.764779000	-1.965250000	-0.259753000
C	-2.320739000	0.706192000	2.755793000
C	-2.587928000	1.305582000	-2.337008000
C	-2.827615000	5.237954000	0.712964000
C	-1.756844000	-3.851716000	0.054919000
C	-5.144542000	0.054420000	-0.056445000
C	-6.648481000	-4.680363000	-0.379031000
H	-1.910945000	5.654039000	1.140932000
H	-3.014953000	5.738923000	-0.238561000
H	-3.645137000	5.476780000	1.398877000
H	-1.328515000	0.286623000	2.927957000
H	-2.589105000	1.311598000	3.622963000
H	-3.023558000	-0.131935000	2.706694000
H	-2.882376000	0.257193000	-2.401689000
H	-3.342681000	1.891230000	-2.866153000
H	-1.646107000	1.434578000	-2.880600000
H	-1.234693000	-3.807569000	-0.904451000
H	-1.138890000	-3.350660000	0.801910000
H	-1.828714000	-4.902763000	0.338208000
H	-6.618507000	-5.168367000	-1.358249000
H	-6.576553000	-5.465607000	0.378407000
H	-7.618182000	-4.189754000	-0.275257000
H	-4.796576000	0.612940000	-0.926025000
H	-6.234261000	0.106690000	-0.047861000
H	-4.781089000	0.568797000	0.833597000
C	1.043868000	-1.600827000	2.134092000
O	-0.231021000	-1.723889000	2.329291000
O	1.754215000	-2.743225000	1.904602000
H	1.619420000	-0.949617000	2.800980000
H	1.141435000	-3.393414000	1.539128000

TSF8

	Coordinates (Angstroms)		
	X	Y	Z
B	-1.533149000	-0.034247000	0.185386000
P	2.252748000	0.008068000	0.107151000
C	-2.071185000	-1.586483000	0.092365000
C	-2.861511000	-2.113198000	1.106249000
C	-1.848957000	-2.460423000	-0.962137000
C	-3.338087000	-3.411563000	1.129110000

C	-2.303405000	-3.769648000	-0.985867000
C	-3.047367000	-4.254401000	0.072529000
C	-2.763966000	1.050233000	-0.045023000
C	-2.651060000	2.375615000	0.365626000
C	-3.931201000	0.771215000	-0.746916000
C	-3.613974000	3.344111000	0.136445000
C	-4.919458000	1.710002000	-1.000689000
C	-4.763475000	3.007347000	-0.552557000
F	-3.243684000	-1.333551000	2.141330000
F	-4.080344000	-3.851837000	2.147462000
F	-3.491571000	-5.509850000	0.068017000
F	-2.039745000	-4.561835000	-2.027883000
F	-1.195687000	-2.061807000	-2.068806000
F	-1.554936000	2.797186000	1.024774000
F	-3.439028000	4.595528000	0.566440000
F	-5.702407000	3.921157000	-0.788357000
F	-6.017970000	1.371960000	-1.679383000
F	-4.164711000	-0.452332000	-1.249209000
C	-0.356020000	0.410484000	-0.847446000
H	-0.835008000	0.480954000	-1.830027000
H	-0.079753000	1.444448000	-0.605817000
C	0.922626000	-0.427187000	-1.064494000
H	0.741176000	-1.488361000	-0.894052000
H	1.269937000	-0.314230000	-2.088923000
C	3.782522000	-0.994615000	-0.057815000
C	4.704589000	-0.831959000	1.000618000
C	4.065321000	-1.925884000	-1.082627000
C	5.891977000	-1.560535000	0.996464000
C	5.262764000	-2.636032000	-1.029193000
C	6.193779000	-2.466799000	-0.010105000
H	6.592747000	-1.418899000	1.813582000
H	5.473950000	-3.350993000	-1.818851000
C	2.645402000	1.782323000	-0.096463000
C	2.237646000	2.682816000	0.912301000
C	3.312463000	2.265455000	-1.238768000
C	2.517785000	4.036838000	0.758860000
C	3.574288000	3.631000000	-1.340227000
C	3.187990000	4.531930000	-0.355834000
H	2.197695000	4.727420000	1.533495000
H	4.089009000	3.998579000	-2.222815000
C	4.456675000	0.095522000	2.162893000
C	3.163633000	-2.217310000	-2.255676000
C	7.482777000	-3.241421000	-0.009738000
C	1.497087000	2.247205000	2.149727000
C	3.740726000	1.379034000	-2.378270000
C	3.476435000	6.002601000	-0.482130000
H	7.303253000	-4.302099000	-0.204113000
H	8.153475000	-2.874534000	-0.793257000
H	8.000969000	-3.148800000	0.946799000
H	3.519627000	-0.150020000	2.671226000
H	5.261334000	0.009084000	2.894240000
H	4.402099000	1.141149000	1.848679000
H	2.972143000	-1.325019000	-2.853214000
H	3.635296000	-2.953464000	-2.907999000
H	2.201729000	-2.626039000	-1.941847000
H	0.565628000	1.739330000	1.897088000
H	2.096242000	1.566533000	2.760158000

H	1.249760000	3.115264000	2.763189000
H	2.553732000	6.585837000	-0.413044000
H	4.135608000	6.338462000	0.324054000
H	3.958213000	6.233550000	-1.434034000
H	2.878286000	0.903389000	-2.852597000
H	4.252961000	1.965174000	-3.142540000
H	4.420270000	0.590752000	-2.048098000
O	-1.072428000	0.196847000	1.669238000
H	-1.794787000	0.552484000	2.198260000
H	1.569162000	-0.482513000	1.436437000
C	-0.203149000	-0.829760000	2.724537000
H	-0.026137000	-0.046219000	3.474999000
H	-1.000445000	-1.523577000	3.012288000
O	0.800562000	-1.340354000	2.123084000

TSF9

	Coordinates (Angstroms)		
	X	Y	Z
B	-1.565088000	0.017543000	-0.166536000
P	1.971457000	0.292773000	0.048418000
C	-1.832717000	-1.565944000	-0.378679000
C	-1.047536000	-2.571207000	0.165559000
C	-2.824154000	-2.001160000	-1.247156000
C	-1.224210000	-3.915348000	-0.116456000
C	-3.042833000	-3.334566000	-1.548986000
C	-2.234722000	-4.301312000	-0.977419000
C	-2.884567000	0.891863000	0.149854000
C	-3.170074000	2.129897000	-0.413292000
C	-3.832490000	0.439341000	1.064647000
C	-4.302125000	2.867095000	-0.101758000
C	-4.972567000	1.144683000	1.405341000
C	-5.210089000	2.372608000	0.814241000
F	-0.034381000	-2.265668000	0.993678000
F	-0.419513000	-4.834043000	0.420319000
F	-2.420902000	-5.588078000	-1.262632000
F	-4.012528000	-3.693905000	-2.391510000
F	-3.619046000	-1.108399000	-1.861329000
F	-2.353261000	2.686472000	-1.320557000
F	-4.521997000	4.048765000	-0.680394000
F	-6.299397000	3.068918000	1.125535000
F	-5.839559000	0.656159000	2.292733000
F	-3.665832000	-0.744435000	1.676979000
C	-0.480111000	0.586407000	-1.215498000
H	-0.959844000	0.518822000	-2.201666000
H	-0.272632000	1.648617000	-1.077330000
C	0.835109000	-0.199034000	-1.305237000
H	0.651055000	-1.268375000	-1.247434000
H	1.344181000	-0.006316000	-2.251568000
H	-0.937063000	0.063586000	1.076454000
H	1.217021000	0.357653000	1.401191000
C	3.378868000	-0.825729000	0.392193000
C	4.189369000	-0.422630000	1.476428000
C	3.667886000	-2.027813000	-0.287740000
C	5.294991000	-1.197204000	1.822388000
C	4.781222000	-2.764886000	0.105806000
C	5.614312000	-2.366498000	1.146619000
H	5.916011000	-0.874526000	2.652447000

H	5.002448000	-3.689209000	-0.419890000
C	2.612049000	1.931915000	-0.459012000
C	2.067532000	3.094233000	0.131372000
C	3.616103000	2.046905000	-1.439562000
C	2.542175000	4.337229000	-0.273738000
C	4.056492000	3.318360000	-1.806850000
C	3.536852000	4.473343000	-1.238281000
H	2.119993000	5.229325000	0.179540000
H	4.829554000	3.401320000	-2.564962000
C	3.919292000	0.817386000	2.293418000
C	2.835926000	-2.598054000	-1.407997000
C	6.818877000	-3.185562000	1.520474000
C	0.992085000	3.059425000	1.187286000
C	4.272421000	0.865419000	-2.107040000
C	4.025673000	5.836761000	-1.642736000
H	6.548777000	-4.234370000	1.670694000
H	7.569172000	-3.153211000	0.724332000
H	7.282311000	-2.815379000	2.436890000
H	2.896094000	0.835959000	2.678699000
H	4.592617000	0.850005000	3.150986000
H	4.076968000	1.730467000	1.713388000
H	2.661172000	-1.882590000	-2.212583000
H	3.339968000	-3.462978000	-1.841039000
H	1.865816000	-2.939294000	-1.036206000
H	0.072399000	2.605399000	0.809491000
H	1.296286000	2.485800000	2.064959000
H	0.750530000	4.073510000	1.508741000
H	3.199400000	6.453529000	-2.007811000
H	4.466815000	6.357221000	-0.787429000
H	4.780201000	5.773732000	-2.428991000
H	3.553662000	0.109591000	-2.426152000
H	4.820804000	1.192823000	-2.991629000
H	4.981535000	0.377536000	-1.433137000
C	-0.731290000	0.300882000	2.329994000
O	0.530612000	0.185724000	2.588351000
H	-1.403010000	-0.464932000	2.741680000
H	-1.182313000	1.308256000	2.353435000

G1

	Coordinates (Angstroms)		
	X	Y	Z
B	1.007219000	0.182233000	-0.905780000
P	-1.926756000	-0.737731000	0.136357000
C	0.704883000	1.643597000	-0.384341000
C	0.821491000	1.965372000	0.962300000
C	0.261712000	2.660062000	-1.220323000
C	0.499022000	3.213949000	1.463041000
C	-0.053398000	3.925046000	-0.758524000
C	0.062448000	4.199002000	0.593686000
C	2.361218000	-0.497358000	-0.478743000
C	2.420365000	-1.806960000	-0.014643000
C	3.571167000	0.185058000	-0.543346000
C	3.605140000	-2.407645000	0.372322000
C	4.775802000	-0.390157000	-0.182494000
C	4.788617000	-1.694486000	0.282218000
F	1.222850000	1.040214000	1.840555000
F	0.601967000	3.476278000	2.763777000

F	-0.244391000	5.404649000	1.055763000
F	-0.466810000	4.874485000	-1.594905000
F	0.134816000	2.439400000	-2.534986000
F	1.303479000	-2.529720000	0.112580000
F	3.619057000	-3.657371000	0.829972000
F	5.933283000	-2.260018000	0.641363000
F	5.914725000	0.292261000	-0.276243000
F	3.606221000	1.443984000	-0.994630000
C	0.005161000	-0.539603000	-1.868913000
H	0.271045000	-0.156467000	-2.868204000
H	0.197952000	-1.614089000	-1.912322000
C	-1.478239000	-0.261191000	-1.597527000
H	-1.686788000	0.810280000	-1.659630000
H	-2.129637000	-0.743265000	-2.331716000
C	-2.177663000	-2.556797000	0.053112000
C	-2.134831000	-3.313186000	-1.119268000
C	-2.396527000	-3.215115000	1.268036000
C	-2.314284000	-4.692951000	-1.077805000
H	-1.961546000	-2.836628000	-2.077259000
C	-2.590012000	-4.589366000	1.308318000
H	-2.416367000	-2.645173000	2.192430000
C	-2.546504000	-5.333182000	0.133036000
H	-2.274710000	-5.266043000	-1.997853000
H	-2.764213000	-5.081586000	2.258817000
H	-2.688686000	-6.407857000	0.163211000
C	-3.652371000	-0.101132000	0.211565000
C	-4.758123000	-0.787386000	-0.295310000
C	-3.840349000	1.163415000	0.775653000
C	-6.024739000	-0.217386000	-0.240732000
H	-4.631814000	-1.772487000	-0.732025000
C	-5.106121000	1.736885000	0.823075000
H	-2.989549000	1.700821000	1.184726000
C	-6.200838000	1.045462000	0.316380000
H	-6.876997000	-0.759986000	-0.635287000
H	-5.238292000	2.718498000	1.264816000
H	-7.190102000	1.487646000	0.359986000

G1_CO

	Coordinates (Angstroms)		
	X	Y	Z
B	-1.060288000	-0.301269000	-0.001744000
P	2.186034000	-0.805874000	-0.141176000
C	-0.587920000	1.239879000	-0.212133000
C	-0.234895000	1.786178000	-1.434800000
C	-0.490514000	2.107195000	0.867240000
C	0.219087000	3.084309000	-1.594850000
C	-0.038165000	3.409414000	0.756890000
C	0.324414000	3.901603000	-0.484945000
C	-2.681099000	-0.554489000	0.055736000
C	-3.196372000	-1.846764000	0.078824000
C	-3.634212000	0.453376000	0.149229000
C	-4.541644000	-2.140594000	0.200601000
C	-4.991601000	0.200139000	0.278561000
C	-5.450305000	-1.103128000	0.305506000
F	-0.312969000	1.035983000	-2.550259000
F	0.563749000	3.543281000	-2.796863000
F	0.771131000	5.147698000	-0.608077000

F	0.049992000	4.192625000	1.831093000
F	-0.854452000	1.698143000	2.091494000
F	-2.364096000	-2.896628000	-0.044648000
F	-4.966128000	-3.402823000	0.211958000
F	-6.748514000	-1.356745000	0.425202000
F	-5.858382000	1.207494000	0.373504000
F	-3.288465000	1.746098000	0.124945000
C	-0.277200000	-1.070145000	1.229467000
H	-0.828483000	-0.822302000	2.141495000
H	-0.363379000	-2.154816000	1.119277000
C	1.194321000	-0.685982000	1.418014000
H	1.278688000	0.361276000	1.716367000
H	1.662211000	-1.264741000	2.220464000
C	2.860619000	-2.511419000	-0.141009000
C	2.437281000	-3.509023000	0.738086000
C	3.788562000	-2.841060000	-1.136319000
C	2.942363000	-4.802488000	0.634670000
H	1.709846000	-3.289770000	1.511115000
C	4.303118000	-4.126410000	-1.228810000
H	4.118008000	-2.081856000	-1.839517000
C	3.878612000	-5.112992000	-0.342772000
H	2.602615000	-5.566153000	1.325783000
H	5.030735000	-4.361524000	-1.997708000
H	4.275011000	-6.119453000	-0.419143000
C	3.628359000	0.235113000	0.317596000
C	4.777904000	-0.248533000	0.943706000
C	3.516955000	1.605704000	0.066998000
C	5.795510000	0.625112000	1.311996000
H	4.883160000	-1.309170000	1.144572000
C	4.527324000	2.479681000	0.448730000
H	2.630808000	1.992030000	-0.427757000
C	5.671329000	1.989045000	1.069384000
H	6.686059000	0.237945000	1.795039000
H	4.423026000	3.541439000	0.253661000
H	6.464701000	2.668035000	1.361747000
C	-0.626382000	-1.146263000	-1.312142000
O	-0.460194000	-1.872170000	-2.150612000

G2

	Coordinates (Angstroms)		
	X	Y	Z
B	1.151442000	0.605003000	-0.054674000
P	-2.078240000	1.394286000	-0.307960000
C	0.591789000	-0.932818000	0.010214000
C	0.238895000	-1.706784000	-1.081787000
C	0.380385000	-1.551556000	1.236733000
C	-0.337086000	-2.962645000	-0.992577000
C	-0.196055000	-2.799161000	1.379994000
C	-0.570464000	-3.508332000	0.252829000
C	2.776289000	0.749386000	-0.053936000
C	3.373540000	1.985343000	-0.292284000
C	3.670185000	-0.280791000	0.212601000
C	4.741610000	2.193807000	-0.276766000
C	5.046341000	-0.111095000	0.244744000
C	5.588007000	1.134814000	-0.002323000
F	0.430223000	-1.247544000	-2.343509000
F	-0.684096000	-3.632789000	-2.091018000

F	-1.161730000	-4.694159000	0.368657000
F	-0.415856000	-3.315571000	2.590104000
F	0.742393000	-0.933886000	2.374359000
F	2.623325000	3.065511000	-0.560137000
F	5.249795000	3.403641000	-0.517965000
F	6.905621000	1.314738000	0.021920000
F	5.850092000	-1.142914000	0.508867000
F	3.242422000	-1.528092000	0.460676000
C	0.427067000	1.596540000	1.006040000
H	0.941858000	1.518077000	1.968465000
H	0.594014000	2.625446000	0.669729000
C	-1.081314000	1.407161000	1.264125000
H	-1.268646000	0.490653000	1.825541000
H	-1.456848000	2.230514000	1.877161000
O	0.713768000	1.230907000	-1.497808000
H	0.874009000	0.655683000	-2.259364000
H	-0.253702000	1.468953000	-1.482704000
C	-2.790263000	-0.297810000	-0.439345000
C	-3.155673000	-1.076621000	0.660459000
C	-2.984136000	-0.812785000	-1.723447000
C	-3.698953000	-2.342525000	0.478967000
H	-3.016835000	-0.702522000	1.668882000
C	-3.533243000	-2.077047000	-1.905688000
H	-2.693772000	-0.224710000	-2.588563000
C	-3.887878000	-2.846040000	-0.803748000
H	-3.965772000	-2.941016000	1.343119000
H	-3.668552000	-2.467074000	-2.908086000
H	-4.302146000	-3.838239000	-0.942369000
C	-3.559308000	2.401585000	0.092515000
C	-4.027081000	3.323261000	-0.843874000
C	-4.242088000	2.253298000	1.303568000
C	-5.162691000	4.083520000	-0.576628000
H	-3.503982000	3.449317000	-1.786286000
C	-5.371813000	3.013754000	1.570431000
H	-3.892323000	1.539057000	2.041786000
C	-5.834517000	3.929888000	0.629184000
H	-5.518091000	4.797368000	-1.311442000
H	-5.893427000	2.891271000	2.513209000
H	-6.718508000	4.522164000	0.838538000

G3

	Coordinates (Angstroms)		
	X	Y	Z
B	-1.098831000	0.721579000	0.250001000
P	2.157089000	1.111450000	-0.034024000
C	-0.511936000	-0.842907000	0.143652000
C	0.107480000	-1.541538000	1.168614000
C	-0.557374000	-1.534267000	-1.059849000
C	0.678210000	-2.797050000	1.016399000
C	0.008103000	-2.778361000	-1.267859000
C	0.641417000	-3.416091000	-0.215594000
C	-2.765607000	0.710774000	0.144130000
C	-3.465562000	1.879679000	-0.136580000
C	-3.581828000	-0.383860000	0.399786000
C	-4.846522000	1.965695000	-0.195950000
C	-4.968259000	-0.346642000	0.351344000
C	-5.608363000	0.837939000	0.047508000

F	0.212062000	-1.018588000	2.409054000
F	1.278379000	-3.401495000	2.045890000
F	1.217448000	-4.604925000	-0.395458000
F	-0.033841000	-3.363885000	-2.468678000
F	-1.165733000	-0.976289000	-2.127113000
F	-2.811004000	3.033589000	-0.366272000
F	-5.450442000	3.124097000	-0.481001000
F	-6.939588000	0.896208000	-0.003997000
F	-5.690070000	-1.443878000	0.602508000
F	-3.061079000	-1.580519000	0.735116000
C	-0.429383000	1.633049000	-0.956489000
H	-1.044278000	1.634151000	-1.861498000
H	-0.410837000	2.667376000	-0.600070000
C	0.991195000	1.260328000	-1.422058000
H	0.995207000	0.305562000	-1.949943000
H	1.412426000	2.005829000	-2.102174000
O	-0.695017000	1.375603000	1.513731000
H	-1.016291000	0.890795000	2.274421000
H	1.422839000	1.194935000	1.159156000
C	3.322138000	2.482947000	0.006503000
C	4.348363000	2.563869000	-0.937172000
C	3.167007000	3.474975000	0.974324000
C	5.218666000	3.643454000	-0.908076000
H	4.468355000	1.785999000	-1.684786000
C	4.044658000	4.552696000	0.995276000
H	2.369194000	3.407804000	1.706076000
C	5.066251000	4.635780000	0.057037000
H	6.017635000	3.710444000	-1.637192000
H	3.929516000	5.324748000	1.747012000
H	5.751049000	5.476085000	0.077309000
C	3.030224000	-0.464801000	-0.029732000
C	3.176755000	-1.223204000	-1.189909000
C	3.534076000	-0.930716000	1.187184000
C	3.815672000	-2.455694000	-1.126798000
H	2.792879000	-0.869643000	-2.139622000
C	4.170388000	-2.161639000	1.240081000
H	3.410815000	-0.345737000	2.092268000
C	4.304948000	-2.926253000	0.085140000
H	3.916509000	-3.052359000	-2.025604000
H	4.546237000	-2.532663000	2.185957000
H	4.787220000	-3.895602000	0.133286000

G4

	Coordinates (Angstroms)		
	X	Y	Z
B	1.165255000	-0.305469000	0.151962000
P	-2.494753000	-0.382769000	0.164256000
C	0.776571000	1.241910000	0.432279000
C	0.329044000	1.831566000	1.602386000
C	0.820858000	2.092113000	-0.666164000
C	-0.058089000	3.161603000	1.683115000
C	0.447425000	3.420474000	-0.633049000
C	-0.000495000	3.962111000	0.559771000
C	2.731716000	-0.595210000	-0.182344000
C	3.149546000	-1.870112000	-0.562004000
C	3.750003000	0.351531000	-0.116070000
C	4.456569000	-2.185917000	-0.887160000

C	5.069108000	0.076275000	-0.442459000
C	5.425267000	-1.200361000	-0.831961000
F	0.235695000	1.133815000	2.749970000
F	-0.487160000	3.669349000	2.838229000
F	-0.368992000	5.238349000	0.621585000
F	0.511140000	4.177727000	-1.727508000
F	1.252264000	1.609796000	-1.846453000
F	2.273536000	-2.883308000	-0.605496000
F	4.789764000	-3.425686000	-1.243494000
F	6.685820000	-1.481332000	-1.139965000
F	5.996576000	1.030434000	-0.373368000
F	3.508156000	1.606735000	0.282092000
C	0.104684000	-1.090326000	-0.792961000
H	0.624197000	-1.292532000	-1.735535000
H	-0.100349000	-2.085105000	-0.381275000
C	-1.220314000	-0.407574000	-1.176136000
H	-1.044523000	0.631923000	-1.459592000
H	-1.642743000	-0.896554000	-2.058845000
C	-3.928959000	0.426352000	-0.663304000
C	-5.177360000	0.329916000	-0.037321000
C	-3.828195000	1.166428000	-1.842424000
C	-6.296400000	0.940337000	-0.585068000
C	-4.950721000	1.785234000	-2.387339000
C	-6.186346000	1.672112000	-1.764537000
H	-7.256719000	0.846508000	-0.089884000
H	-4.854255000	2.352152000	-3.307025000
C	-3.061765000	-2.142301000	0.155765000
C	-3.136622000	-2.833874000	1.365664000
C	-3.430382000	-2.803888000	-1.020101000
C	-3.558093000	-4.160970000	1.402623000
C	-3.848771000	-4.126655000	-0.985851000
C	-3.910859000	-4.809268000	0.226921000
H	-3.611534000	-4.682996000	2.351571000
H	-4.131515000	-4.626838000	-1.905765000
O	1.144406000	-1.127422000	1.714210000
H	2.001690000	-1.280217000	2.149665000
C	0.088518000	-1.518568000	2.511179000
H	-0.856501000	-1.242513000	2.037865000
O	0.266537000	-2.065160000	3.545833000
H	-3.401527000	-2.279413000	-1.969782000
H	-2.872118000	-2.334576000	2.292982000
H	-4.239090000	-5.842605000	0.252289000
H	-5.276422000	-0.234745000	0.885072000
H	-7.059827000	2.150892000	-2.193114000
H	-2.878103000	1.265627000	-2.353666000

G7

	Coordinates (Angstroms)		
	X	Y	Z
B	-0.820020000	0.684737000	-0.154631000
P	2.333078000	1.383099000	0.066186000
C	-0.649463000	-0.938847000	-0.326198000
C	-0.760578000	-1.748623000	0.798196000
C	-0.346540000	-1.624777000	-1.492270000
C	-0.548350000	-3.115683000	0.791766000
C	-0.126441000	-2.992698000	-1.549022000
C	-0.223239000	-3.745375000	-0.395093000

C	-2.403109000	1.078488000	-0.021439000
C	-2.897945000	1.858182000	1.014035000
C	-3.354199000	0.679223000	-0.949966000
C	-4.233328000	2.209887000	1.138909000
C	-4.698112000	1.003600000	-0.866347000
C	-5.141941000	1.776709000	0.191251000
F	-1.068654000	-1.207519000	1.991139000
F	-0.622612000	-3.828541000	1.920983000
F	0.012170000	-5.059333000	-0.420617000
F	0.190848000	-3.588294000	-2.704589000
F	-0.226431000	-0.971824000	-2.666572000
F	-2.076614000	2.325414000	1.973356000
F	-4.653154000	2.962349000	2.161880000
F	-6.431198000	2.105753000	0.292552000
F	-5.567137000	0.586128000	-1.792826000
F	-2.987837000	-0.064264000	-2.011132000
C	-0.109447000	1.609034000	-1.298966000
H	-0.568341000	1.487891000	-2.285303000
H	-0.265611000	2.659441000	-1.019981000
C	1.400031000	1.396706000	-1.489451000
H	1.619955000	0.439448000	-1.969663000
H	1.857739000	2.172807000	-2.109752000
H	-0.290771000	0.947393000	0.912196000
H	1.718254000	2.241149000	0.984334000
C	2.409016000	-0.235613000	0.838807000
C	2.128340000	-0.364716000	2.197520000
C	2.760337000	-1.351261000	0.076366000
C	2.199068000	-1.616759000	2.793851000
C	2.832516000	-2.596962000	0.682080000
C	2.550873000	-2.729003000	2.038453000
H	1.965701000	-1.724739000	3.846199000
H	3.095293000	-3.467675000	0.092674000
C	4.008131000	2.009621000	-0.202364000
C	4.160321000	3.265028000	-0.798483000
C	5.128215000	1.283055000	0.200278000
C	5.431246000	3.785172000	-0.993385000
C	6.398031000	1.810695000	-0.003196000
C	6.549618000	3.056929000	-0.598019000
H	5.549081000	4.758677000	-1.454530000
H	7.268870000	1.244976000	0.306802000
H	3.291111000	3.837838000	-1.106415000
H	7.541670000	3.465881000	-0.752476000
H	5.016805000	0.313181000	0.670745000
H	1.836610000	0.500382000	2.782090000
H	2.970485000	-1.251451000	-0.983087000
H	2.594335000	-3.706266000	2.505096000

G8

	Coordinates (Angstroms)		
	X	Y	Z
B	-0.716865000	-0.078549000	0.079502000
P	2.937906000	-0.372359000	0.544521000
C	-1.611833000	-1.441586000	0.093141000
C	-2.677916000	-1.558540000	0.977541000
C	-1.460578000	-2.514782000	-0.773160000
C	-3.516991000	-2.656475000	1.038889000
C	-2.280416000	-3.632148000	-0.751767000

C	-3.313963000	-3.706719000	0.162644000
C	-1.695057000	1.223932000	-0.153784000
C	-1.697864000	2.399494000	0.577326000
C	-2.547091000	1.235600000	-1.252082000
C	-2.485596000	3.499420000	0.286866000
C	-3.354747000	2.309213000	-1.586631000
C	-3.327428000	3.452140000	-0.807919000
F	-2.934359000	-0.565542000	1.851628000
F	-4.512019000	-2.709707000	1.925357000
F	-4.106473000	-4.774589000	0.197262000
F	-2.083609000	-4.634678000	-1.609594000
F	-0.504232000	-2.519531000	-1.713040000
F	-0.882412000	2.539017000	1.658452000
F	-2.434538000	4.594601000	1.044042000
F	-4.095629000	4.494567000	-1.112217000
F	-4.153395000	2.253305000	-2.652029000
F	-2.608773000	0.170389000	-2.068146000
C	0.518042000	0.070911000	-0.948873000
H	0.100535000	-0.082462000	-1.950949000
H	0.823175000	1.124686000	-0.939164000
C	1.784402000	-0.797025000	-0.852238000
H	1.532130000	-1.852800000	-0.733124000
H	2.349746000	-0.703178000	-1.785099000
C	4.551260000	-1.008552000	-0.063731000
C	5.733197000	-0.471842000	0.459459000
C	4.639288000	-2.077695000	-0.957766000
C	6.969548000	-0.976909000	0.080706000
C	5.879808000	-2.586604000	-1.332063000
C	7.047199000	-2.037366000	-0.817807000
H	7.875108000	-0.542411000	0.489992000
H	5.929124000	-3.413719000	-2.032023000
C	3.119169000	1.441086000	0.324862000
C	2.607001000	2.289150000	1.305892000
C	3.669007000	1.997916000	-0.833505000
C	2.627629000	3.670190000	1.131153000
C	3.697811000	3.374679000	-1.005660000
C	3.172246000	4.213320000	-0.025075000
H	2.218924000	4.318371000	1.898412000
H	4.126565000	3.797119000	-1.907799000
O	-0.120367000	0.046792000	1.591800000
H	-0.146800000	0.935535000	1.971799000
H	1.642708000	-1.733826000	1.961893000
C	0.037912000	-0.988345000	2.631331000
H	0.539870000	-0.459814000	3.446013000
H	-0.950331000	-1.323817000	2.935790000
O	0.734845000	-2.041206000	2.135114000
H	3.742427000	-2.523267000	-1.373083000
H	5.687608000	0.354259000	1.162701000
H	8.012638000	-2.433447000	-1.112665000
H	4.072471000	1.349605000	-1.604783000
H	2.187677000	1.868776000	2.214250000
H	3.191069000	5.288576000	-0.164733000

TSG2-3

	Coordinates (Angstroms)		
	X	Y	Z
B	1.096394000	0.529632000	0.005083000

P	-1.985630000	1.246731000	-0.002841000
C	0.500313000	-1.017777000	0.014681000
C	0.033189000	-1.683230000	-1.107908000
C	0.327461000	-1.724694000	1.198666000
C	-0.621842000	-2.903842000	-1.074839000
C	-0.326350000	-2.939464000	1.287333000
C	-0.818528000	-3.530987000	0.137835000
C	2.736092000	0.607177000	-0.049468000
C	3.366915000	1.839809000	-0.198876000
C	3.605480000	-0.470847000	0.056720000
C	4.740840000	2.002779000	-0.245778000
C	4.987175000	-0.351039000	0.018441000
C	5.561312000	0.894959000	-0.135873000
F	0.173022000	-1.143521000	-2.341594000
F	-1.080359000	-3.462847000	-2.196279000
F	-1.479195000	-4.685204000	0.202870000
F	-0.504927000	-3.537862000	2.467562000
F	0.794997000	-1.225070000	2.357392000
F	2.646974000	2.968968000	-0.308808000
F	5.281183000	3.215327000	-0.392730000
F	6.885597000	1.029528000	-0.176472000
F	5.766175000	-1.430527000	0.127974000
F	3.147011000	-1.725227000	0.205784000
C	0.496250000	1.408672000	1.252785000
H	1.047028000	1.205636000	2.174978000
H	0.679154000	2.462281000	1.017766000
C	-1.007160000	1.232944000	1.559522000
H	-1.192899000	0.287046000	2.070281000
H	-1.387904000	2.024118000	2.209883000
C	-2.918312000	-0.299431000	-0.178817000
C	-3.228783000	-0.716873000	-1.475750000
C	-3.294392000	-1.087545000	0.908756000
C	-3.900669000	-1.914006000	-1.681262000
C	-3.959025000	-2.290025000	0.697351000
C	-4.259022000	-2.705082000	-0.594868000
H	-4.125554000	-2.237946000	-2.690630000
H	-4.233321000	-2.907233000	1.545189000
C	-3.173048000	2.617730000	0.074446000
C	-2.668968000	3.917761000	-0.025351000
C	-4.542357000	2.414064000	0.240316000
C	-3.530304000	5.003645000	0.052344000
C	-5.400042000	3.506282000	0.312860000
C	-4.896180000	4.798448000	0.221056000
H	-3.134767000	6.009904000	-0.026249000
H	-6.464017000	3.344506000	0.443320000
O	0.553469000	1.256052000	-1.249277000
H	0.751391000	0.793856000	-2.069792000
H	-0.719253000	1.333941000	-1.022640000
H	-1.605367000	4.083189000	-0.167007000
H	-5.569049000	5.646894000	0.276658000
H	-4.942561000	1.408849000	0.313023000
H	-2.929053000	-0.114350000	-2.326974000
H	-3.069770000	-0.776192000	1.922340000
H	-4.763899000	-3.650676000	-0.755974000

TSG3-4

Coordinates (Angstroms)

	X	Y	Z
B	0.849755000	-0.139550000	0.013846000
P	-2.714280000	-0.212696000	0.098056000
C	1.168778000	1.482203000	0.077262000
C	1.751048000	2.042139000	1.208474000
C	0.947054000	2.390327000	-0.948806000
C	2.036313000	3.388583000	1.354919000
C	1.211226000	3.747140000	-0.849772000
C	1.753814000	4.254623000	0.315306000
C	2.233220000	-1.019883000	-0.203871000
C	2.278740000	-2.374463000	0.114265000
C	3.397356000	-0.532286000	-0.785546000
C	3.384513000	-3.181600000	-0.095232000
C	4.526062000	-1.304689000	-1.014121000
C	4.522748000	-2.640573000	-0.662502000
F	2.113831000	1.256938000	2.244889000
F	2.590262000	3.853443000	2.476651000
F	2.016869000	5.555048000	0.427956000
F	0.959746000	4.565778000	-1.874130000
F	0.487519000	1.983076000	-2.145959000
F	1.208400000	-2.988575000	0.651860000
F	3.360158000	-4.473778000	0.239983000
F	5.597526000	-3.397905000	-0.873468000
F	5.613650000	-0.770421000	-1.574516000
F	3.490181000	0.746900000	-1.186956000
C	-0.196285000	-0.629665000	-1.135435000
H	0.311380000	-0.524315000	-2.100346000
H	-0.334174000	-1.709974000	-1.005530000
C	-1.580609000	0.020952000	-1.315524000
H	-1.494225000	1.102116000	-1.446806000
H	-2.068807000	-0.376389000	-2.211364000
C	-4.278220000	0.618534000	-0.314684000
C	-5.266221000	0.679007000	0.673713000
C	-4.513942000	1.210791000	-1.555841000
C	-6.476086000	1.308701000	0.417861000
C	-5.724882000	1.848708000	-1.804956000
C	-6.705931000	1.896639000	-0.822615000
H	-7.237720000	1.345802000	1.188434000
H	-5.899070000	2.307856000	-2.771554000
C	-3.086231000	-1.995577000	0.159825000
C	-2.176885000	-2.826147000	0.821838000
C	-4.218767000	-2.551633000	-0.437774000
C	-2.394560000	-4.197530000	0.874051000
C	-4.436187000	-3.922726000	-0.373793000
C	-3.525787000	-4.746369000	0.280456000
H	-1.680204000	-4.835404000	1.382412000
H	-5.318887000	-4.348754000	-0.837498000
O	0.303534000	-0.535671000	1.417657000
H	1.026889000	-0.721599000	2.025794000
H	-1.838248000	0.325057000	1.539910000
C	-0.905902000	0.435547000	2.464113000
O	-0.536862000	0.570868000	3.535454000
H	-4.933468000	-1.917276000	-0.950857000
H	-1.289100000	-2.401670000	1.278504000
H	-3.698960000	-5.815771000	0.328189000
H	-5.091764000	0.226147000	1.644929000
H	-3.761831000	1.178571000	-2.335050000

H	-7.649315000	2.393157000	-1.020593000

TSG1-7

	Coordinates (Angstroms)		
	X	Y	Z
C	0.299545000	1.598257000	1.185245000
C	0.502288000	1.080827000	-0.087058000
C	0.041408000	1.878109000	-1.124472000
C	-0.643513000	3.064405000	-0.924688000
C	-0.859577000	3.514408000	0.364709000
C	-0.368938000	2.783659000	1.430403000
B	1.103200000	-0.410451000	-0.298183000
C	2.698005000	-0.577268000	-0.099449000
C	3.263226000	-1.669772000	0.541979000
C	4.628629000	-1.847068000	0.681884000
C	5.490740000	-0.902092000	0.154007000
C	4.972897000	0.198418000	-0.504926000
C	3.599744000	0.339371000	-0.620360000
F	2.470132000	-2.618987000	1.069381000
F	5.119035000	-2.913471000	1.315020000
F	6.807283000	-1.054268000	0.274358000
F	5.797834000	1.107181000	-1.026796000
F	3.159382000	1.415606000	-1.289741000
F	0.225507000	1.505543000	-2.400687000
F	-1.110352000	3.765650000	-1.958509000
F	-1.547516000	4.632177000	0.579468000
F	-0.596261000	3.196750000	2.678092000
F	0.703727000	0.903348000	2.262957000
C	0.511894000	-1.287350000	-1.518204000
C	-1.022225000	-1.231131000	-1.690208000
P	-1.832110000	-1.285344000	-0.028944000
C	-2.881424000	0.200136000	0.108910000
C	-3.004798000	0.802512000	1.361934000
C	-3.747324000	1.968605000	1.512226000
C	-4.362662000	2.546101000	0.408462000
C	-4.251313000	1.946596000	-0.842596000
C	-3.521432000	0.774640000	-0.992029000
C	-2.955873000	-2.720835000	-0.060478000
C	-2.397220000	-3.981635000	-0.293491000
C	-3.202063000	-5.112562000	-0.302184000
C	-4.569150000	-4.997858000	-0.066866000
C	-5.126633000	-3.748410000	0.174909000
C	-4.325176000	-2.611029000	0.179776000
H	-1.392287000	-2.058164000	-2.299890000
H	-1.316078000	-0.306976000	-2.188737000
H	0.819674000	-2.328063000	-1.356001000
H	0.983699000	-0.977289000	-2.455096000
H	-2.759890000	-6.085255000	-0.486478000
H	-6.191046000	-3.652744000	0.358385000
H	-3.824570000	2.436358000	2.486948000
H	-4.730092000	2.395332000	-1.705533000
H	0.765568000	-1.011280000	0.884613000
H	-0.096601000	-1.139321000	0.695381000
H	-1.330209000	-4.081304000	-0.469670000
H	-4.772572000	-1.640799000	0.364797000
H	-5.196814000	-5.882077000	-0.070118000

H	-2.500178000	0.371386000	2.220782000
H	-3.444647000	0.314397000	-1.970918000
H	-4.921596000	3.468283000	0.520136000

TSG7

	Coordinates (Angstroms)		
	X	Y	Z
B	-1.096957000	-0.488232000	0.216387000
P	2.175395000	-1.134400000	-0.091818000
C	-0.550714000	1.041698000	0.292594000
C	-0.075123000	1.773298000	-0.785120000
C	-0.480921000	1.691279000	1.516265000
C	0.444924000	3.051377000	-0.667935000
C	0.053900000	2.956706000	1.684870000
C	0.525330000	3.640986000	0.579039000
C	-2.714417000	-0.604607000	0.142328000
C	-3.482839000	-1.516673000	0.857634000
C	-3.449313000	0.213900000	-0.711378000
C	-4.863428000	-1.599573000	0.760976000
C	-4.826059000	0.162175000	-0.841053000
C	-5.542833000	-0.753893000	-0.093990000
F	-0.075489000	1.247747000	-2.019970000
F	0.885282000	3.710443000	-1.741215000
F	1.062049000	4.851960000	0.715642000
F	0.125884000	3.517106000	2.894510000
F	-0.926482000	1.072553000	2.625326000
F	-2.918832000	-2.393502000	1.703276000
F	-5.540910000	-2.495052000	1.483156000
F	-6.867225000	-0.824417000	-0.202773000
F	-5.464229000	0.978226000	-1.682422000
F	-2.823929000	1.107606000	-1.494202000
C	-0.319228000	-1.518768000	1.190684000
H	-0.801030000	-1.527788000	2.171578000
H	-0.435529000	-2.539427000	0.806339000
C	1.168652000	-1.241599000	1.443611000
H	1.296339000	-0.304631000	1.989123000
H	1.614181000	-2.025368000	2.060824000
H	-0.749120000	-0.869403000	-1.011501000
H	1.297037000	-1.536592000	-1.398517000
C	2.910061000	0.516133000	-0.237759000
C	3.129611000	1.023314000	-1.520122000
C	3.258168000	1.277383000	0.878953000
C	3.686073000	2.285236000	-1.681977000
C	3.803554000	2.543653000	0.710594000
C	4.015320000	3.048584000	-0.567721000
H	3.842111000	2.680555000	-2.678726000
H	4.058252000	3.138523000	1.580331000
C	3.549536000	-2.312390000	0.097409000
C	3.227894000	-3.662033000	0.268150000
C	4.886293000	-1.921319000	0.042610000
C	4.235494000	-4.607134000	0.398273000
C	5.892165000	-2.873639000	0.170364000
C	5.569782000	-4.213075000	0.349815000
H	3.980925000	-5.651955000	0.535029000
H	6.930460000	-2.564410000	0.129395000
C	-0.711935000	-1.719879000	-2.107115000
O	0.534098000	-1.884207000	-2.361026000

O	-1.442221000	-2.795614000	-1.734532000
H	-1.312185000	-1.051350000	-2.729839000
H	-0.828054000	-3.460150000	-1.395588000
H	5.146689000	-0.878242000	-0.097070000
H	2.188840000	-3.975806000	0.302488000
H	6.356487000	-4.952460000	0.449538000
H	2.849596000	0.440849000	-2.391119000
H	3.107608000	0.891166000	1.880804000
H	4.431495000	4.041353000	-0.695240000

TSG8

	Coordinates (Angstroms)		
	X	Y	Z
B	-0.715286000	0.134702000	-0.105466000
P	2.776776000	0.057966000	0.056717000
C	-1.213328000	-1.415951000	0.139232000
C	-2.032552000	-1.698598000	1.226618000
C	-0.943776000	-2.510194000	-0.667443000
C	-2.501554000	-2.962347000	1.537947000
C	-1.393696000	-3.794235000	-0.399135000
C	-2.173619000	-4.025694000	0.716905000
C	-2.071565000	1.053745000	-0.359811000
C	-2.418981000	2.208974000	0.321607000
C	-2.950713000	0.709051000	-1.379666000
C	-3.548913000	2.962832000	0.046840000
C	-4.092108000	1.427132000	-1.692403000
C	-4.396789000	2.565987000	-0.969095000
F	-2.422758000	-0.709604000	2.054423000
F	-3.269143000	-3.163588000	2.612090000
F	-2.613645000	-5.252513000	0.992395000
F	-1.086889000	-4.806500000	-1.214910000
F	-0.227553000	-2.381376000	-1.798643000
F	-1.636678000	2.685639000	1.320290000
F	-3.821744000	4.064963000	0.748740000
F	-5.488419000	3.274773000	-1.253211000
F	-4.896676000	1.036413000	-2.683136000
F	-2.712339000	-0.376985000	-2.137621000
C	0.292400000	0.454979000	-1.346926000
H	-0.193448000	0.194365000	-2.293473000
H	0.397447000	1.548133000	-1.371058000
C	1.703199000	-0.152931000	-1.406472000
H	1.663007000	-1.236238000	-1.542309000
H	2.261183000	0.244337000	-2.261310000
C	4.406763000	-0.551053000	-0.475084000
C	5.440465000	0.302134000	-0.861573000
C	4.602431000	-1.934479000	-0.507924000
C	6.654827000	-0.225614000	-1.285121000
C	5.815608000	-2.456688000	-0.937292000
C	6.842360000	-1.602496000	-1.326348000
H	7.454998000	0.442410000	-1.583334000
H	5.961215000	-3.530656000	-0.962648000
C	2.954545000	1.823722000	0.417487000
C	3.222802000	2.207297000	1.732831000
C	2.835233000	2.796074000	-0.576574000
C	3.372494000	3.551486000	2.050164000
C	2.981760000	4.139840000	-0.254399000
C	3.249325000	4.517480000	1.057266000

H	3.580057000	3.843529000	3.073242000
H	2.885896000	4.892458000	-1.028681000
O	-0.016088000	0.550407000	1.197011000
H	-0.099484000	1.483354000	1.408425000
H	2.184013000	-0.748557000	1.316629000
C	0.637482000	-0.394941000	2.631915000
H	1.098964000	0.512992000	3.039322000
H	-0.314393000	-0.668992000	3.088960000
O	1.362947000	-1.316045000	2.165381000
H	3.314348000	1.454193000	2.509228000
H	3.362232000	5.566996000	1.305037000
H	2.630225000	2.509748000	-1.602232000
H	5.302803000	1.377286000	-0.829484000
H	3.808702000	-2.606035000	-0.195489000
H	7.790147000	-2.011293000	-1.658415000

TSG9

	Coordinates (Angstroms)		
	X	Y	Z
B	0.909732000	-0.114496000	-0.128855000
P	-2.606581000	-0.500821000	0.151449000
C	1.066476000	1.497604000	-0.196241000
C	0.397494000	2.388359000	0.626834000
C	1.866677000	2.076627000	-1.170247000
C	0.516565000	3.763973000	0.514481000
C	2.017469000	3.444952000	-1.317201000
C	1.337246000	4.295541000	-0.463684000
C	2.326345000	-0.881784000	0.014628000
C	2.742724000	-1.952838000	-0.765310000
C	3.239753000	-0.478233000	0.984435000
C	3.969395000	-2.578864000	-0.605746000
C	4.472003000	-1.076338000	1.178419000
C	4.840424000	-2.138526000	0.372440000
F	-0.422918000	1.931053000	1.587013000
F	-0.151099000	4.577544000	1.334757000
F	1.465195000	5.615260000	-0.588720000
F	2.803114000	3.950210000	-2.270140000
F	2.535098000	1.296636000	-2.037822000
F	1.966754000	-2.436905000	-1.747224000
F	4.317940000	-3.600285000	-1.390607000
F	6.020211000	-2.730928000	0.538097000
F	5.301719000	-0.645097000	2.129664000
F	2.939503000	0.542864000	1.804388000
C	-0.165624000	-0.681311000	-1.188934000
H	0.272697000	-0.561014000	-2.186455000
H	-0.319168000	-1.758938000	-1.071360000
C	-1.523250000	0.036025000	-1.209914000
H	-1.405052000	1.120317000	-1.128006000
H	-2.068027000	-0.163177000	-2.138152000
H	0.381853000	-0.308489000	1.104110000
H	-1.897117000	-0.570452000	1.511386000
C	-4.122159000	0.489397000	0.142187000
C	-5.088144000	0.222947000	1.116658000
C	-4.338291000	1.499420000	-0.794830000
C	-6.261930000	0.961901000	1.147463000
C	-5.517204000	2.235808000	-0.757685000
C	-6.476959000	1.968514000	0.210325000

H	-7.009079000	0.753069000	1.904334000
H	-5.682918000	3.018603000	-1.488927000
C	-3.057540000	-2.222069000	-0.183625000
C	-2.654067000	-3.203980000	0.721864000
C	-3.764771000	-2.576116000	-1.334403000
C	-2.951977000	-4.538962000	0.470041000
C	-4.059458000	-3.909569000	-1.578785000
C	-3.651141000	-4.890175000	-0.677901000
H	-2.637162000	-5.301619000	1.172846000
H	-4.607299000	-4.185854000	-2.472454000
C	0.106247000	-1.113884000	2.136167000
O	-1.034101000	-0.799896000	2.630239000
H	1.002883000	-0.856976000	2.717514000
H	0.212644000	-2.064243000	1.583681000
H	-2.113964000	-2.924954000	1.620995000
H	-4.085703000	-1.813462000	-2.036506000
H	-3.880398000	-5.931804000	-0.873096000
H	-4.924252000	-0.562677000	1.847385000
H	-3.599203000	1.717762000	-1.556406000
H	-7.395347000	2.544186000	0.236475000

H1

	Coordinates (Angstroms)		
	X	Y	Z
C	-4.531158000	4.295343000	-0.629831000
C	-3.649627000	5.203731000	-0.156795000
C	-2.328996000	4.822040000	0.235707000
C	-1.892524000	3.485705000	0.164623000
C	-2.877525000	2.498734000	-0.256512000
C	-4.160288000	2.922979000	-0.684046000
H	-1.852612000	6.866529000	0.756715000
H	-5.525216000	4.585210000	-0.952892000
H	-3.919156000	6.251613000	-0.081948000
C	-1.457070000	5.858732000	0.689439000
C	-0.494613000	3.201351000	0.483668000
C	0.344683000	4.276254000	0.892215000
C	-0.173645000	5.598062000	1.017300000
H	0.489452000	6.387111000	1.355061000
C	-5.065285000	1.945711000	-1.142028000
C	-3.446121000	0.268959000	-0.616384000
H	-6.043479000	2.257879000	-1.493410000
C	1.717837000	4.060003000	1.150964000
C	0.117979000	1.927360000	0.369137000
C	2.281265000	2.830542000	0.971010000
H	3.343616000	2.691654000	1.142059000
C	1.477877000	1.730748000	0.577754000
H	2.320397000	4.904911000	1.467515000
H	-0.500343000	1.089637000	0.084095000
N	-2.574482000	1.180115000	-0.222881000
B	2.123118000	0.357376000	0.318943000
C	-4.715841000	0.624617000	-1.120486000
H	-5.410238000	-0.132696000	-1.454549000
C	3.660204000	0.263938000	-0.044234000
C	4.528754000	-0.541861000	0.679461000
C	4.211774000	0.965932000	-1.107974000
C	5.875777000	-0.644513000	0.381407000
C	5.550318000	0.873765000	-1.446339000

C	6.384856000	0.065604000	-0.692647000
C	1.354492000	-1.014902000	0.425809000
C	1.412705000	-1.954531000	-0.594935000
C	0.721324000	-1.413206000	1.594691000
C	0.884950000	-3.225224000	-0.471484000
C	0.207243000	-2.686964000	1.762851000
C	0.279188000	-3.591091000	0.718196000
F	3.437119000	1.747614000	-1.867209000
F	6.042068000	1.548863000	-2.483184000
F	7.673014000	-0.027539000	-0.999351000
F	6.681784000	-1.413759000	1.110032000
F	4.075046000	-1.236554000	1.730155000
F	0.636723000	-0.574929000	2.632554000
F	-0.368583000	-3.046277000	2.909468000
F	-0.241718000	-4.805457000	0.851467000
F	0.946190000	-4.093489000	-1.480183000
F	1.996939000	-1.636635000	-1.756532000
P	-2.837541000	-1.462476000	-0.335556000
C	-3.406395000	-2.468024000	-1.843603000
C	-3.046449000	-1.630166000	-3.081380000
C	-2.515100000	-3.723857000	-1.851100000
C	-4.867853000	-2.916578000	-1.937620000
H	-3.731274000	-0.790415000	-3.222782000
H	-2.028062000	-1.231492000	-3.019420000
H	-3.102439000	-2.261683000	-3.975143000
H	-2.646013000	-4.333979000	-0.954196000
H	-2.770712000	-4.345442000	-2.716958000
H	-1.459275000	-3.458377000	-1.931918000
H	-5.011128000	-3.450673000	-2.884652000
H	-5.136251000	-3.604688000	-1.133273000
H	-5.574832000	-2.085541000	-1.927102000
C	-3.851286000	-1.922195000	1.205568000
C	-3.637537000	-3.414307000	1.490723000
C	-3.231109000	-1.102121000	2.349597000
C	-5.352928000	-1.619916000	1.166334000
H	-4.138502000	-4.046900000	0.754207000
H	-2.578320000	-3.680363000	1.505343000
H	-4.053911000	-3.657343000	2.474737000
H	-3.363606000	-0.027383000	2.196288000
H	-3.722854000	-1.367559000	3.292417000
H	-2.162802000	-1.295045000	2.462546000
H	-5.804797000	-1.950116000	2.109359000
H	-5.547115000	-0.549381000	1.070959000
H	-5.868136000	-2.136513000	0.356447000

H1_CO

	Coordinates (Angstroms)		
	X	Y	Z
C	4.625803000	4.322993000	-0.088995000
C	3.662752000	5.203099000	-0.444451000
C	2.295824000	4.801180000	-0.575102000
C	1.898313000	3.471358000	-0.347613000
C	2.952970000	2.510575000	-0.056738000
C	4.288060000	2.954294000	0.108476000
H	1.700080000	6.807911000	-1.124625000
H	5.657524000	4.634073000	0.035145000
H	3.904893000	6.245454000	-0.622405000

C	1.340832000	5.801836000	-0.936484000
C	0.473223000	3.159409000	-0.401502000
C	-0.441122000	4.188877000	-0.756212000
C	0.026724000	5.506173000	-1.042325000
H	-0.695225000	6.265754000	-1.322917000
C	5.262781000	2.000975000	0.461760000
C	3.585859000	0.302187000	0.339975000
H	6.284840000	2.328804000	0.622641000
C	-1.826212000	3.920060000	-0.796776000
C	-0.084946000	1.899801000	-0.073858000
C	-2.323406000	2.686056000	-0.466817000
H	-3.393965000	2.513271000	-0.497435000
C	-1.444165000	1.648935000	-0.098182000
H	-2.498583000	4.722628000	-1.082774000
H	0.597477000	1.105155000	0.189817000
N	2.659816000	1.194144000	0.036848000
B	-1.992849000	0.171373000	0.321016000
C	4.920555000	0.682667000	0.598600000
H	5.659736000	-0.048992000	0.889579000
C	-3.612485000	0.031122000	0.342431000
C	-4.327009000	-0.499730000	-0.723483000
C	-4.385758000	0.505496000	1.392232000
C	-5.711054000	-0.570376000	-0.741136000
C	-5.767217000	0.454303000	1.415950000
C	-6.435634000	-0.094226000	0.336136000
C	-1.226338000	-1.079802000	-0.385689000
C	-1.028704000	-2.287425000	0.265074000
C	-0.776279000	-1.029080000	-1.698709000
C	-0.413763000	-3.381703000	-0.315563000
C	-0.170732000	-2.106308000	-2.320742000
C	0.015713000	-3.287372000	-1.625504000
F	-3.780547000	1.063424000	2.457993000
F	-6.451973000	0.924935000	2.456233000
F	-7.762834000	-0.154366000	0.330588000
F	-6.348524000	-1.086749000	-1.790378000
F	-3.697674000	-0.954259000	-1.813345000
F	-0.956335000	0.064698000	-2.443007000
F	0.240314000	-2.015837000	-3.585482000
F	0.616053000	-4.319203000	-2.211144000
F	-0.218883000	-4.506224000	0.372217000
F	-1.437185000	-2.424302000	1.542794000
P	2.891036000	-1.420416000	0.418676000
C	3.145613000	-1.896239000	2.240066000
C	2.681210000	-0.685359000	3.065169000
C	2.180984000	-3.060908000	2.521330000
C	4.554649000	-2.293682000	2.683966000
H	3.399944000	0.137098000	3.023684000
H	1.714308000	-0.304827000	2.721212000
H	2.568895000	-0.981247000	4.114013000
H	2.437894000	-3.958645000	1.955096000
H	2.222393000	-3.316495000	3.586580000
H	1.148971000	-2.792294000	2.280375000
H	4.548394000	-2.486201000	3.763755000
H	4.895320000	-3.206954000	2.191627000
H	5.287065000	-1.505036000	2.498271000
C	4.017126000	-2.439165000	-0.715237000
C	3.648601000	-3.916243000	-0.514257000

C	3.612609000	-2.013206000	-2.136966000
C	5.536862000	-2.276762000	-0.594200000
H	4.051347000	-4.310486000	0.421904000
H	2.566756000	-4.073958000	-0.512700000
H	4.069791000	-4.509767000	-1.333388000
H	3.795720000	-0.947494000	-2.307639000
H	4.203722000	-2.575653000	-2.868532000
H	2.557909000	-2.208885000	-2.336663000
H	6.020043000	-3.009976000	-1.251067000
H	5.862808000	-1.288640000	-0.925006000
H	5.909200000	-2.445900000	0.416532000
C	-1.469760000	0.166312000	1.850130000
O	-1.001731000	0.271484000	2.861875000

H2

	Coordinates (Angstroms)		
	X	Y	Z
C	5.719405000	2.994212000	0.027866000
C	5.023130000	4.028443000	0.561408000
C	3.615157000	3.940495000	0.812163000
C	2.897829000	2.794972000	0.437896000
C	3.659325000	1.664702000	-0.054099000
C	5.053390000	1.766020000	-0.259400000
H	3.528582000	5.883563000	1.770036000
H	6.786010000	3.072892000	-0.153340000
H	5.530131000	4.951178000	0.823899000
C	2.950931000	5.007634000	1.494150000
C	1.465809000	2.754952000	0.662666000
C	0.870760000	3.768959000	1.449727000
C	1.644628000	4.902945000	1.844079000
H	1.161610000	5.688960000	2.415770000
C	5.730772000	0.609243000	-0.700609000
C	3.661393000	-0.587746000	-0.639073000
H	6.801938000	0.659388000	-0.868477000
C	-0.488773000	3.637998000	1.825407000
C	0.614117000	1.747445000	0.145398000
C	-1.232246000	2.557771000	1.426813000
H	-2.259012000	2.461412000	1.767992000
C	-0.696426000	1.599148000	0.530965000
H	-0.927867000	4.405907000	2.454832000
H	1.040143000	1.058282000	-0.558938000
N	3.024933000	0.491879000	-0.239249000
B	-1.556986000	0.332573000	0.050617000
C	5.050423000	-0.567906000	-0.894610000
H	5.570841000	-1.459254000	-1.217509000
P	2.467778000	-2.004465000	-0.726719000
C	3.136762000	-3.264573000	0.511233000
C	3.490086000	-2.467294000	1.778092000
C	1.978227000	-4.215428000	0.856783000
C	4.352647000	-4.079754000	0.066835000
H	4.381260000	-1.850293000	1.637728000
H	2.669410000	-1.815487000	2.092765000
H	3.692663000	-3.168823000	2.594632000
H	1.646682000	-4.800700000	-0.003585000
H	2.310018000	-4.920424000	1.627203000
H	1.119405000	-3.667330000	1.253608000
H	4.674312000	-4.722002000	0.895119000

H	4.119827000	-4.729297000	-0.780078000
H	5.199487000	-3.444473000	-0.203472000
C	2.572555000	-2.610740000	-2.510552000
C	1.774087000	-3.916250000	-2.619016000
C	1.854159000	-1.518502000	-3.323402000
C	3.971019000	-2.809545000	-3.105007000
H	2.298598000	-4.754684000	-2.154758000
H	0.785670000	-3.830587000	-2.155709000
H	1.625070000	-4.160468000	-3.676543000
H	2.344036000	-0.545357000	-3.216922000
H	1.871656000	-1.785068000	-4.385966000
H	0.809243000	-1.404887000	-3.022285000
H	3.868688000	-3.231014000	-4.111801000
H	4.501600000	-1.860390000	-3.205114000
H	4.588590000	-3.493459000	-2.521958000
C	-2.181338000	-0.438943000	1.349685000
C	-1.386308000	-1.220760000	2.177424000
C	-3.499124000	-0.307984000	1.764790000
C	-1.853788000	-1.870177000	3.305694000
C	-4.007855000	-0.930534000	2.893677000
C	-3.181395000	-1.725349000	3.666092000
C	-2.647904000	0.560472000	-1.145023000
C	-3.277456000	-0.535362000	-1.719735000
C	-3.031446000	1.786762000	-1.668976000
C	-4.221953000	-0.458208000	-2.723780000
C	-3.980336000	1.914877000	-2.673929000
C	-4.579600000	0.789047000	-3.204768000
F	-4.358731000	0.466471000	1.082891000
F	-5.284866000	-0.768094000	3.242193000
F	-3.655084000	-2.335402000	4.749316000
F	-1.039456000	-2.624874000	4.044354000
F	-0.078053000	-1.380293000	1.902171000
F	-2.960904000	-1.779512000	-1.286510000
F	-4.784360000	-1.556393000	-3.227719000
F	-2.509221000	2.932702000	-1.217581000
F	-5.486227000	0.900664000	-4.171193000
F	-4.316628000	3.119844000	-3.135611000
O	-0.530022000	-0.709319000	-0.675846000
H	-0.952130000	-1.542250000	-0.929890000
H	0.385240000	-0.942080000	-0.360033000

H3

	Coordinates (Angstroms)		
	X	Y	Z
C	5.656203000	3.102797000	0.002206000
C	4.890361000	4.148338000	0.394773000
C	3.470081000	4.035446000	0.546626000
C	2.806125000	2.832921000	0.249553000
C	3.639748000	1.699530000	-0.105869000
C	5.045790000	1.837716000	-0.238161000
H	3.284187000	6.075667000	1.249955000
H	6.729487000	3.203511000	-0.114780000
H	5.346881000	5.109392000	0.607542000
C	2.739936000	5.163880000	1.028095000
C	1.356810000	2.778531000	0.364513000
C	0.680946000	3.897685000	0.916845000
C	1.402847000	5.084688000	1.237242000

H	0.857279000	5.932130000	1.640395000
C	5.798367000	0.697416000	-0.583995000
C	3.791463000	-0.554086000	-0.596557000
H	6.873267000	0.792042000	-0.694588000
C	-0.716249000	3.823616000	1.135805000
C	0.558994000	1.678167000	-0.040409000
C	-1.426240000	2.697803000	0.809995000
H	-2.492053000	2.661883000	1.018398000
C	-0.797943000	1.597110000	0.178197000
H	-1.213255000	4.682383000	1.577510000
H	1.031206000	0.842342000	-0.518310000
N	3.070716000	0.492281000	-0.273896000
B	-1.548839000	0.191414000	-0.148416000
C	5.183261000	-0.516387000	-0.775314000
H	5.744138000	-1.402326000	-1.044228000
P	2.729551000	-2.023032000	-0.798605000
C	3.162959000	-3.346143000	0.421479000
C	3.494855000	-2.626878000	1.739400000
C	1.927731000	-4.234808000	0.633191000
C	4.357048000	-4.177138000	-0.054140000
H	4.412246000	-2.038360000	1.666619000
H	2.681311000	-1.973242000	2.064645000
H	3.641610000	-3.387966000	2.511322000
H	1.642708000	-4.781049000	-0.266656000
H	2.171763000	-4.970131000	1.405541000
H	1.072141000	-3.652978000	0.982829000
H	4.614096000	-4.893205000	0.731849000
H	4.120219000	-4.740682000	-0.959034000
H	5.238572000	-3.558446000	-0.243103000
C	2.611997000	-2.447282000	-2.596760000
C	1.749036000	-3.703305000	-2.765244000
C	1.911540000	-1.236720000	-3.240645000
C	3.991524000	-2.652601000	-3.232099000
H	2.251419000	-4.595292000	-2.385306000
H	0.778039000	-3.605383000	-2.272132000
H	1.566691000	-3.850421000	-3.833747000
H	2.516200000	-0.328822000	-3.164983000
H	1.764752000	-1.453040000	-4.302870000
H	0.936734000	-1.039166000	-2.786205000
H	3.841557000	-2.890473000	-4.289452000
H	4.600327000	-1.747351000	-3.182917000
H	4.544057000	-3.478032000	-2.779439000
C	-1.941472000	-0.398816000	1.368665000
C	-1.029873000	-1.126563000	2.122005000
C	-3.131613000	-0.131127000	2.031278000
C	-1.278333000	-1.593435000	3.402527000
C	-3.425028000	-0.571540000	3.311708000
C	-2.490442000	-1.317709000	4.005215000
C	-2.905800000	0.306122000	-1.102357000
C	-3.739776000	-0.795310000	-1.245684000
C	-3.255069000	1.389558000	-1.893821000
C	-4.856545000	-0.829095000	-2.059991000
C	-4.367334000	1.405077000	-2.725171000
C	-5.177595000	0.289982000	-2.807263000
F	-4.091679000	0.611155000	1.441440000
F	-4.598111000	-0.282199000	3.884108000
F	-2.750607000	-1.754868000	5.238644000

F	-0.353258000	-2.301687000	4.060849000
F	0.197681000	-1.415588000	1.644256000
F	-3.471110000	-1.931996000	-0.565458000
F	-5.619365000	-1.924207000	-2.141764000
F	-2.515468000	2.511705000	-1.908538000
F	-6.246610000	0.285691000	-3.605153000
F	-4.655691000	2.486709000	-3.456571000
O	-0.607269000	-0.711204000	-0.854096000
H	-1.037764000	-1.546369000	-1.041468000
H	1.454371000	-1.541614000	-0.447531000

H4

	Coordinates (Angstroms)		
	X	Y	Z
C	5.420641000	3.343749000	-0.052753000
C	4.598286000	4.414018000	-0.139189000
C	3.178959000	4.262875000	-0.241983000
C	2.579127000	2.990784000	-0.289841000
C	3.474592000	1.842726000	-0.247911000
C	4.870462000	2.031280000	-0.097136000
H	2.893240000	6.408316000	-0.246195000
H	6.493590000	3.463766000	0.050802000
H	5.000583000	5.421125000	-0.110930000
C	2.384502000	5.450482000	-0.265337000
C	1.121925000	2.914611000	-0.327667000
C	0.373226000	4.120365000	-0.296137000
C	1.034088000	5.383713000	-0.287362000
H	0.432564000	6.286940000	-0.286168000
C	5.686585000	0.887354000	0.011033000
C	3.743720000	-0.472647000	-0.256321000
H	6.755435000	1.016676000	0.146864000
C	-1.038511000	4.066655000	-0.247488000
C	0.382229000	1.704087000	-0.346765000
C	-1.706516000	2.872247000	-0.231178000
H	-2.789080000	2.869487000	-0.170710000
C	-0.995478000	1.651901000	-0.301172000
H	-1.587443000	5.002596000	-0.211830000
H	0.951723000	0.792274000	-0.378833000
N	2.971336000	0.592293000	-0.342362000
B	-1.743014000	0.245706000	-0.114790000
C	5.135277000	-0.364607000	-0.052354000
H	5.752520000	-1.247418000	0.040310000
P	2.780104000	-2.040952000	-0.479000000
C	3.313321000	-3.171142000	0.949437000
C	3.357800000	-2.299296000	2.215497000
C	2.179351000	-4.199177000	1.116025000
C	4.639243000	-3.923401000	0.813561000
H	4.223689000	-1.632892000	2.223567000
H	2.457156000	-1.690704000	2.329779000
H	3.429665000	-2.950960000	3.093440000
H	2.052228000	-4.821332000	0.226020000
H	2.412923000	-4.864215000	1.955049000
H	1.226125000	-3.709193000	1.331660000
H	4.827799000	-4.474066000	1.742792000
H	4.612429000	-4.653620000	0.001884000
H	5.487510000	-3.255395000	0.652606000
C	3.438302000	-2.663911000	-2.147005000

C	2.757814000	-4.009508000	-2.430894000
C	2.970137000	-1.625725000	-3.181442000
C	4.955353000	-2.814882000	-2.308344000
H	3.120737000	-4.793807000	-1.761552000
H	1.669923000	-3.948690000	-2.324540000
H	2.975836000	-4.320646000	-3.458560000
H	3.458057000	-0.658435000	-3.030839000
H	3.231342000	-1.976659000	-4.185878000
H	1.891513000	-1.459956000	-3.159922000
H	5.163738000	-3.155693000	-3.329382000
H	5.472498000	-1.862598000	-2.175212000
H	5.387798000	-3.544337000	-1.625481000
C	-1.849137000	-0.043343000	1.489353000
C	-0.928003000	-0.718980000	2.269237000
C	-2.887701000	0.538536000	2.207154000
C	-1.009292000	-0.841555000	3.644689000
C	-3.013070000	0.447533000	3.582174000
C	-2.065786000	-0.250271000	4.310332000
C	-3.102782000	-0.093733000	-0.923218000
C	-3.882078000	-1.192569000	-0.575173000
C	-3.569967000	0.634268000	-2.012042000
C	-5.063213000	-1.527556000	-1.213864000
C	-4.750820000	0.335071000	-2.671489000
C	-5.505681000	-0.749905000	-2.267718000
F	-3.837320000	1.240040000	1.567193000
F	-4.034786000	1.027674000	4.209694000
F	-2.169555000	-0.348010000	5.632194000
F	-0.080874000	-1.514794000	4.322412000
F	0.147061000	-1.313804000	1.694328000
F	-3.495883000	-2.010448000	0.416932000
F	-5.769830000	-2.591061000	-0.828579000
F	-2.878107000	1.663661000	-2.505631000
F	-6.637283000	-1.052584000	-2.895769000
F	-5.155799000	1.073873000	-3.705223000
O	-0.674802000	-0.967829000	-0.714939000
H	0.029923000	-1.349282000	-0.148377000
H	0.093008000	-2.275026000	-2.079756000
C	-0.533460000	-1.379133000	-2.031043000
O	-1.057970000	-0.813788000	-2.924695000

H5

	Coordinates (Angstroms)		
	X	Y	Z
B	1.795812000	0.216452000	-0.119895000
C	2.780029000	0.614510000	1.112251000
C	4.127755000	0.884807000	0.920954000
C	2.317219000	0.795647000	2.406909000
C	4.968918000	1.288748000	1.945126000
C	3.123806000	1.187625000	3.459741000
C	4.464380000	1.435680000	3.224279000
C	2.481871000	-0.809038000	-1.169616000
C	2.624544000	-0.587119000	-2.530441000
C	2.979941000	-2.022136000	-0.709319000
C	3.235544000	-1.497283000	-3.380565000
C	3.586554000	-2.958181000	-1.524405000
C	3.719028000	-2.688966000	-2.875587000
F	4.677904000	0.786604000	-0.299006000

F	6.255877000	1.541132000	1.707317000
F	5.259943000	1.819209000	4.218514000
F	2.625314000	1.332078000	4.686935000
F	1.020086000	0.577866000	2.682173000
F	2.857460000	-2.335878000	0.590434000
F	4.038729000	-4.110102000	-1.028232000
F	4.303078000	-3.571857000	-3.680401000
F	3.356838000	-1.231601000	-4.681926000
F	2.177465000	0.538077000	-3.100746000
C	1.021158000	3.744342000	-1.461237000
C	-0.390042000	3.812547000	-1.444996000
C	-1.156416000	2.693271000	-1.018055000
C	0.955785000	1.478827000	-0.635120000
C	1.678277000	2.616726000	-1.061631000
C	-3.552269000	1.766974000	-0.600355000
C	-4.944614000	2.042952000	-0.566794000
C	-5.821622000	1.026822000	-0.145039000
C	-3.943963000	-0.416651000	0.114057000
H	-6.885604000	1.235460000	-0.100966000
H	2.763320000	2.596106000	-1.080243000
H	1.574305000	4.618159000	-1.790828000
C	-2.614409000	2.814389000	-0.997733000
C	-5.450593000	3.314944000	-0.953814000
C	-3.179357000	4.048130000	-1.382929000
C	-1.019534000	5.025102000	-1.846682000
C	-2.364134000	5.138798000	-1.810897000
C	-4.591134000	4.274088000	-1.357402000
H	-6.520619000	3.488703000	-0.920681000
H	-4.954531000	5.248563000	-1.665473000
H	-2.854190000	6.061023000	-2.103604000
H	-0.396332000	5.852144000	-2.170442000
N	-3.106464000	0.529562000	-0.276167000
C	-0.425004000	1.538454000	-0.619204000
H	-0.991947000	0.682647000	-0.288130000
O	-0.252792000	-1.867561000	-0.915530000
H	-1.144468000	-1.946040000	-0.500952000
H	0.663299000	-0.848343000	0.049464000
H	0.925220000	-0.469920000	0.731265000
H	-0.403249000	-1.446088000	-1.768600000
C	-5.331954000	-0.199030000	0.209997000
H	-5.995334000	-0.979195000	0.554325000
P	-3.082776000	-1.997512000	0.546667000
C	-4.218941000	-3.429223000	0.062470000
C	-5.398927000	-3.766455000	0.978982000
C	-4.731949000	-3.123711000	-1.354314000
C	-3.299984000	-4.662737000	-0.027643000
H	-5.068326000	-4.082268000	1.970401000
H	-6.097440000	-2.937110000	1.101828000
H	-5.957136000	-4.600977000	0.538928000
H	-3.920752000	-2.817874000	-2.023587000
H	-5.184343000	-4.028732000	-1.774027000
H	-5.490741000	-2.338584000	-1.360218000
H	-3.883621000	-5.520576000	-0.379763000
H	-2.480249000	-4.500570000	-0.733413000
H	-2.867282000	-4.930930000	0.938431000
C	-2.911093000	-1.829020000	2.421514000
C	-2.382934000	-3.152224000	2.990673000

C	-1.831101000	-0.753597000	2.622704000
C	-4.182369000	-1.406802000	3.163001000
H	-3.131853000	-3.946048000	2.955941000
H	-1.490076000	-3.494823000	2.458515000
H	-2.108594000	-3.003007000	4.040819000
H	-2.132320000	0.216969000	2.220496000
H	-1.641047000	-0.627239000	3.694114000
H	-0.890693000	-1.041727000	2.146202000
H	-3.962017000	-1.326308000	4.233809000
H	-4.539201000	-0.429108000	2.829273000
H	-4.993055000	-2.127351000	3.045151000

H6

	Coordinates (Angstroms)		
	X	Y	Z
B	1.804431000	0.283893000	-0.348436000
C	1.526983000	-0.570829000	1.028972000
C	1.217364000	-0.050020000	2.279091000
C	1.570388000	-1.957564000	0.986067000
C	0.924663000	-0.836615000	3.384198000
C	1.298960000	-2.783427000	2.062275000
C	0.960409000	-2.213600000	3.274896000
C	3.402503000	0.271256000	-0.689319000
C	3.906875000	-0.183782000	-1.898613000
C	4.362801000	0.705069000	0.213420000
C	5.259736000	-0.213479000	-2.201078000
C	5.723281000	0.697359000	-0.044173000
C	6.175639000	0.231012000	-1.265684000
F	1.182934000	1.275026000	2.487361000
F	0.605552000	-0.277155000	4.555535000
F	0.657328000	-2.984486000	4.321366000
F	1.333890000	-4.114181000	1.938852000
F	1.881322000	-2.586968000	-0.171951000
F	3.985500000	1.178665000	1.415434000
F	6.600558000	1.132687000	0.865745000
F	7.481312000	0.213237000	-1.539556000
F	5.689716000	-0.665980000	-3.383654000
F	3.080928000	-0.633190000	-2.862441000
C	1.231965000	4.188366000	-0.428557000
C	-0.166841000	4.276110000	-0.270811000
C	-0.940078000	3.083149000	-0.197036000
C	1.146434000	1.766821000	-0.339242000
C	1.868461000	2.972118000	-0.467080000
C	-3.317402000	2.088899000	-0.060038000
C	-4.689210000	2.291296000	0.253619000
C	-5.542502000	1.173354000	0.289776000
C	-3.715145000	-0.163504000	-0.392249000
H	-6.584780000	1.315240000	0.554228000
H	2.948091000	2.946834000	-0.583555000
H	1.800089000	5.111375000	-0.499452000
C	-2.385511000	3.203820000	-0.041995000
C	-5.180659000	3.604447000	0.501417000
C	-2.939587000	4.482890000	0.145427000
C	-0.791231000	5.552136000	-0.148797000
C	-2.121296000	5.652193000	0.077942000
C	-4.334445000	4.655872000	0.416349000
H	-6.231460000	3.741080000	0.730976000

H	-4.698845000	5.666012000	0.570370000
H	-2.594117000	6.619818000	0.206500000
H	-0.172259000	6.441433000	-0.213054000
N	-2.888134000	0.857854000	-0.408450000
C	-0.230842000	1.860650000	-0.226889000
H	-0.790828000	0.944256000	-0.140605000
H	1.235848000	-0.336967000	-1.223461000
O	0.107349000	-2.269240000	-2.591285000
H	0.328909000	-1.499829000	-3.124090000
H	0.777232000	-2.257782000	-1.899616000
C	-5.063933000	-0.075926000	-0.014833000
H	-5.702826000	-0.948463000	0.017303000
P	-2.902455000	-1.722126000	-0.881511000
C	-3.667244000	-2.414966000	-2.417325000
C	-5.179937000	-2.616189000	-2.273716000
C	-3.378498000	-1.372056000	-3.510446000
C	-2.979831000	-3.741710000	-2.762280000
H	-5.438970000	-3.280773000	-1.447507000
H	-5.707474000	-1.668732000	-2.150415000
H	-5.543876000	-3.076960000	-3.196758000
H	-2.305561000	-1.235139000	-3.664584000
H	-3.814069000	-1.731324000	-4.447217000
H	-3.826986000	-0.401219000	-3.282640000
H	-3.313188000	-4.043323000	-3.759940000
H	-1.891809000	-3.643199000	-2.782661000
H	-3.261951000	-4.532883000	-2.064922000
C	-2.684461000	-2.807064000	0.603385000
C	-1.585950000	-3.839249000	0.310813000
C	-2.210133000	-1.869966000	1.727721000
C	-3.990330000	-3.504359000	0.993542000
H	-1.904731000	-4.591014000	-0.410258000
H	-0.672850000	-3.370081000	-0.059756000
H	-1.347493000	-4.351638000	1.246620000
H	-2.984684000	-1.161487000	2.029076000
H	-1.954426000	-2.482571000	2.596929000
H	-1.316521000	-1.307924000	1.440367000
H	-3.825655000	-4.037886000	1.934284000
H	-4.805052000	-2.792894000	1.152360000
H	-4.298268000	-4.234719000	0.242358000
H	-1.608425000	-1.331845000	-1.249304000

H7

	Coordinates (Angstroms)		
	X	Y	Z
B	1.756984000	0.295388000	-0.341961000
C	1.512460000	-0.834592000	0.829486000
C	1.325672000	-0.575729000	2.181878000
C	1.464213000	-2.183135000	0.495193000
C	1.092754000	-1.561865000	3.128845000
C	1.247646000	-3.201384000	1.409046000
C	1.047965000	-2.887160000	2.739684000
C	3.347677000	0.384022000	-0.723965000
C	3.787051000	0.377629000	-2.041101000
C	4.359836000	0.487256000	0.219962000
C	5.122837000	0.450414000	-2.404778000
C	5.706798000	0.562919000	-0.095137000
C	6.091798000	0.541917000	-1.422977000

F	1.364382000	0.679989000	2.654028000
F	0.897581000	-1.244888000	4.413313000
F	0.795339000	-3.848051000	3.630852000
F	1.195846000	-4.479711000	1.014862000
F	1.618639000	-2.571783000	-0.785844000
F	4.058039000	0.535933000	1.531204000
F	6.634186000	0.659919000	0.863193000
F	7.382695000	0.614406000	-1.753159000
F	5.486175000	0.434535000	-3.692008000
F	2.908065000	0.300491000	-3.056450000
C	1.191189000	4.096223000	0.587362000
C	-0.217391000	4.189797000	0.562585000
C	-0.991484000	3.037394000	0.249236000
C	1.105194000	1.744129000	0.007127000
C	1.833258000	2.912845000	0.319160000
C	-3.372732000	2.085002000	-0.071388000
C	-4.777851000	2.300396000	-0.055412000
C	-5.630004000	1.215834000	-0.339817000
C	-3.714994000	-0.132653000	-0.629895000
H	-6.702776000	1.375402000	-0.325615000
H	2.918671000	2.883041000	0.351687000
H	1.759707000	4.990489000	0.826326000
C	-2.446591000	3.165265000	0.226276000
C	-5.296594000	3.592106000	0.242898000
C	-3.020233000	4.417061000	0.515177000
C	-0.852113000	5.434628000	0.845147000
C	-2.200474000	5.545731000	0.822538000
C	-4.440137000	4.603227000	0.513985000
H	-6.370181000	3.743852000	0.246949000
H	-4.819594000	5.593921000	0.740924000
H	-2.685627000	6.491783000	1.037279000
H	-0.230422000	6.292701000	1.080586000
N	-2.897742000	0.860094000	-0.368450000
C	-0.274672000	1.847809000	-0.025087000
H	-0.836272000	0.966354000	-0.277717000
H	1.171098000	-0.126455000	-1.322107000
C	-5.112416000	-0.023090000	-0.627800000
H	-5.749392000	-0.871119000	-0.844408000
P	-2.799235000	-1.672679000	-0.956845000
H	-1.472013000	-1.231705000	-1.041006000
C	-3.187427000	-2.377386000	-2.621491000
C	-3.319399000	-1.176429000	-3.573134000
C	-1.997107000	-3.244477000	-3.061652000
C	-4.481163000	-3.194893000	-2.601176000
H	-4.200351000	-0.570652000	-3.350656000
H	-2.435730000	-0.531896000	-3.546906000
H	-3.422647000	-1.563319000	-4.590858000
H	-1.879388000	-4.135879000	-2.444923000
H	-2.181267000	-3.571164000	-4.089119000
H	-1.059100000	-2.683075000	-3.050764000
H	-4.705605000	-3.505340000	-3.625778000
H	-4.380975000	-4.095515000	-1.991990000
H	-5.330628000	-2.612692000	-2.235354000
C	-2.868410000	-2.752935000	0.542650000
C	-2.064682000	-4.029949000	0.271889000
C	-2.202811000	-1.924887000	1.654890000
C	-4.311164000	-3.086275000	0.935999000

H	-2.587401000	-4.689751000	-0.424300000
H	-1.070075000	-3.816599000	-0.126933000
H	-1.939102000	-4.564310000	1.217878000
H	-2.790740000	-1.041640000	1.915603000
H	-2.120507000	-2.551497000	2.547463000
H	-1.200418000	-1.597718000	1.372929000
H	-4.279208000	-3.690075000	1.847726000
H	-4.889191000	-2.185891000	1.157035000
H	-4.830577000	-3.664818000	0.170554000

H8

	Coordinates (Angstroms)		
	X	Y	Z
B	1.802359000	0.016102000	-0.050231000
C	2.633503000	0.755143000	1.162030000
C	3.969287000	1.123940000	1.080130000
C	1.991169000	1.187246000	2.313255000
C	4.630994000	1.829701000	2.071618000
C	2.609417000	1.888023000	3.333169000
C	3.948488000	2.209425000	3.212183000
C	2.686475000	-0.995607000	-0.967789000
C	2.542063000	-1.122282000	-2.343801000
C	3.617436000	-1.857710000	-0.396699000
C	3.295935000	-2.003662000	-3.106169000
C	4.384154000	-2.751574000	-1.119615000
C	4.224084000	-2.821128000	-2.492342000
F	4.699712000	0.817935000	-0.006179000
F	5.918922000	2.147469000	1.934202000
F	4.569272000	2.884893000	4.176191000
F	1.928574000	2.262064000	4.416609000
F	0.668702000	0.951804000	2.480194000
F	3.794156000	-1.867325000	0.934626000
F	5.263011000	-3.549701000	-0.511210000
F	4.949414000	-3.673735000	-3.209848000
F	3.122122000	-2.075461000	-4.426739000
F	1.647649000	-0.396068000	-3.021074000
C	1.216028000	3.262028000	-2.041443000
C	-0.187605000	3.427277000	-2.043809000
C	-1.021449000	2.447522000	-1.439387000
C	1.005768000	1.184975000	-0.822968000
C	1.796513000	2.182216000	-1.442647000
C	-3.468300000	1.749348000	-0.907449000
C	-4.844501000	2.093870000	-0.958864000
C	-5.783266000	1.196622000	-0.416537000
C	-3.989036000	-0.272761000	0.127004000
H	-6.836410000	1.456732000	-0.445210000
H	2.878393000	2.086545000	-1.447854000
H	1.824687000	4.023463000	-2.519196000
C	-2.468212000	2.661406000	-1.455407000
C	-5.272196000	3.313299000	-1.553839000
C	-2.957077000	3.845034000	-2.047059000
C	-0.740120000	4.593051000	-2.645975000
C	-2.075085000	4.796932000	-2.640523000
C	-4.354151000	4.149772000	-2.082848000
H	-6.331603000	3.544267000	-1.578480000
H	-4.658650000	5.080173000	-2.550098000
H	-2.505107000	5.686220000	-3.088310000

H	-0.066429000	5.313399000	-3.098354000
O	0.781027000	-0.971879000	0.715567000
N	-3.094619000	0.564246000	-0.368699000
C	-0.365980000	1.331920000	-0.842714000
H	-0.998678000	0.578654000	-0.403658000
C	0.299514000	-2.284103000	0.233408000
H	-0.211952000	-2.704469000	1.102729000
H	1.201766000	-2.841141000	0.002398000
O	-0.485553000	-2.172569000	-0.863281000
H	-1.394300000	-1.986891000	-0.537475000
H	0.077310000	-0.499208000	1.183393000
C	-5.367477000	0.016174000	0.133627000
H	-6.077944000	-0.677252000	0.560922000
P	-3.225653000	-1.827824000	0.778136000
C	-4.291049000	-3.255917000	0.146149000
C	-4.519414000	-2.987848000	-1.350554000
C	-3.413804000	-4.515791000	0.271313000
C	-5.635493000	-3.515078000	0.830546000
H	-5.219887000	-2.166777000	-1.520404000
H	-3.584817000	-2.751634000	-1.870064000
H	-4.939061000	-3.886718000	-1.814834000
H	-3.210351000	-4.777717000	1.311800000
H	-3.932795000	-5.363761000	-0.188845000
H	-2.456434000	-4.388943000	-0.242708000
H	-6.114615000	-4.377966000	0.353469000
H	-5.519507000	-3.749361000	1.890351000
H	-6.322601000	-2.672392000	0.737977000
C	-3.371387000	-1.611203000	2.650753000
C	-3.037277000	-2.940263000	3.339853000
C	-2.284244000	-0.588217000	3.020894000
C	-4.724542000	-1.091679000	3.147237000
H	-3.831294000	-3.680010000	3.219381000
H	-2.105098000	-3.372251000	2.962180000
H	-2.909116000	-2.762822000	4.413273000
H	-2.373249000	0.342453000	2.452796000
H	-2.367609000	-0.338106000	4.084116000
H	-1.284914000	-1.004435000	2.864354000
H	-4.709974000	-1.055214000	4.242514000
H	-4.920994000	-0.078491000	2.789430000
H	-5.555985000	-1.731087000	2.846800000

TSH2-3

	Coordinates (Angstroms)		
	X	Y	Z
C	5.998354000	2.407408000	-0.258669000
C	5.407579000	3.586247000	0.065501000
C	3.988975000	3.704754000	0.242110000
C	3.157802000	2.603543000	-0.000295000
C	3.799415000	1.336978000	-0.271799000
C	5.201470000	1.231428000	-0.400782000
H	4.079405000	5.770229000	0.898028000
H	7.073664000	2.336660000	-0.381280000
H	6.012865000	4.474172000	0.217256000
C	3.421066000	4.925409000	0.724842000
C	1.724946000	2.737401000	0.165501000
C	1.219685000	3.905427000	0.781647000
C	2.099436000	5.004525000	1.028208000

H	1.688544000	5.911375000	1.460720000
C	5.736168000	-0.057832000	-0.619603000
C	3.529609000	-0.954188000	-0.528478000
H	6.810445000	-0.171412000	-0.722626000
C	-0.148841000	3.942571000	1.150010000
C	0.793489000	1.737316000	-0.205517000
C	-0.970112000	2.864096000	0.932707000
H	-1.996335000	2.898719000	1.288667000
C	-0.513253000	1.731131000	0.212790000
H	-0.529701000	4.833575000	1.640070000
H	1.143964000	0.914367000	-0.794285000
N	3.030322000	0.239441000	-0.324130000
B	-1.398040000	0.398556000	-0.007005000
C	4.915207000	-1.160836000	-0.688537000
H	5.324448000	-2.151065000	-0.843445000
P	2.129460000	-2.150817000	-0.573968000
C	2.422669000	-3.461878000	0.729025000
C	2.965374000	-2.736154000	1.970874000
C	1.055916000	-4.074442000	1.073755000
C	3.403669000	-4.555781000	0.304270000
H	3.977072000	-2.355096000	1.811995000
H	2.326278000	-1.903244000	2.273263000
H	3.000368000	-3.448841000	2.801208000
H	0.595542000	-4.574672000	0.219372000
H	1.191199000	-4.821748000	1.862650000
H	0.365301000	-3.314485000	1.447677000
H	3.593728000	-5.211931000	1.160481000
H	3.001515000	-5.171931000	-0.502291000
H	4.362703000	-4.141877000	-0.017099000
C	2.060080000	-2.789907000	-2.333911000
C	0.970862000	-3.866922000	-2.422463000
C	1.637411000	-1.570555000	-3.174397000
C	3.383627000	-3.331011000	-2.883357000
H	1.271024000	-4.790652000	-1.923008000
H	0.022271000	-3.532275000	-1.990279000
H	0.789846000	-4.100074000	-3.476720000
H	2.380708000	-0.769258000	-3.130314000
H	1.541605000	-1.878816000	-4.220485000
H	0.676177000	-1.163581000	-2.850914000
H	3.223846000	-3.646470000	-3.920137000
H	4.160457000	-2.563368000	-2.892952000
H	3.751225000	-4.194294000	-2.328562000
C	-1.990680000	-0.049085000	1.462725000
C	-1.155410000	-0.642143000	2.400868000
C	-3.284759000	0.179913000	1.906596000
C	-1.567895000	-1.038631000	3.660683000
C	-3.739680000	-0.189958000	3.163165000
C	-2.876792000	-0.812112000	4.045568000
C	-2.574429000	0.474674000	-1.156591000
C	-3.321813000	-0.658205000	-1.451341000
C	-2.896892000	1.585751000	-1.921769000
C	-4.320708000	-0.715170000	-2.403073000
C	-3.893843000	1.579126000	-2.888398000
C	-4.611731000	0.424705000	-3.131525000
F	-4.180653000	0.804611000	1.121330000
F	-5.000749000	0.051618000	3.529736000
F	-3.297481000	-1.179121000	5.255069000

F	-0.716536000	-1.628061000	4.503988000
F	0.141560000	-0.855404000	2.115538000
F	-3.076471000	-1.806472000	-0.774905000
F	-4.998374000	-1.842621000	-2.626581000
F	-2.258042000	2.753010000	-1.764511000
F	-5.567240000	0.406059000	-4.058411000
F	-4.162110000	2.681953000	-3.591027000
O	-0.470947000	-0.764480000	-0.522839000
H	-1.003996000	-1.547502000	-0.699137000
H	0.658520000	-1.232976000	-0.365759000

TSH3-4

	Coordinates (Angstroms)		
	X	Y	Z
C	5.457893000	3.344894000	-0.745778000
C	4.612355000	4.288871000	-1.212932000
C	3.210301000	4.038385000	-1.350273000
C	2.646503000	2.790659000	-1.016577000
C	3.578231000	1.746169000	-0.605480000
C	4.955171000	2.050078000	-0.433563000
H	2.882872000	6.046223000	-2.086349000
H	6.515474000	3.545074000	-0.613657000
H	4.979259000	5.275006000	-1.477060000
C	2.397752000	5.112365000	-1.823421000
C	1.194791000	2.649656000	-1.086574000
C	0.427864000	3.750933000	-1.552943000
C	1.058207000	4.969076000	-1.936431000
H	0.439300000	5.783959000	-2.297917000
C	5.812147000	1.042330000	0.045814000
C	3.967699000	-0.454522000	0.031156000
H	6.858707000	1.276190000	0.210925000
C	-0.981287000	3.651969000	-1.605078000
C	0.470962000	1.500053000	-0.669145000
C	-1.628525000	2.517083000	-1.202540000
H	-2.710907000	2.480025000	-1.252109000
C	-0.906268000	1.398276000	-0.723809000
H	-1.543210000	4.508860000	-1.964325000
H	1.037960000	0.675842000	-0.273926000
N	3.142364000	0.482246000	-0.398404000
B	-1.656652000	0.090864000	-0.118605000
C	5.325547000	-0.211381000	0.304165000
H	5.963277000	-0.988246000	0.702315000
P	3.116894000	-2.059900000	0.308086000
C	3.038691000	-2.266707000	2.173530000
C	2.735676000	-0.877303000	2.757543000
C	1.850395000	-3.191983000	2.479734000
C	4.316788000	-2.821641000	2.804472000
H	3.597420000	-0.208993000	2.689945000
H	1.889559000	-0.396573000	2.260231000
H	2.480007000	-0.992415000	3.815738000
H	1.975129000	-4.189826000	2.054487000
H	1.753167000	-3.301734000	3.564846000
H	0.914593000	-2.769057000	2.104315000
H	4.197879000	-2.825676000	3.893623000
H	4.516030000	-3.848268000	2.490168000
H	5.189053000	-2.206422000	2.568987000
C	4.068972000	-3.395560000	-0.599686000

C	3.409202000	-4.740251000	-0.262393000
C	3.838303000	-3.081857000	-2.089030000
C	5.577072000	-3.475024000	-0.343745000
H	3.627023000	-5.055348000	0.760563000
H	2.322807000	-4.707431000	-0.392166000
H	3.801278000	-5.506633000	-0.938985000
H	4.233016000	-2.099093000	-2.363696000
H	4.358270000	-3.830923000	-2.695250000
H	2.778331000	-3.114243000	-2.355738000
H	5.970816000	-4.350994000	-0.870730000
H	6.097005000	-2.600069000	-0.737868000
H	5.824276000	-3.585909000	0.712461000
C	-2.350113000	0.567074000	1.307893000
C	-1.744242000	0.449133000	2.551328000
C	-3.582281000	1.209572000	1.333803000
C	-2.300379000	0.923527000	3.728926000
C	-4.174645000	1.700915000	2.484418000
C	-3.527525000	1.556651000	3.697368000
C	-2.787772000	-0.665259000	-1.060203000
C	-3.493750000	-1.726950000	-0.507924000
C	-3.081768000	-0.413055000	-2.391548000
C	-4.451603000	-2.461132000	-1.183079000
C	-4.037240000	-1.119526000	-3.108470000
C	-4.731336000	-2.147678000	-2.500633000
F	-4.279374000	1.394759000	0.196233000
F	-5.361869000	2.309628000	2.434699000
F	-4.079000000	2.022044000	4.817403000
F	-1.660664000	0.772218000	4.891585000
F	-0.547778000	-0.155228000	2.685389000
F	-3.248426000	-2.103651000	0.765780000
F	-5.097384000	-3.466122000	-0.585659000
F	-2.436339000	0.539626000	-3.079759000
F	-5.647962000	-2.837764000	-3.178056000
F	-4.283261000	-0.822709000	-4.387425000
O	-0.577483000	-0.950111000	0.154577000
H	-0.829184000	-1.631179000	0.786144000
H	1.515084000	-1.927260000	-0.525477000
C	0.434776000	-1.696054000	-1.227301000
O	-0.042747000	-1.591433000	-2.257103000

TSH5-6

	Coordinates (Angstroms)		
	X	Y	Z
B	1.771120000	0.200270000	-0.113174000
C	2.751302000	0.617973000	1.118312000
C	4.093402000	0.916954000	0.931791000
C	2.284303000	0.780805000	2.413863000
C	4.926476000	1.329556000	1.959122000
C	3.082500000	1.182042000	3.470068000
C	4.418182000	1.457978000	3.238725000
C	2.491411000	-0.807213000	-1.163117000
C	2.640612000	-0.587947000	-2.523819000
C	3.022087000	-2.003684000	-0.694821000
C	3.290830000	-1.480347000	-3.363942000
C	3.669145000	-2.922149000	-1.499120000
C	3.808341000	-2.653478000	-2.849646000
F	4.647700000	0.837630000	-0.288782000

F	6.209167000	1.608203000	1.724315000
F	5.205180000	1.850475000	4.237181000
F	2.579881000	1.309016000	4.698247000
F	0.991838000	0.538288000	2.689216000
F	2.896931000	-2.318403000	0.604718000
F	4.153630000	-4.056881000	-0.993237000
F	4.429842000	-3.519559000	-3.645189000
F	3.416584000	-1.215743000	-4.665412000
F	2.161190000	0.518440000	-3.106022000
C	1.005192000	3.730145000	-1.477847000
C	-0.405905000	3.803401000	-1.451180000
C	-1.171501000	2.686748000	-1.016986000
C	0.940134000	1.466656000	-0.641545000
C	1.661487000	2.600801000	-1.081167000
C	-3.564842000	1.758009000	-0.600609000
C	-4.957708000	2.030557000	-0.564555000
C	-5.832309000	1.008068000	-0.152995000
C	-3.950943000	-0.431351000	0.097505000
H	-6.896971000	1.213132000	-0.108787000
H	2.746280000	2.576298000	-1.108660000
H	1.558769000	4.601505000	-1.813419000
C	-2.628857000	2.808510000	-0.992610000
C	-5.465221000	3.305297000	-0.940919000
C	-3.195215000	4.043253000	-1.371858000
C	-1.036436000	5.017101000	-1.847724000
C	-2.380800000	5.133837000	-1.801438000
C	-4.607215000	4.267809000	-1.340160000
H	-6.535230000	3.478350000	-0.904350000
H	-4.972394000	5.243878000	-1.641074000
H	-2.870901000	6.057737000	-2.088792000
H	-0.414279000	5.843368000	-2.175579000
N	-3.116129000	0.519668000	-0.285065000
C	-0.440045000	1.533369000	-0.614375000
H	-1.008059000	0.684329000	-0.269411000
O	-0.238311000	-1.840140000	-0.836597000
H	-1.146342000	-1.923502000	-0.439047000
H	0.586782000	-0.917272000	-0.005136000
H	0.917620000	-0.495779000	0.671796000
H	-0.366030000	-1.459208000	-1.713742000
C	-5.339802000	-0.219912000	0.191751000
H	-6.001349000	-1.005731000	0.526844000
P	-3.083077000	-2.008715000	0.525817000
C	-4.197756000	-3.444753000	0.011084000
C	-5.385950000	-3.797561000	0.911083000
C	-4.696094000	-3.126571000	-1.408114000
C	-3.269179000	-4.670627000	-0.080958000
H	-5.064311000	-4.119247000	1.903517000
H	-6.091193000	-2.973693000	1.032329000
H	-5.933057000	-4.632100000	0.457503000
H	-3.878241000	-2.813201000	-2.065659000
H	-5.142387000	-4.028436000	-1.840770000
H	-5.456024000	-2.342521000	-1.414602000
H	-3.841632000	-5.527148000	-0.453776000
H	-2.439901000	-4.493696000	-0.771890000
H	-2.849715000	-4.949474000	0.887886000
C	-2.926619000	-1.859729000	2.402679000
C	-2.396735000	-3.186034000	2.962926000

C	-1.853736000	-0.780869000	2.622248000
C	-4.205909000	-1.450771000	3.137692000
H	-3.142531000	-3.982242000	2.916719000
H	-1.499062000	-3.520534000	2.433749000
H	-2.129644000	-3.044655000	4.015953000
H	-2.154538000	0.190876000	2.222442000
H	-1.676564000	-0.659954000	3.696437000
H	-0.905957000	-1.061456000	2.156186000
H	-3.993758000	-1.380440000	4.210791000
H	-4.564199000	-0.471174000	2.811373000
H	-5.012501000	-2.173598000	3.006637000

TSH7

	Coordinates (Angstroms)		
	X	Y	Z
B	-1.940987000	0.188252000	0.088041000
C	-1.505110000	-0.895466000	-1.043188000
C	-1.137773000	-0.581684000	-2.344862000
C	-1.471200000	-2.249723000	-0.740068000
C	-0.714018000	-1.527604000	-3.264909000
C	-1.062387000	-3.227975000	-1.629767000
C	-0.668566000	-2.860573000	-2.902190000
C	-3.528901000	0.184936000	0.443399000
C	-3.964026000	0.642806000	1.679090000
C	-4.542795000	-0.220275000	-0.412001000
C	-5.290064000	0.687669000	2.068384000
C	-5.886134000	-0.195939000	-0.067218000
C	-6.262690000	0.256696000	1.183249000
F	-1.199952000	0.682581000	-2.789605000
F	-0.345600000	-1.165603000	-4.497089000
F	-0.231904000	-3.780067000	-3.763490000
F	-1.024661000	-4.513883000	-1.267695000
F	-1.855195000	-2.674810000	0.477129000
F	-4.263325000	-0.649831000	-1.653217000
F	-6.819064000	-0.601167000	-0.931471000
F	-7.548182000	0.285370000	1.531346000
F	-5.640239000	1.137511000	3.275250000
F	-3.063313000	1.096873000	2.582153000
C	-1.552945000	4.064655000	-0.308606000
C	-0.158393000	4.213619000	-0.455855000
C	0.687255000	3.072976000	-0.363500000
C	-1.320161000	1.663606000	-0.085967000
C	-2.116841000	2.828831000	-0.122442000
C	3.127280000	2.222008000	-0.344765000
C	4.474840000	2.464447000	-0.715324000
C	5.396110000	1.407203000	-0.599578000
C	3.668574000	0.075932000	0.361047000
H	6.421463000	1.564307000	-0.918001000
H	-3.195916000	2.749360000	-0.031766000
H	-2.175807000	4.952110000	-0.369853000
C	2.125077000	3.260619000	-0.528745000
C	4.880761000	3.752394000	-1.166596000
C	2.595817000	4.532326000	-0.903718000
C	0.385703000	5.500380000	-0.744082000
C	1.706455000	5.647884000	-0.989819000
C	3.973957000	4.753760000	-1.218244000
H	5.919309000	3.914228000	-1.433950000

H	4.271326000	5.752123000	-1.521431000
H	2.119959000	6.615929000	-1.251423000
H	-0.288702000	6.349131000	-0.796032000
N	2.781820000	1.042132000	0.218251000
C	0.052856000	1.818634000	-0.188946000
H	0.674182000	0.937488000	-0.151814000
H	-1.401482000	-0.282934000	1.122050000
O	-0.013696000	0.639329000	2.715852000
C	-0.558554000	-0.534083000	2.554519000
H	-1.527321000	-0.747220000	2.997127000
O	0.192095000	-1.524000000	2.300390000
H	-0.694040000	1.315596000	2.853661000
C	4.999562000	0.201860000	-0.084690000
H	5.694982000	-0.621799000	-0.011781000
P	2.958113000	-1.436920000	1.143508000
H	1.143342000	-1.304567000	1.890375000
C	2.947477000	-2.719758000	-0.236182000
C	1.962759000	-3.828976000	0.168077000
C	2.375140000	-1.996644000	-1.466481000
C	4.303640000	-3.336491000	-0.579762000
H	2.320980000	-4.413622000	1.016857000
H	0.980725000	-3.424390000	0.424195000
H	1.833455000	-4.514723000	-0.675797000
H	3.074145000	-1.261964000	-1.873644000
H	2.167630000	-2.734037000	-2.248627000
H	1.437048000	-1.482542000	-1.233667000
H	4.180682000	-4.014875000	-1.431779000
H	5.040301000	-2.581525000	-0.864100000
H	4.704327000	-3.921364000	0.251323000
C	4.078506000	-1.883186000	2.587686000
C	5.590298000	-1.903432000	2.338959000
C	3.773525000	-0.810271000	3.648107000
C	3.634180000	-3.253543000	3.117640000
H	5.876675000	-2.548864000	1.507311000
H	5.983368000	-0.901575000	2.157634000
H	6.083530000	-2.285798000	3.239684000
H	2.722145000	-0.822381000	3.949129000
H	4.381017000	-1.001238000	4.539288000
H	4.013216000	0.195237000	3.288337000
H	4.100527000	-3.423733000	4.093927000
H	2.550147000	-3.317250000	3.251748000
H	3.949714000	-4.062575000	2.454792000

TSH8

	Coordinates (Angstroms)		
	X	Y	Z
B	1.775852000	-0.026131000	0.019151000
C	2.781567000	0.817178000	1.037660000
C	4.114311000	1.106273000	0.783181000
C	2.287270000	1.393663000	2.199160000
C	4.911505000	1.870512000	1.619790000
C	3.042967000	2.161845000	3.068067000
C	4.373423000	2.399017000	2.778060000
C	2.563790000	-1.151414000	-0.879977000
C	2.304015000	-1.409481000	-2.218744000
C	3.492894000	-2.005209000	-0.295919000
C	2.942809000	-2.409795000	-2.939321000

C	4.149926000	-3.014055000	-0.975528000
C	3.873584000	-3.216245000	-2.315935000
F	4.713468000	0.653859000	-0.335162000
F	6.191139000	2.103162000	1.317215000
F	5.122578000	3.136168000	3.597463000
F	2.500409000	2.680340000	4.172745000
F	0.984229000	1.249470000	2.532559000
F	3.791558000	-1.885318000	1.009365000
F	5.037445000	-3.796617000	-0.355174000
F	4.492615000	-4.182124000	-2.992744000
F	2.657089000	-2.605197000	-4.229580000
F	1.396704000	-0.701811000	-2.904384000
C	1.229833000	3.059032000	-2.264898000
C	-0.162301000	3.294746000	-2.203106000
C	-1.003558000	2.406480000	-1.477374000
C	1.003255000	1.117051000	-0.837826000
C	1.794480000	2.009692000	-1.600488000
C	-3.458724000	1.848472000	-0.831432000
C	-4.817176000	2.269864000	-0.813380000
C	-5.777332000	1.439932000	-0.207178000
C	-4.051679000	-0.120230000	0.239434000
H	-6.811732000	1.765607000	-0.180376000
H	2.868541000	1.859521000	-1.659480000
H	1.842689000	3.743323000	-2.843637000
C	-2.438220000	2.689538000	-1.448032000
C	-5.207613000	3.497466000	-1.417450000
C	-2.898926000	3.867540000	-2.072736000
C	-0.691152000	4.443757000	-2.855787000
C	-2.008765000	4.730845000	-2.776579000
C	-4.276479000	4.252926000	-2.036775000
H	-6.250283000	3.793233000	-1.384018000
H	-4.554265000	5.181246000	-2.524361000
H	-2.418087000	5.615850000	-3.251177000
H	-0.011741000	5.089728000	-3.402285000
O	0.843797000	-0.854800000	0.905754000
N	-3.128286000	0.647869000	-0.303709000
C	-0.362140000	1.323948000	-0.806720000
H	-0.989524000	0.635006000	-0.264804000
C	0.025812000	-2.361060000	0.399359000
H	-0.340775000	-2.573258000	1.416926000
H	0.951911000	-2.884079000	0.151588000
O	-0.813230000	-2.140679000	-0.515956000
H	0.186502000	-0.295046000	1.331575000
C	-5.406647000	0.237290000	0.330581000
H	-6.127158000	-0.415790000	0.804008000
P	-3.382533000	-1.702844000	0.871184000
C	-4.394485000	-3.135380000	0.230933000
C	-5.704394000	-3.399339000	0.975342000
C	-4.677709000	-2.826707000	-1.248943000
C	-3.490935000	-4.380045000	0.307041000
H	-5.535185000	-3.651000000	2.024035000
H	-6.390276000	-2.551267000	0.926366000
H	-6.202813000	-4.253028000	0.504414000
H	-3.764502000	-2.579805000	-1.798712000
H	-5.115038000	-3.717230000	-1.710748000
H	-5.385806000	-2.003659000	-1.368581000
H	-4.012015000	-5.215920000	-0.169909000

H	-2.546516000	-4.226060000	-0.221083000
H	-3.271133000	-4.671084000	1.335640000
C	-3.294508000	-1.525109000	2.729402000
C	-2.899546000	-2.864885000	3.360944000
C	-2.193191000	-0.489493000	3.009815000
C	-4.615501000	-1.025026000	3.327053000
H	-3.696374000	-3.606929000	3.281821000
H	-1.991066000	-3.277282000	2.912127000
H	-2.700738000	-2.700319000	4.424540000
H	-2.347109000	0.449131000	2.470821000
H	-2.191815000	-0.262367000	4.080281000
H	-1.206432000	-0.886842000	2.763453000
H	-4.507687000	-0.993270000	4.415876000
H	-4.853235000	-0.013459000	2.991500000
H	-5.457673000	-1.678658000	3.095055000
H	-2.006349000	-1.863170000	0.152334000

TSH9

	Coordinates (Angstroms)		
	X	Y	Z
B	-1.986593000	0.314065000	-0.043017000
C	-1.494060000	-0.916523000	-0.949990000
C	-0.775531000	-0.810635000	-2.133899000
C	-1.735462000	-2.219929000	-0.523997000
C	-0.292681000	-1.909587000	-2.826952000
C	-1.265514000	-3.339954000	-1.185106000
C	-0.526763000	-3.181321000	-2.342187000
C	-3.556524000	0.324584000	0.327627000
C	-4.004077000	0.793632000	1.555013000
C	-4.551179000	-0.075860000	-0.552145000
C	-5.340086000	0.845481000	1.910569000
C	-5.900454000	-0.036785000	-0.238215000
C	-6.296546000	0.422057000	1.004528000
F	-0.515732000	0.383100000	-2.685015000
F	0.428881000	-1.748897000	-3.940128000
F	-0.020309000	-4.242793000	-2.968133000
F	-1.482619000	-4.564980000	-0.700149000
F	-2.472618000	-2.440589000	0.572683000
F	-4.237668000	-0.509760000	-1.783094000
F	-6.818935000	-0.432905000	-1.121610000
F	-7.588127000	0.463476000	1.325143000
F	-5.715316000	1.298735000	3.108198000
F	-3.118914000	1.234321000	2.464640000
C	-1.520835000	4.154072000	-0.456510000
C	-0.118644000	4.290321000	-0.507647000
C	0.712216000	3.141917000	-0.374367000
C	-1.321637000	1.755405000	-0.217791000
C	-2.107282000	2.924249000	-0.308265000
C	3.145944000	2.268838000	-0.241125000
C	4.522103000	2.526101000	-0.483410000
C	5.441862000	1.472294000	-0.331699000
C	3.646208000	0.093526000	0.374253000
H	6.490775000	1.654629000	-0.539559000
H	-3.190039000	2.853159000	-0.280294000
H	-2.129449000	5.048032000	-0.550941000
C	2.160236000	3.320104000	-0.445490000
C	4.961698000	3.829908000	-0.847144000

C	2.661413000	4.601148000	-0.742297000
C	0.450569000	5.579028000	-0.729636000
C	1.786522000	5.724197000	-0.866129000
C	4.059214000	4.831161000	-0.939541000
H	6.018294000	4.000344000	-1.021288000
H	4.377070000	5.839382000	-1.182576000
H	2.224605000	6.695623000	-1.068008000
H	-0.214072000	6.432654000	-0.813223000
N	2.759886000	1.054108000	0.211115000
C	0.056943000	1.894083000	-0.241450000
H	0.664934000	1.006709000	-0.169763000
H	-1.433753000	-0.114987000	1.144685000
C	-0.625288000	-0.551697000	2.059977000
H	-0.103832000	0.409939000	2.208406000
O	0.056963000	-1.567865000	1.663847000
H	-1.404688000	-0.732608000	2.815805000
C	5.013846000	0.240157000	0.085222000
H	5.705025000	-0.583747000	0.202190000
P	2.929944000	-1.458327000	1.024276000
H	1.428764000	-1.322308000	1.225006000
C	3.601055000	-1.741995000	2.735623000
C	5.128808000	-1.718319000	2.830585000
C	3.014386000	-0.598484000	3.581798000
C	3.051770000	-3.083491000	3.240667000
H	5.600539000	-2.463023000	2.187545000
H	5.534954000	-0.734422000	2.585622000
H	5.411232000	-1.941321000	3.864553000
H	1.921627000	-0.624987000	3.591339000
H	3.366227000	-0.711825000	4.611917000
H	3.336054000	0.383695000	3.223845000
H	3.272772000	-3.171292000	4.308921000
H	1.967448000	-3.151501000	3.114484000
H	3.519561000	-3.929341000	2.731948000
C	3.188613000	-2.818623000	-0.223936000
C	2.088975000	-3.866999000	0.021598000
C	2.970508000	-2.169426000	-1.600584000
C	4.568067000	-3.475420000	-0.159926000
H	2.241446000	-4.410083000	0.955585000
H	1.097099000	-3.411919000	0.054317000
H	2.108759000	-4.593865000	-0.795997000
H	3.807189000	-1.526286000	-1.883381000
H	2.877862000	-2.958287000	-2.352842000
H	2.057152000	-1.569011000	-1.630036000
H	4.636662000	-4.219189000	-0.960550000
H	5.375403000	-2.754041000	-0.305526000
H	4.725130000	-3.992062000	0.789246000

I1

	Coordinates (Angstroms)		
	X	Y	Z
C	4.355656000	4.598465000	-0.108069000
C	3.350837000	5.501517000	-0.086000000
C	1.982303000	5.088867000	-0.097838000
C	1.618133000	3.730773000	-0.160899000
C	2.705483000	2.760808000	-0.171819000
C	4.050180000	3.209562000	-0.135402000
H	1.325282000	7.145902000	0.024901000

H	5.394969000	4.908120000	-0.092088000
H	3.561639000	6.564839000	-0.050837000
C	0.990325000	6.115608000	-0.028262000
C	0.193009000	3.405218000	-0.188150000
C	-0.756320000	4.461829000	-0.098354000
C	-0.326750000	5.818379000	-0.015247000
H	-1.076257000	6.599588000	0.049570000
C	5.077102000	2.245310000	-0.111007000
C	3.411764000	0.546952000	-0.134224000
H	6.109294000	2.579312000	-0.087736000
C	-2.141967000	4.183073000	-0.103401000
C	-0.330568000	2.097730000	-0.324874000
C	-2.603251000	2.901602000	-0.203210000
H	-3.671860000	2.714958000	-0.204754000
C	-1.692806000	1.823295000	-0.337109000
H	-2.834527000	5.014861000	-0.027753000
H	0.374021000	1.289329000	-0.423096000
N	2.438818000	1.434124000	-0.194819000
B	-2.218847000	0.378196000	-0.480282000
C	4.773056000	0.912597000	-0.099493000
H	5.551124000	0.161615000	-0.063110000
C	-3.707503000	0.057064000	-0.047442000
C	-4.619496000	-0.490257000	-0.936812000
C	-4.166461000	0.292769000	1.240316000
C	-5.925564000	-0.780231000	-0.583920000
C	-5.459481000	-0.000667000	1.634963000
C	-6.343291000	-0.536814000	0.713575000
C	-1.362264000	-0.813995000	-1.048592000
C	-1.462944000	-2.092971000	-0.504363000
C	-0.496178000	-0.685004000	-2.132963000
C	-0.735371000	-3.169148000	-0.976023000
C	0.244846000	-1.741654000	-2.628074000
C	0.134306000	-2.986187000	-2.035178000
F	-3.338723000	0.797491000	2.163531000
F	-5.860868000	0.224452000	2.884625000
F	-7.590499000	-0.816503000	1.073919000
F	-6.777892000	-1.289583000	-1.471043000
F	-4.247751000	-0.740048000	-2.199258000
F	-0.367805000	0.482325000	-2.769483000
F	1.084437000	-1.566689000	-3.645609000
F	0.879670000	-3.995667000	-2.465253000
F	-0.838414000	-4.367256000	-0.404407000
F	-2.266648000	-2.325744000	0.537583000
P	2.882176000	-1.218759000	-0.270447000
C	4.354700000	-2.091495000	0.401474000
C	5.127432000	-2.852544000	-0.475188000
C	4.734937000	-2.003258000	1.744451000
C	6.269351000	-3.508379000	-0.021778000
H	4.837284000	-2.934066000	-1.517915000
C	5.870434000	-2.659293000	2.196742000
H	4.139001000	-1.416809000	2.436025000
C	6.640586000	-3.412382000	1.312968000
H	6.863342000	-4.098111000	-0.711169000
H	6.156901000	-2.583758000	3.239981000
H	7.527690000	-3.924932000	1.668616000
C	1.681288000	-1.454711000	1.111528000
C	1.346540000	-2.784128000	1.395201000

C	1.044028000	-0.439340000	1.822966000
C	0.407404000	-3.089075000	2.369910000
H	1.821326000	-3.592426000	0.846360000
C	0.091129000	-0.746412000	2.790873000
H	1.276572000	0.597278000	1.621810000
C	-0.229437000	-2.068206000	3.068376000
H	0.161548000	-4.125571000	2.570684000
H	-0.400555000	0.058418000	3.326195000
H	-0.977552000	-2.303624000	3.816849000

I1_CO

	Coordinates (Angstroms)		
	X	Y	Z
C	4.057593000	4.780787000	-0.379297000
C	3.011352000	5.626800000	-0.250609000
C	1.674724000	5.141779000	-0.090843000
C	1.390807000	3.764696000	-0.057019000
C	2.522176000	2.856419000	-0.193631000
C	3.833262000	3.375178000	-0.352439000
H	0.901527000	7.161251000	-0.001361000
H	5.069858000	5.150287000	-0.502768000
H	3.163963000	6.700524000	-0.268982000
C	0.630721000	6.111264000	0.027910000
C	-0.001072000	3.353247000	0.100290000
C	-1.008037000	4.352280000	0.198706000
C	-0.660028000	5.735226000	0.163922000
H	-1.450185000	6.474098000	0.246102000
C	4.899968000	2.464735000	-0.482056000
C	3.341684000	0.683432000	-0.270660000
H	5.905855000	2.852024000	-0.608696000
C	-2.363415000	3.979329000	0.331585000
C	-0.436569000	2.008155000	0.153704000
C	-2.740742000	2.661725000	0.360479000
H	-3.790760000	2.406841000	0.458985000
C	-1.766538000	1.649123000	0.253586000
H	-3.108838000	4.764645000	0.405551000
H	0.314711000	1.237934000	0.089419000
N	2.328638000	1.520573000	-0.171433000
B	-2.161845000	0.068299000	0.302933000
C	4.669944000	1.114510000	-0.439422000
H	5.480332000	0.402405000	-0.526185000
C	-3.749595000	-0.246730000	0.189388000
C	-4.391632000	-0.032880000	-1.023178000
C	-4.549024000	-0.737568000	1.207547000
C	-5.737377000	-0.285939000	-1.220336000
C	-5.898603000	-1.006201000	1.053565000
C	-6.496421000	-0.777660000	-0.171882000
C	-1.282414000	-0.940279000	-0.629036000
C	-1.085656000	-2.257226000	-0.236489000
C	-0.744905000	-0.604081000	-1.866369000
C	-0.386067000	-3.185982000	-0.982638000
C	-0.034476000	-1.507066000	-2.641090000
C	0.154013000	-2.800355000	-2.194978000
F	-4.018814000	-0.976664000	2.421068000
F	-6.619061000	-1.480429000	2.068522000
F	-7.790408000	-1.027816000	-0.342862000
F	-6.306695000	-0.061496000	-2.403127000

F	-3.701204000	0.448403000	-2.063790000
F	-0.886190000	0.620629000	-2.377222000
F	0.503702000	-1.125182000	-3.798613000
F	0.875276000	-3.657362000	-2.910194000
F	-0.195490000	-4.425434000	-0.528634000
F	-1.581766000	-2.673735000	0.944517000
P	2.801182000	-1.083945000	-0.226086000
C	4.405440000	-1.982735000	-0.195639000
C	4.825217000	-2.576559000	-1.387757000
C	5.221804000	-2.076280000	0.934726000
C	6.047611000	-3.237535000	-1.456402000
H	4.190220000	-2.524635000	-2.267053000
C	6.439115000	-2.741389000	0.867616000
H	4.905521000	-1.626499000	1.869848000
C	6.855628000	-3.319261000	-0.328743000
H	6.363597000	-3.693598000	-2.388054000
H	7.065613000	-2.808275000	1.750409000
H	7.807023000	-3.837369000	-0.378703000
C	2.243941000	-1.255693000	1.522008000
C	1.626382000	-2.463827000	1.859565000
C	2.409689000	-0.286762000	2.513552000
C	1.193099000	-2.703664000	3.157235000
H	1.491302000	-3.231469000	1.104595000
C	1.970137000	-0.525282000	3.811297000
H	2.885119000	0.659219000	2.283920000
C	1.363374000	-1.732471000	4.137453000
H	0.714375000	-3.646544000	3.396940000
H	2.104138000	0.238102000	4.569950000
H	1.018157000	-1.912721000	5.149451000
C	-1.662399000	-0.182989000	1.813813000
O	-1.226363000	-0.176798000	2.845214000

I2

	Coordinates (Angstroms)		
	X	Y	Z
C	-4.133402000	4.742879000	0.181485000
C	-3.083264000	5.587434000	0.292181000
C	-1.735937000	5.105829000	0.321468000
C	-1.445610000	3.733424000	0.226106000
C	-2.581630000	2.824695000	0.153458000
C	-3.903442000	3.339229000	0.115377000
H	-0.961794000	7.116407000	0.537437000
H	-5.153028000	5.111717000	0.151878000
H	-3.241768000	6.658311000	0.361355000
C	-0.688230000	6.069524000	0.459974000
C	-0.044738000	3.321858000	0.223288000
C	0.962519000	4.311426000	0.393566000
C	0.607756000	5.689050000	0.511210000
H	1.398112000	6.422356000	0.634069000
C	-4.971884000	2.425579000	0.025985000
C	-3.392129000	0.650413000	0.040103000
H	-5.986784000	2.808663000	-0.008039000
C	2.320916000	3.931375000	0.440296000
C	0.397551000	1.988980000	0.059501000
C	2.697769000	2.617540000	0.312648000
H	3.750590000	2.357318000	0.366777000
C	1.728296000	1.615597000	0.111143000

H	3.067683000	4.706829000	0.580201000
H	-0.350139000	1.225858000	-0.078013000
N	-2.381860000	1.489903000	0.139126000
B	2.074946000	0.057657000	-0.059719000
C	-4.731251000	1.076899000	-0.022439000
H	-5.540903000	0.363070000	-0.102469000
P	-2.841927000	-1.114656000	0.066275000
C	3.659652000	-0.266175000	-0.218919000
C	4.361454000	0.108472000	-1.357057000
C	4.428509000	-0.860576000	0.772188000
C	5.717037000	-0.100749000	-1.532161000
C	5.789687000	-1.088169000	0.640725000
C	6.437333000	-0.711577000	-0.520939000
C	1.271187000	-0.951232000	0.934198000
C	1.051646000	-2.272275000	0.576225000
C	0.724924000	-0.582673000	2.156315000
C	0.295861000	-3.162808000	1.313443000
C	-0.042441000	-1.440743000	2.928659000
C	-0.269169000	-2.734427000	2.500170000
F	3.879523000	-1.232415000	1.936917000
F	6.479518000	-1.665385000	1.624500000
F	7.742147000	-0.925074000	-0.661751000
F	6.331005000	0.280191000	-2.652053000
F	3.718006000	0.731011000	-2.366925000
F	1.590693000	-2.746868000	-0.572649000
F	0.086366000	-4.408162000	0.882580000
F	0.932257000	0.635957000	2.667546000
F	-1.030613000	-3.557042000	3.216147000
F	-0.574385000	-1.024417000	4.078151000
O	1.358446000	-0.288746000	-1.504846000
H	1.449793000	-1.186754000	-1.858224000
H	1.535099000	0.353025000	-2.206433000
C	-1.963484000	-1.261256000	-1.547916000
C	-1.878873000	-0.254965000	-2.511031000
C	-1.329385000	-2.482163000	-1.794249000
C	-1.170886000	-0.464288000	-3.689907000
H	-2.358924000	0.702297000	-2.348882000
C	-0.632093000	-2.696344000	-2.976832000
H	-1.377474000	-3.275660000	-1.055082000
C	-0.545605000	-1.683605000	-3.927425000
H	-1.114303000	0.328524000	-4.427876000
H	-0.149514000	-3.651870000	-3.149529000
H	0.000704000	-1.846239000	-4.850054000
C	-4.408628000	-2.013933000	-0.279600000
C	-5.059032000	-2.602370000	0.807117000
C	-4.983075000	-2.114284000	-1.549638000
C	-6.270697000	-3.263340000	0.632845000
H	-4.614696000	-2.542501000	1.795883000
C	-6.188772000	-2.781161000	-1.724689000
H	-4.488557000	-1.667762000	-2.405696000
C	-6.835950000	-3.354315000	-0.633310000
H	-6.766337000	-3.714150000	1.485490000
H	-6.625768000	-2.854715000	-2.714550000
H	-7.777963000	-3.873337000	-0.772393000

I3

Coordinates (Angstroms)

	X	Y	Z
C	-4.802029000	4.288985000	-0.018363000
C	-3.863519000	5.182007000	-0.413207000
C	-2.489807000	4.814217000	-0.589985000
C	-2.058119000	3.501692000	-0.334882000
C	-3.083549000	2.531831000	-0.002481000
C	-4.434304000	2.926982000	0.183686000
H	-1.932098000	6.803687000	-1.236809000
H	-5.835156000	4.584016000	0.128112000
H	-4.137224000	6.215457000	-0.598757000
C	-1.563155000	5.802884000	-1.039346000
C	-0.642786000	3.188953000	-0.443746000
C	0.235247000	4.186173000	-0.942098000
C	-0.258545000	5.490960000	-1.237666000
H	0.437115000	6.237850000	-1.607172000
C	-5.375477000	1.943620000	0.549808000
C	-3.649182000	0.339693000	0.437684000
H	-6.407098000	2.237239000	0.711239000
C	1.604745000	3.873978000	-1.123734000
C	-0.065387000	1.952017000	-0.062614000
C	2.097128000	2.634035000	-0.808693000
H	3.149664000	2.421622000	-0.977416000
C	1.263238000	1.642020000	-0.238228000
H	2.260284000	4.643530000	-1.520825000
H	-0.692367000	1.196313000	0.367678000
N	-2.752296000	1.232525000	0.101893000
B	1.758341000	0.131647000	0.103957000
C	-4.995180000	0.631204000	0.700682000
H	-5.696123000	-0.138638000	0.998093000
P	-2.900559000	-1.317143000	0.500506000
C	2.331821000	-0.459991000	-1.351117000
C	1.451508000	-0.935949000	-2.315112000
C	3.660463000	-0.455120000	-1.751985000
C	1.846876000	-1.427731000	-3.548686000
C	4.102783000	-0.927686000	-2.977676000
C	3.187637000	-1.428306000	-3.883946000
C	2.925386000	0.027791000	1.287501000
C	3.520409000	-1.199404000	1.552438000
C	3.311443000	1.042339000	2.149795000
C	4.449153000	-1.420172000	2.551887000
C	4.240374000	0.870826000	3.168232000
C	4.816558000	-0.367137000	3.370651000
F	4.621222000	0.040295000	-0.944962000
F	5.401803000	-0.902192000	-3.294085000
F	3.590469000	-1.892075000	-5.068533000
F	0.943799000	-1.892492000	-4.420197000
F	0.122522000	-0.927047000	-2.103504000
F	3.192714000	-2.276894000	0.803675000
F	4.987026000	-2.629308000	2.742073000
F	2.795209000	2.279745000	2.053475000
F	5.706080000	-0.549657000	4.347866000
F	4.575684000	1.892611000	3.963164000
O	0.598765000	-0.662785000	0.589333000
H	0.885266000	-1.551985000	0.801133000
H	-1.507645000	-1.076761000	0.448049000
C	-3.334654000	-2.311317000	-0.927086000
C	-4.674408000	-2.493703000	-1.278660000

C	-2.313110000	-2.904517000	-1.668661000
C	-4.986440000	-3.277532000	-2.378794000
H	-5.465107000	-2.026579000	-0.701229000
C	-2.638292000	-3.684696000	-2.771303000
H	-1.275439000	-2.745017000	-1.402212000
C	-3.969568000	-3.871737000	-3.122400000
H	-6.023091000	-3.423426000	-2.658320000
H	-1.847468000	-4.137919000	-3.357125000
H	-4.219418000	-4.479340000	-3.984820000
C	-3.360570000	-2.156614000	2.025987000
C	-3.303004000	-1.441485000	3.225603000
C	-3.724691000	-3.503002000	2.017348000
C	-3.614845000	-2.081179000	4.415966000
H	-3.018201000	-0.394805000	3.232006000
C	-4.035506000	-4.132638000	3.216020000
H	-3.767457000	-4.057327000	1.086800000
C	-3.980585000	-3.424140000	4.410376000
H	-3.571344000	-1.531519000	5.348678000
H	-4.319041000	-5.178433000	3.214231000
H	-4.222513000	-3.920263000	5.343445000

I4

	Coordinates (Angstroms)		
	X	Y	Z
C	4.783453000	4.252874000	0.198995000
C	3.815377000	5.143240000	0.502327000
C	2.436986000	4.761277000	0.543962000
C	2.020305000	3.448041000	0.246123000
C	3.072139000	2.486218000	-0.073495000
C	4.430823000	2.903190000	-0.084120000
H	1.872082000	6.764472000	1.131326000
H	5.829276000	4.539137000	0.171710000
H	4.062925000	6.174877000	0.728376000
C	1.497548000	5.769381000	0.916863000
C	0.590337000	3.152522000	0.305777000
C	-0.300295000	4.180259000	0.719472000
C	0.180145000	5.487813000	1.016348000
H	-0.532326000	6.248445000	1.318140000
C	5.425324000	1.952125000	-0.374823000
C	3.718390000	0.321977000	-0.617936000
H	6.464468000	2.264919000	-0.379212000
C	-1.682232000	3.911847000	0.844650000
C	0.009665000	1.895797000	-0.016498000
C	-2.188636000	2.675427000	0.560988000
H	-3.252308000	2.490720000	0.682026000
C	-1.340437000	1.642374000	0.098386000
H	-2.335011000	4.712413000	1.178086000
H	0.674138000	1.113813000	-0.339361000
N	2.768334000	1.199229000	-0.355816000
B	-2.010095000	0.213660000	-0.132991000
C	5.084158000	0.652634000	-0.639854000
H	5.837041000	-0.093739000	-0.856216000
P	3.064333000	-1.353635000	-1.041409000
C	-2.482934000	-0.398145000	1.296863000
C	-1.552969000	-0.651977000	2.297817000
C	-3.809679000	-0.549228000	1.679269000
C	-1.886909000	-1.063804000	3.574566000

C	-4.188760000	-0.955191000	2.948951000
C	-3.222017000	-1.221486000	3.900716000
C	-3.023048000	-0.024458000	-1.367604000
C	-3.552897000	-1.289414000	-1.602578000
C	-3.400723000	0.948519000	-2.283406000
C	-4.419304000	-1.581370000	-2.637719000
C	-4.275813000	0.698099000	-3.330552000
C	-4.788855000	-0.571630000	-3.509100000
F	-4.808683000	-0.279563000	0.826945000
F	-5.477732000	-1.085795000	3.261668000
F	-3.572177000	-1.616417000	5.120376000
F	-0.945973000	-1.302871000	4.487019000
F	-0.246728000	-0.499566000	2.033516000
F	-3.213346000	-2.314120000	-0.800178000
F	-4.895482000	-2.815074000	-2.806808000
F	-2.928118000	2.195302000	-2.210312000
F	-5.621726000	-0.825252000	-4.513039000
F	-4.613421000	1.671770000	-4.176393000
O	-0.691783000	-0.884027000	-0.597226000
H	-0.416041000	-1.581499000	0.023434000
H	0.208678000	-2.105962000	-1.986090000
C	-0.238759000	-1.112628000	-1.889496000
O	-0.375643000	-0.296799000	-2.731790000
C	2.432263000	-1.948389000	0.580209000
C	2.772134000	-1.391960000	1.814399000
C	1.576507000	-3.055444000	0.550940000
C	2.272781000	-1.936776000	2.991178000
H	3.428419000	-0.530371000	1.862022000
C	1.076791000	-3.599120000	1.730195000
H	1.317903000	-3.512359000	-0.401081000
C	1.426489000	-3.038964000	2.953057000
H	2.537117000	-1.489842000	3.942844000
H	0.417700000	-4.459457000	1.691435000
H	1.033240000	-3.454806000	3.873274000
C	4.602230000	-2.340508000	-1.246830000
C	5.268518000	-2.976657000	-0.197141000
C	5.129505000	-2.430608000	-2.538233000
C	6.444360000	-3.678527000	-0.434641000
H	4.871832000	-2.924325000	0.810624000
C	6.311906000	-3.122967000	-2.772715000
H	4.615493000	-1.950417000	-3.365454000
C	6.970486000	-3.749350000	-1.720384000
H	6.951753000	-4.169374000	0.388638000
H	6.714133000	-3.180167000	-3.778121000
H	7.890427000	-4.294064000	-1.902176000

I5

	Coordinates (Angstroms)		
	X	Y	Z
B	2.214461000	-0.176507000	-0.004577000
C	3.089142000	0.750435000	1.005083000
C	4.420553000	1.031311000	0.735437000
C	2.550092000	1.415847000	2.094069000
C	5.180292000	1.898136000	1.501016000
C	3.274842000	2.286421000	2.889384000
C	4.602303000	2.529825000	2.588249000
C	2.881946000	-1.591807000	-0.412226000

C	2.668335000	-2.212902000	-1.636656000
C	3.690930000	-2.297517000	0.471725000
C	3.258342000	-3.415815000	-1.989737000
C	4.295204000	-3.501806000	0.158315000
C	4.082374000	-4.062394000	-1.087983000
F	5.031629000	0.453139000	-0.311105000
F	6.457660000	2.130251000	1.201015000
F	5.317271000	3.365841000	3.335883000
F	2.707064000	2.891985000	3.931769000
F	1.258485000	1.223766000	2.421788000
F	3.913324000	-1.828719000	1.708254000
F	5.069490000	-4.127680000	1.044703000
F	4.652096000	-5.219256000	-1.408954000
F	3.025727000	-3.960795000	-3.184048000
F	1.846621000	-1.675378000	-2.547105000
C	1.927473000	2.214780000	-3.023413000
C	0.643084000	2.783605000	-2.864047000
C	-0.227779000	2.283936000	-1.861556000
C	1.561995000	0.771891000	-1.119370000
C	2.375690000	1.236059000	-2.179912000
C	-2.596109000	2.380300000	-0.855964000
C	-3.744254000	3.172692000	-0.602004000
C	-4.711737000	2.676869000	0.292625000
C	-3.459581000	0.661698000	0.432939000
H	-5.578195000	3.287696000	0.524640000
H	3.375148000	0.833384000	-2.309074000
H	2.567496000	2.595160000	-3.813237000
C	-1.540089000	2.892988000	-1.712556000
C	-3.924583000	4.414933000	-1.275969000
C	-1.820854000	4.065229000	-2.436614000
C	0.257478000	3.899556000	-3.664375000
C	-0.909354000	4.542569000	-3.427410000
C	-3.016472000	4.814203000	-2.195355000
H	-4.812866000	5.001169000	-1.067021000
H	-3.164851000	5.731163000	-2.755701000
H	-1.181054000	5.428387000	-3.991563000
H	0.940607000	4.250100000	-4.431057000
N	-2.507870000	1.128472000	-0.351180000
C	0.288078000	1.286115000	-0.992718000
H	-0.344092000	0.944373000	-0.187326000
O	-0.233150000	-1.959059000	-0.291485000
H	-1.054917000	-1.804021000	0.205789000
H	0.935705000	-0.874027000	0.441902000
H	1.252670000	-0.388195000	1.009895000
H	-0.416068000	-1.616995000	-1.174094000
C	-4.570191000	1.426629000	0.834340000
H	-5.307669000	1.019504000	1.514594000
P	-3.223543000	-1.122530000	0.847830000
C	-4.105863000	-1.921362000	-0.552402000
C	-5.222428000	-1.364825000	-1.182450000
C	-3.616273000	-3.150373000	-1.000277000
C	-5.838431000	-2.028929000	-2.234821000
H	-5.616238000	-0.409440000	-0.853780000
C	-4.237843000	-3.817674000	-2.050808000
H	-2.739346000	-3.586730000	-0.532834000
C	-5.348367000	-3.257004000	-2.669360000
H	-6.702526000	-1.586195000	-2.717669000

H	-3.846382000	-4.770107000	-2.390276000
H	-5.829112000	-3.771718000	-3.493910000
C	-4.349361000	-1.350437000	2.278936000
C	-3.921747000	-0.824461000	3.503034000
C	-5.571463000	-2.019891000	2.217575000
C	-4.714846000	-0.940434000	4.636457000
H	-2.965096000	-0.314517000	3.569098000
C	-6.359012000	-2.145901000	3.358170000
H	-5.917280000	-2.444016000	1.281874000
C	-5.936738000	-1.603002000	4.565489000
H	-4.375292000	-0.519908000	5.576449000
H	-7.307761000	-2.667725000	3.298523000
H	-6.554704000	-1.699007000	5.451368000

I6

	Coordinates (Angstroms)		
	X	Y	Z
B	-2.139765000	-0.072265000	0.551186000
C	-1.321256000	-1.270910000	-0.209039000
C	-1.360080000	-1.455582000	-1.585085000
C	-0.527491000	-2.184595000	0.466489000
C	-0.698838000	-2.474576000	-2.245655000
C	0.183534000	-3.199757000	-0.156092000
C	0.083011000	-3.358408000	-1.523325000
C	-3.744792000	-0.369240000	0.725829000
C	-4.472997000	0.454199000	1.580254000
C	-4.499413000	-1.362676000	0.118677000
C	-5.829546000	0.323914000	1.817903000
C	-5.861506000	-1.532355000	0.327434000
C	-6.534239000	-0.683373000	1.182936000
F	-2.105162000	-0.638878000	-2.349498000
F	-0.790576000	-2.607403000	-3.572788000
F	0.734854000	-4.344954000	-2.139820000
F	0.973744000	-4.016214000	0.552072000
F	-0.399939000	-2.130598000	1.809691000
F	-3.940870000	-2.244147000	-0.731530000
F	-6.527725000	-2.511644000	-0.293377000
F	-7.843264000	-0.830293000	1.394721000
F	-6.465352000	1.154612000	2.650599000
F	-3.854157000	1.454985000	2.232525000
C	-2.566887000	3.369756000	-1.335351000
C	-1.303035000	3.970121000	-1.145501000
C	-0.269235000	3.234278000	-0.504473000
C	-1.859141000	1.375071000	-0.146864000
C	-2.831552000	2.109784000	-0.862350000
C	2.228232000	3.194506000	0.116688000
C	3.430710000	3.915616000	0.350871000
C	4.561589000	3.211453000	0.804005000
C	3.304632000	1.225598000	0.653332000
H	5.476209000	3.758758000	1.004972000
H	-3.818000000	1.682420000	-1.017725000
H	-3.336809000	3.939972000	-1.846870000
C	1.031310000	3.871371000	-0.345336000
C	3.480618000	5.314158000	0.083604000
C	1.170760000	5.228058000	-0.685624000
C	-1.085754000	5.321270000	-1.546517000
C	0.091499000	5.939660000	-1.292071000

C	2.396856000	5.928424000	-0.443817000
H	4.400226000	5.855175000	0.276180000
H	2.433765000	6.983828000	-0.692336000
H	0.244213000	6.980189000	-1.557193000
H	-1.897617000	5.854019000	-2.031301000
N	2.220521000	1.856794000	0.267283000
C	-0.609314000	1.952188000	-0.004229000
H	0.138486000	1.404267000	0.545222000
H	-1.704303000	-0.001352000	1.689328000
O	0.679607000	0.350155000	2.912548000
H	0.624132000	1.302578000	2.792357000
H	-0.175703000	0.026759000	2.601077000
C	4.517539000	1.846991000	0.967244000
H	5.377200000	1.283869000	1.306478000
P	2.987251000	-0.566653000	0.639399000
H	1.788186000	-0.694537000	1.351686000
C	2.794415000	-1.109142000	-1.072322000
C	2.015016000	-0.348769000	-1.948867000
C	3.408044000	-2.284548000	-1.508694000
C	1.872721000	-0.759875000	-3.265697000
H	1.519323000	0.550804000	-1.605624000
C	3.259933000	-2.681486000	-2.831033000
H	3.992806000	-2.892958000	-0.829741000
C	2.498151000	-1.919885000	-3.708571000
H	1.258049000	-0.176528000	-3.940766000
H	3.732882000	-3.595077000	-3.170366000
H	2.376878000	-2.239657000	-4.736947000
C	4.218534000	-1.532880000	1.506833000
C	3.826618000	-2.226606000	2.653411000
C	5.538208000	-1.597312000	1.049106000
C	4.763658000	-2.984105000	3.344140000
H	2.801142000	-2.178007000	3.002327000
C	6.464567000	-2.355088000	1.748973000
H	5.835985000	-1.066541000	0.151225000
C	6.076815000	-3.046742000	2.893443000
H	4.464629000	-3.525365000	4.233754000
H	7.488429000	-2.410426000	1.398897000
H	6.804142000	-3.640089000	3.435781000

I7

	Coordinates (Angstroms)		
	X	Y	Z
B	-2.191653000	0.155444000	0.591871000
C	-1.282617000	-0.889030000	-0.304664000
C	-0.750537000	-0.651170000	-1.568096000
C	-0.987311000	-2.148988000	0.204865000
C	0.021308000	-1.571928000	-2.263308000
C	-0.210937000	-3.094403000	-0.447473000
C	0.296591000	-2.804027000	-1.700348000
C	-3.777604000	-0.221733000	0.429819000
C	-4.603574000	-0.475940000	1.515938000
C	-4.408414000	-0.299006000	-0.804258000
C	-5.948414000	-0.794341000	1.398159000
C	-5.746881000	-0.612029000	-0.971356000
C	-6.524600000	-0.865911000	0.143984000
F	-0.961976000	0.511451000	-2.202176000
F	0.509277000	-1.276927000	-3.472983000

F	1.046154000	-3.693125000	-2.351106000
F	0.067199000	-4.269915000	0.126856000
F	-1.444979000	-2.513677000	1.417505000
F	-3.714279000	-0.047345000	-1.929082000
F	-6.294913000	-0.669711000	-2.189782000
F	-7.816877000	-1.170479000	0.010596000
F	-6.694648000	-1.031720000	2.482637000
F	-4.126577000	-0.418419000	2.771715000
C	-2.516758000	4.032686000	-0.056444000
C	-1.168722000	4.447724000	-0.035796000
C	-0.139845000	3.480009000	0.144947000
C	-1.873553000	1.722640000	0.305801000
C	-2.858653000	2.710635000	0.095717000
C	2.410511000	3.063764000	0.195037000
C	3.729302000	3.596197000	0.152491000
C	4.821721000	2.708012000	0.207358000
C	3.281890000	0.931123000	0.332319000
H	5.827492000	3.112084000	0.169428000
H	-3.907887000	2.430187000	0.062919000
H	-3.284450000	4.787698000	-0.199222000
C	1.249041000	3.933569000	0.116374000
C	3.924341000	5.001022000	0.034435000
C	1.508227000	5.308433000	-0.029609000
C	-0.847044000	5.828468000	-0.188098000
C	0.439806000	6.245896000	-0.171229000
C	2.847420000	5.814433000	-0.056630000
H	4.934054000	5.395004000	0.008205000
H	2.979796000	6.886016000	-0.161963000
H	0.686214000	7.296435000	-0.279917000
H	-1.657384000	6.539807000	-0.312920000
N	2.243366000	1.730107000	0.284917000
C	-0.553928000	2.142459000	0.340585000
H	0.204397000	1.397853000	0.507633000
H	-1.883077000	-0.053230000	1.750072000
C	4.616702000	1.353104000	0.293838000
H	5.435602000	0.644745000	0.320273000
P	2.764122000	-0.807902000	0.426629000
H	1.373615000	-0.727955000	0.292006000
C	3.076772000	-1.580534000	2.010900000
C	4.348893000	-1.538853000	2.586543000
C	2.023600000	-2.242233000	2.643745000
C	4.559840000	-2.162007000	3.806905000
H	5.163770000	-1.023009000	2.090416000
C	2.249396000	-2.865333000	3.864177000
H	1.038753000	-2.274963000	2.191929000
C	3.512156000	-2.822927000	4.442891000
H	5.541380000	-2.130072000	4.264431000
H	1.434990000	-3.378216000	4.361418000
H	3.682564000	-3.306655000	5.397926000
C	3.468551000	-1.757090000	-0.925363000
C	3.474107000	-1.187224000	-2.201312000
C	3.940640000	-3.052806000	-0.718064000
C	3.955185000	-1.924606000	-3.271951000
H	3.096639000	-0.182655000	-2.360550000
C	4.428747000	-3.777124000	-1.797984000
H	3.925672000	-3.496598000	0.270701000
C	4.435063000	-3.215082000	-3.068954000

H	3.952365000	-1.491481000	-4.264776000
H	4.798975000	-4.783741000	-1.644849000
H	4.811447000	-3.787001000	-3.909335000

I8

	Coordinates (Angstroms)		
	X	Y	Z
B	2.156721000	-0.199503000	0.074221000
C	3.252885000	0.654732000	0.953841000
C	4.615397000	0.670403000	0.688752000
C	2.852192000	1.541216000	1.942799000
C	5.518975000	1.468230000	1.370945000
C	3.716036000	2.354871000	2.654031000
C	5.067769000	2.314119000	2.366950000
C	2.700568000	-1.618027000	-0.504450000
C	2.369930000	-2.114992000	-1.759166000
C	3.502939000	-2.454815000	0.263943000
C	2.834958000	-3.331345000	-2.237012000
C	3.984341000	-3.674219000	-0.174361000
C	3.649121000	-4.114289000	-1.442332000
F	5.133907000	-0.102277000	-0.281335000
F	6.818477000	1.429161000	1.074166000
F	5.921763000	3.086949000	3.033782000
F	3.259126000	3.177946000	3.598201000
F	1.539209000	1.669633000	2.247094000
F	3.833985000	-2.103466000	1.517576000
F	4.754211000	-4.430093000	0.610593000
F	4.098661000	-5.284370000	-1.886615000
F	2.494014000	-3.756922000	-3.454634000
F	1.565706000	-1.439961000	-2.584736000
C	2.052718000	2.405002000	-2.774648000
C	0.766290000	2.982119000	-2.664108000
C	-0.173535000	2.435746000	-1.752096000
C	1.547398000	0.846106000	-0.991832000
C	2.435127000	1.377717000	-1.959978000
C	-2.595179000	2.551490000	-0.866490000
C	-3.744851000	3.354684000	-0.650879000
C	-4.771746000	2.848047000	0.168605000
C	-3.558087000	0.812222000	0.321383000
H	-5.640012000	3.467355000	0.368926000
H	3.440260000	0.975133000	-2.045760000
H	2.745642000	2.821473000	-3.499135000
C	-1.480535000	3.069391000	-1.643754000
C	-3.865434000	4.624354000	-1.284840000
C	-1.697248000	4.281701000	-2.325258000
C	0.451102000	4.146265000	-3.423725000
C	-0.721031000	4.793888000	-3.231714000
C	-2.893189000	5.042645000	-2.125900000
H	-4.756032000	5.217647000	-1.108747000
H	-2.989980000	5.984530000	-2.655274000
H	-0.944905000	5.711247000	-3.765668000
H	1.188069000	4.524901000	-4.124472000
O	1.030426000	-0.632788000	1.149453000
N	-2.556165000	1.285801000	-0.392737000
C	0.270272000	1.360836000	-0.931259000
H	-0.441102000	0.955427000	-0.229573000
C	0.276295000	-1.902864000	1.143623000

H	-0.375820000	-1.827895000	2.017049000
H	1.032210000	-2.669167000	1.284825000
O	-0.383330000	-2.105825000	-0.022597000
H	-1.264180000	-1.692989000	0.051726000
H	0.494149000	0.103436000	1.474445000
C	-4.680786000	1.580019000	0.679050000
H	-5.460461000	1.162607000	1.304240000
P	-3.358541000	-0.974233000	0.726623000
C	-4.365891000	-1.801414000	-0.565086000
C	-5.407880000	-1.187932000	-1.263480000
C	-4.056455000	-3.136431000	-0.838622000
C	-6.131054000	-1.901958000	-2.211256000
H	-5.658852000	-0.150449000	-1.075112000
C	-4.788630000	-3.852434000	-1.778362000
H	-3.234225000	-3.618081000	-0.318349000
C	-5.827017000	-3.235043000	-2.466185000
H	-6.935908000	-1.414904000	-2.750589000
H	-4.541813000	-4.889016000	-1.978486000
H	-6.394499000	-3.789741000	-3.205238000
C	-4.371696000	-1.152877000	2.246755000
C	-3.841617000	-0.622576000	3.427676000
C	-5.609120000	-1.794994000	2.286133000
C	-4.545236000	-0.714999000	4.620711000
H	-2.874456000	-0.128172000	3.412983000
C	-6.306860000	-1.897656000	3.485879000
H	-6.034888000	-2.217495000	1.383125000
C	-5.780171000	-1.356230000	4.652061000
H	-4.126321000	-0.292924000	5.527388000
H	-7.267239000	-2.400846000	3.505536000
H	-6.326671000	-1.436433000	5.585142000

TSI2-3

	Coordinates (Angstroms)		
	X	Y	Z
C	5.195903000	3.763027000	-0.455686000
C	4.464639000	4.757509000	0.114382000
C	3.079748000	4.590687000	0.450024000
C	2.430680000	3.394219000	0.128819000
C	3.243404000	2.302851000	-0.346931000
C	4.605011000	2.481279000	-0.674876000
H	2.869315000	6.531191000	1.399867000
H	6.238553000	3.916884000	-0.711558000
H	4.928770000	5.714398000	0.330511000
C	2.359110000	5.605215000	1.156142000
C	1.020303000	3.241273000	0.396530000
C	0.383247000	4.189638000	1.226623000
C	1.084139000	5.386653000	1.576221000
H	0.570999000	6.131837000	2.176167000
C	5.305616000	1.356633000	-1.168431000
C	3.346314000	0.052775000	-0.855695000
H	6.348294000	1.468045000	-1.447374000
C	-0.928986000	3.908298000	1.683835000
C	0.248713000	2.162449000	-0.094607000
C	-1.563086000	2.740067000	1.335481000
H	-2.539419000	2.522542000	1.760462000
C	-0.992149000	1.843683000	0.394863000
H	-1.414068000	4.621573000	2.343452000

H	0.676160000	1.527195000	-0.845264000
N	2.684462000	1.085456000	-0.402544000
B	-1.636080000	0.412339000	-0.002712000
C	4.682523000	0.133639000	-1.289271000
H	5.202262000	-0.733485000	-1.678192000
P	2.217677000	-1.388366000	-0.798697000
C	-1.933086000	-0.401499000	1.403568000
C	-0.858195000	-0.838391000	2.170912000
C	-3.177609000	-0.676511000	1.950113000
C	-0.989186000	-1.544204000	3.353728000
C	-3.357140000	-1.372473000	3.136662000
C	-2.254883000	-1.818897000	3.839885000
C	-2.948107000	0.458697000	-1.002514000
C	-3.488442000	-0.726703000	-1.484841000
C	-3.581386000	1.601330000	-1.469288000
C	-4.566380000	-0.809060000	-2.343381000
C	-4.671115000	1.571161000	-2.330017000
C	-5.168311000	0.361222000	-2.771344000
F	-4.305916000	-0.260648000	1.346657000
F	-4.584477000	-1.615103000	3.605092000
F	-2.407973000	-2.495115000	4.977906000
F	0.086589000	-1.951836000	4.031844000
F	0.400366000	-0.568481000	1.786923000
F	-2.943153000	-1.906310000	-1.100628000
F	-5.028557000	-1.989402000	-2.760575000
F	-3.176047000	2.826479000	-1.106799000
F	-6.208428000	0.319230000	-3.601740000
F	-5.240109000	2.707177000	-2.740392000
O	-0.581440000	-0.413890000	-0.809969000
H	-0.999509000	-1.212699000	-1.147024000
H	0.678478000	-0.782236000	-0.709141000
C	2.521020000	-2.388856000	-2.279467000
C	1.801074000	-2.066990000	-3.432648000
C	3.477086000	-3.405579000	-2.313560000
C	2.041678000	-2.756170000	-4.614259000
H	1.053300000	-1.280743000	-3.408242000
C	3.710580000	-4.092325000	-3.498194000
H	4.033413000	-3.664255000	-1.419130000
C	2.995911000	-3.767069000	-4.646741000
H	1.482327000	-2.504794000	-5.507997000
H	4.452808000	-4.881872000	-3.524240000
H	3.181964000	-4.304356000	-5.569933000
C	2.661892000	-2.383772000	0.650354000
C	1.952937000	-3.565631000	0.881427000
C	3.609746000	-1.947056000	1.573164000
C	2.204272000	-4.309178000	2.025391000
H	1.205809000	-3.903674000	0.170513000
C	3.854260000	-2.696095000	2.718566000
H	4.150565000	-1.021392000	1.412638000
C	3.154486000	-3.874304000	2.944950000
H	1.654144000	-5.226043000	2.203076000
H	4.589829000	-2.352334000	3.436542000
H	3.343485000	-4.452226000	3.842484000

TSI3-4

Coordinates (Angstroms)

	X	Y	Z
C	4.845253000	4.192313000	-0.399275000
C	3.879569000	5.136931000	-0.358652000
C	2.489068000	4.794114000	-0.331744000
C	2.063993000	3.452250000	-0.353527000
C	3.109674000	2.438078000	-0.395850000
C	4.479962000	2.815301000	-0.414665000
H	1.916977000	6.879528000	-0.261442000
H	5.896607000	4.457706000	-0.417600000
H	4.141286000	6.189682000	-0.342997000
C	1.542712000	5.861491000	-0.275851000
C	0.631692000	3.178304000	-0.322921000
C	-0.272276000	4.271519000	-0.253447000
C	0.213330000	5.610554000	-0.234512000
H	-0.503925000	6.423674000	-0.185414000
C	5.458640000	1.803899000	-0.449383000
C	3.720946000	0.202707000	-0.434552000
H	6.505542000	2.088358000	-0.464972000
C	-1.664816000	4.024728000	-0.189170000
C	0.060385000	1.880415000	-0.346459000
C	-2.162153000	2.749518000	-0.204098000
H	-3.235582000	2.593605000	-0.140679000
C	-1.294854000	1.636213000	-0.302020000
H	-2.334586000	4.876976000	-0.122740000
H	0.729924000	1.042518000	-0.363804000
N	2.786482000	1.128744000	-0.416012000
B	-1.832080000	0.109825000	-0.206376000
C	5.094567000	0.480011000	-0.457650000
H	5.829291000	-0.315195000	-0.478698000
P	2.977712000	-1.469353000	-0.431737000
C	-2.437952000	-0.051381000	1.325372000
C	-1.583655000	-0.145900000	2.419534000
C	-3.785750000	0.003316000	1.653962000
C	-2.020812000	-0.236118000	3.730762000
C	-4.266087000	-0.078150000	2.951760000
C	-3.376000000	-0.207909000	4.000722000
C	-2.913252000	-0.376858000	-1.360269000
C	-3.429003000	-1.664923000	-1.285017000
C	-3.295268000	0.325809000	-2.492056000
C	-4.290190000	-2.215851000	-2.214710000
C	-4.157846000	-0.184874000	-3.453028000
C	-4.662359000	-1.462358000	-3.314035000
F	-4.722952000	0.160004000	0.700261000
F	-5.578293000	-0.028433000	3.197974000
F	-3.817235000	-0.290300000	5.255907000
F	-1.146150000	-0.338491000	4.736430000
F	-0.253745000	-0.131427000	2.251963000
F	-3.091816000	-2.460038000	-0.246354000
F	-4.758611000	-3.458867000	-2.070080000
F	-2.829333000	1.559621000	-2.740143000
F	-5.487749000	-1.967884000	-4.230284000
F	-4.493548000	0.545482000	-4.520589000
O	-0.632931000	-0.816191000	-0.361421000
H	-0.755657000	-1.676876000	0.049383000
H	1.529321000	-1.337786000	-1.421087000
C	0.385629000	-0.977686000	-2.036803000
O	-0.175553000	-0.574395000	-2.936347000

C	2.507869000	-1.811431000	1.287457000
C	2.947179000	-1.039794000	2.361953000
C	1.628885000	-2.875416000	1.508044000
C	2.509443000	-1.333729000	3.647824000
H	3.619768000	-0.204683000	2.201626000
C	1.200068000	-3.167485000	2.795536000
H	1.278960000	-3.476344000	0.674067000
C	1.636820000	-2.393135000	3.865434000
H	2.843596000	-0.724825000	4.479888000
H	0.514388000	-3.990547000	2.961620000
H	1.288752000	-2.609970000	4.868769000
C	4.288337000	-2.642258000	-0.882103000
C	5.020596000	-3.346169000	0.074946000
C	4.567750000	-2.816345000	-2.240177000
C	6.028669000	-4.213803000	-0.327665000
H	4.805900000	-3.219539000	1.130251000
C	5.579635000	-3.681144000	-2.635669000
H	3.996817000	-2.275692000	-2.988618000
C	6.308674000	-4.380789000	-1.679488000
H	6.595440000	-4.760907000	0.417086000
H	5.792907000	-3.814685000	-3.690009000
H	7.093937000	-5.061143000	-1.989288000

TSI5-6

	Coordinates (Angstroms)		
	X	Y	Z
B	-2.174902000	0.198890000	-0.001739000
C	-3.029478000	-0.717035000	1.037824000
C	-4.352422000	-1.050742000	0.789058000
C	-2.466934000	-1.316408000	2.153322000
C	-5.083675000	-1.907488000	1.592420000
C	-3.162363000	-2.177684000	2.985683000
C	-4.482042000	-2.476380000	2.701250000
C	-2.883095000	1.586952000	-0.459108000
C	-2.642332000	2.195199000	-1.684771000
C	-3.744821000	2.287722000	0.377746000
C	-3.247128000	3.375890000	-2.084997000
C	-4.368411000	3.469461000	0.018077000
C	-4.122769000	4.015542000	-1.228540000
F	-4.988136000	-0.529543000	-0.274313000
F	-6.355832000	-2.190403000	1.309767000
F	-5.167556000	-3.304478000	3.486484000
F	-2.572783000	-2.722076000	4.051069000
F	-1.182238000	-1.073266000	2.474324000
F	-4.006313000	1.837797000	1.614085000
F	-5.193847000	4.089255000	0.862385000
F	-4.710459000	5.151610000	-1.591812000
F	-2.980311000	3.905534000	-3.279540000
F	-1.765126000	1.666106000	-2.552582000
C	-1.976010000	-2.235566000	-3.017831000
C	-0.692648000	-2.812152000	-2.876650000
C	0.196472000	-2.309518000	-1.892355000
C	-1.572216000	-0.779491000	-1.128322000
C	-2.402370000	-1.244553000	-2.176181000
C	2.583559000	-2.392651000	-0.937651000
C	3.741190000	-3.176890000	-0.702551000
C	4.721204000	-2.672938000	0.174286000

C	3.456613000	-0.667445000	0.334332000
H	5.595487000	-3.277538000	0.392606000
H	-3.399080000	-0.832628000	-2.296502000
H	-2.631727000	-2.617138000	-3.794289000
C	1.512267000	-2.914891000	-1.767964000
C	3.913617000	-4.421142000	-1.375147000
C	1.782005000	-4.089689000	-2.491437000
C	-0.322373000	-3.934118000	-3.676147000
C	0.849931000	-4.575122000	-3.458978000
C	2.987265000	-4.830154000	-2.272258000
H	4.809255000	-5.001822000	-1.182944000
H	3.128798000	-5.749481000	-2.830539000
H	1.110651000	-5.464287000	-4.023148000
H	-1.019966000	-4.290133000	-4.427200000
N	2.497388000	-1.138981000	-0.437086000
C	-0.302194000	-1.307794000	-1.018957000
H	0.342792000	-0.970347000	-0.222594000
O	0.229267000	1.810695000	-0.264420000
H	1.087623000	1.653269000	0.191039000
H	-0.753841000	0.979851000	0.308470000
H	-1.225100000	0.492635000	0.881190000
H	0.368686000	1.541139000	-1.182510000
C	4.578474000	-1.424035000	0.719379000
H	5.320737000	-1.010947000	1.391128000
P	3.204805000	1.105777000	0.773350000
C	4.183467000	1.970292000	-0.515630000
C	5.260809000	1.396330000	-1.194194000
C	3.815951000	3.285793000	-0.810739000
C	5.961122000	2.129264000	-2.144226000
H	5.558227000	0.374801000	-0.986505000
C	4.525243000	4.021509000	-1.753352000
H	2.968240000	3.736439000	-0.303689000
C	5.598467000	3.443307000	-2.421359000
H	6.794295000	1.672816000	-2.666959000
H	4.234325000	5.043307000	-1.969783000
H	6.148504000	4.013777000	-3.161586000
C	4.208586000	1.294128000	2.296097000
C	3.682149000	0.750157000	3.472365000
C	5.436295000	1.954315000	2.340561000
C	4.382409000	0.846872000	4.667097000
H	2.721366000	0.244027000	3.452657000
C	6.130054000	2.060283000	3.542147000
H	5.857354000	2.387500000	1.440402000
C	5.608126000	1.505101000	4.704137000
H	3.967036000	0.415285000	5.570878000
H	7.083064000	2.577079000	3.566940000
H	6.151309000	1.588371000	5.638916000

TSI7

	Coordinates (Angstroms)		
	X	Y	Z
B	-2.132358000	0.147957000	0.295572000
C	-1.573705000	-1.072543000	-0.626159000
C	-1.176802000	-0.975715000	-1.952394000
C	-1.528533000	-2.356007000	-0.095816000
C	-0.771812000	-2.066901000	-2.705575000
C	-1.127183000	-3.470920000	-0.810235000

C	-0.759312000	-3.324655000	-2.134548000
C	-3.718140000	-0.024300000	0.631796000
C	-4.236717000	0.304764000	1.874200000
C	-4.658505000	-0.448502000	-0.295479000
C	-5.577094000	0.201508000	2.203424000
C	-6.010358000	-0.567925000	-0.014069000
C	-6.472969000	-0.244743000	1.248566000
F	-1.215348000	0.199139000	-2.599182000
F	-0.411019000	-1.918995000	-3.983734000
F	-0.393590000	-4.387493000	-2.851563000
F	-1.092569000	-4.680859000	-0.242579000
F	-1.922219000	-2.569390000	1.173931000
F	-4.285749000	-0.745590000	-1.552025000
F	-6.870269000	-0.982937000	-0.947318000
F	-7.768785000	-0.352406000	1.540265000
F	-6.013850000	0.527954000	3.422689000
F	-3.420866000	0.769847000	2.845799000
C	-2.357656000	3.900249000	-0.801855000
C	-0.999485000	4.272861000	-0.883557000
C	0.015438000	3.321736000	-0.579760000
C	-1.751606000	1.647785000	-0.152501000
C	-2.724544000	2.630149000	-0.437281000
C	2.560438000	2.917201000	-0.319762000
C	3.877541000	3.439428000	-0.422219000
C	4.959760000	2.609480000	-0.072962000
C	3.405721000	0.890839000	0.424070000
H	5.967839000	3.003364000	-0.151050000
H	-3.779898000	2.380355000	-0.381036000
H	-3.110580000	4.646218000	-1.037906000
C	1.411960000	3.748826000	-0.652336000
C	4.092113000	4.777247000	-0.858673000
C	1.687809000	5.069027000	-1.055098000
C	-0.660243000	5.601156000	-1.275871000
C	0.633307000	5.981008000	-1.367481000
C	3.029460000	5.555708000	-1.160131000
H	5.107277000	5.150864000	-0.936774000
H	3.172585000	6.579803000	-1.488057000
H	0.897766000	6.988352000	-1.670780000
H	-1.461163000	6.296973000	-1.503807000
N	2.378144000	1.648345000	0.105245000
C	-0.418947000	2.018815000	-0.230002000
H	0.333584000	1.277704000	-0.021238000
H	-1.550533000	-0.044498000	1.384949000
O	0.034416000	1.074197000	2.704951000
C	-0.478536000	-0.115261000	2.763537000
H	-1.396619000	-0.300836000	3.311231000
O	0.272703000	-1.112386000	2.531723000
H	-0.640917000	1.752959000	2.863647000
C	4.739239000	1.327188000	0.357499000
H	5.556863000	0.672940000	0.631498000
P	2.892486000	-0.796821000	0.945720000
H	1.145563000	-0.885220000	2.044022000
C	2.764201000	-1.714804000	-0.632169000
C	2.503381000	-3.086494000	-0.551539000
C	2.903394000	-1.117747000	-1.883822000
C	2.425249000	-3.853597000	-1.704706000
H	2.371450000	-3.560450000	0.416584000

C	2.803177000	-1.887681000	-3.038636000
H	3.088575000	-0.053629000	-1.971028000
C	2.575924000	-3.254685000	-2.951784000
H	2.227883000	-4.916661000	-1.629256000
H	2.904999000	-1.412856000	-4.007724000
H	2.498877000	-3.850490000	-3.853883000
C	4.394316000	-1.482216000	1.733511000
C	4.537548000	-1.287451000	3.109166000
C	5.401423000	-2.141966000	1.025811000
C	5.677871000	-1.733472000	3.766622000
H	3.754638000	-0.784184000	3.668386000
C	6.536594000	-2.593770000	1.687186000
H	5.300591000	-2.303979000	-0.041638000
C	6.677704000	-2.387973000	3.055890000
H	5.782414000	-1.575510000	4.834106000
H	7.314048000	-3.106548000	1.131870000
H	7.565155000	-2.742310000	3.568405000

TSI8

	Coordinates (Angstroms)		
	X	Y	Z
B	1.875102000	-0.011477000	-0.202028000
C	2.639531000	-0.004521000	1.278963000
C	4.000711000	0.149901000	1.492619000
C	1.885847000	-0.060753000	2.442296000
C	4.581858000	0.201786000	2.750239000
C	2.416657000	-0.019294000	3.719267000
C	3.784372000	0.109004000	3.875608000
C	2.859396000	-0.548533000	-1.408371000
C	3.010082000	0.046608000	-2.651443000
C	3.529288000	-1.758645000	-1.262038000
C	3.788150000	-0.492850000	-3.667907000
C	4.309140000	-2.334191000	-2.248232000
C	4.442871000	-1.691098000	-3.466265000
F	4.850479000	0.276594000	0.454525000
F	5.903579000	0.343310000	2.886711000
F	4.325017000	0.152219000	5.093813000
F	1.626311000	-0.093600000	4.794701000
F	0.538588000	-0.133841000	2.370953000
F	3.439322000	-2.446635000	-0.110237000
F	4.930990000	-3.499173000	-2.041747000
F	5.190069000	-2.225011000	-4.432211000
F	3.899216000	0.132220000	-4.843870000
F	2.387220000	1.195282000	-2.956873000
C	1.778412000	3.885461000	-0.409456000
C	0.395491000	4.174257000	-0.486412000
C	-0.551721000	3.113515000	-0.495768000
C	1.321902000	1.508957000	-0.376323000
C	2.226396000	2.597028000	-0.340261000
C	-3.071590000	2.501082000	-0.497412000
C	-4.421740000	2.951826000	-0.535219000
C	-5.457702000	2.001982000	-0.489477000
C	-3.815321000	0.315103000	-0.374658000
H	-6.485811000	2.346942000	-0.518458000
H	3.293614000	2.406332000	-0.265984000
H	2.477031000	4.716772000	-0.399573000
C	-1.971270000	3.456944000	-0.535475000

C	-4.715790000	4.342796000	-0.615936000
C	-2.331230000	4.818274000	-0.598437000
C	-0.025498000	5.532547000	-0.549049000
C	-1.340175000	5.843386000	-0.611347000
C	-3.701355000	5.233081000	-0.644394000
H	-5.751650000	4.661673000	-0.650339000
H	-3.904846000	6.297119000	-0.702226000
H	-1.667216000	6.876115000	-0.664025000
H	0.730501000	6.311112000	-0.547484000
O	0.781707000	-1.043408000	-0.178807000
N	-2.823257000	1.177962000	-0.420601000
C	-0.026192000	1.790581000	-0.458951000
H	-0.724413000	0.972546000	-0.492345000
C	-0.072593000	-2.084974000	-1.717935000
H	-0.272607000	-2.862676000	-0.974591000
H	0.887243000	-2.127658000	-2.233088000
O	-0.999850000	-1.424637000	-2.212172000
H	0.115396000	-0.854736000	0.487561000
C	-5.170047000	0.662468000	-0.407389000
H	-5.947225000	-0.090268000	-0.369167000
P	-3.198210000	-1.398845000	-0.279030000
H	-2.059638000	-1.499427000	-1.393248000
C	-2.540548000	-1.625564000	1.395350000
C	-1.767908000	-2.761764000	1.651375000
C	-2.784615000	-0.704374000	2.413981000
C	-1.247627000	-2.973445000	2.920357000
H	-1.573803000	-3.481399000	0.862876000
C	-2.257192000	-0.922169000	3.680597000
H	-3.372568000	0.185935000	2.223686000
C	-1.489842000	-2.052200000	3.933765000
H	-0.644470000	-3.852680000	3.114239000
H	-2.435762000	-0.197624000	4.466303000
H	-1.066740000	-2.209064000	4.919160000
C	-4.590680000	-2.528980000	-0.510736000
C	-5.284225000	-3.077829000	0.568384000
C	-4.980278000	-2.822063000	-1.820032000
C	-6.366566000	-3.916669000	0.333868000
H	-4.979998000	-2.857346000	1.585656000
C	-6.066961000	-3.656413000	-2.045674000
H	-4.438199000	-2.399809000	-2.659959000
C	-6.757701000	-4.204090000	-0.969565000
H	-6.902737000	-4.347854000	1.171307000
H	-6.368689000	-3.883322000	-3.061595000
H	-7.601377000	-4.861280000	-1.147990000

TSI9

	Coordinates (Angstroms)		
	X	Y	Z
B	-2.132752000	0.173595000	0.240999000
C	-1.526888000	-1.040421000	-0.641114000
C	-1.022211000	-0.930935000	-1.930125000
C	-1.534436000	-2.330835000	-0.122492000
C	-0.568476000	-2.017277000	-2.662691000
C	-1.075677000	-3.438021000	-0.814420000
C	-0.602393000	-3.280222000	-2.103755000
C	-3.697665000	-0.017010000	0.625134000
C	-4.197241000	0.436162000	1.837609000

C	-4.640191000	-0.571872000	-0.227229000
C	-5.526838000	0.329512000	2.206428000
C	-5.982176000	-0.694915000	0.097942000
C	-6.427675000	-0.244848000	1.326974000
F	-1.000621000	0.249544000	-2.567186000
F	-0.126787000	-1.859136000	-3.913991000
F	-0.194862000	-4.338943000	-2.803538000
F	-1.104578000	-4.655453000	-0.263902000
F	-2.057319000	-2.561103000	1.092096000
F	-4.283794000	-1.004407000	-1.448742000
F	-6.847532000	-1.237262000	-0.762400000
F	-7.713325000	-0.355966000	1.659398000
F	-5.949827000	0.775729000	3.391841000
F	-3.373635000	1.025628000	2.723455000
C	-2.367055000	3.924369000	-0.837058000
C	-1.013066000	4.320208000	-0.842957000
C	0.001601000	3.379027000	-0.509464000
C	-1.756785000	1.678137000	-0.178084000
C	-2.731310000	2.643815000	-0.506945000
C	2.540287000	2.994380000	-0.186116000
C	3.855494000	3.535191000	-0.211489000
C	4.938469000	2.700926000	0.122662000
C	3.396105000	0.945633000	0.455458000
H	5.941758000	3.112891000	0.106199000
H	-3.783598000	2.375839000	-0.505777000
H	-3.119347000	4.662544000	-1.097703000
C	1.392623000	3.826474000	-0.514818000
C	4.065180000	4.898514000	-0.562850000
C	1.665365000	5.164914000	-0.852406000
C	-0.676860000	5.663997000	-1.180732000
C	0.612235000	6.070286000	-1.186250000
C	3.002770000	5.674584000	-0.871076000
H	5.075776000	5.291133000	-0.575593000
H	3.143277000	6.716507000	-1.137888000
H	0.872996000	7.091935000	-1.440456000
H	-1.477380000	6.352285000	-1.431687000
N	2.364015000	1.699590000	0.150990000
C	-0.428938000	2.068795000	-0.189825000
H	0.320084000	1.333664000	0.048554000
H	-1.516127000	-0.016204000	1.365960000
C	-0.536896000	-0.216947000	2.434477000
H	-0.220884000	0.835371000	2.374536000
O	0.274546000	-1.140550000	2.164042000
H	-1.339199000	-0.421099000	3.154400000
C	4.725164000	1.389672000	0.464530000
H	5.540014000	0.728177000	0.730150000
P	2.909247000	-0.770969000	0.830507000
H	1.545921000	-0.812604000	1.498631000
C	2.881701000	-1.697743000	-0.721946000
C	2.678546000	-3.078865000	-0.653780000
C	3.033914000	-1.069206000	-1.956383000
C	2.655157000	-3.827831000	-1.820369000
H	2.544334000	-3.568925000	0.304962000
C	2.998094000	-1.827751000	-3.120621000
H	3.176876000	0.003231000	-2.019747000
C	2.815967000	-3.202946000	-3.053147000
H	2.496422000	-4.898095000	-1.766479000

H	3.109400000	-1.338971000	-4.081099000
H	2.784651000	-3.789004000	-3.964249000
C	4.155632000	-1.501373000	1.914507000
C	5.288537000	-2.140465000	1.406854000
C	3.981008000	-1.367005000	3.293370000
C	6.244539000	-2.638700000	2.281902000
H	5.422402000	-2.254347000	0.336862000
C	4.942888000	-1.867464000	4.161206000
H	3.095328000	-0.881218000	3.689145000
C	6.072794000	-2.500759000	3.655672000
H	7.124068000	-3.135929000	1.889702000
H	4.805768000	-1.765398000	5.231281000
H	6.820804000	-2.892744000	4.335630000

J1(B(C_6F_5)$_3$)

	Coordinates (Angstroms)		
	X	Y	Z
B	0.002446000	-0.002949000	0.002781000
C	1.542451000	-0.285786000	0.001187000
C	2.106690000	-1.300693000	0.770109000
C	2.426820000	0.464051000	-0.770015000
C	3.465080000	-1.556164000	0.786660000
C	3.787538000	0.221910000	-0.791315000
C	4.307077000	-0.791593000	-0.003572000
C	-1.015617000	-1.192980000	0.005205000
C	-2.176318000	-1.168141000	0.774444000
C	-0.814743000	-2.335540000	-0.765445000
C	-3.082683000	-2.211753000	0.790737000
C	-1.710670000	-3.388141000	-0.786451000
C	-2.848503000	-3.324654000	0.000496000
C	-0.521403000	1.473294000	0.000526000
C	0.064055000	2.463263000	0.785075000
C	-1.600053000	1.870101000	-0.785281000
C	-0.392972000	3.767708000	0.803485000
C	-2.069216000	3.170117000	-0.805159000
C	-1.463269000	4.121221000	-0.001333000
F	1.336204000	-2.058427000	1.554708000
F	3.968575000	-2.523020000	1.548435000
F	5.609822000	-1.030106000	-0.006316000
F	4.598212000	0.947903000	-1.555570000
F	1.972468000	1.444914000	-1.553943000
F	-2.441155000	-0.121513000	1.560564000
F	-4.171709000	-2.157253000	1.552122000
F	-3.712717000	-4.328167000	-0.002281000
F	-1.492476000	-4.454856000	-1.550294000
F	0.261907000	-2.438873000	-1.548975000
F	-2.209054000	0.991362000	-1.585863000
F	-3.090899000	3.514698000	-1.583722000
F	-1.907221000	5.369109000	-0.002437000
F	0.181402000	4.680799000	1.581129000
F	1.092436000	2.167590000	1.584663000

J1

	Coordinates (Angstroms)		
	X	Y	Z
C	-1.177170000	1.821915000	0.000196000

C	0.058767000	2.506080000	0.000290000
C	1.203864000	1.783558000	0.000134000
C	0.002891000	-0.311468000	0.000155000
O	0.030184000	-1.542626000	0.000294000
H	0.107017000	3.586428000	0.000430000
H	2.161957000	2.275352000	0.000176000
C	-1.240386000	0.460279000	0.000097000
N	1.208306000	0.419119000	-0.000068000
H	-2.086610000	2.409856000	0.000244000
C	2.519076000	-0.340574000	-0.000133000
C	2.605272000	-1.193061000	-1.272463000
H	3.577545000	-1.691982000	-1.302269000
H	1.822522000	-1.947082000	-1.302858000
H	2.522818000	-0.556008000	-2.158131000
C	2.605674000	-1.192311000	1.272662000
H	1.823137000	-1.946530000	1.303802000
H	3.578033000	-1.691068000	1.302542000
H	2.523333000	-0.554738000	2.157963000
C	3.715670000	0.615884000	-0.000614000
H	3.748686000	1.246424000	-0.892768000
H	3.748798000	1.247193000	0.890991000
H	4.619445000	0.003438000	-0.000407000
C	-2.569518000	-0.305002000	-0.000048000
C	-3.769417000	0.651363000	-0.000010000
H	-3.784911000	1.291425000	-0.887722000
H	-4.692837000	0.065197000	-0.000191000
H	-3.785079000	1.291146000	0.887902000
C	-2.672167000	-1.183312000	-1.261051000
H	-1.871967000	-1.922077000	-1.293114000
H	-3.632642000	-1.709151000	-1.269601000
H	-2.617434000	-0.568287000	-2.165335000
C	-2.672344000	-1.183627000	1.260719000
H	-1.872115000	-1.922359000	1.292727000
H	-2.617787000	-0.568829000	2.165169000
H	-3.632801000	-1.709503000	1.268972000

J1_CO

	Coordinates (Angstroms)		
	X	Y	Z
B	0.016678000	-0.009050000	0.618236000
C	1.592157000	0.115637000	0.232810000
C	2.545413000	-0.659133000	0.878312000
C	2.074088000	0.938702000	-0.775708000
C	3.892227000	-0.631979000	0.572387000
C	3.416854000	0.995297000	-1.113156000
C	4.330685000	0.209638000	-0.434869000
C	-0.680186000	-1.431291000	0.241872000
C	-1.851275000	-1.823058000	0.874677000
C	-0.218766000	-2.288867000	-0.747109000
C	-2.537579000	-2.982412000	0.570842000
C	-0.877128000	-3.461863000	-1.081128000
C	-2.039937000	-3.811684000	-0.418833000
C	-0.880486000	1.295746000	0.242726000
C	-0.695910000	2.499699000	0.906208000
C	-1.819100000	1.314330000	-0.779663000
C	-1.388767000	3.656332000	0.605154000
C	-2.535170000	2.452372000	-1.113144000

C	-2.321704000	3.628034000	-0.416415000
F	2.151007000	-1.500345000	1.852883000
F	4.759606000	-1.398821000	1.227903000
F	5.618735000	0.258304000	-0.751397000
F	3.833000000	1.797221000	-2.090716000
F	1.248910000	1.703664000	-1.497507000
F	-2.369624000	-1.033202000	1.833936000
F	-3.657198000	-3.304681000	1.212994000
F	-2.676934000	-4.932640000	-0.733926000
F	-0.402079000	-4.250696000	-2.042353000
F	0.877524000	-2.000376000	-1.455563000
F	-2.053069000	0.226555000	-1.521211000
F	-3.422722000	2.425170000	-2.104808000
F	-3.003690000	4.723146000	-0.728409000
F	-1.170001000	4.781654000	1.280224000
F	0.214231000	2.565393000	1.896397000
C	0.017191000	-0.021866000	2.254331000
O	0.013061000	-0.044953000	3.370391000

J1-2

	Coordinates (Angstroms)		
	X	Y	Z
C	-4.023894000	0.642538000	-1.241145000
C	-4.191488000	2.006368000	-1.042379000
C	-3.119212000	2.808675000	-1.292074000
C	-1.657853000	0.942277000	-1.398892000
H	-5.152458000	2.441831000	-0.808878000
H	-3.216820000	3.879902000	-1.316134000
C	-2.787255000	0.077339000	-1.504146000
H	-4.900263000	0.006736000	-1.220190000
N	-1.889027000	2.303322000	-1.534261000
O	-0.431060000	0.572632000	-1.208327000
B	0.263002000	-0.142037000	-0.006559000
C	1.355599000	-1.232469000	-0.574840000
C	2.068945000	-1.994133000	0.345148000
C	1.792275000	-1.366538000	-1.885757000
C	3.078886000	-2.874950000	0.009161000
C	2.799283000	-2.241284000	-2.268430000
C	3.444669000	-3.006410000	-1.318138000
C	1.251164000	0.937383000	0.789592000
C	1.287845000	1.176170000	2.159117000
C	2.254195000	1.595218000	0.081489000
C	2.191870000	2.035674000	2.766942000
C	3.170735000	2.463559000	0.643891000
C	3.137576000	2.692379000	2.006311000
C	-0.919088000	-0.738772000	0.967774000
C	-1.811677000	0.152759000	1.553398000
C	-1.146628000	-2.064965000	1.306691000
C	-2.840338000	-0.208333000	2.399438000
C	-2.147002000	-2.474428000	2.177113000
C	-3.004700000	-1.542512000	2.725010000
F	1.792827000	-1.883306000	1.656619000
F	3.705715000	-3.587914000	0.947292000
F	4.414455000	-3.848197000	-1.671183000
F	3.159764000	-2.335118000	-3.551020000
F	1.276061000	-0.631644000	-2.882938000
F	-0.426570000	-3.062695000	0.770312000

F	-2.317945000	-3.770069000	2.444545000
F	-3.986416000	-1.921997000	3.537815000
F	-3.660185000	0.711262000	2.910662000
F	-1.683518000	1.471952000	1.315913000
F	2.361366000	1.432309000	-1.246510000
F	4.073818000	3.086271000	-0.116253000
F	4.006455000	3.523982000	2.574887000
F	2.159205000	2.223509000	4.087906000
F	0.444455000	0.566726000	3.006193000
C	-0.798321000	3.290528000	-1.967256000
C	-0.061859000	3.765263000	-0.719834000
C	0.101494000	2.630981000	-3.017070000
C	-1.443034000	4.501677000	-2.658659000
H	-0.722505000	4.379558000	-0.102177000
H	0.281915000	2.932921000	-0.116437000
H	0.800724000	4.369814000	-1.010944000
H	-0.499307000	2.317290000	-3.876212000
H	0.817729000	3.379345000	-3.361665000
H	0.650540000	1.778912000	-2.640159000
H	-0.632397000	5.077247000	-3.108660000
H	-2.124925000	4.199987000	-3.457683000
H	-1.959952000	5.173893000	-1.972222000
C	-2.799770000	-1.368085000	-2.052946000
C	-1.428153000	-1.995479000	-2.279267000
C	-3.633605000	-2.305941000	-1.166040000
C	-3.477302000	-1.265244000	-3.438775000
H	-0.804951000	-1.371011000	-2.915656000
H	-0.899725000	-2.190219000	-1.350329000
H	-1.566164000	-2.957686000	-2.780467000
H	-4.688173000	-2.024149000	-1.124214000
H	-3.586185000	-3.319240000	-1.574551000
H	-3.248223000	-2.337555000	-0.147461000
H	-3.542126000	-2.259992000	-3.889617000
H	-4.489828000	-0.858791000	-3.369764000
H	-2.895781000	-0.624032000	-4.108079000

J2

	Coordinates (Angstroms)		
	X	Y	Z
C	4.867143000	1.109760000	-0.035142000
C	5.314481000	-0.094318000	0.532824000
C	4.750567000	-1.257804000	0.124823000
C	3.156212000	-0.103728000	-1.225737000
O	2.100566000	-0.159914000	-1.907501000
H	6.112232000	-0.113475000	1.262101000
H	5.096746000	-2.204008000	0.502438000
C	3.826344000	1.138426000	-0.924666000
N	3.724249000	-1.287577000	-0.771732000
H	5.369884000	2.026164000	0.244628000
C	3.441277000	-2.552359000	-2.878760000
H	3.184361000	-3.524570000	-3.305795000
H	2.798160000	-1.798023000	-3.326864000
H	4.482820000	-2.332958000	-3.130156000
C	4.143441000	-3.764911000	-0.837534000
H	4.026959000	-3.928362000	0.236952000
H	3.811976000	-4.674826000	-1.340743000
H	5.201252000	-3.625262000	-1.072838000

C	1.821264000	-2.932698000	-0.963013000
H	1.575472000	-3.935844000	-1.320100000
H	1.710279000	-2.930022000	0.122769000
H	1.109961000	-2.238764000	-1.397626000
C	3.439041000	2.259174000	-3.138661000
H	2.773466000	1.466519000	-3.481003000
H	3.144348000	3.191551000	-3.630281000
H	4.459183000	2.019116000	-3.454608000
C	1.939210000	2.818951000	-1.196044000
H	1.863817000	2.950221000	-0.113652000
H	1.659851000	3.763065000	-1.674525000
H	1.221721000	2.063246000	-1.503174000
C	4.283442000	3.611175000	-1.235769000
H	5.326243000	3.427340000	-1.511413000
H	3.950133000	4.502658000	-1.773748000
H	4.241401000	3.838062000	-0.166289000
C	3.266812000	-2.621149000	-1.356829000
C	3.370120000	2.435715000	-1.610064000
B	-1.173261000	-0.007957000	-0.060108000
C	-0.356377000	-0.917511000	1.023276000
C	0.904092000	-0.517158000	1.451958000
C	-0.811429000	-2.096435000	1.598979000
C	1.674508000	-1.215669000	2.361751000
C	-0.067280000	-2.835406000	2.507876000
C	1.184815000	-2.396048000	2.890765000
C	-1.255289000	1.570607000	0.372121000
C	-1.335564000	2.577963000	-0.575986000
C	-1.254385000	2.016116000	1.686535000
C	-1.347259000	3.928087000	-0.277265000
C	-1.273493000	3.355413000	2.036495000
C	-1.311506000	4.321217000	1.047097000
C	-2.617212000	-0.608080000	-0.529176000
C	-2.687783000	-1.761399000	-1.306263000
C	-3.845018000	-0.041253000	-0.216936000
C	-3.872817000	-2.301627000	-1.769448000
C	-5.055520000	-0.554214000	-0.659719000
C	-5.070355000	-1.688043000	-1.446728000
F	1.433939000	0.624458000	0.983571000
F	2.875181000	-0.769237000	2.729831000
F	1.909564000	-3.094806000	3.759193000
F	-0.556083000	-3.965467000	3.019099000
F	-2.023512000	-2.592525000	1.318870000
F	-1.402964000	2.266756000	-1.893021000
F	-1.383133000	4.842664000	-1.246201000
F	-1.322239000	5.612209000	1.366977000
F	-1.261431000	3.723024000	3.318216000
F	-1.252531000	1.141230000	2.704052000
F	-3.932528000	1.047636000	0.561288000
F	-6.203717000	0.038557000	-0.328292000
F	-6.222062000	-2.193170000	-1.878970000
F	-3.874515000	-3.413129000	-2.507102000
F	-1.572940000	-2.443245000	-1.609199000
O	-0.303711000	-0.083440000	-1.378634000
H	0.724588000	-0.102715000	-1.451491000
H	-0.651316000	0.439185000	-2.112207000

J3

	Coordinates (Angstroms)		
	X	Y	Z
C	4.768903000	1.150470000	-0.028347000
C	5.197913000	-0.036528000	0.553932000
C	4.648946000	-1.214467000	0.140595000
C	3.158767000	-0.080140000	-1.284268000
O	2.141433000	-0.166540000	-2.108756000
H	5.970006000	-0.049157000	1.310429000
H	4.985712000	-2.153734000	0.540730000
C	3.754948000	1.160852000	-0.968910000
N	3.663629000	-1.251979000	-0.787467000
H	5.242539000	2.074374000	0.272722000
C	3.489040000	-2.601445000	-2.862242000
H	3.287047000	-3.600189000	-3.255053000
H	2.861436000	-1.890798000	-3.394507000
H	4.540291000	-2.368116000	-3.052398000
C	4.032637000	-3.743873000	-0.732404000
H	3.864698000	-3.844009000	0.342814000
H	3.692045000	-4.669671000	-1.198462000
H	5.102555000	-3.650498000	-0.932570000
C	1.741199000	-2.879408000	-1.029827000
H	1.482451000	-3.859037000	-1.438434000
H	1.591010000	-2.921653000	0.050050000
H	1.057293000	-2.151614000	-1.453766000
C	3.418759000	2.317220000	-3.179438000
H	2.764784000	1.537880000	-3.569032000
H	3.144116000	3.263238000	-3.655111000
H	4.449523000	2.083786000	-3.463870000
C	1.846786000	2.805908000	-1.265874000
H	1.746141000	2.923772000	-0.183961000
H	1.563086000	3.750946000	-1.739144000
H	1.140637000	2.045831000	-1.590831000
C	4.176660000	3.644196000	-1.225535000
H	5.230414000	3.484162000	-1.472870000
H	3.842698000	4.537576000	-1.758950000
H	4.095667000	3.852745000	-0.154834000
C	3.210770000	-2.612876000	-1.355199000
C	3.295259000	2.461076000	-1.650997000
B	-1.098807000	-0.001535000	-0.094250000
C	-0.325059000	-0.912242000	1.039286000
C	0.947940000	-0.543638000	1.456092000
C	-0.802595000	-2.073684000	1.628259000
C	1.708968000	-1.256828000	2.362821000
C	-0.069621000	-2.829889000	2.533531000
C	1.198004000	-2.424391000	2.900217000
C	-1.243514000	1.574165000	0.393084000
C	-1.376145000	2.583076000	-0.548645000
C	-1.211980000	2.019602000	1.705826000
C	-1.409398000	3.932102000	-0.244681000
C	-1.248232000	3.358117000	2.061989000
C	-1.339709000	4.325155000	1.078515000
C	-2.560886000	-0.619657000	-0.543156000
C	-2.624893000	-1.756143000	-1.344087000
C	-3.796323000	-0.084558000	-0.207744000
C	-3.805772000	-2.307483000	-1.806929000
C	-5.003990000	-0.604954000	-0.650652000
C	-5.010307000	-1.721535000	-1.461719000

F	1.506996000	0.585181000	0.981948000
F	2.926271000	-0.839642000	2.721115000
F	1.915983000	-3.138925000	3.764879000
F	-0.583037000	-3.946173000	3.054890000
F	-2.030700000	-2.544671000	1.361557000
F	-1.483785000	2.278007000	-1.861811000
F	-1.498201000	4.850685000	-1.209900000
F	-1.368984000	5.617373000	1.402245000
F	-1.202105000	3.723574000	3.345959000
F	-1.158058000	1.146572000	2.727420000
F	-3.898430000	0.984145000	0.600449000
F	-6.159463000	-0.036801000	-0.295626000
F	-6.159673000	-2.236114000	-1.895263000
F	-3.796390000	-3.404687000	-2.568979000
F	-1.505045000	-2.419996000	-1.676081000
O	-0.205067000	-0.034071000	-1.308958000
H	1.211616000	-0.094143000	-1.665388000
H	-0.658934000	0.338788000	-2.068504000

J4

	Coordinates (Angstroms)		
	X	Y	Z
C	4.812798000	1.110950000	0.881010000
C	4.693049000	0.135053000	1.892180000
C	4.137732000	-1.060123000	1.583324000
C	3.741719000	-0.397271000	-0.699783000
O	3.289967000	-0.671130000	-1.816992000
H	5.032135000	0.322793000	2.901162000
H	4.035889000	-1.826571000	2.331901000
C	4.367791000	0.884399000	-0.388854000
N	3.669932000	-1.347618000	0.334382000
H	5.274851000	2.055458000	1.137245000
C	4.160061000	-3.398750000	-0.949865000
H	3.822819000	-4.413523000	-1.177249000
H	4.230396000	-2.841225000	-1.882878000
H	5.151655000	-3.462192000	-0.492838000
C	3.088973000	-3.599164000	1.285893000
H	2.401853000	-3.177793000	2.024895000
H	2.698126000	-4.575277000	0.993113000
H	4.063489000	-3.767781000	1.749502000
C	1.757947000	-2.688219000	-0.582442000
H	1.336447000	-3.696656000	-0.592637000
H	1.107785000	-2.063120000	0.032334000
H	1.769745000	-2.308753000	-1.598558000
C	5.533387000	1.309829000	-2.562060000
H	5.128062000	0.396122000	-2.999203000
H	5.700777000	2.032301000	-3.367614000
H	6.501431000	1.081472000	-2.105250000
C	3.237145000	2.263090000	-2.206277000
H	2.517092000	2.656307000	-1.484609000
H	3.411935000	3.043978000	-2.953046000
H	2.805880000	1.400732000	-2.709578000
C	5.176700000	3.208331000	-0.991629000
H	6.165282000	3.055830000	-0.548685000
H	5.294439000	3.908872000	-1.822897000
H	4.532006000	3.684228000	-0.246209000
H	1.135863000	-0.068860000	-1.757830000

C	3.168299000	-2.744759000	0.017382000
C	4.566724000	1.902917000	-1.519809000
B	-1.400367000	0.148789000	-0.352430000
C	-0.155280000	0.715438000	0.514585000
C	0.328080000	1.997203000	0.261985000
C	0.420934000	0.082094000	1.608384000
C	1.300402000	2.614866000	1.023541000
C	1.381335000	0.676893000	2.411630000
C	1.828371000	1.949191000	2.115408000
C	-2.724863000	1.099310000	-0.305647000
C	-3.682280000	1.040181000	-1.308131000
C	-3.039297000	1.950091000	0.747573000
C	-4.855322000	1.769587000	-1.311846000
C	-4.205271000	2.697752000	0.789805000
C	-5.117470000	2.611284000	-0.245663000
C	-1.812573000	-1.408223000	-0.145786000
C	-1.549444000	-2.463287000	-1.006422000
C	-2.518214000	-1.754619000	1.001510000
C	-1.930288000	-3.770665000	-0.747779000
C	-2.923605000	-3.042171000	1.294186000
C	-2.625105000	-4.063439000	0.408673000
F	-0.165215000	2.703174000	-0.767157000
F	1.733610000	3.836544000	0.718400000
F	2.758440000	2.527693000	2.863567000
F	1.871651000	0.025670000	3.464062000
F	0.058140000	-1.155456000	1.970495000
F	-3.474747000	0.226379000	-2.372644000
F	-5.721901000	1.670466000	-2.316074000
F	-6.235666000	3.325705000	-0.215657000
F	-4.456340000	3.496438000	1.824636000
F	-2.228139000	2.072621000	1.803983000
F	-2.800901000	-0.812862000	1.913750000
F	-3.585425000	-3.309558000	2.418464000
F	-2.997403000	-5.310900000	0.673039000
F	-1.621146000	-4.743101000	-1.604102000
F	-0.879281000	-2.276817000	-2.157579000
O	-0.891036000	0.320779000	-1.929920000
C	0.362742000	0.094467000	-2.512207000
O	0.467450000	0.112018000	-3.686783000
H	-1.588855000	0.342338000	-2.611900000

J7

	Coordinates (Angstroms)		
	X	Y	Z
B	0.827091000	0.080958000	-0.007814000
C	2.430870000	-0.123436000	0.251711000
C	3.364439000	-0.058543000	-0.773558000
C	2.973263000	-0.319108000	1.512389000
C	4.728897000	-0.182776000	-0.583568000
C	4.333403000	-0.448063000	1.751761000
C	5.220347000	-0.377702000	0.695198000
C	0.180593000	-0.967457000	-1.087672000
C	0.677295000	-2.252042000	-1.279009000
C	-0.992685000	-0.714577000	-1.787660000
C	0.081293000	-3.203641000	-2.092138000
C	-1.612346000	-1.625960000	-2.625195000
C	-1.075225000	-2.888539000	-2.778439000

F	2.952799000	0.113634000	-2.040546000
F	5.574922000	-0.119429000	-1.615853000
F	6.531622000	-0.499344000	0.903066000
F	4.792716000	-0.641225000	2.992143000
F	2.184495000	-0.412954000	2.599718000
F	-1.630887000	0.462427000	-1.656555000
F	-2.755883000	-1.310984000	-3.242027000
F	-1.678206000	-3.793608000	-3.550002000
F	0.609251000	-4.425747000	-2.205829000
F	1.787320000	-2.664436000	-0.639203000
C	0.332646000	3.622614000	-1.766824000
C	0.155466000	4.454417000	-0.675982000
C	0.205923000	3.913309000	0.593926000
C	0.427551000	2.554891000	0.741076000
C	0.584826000	1.670898000	-0.316482000
C	0.548636000	2.268563000	-1.569898000
C	-4.108768000	-0.936996000	-0.172593000
C	-3.604992000	-2.235787000	-0.220722000
C	-2.637064000	-2.600276000	0.662252000
C	-2.526812000	-0.428552000	1.525540000
O	-1.933418000	0.477084000	2.286972000
H	-3.973954000	-2.958519000	-0.935131000
H	-2.244022000	-3.603165000	0.683112000
C	-3.586724000	-0.001016000	0.697766000
N	-2.128795000	-1.726802000	1.572686000
H	-4.894539000	-0.662157000	-0.861589000
F	0.448200000	2.104220000	2.014510000
F	0.017964000	4.694834000	1.661055000
F	-0.060388000	5.758437000	-0.848250000
F	0.291969000	4.134207000	-3.000430000
F	0.695807000	1.535328000	-2.684406000
C	-0.784035000	-1.445680000	3.779770000
H	-0.300835000	-2.078813000	4.526533000
H	-0.023763000	-0.742745000	3.440376000
H	-1.599731000	-0.916891000	4.270739000
C	-2.182286000	-3.431891000	3.343368000
H	-2.489008000	-4.228197000	2.664458000
H	-1.638440000	-3.895108000	4.168474000
H	-3.075283000	-2.949207000	3.748549000
C	-0.046437000	-3.040439000	2.024008000
H	0.526734000	-3.544512000	2.805197000
H	-0.305083000	-3.789349000	1.273898000
H	0.591201000	-2.283657000	1.570539000
C	-4.729526000	1.655619000	2.182924000
H	-3.975823000	1.566491000	2.967156000
H	-5.158844000	2.659923000	2.243168000
H	-5.527359000	0.933645000	2.381839000
C	-3.039680000	2.493014000	0.510497000
H	-2.526008000	2.300863000	-0.433499000
H	-3.513883000	3.476395000	0.443106000
H	-2.300703000	2.531810000	1.307182000
C	-5.243016000	1.649726000	-0.252247000
H	-6.083090000	0.965340000	-0.102675000
H	-5.627138000	2.667294000	-0.149190000
H	-4.873402000	1.534566000	-1.275628000
H	0.264296000	-0.173459000	1.038373000
H	-0.968815000	0.384187000	2.255629000

C	-1.264495000	-2.395451000	2.681178000
C	-4.128750000	1.437079000	0.781518000

J8

	Coordinates (Angstroms)		
	X	Y	Z
C	4.216691000	0.694725000	1.178589000
C	4.431857000	-0.643138000	1.561509000
C	4.347885000	-1.610744000	0.617767000
C	3.658736000	-0.045103000	-1.063911000
O	3.199256000	0.157222000	-2.203317000
H	4.687187000	-0.905783000	2.578653000
H	4.544304000	-2.638959000	0.865460000
C	3.879847000	1.023662000	-0.103835000
N	4.004455000	-1.342375000	-0.674413000
H	4.335722000	1.467459000	1.927140000
C	4.791894000	-2.098417000	-2.911718000
H	4.818823000	-2.952837000	-3.592318000
H	4.386250000	-1.241793000	-3.442491000
H	5.818196000	-1.874956000	-2.605789000
C	4.538325000	-3.760227000	-1.104534000
H	3.941713000	-4.168424000	-0.285848000
H	4.539444000	-4.504205000	-1.903214000
H	5.571512000	-3.629613000	-0.772864000
C	2.486502000	-2.757105000	-2.055492000
H	2.457766000	-3.584152000	-2.769747000
H	1.926518000	-3.055834000	-1.166430000
H	2.003925000	-1.896527000	-2.510606000
C	4.812911000	2.764982000	-1.640011000
H	4.709136000	2.087072000	-2.489042000
H	4.707489000	3.791067000	-2.007318000
H	5.819140000	2.654413000	-1.223900000
C	2.345460000	2.750170000	-1.146607000
H	1.583161000	2.300700000	-0.508170000
H	2.158974000	3.827411000	-1.196741000
H	2.243992000	2.340132000	-2.149728000
C	3.961334000	3.459421000	0.592438000
H	4.949045000	3.350453000	1.049387000
H	3.884701000	4.481458000	0.211575000
H	3.204585000	3.341631000	1.373656000
C	3.946124000	-2.472300000	-1.687315000
C	3.745019000	2.479368000	-0.568946000
B	-1.351862000	-0.004144000	0.001429000
C	-1.039595000	-1.370802000	0.840806000
C	0.259093000	-1.862432000	0.953010000
C	-2.009140000	-2.153859000	1.454484000
C	0.579151000	-3.054407000	1.573371000
C	-1.726631000	-3.350502000	2.097806000
C	-0.426302000	-3.811266000	2.148470000
C	-0.805321000	1.388025000	0.656006000
C	-1.089468000	2.617011000	0.064101000
C	0.000429000	1.474401000	1.786927000
C	-0.597995000	3.828855000	0.511007000
C	0.520769000	2.668851000	2.262524000
C	0.227670000	3.853906000	1.619398000
C	-2.900981000	0.048445000	-0.514150000
C	-3.353151000	-0.745828000	-1.556983000

C	-3.885212000	0.811698000	0.099840000
C	-4.664443000	-0.769289000	-1.999347000
C	-5.207726000	0.820329000	-0.306098000
C	-5.601196000	0.026868000	-1.368929000
F	1.285455000	-1.153749000	0.469069000
F	1.843072000	-3.479640000	1.628323000
F	-0.140623000	-4.959919000	2.752191000
F	-2.703907000	-4.059385000	2.663096000
F	-3.296489000	-1.784078000	1.473675000
F	-1.865351000	2.673433000	-1.033160000
F	-0.883520000	4.959609000	-0.132927000
F	0.748976000	4.998349000	2.046177000
F	1.323237000	2.675740000	3.327528000
F	0.329151000	0.394035000	2.504453000
F	-3.590329000	1.571874000	1.164738000
F	-6.106618000	1.578052000	0.321655000
F	-6.868230000	0.021993000	-1.771183000
F	-5.028592000	-1.553947000	-3.012898000
F	-2.504347000	-1.584745000	-2.193146000
O	-0.421148000	-0.227342000	-1.308836000
C	-0.210156000	0.690608000	-2.462618000
H	-1.152819000	0.757659000	-3.003979000
H	0.064575000	1.634593000	-2.001334000
O	0.740854000	0.153162000	-3.260421000
H	1.629330000	0.238752000	-2.846129000
H	-0.527496000	-1.120721000	-1.669557000

TSJ2-3

	Coordinates (Angstroms)		
	X	Y	Z
C	4.781498000	1.156833000	-0.075479000
C	5.229772000	-0.032230000	0.504368000
C	4.672403000	-1.207134000	0.108264000
C	3.104444000	-0.077801000	-1.262930000
O	2.054230000	-0.158616000	-1.997895000
H	6.024279000	-0.040221000	1.237323000
H	5.022392000	-2.146846000	0.496888000
C	3.740056000	1.166884000	-0.975154000
N	3.654439000	-1.249265000	-0.790856000
H	5.272607000	2.080106000	0.199958000
C	3.405361000	-2.554583000	-2.882307000
H	3.185859000	-3.543638000	-3.290411000
H	2.748078000	-1.831946000	-3.360046000
H	4.444279000	-2.310522000	-3.121234000
C	4.084796000	-3.729218000	-0.811772000
H	3.961854000	-3.870451000	0.264978000
H	3.755777000	-4.649662000	-1.296803000
H	5.143336000	-3.594399000	-1.046350000
C	1.761083000	-2.910938000	-0.976332000
H	1.510829000	-3.900701000	-1.365754000
H	1.653743000	-2.944837000	0.109167000
H	1.048068000	-2.202396000	-1.383425000
C	3.341822000	2.285405000	-3.191815000
H	2.676409000	1.495231000	-3.539148000
H	3.047553000	3.219863000	-3.679096000
H	4.362225000	2.047121000	-3.508075000
C	1.840041000	2.829205000	-1.242443000

H	1.768630000	2.956318000	-0.159248000
H	1.553964000	3.773531000	-1.716097000
H	1.121443000	2.073392000	-1.546730000
C	4.177377000	3.641956000	-1.289124000
H	5.221547000	3.466446000	-1.564991000
H	3.836359000	4.529224000	-1.828684000
H	4.132563000	3.870850000	-0.220307000
C	3.207354000	-2.601689000	-1.362704000
C	3.272098000	2.459776000	-1.663133000
B	-1.101532000	-0.005616000	-0.070232000
C	-0.310298000	-0.909698000	1.046100000
C	0.953492000	-0.520970000	1.472955000
C	-0.778096000	-2.077285000	1.632188000
C	1.716024000	-1.220444000	2.388643000
C	-0.043303000	-2.818239000	2.547869000
C	1.214254000	-2.391921000	2.926341000
C	-1.241254000	1.569588000	0.392972000
C	-1.380653000	2.572047000	-0.554461000
C	-1.216508000	2.020506000	1.704495000
C	-1.425560000	3.922105000	-0.257920000
C	-1.265827000	3.360297000	2.052678000
C	-1.362875000	4.321504000	1.063741000
C	-2.546757000	-0.630698000	-0.536601000
C	-2.599352000	-1.769285000	-1.335538000
C	-3.786283000	-0.101546000	-0.205045000
C	-3.774937000	-2.328690000	-1.801248000
C	-4.988382000	-0.631908000	-0.650495000
C	-4.984102000	-1.750040000	-1.459800000
F	1.496460000	0.614260000	0.998776000
F	2.921991000	-0.784054000	2.756756000
F	1.931677000	-3.091867000	3.801731000
F	-0.545463000	-3.939265000	3.068103000
F	-1.995549000	-2.565270000	1.353748000
F	-1.480307000	2.256485000	-1.866444000
F	-1.518897000	4.833730000	-1.227776000
F	-1.403948000	5.613892000	1.380958000
F	-1.226850000	3.732201000	3.333977000
F	-1.157458000	1.152601000	2.728372000
F	-3.895784000	0.967565000	0.599612000
F	-6.148039000	-0.071594000	-0.299758000
F	-6.127980000	-2.272770000	-1.895167000
F	-3.755450000	-3.426057000	-2.561253000
F	-1.473239000	-2.423696000	-1.661914000
O	-0.212704000	-0.040392000	-1.325519000
H	0.969414000	-0.089832000	-1.547181000
H	-0.643446000	0.362193000	-2.085667000

TSJ3-4

	Coordinates (Angstroms)		
	X	Y	Z
C	4.813734000	1.908145000	-0.246716000
C	5.193897000	1.310686000	0.959391000
C	4.850484000	0.017415000	1.187268000
C	3.698182000	-0.122673000	-0.892299000
O	3.025421000	-0.846188000	-1.694802000
H	5.753181000	1.854281000	1.707736000
H	5.134481000	-0.480214000	2.097516000

C	4.085237000	1.225943000	-1.192128000
N	4.127041000	-0.700150000	0.287339000
H	5.116128000	2.930658000	-0.426898000
C	4.773259000	-2.957351000	-0.460920000
H	4.618900000	-4.028977000	-0.311560000
H	4.519254000	-2.714061000	-1.492605000
H	5.829491000	-2.734409000	-0.286520000
C	4.293872000	-2.576429000	1.951562000
H	3.708514000	-2.044108000	2.705818000
H	4.084308000	-3.641248000	2.063815000
H	5.358717000	-2.437765000	2.148461000
C	2.401050000	-2.536496000	0.349267000
H	2.122497000	-3.324810000	1.051368000
H	1.768799000	-1.675097000	0.553810000
H	2.184523000	-2.888060000	-0.656548000
C	4.163500000	0.963158000	-3.710522000
H	3.626383000	0.016514000	-3.728159000
H	3.981818000	1.481526000	-4.656726000
H	5.235252000	0.749669000	-3.645334000
C	2.205807000	2.128013000	-2.617255000
H	1.899024000	2.845860000	-1.853253000
H	1.951324000	2.542647000	-3.597645000
H	1.616686000	1.225901000	-2.469407000
C	4.424294000	3.216556000	-2.724109000
H	5.513346000	3.118102000	-2.682130000
H	4.163984000	3.619592000	-3.706254000
H	4.110593000	3.951929000	-1.978143000
H	1.753498000	-0.911979000	-1.927455000
C	3.889461000	-2.190706000	0.526203000
C	3.718220000	1.864150000	-2.542739000
B	-1.348393000	0.066268000	-0.273838000
C	-0.315865000	-0.084878000	0.998956000
C	0.680732000	0.874839000	1.153157000
C	-0.355256000	-1.041561000	2.003889000
C	1.593033000	0.876584000	2.194116000
C	0.561166000	-1.095847000	3.043031000
C	1.545874000	-0.132175000	3.137571000
C	-2.164794000	1.473235000	-0.015817000
C	-2.172821000	2.583987000	-0.841932000
C	-2.880775000	1.627085000	1.164845000
C	-2.823404000	3.768364000	-0.530318000
C	-3.545750000	2.785347000	1.518739000
C	-3.513423000	3.871618000	0.661384000
C	-2.349275000	-1.217102000	-0.536354000
C	-1.800804000	-2.483383000	-0.698689000
C	-3.722878000	-1.166174000	-0.726434000
C	-2.524805000	-3.619596000	-1.000407000
C	-4.495080000	-2.280933000	-1.025797000
C	-3.895187000	-3.516249000	-1.163089000
F	0.782747000	1.895480000	0.289544000
F	2.498238000	1.850027000	2.305142000
F	2.440153000	-0.172250000	4.124952000
F	0.509508000	-2.077735000	3.945690000
F	-1.296272000	-1.996769000	2.017953000
F	-1.538129000	2.568081000	-2.035374000
F	-2.789925000	4.804044000	-1.371301000
F	-4.145701000	4.999573000	0.979141000

F	-4.219447000	2.866196000	2.667517000
F	-2.973169000	0.596708000	2.023111000
F	-4.402115000	-0.011092000	-0.641077000
F	-5.814414000	-2.164781000	-1.189998000
F	-4.622223000	-4.592756000	-1.452049000
F	-1.923729000	-4.802924000	-1.133992000
F	-0.469850000	-2.652446000	-0.559370000
O	-0.459966000	0.188957000	-1.507282000
C	0.617608000	-1.101400000	-2.441323000
O	0.022912000	-1.561394000	-3.292652000
H	-0.921760000	0.645517000	-2.218342000

TSJ1-7

	Coordinates (Angstroms)		
	X	Y	Z
C	-3.840560000	-2.123769000	-0.284956000
C	-4.594449000	-1.243964000	0.517523000
C	-4.478592000	0.090452000	0.306023000
C	-2.654578000	-0.236110000	-1.204069000
O	-1.665396000	0.269142000	-1.773931000
H	-5.295484000	-1.611335000	1.253636000
H	-5.097254000	0.794815000	0.836779000
C	-2.917910000	-1.660890000	-1.182734000
H	-4.021516000	-3.185877000	-0.184360000
N	-3.577866000	0.602988000	-0.579950000
B	0.977258000	0.072257000	0.027024000
C	2.015200000	-1.176280000	0.063925000
C	2.416991000	-1.814526000	1.229230000
C	2.622789000	-1.627726000	-1.099438000
C	3.341534000	-2.845696000	1.239695000
C	3.541456000	-2.660415000	-1.133976000
C	3.906540000	-3.272621000	0.051417000
C	-0.206253000	0.059351000	1.129016000
C	-0.654264000	1.194453000	1.791939000
C	-0.873991000	-1.115173000	1.465695000
C	-1.713369000	1.180585000	2.686622000
C	-1.920654000	-1.170463000	2.364944000
C	-2.358354000	-0.006365000	2.968625000
C	1.752623000	1.465509000	-0.229632000
C	2.698520000	1.902871000	0.686330000
C	1.576484000	2.275837000	-1.339238000
C	3.423683000	3.068899000	0.529932000
C	2.281383000	3.453011000	-1.533761000
C	3.211921000	3.851075000	-0.593022000
F	1.915395000	-1.453313000	2.416011000
F	3.693079000	-3.428148000	2.385381000
F	4.795148000	-4.260664000	0.049446000
F	4.078184000	-3.059771000	-2.285561000
F	2.306650000	-1.054928000	-2.274121000
F	-0.093104000	2.389895000	1.580223000
F	-2.133770000	2.314525000	3.248734000
F	-3.396516000	-0.030550000	3.796082000
F	-2.524593000	-2.326466000	2.632207000
F	-0.516034000	-2.280489000	0.911149000
F	0.688253000	1.946684000	-2.288433000
F	2.070711000	4.199358000	-2.617509000
F	3.898120000	4.977339000	-0.762270000

F	4.315023000	3.447762000	1.445099000
F	2.918289000	1.188443000	1.800019000
H	-0.161351000	0.036346000	-1.085565000
H	0.582836000	-0.263364000	-1.248317000
C	-3.638003000	2.068620000	-0.983367000
C	-2.407085000	2.829391000	-0.490389000
C	-3.760056000	2.130841000	-2.511948000
C	-4.879285000	2.736388000	-0.386523000
H	-2.397115000	2.875403000	0.599264000
H	-1.484832000	2.382367000	-0.852073000
H	-2.463473000	3.856259000	-0.861696000
H	-4.620116000	1.545210000	-2.849211000
H	-3.916479000	3.170341000	-2.808673000
H	-2.861662000	1.764004000	-3.004319000
H	-4.912110000	3.757880000	-0.770361000
H	-5.805480000	2.239330000	-0.685653000
H	-4.837988000	2.803897000	0.703452000
C	-2.262007000	-2.567358000	-2.238820000
C	-0.727198000	-2.464143000	-2.314335000
C	-2.613088000	-4.039930000	-1.988077000
C	-2.841304000	-2.154837000	-3.606861000
H	-0.413999000	-1.512934000	-2.739327000
H	-0.261329000	-2.588383000	-1.335720000
H	-0.349856000	-3.258153000	-2.966138000
H	-3.690983000	-4.221053000	-2.017186000
H	-2.158863000	-4.652460000	-2.771389000
H	-2.226684000	-4.388772000	-1.025194000
H	-2.403433000	-2.770256000	-4.399301000
H	-3.926987000	-2.289290000	-3.630769000
H	-2.616157000	-1.108529000	-3.828656000

TSJ7

	Coordinates (Angstroms)		
	X	Y	Z
H	-0.500315000	-0.222748000	-1.184757000
B	-1.225404000	-0.012601000	-0.074712000
H	1.686742000	0.744220000	-2.288116000
C	-0.943566000	1.543049000	0.218703000
C	-1.323039000	2.501168000	-0.717299000
C	-0.359420000	2.054929000	1.371697000
C	-1.155990000	3.861223000	-0.536665000
C	-0.203287000	3.412312000	1.603028000
C	-0.589568000	4.321765000	0.638218000
C	-2.726763000	-0.407134000	-0.534327000
C	-2.954675000	-1.593340000	-1.216478000
C	-3.872357000	0.310878000	-0.216012000
C	-4.205751000	-2.046881000	-1.589608000
C	-5.146891000	-0.107541000	-0.563823000
C	-5.316132000	-1.292239000	-1.256638000
C	-0.695424000	-1.094949000	1.006987000
C	0.322473000	-2.007968000	0.809441000
C	-1.350062000	-1.201748000	2.227710000
C	0.684109000	-2.965913000	1.743431000
C	-1.014375000	-2.127963000	3.196445000
C	0.016033000	-3.020556000	2.950668000
F	-1.907666000	2.117836000	-1.858486000
F	-1.533880000	4.727784000	-1.476266000

F	-0.407184000	5.623770000	0.831534000
F	0.347961000	3.847175000	2.738036000
F	0.094503000	1.249509000	2.342673000
F	-1.904634000	-2.369129000	-1.569162000
F	-4.349665000	-3.189861000	-2.259348000
F	-6.533524000	-1.702835000	-1.597461000
F	-6.212673000	0.619196000	-0.230295000
F	-3.797109000	1.456263000	0.473176000
F	-2.350821000	-0.355057000	2.514001000
F	-1.667390000	-2.172766000	4.357645000
F	0.355049000	-3.923495000	3.866853000
F	1.667402000	-3.831062000	1.486976000
F	1.018974000	-2.010568000	-0.340537000
C	0.044868000	-0.219440000	-2.470706000
O	0.734538000	0.882793000	-2.571556000
H	0.580189000	-1.155220000	-2.316227000
C	3.482982000	0.293819000	1.540255000
C	3.648237000	-1.096626000	1.684279000
C	3.907124000	-1.837358000	0.578117000
C	3.508011000	0.027191000	-0.858140000
O	3.228415000	0.432013000	-2.009605000
H	3.638900000	-1.570825000	2.655809000
H	4.119167000	-2.889353000	0.653406000
C	3.475036000	0.885619000	0.306399000
N	3.889862000	-1.298251000	-0.673070000
H	3.389227000	0.894979000	2.435232000
C	5.416577000	-1.392821000	-2.615046000
H	5.771054000	-2.019279000	-3.436965000
H	5.069408000	-0.447456000	-3.027021000
H	6.256708000	-1.204136000	-1.940748000
C	4.856058000	-3.488226000	-1.420082000
H	4.095643000	-4.123804000	-0.959412000
H	5.212553000	-4.005321000	-2.312774000
H	5.704077000	-3.383903000	-0.738691000
C	3.096018000	-2.404153000	-2.783272000
H	3.409165000	-3.078828000	-3.584107000
H	2.296128000	-2.894452000	-2.224099000
H	2.720129000	-1.488166000	-3.233188000
C	4.904959000	2.744621000	-0.507869000
H	5.027735000	2.253308000	-1.476123000
H	4.987433000	3.824948000	-0.665116000
H	5.723207000	2.432708000	0.148461000
C	2.424107000	2.956891000	-0.793660000
H	1.464971000	2.490959000	-0.571207000
H	2.322493000	4.035838000	-0.643024000
H	2.644402000	2.781975000	-1.845560000
C	3.442685000	3.125814000	1.470018000
H	4.258091000	2.846175000	2.143225000
H	3.505516000	4.205045000	1.308231000
H	2.496251000	2.919809000	1.974811000
C	4.298541000	-2.135797000	-1.873636000
C	3.537531000	2.408573000	0.117464000
O	-0.994374000	-0.249137000	-3.297648000
H	-1.351899000	-1.145086000	-3.328159000

TSJ8

Coordinates (Angstroms)

	X	Y	Z
B	-1.528317000	-0.119430000	-0.388102000
C	-0.653300000	-0.831453000	0.809925000
C	0.647230000	-1.289153000	0.680223000
C	-1.222087000	-1.067110000	2.055646000
C	1.337901000	-1.938137000	1.692930000
C	-0.571814000	-1.705120000	3.095419000
C	0.725801000	-2.150010000	2.911313000
C	-1.722991000	1.507660000	-0.172735000
C	-2.249946000	2.250063000	-1.222393000
C	-1.327425000	2.267301000	0.916360000
C	-2.337831000	3.628297000	-1.237121000
C	-1.386307000	3.653604000	0.945354000
C	-1.890603000	4.342142000	-0.138946000
C	-2.961096000	-0.938847000	-0.497940000
C	-2.947506000	-2.271987000	-0.895357000
C	-4.221242000	-0.438159000	-0.200054000
C	-4.085296000	-3.052321000	-1.014840000
C	-5.384874000	-1.184213000	-0.306250000
C	-5.318198000	-2.500736000	-0.720943000
F	1.344490000	-1.103080000	-0.450253000
F	2.594864000	-2.351611000	1.496273000
F	1.374688000	-2.768208000	3.897818000
F	-1.176305000	-1.891845000	4.271833000
F	-2.468522000	-0.634899000	2.313721000
F	-2.720250000	1.617301000	-2.319802000
F	-2.841998000	4.274744000	-2.291277000
F	-1.952313000	5.672791000	-0.128120000
F	-0.947756000	4.326175000	2.013168000
F	-0.843256000	1.697299000	2.031440000
F	-4.382743000	0.819911000	0.238533000
F	-6.568931000	-0.644237000	-0.007294000
F	-6.426644000	-3.230578000	-0.831271000
F	-4.003192000	-4.326659000	-1.405808000
F	-1.784334000	-2.882698000	-1.179669000
H	2.279535000	1.063342000	-1.551197000
O	-0.815210000	-0.309879000	-1.703064000
C	0.484678000	0.984859000	-2.612790000
O	1.187515000	1.581122000	-1.775637000
H	-0.363324000	1.513347000	-3.044960000
H	0.879037000	0.125986000	-3.157864000
H	-1.460909000	-0.468942000	-2.395945000
C	5.033716000	0.088518000	1.540514000
C	5.698331000	-1.098487000	1.228566000
C	5.567379000	-1.606736000	-0.023462000
C	4.065593000	0.124781000	-0.655679000
O	3.334455000	0.590115000	-1.596291000
H	6.323303000	-1.605566000	1.950097000
H	6.087509000	-2.502389000	-0.311365000
C	4.230205000	0.734880000	0.629295000
N	4.783841000	-1.014135000	-0.961868000
H	5.175677000	0.507881000	2.526528000
C	5.212246000	-0.573084000	-3.380384000
H	5.267473000	-1.044492000	-4.364341000
H	4.550520000	0.286179000	-3.444932000
H	6.216215000	-0.231943000	-3.112282000
C	5.648229000	-2.831517000	-2.480402000

H	5.348532000	-3.650768000	-1.822699000
H	5.569303000	-3.196583000	-3.505667000
H	6.697099000	-2.581837000	-2.303079000
C	3.297292000	-2.100738000	-2.656879000
H	3.296631000	-2.618146000	-3.619258000
H	2.975489000	-2.807114000	-1.888333000
H	2.582485000	-1.285666000	-2.703049000
C	3.731351000	3.128117000	-0.094137000
H	3.248733000	2.883119000	-1.036974000
H	3.306913000	4.073154000	0.256316000
H	4.796290000	3.289549000	-0.288859000
C	2.049365000	1.806948000	1.276896000
H	1.931294000	1.104394000	2.106552000
H	1.563751000	2.745765000	1.557915000
H	1.516599000	1.406340000	0.417763000
C	4.140053000	2.648267000	2.287210000
H	5.218248000	2.812802000	2.198720000
H	3.668231000	3.615171000	2.479051000
H	3.950172000	2.020713000	3.161932000
C	4.720945000	-1.616764000	-2.370713000
C	3.539468000	2.059347000	0.999014000

TSJ9

	Coordinates (Angstroms)		
	X	Y	Z
H	-0.410341000	-0.209091000	-1.096947000
B	-1.191128000	-0.025233000	-0.128830000
H	2.131661000	0.664656000	-2.351084000
C	-1.035737000	1.558751000	0.178537000
C	-1.406093000	2.476343000	-0.799454000
C	-0.507368000	2.128254000	1.328793000
C	-1.285000000	3.846529000	-0.662049000
C	-0.384402000	3.496776000	1.515550000
C	-0.766038000	4.363632000	0.511133000
C	-2.662323000	-0.492368000	-0.650656000
C	-2.781874000	-1.645618000	-1.414257000
C	-3.863252000	0.131225000	-0.343907000
C	-3.988545000	-2.149998000	-1.865681000
C	-5.094551000	-0.340234000	-0.772011000
C	-5.158543000	-1.488134000	-1.539742000
C	-0.715130000	-1.060687000	1.029827000
C	0.305058000	-1.980499000	0.868449000
C	-1.394980000	-1.146711000	2.236995000
C	0.653828000	-2.913915000	1.832457000
C	-1.076210000	-2.050883000	3.232653000
C	-0.037334000	-2.943838000	3.027725000
F	-1.921247000	2.040186000	-1.962300000
F	-1.653158000	4.669700000	-1.645380000
F	-0.622733000	5.678269000	0.663631000
F	0.132169000	3.983200000	2.647299000
F	-0.055137000	1.367652000	2.340358000
F	-1.678755000	-2.341308000	-1.751288000
F	-4.035591000	-3.262300000	-2.602299000
F	-6.334034000	-1.953570000	-1.958445000
F	-6.219248000	0.301117000	-0.447319000
F	-3.887669000	1.244986000	0.404049000
F	-2.412297000	-0.306102000	2.482401000

F	-1.753658000	-2.076458000	4.382153000
F	0.288214000	-3.825066000	3.971966000
F	1.650762000	-3.778523000	1.618965000
F	1.020552000	-2.012982000	-0.270435000
C	0.207359000	-0.106092000	-2.611794000
O	0.988543000	0.868372000	-2.688697000
H	-0.794540000	0.008544000	-3.025035000
H	0.586690000	-1.116398000	-2.452795000
C	3.370698000	0.308764000	1.499355000
C	3.581500000	-1.063421000	1.653067000
C	3.874815000	-1.807382000	0.552430000
C	3.473382000	0.034621000	-0.877487000
O	3.262510000	0.439237000	-2.080867000
H	3.574530000	-1.532930000	2.626746000
H	4.118762000	-2.850735000	0.638041000
C	3.370790000	0.897435000	0.253674000
N	3.848463000	-1.276858000	-0.696553000
H	3.230544000	0.910994000	2.386524000
C	5.442270000	-1.408082000	-2.587867000
H	5.807734000	-2.041803000	-3.399022000
H	5.138102000	-0.452236000	-3.009038000
H	6.262778000	-1.247258000	-1.883114000
C	4.784774000	-3.493416000	-1.421666000
H	3.993870000	-4.100665000	-0.974589000
H	5.139735000	-4.023236000	-2.307089000
H	5.624723000	-3.416127000	-0.727344000
C	3.100676000	-2.366519000	-2.839597000
H	3.407974000	-3.097683000	-3.590689000
H	2.249961000	-2.782081000	-2.295213000
H	2.796302000	-1.459337000	-3.355316000
C	4.783982000	2.789051000	-0.510103000
H	4.938514000	2.318069000	-1.483761000
H	4.852843000	3.872897000	-0.645790000
H	5.590394000	2.477903000	0.160825000
C	2.306618000	2.967729000	-0.852168000
H	1.344778000	2.499471000	-0.647192000
H	2.201174000	4.045569000	-0.699608000
H	2.543996000	2.803863000	-1.901970000
C	3.267364000	3.127530000	1.433949000
H	4.075540000	2.862143000	2.121217000
H	3.309525000	4.208446000	1.279541000
H	2.315759000	2.897710000	1.917919000
C	4.280574000	-2.127341000	-1.894036000
C	3.405928000	2.425733000	0.076401000

K1

	Coordinates (Angstroms)		
	X	Y	Z
B	-3.011503000	-0.329957000	0.278972000
C	-2.275011000	3.462582000	0.571415000
C	-0.869763000	3.494671000	0.413259000
C	-0.147140000	2.288811000	0.204557000
C	-2.279060000	1.055762000	0.338882000
C	-2.965483000	2.281271000	0.526695000
C	2.158178000	1.182305000	-0.150170000
C	3.568188000	1.372994000	-0.192403000
C	4.345437000	0.236783000	-0.324291000

C	2.400026000	-1.135816000	-0.496376000
O	1.918381000	-2.239129000	-0.698034000
H	5.421042000	0.333547000	-0.325264000
N	3.832052000	-0.983049000	-0.444127000
C	4.721650000	-2.205020000	-0.542637000
H	-4.043456000	2.283460000	0.651577000
H	-2.794895000	4.401874000	0.731907000
C	1.300665000	2.347382000	0.066769000
C	4.161967000	2.665322000	-0.081178000
C	1.935882000	3.597929000	0.160381000
C	-0.179463000	4.739066000	0.475729000
C	1.168105000	4.785777000	0.356442000
C	3.364453000	3.741130000	0.076566000
H	5.241505000	2.759137000	-0.131117000
H	3.782393000	4.737927000	0.158706000
H	1.694338000	5.732494000	0.413932000
H	-0.752829000	5.647440000	0.629080000
N	1.647857000	-0.030048000	-0.308261000
C	-0.901373000	1.089977000	0.183169000
H	-0.367645000	0.167173000	0.029748000
C	4.364695000	-3.160062000	0.602056000
C	4.523917000	-2.849431000	-1.918405000
C	6.194496000	-1.817602000	-0.397291000
H	5.207229000	-3.697243000	-2.011676000
H	3.503583000	-3.202294000	-2.050405000
H	4.760524000	-2.131480000	-2.708951000
H	4.487060000	-2.657912000	1.566082000
H	3.343822000	-3.525277000	0.516598000
H	5.047854000	-4.012369000	0.573001000
H	6.776591000	-2.739648000	-0.444069000
H	6.542518000	-1.171900000	-1.207926000
H	6.407992000	-1.342409000	0.564027000
C	-2.265345000	-1.612431000	0.788740000
C	-1.364973000	-1.561031000	1.866002000
C	-2.431031000	-2.849885000	0.146259000
C	-0.666832000	-2.686437000	2.283142000
H	-1.208971000	-0.621714000	2.386046000
C	-1.714642000	-3.973622000	0.540270000
H	-3.117210000	-2.928341000	-0.691078000
C	-0.831945000	-3.893552000	1.612001000
H	0.019523000	-2.620246000	3.120485000
H	-1.844021000	-4.912487000	0.012665000
H	-0.273001000	-4.769915000	1.922474000
C	-4.456226000	-0.439745000	-0.321069000
C	-5.378571000	-1.383157000	0.160233000
C	-4.872921000	0.393820000	-1.371666000
C	-6.658440000	-1.482512000	-0.371569000
H	-5.089732000	-2.046330000	0.969534000
C	-6.141229000	0.280408000	-1.927880000
H	-4.185026000	1.132692000	-1.770594000
C	-7.039152000	-0.654794000	-1.423183000
H	-7.357039000	-2.211176000	0.025628000
H	-6.432772000	0.924635000	-2.750501000
H	-8.032621000	-0.740495000	-1.850483000

K1_CO

Coordinates (Angstroms)

	X	Y	Z
B	-2.761944000	-0.438375000	-0.201414000
C	-1.934131000	3.310356000	0.484834000
C	-0.522403000	3.330418000	0.491827000
C	0.207862000	2.136003000	0.240847000
C	-1.936528000	0.947098000	-0.004126000
C	-2.626751000	2.154627000	0.234342000
C	2.538575000	1.047705000	-0.003556000
C	3.948364000	1.235842000	0.068329000
C	4.739526000	0.123669000	-0.150441000
C	2.814546000	-1.230193000	-0.543311000
O	2.348344000	-2.318220000	-0.844013000
H	5.813103000	0.223286000	-0.087071000
N	4.241845000	-1.076135000	-0.431901000
C	5.146208000	-2.273490000	-0.641169000
H	-3.711722000	2.169496000	0.226661000
H	-2.462404000	4.238549000	0.680415000
C	1.663988000	2.191141000	0.253074000
C	4.530046000	2.505255000	0.358778000
C	2.290343000	3.421377000	0.525978000
C	0.162278000	4.551169000	0.753767000
C	1.514594000	4.593412000	0.772883000
C	3.720721000	3.561346000	0.573974000
H	5.610074000	2.597408000	0.399705000
H	4.129452000	4.541381000	0.791993000
H	2.037021000	5.522015000	0.975758000
H	-0.422220000	5.446151000	0.941238000
N	2.045327000	-0.146373000	-0.301730000
C	-0.553195000	0.962568000	0.005242000
H	-0.006438000	0.045907000	-0.159958000
C	4.790438000	-3.342589000	0.397849000
C	4.970423000	-2.780671000	-2.076208000
C	6.612909000	-1.886708000	-0.443194000
H	5.667508000	-3.605092000	-2.245377000
H	3.957185000	-3.133834000	-2.253316000
H	5.203498000	-1.985646000	-2.790392000
H	4.894741000	-2.936465000	1.408095000
H	3.775741000	-3.710383000	0.263548000
H	5.486369000	-4.178482000	0.292949000
H	7.207238000	-2.789677000	-0.593169000
H	6.954351000	-1.146521000	-1.171304000
H	6.816388000	-1.524240000	0.567951000
C	-2.701250000	-1.453385000	1.071552000
C	-2.207196000	-1.036681000	2.310461000
C	-3.179824000	-2.767461000	0.975375000
C	-2.205197000	-1.887457000	3.412968000
H	-1.816603000	-0.030496000	2.421855000
C	-3.172224000	-3.626687000	2.067432000
H	-3.577858000	-3.127090000	0.029188000
C	-2.685625000	-3.185839000	3.294464000
H	-1.821626000	-1.533994000	4.364483000
H	-3.547851000	-4.639134000	1.961948000
H	-2.679582000	-3.852327000	4.150406000
C	-4.241147000	-0.235908000	-0.857472000
C	-5.404894000	-0.564763000	-0.156835000
C	-4.392346000	0.348529000	-2.122281000
C	-6.666523000	-0.317303000	-0.692209000

H	-5.329361000	-1.018716000	0.825675000
C	-5.646992000	0.588920000	-2.669114000
H	-3.510598000	0.634552000	-2.691489000
C	-6.792183000	0.256002000	-1.951858000
H	-7.553463000	-0.576436000	-0.123179000
H	-5.732775000	1.039634000	-3.652278000
H	-7.774114000	0.442080000	-2.373495000
C	-1.966278000	-1.231584000	-1.339095000
O	-1.436261000	-1.810629000	-2.140612000

K2

	Coordinates (Angstroms)		
	X	Y	Z
B	-2.754812000	-0.469979000	-0.016534000
C	-1.836401000	3.300223000	0.027997000
C	-0.424242000	3.299968000	0.109217000
C	0.280182000	2.067126000	0.108516000
C	-1.877120000	0.877816000	0.017514000
C	-2.540308000	2.128055000	-0.038546000
C	2.580593000	0.908452000	0.014077000
C	3.990736000	1.059368000	0.139706000
C	4.751175000	-0.090496000	0.043958000
C	2.805026000	-1.403942000	-0.377844000
O	2.316040000	-2.491079000	-0.651818000
H	5.823002000	-0.022427000	0.157719000
N	4.225712000	-1.291455000	-0.183386000
H	-3.623004000	2.158192000	-0.122705000
H	-2.351407000	4.256070000	0.016300000
C	1.733761000	2.091815000	0.146315000
C	4.598888000	2.334782000	0.343178000
C	2.386330000	3.330692000	0.285763000
C	0.284854000	4.529712000	0.208213000
C	1.635524000	4.542653000	0.317512000
C	3.817548000	3.432179000	0.395025000
H	5.677479000	2.398562000	0.439408000
H	4.249451000	4.417762000	0.527328000
H	2.173491000	5.479221000	0.416232000
H	-0.278882000	5.457275000	0.210347000
O	-1.599117000	-1.695233000	-0.261255000
H	-0.999967000	-1.556231000	-1.010030000
C	-0.498880000	0.880543000	0.102518000
H	0.037258000	-0.050394000	0.168087000
N	2.062331000	-0.285180000	-0.243356000
C	5.095506000	-2.530127000	-0.255962000
C	4.631417000	-3.514087000	0.823434000
C	4.990416000	-3.124749000	-1.664249000
C	6.560645000	-2.182503000	0.012851000
H	4.702335000	-3.051366000	1.811950000
H	3.607869000	-3.840647000	0.654557000
H	5.287302000	-4.387977000	0.808130000
H	5.302368000	-2.388141000	-2.410183000
H	5.660453000	-3.985316000	-1.733727000
H	3.975638000	-3.448333000	-1.884350000
H	7.126742000	-3.114556000	-0.031952000
H	6.978223000	-1.510630000	-0.741810000
H	6.713169000	-1.753040000	1.006624000
C	-3.737437000	-0.519912000	-1.298849000

C	-5.120876000	-0.342405000	-1.175030000
C	-3.232509000	-0.652485000	-2.600462000
C	-5.959277000	-0.306922000	-2.286721000
H	-5.557593000	-0.227441000	-0.187646000
C	-4.060014000	-0.633657000	-3.717441000
H	-2.162141000	-0.762204000	-2.759144000
C	-5.431984000	-0.459908000	-3.563422000
H	-7.026650000	-0.163039000	-2.153208000
H	-3.633437000	-0.746370000	-4.708939000
H	-6.082595000	-0.439825000	-4.431383000
C	-3.396179000	-0.929941000	1.388955000
C	-4.208212000	-2.071618000	1.469399000
C	-3.136439000	-0.252272000	2.585521000
C	-4.727914000	-2.520878000	2.678242000
H	-4.448386000	-2.621297000	0.562345000
C	-3.659546000	-0.685668000	3.801075000
H	-2.509285000	0.634117000	2.570415000
C	-4.455621000	-1.824143000	3.851603000
H	-5.349290000	-3.410316000	2.705444000
H	-3.441756000	-0.136125000	4.711293000
H	-4.862968000	-2.166552000	4.797105000
H	-2.002910000	-2.568158000	-0.366144000

K3

	Coordinates (Angstroms)		
	X	Y	Z
B	-2.649626000	-0.643474000	0.100836000
C	-1.869775000	3.192923000	-0.415820000
C	-0.458483000	3.229785000	-0.331262000
C	0.263231000	2.019391000	-0.140513000
C	-1.874288000	0.787902000	-0.072184000
C	-2.551740000	2.010005000	-0.302592000
C	2.591685000	0.954761000	-0.001628000
C	4.000326000	1.125235000	0.059342000
C	4.780782000	-0.014297000	0.126137000
C	2.896183000	-1.329170000	0.072297000
O	2.371393000	-2.539037000	0.081768000
H	5.854329000	0.075456000	0.174129000
N	4.266790000	-1.251774000	0.132012000
H	-3.635717000	2.016365000	-0.375438000
H	-2.399280000	4.128802000	-0.568862000
C	1.718151000	2.092432000	-0.090463000
C	4.591340000	2.423044000	0.031901000
C	2.351295000	3.355444000	-0.161790000
C	0.227381000	4.471279000	-0.421229000
C	1.579867000	4.538333000	-0.322856000
C	3.778286000	3.493633000	-0.083171000
H	5.668447000	2.522548000	0.093691000
H	4.190694000	4.495401000	-0.123516000
H	2.095624000	5.490212000	-0.378802000
H	-0.355598000	5.376174000	-0.561648000
O	-1.591511000	-1.685067000	0.311142000
H	1.406809000	-2.436168000	0.038731000
N	2.113112000	-0.304947000	0.008099000
C	-0.494170000	0.832176000	0.003043000
H	0.007842000	-0.099538000	0.185145000
C	5.165406000	-2.495694000	0.191453000

C	4.835070000	-3.269710000	1.469422000
C	4.934580000	-3.316016000	-1.079805000
C	6.632026000	-2.071481000	0.238808000
H	4.952810000	-2.627695000	2.346053000
H	3.828236000	-3.681628000	1.460790000
H	5.541063000	-4.098250000	1.556096000
H	5.130839000	-2.708795000	-1.967181000
H	5.638179000	-4.151097000	-1.076765000
H	3.927237000	-3.722341000	-1.140463000
H	7.223010000	-2.987857000	0.273858000
H	6.938440000	-1.520616000	-0.653985000
H	6.874093000	-1.493171000	1.133967000
C	-3.655062000	-0.620137000	1.405617000
C	-3.505938000	0.282382000	2.468003000
C	-4.663104000	-1.585946000	1.557292000
C	-4.309961000	0.236591000	3.605021000
H	-2.739231000	1.050478000	2.407168000
C	-5.471350000	-1.650949000	2.688910000
H	-4.827431000	-2.307226000	0.759491000
C	-5.299849000	-0.733186000	3.721406000
H	-4.164489000	0.960791000	4.401413000
H	-6.240462000	-2.414169000	2.764021000
H	-5.931364000	-0.772567000	4.603262000
C	-3.485305000	-0.936084000	-1.288569000
C	-2.937656000	-1.753158000	-2.286255000
C	-4.735195000	-0.365045000	-1.573170000
C	-3.586041000	-1.987585000	-3.496744000
H	-1.974724000	-2.219022000	-2.094891000
C	-5.394877000	-0.584465000	-2.779347000
H	-5.214372000	0.262182000	-0.825126000
C	-4.821257000	-1.399793000	-3.751117000
H	-3.128409000	-2.630284000	-4.243618000
H	-6.361624000	-0.123556000	-2.960528000
H	-5.331942000	-1.576634000	-4.692369000
H	-2.012606000	-2.496142000	0.596312000

K4

	Coordinates (Angstroms)		
	X	Y	Z
B	2.915681000	-0.104011000	0.515542000
C	2.062278000	3.664067000	0.428272000
C	0.652958000	3.650921000	0.330374000
C	-0.044953000	2.416415000	0.240282000
C	2.116193000	1.244738000	0.424074000
C	2.777350000	2.497421000	0.458384000
C	-2.331796000	1.261146000	-0.041788000
C	-3.744301000	1.401819000	0.030498000
C	-4.493614000	0.244219000	-0.072093000
C	-2.543484000	-1.032033000	-0.472642000
O	-2.047649000	-2.120907000	-0.789710000
H	-5.567648000	0.300094000	0.022904000
N	-3.950810000	-0.951911000	-0.283221000
H	3.858641000	2.533606000	0.537001000
H	2.565598000	4.623520000	0.490405000
C	-1.497874000	2.439316000	0.149052000
C	-4.369776000	2.673100000	0.197398000
C	-2.162202000	3.675184000	0.247698000

C	-0.065731000	4.881127000	0.355320000
C	-1.418591000	4.890052000	0.336976000
C	-3.595487000	3.774322000	0.272496000
H	-5.451638000	2.730884000	0.243835000
H	-4.035735000	4.759570000	0.374033000
H	-1.966797000	5.824364000	0.386104000
H	0.493399000	5.809147000	0.413343000
O	0.384974000	-2.331481000	-1.748492000
H	0.160694000	-0.332507000	-2.049698000
N	-1.800631000	0.068653000	-0.303934000
C	0.732922000	1.234971000	0.316253000
H	0.218201000	0.288978000	0.283399000
C	0.857374000	-1.177286000	-2.173907000
O	1.950748000	-1.057476000	-2.667034000
C	-4.805655000	-2.206884000	-0.355739000
C	-4.710713000	-2.788370000	-1.769731000
C	-4.313024000	-3.187715000	0.713169000
C	-6.269967000	-1.875265000	-0.065255000
H	-5.028417000	-2.045795000	-2.507065000
H	-3.699865000	-3.114020000	-2.003343000
H	-5.383502000	-3.646557000	-1.839166000
H	-4.385119000	-2.733286000	1.705317000
H	-4.953511000	-4.072577000	0.695845000
H	-3.285854000	-3.497942000	0.535590000
H	-6.823403000	-2.815316000	-0.094243000
H	-6.411725000	-1.440767000	0.927934000
H	-6.709150000	-1.216041000	-0.818764000
H	-0.516207000	-2.209872000	-1.346104000
C	4.376441000	-0.180301000	-0.050615000
C	5.374804000	-0.922199000	0.599618000
C	4.734749000	0.494469000	-1.228448000
C	6.673741000	-0.977797000	0.107122000
H	5.132923000	-1.457761000	1.512309000
C	6.022547000	0.417959000	-1.743217000
H	3.983531000	1.066505000	-1.762649000
C	6.997594000	-0.312642000	-1.070658000
H	7.430861000	-1.547005000	0.635948000
H	6.268536000	0.930925000	-2.666893000
H	8.006193000	-0.366908000	-1.466720000
C	2.267686000	-1.360618000	1.191371000
C	2.590225000	-2.656575000	0.757286000
C	1.334096000	-1.243943000	2.235004000
C	1.998120000	-3.779317000	1.320730000
H	3.297546000	-2.784363000	-0.055154000
C	0.753421000	-2.363199000	2.817923000
H	1.064293000	-0.259666000	2.603351000
C	1.079405000	-3.634122000	2.354774000
H	2.248594000	-4.767622000	0.950670000
H	0.040280000	-2.246013000	3.626936000
H	0.617963000	-4.509688000	2.799242000

K5

	Coordinates (Angstroms)		
	X	Y	Z
B	3.000702000	-0.264723000	-0.278026000
C	2.208140000	3.489434000	-0.598525000
C	0.808673000	3.529495000	-0.416447000

C	0.078015000	2.330257000	-0.186655000
C	2.205622000	1.087952000	-0.308167000
C	2.889612000	2.304973000	-0.550849000
C	-2.262122000	1.265188000	0.089504000
C	-3.668089000	1.485572000	0.113660000
C	-4.477437000	0.367012000	0.177148000
C	-2.581015000	-1.047859000	0.333443000
O	-2.133031000	-2.179482000	0.517783000
H	-5.549520000	0.491328000	0.155886000
N	-3.996750000	-0.869383000	0.257487000
H	3.963236000	2.297888000	-0.704687000
H	2.730442000	4.423364000	-0.780279000
C	-1.373554000	2.413175000	-0.067228000
C	-4.234752000	2.791842000	0.044993000
C	-1.986441000	3.677989000	-0.145277000
C	0.139347000	4.785617000	-0.470086000
C	-1.203106000	4.856828000	-0.328529000
C	-3.410825000	3.851755000	-0.069065000
H	-5.312237000	2.907240000	0.086346000
H	-3.804014000	4.860253000	-0.124912000
H	-1.714780000	5.812064000	-0.370894000
H	0.728196000	5.682595000	-0.630670000
N	-1.788153000	0.028906000	0.198907000
C	0.829289000	1.127871000	-0.122323000
H	0.301200000	0.212363000	0.093818000
O	0.282439000	-2.270141000	1.890352000
H	-0.545176000	-2.199210000	1.383811000
H	1.805143000	-0.667395000	2.615130000
H	2.368052000	-0.204694000	2.786460000
C	-4.919410000	-2.073675000	0.302742000
C	-4.562329000	-3.005118000	-0.860403000
C	-4.767378000	-2.758251000	1.664754000
C	-6.377237000	-1.640408000	0.137553000
H	-4.652062000	-2.475016000	-1.812888000
H	-3.554141000	-3.401466000	-0.764255000
H	-5.268061000	-3.839161000	-0.867617000
H	-4.986543000	-2.051060000	2.469733000
H	-5.486620000	-3.578724000	1.726595000
H	-3.765296000	-3.157573000	1.803540000
H	-6.984892000	-2.546905000	0.143309000
H	-6.725529000	-1.010767000	0.960595000
H	-6.556306000	-1.131040000	-0.813230000
H	0.948392000	-2.401584000	1.211503000
C	2.336607000	-1.606204000	-0.745904000
C	2.776955000	-2.841813000	-0.236904000
C	1.276298000	-1.631871000	-1.667136000
C	2.172467000	-4.037463000	-0.606552000
H	3.588997000	-2.862941000	0.482275000
C	0.680555000	-2.825195000	-2.054925000
H	0.911743000	-0.702519000	-2.090043000
C	1.122019000	-4.029968000	-1.518447000
H	2.517270000	-4.972956000	-0.180035000
H	-0.136620000	-2.815491000	-2.767741000
H	0.646935000	-4.960981000	-1.808130000
C	4.501231000	-0.261368000	0.186144000
C	4.929420000	0.535972000	1.259522000
C	5.469075000	-1.042450000	-0.464486000

C	6.254499000	0.538780000	1.677285000
H	4.208755000	1.155740000	1.784133000
C	6.802949000	-1.019489000	-0.073375000
H	5.175119000	-1.670722000	-1.299501000
C	7.196276000	-0.234359000	1.005208000
H	6.555862000	1.150202000	2.521150000
H	7.533832000	-1.620156000	-0.604205000
H	8.234071000	-0.224073000	1.321052000

K6

	Coordinates (Angstroms)		
	X	Y	Z
B	2.766188000	-0.504942000	-0.418411000
C	2.037403000	3.270803000	0.440532000
C	0.627439000	3.309177000	0.486743000
C	-0.118670000	2.124355000	0.229404000
C	2.006342000	0.908318000	-0.124080000
C	2.703626000	2.109657000	0.141545000
C	-2.475406000	1.103542000	0.053987000
C	-3.880480000	1.309353000	0.072618000
C	-4.689592000	0.219208000	-0.188730000
C	-2.830570000	-1.138194000	-0.392755000
O	-2.364462000	-2.335048000	-0.591522000
H	-5.760708000	0.344419000	-0.202301000
N	-4.206528000	-1.003120000	-0.433310000
H	3.789298000	2.115331000	0.104026000
H	2.582523000	4.188708000	0.640782000
C	-1.575866000	2.208827000	0.275091000
C	-4.444962000	2.590992000	0.339024000
C	-2.182720000	3.455981000	0.548769000
C	-0.035396000	4.533609000	0.776923000
C	-1.388309000	4.609032000	0.803426000
C	-3.608271000	3.620915000	0.578216000
H	-5.521927000	2.710568000	0.347383000
H	-3.998000000	4.609829000	0.790966000
H	-1.890388000	5.546274000	1.015412000
H	0.567516000	5.414840000	0.972229000
H	-1.355974000	-2.356201000	-0.503285000
N	-2.022995000	-0.144302000	-0.170677000
C	0.620471000	0.955476000	-0.069351000
H	0.077787000	0.045185000	-0.257801000
H	2.076810000	-1.124154000	-1.253644000
O	0.160244000	-2.644829000	-0.343875000
H	0.817166000	-2.075477000	-0.784269000
C	-5.135831000	-2.184629000	-0.737599000
C	-4.800316000	-2.717502000	-2.133112000
C	-4.960824000	-3.237470000	0.359776000
C	-6.590000000	-1.715538000	-0.735407000
H	-4.888402000	-1.921543000	-2.877162000
H	-3.803131000	-3.148193000	-2.187221000
H	-5.523385000	-3.497003000	-2.381997000
H	-5.147378000	-2.796513000	1.342537000
H	-5.697704000	-4.026567000	0.195351000
H	-3.970275000	-3.686312000	0.351321000
H	-7.202925000	-2.586842000	-0.970510000
H	-6.910988000	-1.344873000	0.240973000
H	-6.787362000	-0.960994000	-1.500723000

H	0.482768000	-2.698219000	0.562952000
C	4.225815000	-0.274333000	-1.105110000
C	4.339954000	0.000761000	-2.476661000
C	5.422742000	-0.282783000	-0.375202000
C	5.564907000	0.252504000	-3.086218000
H	3.437672000	0.016370000	-3.084565000
C	6.657670000	-0.030224000	-0.969543000
H	5.389964000	-0.495787000	0.690448000
C	6.735174000	0.238462000	-2.331661000
H	5.610439000	0.456365000	-4.152032000
H	7.562187000	-0.046391000	-0.368478000
H	7.694516000	0.430031000	-2.801388000
C	2.866655000	-1.420938000	0.930321000
C	3.200771000	-2.783154000	0.833762000
C	2.654351000	-0.929986000	2.225138000
C	3.310033000	-3.604963000	1.950055000
H	3.373290000	-3.211954000	-0.150916000
C	2.765381000	-1.739528000	3.355640000
H	2.392056000	0.115892000	2.360722000
C	3.091863000	-3.083627000	3.223589000
H	3.563424000	-4.653925000	1.828802000
H	2.595310000	-1.316740000	4.341396000
H	3.175007000	-3.719044000	4.099359000

K7

	Coordinates (Angstroms)		
	X	Y	Z
B	2.778019000	-0.644622000	-0.465294000
C	2.102462000	3.172165000	0.290621000
C	0.694822000	3.218649000	0.369754000
C	-0.063327000	2.035648000	0.136037000
C	2.048647000	0.795540000	-0.213207000
C	2.756211000	2.000078000	0.004196000
C	-2.432113000	1.047885000	-0.029311000
C	-3.832430000	1.233747000	0.124733000
C	-4.655484000	0.147104000	-0.106957000
C	-2.828183000	-1.140574000	-0.638897000
O	-2.364876000	-2.307801000	-1.038867000
H	-5.722122000	0.251572000	0.015718000
N	-4.190611000	-1.052278000	-0.478350000
H	3.841104000	1.998076000	-0.050562000
H	2.656888000	4.089208000	0.467801000
C	-1.518648000	2.129402000	0.221789000
C	-4.378443000	2.494014000	0.505310000
C	-2.109421000	3.366997000	0.570484000
C	0.046635000	4.442247000	0.693042000
C	-1.302883000	4.515783000	0.800535000
C	-3.529010000	3.521594000	0.709153000
H	-5.450530000	2.602298000	0.617816000
H	-3.904577000	4.498865000	0.990458000
H	-1.792123000	5.448078000	1.058284000
H	0.658972000	5.322079000	0.863775000
H	-1.400090000	-2.235628000	-1.107521000
N	-2.004004000	-0.168162000	-0.428285000
C	0.662874000	0.856019000	-0.144492000
H	0.118644000	-0.058785000	-0.306197000
H	2.121194000	-1.244135000	-1.329325000

C	-5.131366000	-2.244735000	-0.711236000
C	-5.030308000	-2.658577000	-2.180863000
C	-4.741194000	-3.366021000	0.255095000
C	-6.570925000	-1.829858000	-0.415226000
H	-5.273522000	-1.815398000	-2.832439000
H	-4.045062000	-3.039699000	-2.440919000
H	-5.760305000	-3.450360000	-2.361290000
H	-4.764204000	-3.004791000	1.286385000
H	-5.478167000	-4.165888000	0.158033000
H	-3.759001000	-3.782928000	0.044338000
H	-7.193473000	-2.708326000	-0.590214000
H	-6.714016000	-1.529302000	0.625527000
H	-6.926684000	-1.041505000	-1.081984000
C	2.751959000	-1.555001000	0.891204000
C	2.940077000	-2.945117000	0.814507000
C	2.585178000	-1.029420000	2.179809000
C	2.962153000	-3.759455000	1.941544000
H	3.075734000	-3.401923000	-0.163642000
C	2.605698000	-1.831148000	3.320776000
H	2.431247000	0.039850000	2.301592000
C	2.793554000	-3.203546000	3.207647000
H	3.109825000	-4.830167000	1.834335000
H	2.473177000	-1.380860000	4.300332000
H	2.807916000	-3.832996000	4.091705000
C	4.294729000	-0.454562000	-1.037093000
C	5.437411000	-0.509572000	-0.226246000
C	4.516461000	-0.165829000	-2.393048000
C	6.718900000	-0.289254000	-0.728479000
H	5.323883000	-0.733041000	0.831802000
C	5.788433000	0.054665000	-2.911672000
H	3.661493000	-0.114772000	-3.064233000
C	6.901808000	-0.005795000	-2.077344000
H	7.576737000	-0.341133000	-0.064171000
H	5.915339000	0.270043000	-3.968678000
H	7.897268000	0.161715000	-2.475866000

K8

	Coordinates (Angstroms)		
	X	Y	Z
B	2.697408000	-0.321532000	0.149147000
C	1.803978000	3.464289000	-0.272135000
C	0.393390000	3.455464000	-0.359908000
C	-0.308071000	2.224842000	-0.261393000
C	1.853969000	1.053570000	-0.022166000
C	2.510338000	2.305780000	-0.083947000
C	-2.609508000	1.078642000	-0.091069000
C	-4.022944000	1.232372000	-0.125756000
C	-4.780820000	0.103221000	0.120289000
C	-2.830195000	-1.218327000	0.340636000
O	-2.316163000	-2.333108000	0.502876000
H	-5.857380000	0.183219000	0.124772000
N	-4.246557000	-1.093367000	0.362022000
H	3.590410000	2.353792000	0.010844000
H	2.317561000	4.418164000	-0.345513000
C	-1.763374000	2.247230000	-0.293291000
C	-4.637087000	2.498197000	-0.367541000
C	-2.418635000	3.477533000	-0.491550000

C	-0.316775000	4.673902000	-0.551372000
C	-1.669183000	4.682092000	-0.632458000
C	-3.852352000	3.580079000	-0.542829000
H	-5.718952000	2.567195000	-0.397711000
H	-4.285668000	4.558374000	-0.717230000
H	-2.209098000	5.609636000	-0.787717000
H	0.246921000	5.597491000	-0.636167000
O	1.622490000	-1.347320000	0.936693000
N	-2.087885000	-0.122082000	0.143628000
C	0.475131000	1.048549000	-0.138618000
H	-0.053237000	0.110433000	-0.148199000
C	1.099768000	-2.634253000	0.455800000
H	0.714860000	-3.123556000	1.354373000
H	1.979735000	-3.146299000	0.075746000
O	0.171442000	-2.493616000	-0.524690000
H	-0.723042000	-2.398348000	-0.127179000
C	-5.117838000	-2.304072000	0.648401000
C	-4.901268000	-3.339369000	-0.459612000
C	-4.753256000	-2.849559000	2.032974000
C	-6.595953000	-1.912035000	0.661941000
H	-5.125270000	-2.901263000	-1.436240000
H	-3.881211000	-3.716702000	-0.464231000
H	-5.585908000	-4.174896000	-0.295234000
H	-4.888898000	-2.075526000	2.793773000
H	-5.423449000	-3.679596000	2.268596000
H	-3.727122000	-3.208663000	2.067391000
H	-7.164929000	-2.814021000	0.893225000
H	-6.826081000	-1.172641000	1.433498000
H	-6.943564000	-1.547234000	-0.307905000
H	0.921705000	-0.854164000	1.384347000
C	3.928101000	-0.160082000	1.186358000
C	5.172918000	0.300543000	0.731909000
C	3.806363000	-0.378797000	2.563842000
C	6.233926000	0.537198000	1.600064000
H	5.319210000	0.474882000	-0.330534000
C	4.862715000	-0.153378000	3.442446000
H	2.866935000	-0.741299000	2.969202000
C	6.082951000	0.310090000	2.964091000
H	7.181247000	0.895995000	1.209993000
H	4.730352000	-0.337946000	4.503950000
H	6.907366000	0.489632000	3.646189000
C	3.083920000	-1.054410000	-1.238881000
C	2.458944000	-0.732906000	-2.449308000
C	4.057885000	-2.062745000	-1.279367000
C	2.782573000	-1.380014000	-3.637973000
H	1.697339000	0.040179000	-2.465522000
C	4.385785000	-2.723389000	-2.458764000
H	4.574906000	-2.337503000	-0.364239000
C	3.747065000	-2.381290000	-3.646415000
H	2.279039000	-1.104244000	-4.559086000
H	5.142245000	-3.501641000	-2.452796000
H	4.001107000	-2.889108000	-4.571092000

TSK2-3

	Coordinates (Angstroms)		
	X	Y	Z
B	2.274541000	-0.250157000	-0.176751000

C	1.427328000	3.605581000	0.429276000
C	0.059339000	3.612400000	0.049195000
C	-0.463527000	2.430774000	-0.514495000
C	1.664218000	1.272946000	-0.292977000
C	2.162586000	2.440766000	0.353946000
C	-2.431780000	0.916980000	-0.434679000
C	-3.822213000	0.690242000	-0.344685000
C	-4.201503000	-0.641368000	-0.263636000
C	-1.964454000	-1.343430000	-0.248597000
O	-1.053643000	-2.245283000	-0.171866000
H	-5.248008000	-0.898264000	-0.209028000
N	-3.321834000	-1.664559000	-0.241809000
H	3.155789000	2.421496000	0.795610000
H	1.859231000	4.503929000	0.860140000
C	-1.888520000	2.242543000	-0.507680000
C	-4.707124000	1.819488000	-0.282319000
C	-2.757290000	3.307113000	-0.254663000
C	-0.840338000	4.701593000	0.267051000
C	-2.186654000	4.577343000	0.060849000
C	-4.181346000	3.068727000	-0.216088000
H	-5.778492000	1.658792000	-0.244359000
H	-4.837738000	3.925370000	-0.105969000
H	-2.846792000	5.420741000	0.232523000
H	-0.437305000	5.643770000	0.626235000
O	1.157512000	-1.295479000	-0.644213000
H	0.001174000	-1.742854000	-0.333433000
N	-1.592774000	-0.101915000	-0.330606000
C	0.419547000	1.388845000	-0.860554000
H	0.058535000	0.568919000	-1.449468000
C	-3.784718000	-3.116487000	-0.189568000
C	-3.241318000	-3.830992000	-1.429814000
C	-3.273860000	-3.736413000	1.113465000
C	-5.310000000	-3.195145000	-0.208481000
H	-3.598361000	-3.340096000	-2.339105000
H	-2.153424000	-3.858980000	-1.441370000
H	-3.612527000	-4.858213000	-1.427567000
H	-3.642579000	-3.171319000	1.973726000
H	-3.657502000	-4.756591000	1.185992000
H	-2.186806000	-3.772778000	1.149612000
H	-5.574716000	-4.253626000	-0.191628000
H	-5.764539000	-2.731301000	0.670358000
H	-5.739412000	-2.763174000	-1.115854000
C	3.565176000	-0.557468000	-1.112419000
C	4.212510000	-1.801892000	-1.042313000
C	4.079741000	0.366242000	-2.029015000
C	5.308024000	-2.110330000	-1.840632000
H	3.852920000	-2.545258000	-0.334928000
C	5.180975000	0.073374000	-2.831835000
H	3.608149000	1.341039000	-2.119444000
C	5.799089000	-1.168142000	-2.740896000
H	5.785628000	-3.081663000	-1.757239000
H	5.554681000	0.815209000	-3.530936000
H	6.656841000	-1.400954000	-3.363511000
C	2.535745000	-0.528493000	1.404215000
C	1.490342000	-0.927008000	2.248637000
C	3.781441000	-0.313822000	2.007396000
C	1.674379000	-1.112096000	3.614707000

H	0.504179000	-1.098615000	1.826788000
C	3.979625000	-0.490895000	3.374822000
H	4.623381000	-0.006732000	1.392295000
C	2.924833000	-0.894252000	4.186252000
H	0.841539000	-1.425942000	4.236911000
H	4.960644000	-0.319044000	3.807315000
H	3.075144000	-1.038904000	5.251098000
H	1.477445000	-1.773099000	-1.412261000

TSK3-4

	Coordinates (Angstroms)		
	X	Y	Z
B	2.581285000	-0.384226000	0.187926000
C	1.771899000	3.454595000	-0.334721000
C	0.356339000	3.459830000	-0.375341000
C	-0.340915000	2.234570000	-0.226934000
C	1.814060000	1.045124000	-0.016318000
C	2.476743000	2.291114000	-0.148801000
C	-2.615815000	1.073568000	-0.042608000
C	-4.029653000	1.179883000	-0.062866000
C	-4.745984000	0.010885000	0.126656000
C	-2.771873000	-1.216883000	0.349515000
O	-2.182479000	-2.331178000	0.545154000
H	-5.824734000	0.042469000	0.114304000
N	-4.167486000	-1.180206000	0.323940000
H	3.561384000	2.332732000	-0.105659000
H	2.291611000	4.401559000	-0.447252000
C	-1.793989000	2.247056000	-0.235886000
C	-4.671392000	2.439910000	-0.268568000
C	-2.468959000	3.468456000	-0.421979000
C	-0.370337000	4.669901000	-0.560719000
C	-1.728426000	4.674245000	-0.590733000
C	-3.905646000	3.538709000	-0.439433000
H	-5.754160000	2.491494000	-0.281019000
H	-4.363207000	4.509643000	-0.592353000
H	-2.274422000	5.599816000	-0.736121000
H	0.182044000	5.596698000	-0.680050000
O	1.504462000	-1.455113000	0.642864000
H	-0.999512000	-2.257763000	0.250985000
N	-2.066857000	-0.125523000	0.171534000
C	0.437333000	1.065793000	-0.073052000
H	-0.090685000	0.139714000	0.016306000
C	0.245314000	-2.306941000	-0.341979000
O	0.697627000	-2.732983000	-1.306942000
C	-4.995935000	-2.449949000	0.503056000
C	-4.641337000	-3.419840000	-0.627772000
C	-4.703030000	-3.028523000	1.889610000
C	-6.486943000	-2.127136000	0.415876000
H	-4.813869000	-2.949634000	-1.599673000
H	-3.607956000	-3.754564000	-0.571116000
H	-5.293370000	-4.292593000	-0.550609000
H	-4.941547000	-2.295486000	2.664959000
H	-5.339574000	-3.903333000	2.039266000
H	-3.664765000	-3.335568000	1.994858000
H	-7.023427000	-3.067792000	0.548711000
H	-6.816350000	-1.448140000	1.206390000
H	-6.771212000	-1.722717000	-0.558732000

C	3.233061000	-0.961402000	-1.185401000
C	3.997256000	-2.137770000	-1.160735000
C	3.067452000	-0.348371000	-2.430557000
C	4.552423000	-2.681147000	-2.312114000
H	4.156986000	-2.642636000	-0.211517000
C	3.625511000	-0.876714000	-3.594234000
H	2.483892000	0.565195000	-2.502222000
C	4.368290000	-2.048949000	-3.540148000
H	5.131701000	-3.597848000	-2.256184000
H	3.475895000	-0.372682000	-4.544276000
H	4.802830000	-2.466951000	-4.442466000
C	3.687837000	-0.259048000	1.380071000
C	3.411556000	-0.550414000	2.721952000
C	4.976086000	0.222834000	1.102692000
C	4.357844000	-0.374733000	3.728752000
H	2.434302000	-0.932838000	3.005837000
C	5.929518000	0.410547000	2.098117000
H	5.244534000	0.448058000	0.073937000
C	5.623959000	0.110359000	3.421938000
H	4.105537000	-0.616971000	4.756669000
H	6.915176000	0.785789000	1.840175000
H	6.364761000	0.248147000	4.202652000
H	1.031760000	-1.145732000	1.423625000

TSK1-7

	Coordinates (Angstroms)		
	X	Y	Z
B	-2.306807000	0.033569000	0.060187000
C	-1.112975000	3.796703000	-0.426946000
C	0.218415000	3.672659000	0.045726000
C	0.608922000	2.428608000	0.579933000
C	-1.585684000	1.473582000	0.182131000
C	-1.939680000	2.690931000	-0.461407000
C	2.401257000	0.736650000	0.372372000
C	3.758792000	0.384186000	0.212194000
C	3.991931000	-0.948687000	-0.089338000
C	1.681086000	-1.399939000	-0.183940000
O	0.693263000	-2.155372000	-0.446142000
H	5.004318000	-1.303646000	-0.208847000
N	3.007103000	-1.850757000	-0.270773000
H	-2.896926000	2.764303000	-0.969934000
H	-1.441657000	4.744910000	-0.841084000
C	2.007813000	2.101465000	0.593884000
C	4.759252000	1.412508000	0.277574000
C	2.979383000	3.087827000	0.425976000
C	1.221600000	4.688737000	-0.063848000
C	2.538916000	4.427787000	0.187732000
C	4.372019000	2.710491000	0.359764000
H	5.807441000	1.145438000	0.204336000
H	5.114907000	3.500410000	0.335668000
H	3.280027000	5.214291000	0.094437000
H	0.921753000	5.683333000	-0.379890000
H	-0.406410000	-1.400313000	-0.357565000
N	1.452098000	-0.160422000	0.162118000
C	-0.375299000	1.455969000	0.836287000
H	-0.123082000	0.597253000	1.434076000
H	-1.192028000	-0.851029000	-0.329739000

C	3.310729000	-3.305375000	-0.603547000
C	2.659926000	-4.183700000	0.468538000
C	2.770996000	-3.600784000	-2.005474000
C	4.816859000	-3.557948000	-0.592432000
H	3.055278000	-3.933702000	1.456775000
H	1.576885000	-4.077570000	0.479583000
H	2.902182000	-5.227525000	0.257007000
H	3.232388000	-2.933651000	-2.738741000
H	3.027847000	-4.629675000	-2.267755000
H	1.689478000	-3.490153000	-2.054807000
H	4.965082000	-4.618026000	-0.804679000
H	5.343246000	-2.994216000	-1.366699000
H	5.267964000	-3.352362000	0.381787000
C	-3.261603000	-0.140631000	-1.217403000
C	-4.630133000	-0.403591000	-1.088730000
C	-2.765448000	0.030018000	-2.518392000
C	-5.467090000	-0.486524000	-2.199998000
H	-5.055386000	-0.545790000	-0.099354000
C	-3.588090000	-0.059647000	-3.634531000
H	-1.706028000	0.231327000	-2.656917000
C	-4.948053000	-0.316417000	-3.477755000
H	-6.525327000	-0.687343000	-2.065767000
H	-3.170910000	0.071337000	-4.628043000
H	-5.595824000	-0.384472000	-4.345589000
C	-2.786023000	-0.652133000	1.427020000
C	-3.126940000	0.115100000	2.546801000
C	-2.938816000	-2.041763000	1.537917000
C	-3.604788000	-0.468643000	3.718384000
H	-3.015999000	1.194950000	2.502760000
C	-3.407435000	-2.636592000	2.702633000
H	-2.679738000	-2.670261000	0.689468000
C	-3.745779000	-1.848462000	3.800661000
H	-3.864323000	0.155504000	4.567759000
H	-3.511577000	-3.715637000	2.758339000
H	-4.114407000	-2.308617000	4.711485000

TSK5-6

	Coordinates (Angstroms)		
	X	Y	Z
B	2.733583000	-0.376971000	0.203878000
C	1.886450000	3.379471000	-0.503107000
C	0.471872000	3.379231000	-0.514929000
C	-0.228892000	2.165967000	-0.284581000
C	1.930932000	1.003075000	-0.027111000
C	2.596185000	2.229788000	-0.262340000
C	-2.513398000	1.006669000	-0.087636000
C	-3.926545000	1.147337000	-0.041422000
C	-4.656588000	0.000961000	0.212646000
C	-2.687333000	-1.305076000	0.270572000
O	-2.139028000	-2.414626000	0.377374000
H	-5.731808000	0.066147000	0.282908000
N	-4.096897000	-1.198146000	0.388337000
H	3.682074000	2.265000000	-0.247971000
H	2.401153000	4.319360000	-0.679349000
C	-1.683100000	2.183609000	-0.288312000
C	-4.556339000	2.414953000	-0.234819000
C	-2.350160000	3.404967000	-0.491648000

C	-0.250090000	4.585536000	-0.741455000
C	-1.606298000	4.599396000	-0.724499000
C	-3.786103000	3.498511000	-0.464317000
H	-5.637976000	2.483068000	-0.196114000
H	-4.234501000	4.472932000	-0.622008000
H	-2.151420000	5.522912000	-0.886640000
H	0.306462000	5.500215000	-0.920077000
N	-1.974024000	-0.199965000	0.051176000
C	0.551307000	1.010399000	-0.044708000
H	0.025207000	0.089956000	0.125455000
O	0.469710000	-2.443954000	-0.072060000
H	-0.504241000	-2.358747000	0.150425000
H	1.327754000	-1.603429000	0.463629000
H	1.902188000	-1.094900000	0.986349000
C	-4.936240000	-2.423792000	0.707667000
C	-4.783479000	-3.433186000	-0.433734000
C	-4.471352000	-2.992341000	2.052435000
C	-6.412571000	-2.046818000	0.830653000
H	-5.104115000	-2.987575000	-1.379544000
H	-3.756183000	-3.777721000	-0.530955000
H	-5.424086000	-4.293815000	-0.226980000
H	-4.548646000	-2.230806000	2.833476000
H	-5.124173000	-3.825632000	2.322765000
H	-3.446559000	-3.353431000	2.005742000
H	-6.957635000	-2.959649000	1.076577000
H	-6.593993000	-1.329264000	1.635284000
H	-6.827922000	-1.660636000	-0.103623000
H	0.552518000	-2.363858000	-1.030028000
C	3.075750000	-1.198476000	-1.144632000
C	3.610195000	-2.494810000	-1.078601000
C	2.862766000	-0.666751000	-2.421031000
C	3.916749000	-3.222146000	-2.221985000
H	3.786807000	-2.944953000	-0.105148000
C	3.174702000	-1.382359000	-3.575914000
H	2.441968000	0.329470000	-2.520609000
C	3.702334000	-2.663830000	-3.479981000
H	4.326892000	-4.223229000	-2.134507000
H	3.003486000	-0.936754000	-4.550705000
H	3.944643000	-3.224874000	-4.376558000
C	3.946131000	-0.249363000	1.266014000
C	3.690422000	0.013976000	2.619456000
C	5.289173000	-0.321011000	0.878426000
C	4.715215000	0.186180000	3.541937000
H	2.659486000	0.085538000	2.958753000
C	6.326148000	-0.140700000	1.791980000
H	5.533405000	-0.523876000	-0.160222000
C	6.043144000	0.109983000	3.129056000
H	4.481973000	0.380701000	4.584028000
H	7.357124000	-0.201611000	1.457539000
H	6.847262000	0.243628000	3.845138000

TSK7

	Coordinates (Angstroms)		
	X	Y	Z
B	2.726754000	-0.224012000	0.101922000
C	1.812370000	3.565547000	-0.336224000
C	0.398462000	3.541419000	-0.317896000

C	-0.281334000	2.305872000	-0.140024000
C	1.903218000	1.161439000	-0.030258000
C	2.542248000	2.413650000	-0.194244000
C	-2.559609000	1.123686000	0.059485000
C	-3.974018000	1.236619000	-0.022093000
C	-4.704815000	0.070753000	0.104562000
C	-2.742060000	-1.170505000	0.464162000
O	-2.211794000	-2.275272000	0.732070000
H	-5.780177000	0.108901000	0.021097000
N	-4.143384000	-1.120012000	0.320297000
H	3.627752000	2.465080000	-0.208572000
H	2.307650000	4.523153000	-0.467703000
C	-1.737521000	2.306079000	-0.131960000
C	-4.616635000	2.492959000	-0.238766000
C	-2.418935000	3.524087000	-0.327759000
C	-0.338099000	4.746697000	-0.487712000
C	-1.693828000	4.738602000	-0.498643000
C	-3.854279000	3.596471000	-0.372992000
H	-5.698986000	2.535471000	-0.288062000
H	-4.308434000	4.568344000	-0.529614000
H	-2.252172000	5.657461000	-0.639894000
H	0.207253000	5.676176000	-0.618078000
H	-0.846560000	-2.350210000	0.920315000
N	-2.018779000	-0.070182000	0.314013000
C	0.521026000	1.147174000	-0.003179000
H	0.020350000	0.204533000	0.126772000
H	1.841725000	-1.048316000	0.528926000
O	0.556061000	-2.288133000	-0.983430000
C	1.011659000	-2.489699000	0.230091000
H	1.981855000	-2.963391000	0.360954000
O	0.171214000	-2.588805000	1.172690000
C	-4.984183000	-2.386382000	0.412485000
C	-4.520289000	-3.355744000	-0.679387000
C	-4.835805000	-2.973248000	1.819011000
C	-6.460049000	-2.066273000	0.173124000
H	-4.614837000	-2.889912000	-1.664264000
H	-3.490886000	-3.673304000	-0.531461000
H	-5.164450000	-4.237750000	-0.656634000
H	-5.146850000	-2.241509000	2.569643000
H	-5.488411000	-3.845511000	1.901804000
H	-3.813044000	-3.281313000	2.022934000
H	-7.004404000	-3.010279000	0.229898000
H	-6.876091000	-1.404024000	0.936784000
H	-6.640632000	-1.641879000	-0.817951000
H	1.301357000	-2.142803000	-1.591301000
C	3.856906000	-0.184278000	1.256633000
C	3.534432000	-0.440108000	2.596860000
C	5.188270000	0.166409000	0.988871000
C	4.479812000	-0.353484000	3.614445000
H	2.512039000	-0.713478000	2.847869000
C	6.143370000	0.262077000	1.997341000
H	5.487674000	0.364895000	-0.037143000
C	5.792616000	0.000071000	3.317893000
H	4.193996000	-0.561981000	4.640974000
H	7.165052000	0.536740000	1.752854000
H	6.534808000	0.068429000	4.106471000
C	3.210794000	-0.846145000	-1.315522000

C	2.714772000	-0.392725000	-2.548735000
C	4.067588000	-1.960928000	-1.362657000
C	3.052368000	-1.005721000	-3.754157000
H	2.042939000	0.460706000	-2.566187000
C	4.408500000	-2.584003000	-2.558607000
H	4.482049000	-2.347459000	-0.435343000
C	3.901068000	-2.106437000	-3.763756000
H	2.650078000	-0.621463000	-4.686210000
H	5.073680000	-3.441875000	-2.552274000
H	4.165096000	-2.587932000	-4.699384000

TSK8

	Coordinates (Angstroms)		
	X	Y	Z
B	2.675530000	-0.402540000	0.280257000
C	1.847716000	3.273294000	-0.882151000
C	0.437523000	3.323721000	-0.800879000
C	-0.284095000	2.154618000	-0.439859000
C	1.857836000	0.954634000	-0.151141000
C	2.531469000	2.128885000	-0.569955000
C	-2.614075000	1.120164000	-0.135909000
C	-4.009049000	1.327866000	0.023897000
C	-4.784234000	0.219676000	0.310630000
C	-2.923057000	-1.158048000	0.141343000
O	-2.449294000	-2.356165000	0.132833000
H	-5.843784000	0.339388000	0.475254000
N	-4.281375000	-1.016678000	0.394158000
H	3.615367000	2.132395000	-0.629014000
H	2.378529000	4.173227000	-1.178725000
C	-1.736756000	2.240746000	-0.375162000
C	-4.589426000	2.624103000	-0.114161000
C	-2.363137000	3.490956000	-0.569898000
C	-0.245488000	4.546417000	-1.048447000
C	-1.592326000	4.636345000	-0.914622000
C	-3.782941000	3.658848000	-0.427101000
H	-5.658067000	2.748527000	0.016275000
H	-4.191182000	4.653360000	-0.567324000
H	-2.105515000	5.577870000	-1.074557000
H	0.338265000	5.418587000	-1.325759000
O	1.726260000	-1.377843000	0.974466000
N	-2.144139000	-0.135372000	-0.084926000
C	0.474617000	0.999350000	-0.119618000
H	-0.059037000	0.105658000	0.154862000
C	0.698348000	-2.939249000	0.066423000
H	0.431842000	-3.259284000	1.079961000
H	1.669085000	-3.260650000	-0.313917000
O	-0.167225000	-2.542826000	-0.723087000
H	-1.432987000	-2.380738000	-0.215608000
C	-5.167885000	-2.217618000	0.731418000
C	-5.186052000	-3.164715000	-0.471305000
C	-4.622065000	-2.878024000	2.000491000
C	-6.597019000	-1.752309000	1.007120000
H	-5.521598000	-2.635907000	-1.367414000
H	-4.211300000	-3.607705000	-0.661758000
H	-5.897228000	-3.967470000	-0.264162000
H	-4.581331000	-2.153295000	2.817984000
H	-5.304918000	-3.679625000	2.289881000

H	-3.633673000	-3.307135000	1.852520000
H	-7.174369000	-2.640719000	1.267224000
H	-6.656401000	-1.062829000	1.852948000
H	-7.068684000	-1.302039000	0.130418000
H	1.137926000	-0.920416000	1.579912000
C	3.240830000	-1.207909000	-1.026862000
C	4.239842000	-2.184189000	-0.895563000
C	2.695745000	-1.044668000	-2.305986000
C	4.666433000	-2.957955000	-1.970686000
H	4.696697000	-2.344899000	0.077505000
C	3.114227000	-1.807879000	-3.393699000
H	1.913913000	-0.305822000	-2.457455000
C	4.102566000	-2.771830000	-3.229769000
H	5.440330000	-3.706499000	-1.828766000
H	2.664887000	-1.652122000	-4.369916000
H	4.432547000	-3.370017000	-4.073098000
C	3.877456000	0.000491000	1.322425000
C	3.711180000	-0.098704000	2.709838000
C	5.112512000	0.503757000	0.884904000
C	4.700857000	0.290251000	3.609671000
H	2.782743000	-0.501405000	3.105827000
C	6.111123000	0.899701000	1.769655000
H	5.305489000	0.578997000	-0.182288000
C	5.908914000	0.796455000	3.142666000
H	4.530130000	0.193616000	4.677886000
H	7.051779000	1.284629000	1.387039000
H	6.684026000	1.100964000	3.838398000

TSK9

	Coordinates (Angstroms)		
	X	Y	Z
B	2.761526000	-0.404145000	-0.142953000
C	1.988308000	3.439142000	0.224491000
C	0.578148000	3.451641000	0.280503000
C	-0.141776000	2.229889000	0.161494000
C	2.004560000	1.023809000	-0.013753000
C	2.678798000	2.263252000	0.067565000
C	-2.465592000	1.149508000	-0.019159000
C	-3.869831000	1.295120000	0.129466000
C	-4.647617000	0.163876000	-0.034431000
C	-2.764271000	-1.083614000	-0.547249000
O	-2.287348000	-2.229428000	-0.911833000
H	-5.715737000	0.229280000	0.099773000
N	-4.135851000	-1.029381000	-0.348830000
H	3.763646000	2.285322000	0.014942000
H	2.514565000	4.385058000	0.313081000
C	-1.598079000	2.281347000	0.197027000
C	-4.463641000	2.555692000	0.433003000
C	-2.233530000	3.518924000	0.440362000
C	-0.114834000	4.678634000	0.477604000
C	-1.466460000	4.710952000	0.575667000
C	-3.659266000	3.630705000	0.560766000
H	-5.539520000	2.627076000	0.541699000
H	-4.074797000	4.610247000	0.768338000
H	-1.989253000	5.644727000	0.748597000
H	0.464057000	5.592613000	0.565183000
H	-1.264095000	-2.228675000	-1.040453000

N	-1.984238000	-0.053396000	-0.386059000
C	0.619351000	1.045817000	0.044677000
H	0.099724000	0.103825000	-0.007543000
H	2.048676000	-1.075605000	-0.941690000
C	0.901406000	-1.843081000	-1.991481000
H	0.589515000	-0.837667000	-2.304673000
O	0.143405000	-2.582750000	-1.355980000
H	1.784273000	-2.263424000	-2.487415000
C	-5.027690000	-2.269185000	-0.483358000
C	-4.950091000	-2.775462000	-1.925955000
C	-4.562792000	-3.308725000	0.539961000
C	-6.479206000	-1.902924000	-0.175523000
H	-5.231066000	-1.982113000	-2.623686000
H	-3.960463000	-3.144762000	-2.183671000
H	-5.663521000	-3.594726000	-2.038300000
H	-4.593322000	-2.888043000	1.548455000
H	-5.251169000	-4.155718000	0.503519000
H	-3.559367000	-3.674777000	0.335723000
H	-7.061898000	-2.820791000	-0.265425000
H	-6.611286000	-1.530033000	0.843204000
H	-6.891149000	-1.184480000	-0.888640000
C	4.217185000	-0.293749000	-0.848396000
C	4.321696000	0.100668000	-2.192545000
C	5.421953000	-0.545038000	-0.179626000
C	5.548006000	0.237915000	-2.832880000
H	3.410770000	0.309670000	-2.750083000
C	6.659493000	-0.407807000	-0.806405000
H	5.394361000	-0.856212000	0.861396000
C	6.728527000	-0.016552000	-2.138492000
H	5.587335000	0.542517000	-3.874530000
H	7.572352000	-0.608906000	-0.253609000
H	7.689422000	0.089803000	-2.631322000
C	2.749508000	-1.261461000	1.231443000
C	2.914384000	-2.655320000	1.197026000
C	2.595219000	-0.684830000	2.498779000
C	2.931198000	-3.429292000	2.352074000
H	3.031166000	-3.145806000	0.233351000
C	2.613246000	-1.446581000	3.665998000
H	2.454779000	0.390052000	2.579968000
C	2.780777000	-2.825006000	3.597789000
H	3.059233000	-4.505382000	2.282477000
H	2.492937000	-0.962386000	4.630585000
H	2.791824000	-3.422680000	4.503598000

L1

	Coordinates (Angstroms)		
	X	Y	Z
B	-1.963251000	0.010441000	0.252242000
C	-3.503501000	-0.189574000	0.008889000
C	-4.248010000	-1.078850000	0.773930000
C	-4.185607000	0.471146000	-1.005609000
C	-5.598472000	-1.294200000	0.564356000
C	-5.529505000	0.266487000	-1.255096000
C	-6.238874000	-0.619881000	-0.461100000
C	-1.061617000	-1.277948000	0.376986000
C	-1.096930000	-2.262026000	-0.602722000
C	-0.141149000	-1.466894000	1.402567000

C	-0.253583000	-3.358196000	-0.592157000
C	0.708293000	-2.555014000	1.448032000
C	0.654237000	-3.500609000	0.440157000
F	-3.669260000	-1.745387000	1.778745000
F	-6.282981000	-2.138713000	1.332625000
F	-7.530967000	-0.821347000	-0.683373000
F	-6.145829000	0.906157000	-2.246427000
F	-3.535248000	1.322852000	-1.803784000
F	-0.057523000	-0.578558000	2.399931000
F	1.600578000	-2.685338000	2.428718000
F	1.490603000	-4.529651000	0.453345000
F	-0.294142000	-4.260839000	-1.570854000
F	-1.941964000	-2.148942000	-1.635897000
C	-1.242649000	3.801026000	0.497318000
C	0.147810000	3.851494000	0.280906000
C	0.853088000	2.637947000	0.069879000
C	-1.270217000	1.397805000	0.294411000
C	-1.928021000	2.621620000	0.515789000
C	3.140799000	1.505114000	-0.274837000
C	4.550533000	1.682645000	-0.308750000
C	5.319682000	0.531667000	-0.362325000
C	3.365602000	-0.820648000	-0.549779000
O	2.880847000	-1.922388000	-0.755978000
H	6.396162000	0.616735000	-0.329526000
N	4.793861000	-0.684689000	-0.438157000
C	5.663333000	-1.926712000	-0.428044000
C	2.296103000	2.683995000	-0.093620000
C	5.152968000	2.973424000	-0.249603000
C	2.937490000	3.931882000	-0.043172000
C	0.831302000	5.099600000	0.290884000
C	2.174793000	5.127741000	0.130711000
C	4.364471000	4.063016000	-0.140068000
H	6.233244000	3.056532000	-0.298157000
H	4.789994000	5.058799000	-0.098747000
H	2.704975000	6.073542000	0.145734000
H	0.271888000	6.014879000	0.435483000
N	2.618181000	0.293181000	-0.403031000
C	0.099774000	1.445288000	0.074254000
H	0.631397000	0.527457000	-0.111405000
C	5.218916000	-2.817521000	0.737930000
C	5.532404000	-2.633269000	-1.780922000
C	7.131324000	-1.555224000	-0.213687000
H	6.212026000	-3.488983000	-1.797161000
H	4.517528000	-2.985441000	-1.950340000
H	5.817985000	-1.955453000	-2.590481000
H	5.300223000	-2.274324000	1.683796000
H	4.196315000	-3.165874000	0.613813000
H	5.881171000	-3.685221000	0.784765000
H	7.697257000	-2.487482000	-0.173732000
H	7.540147000	-0.962924000	-1.036732000
H	7.295219000	-1.030560000	0.731542000
F	-3.244516000	2.659569000	0.767601000
F	-1.907538000	4.947284000	0.711928000

L1_CO

Coordinates (Angstroms)

	X	Y	Z
B	-1.585297000	-0.201751000	-0.168482000
C	-2.247345000	-1.424455000	0.686111000
C	-2.919817000	-2.444877000	0.024960000
C	-2.209811000	-1.534212000	2.069274000
C	-3.537564000	-3.500417000	0.665941000
C	-2.820728000	-2.576241000	2.750293000
C	-3.486499000	-3.563306000	2.047733000
C	-2.613282000	0.584074000	-1.164039000
C	-3.974689000	0.720127000	-0.930683000
C	-2.128255000	1.260279000	-2.274091000
C	-4.800608000	1.475320000	-1.747437000
C	-2.917909000	2.022425000	-3.114369000
C	-4.270899000	2.127698000	-2.846218000
F	-2.980984000	-2.420243000	-1.318871000
F	-4.169974000	-4.447197000	-0.023421000
F	-4.071033000	-4.565852000	2.692835000
F	-2.769022000	-2.633826000	4.079959000
F	-1.587899000	-0.623751000	2.823171000
F	-0.816763000	1.182997000	-2.567733000
F	-2.394154000	2.646518000	-4.167317000
F	-5.055084000	2.853282000	-3.635692000
F	-6.100981000	1.582320000	-1.479505000
F	-4.553616000	0.137080000	0.124436000
C	-0.432164000	2.797057000	2.001864000
C	0.971879000	2.764762000	1.940064000
C	1.598247000	1.723955000	1.205640000
C	-0.622483000	0.833356000	0.618581000
C	-1.199961000	1.869012000	1.362348000
C	3.828775000	0.681765000	0.439799000
C	5.247184000	0.786940000	0.443641000
C	5.945851000	-0.182609000	-0.253357000
C	3.917019000	-1.283267000	-0.859160000
O	3.362189000	-2.214614000	-1.420311000
H	7.024443000	-0.132276000	-0.282650000
N	5.353476000	-1.181870000	-0.896947000
C	6.161514000	-2.214663000	-1.657788000
C	3.051178000	1.696787000	1.148339000
C	5.928225000	1.838364000	1.124752000
C	3.771552000	2.706093000	1.811572000
C	1.738024000	3.761379000	2.606707000
C	3.088434000	3.727944000	2.537062000
C	5.207466000	2.763218000	1.790232000
H	7.012012000	1.873082000	1.094726000
H	5.693008000	3.574581000	2.320013000
H	3.681089000	4.485724000	3.037369000
H	1.230067000	4.541375000	3.159632000
N	3.239003000	-0.322810000	-0.193484000
C	0.755131000	0.783052000	0.562713000
H	1.250450000	-0.001855000	0.012159000
C	5.930394000	-3.586850000	-1.016734000
C	5.736023000	-2.184157000	-3.129703000
C	7.655325000	-1.893475000	-1.586756000
H	6.360033000	-2.885148000	-3.689345000
H	4.693314000	-2.467084000	-3.253140000
H	5.887579000	-1.184993000	-3.548118000
H	6.216178000	-3.564987000	0.038812000

H	4.890449000	-3.894893000	-1.098758000
H	6.557676000	-4.322873000	-1.525399000
H	8.177616000	-2.660823000	-2.160751000
H	7.896917000	-0.926951000	-2.037052000
H	8.045684000	-1.928626000	-0.566355000
F	-2.537324000	1.948743000	1.482601000
F	-1.032361000	3.767904000	2.709662000
C	-0.597983000	-1.022403000	-1.169907000
O	0.046816000	-1.646706000	-1.833581000

L2

	Coordinates (Angstroms)		
	X	Y	Z
B	-1.559163000	-0.223199000	-0.174374000
C	-2.584988000	0.492443000	-1.221715000
C	-3.957342000	0.593084000	-1.036765000
C	-2.098408000	1.138189000	-2.350233000
C	-4.790676000	1.272174000	-1.911079000
C	-2.892858000	1.823178000	-3.251232000
C	-4.256313000	1.887843000	-3.027904000
C	-2.230587000	-1.373898000	0.755565000
C	-2.306648000	-1.349251000	2.140345000
C	-2.793688000	-2.493591000	0.160022000
C	-2.907279000	-2.359715000	2.877593000
C	-3.398782000	-3.523722000	0.851291000
C	-3.456055000	-3.451588000	2.232498000
F	-4.548587000	0.047847000	0.034487000
F	-6.102236000	1.339001000	-1.683686000
F	-5.044508000	2.540140000	-3.876610000
F	-2.362500000	2.418235000	-4.318856000
F	-0.773211000	1.126316000	-2.608683000
F	-2.762832000	-2.603579000	-1.188912000
F	-3.919847000	-4.570198000	0.213304000
F	-4.030111000	-4.425635000	2.931003000
F	-2.957304000	-2.286433000	4.207396000
F	-1.808638000	-0.328642000	2.847389000
C	-0.408508000	2.899492000	1.834593000
C	0.993402000	2.798237000	1.861273000
C	1.605542000	1.707252000	1.192041000
C	-0.621016000	0.856319000	0.566960000
C	-1.182100000	1.972681000	1.199239000
C	3.819194000	0.636954000	0.431607000
C	5.235012000	0.624060000	0.564190000
C	5.918982000	-0.336544000	-0.158572000
C	3.886835000	-1.129536000	-1.125402000
O	3.325509000	-1.884218000	-1.905826000
H	6.993777000	-0.395097000	-0.071020000
N	5.315783000	-1.201483000	-0.967254000
C	3.055816000	1.622767000	1.192699000
C	5.926728000	1.563587000	1.385157000
C	3.787899000	2.562443000	1.938919000
C	1.772153000	3.754133000	2.572124000
C	3.117865000	3.619598000	2.625424000
C	5.221170000	2.510244000	2.036690000
H	7.007350000	1.506855000	1.459152000
H	5.718006000	3.252054000	2.651315000
H	3.717347000	4.329823000	3.184070000

H	1.277214000	4.573709000	3.077786000
O	-0.515804000	-1.102618000	-1.082681000
H	0.097544000	-0.575249000	-1.619496000
C	0.754241000	0.748473000	0.589639000
H	1.232880000	-0.100050000	0.132380000
N	3.219640000	-0.215783000	-0.388585000
C	6.107881000	-2.240865000	-1.735970000
C	5.612398000	-3.632332000	-1.328599000
C	5.934183000	-1.979431000	-3.235717000
C	7.596947000	-2.146575000	-1.399271000
H	5.749561000	-3.783341000	-0.254060000
H	4.563508000	-3.774606000	-1.578634000
H	6.204792000	-4.383455000	-1.856674000
H	6.253686000	-0.962685000	-3.481666000
H	6.564474000	-2.678454000	-3.790780000
H	4.900686000	-2.113414000	-3.546748000
H	8.105231000	-2.929529000	-1.964731000
H	8.035984000	-1.190912000	-1.697832000
H	7.798772000	-2.325546000	-0.339741000
F	-1.000583000	3.930199000	2.462088000
F	-2.519523000	2.127453000	1.234408000
H	-0.947697000	-1.747540000	-1.664926000

L3

	Coordinates (Angstroms)		
	X	Y	Z
B	1.495677000	-0.319143000	0.294036000
C	2.441244000	0.674985000	1.246849000
C	3.808464000	0.880575000	1.122453000
C	1.841148000	1.425243000	2.252909000
C	4.539851000	1.729872000	1.938629000
C	2.532658000	2.285617000	3.090859000
C	3.897604000	2.436579000	2.936590000
C	2.420553000	-1.420042000	-0.550249000
C	2.455190000	-1.615354000	-1.920864000
C	3.168782000	-2.340365000	0.172682000
C	3.190047000	-2.621642000	-2.535218000
C	3.918569000	-3.354974000	-0.389587000
C	3.929109000	-3.497493000	-1.766360000
F	4.521287000	0.256369000	0.166981000
F	5.858431000	1.874792000	1.768167000
F	4.582210000	3.258523000	3.733931000
F	1.893372000	2.976529000	4.041924000
F	0.513968000	1.366441000	2.457170000
F	3.203035000	-2.257552000	1.521899000
F	4.625298000	-4.197000000	0.371281000
F	4.639394000	-4.470574000	-2.339392000
F	3.182555000	-2.754236000	-3.865971000
F	1.760467000	-0.825932000	-2.760969000
C	0.423653000	2.481071000	-2.252825000
C	-0.979533000	2.413493000	-2.240423000
C	-1.603086000	1.448424000	-1.403478000
C	0.617086000	0.666552000	-0.669461000
C	1.186274000	1.640312000	-1.493947000
C	-3.851370000	0.516799000	-0.586933000
C	-5.267871000	0.533505000	-0.672229000
C	-5.969671000	-0.338112000	0.142514000

C	-3.997932000	-1.132737000	1.016020000
O	-3.397111000	-1.959157000	1.848066000
H	-7.047712000	-0.354159000	0.104448000
N	-5.372150000	-1.178391000	0.995432000
C	-3.060075000	1.400306000	-1.401788000
C	-5.950087000	1.421603000	-1.553673000
C	-3.781162000	2.276287000	-2.243433000
C	-1.743946000	3.288204000	-3.061275000
C	-3.096006000	3.214078000	-3.066944000
C	-5.214098000	2.265066000	-2.306499000
H	-7.032536000	1.405245000	-1.597916000
H	-5.694655000	2.961428000	-2.984077000
H	-3.682300000	3.872308000	-3.697586000
H	-1.230376000	4.008196000	-3.686387000
O	0.544871000	-1.087047000	1.111899000
H	-2.441016000	-1.816174000	1.762666000
N	-3.285158000	-0.344321000	0.281552000
C	-0.762194000	0.602447000	-0.643457000
H	-1.194674000	-0.134671000	0.006483000
C	-6.184825000	-2.122406000	1.897017000
C	-5.822029000	-3.564347000	1.536179000
C	-5.879887000	-1.775387000	3.356249000
C	-7.676785000	-1.913920000	1.647943000
H	-6.017571000	-3.753600000	0.477689000
H	-4.783519000	-3.802446000	1.755842000
H	-6.456008000	-4.229552000	2.125893000
H	-6.100269000	-0.723029000	3.552367000
H	-6.526889000	-2.384422000	3.990656000
H	-4.847210000	-1.980747000	3.629149000
H	-8.205528000	-2.600013000	2.311098000
H	-8.004247000	-0.901371000	1.895958000
H	-7.970139000	-2.158209000	0.624267000
F	2.525557000	1.756635000	-1.579564000
F	1.025629000	3.392204000	-3.039902000
H	1.016474000	-1.595660000	1.771164000

L4

	Coordinates (Angstroms)		
	X	Y	Z
B	-1.590543000	0.166859000	0.112304000
C	-2.330597000	-0.651030000	1.311840000
C	-3.672664000	-1.010887000	1.270715000
C	-1.629575000	-1.164951000	2.392819000
C	-4.271917000	-1.806057000	2.235033000
C	-2.185021000	-1.952366000	3.382648000
C	-3.526574000	-2.277876000	3.299448000
C	-2.520836000	1.219388000	-0.677170000
C	-2.614692000	1.318838000	-2.057566000
C	-3.281265000	2.138639000	0.036583000
C	-3.422763000	2.249262000	-2.692342000
C	-4.092675000	3.085420000	-0.558516000
C	-4.165466000	3.138320000	-1.939397000
F	-4.463869000	-0.609077000	0.269473000
F	-5.563018000	-2.119277000	2.143515000
F	-4.091748000	-3.036374000	4.232121000
F	-1.449063000	-2.397253000	4.398455000
F	-0.306816000	-0.890906000	2.521009000

F	-3.246295000	2.136617000	1.378724000
F	-4.800742000	3.941750000	0.178630000
F	-4.939911000	4.039609000	-2.535488000
F	-3.480383000	2.300492000	-4.023623000
F	-1.907651000	0.515390000	-2.860611000
C	-0.434411000	-2.799611000	-2.108294000
C	0.970664000	-2.738339000	-2.078672000
C	1.583276000	-1.698730000	-1.334071000
C	-0.640872000	-0.837134000	-0.716029000
C	-1.206141000	-1.888586000	-1.447069000
C	3.791139000	-0.622596000	-0.552055000
C	5.210791000	-0.661798000	-0.608617000
C	5.892117000	0.314100000	0.097099000
C	3.850881000	1.256362000	0.868134000
O	3.271650000	2.106343000	1.540539000
H	6.971796000	0.323573000	0.072346000
N	5.281562000	1.253369000	0.812755000
C	3.034230000	-1.629969000	-1.291427000
C	5.912418000	-1.655706000	-1.353502000
C	3.772844000	-2.591432000	-2.004071000
C	1.754915000	-3.696330000	-2.779951000
C	3.105647000	-3.616537000	-2.740649000
C	5.209584000	-2.590080000	-2.025013000
H	6.996910000	-1.641333000	-1.363192000
H	5.712169000	-3.361628000	-2.596800000
H	3.711547000	-4.339573000	-3.275376000
H	1.262739000	-4.481116000	-3.340559000
O	-0.507173000	1.197413000	0.946643000
H	1.242556000	2.308726000	1.008447000
N	3.186890000	0.316875000	0.169691000
C	0.734610000	-0.781631000	-0.672970000
H	1.233008000	-0.023363000	-0.100170000
C	0.343589000	2.175731000	0.386754000
O	0.056509000	2.718078000	-0.618512000
C	6.072167000	2.304265000	1.571250000
C	5.680026000	3.688089000	1.043603000
C	5.778990000	2.148340000	3.066780000
C	7.573407000	2.114094000	1.350515000
H	5.872445000	3.753919000	-0.030966000
H	4.631677000	3.906898000	1.232258000
H	6.293163000	4.439736000	1.546406000
H	6.042217000	1.140924000	3.401773000
H	6.391734000	2.863078000	3.621238000
H	4.731232000	2.336296000	3.290154000
H	8.082263000	2.900951000	1.909993000
H	7.937383000	1.155978000	1.730780000
H	7.859042000	2.223534000	0.300879000
H	-0.090832000	0.784591000	1.725069000
F	-2.545719000	-2.006767000	-1.516425000
F	-1.030382000	-3.781659000	-2.805260000

L5

	Coordinates (Angstroms)		
	X	Y	Z
B	-1.620364000	0.087517000	0.142254000
C	-2.774294000	-0.542303000	1.091825000
C	-4.121067000	-0.538214000	0.757124000

C	-2.455226000	-1.194371000	2.274445000
C	-5.092138000	-1.129257000	1.548754000
C	-3.394204000	-1.790981000	3.095755000
C	-4.727057000	-1.756833000	2.726097000
C	-2.069018000	1.386260000	-0.703725000
C	-2.013171000	1.480735000	-2.086027000
C	-2.552860000	2.509895000	-0.044358000
C	-2.413049000	2.615372000	-2.775637000
C	-2.953773000	3.660859000	-0.694963000
C	-2.883945000	3.710915000	-2.076716000
F	-4.542558000	0.031659000	-0.378651000
F	-6.373660000	-1.103474000	1.183563000
F	-5.649498000	-2.326758000	3.495155000
F	-3.031270000	-2.393799000	4.227237000
F	-1.171815000	-1.251237000	2.670033000
F	-2.632739000	2.504240000	1.293859000
F	-3.403562000	4.713672000	-0.013395000
F	-3.269124000	4.804700000	-2.726308000
F	-2.348027000	2.657320000	-4.106542000
F	-1.576023000	0.459059000	-2.831699000
C	-0.468651000	-3.043011000	-1.849014000
C	0.931650000	-3.028705000	-1.723987000
C	1.538926000	-1.959134000	-1.015414000
C	-0.690631000	-1.017123000	-0.561019000
C	-1.249040000	-2.071321000	-1.291148000
C	3.746877000	-0.880799000	-0.255695000
C	5.160373000	-0.994743000	-0.170844000
C	5.830553000	0.032590000	0.468995000
C	3.797567000	1.187707000	0.852640000
O	3.205938000	2.175897000	1.300185000
H	6.904711000	-0.018931000	0.565739000
N	5.213438000	1.096421000	0.979959000
C	2.988661000	-1.942240000	-0.904210000
C	5.859576000	-2.107447000	-0.727015000
C	3.725529000	-3.002533000	-1.459468000
C	1.715089000	-4.073212000	-2.291348000
C	3.061170000	-4.059752000	-2.150673000
C	5.157930000	-3.071070000	-1.357986000
H	6.939244000	-2.154480000	-0.637292000
H	5.657853000	-3.924481000	-1.801332000
H	3.663713000	-4.857875000	-2.569647000
H	1.222344000	-4.877105000	-2.823622000
N	3.142050000	0.190002000	0.245277000
C	0.683663000	-0.986644000	-0.444283000
H	1.143395000	-0.181176000	0.100855000
O	0.583468000	2.270365000	0.519909000
H	1.501098000	2.185214000	0.865083000
H	-0.492050000	1.017340000	0.809714000
H	-0.859996000	0.441804000	1.262129000
C	5.989340000	2.202234000	1.676265000
C	5.781022000	3.508077000	0.902905000
C	5.502218000	2.299757000	3.125153000
C	7.484672000	1.884214000	1.690695000
H	6.101784000	3.388798000	-0.135885000
H	4.741015000	3.825545000	0.921811000
H	6.394497000	4.286821000	1.362040000
H	5.650011000	1.346060000	3.639551000

H	6.091876000	3.061094000	3.641281000
H	4.451481000	2.575387000	3.179969000
H	7.982929000	2.708405000	2.203693000
H	7.714038000	0.969077000	2.242699000
H	7.910686000	1.821093000	0.685958000
H	0.689753000	2.395029000	-0.428100000
F	-2.581595000	-2.137840000	-1.468263000
F	-1.052393000	-4.041296000	-2.533442000

L6

	Coordinates (Angstroms)		
	X	Y	Z
B	1.782330000	-0.085869000	-0.524714000
C	1.270738000	-1.222010000	0.545119000
C	0.884517000	-0.978269000	1.857543000
C	1.179614000	-2.552407000	0.160998000
C	0.416053000	-1.963374000	2.713124000
C	0.716359000	-3.569571000	0.978107000
C	0.328936000	-3.270426000	2.270755000
C	3.412435000	-0.093539000	-0.613807000
C	4.081887000	-0.203703000	-1.823458000
C	4.235885000	-0.025403000	0.500927000
C	5.463714000	-0.252953000	-1.932269000
C	5.617498000	-0.069265000	0.441350000
C	6.237882000	-0.188152000	-0.789599000
F	0.943839000	0.258287000	2.375302000
F	0.050719000	-1.664395000	3.963489000
F	-0.119960000	-4.229868000	3.080950000
F	0.638707000	-4.827686000	0.533918000
F	1.542572000	-2.921440000	-1.087142000
F	3.696604000	0.113092000	1.724646000
F	6.357568000	0.006624000	1.552235000
F	7.568642000	-0.233431000	-0.872039000
F	6.054083000	-0.362249000	-3.127041000
F	3.401506000	-0.264829000	-2.982495000
C	0.973328000	3.730502000	0.159563000
C	-0.425408000	3.734666000	0.235487000
C	-1.111218000	2.509192000	0.013662000
C	1.056581000	1.348493000	-0.256586000
C	1.683738000	2.583048000	-0.082192000
C	-3.413081000	1.398948000	-0.211517000
C	-4.809830000	1.480919000	0.019594000
C	-5.563704000	0.348253000	-0.232612000
C	-3.683850000	-0.754672000	-1.001304000
O	-3.193974000	-1.823621000	-1.555239000
H	-6.623940000	0.359836000	-0.033809000
N	-5.037757000	-0.780169000	-0.716857000
C	-2.566010000	2.522443000	0.095998000
C	-5.419728000	2.680351000	0.489404000
C	-3.219313000	3.707889000	0.491441000
C	-1.130192000	4.926886000	0.566100000
C	-2.475592000	4.904315000	0.712759000
C	-4.638765000	3.762547000	0.688894000
H	-6.489830000	2.704702000	0.658375000
H	-5.068345000	4.701084000	1.019579000
H	-3.015072000	5.801362000	0.993814000
H	-0.575401000	5.843607000	0.722891000

H	-2.225909000	-1.693552000	-1.806873000
N	-2.919512000	0.265157000	-0.745731000
C	-0.330868000	1.360557000	-0.217214000
H	-0.837865000	0.419425000	-0.349555000
H	1.388643000	-0.440837000	-1.620927000
O	-0.779522000	-1.657243000	-2.406297000
H	-0.165099000	-0.959050000	-2.133632000
C	-5.899693000	-2.027061000	-0.960280000
C	-5.921184000	-2.316207000	-2.462991000
C	-5.321776000	-3.181642000	-0.137394000
C	-7.330918000	-1.769429000	-0.491728000
H	-6.302367000	-1.450214000	-3.010412000
H	-4.939734000	-2.579911000	-2.850569000
H	-6.598253000	-3.154812000	-2.638944000
H	-5.266837000	-2.907701000	0.919467000
H	-5.995693000	-4.035482000	-0.232060000
H	-4.335424000	-3.486392000	-0.479354000
H	-7.888870000	-2.690132000	-0.667932000
H	-7.388834000	-1.551207000	0.577646000
H	-7.824194000	-0.977203000	-1.059744000
H	-0.259608000	-2.466133000	-2.352698000
F	3.025760000	2.696176000	-0.121684000
F	1.640141000	4.882873000	0.352245000

L7

	Coordinates (Angstroms)		
	X	Y	Z
B	1.769317000	-0.242439000	-0.607823000
C	1.266071000	-1.410882000	0.427157000
C	1.746561000	-1.519264000	1.725470000
C	0.218110000	-2.264701000	0.137056000
C	1.252242000	-2.412123000	2.658803000
C	-0.322951000	-3.169084000	1.037184000
C	0.202333000	-3.246648000	2.311739000
C	3.373026000	-0.204778000	-0.918589000
C	3.902562000	0.861386000	-1.637517000
C	4.293064000	-1.199599000	-0.622237000
C	5.232596000	0.971277000	-1.999545000
C	5.636453000	-1.134148000	-0.964592000
C	6.113690000	-0.038330000	-1.655381000
F	2.745354000	-0.717535000	2.130738000
F	1.763759000	-2.472917000	3.890854000
F	-0.297174000	-4.107906000	3.198678000
F	-1.349033000	-3.952268000	0.685822000
F	-0.380710000	-2.239212000	-1.086561000
F	3.919407000	-2.321486000	0.022382000
F	6.471212000	-2.126567000	-0.637530000
F	7.401923000	0.042549000	-1.994454000
F	5.674364000	2.034051000	-2.680373000
F	3.099811000	1.869677000	-2.027144000
C	1.077108000	3.236844000	1.184995000
C	-0.313296000	3.362408000	1.057438000
C	-1.023461000	2.313560000	0.412253000
C	1.107469000	1.130074000	-0.004846000
C	1.754861000	2.150152000	0.696646000
C	-3.349277000	1.383687000	-0.134221000
C	-4.749669000	1.600773000	-0.196231000

C	-5.538453000	0.562824000	-0.662092000
C	-3.672957000	-0.758810000	-0.921193000
O	-3.181540000	-1.927752000	-1.266242000
H	-6.606352000	0.695113000	-0.733864000
N	-5.037707000	-0.619345000	-1.035501000
C	-2.474762000	2.421865000	0.340788000
C	-5.330711000	2.831957000	0.225212000
C	-3.098881000	3.599600000	0.804484000
C	-0.985850000	4.520752000	1.539494000
C	-2.326304000	4.643635000	1.392018000
C	-4.517507000	3.788377000	0.720758000
H	-6.402833000	2.970094000	0.154021000
H	-4.924632000	4.728814000	1.073643000
H	-2.839636000	5.532895000	1.738899000
H	-0.411249000	5.307665000	2.012114000
H	-2.215068000	-1.905754000	-1.158601000
N	-2.880063000	0.173450000	-0.503048000
C	-0.269047000	1.253119000	-0.125109000
H	-0.784008000	0.471752000	-0.655962000
H	1.229809000	-0.437441000	-1.680202000
C	-5.945161000	-1.751924000	-1.544059000
C	-5.523231000	-2.111046000	-2.971002000
C	-5.834433000	-2.928036000	-0.571144000
C	-7.395751000	-1.274718000	-1.574563000
H	-5.548321000	-1.225389000	-3.611287000
H	-4.533267000	-2.558980000	-3.017969000
H	-6.242143000	-2.833915000	-3.361866000
H	-6.109568000	-2.614145000	0.439112000
H	-6.537919000	-3.699020000	-0.891816000
H	-4.838379000	-3.364979000	-0.550613000
H	-7.992594000	-2.111048000	-1.940785000
H	-7.775825000	-1.013387000	-0.584229000
H	-7.546191000	-0.439278000	-2.263130000
F	3.084475000	2.114920000	0.905572000
F	1.764571000	4.210392000	1.809393000

L8

	Coordinates (Angstroms)		
	X	Y	Z
B	-1.562334000	0.185361000	0.198283000
C	-2.536613000	-0.753490000	1.129419000
C	-3.897927000	-0.938812000	0.928103000
C	-1.998323000	-1.516521000	2.155993000
C	-4.672443000	-1.788808000	1.701949000
C	-2.730337000	-2.375261000	2.955063000
C	-4.087216000	-2.509235000	2.726358000
C	-2.316546000	1.399587000	-0.567400000
C	-2.168947000	1.693307000	-1.915749000
C	-3.171877000	2.247823000	0.128059000
C	-2.835164000	2.739134000	-2.538799000
C	-3.846990000	3.303898000	-0.451988000
C	-3.678259000	3.548810000	-1.803761000
F	-4.543831000	-0.296464000	-0.054855000
F	-5.977981000	-1.919925000	1.464305000
F	-4.818885000	-3.326233000	3.479117000
F	-2.144860000	-3.070876000	3.930143000
F	-0.671365000	-1.453573000	2.416440000

F	-3.362130000	2.066856000	1.445833000
F	-4.649218000	4.086180000	0.270957000
F	-4.317952000	4.558359000	-2.386166000
F	-2.662162000	2.972041000	-3.840337000
F	-1.364437000	0.970263000	-2.702777000
C	-0.467779000	-2.673045000	-2.208831000
C	0.934635000	-2.698918000	-2.107420000
C	1.569552000	-1.714872000	-1.306742000
C	-0.634718000	-0.792531000	-0.699985000
C	-1.218103000	-1.756351000	-1.531491000
C	3.806860000	-0.753711000	-0.485470000
C	5.217901000	-0.902605000	-0.419738000
C	5.910663000	0.052182000	0.302593000
C	3.910501000	1.228392000	0.764688000
O	3.339436000	2.213585000	1.252092000
H	6.983167000	-0.030722000	0.394300000
N	5.317925000	1.086137000	0.899506000
C	3.020105000	-1.737694000	-1.214211000
C	5.888989000	-1.979615000	-1.073680000
C	3.730393000	-2.757069000	-1.872006000
C	1.690433000	-3.696451000	-2.784759000
C	3.038100000	-3.727656000	-2.655897000
C	5.161748000	-2.865860000	-1.784191000
H	6.967386000	-2.060179000	-0.993483000
H	5.640920000	-3.688174000	-2.303029000
H	3.618611000	-4.494551000	-3.156442000
H	1.175706000	-4.432188000	-3.390060000
O	-0.602126000	0.923707000	1.272280000
N	3.229488000	0.286618000	0.104114000
C	0.741196000	-0.790891000	-0.623819000
H	1.233190000	-0.044058000	-0.029351000
C	-0.048644000	2.309470000	1.247232000
H	0.366298000	2.427645000	2.250324000
H	-0.925842000	2.934039000	1.109881000
O	0.839916000	2.502536000	0.251864000
H	1.752811000	2.333594000	0.582393000
C	6.118333000	2.107930000	1.689266000
C	5.980919000	3.471903000	1.006088000
C	5.597063000	2.124284000	3.129575000
C	7.598222000	1.724923000	1.717062000
H	6.328159000	3.413912000	-0.029264000
H	4.952524000	3.826220000	1.018702000
H	6.608134000	4.192026000	1.536865000
H	5.687666000	1.130903000	3.578034000
H	6.206996000	2.817473000	3.713580000
H	4.559365000	2.446673000	3.179431000
H	8.115190000	2.482539000	2.308349000
H	7.772035000	0.758888000	2.198483000
H	8.050711000	1.724348000	0.722271000
H	0.003574000	0.315000000	1.717353000
F	-2.556299000	-1.786459000	-1.687654000
F	-1.082990000	-3.582568000	-2.984789000

TSL2-3

	Coordinates (Angstroms)		
	X	Y	Z
B	1.266667000	-0.044427000	-0.257429000

C	1.563868000	-0.634496000	1.248775000
C	2.809125000	-0.685512000	1.859610000
C	0.512382000	-1.078924000	2.041141000
C	3.012829000	-1.170909000	3.141904000
C	0.671074000	-1.573581000	3.324300000
C	1.935927000	-1.624078000	3.879706000
C	2.595275000	-0.044522000	-1.219780000
C	3.175561000	1.086087000	-1.776824000
C	3.243170000	-1.233708000	-1.527393000
C	4.313107000	1.042557000	-2.571114000
C	4.374426000	-1.327463000	-2.313621000
C	4.917300000	-0.169489000	-2.841841000
F	3.907537000	-0.239475000	1.228629000
F	4.238028000	-1.199656000	3.672002000
F	2.112660000	-2.094404000	5.113123000
F	-0.382521000	-1.992537000	4.030341000
F	-0.749697000	-1.028587000	1.584024000
F	2.757917000	-2.395947000	-1.033586000
F	4.943022000	-2.507213000	-2.566133000
F	6.006387000	-0.225748000	-3.604430000
F	4.828101000	2.164413000	-3.077603000
F	2.669089000	2.308757000	-1.566317000
C	0.000856000	3.471750000	1.067567000
C	-1.302797000	3.514641000	0.531726000
C	-1.660425000	2.464485000	-0.337175000
C	0.516088000	1.394109000	-0.097225000
C	0.829762000	2.401844000	0.833431000
C	-3.513801000	0.876874000	-0.806216000
C	-4.885499000	0.546247000	-0.811154000
C	-5.167360000	-0.807487000	-0.931199000
C	-2.881293000	-1.351502000	-0.935471000
O	-1.910225000	-2.174335000	-0.907220000
H	-6.193328000	-1.142418000	-0.954458000
N	-4.217070000	-1.761964000	-0.992348000
C	-3.063185000	2.218652000	-0.549130000
C	-5.854123000	1.585415000	-0.605174000
C	-4.023892000	3.152863000	-0.161607000
C	-2.282714000	4.501265000	0.862174000
C	-3.581661000	4.355771000	0.472314000
C	-5.426796000	2.832428000	-0.284837000
H	-6.911519000	1.353368000	-0.661709000
H	-6.147478000	3.612493000	-0.065390000
H	-4.315143000	5.109480000	0.736084000
H	-1.987233000	5.362242000	1.450403000
O	0.266594000	-1.053022000	-0.970870000
H	-0.833236000	-1.519439000	-0.835324000
N	-2.607460000	-0.078546000	-0.887549000
C	-0.667334000	1.561318000	-0.768972000
H	-0.889147000	0.875432000	-1.559526000
C	-4.571629000	-3.241025000	-1.073625000
C	-3.929529000	-3.816707000	-2.338591000
C	-4.063406000	-3.920079000	0.201022000
C	-6.084398000	-3.427634000	-1.163996000
H	-4.296370000	-3.291674000	-3.224684000
H	-2.843481000	-3.752286000	-2.308456000
H	-4.210901000	-4.868504000	-2.423223000
H	-4.530679000	-3.471581000	1.081833000

H	-4.339629000	-4.976328000	0.165988000
H	-2.981635000	-3.847746000	0.297167000
H	-6.269733000	-4.499597000	-1.249947000
H	-6.604330000	-3.077417000	-0.268772000
H	-6.511819000	-2.948971000	-2.048756000
H	0.773755000	-1.778954000	-1.346203000
F	2.000810000	2.356628000	1.499692000
F	0.403544000	4.452804000	1.897345000

TSL3-4

	Coordinates (Angstroms)		
	X	Y	Z
B	-1.486926000	-0.179546000	-0.209556000
C	-2.424710000	0.716859000	-1.230367000
C	-3.782163000	0.952929000	-1.057448000
C	-1.860523000	1.388853000	-2.305183000
C	-4.528505000	1.767037000	-1.895328000
C	-2.562860000	2.209789000	-3.168810000
C	-3.916775000	2.398330000	-2.961877000
C	-2.325940000	-1.320761000	0.606220000
C	-2.286241000	-1.528133000	1.975543000
C	-3.097699000	-2.235760000	-0.100515000
C	-2.969074000	-2.555735000	2.610572000
C	-3.792444000	-3.273811000	0.491432000
C	-3.727614000	-3.434954000	1.864188000
F	-4.457182000	0.395657000	-0.040840000
F	-5.832890000	1.948307000	-1.680457000
F	-4.619999000	3.181994000	-3.776831000
F	-1.951910000	2.818788000	-4.186663000
F	-0.533943000	1.269400000	-2.554444000
F	-3.208317000	-2.134403000	-1.435464000
F	-4.522634000	-4.118280000	-0.241896000
F	-4.388654000	-4.428022000	2.456909000
F	-2.890706000	-2.706905000	3.935430000
F	-1.553761000	-0.741065000	2.779506000
C	-0.435929000	2.752652000	2.218736000
C	0.969950000	2.747360000	2.133910000
C	1.578136000	1.765253000	1.315009000
C	-0.640275000	0.876828000	0.703841000
C	-1.205850000	1.852955000	1.532644000
C	3.764443000	0.743341000	0.472033000
C	5.178566000	0.804075000	0.416120000
C	5.814600000	-0.181375000	-0.321142000
C	3.761683000	-1.159550000	-0.875410000
O	3.097493000	-2.058578000	-1.476773000
H	6.891370000	-0.178068000	-0.392852000
N	5.159100000	-1.158246000	-0.958052000
C	3.025640000	1.732153000	1.226930000
C	5.900277000	1.824210000	1.108806000
C	3.777352000	2.697902000	1.917373000
C	1.764533000	3.698066000	2.835612000
C	3.116157000	3.676131000	2.718755000
C	5.212653000	2.729415000	1.838156000
H	6.981935000	1.848568000	1.042403000
H	5.733251000	3.508906000	2.382896000
H	3.722248000	4.405304000	3.244456000
H	1.278934000	4.441217000	3.456161000

O	-0.498138000	-1.008684000	-1.069750000
H	1.872981000	-2.062938000	-1.074544000
N	3.135384000	-0.237980000	-0.176730000
C	0.731620000	0.878591000	0.618601000
H	1.201030000	0.164092000	-0.022979000
C	0.719544000	-2.253943000	-0.472987000
O	0.249635000	-2.932508000	0.317522000
C	5.901330000	-2.240501000	-1.737887000
C	5.565632000	-3.599348000	-1.116447000
C	5.483084000	-2.148054000	-3.207333000
C	7.410856000	-2.024074000	-1.642002000
H	5.831965000	-3.609808000	-0.056018000
H	4.512230000	-3.847851000	-1.223369000
H	6.156033000	-4.364984000	-1.624338000
H	5.709575000	-1.155443000	-3.605945000
H	6.055901000	-2.883166000	-3.776935000
H	4.423495000	-2.354410000	-3.343671000
H	7.884114000	-2.827760000	-2.208316000
H	7.729328000	-1.079354000	-2.089134000
H	7.778596000	-2.088574000	-0.614575000
F	-2.543002000	1.918599000	1.680229000
F	-1.035566000	3.671828000	2.996889000
H	-0.051327000	-0.469707000	-1.731772000

TSL1-7

	Coordinates (Angstroms)		
	X	Y	Z
B	-1.335085000	-0.186407000	-0.231278000
C	-2.274760000	-0.615804000	0.995266000
C	-3.654743000	-0.475390000	0.989288000
C	-1.736795000	-1.208052000	2.130876000
C	-4.457147000	-0.880577000	2.043822000
C	-2.503022000	-1.619111000	3.206235000
C	-3.876380000	-1.453114000	3.160155000
C	-1.824329000	1.028339000	-1.154174000
C	-2.110226000	0.884057000	-2.504796000
C	-2.002360000	2.305638000	-0.637109000
C	-2.523412000	1.939145000	-3.302577000
C	-2.405904000	3.384768000	-1.402375000
C	-2.668201000	3.197515000	-2.748191000
F	-4.283541000	0.049978000	-0.071659000
F	-5.781015000	-0.728204000	1.990152000
F	-4.632335000	-1.846722000	4.181410000
F	-1.937163000	-2.174343000	4.278539000
F	-0.413068000	-1.400426000	2.219886000
F	-1.781063000	2.536584000	0.664210000
F	-2.551162000	4.594039000	-0.859520000
F	-3.064008000	4.219810000	-3.501546000
F	-2.789616000	1.750986000	-4.595883000
F	-2.019517000	-0.312078000	-3.100172000
C	0.214830000	-3.645843000	-1.344323000
C	1.541885000	-3.248319000	-1.603769000
C	1.816569000	-1.870357000	-1.508433000
C	-0.485052000	-1.354172000	-0.925006000
C	-0.726371000	-2.739212000	-0.915521000
C	3.442886000	-0.296485000	-0.498018000
C	4.760831000	0.043866000	-0.127663000

C	4.871409000	1.135027000	0.721880000
C	2.523042000	1.355674000	0.859288000
O	1.476456000	1.863567000	1.345659000
H	5.848098000	1.470306000	1.035892000
N	3.811699000	1.805237000	1.209257000
C	3.178318000	-1.466110000	-1.295283000
C	5.849057000	-0.786113000	-0.560732000
C	4.230276000	-2.352417000	-1.514916000
C	2.624530000	-4.140979000	-1.885041000
C	3.911829000	-3.691368000	-1.910037000
C	5.581827000	-1.946087000	-1.211690000
H	6.867483000	-0.503539000	-0.319218000
H	6.389498000	-2.616623000	-1.483622000
H	4.721081000	-4.380646000	-2.124248000
H	2.414431000	-5.189648000	-2.059006000
H	0.373477000	1.053071000	0.833527000
N	2.415025000	0.364738000	0.004088000
C	0.750510000	-0.963111000	-1.382421000
H	0.936159000	0.087342000	-1.505958000
H	-0.274450000	0.504920000	0.552555000
C	3.983173000	2.997768000	2.140652000
C	3.306885000	4.206371000	1.487586000
C	3.362581000	2.645956000	3.495523000
C	5.463228000	3.315844000	2.345586000
H	3.760906000	4.415762000	0.515214000
H	2.237419000	4.051772000	1.358017000
H	3.454417000	5.077553000	2.129534000
H	3.836071000	1.751933000	3.910498000
H	3.536412000	3.475688000	4.184465000
H	2.290474000	2.477882000	3.416859000
H	5.516038000	4.190491000	2.995992000
H	6.000196000	2.505341000	2.845031000
H	5.969621000	3.570907000	1.411019000
F	-1.923599000	-3.211643000	-0.521301000
F	-0.111737000	-4.948303000	-1.424488000

TSL5-6

	Coordinates (Angstroms)		
	X	Y	Z
B	-1.594806000	0.085822000	0.134978000
C	-2.713934000	-0.559498000	1.117573000
C	-4.068050000	-0.598018000	0.817120000
C	-2.349338000	-1.177010000	2.305788000
C	-5.004632000	-1.194218000	1.645918000
C	-3.252899000	-1.777557000	3.163492000
C	-4.595055000	-1.784830000	2.827545000
C	-2.100759000	1.358618000	-0.720979000
C	-2.070484000	1.443705000	-2.104678000
C	-2.619676000	2.468971000	-0.065320000
C	-2.532386000	2.552136000	-2.798556000
C	-3.084374000	3.593713000	-0.719817000
C	-3.041440000	3.632456000	-2.103044000
F	-4.532843000	-0.062236000	-0.318986000
F	-6.295438000	-1.207928000	1.312504000
F	-5.483535000	-2.360031000	3.632638000
F	-2.847144000	-2.345602000	4.298889000
F	-1.056186000	-1.196324000	2.671649000

F	-2.675131000	2.476299000	1.274529000
F	-3.568204000	4.632799000	-0.040031000
F	-3.485800000	4.701299000	-2.756655000
F	-2.490266000	2.583324000	-4.130873000
F	-1.598016000	0.436590000	-2.849285000
C	-0.434178000	-3.040664000	-1.873848000
C	0.966764000	-3.016914000	-1.751098000
C	1.564310000	-1.943155000	-1.042203000
C	-0.671048000	-1.016612000	-0.582878000
C	-1.221497000	-2.074743000	-1.313985000
C	3.755006000	-0.837143000	-0.285456000
C	5.167587000	-0.937734000	-0.181780000
C	5.818169000	0.098618000	0.464369000
C	3.771898000	1.242819000	0.800411000
O	3.161148000	2.232916000	1.222276000
H	6.890720000	0.056420000	0.580765000
N	5.183515000	1.160540000	0.959891000
C	3.012338000	-1.911105000	-0.928746000
C	5.881270000	-2.048146000	-0.725217000
C	3.761849000	-2.966328000	-1.475174000
C	1.762509000	-4.054431000	-2.314708000
C	3.108458000	-4.030021000	-2.167256000
C	5.194181000	-3.019879000	-1.360775000
H	6.960246000	-2.086579000	-0.623859000
H	5.706573000	-3.869943000	-1.796362000
H	3.719142000	-4.824342000	-2.581715000
H	1.279507000	-4.863396000	-2.848405000
N	3.135502000	0.235605000	0.192736000
C	0.702187000	-0.980591000	-0.467327000
H	1.158014000	-0.178232000	0.083411000
O	0.547922000	2.263224000	0.484834000
H	1.478250000	2.192033000	0.813550000
H	-0.411732000	1.092713000	0.762299000
H	-0.816627000	0.484292000	1.188083000
C	5.937073000	2.272940000	1.670619000
C	5.752499000	3.570649000	0.878223000
C	5.403517000	2.383567000	3.102311000
C	7.431032000	1.955204000	1.736092000
H	6.125861000	3.447863000	-0.142424000
H	4.709204000	3.876251000	0.843762000
H	6.333299000	4.360035000	1.361094000
H	5.530362000	1.432768000	3.627545000
H	5.980382000	3.145667000	3.631465000
H	4.353002000	2.664741000	3.121025000
H	7.912539000	2.781670000	2.261241000
H	7.640977000	1.042056000	2.299057000
H	7.889948000	1.887498000	0.746399000
H	0.625817000	2.408799000	-0.463910000
F	-2.554052000	-2.153185000	-1.486573000
F	-1.011441000	-4.043935000	-2.557013000

TSL7

	Coordinates (Angstroms)		
	X	Y	Z
B	1.620556000	-0.088818000	0.063602000
C	2.501474000	0.587044000	1.244224000
C	3.860848000	0.854071000	1.159645000

C	1.885379000	1.028623000	2.407399000
C	4.571111000	1.495632000	2.160836000
C	2.558538000	1.669570000	3.433360000
C	3.915768000	1.903665000	3.307958000
C	2.388472000	-1.162688000	-0.875988000
C	2.265636000	-1.236023000	-2.259833000
C	3.216804000	-2.133063000	-0.317093000
C	2.923889000	-2.181936000	-3.032330000
C	3.888306000	-3.093996000	-1.049688000
C	3.739668000	-3.117298000	-2.425242000
F	4.561653000	0.496427000	0.072568000
F	5.879555000	1.724758000	2.028929000
F	4.584526000	2.519744000	4.280774000
F	1.914568000	2.065420000	4.533261000
F	0.568010000	0.841900000	2.577652000
F	3.396537000	-2.171223000	1.016065000
F	4.666644000	-3.993676000	-0.448947000
F	4.370751000	-4.033114000	-3.153072000
F	2.771490000	-2.198854000	-4.356610000
F	1.492454000	-0.376136000	-2.933926000
C	0.451075000	3.058850000	-1.897600000
C	-0.952353000	2.998402000	-1.833714000
C	-1.554240000	1.909064000	-1.149977000
C	0.681490000	1.004992000	-0.650852000
C	1.234245000	2.099119000	-1.324797000
C	-3.757849000	0.807068000	-0.420486000
C	-5.176077000	0.814375000	-0.491712000
C	-5.837779000	-0.230319000	0.127514000
C	-3.796935000	-1.133819000	0.883762000
O	-3.193274000	-2.000991000	1.545078000
H	-6.914914000	-0.277090000	0.075280000
N	-5.206537000	-1.201200000	0.787712000
C	-3.006378000	1.849727000	-1.099776000
C	-5.888293000	1.844835000	-1.175526000
C	-3.753504000	2.850707000	-1.746633000
C	-1.743889000	4.004751000	-2.455128000
C	-3.094759000	3.925312000	-2.414287000
C	-5.190755000	2.833682000	-1.770371000
H	-6.971935000	1.814839000	-1.199576000
H	-5.699584000	3.637614000	-2.289726000
H	-3.703292000	4.683685000	-2.894064000
H	-1.254418000	4.826375000	-2.962959000
H	-1.717337000	-1.840816000	1.689827000
N	-3.143522000	-0.152644000	0.267394000
C	-0.693687000	0.942212000	-0.576104000
H	-1.146139000	0.121393000	-0.051028000
H	0.748533000	-0.830320000	0.716523000
O	-0.378783000	-2.549843000	-0.088647000
C	0.122815000	-2.115247000	1.062246000
H	1.071312000	-2.530207000	1.400673000
O	-0.727643000	-1.847774000	1.988958000
C	-5.970897000	-2.349501000	1.431140000
C	-5.478934000	-3.663998000	0.817045000
C	-5.752492000	-2.294775000	2.945953000
C	-7.467943000	-2.219434000	1.150312000
H	-5.631651000	-3.657279000	-0.265823000
H	-4.427210000	-3.842702000	1.028490000

H	-6.064661000	-4.483992000	1.238710000
H	-6.083140000	-1.331145000	3.343460000
H	-6.352414000	-3.079847000	3.412324000
H	-4.708818000	-2.450646000	3.208917000
H	-7.955847000	-3.078278000	1.613972000
H	-7.903415000	-1.320153000	1.593027000
H	-7.696357000	-2.250901000	0.081820000
H	0.337265000	-2.812488000	-0.683367000
F	2.572271000	2.208015000	-1.438730000
F	1.031179000	4.084982000	-2.545616000

TSL8

	Coordinates (Angstroms)		
	X	Y	Z
B	-1.529779000	0.199035000	0.260798000
C	-2.558601000	-0.766600000	1.139660000
C	-3.912716000	-0.950224000	0.895898000
C	-2.062470000	-1.525236000	2.190416000
C	-4.719471000	-1.791585000	1.646490000
C	-2.826333000	-2.377852000	2.968002000
C	-4.174751000	-2.509253000	2.694546000
C	-2.313579000	1.398555000	-0.538499000
C	-2.178916000	1.693923000	-1.885925000
C	-3.136602000	2.269373000	0.167309000
C	-2.820908000	2.759965000	-2.501635000
C	-3.791759000	3.344106000	-0.404538000
C	-3.632901000	3.591139000	-1.756786000
F	-4.528454000	-0.307049000	-0.109601000
F	-6.019103000	-1.917736000	1.365115000
F	-4.936693000	-3.321385000	3.426458000
F	-2.277680000	-3.072479000	3.968300000
F	-0.746073000	-1.473205000	2.502590000
F	-3.334982000	2.091057000	1.484869000
F	-4.570437000	4.144301000	0.329019000
F	-4.254103000	4.620917000	-2.330170000
F	-2.654122000	2.991976000	-3.806434000
F	-1.400357000	0.952689000	-2.689592000
C	-0.490132000	-2.662797000	-2.224647000
C	0.910409000	-2.709733000	-2.110656000
C	1.549672000	-1.739185000	-1.296656000
C	-0.648235000	-0.805456000	-0.682701000
C	-1.232663000	-1.743689000	-1.540797000
C	3.797092000	-0.798642000	-0.507353000
C	5.195115000	-0.990375000	-0.371237000
C	5.888160000	-0.041072000	0.358343000
C	3.947211000	1.224041000	0.621304000
O	3.401847000	2.307513000	1.016820000
H	6.947095000	-0.165706000	0.523527000
N	5.306167000	1.041628000	0.881535000
C	2.998893000	-1.785800000	-1.198202000
C	5.854429000	-2.109161000	-0.963511000
C	3.699165000	-2.836274000	-1.821377000
C	1.657900000	-3.722684000	-2.774343000
C	3.001143000	-3.794805000	-2.612656000
C	5.121763000	-2.983101000	-1.684615000
H	6.924503000	-2.225613000	-0.837088000
H	5.592221000	-3.830222000	-2.170465000

H	3.571096000	-4.581865000	-3.093243000
H	1.137584000	-4.445465000	-3.390540000
O	-0.631879000	0.934973000	1.224224000
N	3.245581000	0.315921000	-0.014459000
C	0.728182000	-0.823871000	-0.596368000
H	1.204911000	-0.097776000	0.036185000
C	0.289791000	2.711312000	1.061454000
H	0.541463000	2.629230000	2.121832000
H	-0.671638000	3.155777000	0.811969000
O	1.183823000	2.697709000	0.193600000
H	2.314129000	2.436351000	0.615112000
C	6.103052000	2.049584000	1.709187000
C	6.155655000	3.372738000	0.941936000
C	5.435913000	2.190034000	3.080775000
C	7.529901000	1.543891000	1.920877000
H	6.613498000	3.225721000	-0.039892000
H	5.168168000	3.811226000	0.813785000
H	6.775676000	4.072987000	1.506417000
H	5.358498000	1.215079000	3.569395000
H	6.063528000	2.832701000	3.701818000
H	4.446770000	2.636385000	3.013536000
H	8.039236000	2.280629000	2.543811000
H	7.559087000	0.588515000	2.451397000
H	8.092298000	1.465167000	0.987598000
H	-0.091420000	0.335198000	1.744153000
F	-2.568890000	-1.756450000	-1.714325000
F	-1.110307000	-3.557693000	-3.015356000

TSL9

	Coordinates (Angstroms)		
	X	Y	Z
B	-1.644175000	0.200731000	-0.208981000
C	-1.684638000	1.303942000	0.985818000
C	-2.459439000	1.101887000	2.119290000
C	-0.895554000	2.441227000	1.009443000
C	-2.476545000	1.964037000	3.199445000
C	-0.877171000	3.332469000	2.072566000
C	-1.674227000	3.092358000	3.175017000
C	-3.018736000	-0.039695000	-1.038694000
C	-3.084043000	-1.054927000	-1.987364000
C	-4.168942000	0.732673000	-0.945597000
C	-4.196062000	-1.318038000	-2.766257000
C	-5.307396000	0.503257000	-1.704010000
C	-5.324017000	-0.530852000	-2.619637000
F	-3.258071000	0.024194000	2.190482000
F	-3.251119000	1.724163000	4.260614000
F	-1.671191000	3.937551000	4.205965000
F	-0.094730000	4.415241000	2.042093000
F	-0.085974000	2.734095000	-0.023007000
F	-4.239500000	1.771878000	-0.098058000
F	-6.385970000	1.277219000	-1.560600000
F	-6.407637000	-0.760392000	-3.359144000
F	-4.189891000	-2.311848000	-3.657816000
F	-2.014143000	-1.844962000	-2.197636000
C	-0.741238000	-3.316008000	1.349725000
C	0.659801000	-3.259486000	1.419328000
C	1.307034000	-2.114110000	0.888803000

C	-0.882893000	-1.118612000	0.350771000
C	-1.481241000	-2.294742000	0.813717000
C	3.540054000	-1.088448000	0.187874000
C	4.950233000	-1.090187000	0.328205000
C	5.644502000	-0.086957000	-0.324768000
C	3.659571000	0.729532000	-1.255550000
O	3.103668000	1.556221000	-2.039498000
H	6.716545000	-0.026924000	-0.218235000
N	5.047535000	0.828822000	-1.089352000
C	2.757632000	-2.083748000	0.885566000
C	5.624243000	-2.092337000	1.089149000
C	3.464687000	-3.103182000	1.547506000
C	1.412826000	-4.302864000	2.030998000
C	2.761294000	-4.204539000	2.122599000
C	4.896542000	-3.079002000	1.654232000
H	6.703759000	-2.053667000	1.179743000
H	5.379334000	-3.875795000	2.208429000
H	3.334944000	-4.981363000	2.615267000
H	0.894940000	-5.161342000	2.440491000
H	1.907765000	1.491588000	-2.199365000
N	2.963410000	-0.189595000	-0.621439000
C	0.496276000	-1.061210000	0.423222000
H	0.977560000	-0.159535000	0.087356000
H	-0.841827000	0.677684000	-1.073977000
C	0.009206000	0.623483000	-2.471116000
H	0.397328000	-0.363599000	-2.208813000
O	0.775222000	1.612101000	-2.539053000
H	-0.938021000	0.682886000	-3.008849000
C	5.849096000	1.935216000	-1.774099000
C	5.719459000	1.766513000	-3.290524000
C	5.324967000	3.286940000	-1.280758000
C	7.326555000	1.826453000	-1.398200000
H	6.054593000	0.770236000	-3.591942000
H	4.699196000	1.919887000	-3.634072000
H	6.364427000	2.503151000	-3.774713000
H	5.428416000	3.360523000	-0.194799000
H	5.929506000	4.075621000	-1.734083000
H	4.285049000	3.451033000	-1.553229000
H	7.840847000	2.652457000	-1.891724000
H	7.493748000	1.934155000	-0.323596000
H	7.785014000	0.899698000	-1.752588000
F	-2.816050000	-2.455751000	0.759366000
F	-1.373616000	-4.403364000	1.826336000

M1

	Coordinates (Angstroms)		
	X	Y	Z
B	-2.035188000	-0.117715000	0.234905000
C	-3.584052000	-0.222273000	-0.005823000
C	-4.383429000	-1.045214000	0.778288000
C	-4.222710000	0.459159000	-1.034930000
C	-5.744989000	-1.177421000	0.572237000
C	-5.576795000	0.334739000	-1.282175000
C	-6.340959000	-0.486434000	-0.468913000
C	-1.218794000	-1.458000000	0.387630000
C	-1.311343000	-2.453236000	-0.577131000
C	-0.325396000	-1.691760000	1.427271000

C	-0.547716000	-3.605321000	-0.537313000
C	0.443702000	-2.836853000	1.502667000
C	0.333828000	-3.794148000	0.510451000
F	-3.848160000	-1.725953000	1.797242000
F	-6.482066000	-1.958769000	1.358139000
F	-7.643214000	-0.609503000	-0.687649000
F	-6.151636000	0.990523000	-2.287516000
F	-3.518315000	1.251542000	-1.848203000
F	-0.191680000	-0.793077000	2.409922000
F	1.310776000	-3.014586000	2.498452000
F	1.092607000	-4.880869000	0.553064000
F	-0.637823000	-4.518473000	-1.502347000
F	-2.132636000	-2.296773000	-1.623329000
C	-1.075694000	3.629104000	0.435883000
C	0.318232000	3.584273000	0.226177000
C	0.947003000	2.321590000	0.030150000
C	-1.253707000	1.224479000	0.254988000
C	-1.828611000	2.489447000	0.457538000
C	3.139291000	1.003992000	-0.303152000
C	4.558290000	1.070981000	-0.337129000
C	5.236231000	-0.137239000	-0.389837000
C	3.183488000	-1.331440000	-0.580394000
O	2.615003000	-2.389476000	-0.794402000
H	6.316169000	-0.136095000	-0.358358000
N	4.618238000	-1.307965000	-0.461686000
C	5.388691000	-2.615156000	-0.442382000
C	2.389084000	2.247513000	-0.127065000
C	5.262079000	2.307178000	-0.279530000
C	3.140041000	3.432030000	-0.085403000
C	1.119749000	4.756376000	0.220617000
C	2.460531000	4.669291000	0.072021000
C	4.570879000	3.461296000	-0.175313000
H	6.345653000	2.301886000	-0.323917000
H	5.077635000	4.415430000	-0.136063000
N	2.524699000	-0.162563000	-0.430364000
C	0.115503000	1.183194000	0.043551000
H	0.590912000	0.232299000	-0.127939000
C	4.859490000	-3.469356000	0.715462000
C	5.219862000	-3.306285000	-1.798691000
C	6.877504000	-2.358849000	-0.206437000
H	5.821314000	-4.218631000	-1.805425000
H	4.181112000	-3.568047000	-1.986112000
H	5.576900000	-2.655329000	-2.601869000
H	4.981885000	-2.940740000	1.665107000
H	3.811141000	-3.725809000	0.583050000
H	5.442328000	-4.392207000	0.760531000
H	7.366829000	-3.332633000	-0.150368000
H	7.345015000	-1.808193000	-1.026716000
H	7.067429000	-1.840897000	0.737560000
F	-3.139042000	2.617205000	0.699180000
F	-1.693166000	4.798045000	0.641648000
F	0.570069000	5.961216000	0.370946000
F	3.179322000	5.796487000	0.084417000

M1_CO

	Coordinates (Angstroms)		
	X	Y	Z
B	-1.714471000	-0.370024000	-0.111887000
C	-2.505150000	-1.124636000	1.101859000
C	-3.329822000	-2.207748000	0.820323000
C	-2.432968000	-0.762781000	2.440158000
C	-4.066259000	-2.879879000	1.775625000
C	-3.157870000	-1.411464000	3.428457000
C	-3.978680000	-2.472731000	3.095763000
C	-2.647492000	0.094361000	-1.368461000
C	-3.983476000	0.453669000	-1.257502000
C	-2.090531000	0.247997000	-2.629910000
C	-4.720465000	0.927929000	-2.330774000
C	-2.791123000	0.715730000	-3.725889000
C	-4.122657000	1.057825000	-3.571412000
F	-3.428047000	-2.642350000	-0.448252000
F	-4.845336000	-3.906662000	1.443958000
F	-4.674207000	-3.100787000	4.035945000
F	-3.065612000	-1.020697000	4.698030000
F	-1.660448000	0.248670000	2.846351000
F	-0.796955000	-0.073447000	-2.819000000
F	-2.202928000	0.838580000	-4.913726000
F	-4.820413000	1.512698000	-4.606132000
F	-5.999501000	1.264919000	-2.175499000
F	-4.623196000	0.370923000	-0.086321000
C	-0.316389000	3.097821000	0.918127000
C	1.083036000	2.948435000	0.869009000
C	1.626771000	1.677884000	0.523570000
C	-0.659700000	0.792392000	0.280149000
C	-1.147632000	2.054722000	0.627531000
C	3.747476000	0.260217000	0.125409000
C	5.168575000	0.247703000	0.077897000
C	5.772131000	-0.948482000	-0.271592000
C	3.650300000	-2.023231000	-0.448660000
O	3.011154000	-3.040778000	-0.657042000
H	6.849412000	-0.992082000	-0.334757000
N	5.088099000	-2.052849000	-0.540561000
C	5.789273000	-3.340653000	-0.929569000
C	3.070177000	1.511929000	0.464917000
C	5.949198000	1.402651000	0.367028000
C	3.898123000	2.612234000	0.746849000
C	1.965148000	4.024740000	1.153443000
C	3.303006000	3.852899000	1.091308000
C	5.330926000	2.554152000	0.698504000
H	7.030374000	1.336599000	0.316440000
H	5.896898000	3.446749000	0.926178000
N	3.066220000	-0.845983000	-0.132729000
C	0.708854000	0.636342000	0.241681000
H	1.144451000	-0.317357000	-0.013018000
C	5.493812000	-4.399422000	0.137568000
C	5.300669000	-3.764071000	-2.318926000
C	7.302780000	-3.133610000	-0.997449000
H	5.843822000	-4.662718000	-2.620595000
H	4.234871000	-3.980444000	-2.321350000
H	5.509198000	-2.977618000	-3.049892000
H	5.845479000	-4.058897000	1.115712000
H	4.430686000	-4.621454000	0.197523000

H	6.031670000	-5.315438000	-0.118494000
H	7.745218000	-4.084998000	-1.297545000
H	7.589833000	-2.388986000	-1.744503000
H	7.735254000	-2.865105000	-0.030051000
F	-2.472017000	2.261959000	0.709252000
F	-0.867500000	4.270887000	1.252174000
C	-0.819850000	-1.600275000	-0.687556000
O	-0.252657000	-2.499714000	-1.026923000
F	1.490423000	5.226817000	1.482335000
F	4.098609000	4.896459000	1.362405000

M2

	Coordinates (Angstroms)		
	X	Y	Z
B	-1.683259000	-0.361538000	-0.123216000
C	-2.738208000	0.010862000	-1.309127000
C	-4.099780000	0.206966000	-1.122034000
C	-2.287596000	0.229448000	-2.603825000
C	-4.957274000	0.576986000	-2.145809000
C	-3.106872000	0.595302000	-3.655797000
C	-4.459131000	0.768058000	-3.421497000
C	-2.346854000	-1.100204000	1.162626000
C	-2.413858000	-0.570801000	2.442938000
C	-2.927729000	-2.352023000	1.018113000
C	-3.022456000	-1.231891000	3.499860000
C	-3.538847000	-3.049857000	2.040419000
C	-3.586363000	-2.477651000	3.299769000
F	-4.655214000	0.067838000	0.088693000
F	-6.257043000	0.753308000	-1.910831000
F	-5.271533000	1.120469000	-4.412636000
F	-2.611622000	0.783226000	-4.878049000
F	-0.974306000	0.091350000	-2.885440000
F	-2.917526000	-2.946565000	-0.199084000
F	-4.076208000	-4.249844000	1.829615000
F	-4.166706000	-3.122011000	4.306644000
F	-3.064991000	-0.674992000	4.709553000
F	-1.902315000	0.632996000	2.724809000
C	-0.282432000	3.195712000	0.643910000
C	1.112896000	3.010140000	0.676563000
C	1.633021000	1.710081000	0.422332000
C	-0.665428000	0.851075000	0.181977000
C	-1.128763000	2.153994000	0.387828000
C	3.720164000	0.255753000	0.021059000
C	5.135106000	0.166164000	0.122536000
C	5.708393000	-1.046875000	-0.218594000
C	3.580163000	-1.938196000	-0.822857000
O	2.929003000	-2.858317000	-1.292091000
H	6.777773000	-1.166818000	-0.127173000
N	5.003028000	-2.083486000	-0.654829000
C	3.070439000	1.505770000	0.410522000
C	5.935831000	1.267229000	0.542246000
C	3.917849000	2.571015000	0.756636000
C	2.014981000	4.064950000	0.979103000
C	3.345654000	3.838050000	1.040134000
C	5.345234000	2.444481000	0.833122000
H	7.011108000	1.144574000	0.608306000
H	5.930321000	3.302920000	1.132643000

O	-0.715674000	-1.537545000	-0.725594000
H	-0.118357000	-1.251077000	-1.435199000
C	0.699782000	0.666260000	0.220496000
H	1.109687000	-0.318778000	0.082997000
N	3.020167000	-0.769602000	-0.442158000
C	5.670595000	-3.401922000	-0.997760000
C	5.058092000	-4.499491000	-0.121601000
C	5.476643000	-3.676459000	-2.492400000
C	7.170777000	-3.340261000	-0.707922000
H	5.198355000	-4.262638000	0.936908000
H	3.996978000	-4.629613000	-0.321609000
H	5.572113000	-5.440811000	-0.330110000
H	5.890554000	-2.855809000	-3.085177000
H	6.014545000	-4.591158000	-2.753232000
H	4.425545000	-3.803010000	-2.741177000
H	7.586187000	-4.320614000	-0.947210000
H	7.689799000	-2.606523000	-1.330248000
H	7.385602000	-3.142408000	0.345565000
H	-1.198239000	-2.322244000	-1.030204000
F	-2.448252000	2.410485000	0.384802000
F	-0.814444000	4.403972000	0.871035000
F	1.564914000	5.295740000	1.228377000
F	4.158706000	4.855607000	1.357355000

M3

	Coordinates (Angstroms)		
	X	Y	Z
B	1.787678000	-0.448265000	-0.666323000
C	1.381754000	-1.472074000	0.594625000
C	1.890177000	-1.274598000	1.872598000
C	0.416738000	-2.460404000	0.525595000
C	1.492765000	-1.987781000	2.988151000
C	-0.027831000	-3.197515000	1.613212000
C	0.517451000	-2.962494000	2.858738000
C	3.419816000	-0.355776000	-0.924078000
C	3.924014000	0.664884000	-1.721514000
C	4.362756000	-1.292409000	-0.531640000
C	5.256290000	0.783742000	-2.073907000
C	5.709248000	-1.215061000	-0.859425000
C	6.161267000	-0.166777000	-1.635278000
F	2.825568000	-0.330081000	2.073563000
F	2.028271000	-1.744323000	4.186773000
F	0.110260000	-3.658012000	3.920725000
F	-0.978774000	-4.125970000	1.463443000
F	-0.190953000	-2.778370000	-0.652790000
F	4.011630000	-2.367807000	0.200633000
F	6.568914000	-2.150511000	-0.441785000
F	7.450289000	-0.074302000	-1.965515000
F	5.679851000	1.799644000	-2.833098000
F	3.104787000	1.619646000	-2.196612000
C	0.906789000	3.166755000	0.793210000
C	-0.487017000	3.198274000	0.630523000
C	-1.126467000	2.050915000	0.076703000
C	1.072396000	0.960430000	-0.196025000
C	1.646265000	2.072232000	0.417408000
C	-3.363754000	0.887526000	-0.382339000
C	-4.769444000	1.000200000	-0.521969000

C	-5.471685000	-0.143295000	-0.865660000
C	-3.518433000	-1.361360000	-0.857436000
O	-2.943755000	-2.532310000	-1.006051000
H	-6.541449000	-0.093497000	-0.994854000
N	-4.883078000	-1.329325000	-1.044926000
C	-2.577678000	2.040596000	-0.032381000
C	-5.445096000	2.232928000	-0.292236000
C	-3.305407000	3.209750000	0.264583000
C	-1.273945000	4.336160000	0.960761000
C	-2.610116000	4.344752000	0.750887000
C	-4.725974000	3.301598000	0.110343000
H	-6.518912000	2.286925000	-0.424535000
H	-5.210820000	4.244942000	0.321648000
O	1.140150000	-0.818946000	-1.924194000
H	-1.985518000	-2.435957000	-0.863187000
N	-2.804546000	-0.327724000	-0.548744000
C	-0.302733000	0.994851000	-0.350093000
H	-0.754104000	0.144841000	-0.828175000
C	-5.694161000	-2.581921000	-1.419357000
C	-5.182900000	-3.103946000	-2.764015000
C	-5.553447000	-3.602775000	-0.287857000
C	-7.168122000	-2.212456000	-1.566362000
H	-5.240178000	-2.321687000	-3.525076000
H	-4.162702000	-3.476766000	-2.709116000
H	-5.829610000	-3.927681000	-3.072762000
H	-5.893104000	-3.172693000	0.657711000
H	-6.192341000	-4.456462000	-0.523234000
H	-4.534209000	-3.964662000	-0.170817000
H	-7.696228000	-3.126855000	-1.839828000
H	-7.605031000	-1.850158000	-0.632761000
H	-7.338699000	-1.485213000	-2.363939000
H	1.415194000	-1.693910000	-2.198284000
F	2.969455000	2.141625000	0.641076000
F	1.552901000	4.215941000	1.322321000
F	-0.705428000	5.429170000	1.463615000
F	-3.307833000	5.451112000	1.044525000

M4

	Coordinates (Angstroms)		
	X	Y	Z
B	1.709231000	-0.316930000	0.063164000
C	2.449158000	-0.013112000	1.483199000
C	3.758910000	0.444175000	1.570197000
C	1.773444000	-0.058696000	2.693590000
C	4.352162000	0.814765000	2.766731000
C	2.324680000	0.291170000	3.911072000
C	3.633921000	0.735999000	3.945498000
C	2.670068000	-0.881205000	-1.100944000
C	2.713648000	-0.397063000	-2.400097000
C	3.516904000	-1.951822000	-0.838172000
C	3.557433000	-0.915925000	-3.369501000
C	4.366501000	-2.502206000	-1.778780000
C	4.388757000	-1.974562000	-3.057759000
F	4.520556000	0.563766000	0.477007000
F	5.611340000	1.246962000	2.789237000
F	4.194090000	1.083921000	5.098414000
F	1.615166000	0.207096000	5.033827000

F	0.481615000	-0.475535000	2.721223000
F	3.533155000	-2.507159000	0.384139000
F	5.159640000	-3.527845000	-1.468148000
F	5.198936000	-2.485993000	-3.979230000
F	3.564394000	-0.409326000	-4.602857000
F	1.918817000	0.605362000	-2.792465000
C	0.317382000	3.226573000	-0.728337000
C	-1.081869000	3.065281000	-0.703974000
C	-1.612718000	1.772348000	-0.438549000
C	0.677625000	0.877857000	-0.264375000
C	1.153968000	2.168350000	-0.510959000
C	-3.709172000	0.298662000	-0.134777000
C	-5.128696000	0.246319000	-0.159637000
C	-5.715365000	-0.983485000	0.086333000
C	-3.587506000	-1.992805000	0.397790000
O	-2.927354000	-2.992240000	0.665788000
H	-6.791928000	-1.063495000	0.059624000
N	-5.016893000	-2.081255000	0.351607000
C	-3.052007000	1.575945000	-0.407134000
C	-5.927294000	1.394860000	-0.429198000
C	-3.895092000	2.672938000	-0.655864000
C	-1.978631000	4.141693000	-0.935375000
C	-3.314862000	3.942121000	-0.912697000
C	-5.326390000	2.578512000	-0.665814000
H	-7.007178000	1.298035000	-0.436754000
H	-5.906049000	3.468097000	-0.869708000
O	0.722259000	-1.659765000	0.406660000
H	-0.905444000	-2.885803000	0.047069000
N	-3.015998000	-0.801500000	0.137962000
C	-0.689135000	0.725529000	-0.223024000
H	-1.124810000	-0.229906000	-0.001974000
C	-0.086256000	-2.388401000	-0.492236000
O	0.149312000	-2.393109000	-1.645874000
C	-5.702062000	-3.413059000	0.607168000
C	-5.221288000	-4.417590000	-0.444982000
C	-5.374923000	-3.862564000	2.034489000
C	-7.219302000	-3.272310000	0.480894000
H	-5.433683000	-4.044055000	-1.450638000
H	-4.155546000	-4.614091000	-0.354901000
H	-5.764618000	-5.355216000	-0.306575000
H	-5.718871000	-3.115819000	2.755702000
H	-5.901591000	-4.798622000	2.235067000
H	-4.308194000	-4.026109000	2.170014000
H	-7.650918000	-4.257141000	0.666753000
H	-7.640500000	-2.586792000	1.221164000
H	-7.528978000	-2.963423000	-0.521140000
H	0.341103000	-1.663267000	1.303845000
F	2.478843000	2.392145000	-0.549481000
F	0.860655000	4.427028000	-0.966943000
F	-1.519774000	5.368852000	-1.183071000
F	-4.124591000	4.985470000	-1.141829000

M5

	Coordinates (Angstroms)		
	X	Y	Z
B	1.736658000	-0.222793000	0.168807000
C	2.826219000	0.321216000	1.240236000

C	4.162586000	0.531038000	0.929846000
C	2.449237000	0.681291000	2.526398000
C	5.070465000	1.052596000	1.836904000
C	3.325383000	1.197848000	3.463212000
C	4.650533000	1.384949000	3.112040000
C	2.310380000	-1.272575000	-0.914311000
C	2.237019000	-1.103641000	-2.288682000
C	2.935078000	-2.438331000	-0.486853000
C	2.755633000	-2.024338000	-3.186734000
C	3.458437000	-3.381939000	-1.349570000
C	3.369165000	-3.169033000	-2.714539000
F	4.635512000	0.248580000	-0.290242000
F	6.343887000	1.238357000	1.490571000
F	5.512727000	1.882796000	3.992633000
F	2.908489000	1.516446000	4.687984000
F	1.170555000	0.520147000	2.908087000
F	3.037101000	-2.688310000	0.826224000
F	4.042770000	-4.485849000	-0.886032000
F	3.869172000	-4.060420000	-3.563973000
F	2.666873000	-1.813264000	-4.499857000
F	1.661731000	-0.019465000	-2.822453000
C	0.307921000	3.120828000	-1.214043000
C	-1.085507000	2.981026000	-1.063690000
C	-1.597166000	1.754779000	-0.551651000
C	0.705005000	0.911523000	-0.314235000
C	1.161658000	2.116618000	-0.852229000
C	-3.678434000	0.358257000	0.033983000
C	-5.087989000	0.340938000	0.203341000
C	-5.648413000	-0.846610000	0.641835000
C	-3.525216000	-1.894988000	0.672033000
O	-2.843235000	-2.905381000	0.867159000
H	-6.715261000	-0.899866000	0.797930000
N	-4.933436000	-1.941772000	0.884771000
C	-3.033445000	1.589798000	-0.404105000
C	-5.895396000	1.480554000	-0.080137000
C	-3.886280000	2.664160000	-0.706702000
C	-1.993806000	4.023943000	-1.390785000
C	-3.322830000	3.868467000	-1.200702000
C	-5.310453000	2.606477000	-0.537669000
H	-6.967946000	1.419990000	0.065939000
H	-5.898635000	3.481808000	-0.776500000
N	-2.975616000	-0.743715000	0.261989000
C	-0.658350000	0.763955000	-0.181622000
H	-1.049285000	-0.155017000	0.217359000
O	-0.251783000	-2.597343000	0.042003000
H	-1.163529000	-2.674155000	0.405244000
H	0.697020000	-1.375564000	0.611319000
H	1.019038000	-0.867461000	1.175732000
C	-5.591959000	-3.227723000	1.358986000
C	-5.355584000	-4.312372000	0.303198000
C	-5.003328000	-3.602021000	2.722530000
C	-7.099599000	-3.032065000	1.520240000
H	-5.772711000	-4.001652000	-0.658864000
H	-4.297205000	-4.531570000	0.180817000
H	-5.869067000	-5.223479000	0.618911000
H	-5.166959000	-2.794680000	3.441893000
H	-5.515643000	-4.494403000	3.089395000

H	-3.938386000	-3.813117000	2.658005000
H	-7.510504000	-3.979726000	1.872097000
H	-7.347021000	-2.272173000	2.266087000
H	-7.595403000	-2.793133000	0.575567000
H	-0.371306000	-2.508490000	-0.908632000
F	2.477787000	2.312884000	-1.037467000
F	0.830850000	4.248607000	-1.711849000
F	-1.550926000	5.184378000	-1.875165000
F	-4.140170000	4.887235000	-1.500248000

M6

	Coordinates (Angstroms)		
	X	Y	Z
B	1.708979000	-0.300068000	0.281809000
C	2.873472000	0.329179000	1.243187000
C	4.197882000	0.537858000	0.887412000
C	2.552325000	0.717492000	2.537220000
C	5.144195000	1.073884000	1.746885000
C	3.463563000	1.257772000	3.428676000
C	4.775451000	1.435731000	3.029047000
C	2.300890000	-1.329380000	-0.843518000
C	2.255667000	-1.174775000	-2.220693000
C	2.885352000	-2.516353000	-0.415599000
C	2.750551000	-2.115067000	-3.113242000
C	3.393129000	-3.480285000	-1.267796000
C	3.325034000	-3.276500000	-2.634611000
F	4.634532000	0.228434000	-0.345421000
F	6.408224000	1.248698000	1.348390000
F	5.672773000	1.954195000	3.868731000
F	3.092119000	1.607585000	4.664468000
F	1.291875000	0.578996000	2.988322000
F	2.974896000	-2.776689000	0.900913000
F	3.941936000	-4.600859000	-0.790762000
F	3.806551000	-4.190159000	-3.477913000
F	2.678410000	-1.906555000	-4.431392000
F	1.721367000	-0.074047000	-2.778117000
C	0.363654000	3.053678000	-1.270864000
C	-1.030415000	2.948730000	-1.112834000
C	-1.563818000	1.747777000	-0.561718000
C	0.728792000	0.868335000	-0.282737000
C	1.196694000	2.044579000	-0.873246000
C	-3.694665000	0.434681000	0.019423000
C	-5.095581000	0.460893000	0.230071000
C	-5.693165000	-0.704553000	0.679497000
C	-3.661140000	-1.801296000	0.598062000
O	-3.012256000	-2.916783000	0.743447000
H	-6.753337000	-0.715220000	0.877853000
N	-5.015298000	-1.836360000	0.882602000
C	-3.006508000	1.628656000	-0.407891000
C	-5.870205000	1.629261000	-0.023284000
C	-3.831299000	2.731722000	-0.707067000
C	-1.913116000	4.008642000	-1.455490000
C	-3.242917000	3.906762000	-1.233328000
C	-5.249465000	2.726254000	-0.502743000
H	-6.938790000	1.610428000	0.154474000
H	-5.808031000	3.623452000	-0.732271000
N	-3.043883000	-0.727580000	0.201541000

C	-0.642388000	0.759524000	-0.147318000
H	-1.030054000	-0.133871000	0.307514000
O	-0.536741000	-2.840886000	0.062525000
H	-2.042241000	-2.808166000	0.480021000
H	0.050610000	-2.222469000	0.528102000
H	1.014665000	-0.977238000	1.028907000
C	-5.712056000	-3.107501000	1.392566000
C	-5.583540000	-4.194176000	0.322325000
C	-5.070977000	-3.502228000	2.724938000
C	-7.195449000	-2.827749000	1.627164000
H	-6.006554000	-3.848146000	-0.624349000
H	-4.553334000	-4.503582000	0.163395000
H	-6.154953000	-5.064056000	0.652790000
H	-5.152762000	-2.684450000	3.445640000
H	-5.614564000	-4.362064000	3.121804000
H	-4.025218000	-3.781317000	2.617564000
H	-7.635816000	-3.754139000	1.998435000
H	-7.363173000	-2.060167000	2.386875000
H	-7.723640000	-2.561684000	0.708026000
H	-0.383943000	-2.659410000	-0.871411000
F	2.512940000	2.221204000	-1.074206000
F	0.907265000	4.155543000	-1.806969000
F	-1.444865000	5.139499000	-1.978287000
F	-4.033266000	4.945497000	-1.537109000

M7

	Coordinates (Angstroms)		
	X	Y	Z
B	1.871204000	-0.465698000	-0.624372000
C	1.395273000	-1.529601000	0.528128000
C	1.833648000	-1.450937000	1.843456000
C	0.405728000	-2.470586000	0.315520000
C	1.351149000	-2.248173000	2.865242000
C	-0.123622000	-3.284831000	1.304142000
C	0.357890000	-3.175230000	2.593587000
C	3.476114000	-0.392088000	-0.913946000
C	3.968404000	0.601908000	-1.752213000
C	4.434359000	-1.297135000	-0.482797000
C	5.297130000	0.726054000	-2.113816000
C	5.778599000	-1.212667000	-0.817939000
C	6.216334000	-0.192251000	-1.638061000
F	2.774325000	-0.550606000	2.173745000
F	1.818933000	-2.129954000	4.110156000
F	-0.130293000	-3.945762000	3.565743000
F	-1.096857000	-4.157825000	1.021954000
F	-0.147181000	-2.628785000	-0.919989000
F	4.101355000	-2.342644000	0.297678000
F	6.652059000	-2.115766000	-0.360028000
F	7.504177000	-0.096006000	-1.973866000
F	5.701669000	1.714338000	-2.918250000
F	3.126243000	1.518491000	-2.265686000
C	1.009217000	3.157999000	0.774033000
C	-0.385970000	3.202004000	0.624602000
C	-1.044344000	2.056146000	0.087696000
C	1.143573000	0.934434000	-0.185517000
C	1.732785000	2.051469000	0.405131000
C	-3.303636000	0.930199000	-0.383438000

C	-4.711464000	1.058872000	-0.484632000
C	-5.435167000	-0.068715000	-0.836464000
C	-3.494094000	-1.299802000	-0.936175000
O	-2.934573000	-2.466716000	-1.156635000
H	-6.507737000	-0.004564000	-0.930215000
N	-4.864400000	-1.253971000	-1.067904000
C	-2.498023000	2.064556000	-0.013684000
C	-5.370274000	2.292110000	-0.214871000
C	-3.208645000	3.239682000	0.305101000
C	-1.155378000	4.349172000	0.961512000
C	-2.495268000	4.367558000	0.780671000
C	-4.631040000	3.348316000	0.182803000
H	-6.446488000	2.357642000	-0.319140000
H	-5.101565000	4.293589000	0.416171000
H	-1.970626000	-2.381296000	-1.055801000
N	-2.761708000	-0.282795000	-0.614624000
C	-0.234292000	0.984015000	-0.326898000
H	-0.710317000	0.129295000	-0.773275000
H	1.358005000	-0.797660000	-1.675500000
C	-5.700742000	-2.490869000	-1.439539000
C	-5.256255000	-2.980316000	-2.819586000
C	-5.516826000	-3.541743000	-0.342138000
C	-7.176957000	-2.107918000	-1.508872000
H	-5.333618000	-2.175092000	-3.554634000
H	-4.240977000	-3.370087000	-2.820615000
H	-5.929469000	-3.784152000	-3.124148000
H	-5.812699000	-3.134478000	0.627974000
H	-6.170542000	-4.385732000	-0.571549000
H	-4.495741000	-3.911644000	-0.278252000
H	-7.724353000	-3.013739000	-1.772312000
H	-7.566707000	-1.759404000	-0.549447000
H	-7.380225000	-1.365747000	-2.284834000
F	3.057782000	2.100636000	0.624282000
F	1.670587000	4.202764000	1.291522000
F	-0.567950000	5.439438000	1.447749000
F	-3.177389000	5.479777000	1.088545000

M8

	Coordinates (Angstroms)		
	X	Y	Z
B	1.691838000	-0.365529000	0.190070000
C	2.530495000	0.386346000	1.386188000
C	3.853717000	0.797775000	1.294767000
C	1.891656000	0.763210000	2.559030000
C	4.499445000	1.507543000	2.295084000
C	2.494691000	1.466069000	3.585453000
C	3.818443000	1.841756000	3.450445000
C	2.593378000	-1.227576000	-0.844470000
C	2.463733000	-1.185750000	-2.225346000
C	3.557906000	-2.111062000	-0.370883000
C	3.251270000	-1.941821000	-3.081605000
C	4.357409000	-2.885734000	-1.188546000
C	4.203276000	-2.796055000	-2.561372000
F	4.586167000	0.530981000	0.205113000
F	5.773718000	1.871832000	2.151144000
F	4.427162000	2.519873000	4.419123000
F	1.817438000	1.785791000	4.687906000

F	0.585271000	0.452395000	2.742806000
F	3.738479000	-2.253840000	0.951959000
F	5.265107000	-3.715704000	-0.673815000
F	4.961064000	-3.530597000	-3.369601000
F	3.090211000	-1.855592000	-4.402345000
F	1.555581000	-0.399653000	-2.814908000
C	0.368178000	2.922968000	-1.428504000
C	-1.031821000	2.832670000	-1.304152000
C	-1.589893000	1.638265000	-0.767625000
C	0.679505000	0.732154000	-0.439786000
C	1.179431000	1.908499000	-1.006006000
C	-3.726767000	0.334825000	-0.173540000
C	-5.137062000	0.369639000	-0.013906000
C	-5.739102000	-0.785656000	0.453858000
C	-3.660979000	-1.915122000	0.484059000
O	-3.014583000	-2.956649000	0.650972000
H	-6.806598000	-0.794404000	0.615066000
N	-5.064111000	-1.903000000	0.717039000
C	-3.032836000	1.529464000	-0.634313000
C	-5.900866000	1.530744000	-0.331923000
C	-3.844535000	2.624058000	-0.975408000
C	-1.898848000	3.894356000	-1.677023000
C	-3.235118000	3.791879000	-1.499428000
C	-5.271602000	2.621193000	-0.815649000
H	-6.975825000	1.513967000	-0.192194000
H	-5.825462000	3.510648000	-1.083161000
O	0.820198000	-1.480176000	0.964482000
N	-3.066221000	-0.786558000	0.081372000
C	-0.690162000	0.627562000	-0.352340000
H	-1.124929000	-0.272268000	0.040013000
C	0.376265000	-2.822994000	0.485362000
H	0.035389000	-3.309778000	1.401109000
H	1.288739000	-3.277292000	0.111511000
O	-0.553419000	-2.751338000	-0.488391000
H	-1.453863000	-2.761618000	-0.090227000
C	-5.766563000	-3.152844000	1.224023000
C	-5.608023000	-4.257990000	0.175365000
C	-5.154793000	-3.538478000	2.574112000
C	-7.258174000	-2.887465000	1.427611000
H	-6.031799000	-3.936946000	-0.780210000
H	-4.564841000	-4.529289000	0.030625000
H	-6.157001000	-5.140187000	0.513007000
H	-5.264524000	-2.719252000	3.290341000
H	-5.691418000	-4.406300000	2.964199000
H	-4.101665000	-3.793999000	2.480965000
H	-7.699168000	-3.810285000	1.807926000
H	-7.447204000	-2.105516000	2.167942000
H	-7.772520000	-2.641102000	0.495075000
H	0.195406000	-1.111150000	1.604489000
F	2.507020000	2.065980000	-1.155426000
F	0.937636000	4.016889000	-1.952179000
F	-1.408943000	5.023559000	-2.189771000
F	-4.013364000	4.829204000	-1.839275000

TSM2-3

	Coordinates (Angstroms)		
	X	Y	Z
B	1.432894000	-0.142740000	-0.256872000
C	1.789868000	-0.495271000	1.308998000
C	3.028764000	-0.324813000	1.910850000
C	0.791558000	-0.954020000	2.159786000
C	3.278684000	-0.618813000	3.242226000
C	0.997552000	-1.261750000	3.493464000
C	2.256614000	-1.096286000	4.040244000
C	2.755935000	-0.086688000	-1.224081000
C	3.191803000	1.037552000	-1.910926000
C	3.553848000	-1.210308000	-1.395997000
C	4.334441000	1.050378000	-2.698695000
C	4.697085000	-1.246662000	-2.169700000
C	5.093101000	-0.096352000	-2.829180000
F	4.071730000	0.163076000	1.220121000
F	4.495609000	-0.439824000	3.761169000
F	2.478585000	-1.384453000	5.321224000
F	-0.005211000	-1.706847000	4.255113000
F	-0.464724000	-1.105997000	1.709039000
F	3.215937000	-2.360878000	-0.770482000
F	5.415229000	-2.364243000	-2.286100000
F	6.189110000	-0.097636000	-3.583479000
F	4.706461000	2.163586000	-3.332875000
F	2.530130000	2.200864000	-1.840587000
C	-0.268966000	3.337350000	0.620208000
C	-1.562708000	3.146177000	0.090314000
C	-1.785647000	1.953342000	-0.630886000
C	0.503418000	1.198017000	-0.271064000
C	0.680942000	2.348466000	0.516615000
C	-3.408257000	0.090358000	-0.892849000
C	-4.726863000	-0.407888000	-0.907580000
C	-4.836522000	-1.791413000	-0.874931000
C	-2.503604000	-2.039509000	-0.744014000
O	-1.440052000	-2.725908000	-0.615813000
H	-5.812158000	-2.251739000	-0.891766000
N	-3.775979000	-2.618714000	-0.798949000
C	-3.137760000	1.500343000	-0.813606000
C	-5.823538000	0.515602000	-0.862954000
C	-4.227264000	2.335963000	-0.574012000
C	-2.680779000	4.012162000	0.270478000
C	-3.938102000	3.645846000	-0.107140000
C	-5.575854000	1.837248000	-0.685759000
H	-6.839870000	0.144092000	-0.924660000
H	-6.395369000	2.537888000	-0.588498000
O	0.573409000	-1.349078000	-0.828924000
H	-0.458008000	-1.938030000	-0.624009000
N	-2.389797000	-0.742745000	-0.810975000
C	-0.685915000	1.131776000	-0.947647000
H	-0.816844000	0.333595000	-1.648247000
C	-3.945370000	-4.131962000	-0.731582000
C	-3.209413000	-4.746404000	-1.924910000
C	-3.385304000	-4.613978000	0.609558000
C	-5.422607000	-4.510523000	-0.813584000
H	-3.607714000	-4.351148000	-2.863189000
H	-2.138120000	-4.557934000	-1.882406000
H	-3.371228000	-5.826400000	-1.912063000

H	-3.907137000	-4.126495000	1.437515000
H	-3.551658000	-5.690588000	0.687409000
H	-2.317904000	-4.421083000	0.699414000
H	-5.477146000	-5.599585000	-0.771342000
H	-6.000636000	-4.122436000	0.028947000
H	-5.883609000	-4.195454000	-1.753242000
H	1.168570000	-2.037538000	-1.140064000
F	1.842253000	2.538019000	1.169136000
F	0.026559000	4.452052000	1.306638000
F	-2.511330000	5.205894000	0.840915000
F	-4.949993000	4.502946000	0.091060000

TSM3-4

	Coordinates (Angstroms)		
	X	Y	Z
B	1.619643000	-0.354952000	0.171303000
C	2.487863000	0.191658000	1.464476000
C	3.813013000	0.604200000	1.412021000
C	1.882981000	0.370387000	2.700159000
C	4.491655000	1.130307000	2.500360000
C	2.518137000	0.887426000	3.814655000
C	3.842464000	1.270930000	3.712492000
C	2.544794000	-1.037819000	-0.989715000
C	2.505404000	-0.730891000	-2.340148000
C	3.407786000	-2.073548000	-0.651362000
C	3.273224000	-1.384557000	-3.293032000
C	4.188173000	-2.752931000	-1.568050000
C	4.120158000	-2.403487000	-2.905354000
F	4.518834000	0.519056000	0.274758000
F	5.767486000	1.504155000	2.387937000
F	4.481647000	1.774063000	4.766324000
F	1.870153000	1.023992000	4.972740000
F	0.578656000	0.040796000	2.866045000
F	3.528373000	-2.457774000	0.630181000
F	5.004580000	-3.736224000	-1.180311000
F	4.862600000	-3.043996000	-3.806772000
F	3.191253000	-1.044584000	-4.581992000
F	1.690689000	0.225672000	-2.813829000
C	0.295552000	3.160099000	-1.063489000
C	-1.104858000	3.008307000	-0.997267000
C	-1.622404000	1.752731000	-0.580986000
C	0.672091000	0.885767000	-0.310557000
C	1.139721000	2.133386000	-0.732281000
C	-3.688632000	0.305135000	-0.144795000
C	-5.098759000	0.225340000	-0.047945000
C	-5.627912000	-0.991660000	0.350804000
C	-3.491323000	-1.936794000	0.464763000
O	-2.742502000	-2.938014000	0.675692000
H	-6.696233000	-1.095534000	0.461436000
N	-4.877016000	-2.063689000	0.619662000
C	-3.057813000	1.556251000	-0.510079000
C	-5.926555000	1.349374000	-0.343672000
C	-3.921301000	2.621664000	-0.813913000
C	-2.018554000	4.049460000	-1.314622000
C	-3.356154000	3.858245000	-1.216766000
C	-5.349819000	2.508425000	-0.727050000
H	-7.002780000	1.253559000	-0.260072000

H	-5.954220000	3.372685000	-0.967359000
O	0.721062000	-1.519204000	0.650574000
H	-1.544458000	-2.704429000	0.264513000
N	-2.964355000	-0.786650000	0.102090000
C	-0.692790000	0.750502000	-0.239977000
H	-1.093961000	-0.179137000	0.102391000
C	-0.407802000	-2.561576000	-0.379136000
O	0.092345000	-2.843229000	-1.366289000
C	-5.502156000	-3.381120000	1.074197000
C	-5.192330000	-4.450084000	0.023297000
C	-4.938335000	-3.729530000	2.454252000
C	-7.018602000	-3.234533000	1.190155000
H	-5.585327000	-4.149460000	-0.951657000
H	-4.124845000	-4.640273000	-0.064387000
H	-5.687405000	-5.377662000	0.318797000
H	-5.149075000	-2.926432000	3.165573000
H	-5.432726000	-4.636659000	2.808407000
H	-3.866073000	-3.910460000	2.425059000
H	-7.406450000	-4.194217000	1.534935000
H	-7.312580000	-2.480773000	1.925144000
H	-7.494114000	-3.018141000	0.230021000
H	0.252717000	-1.310416000	1.466305000
F	2.461716000	2.354956000	-0.835808000
F	0.831099000	4.324725000	-1.455955000
F	-1.574603000	5.242447000	-1.707601000
F	-4.176907000	4.875126000	-1.515305000

TSM1-7

	Coordinates (Angstroms)		
	X	Y	Z
B	1.463437000	-0.285547000	0.169039000
C	2.373554000	-0.636441000	-1.103411000
C	3.752260000	-0.779917000	-1.044181000
C	1.799160000	-0.863182000	-2.347906000
C	4.521321000	-1.114376000	-2.147160000
C	2.533185000	-1.192079000	-3.472890000
C	3.907693000	-1.318090000	-3.369211000
C	2.124035000	0.576928000	1.345541000
C	2.293279000	0.087324000	2.633485000
C	2.585284000	1.869556000	1.130354000
C	2.863521000	0.833022000	3.652723000
C	3.153323000	2.647319000	2.123080000
C	3.292029000	2.122227000	3.396238000
F	4.407683000	-0.618392000	0.113683000
F	5.844118000	-1.244777000	-2.040529000
F	4.632334000	-1.636220000	-4.437981000
F	1.934995000	-1.389140000	-4.648024000
F	0.470898000	-0.761198000	-2.497894000
F	2.492010000	2.417821000	-0.088931000
F	3.572697000	3.886790000	1.866827000
F	3.841014000	2.850689000	4.364060000
F	3.009020000	0.317496000	4.873955000
F	1.928278000	-1.163353000	2.943884000
C	-0.782298000	-3.524783000	0.526128000
C	-2.022467000	-2.925440000	0.827675000
C	-2.032407000	-1.524471000	0.998134000
C	0.358074000	-1.371923000	0.581203000

C	0.333835000	-2.748548000	0.306512000
C	-3.254125000	0.516282000	0.293474000
C	-4.455666000	1.184380000	-0.018990000
C	-4.302189000	2.419740000	-0.633350000
C	-1.951619000	2.182181000	-0.676433000
O	-0.799204000	2.556701000	-1.020390000
H	-5.174665000	3.001024000	-0.891689000
N	-3.106156000	2.940507000	-0.956792000
C	-3.271103000	-0.814726000	0.844046000
C	-5.708743000	0.529587000	0.220477000
C	-4.494669000	-1.478767000	0.865143000
C	-3.282393000	-3.589566000	0.912233000
C	-4.450590000	-2.894406000	0.999905000
C	-5.723208000	-0.763748000	0.629762000
H	-6.634732000	1.057816000	0.024121000
H	-6.662739000	-1.287602000	0.749831000
H	0.095899000	1.460784000	-0.650403000
N	-2.090026000	1.038917000	-0.044024000
C	-0.807769000	-0.841299000	1.075986000
H	-0.800929000	0.181293000	1.402130000
H	0.609926000	0.753632000	-0.463693000
C	-2.986782000	4.295466000	-1.642667000
C	-2.128740000	5.201686000	-0.755603000
C	-2.367363000	4.081469000	-3.026624000
C	-4.362973000	4.935218000	-1.814448000
H	-2.592937000	5.323040000	0.226919000
H	-1.121036000	4.809729000	-0.630711000
H	-2.061039000	6.184818000	-1.226354000
H	-2.990501000	3.409449000	-3.622985000
H	-2.318658000	5.045805000	-3.537176000
H	-1.361131000	3.672451000	-2.961638000
H	-4.208489000	5.905674000	-2.288887000
H	-5.016172000	4.352255000	-2.468608000
H	-4.864112000	5.112697000	-0.859318000
F	1.441287000	-3.364271000	-0.139948000
F	-0.691480000	-4.852113000	0.359312000
F	-3.333339000	-4.921899000	0.884170000
F	-5.603033000	-3.573483000	1.077057000

TSM5-6

	Coordinates (Angstroms)		
	X	Y	Z
B	1.728777000	-0.226829000	0.168960000
C	2.809512000	0.321052000	1.248414000
C	4.147030000	0.539533000	0.949256000
C	2.420830000	0.675156000	2.532880000
C	5.045644000	1.062670000	1.864719000
C	3.287424000	1.193580000	3.477682000
C	4.614230000	1.388666000	3.137652000
C	2.319442000	-1.263431000	-0.919221000
C	2.250002000	-1.091586000	-2.293408000
C	2.954107000	-2.424519000	-0.493527000
C	2.781740000	-2.003958000	-3.192443000
C	3.491129000	-3.359895000	-1.356922000
C	3.405405000	-3.143657000	-2.721576000
F	4.631276000	0.263161000	-0.268159000
F	6.320902000	1.256137000	1.528694000

F	5.467060000	1.888315000	4.026697000
F	2.859615000	1.506654000	4.700427000
F	1.140479000	0.507423000	2.905496000
F	3.053497000	-2.678204000	0.819319000
F	4.085229000	-4.459164000	-0.894373000
F	3.918250000	-4.027155000	-3.571828000
F	2.695434000	-1.789863000	-4.505366000
F	1.663769000	-0.012788000	-2.826865000
C	0.299611000	3.119027000	-1.218728000
C	-1.093637000	2.979188000	-1.065198000
C	-1.602512000	1.753557000	-0.550136000
C	0.700481000	0.910615000	-0.316447000
C	1.154739000	2.114936000	-0.858765000
C	-3.677913000	0.349691000	0.030918000
C	-5.087163000	0.324498000	0.199259000
C	-5.640930000	-0.867639000	0.633990000
C	-3.512630000	-1.904041000	0.662737000
O	-2.822209000	-2.910371000	0.855563000
H	-6.707479000	-0.928040000	0.789377000
N	-4.919655000	-1.959754000	0.874702000
C	-3.037896000	1.584498000	-0.403198000
C	-5.898980000	1.461844000	-0.081933000
C	-3.894250000	2.655906000	-0.705131000
C	-2.004956000	4.019948000	-1.390825000
C	-3.333700000	3.861844000	-1.198581000
C	-5.318331000	2.591390000	-0.536542000
H	-6.971377000	1.396731000	0.063252000
H	-5.910378000	3.464581000	-0.773813000
N	-2.969942000	-0.749644000	0.255532000
C	-0.662132000	0.765721000	-0.177700000
H	-1.052197000	-0.149561000	0.228814000
O	-0.245402000	-2.571832000	0.040315000
H	-1.159025000	-2.656338000	0.404637000
H	0.657169000	-1.408940000	0.587655000
H	1.016190000	-0.890677000	1.142197000
C	-5.571112000	-3.250281000	1.346545000
C	-5.328336000	-4.331647000	0.288926000
C	-4.980261000	-3.623373000	2.709425000
C	-7.079780000	-3.063052000	1.507849000
H	-5.746450000	-4.021424000	-0.672859000
H	-4.268734000	-4.545164000	0.166838000
H	-5.837211000	-5.246006000	0.602643000
H	-5.148153000	-2.817987000	3.429972000
H	-5.487685000	-4.519050000	3.075034000
H	-3.914194000	-3.828701000	2.644462000
H	-7.485614000	-4.013360000	1.858428000
H	-7.331517000	-2.305437000	2.254567000
H	-7.576646000	-2.825706000	0.563330000
H	-0.361905000	-2.481460000	-0.911117000
F	2.470215000	2.311516000	-1.048243000
F	0.820622000	4.246854000	-1.718807000
F	-1.565155000	5.181087000	-1.876312000
F	-4.153577000	4.878991000	-1.496982000

TSM7

	Coordinates (Angstroms)		
	X	Y	Z
B	1.753241000	-0.201015000	0.029465000
C	2.556194000	-0.109024000	1.433946000
C	3.890267000	0.249403000	1.567828000
C	1.879873000	-0.318359000	2.628795000
C	4.522939000	0.371847000	2.794058000
C	2.474922000	-0.210337000	3.873581000
C	3.811101000	0.137104000	3.956061000
C	2.616060000	-0.617567000	-1.277925000
C	2.491969000	-0.026153000	-2.531000000
C	3.532282000	-1.664980000	-1.222146000
C	3.227688000	-0.425235000	-3.637408000
C	4.281789000	-2.096221000	-2.300565000
C	4.129028000	-1.465987000	-3.522814000
F	4.642562000	0.502835000	0.486026000
F	5.810671000	0.715689000	2.864818000
F	4.405018000	0.248827000	5.142261000
F	1.775423000	-0.431263000	4.988493000
F	0.578240000	-0.642576000	2.611661000
F	3.721775000	-2.326180000	-0.065915000
F	5.141080000	-3.107429000	-2.177331000
F	4.836260000	-1.862989000	-4.575629000
F	3.070266000	0.186720000	-4.811155000
F	1.638683000	0.984332000	-2.740180000
C	0.304089000	3.398375000	-0.294878000
C	-1.090146000	3.201165000	-0.314445000
C	-1.594800000	1.871984000	-0.214718000
C	0.714909000	1.019919000	-0.119649000
C	1.162414000	2.340166000	-0.195502000
C	-3.673346000	0.358371000	-0.084246000
C	-5.087855000	0.271005000	-0.163299000
C	-5.646819000	-0.987362000	-0.025780000
C	-3.516103000	-1.948159000	0.269935000
O	-2.825218000	-2.962945000	0.476530000
H	-6.718539000	-1.099393000	-0.089278000
N	-4.921921000	-2.084431000	0.182599000
C	-3.033017000	1.657031000	-0.230653000
C	-5.905945000	1.421474000	-0.360791000
C	-3.894673000	2.756722000	-0.388998000
C	-2.005891000	4.279427000	-0.444621000
C	-3.337721000	4.056168000	-0.489958000
C	-5.322465000	2.632801000	-0.459483000
H	-6.981674000	1.303205000	-0.421004000
H	-5.914469000	3.527038000	-0.597783000
H	-1.349329000	-2.802697000	0.616722000
N	-2.962489000	-0.745578000	0.129842000
C	-0.648526000	0.824625000	-0.123187000
H	-1.028175000	-0.177205000	-0.045810000
H	0.957220000	-1.231569000	0.210394000
O	-0.107453000	-2.399642000	-1.326244000
C	0.449398000	-2.572348000	-0.133048000
H	1.445891000	-3.008556000	-0.084936000
O	-0.342254000	-2.897171000	0.825342000
C	-5.576636000	-3.450814000	0.335716000
C	-5.056779000	-4.368236000	-0.775344000
C	-5.260866000	-3.987371000	1.735080000

C	-7.094351000	-3.333857000	0.196038000
H	-5.263243000	-3.931129000	-1.756232000
H	-3.988530000	-4.550043000	-0.683028000
H	-5.581777000	-5.323839000	-0.709420000
H	-5.621682000	-3.291694000	2.497729000
H	-5.781019000	-4.938879000	1.867089000
H	-4.195215000	-4.151442000	1.876805000
H	-7.505307000	-4.337843000	0.312551000
H	-7.540429000	-2.708317000	0.973671000
H	-7.398888000	-2.968659000	-0.788197000
H	0.577124000	-2.236442000	-1.990036000
F	2.482564000	2.597104000	-0.199769000
F	0.821073000	4.632090000	-0.381573000
F	-1.569277000	5.535851000	-0.536245000
F	-4.163427000	5.103343000	-0.629035000

TSM8

	Coordinates (Angstroms)		
	X	Y	Z
B	1.660600000	-0.377093000	0.287491000
C	2.655347000	0.461895000	1.322051000
C	3.985775000	0.783968000	1.093292000
C	2.142641000	0.969176000	2.507326000
C	4.756462000	1.527655000	1.973408000
C	2.870260000	1.716211000	3.416942000
C	4.196672000	1.996425000	3.146909000
C	2.485456000	-1.325510000	-0.766781000
C	2.290735000	-1.363252000	-2.138426000
C	3.407737000	-2.248844000	-0.286448000
C	2.968965000	-2.232125000	-2.982327000
C	4.103410000	-3.133128000	-1.089756000
C	3.881555000	-3.123747000	-2.455830000
F	4.610692000	0.387583000	-0.027317000
F	6.034491000	1.798916000	1.696972000
F	4.923039000	2.713443000	4.003551000
F	2.307977000	2.169314000	4.540422000
F	0.843275000	0.760390000	2.826673000
F	3.670296000	-2.317696000	1.029940000
F	4.980797000	-3.993437000	-0.565835000
F	4.539201000	-3.966559000	-3.250785000
F	2.740851000	-2.217409000	-4.298290000
F	1.412397000	-0.546561000	-2.741697000
C	0.396998000	2.879236000	-1.524036000
C	-1.000625000	2.824681000	-1.362670000
C	-1.567686000	1.668364000	-0.755352000
C	0.693641000	0.747354000	-0.406518000
C	1.197705000	1.870227000	-1.066771000
C	-3.724452000	0.425716000	-0.135405000
C	-5.124248000	0.491645000	0.074829000
C	-5.736414000	-0.641155000	0.582919000
C	-3.717226000	-1.803682000	0.507480000
O	-3.095619000	-2.908439000	0.638004000
H	-6.794422000	-0.620968000	0.794934000
N	-5.076078000	-1.774779000	0.828919000
C	-3.011414000	1.592599000	-0.606585000
C	-5.871417000	1.667041000	-0.230784000
C	-3.808279000	2.697562000	-0.956926000

C	-1.854876000	3.890256000	-1.755524000
C	-3.187777000	3.832802000	-1.532653000
C	-5.228494000	2.729843000	-0.756680000
H	-6.940596000	1.680328000	-0.054446000
H	-5.768432000	3.627465000	-1.026055000
O	0.843543000	-1.355756000	1.086927000
N	-3.092207000	-0.732248000	0.078143000
C	-0.677032000	0.676597000	-0.283325000
H	-1.097276000	-0.188056000	0.196379000
C	0.019685000	-3.127171000	0.524896000
H	-0.207841000	-3.302581000	1.579241000
H	1.002476000	-3.426746000	0.167019000
O	-0.893934000	-2.968026000	-0.304917000
H	-2.017797000	-2.877879000	0.187241000
C	-5.781948000	-2.999262000	1.414214000
C	-5.752780000	-4.120717000	0.372890000
C	-5.076695000	-3.384587000	2.717559000
C	-7.237945000	-2.664570000	1.734415000
H	-6.222124000	-3.788736000	-0.556942000
H	-4.740487000	-4.457965000	0.161946000
H	-6.325841000	-4.965443000	0.761555000
H	-5.094684000	-2.549095000	3.422593000
H	-5.618705000	-4.219880000	3.166187000
H	-4.046892000	-3.693098000	2.553245000
H	-7.679401000	-3.558572000	2.177073000
H	-7.330492000	-1.857117000	2.465354000
H	-7.820075000	-2.423327000	0.841506000
H	0.305751000	-0.925537000	1.755972000
F	2.521725000	1.989159000	-1.273222000
F	0.973256000	3.932838000	-2.120153000
F	-1.354348000	4.984488000	-2.327304000
F	-3.950596000	4.877820000	-1.883822000

TSM9

	Coordinates (Angstroms)		
	X	Y	Z
B	1.763762000	-0.376233000	-0.182112000
C	1.806313000	-1.108533000	1.269814000
C	2.572721000	-0.601032000	2.309460000
C	1.015796000	-2.196129000	1.600216000
C	2.583128000	-1.134894000	3.584357000
C	0.990011000	-2.762109000	2.866620000
C	1.780794000	-2.227989000	3.865599000
C	3.159554000	-0.281980000	-1.005864000
C	3.226628000	0.470747000	-2.173339000
C	4.329639000	-0.961048000	-0.692912000
C	4.359411000	0.581736000	-2.959184000
C	5.488662000	-0.877334000	-1.449902000
C	5.506241000	-0.097907000	-2.590383000
F	3.366745000	0.459589000	2.088611000
F	3.349141000	-0.610689000	4.544444000
F	1.770450000	-2.757019000	5.089105000
F	0.207409000	-3.812768000	3.129034000
F	0.209803000	-2.761265000	0.683497000
F	4.399758000	-1.760483000	0.383826000
F	6.585247000	-1.548334000	-1.089971000
F	6.609811000	-0.009317000	-3.330755000

F	4.354916000	1.326573000	-4.067188000
F	2.138529000	1.136324000	-2.605277000
C	0.628310000	3.371995000	0.384132000
C	-0.771512000	3.249934000	0.428498000
C	-1.343118000	1.966704000	0.200339000
C	0.915544000	0.997578000	-0.009504000
C	1.432073000	2.284206000	0.152880000
C	-3.478574000	0.623093000	-0.238812000
C	-4.888563000	0.558856000	-0.119468000
C	-5.494228000	-0.633869000	-0.476636000
C	-3.432441000	-1.522248000	-1.133746000
O	-2.802044000	-2.484114000	-1.662087000
H	-6.562660000	-0.740361000	-0.369106000
N	-4.814202000	-1.677125000	-0.950638000
C	-2.787021000	1.824876000	0.176250000
C	-5.654747000	1.673209000	0.332261000
C	-3.595152000	2.918326000	0.530958000
C	-1.635607000	4.340550000	0.722875000
C	-2.974408000	4.164427000	0.803828000
C	-5.023190000	2.830134000	0.621745000
H	-6.730948000	1.578152000	0.416470000
H	-5.579631000	3.702760000	0.935714000
H	-1.603001000	-2.382558000	-1.846672000
N	-2.819619000	-0.414433000	-0.770120000
C	-0.459855000	0.883620000	0.040570000
H	-0.884907000	-0.098538000	-0.063000000
H	1.026043000	-1.109578000	-0.904566000
C	0.215277000	-1.469191000	-2.318321000
H	-0.252110000	-0.482175000	-2.302845000
O	-0.469211000	-2.501988000	-2.148502000
H	1.186614000	-1.581191000	-2.800876000
C	-5.517405000	-2.988870000	-1.301420000
C	-5.371330000	-3.235325000	-2.805398000
C	-4.902599000	-4.106981000	-0.455249000
C	-7.005400000	-2.893408000	-0.966392000
H	-5.775152000	-2.390411000	-3.369713000
H	-4.335954000	-3.398089000	-3.094803000
H	-5.948751000	-4.125452000	-3.064810000
H	-5.011520000	-3.882312000	0.609182000
H	-5.444099000	-5.032140000	-0.664484000
H	-3.850941000	-4.266087000	-0.681763000
H	-7.448483000	-3.861162000	-1.205837000
H	-7.185198000	-2.705773000	0.095257000
H	-7.524149000	-2.141229000	-1.566164000
F	2.756288000	2.503374000	0.102011000
F	1.211421000	4.564381000	0.571104000
F	-1.138491000	5.555050000	0.951817000
F	-3.747349000	5.212382000	1.122560000

Reference

(1) Lee, C.; Yang, W.; Parr, R. G. Development of the Colle-Salvetti correlation-energy formula into a functional of the electron density. *Phys. Rev. B: Condens. Matter Mater. Phys.* **1988**, *37*, 785-789.

(2) Becke, A. D. Density-functional thermochemistry. III. The role of exact exchange. *J. Chem. Phys.* **1993**, *98*, 5648-5652.

(3) Frisch, M. J.; Trucks, G. W.; Schlegel, H. B.; Scuseria, G. E.; Robb, M. A.; Cheeseman, J. R.; Scalmani, G.; Barone, V.; Mennucci, B.; Petersson, G. A.; Nakatsuji, H.; Caricato, M.; Li, X.; Hratchian, H. P.; Izmaylov, A. F.; Bloino, J.; Zheng, G.; Sonnenberg, J. L.; Hada, M.; Ehara, M.; Toyota, K.; Fukuda, R.; Hasegawa, J.; Ishida, M.; Nakajima, T.; Honda, Y.; Kitao, O.; Nakai, H.; Vreven, T.; Montgomery, J. A., Jr. ; Peralta, J. E.; Ogliaro, F.; Bearpark, M.; Heyd, J. J.; Brothers, E.; Kudin, K. N.; Staroverov, V. N.; Kobayashi, R.; Normand, J.; Raghavachari, K.; Rendell, A.; Burant, J. C.; Iyengar, S. S.; Tomasi, J.; Cossi, M.; Rega, N.; Millam, J. M.; Klene, M.; Knox, J. E.; Cross, J. B.; Bakken, V.; Adamo, C.; Jaramillo, J.; Gomperts, R.; Stratmann, R. E.; Yazyev, O.; Austin, A. J.; Cammi, R.; Pomelli, C.; Ochterski, J. W.; Martin, R. L.; Morokuma, K.; Zakrzewski, V. G.; Voth, G. A.; Salvador, P.; Dannenberg, J. J.; Dapprich, S.; Daniels, A. D.; Farkas, O.; Foresman, J. B.; Ortiz, J. V.; Cioslowski, J.; Fox, D. J. *Gaussian 09,* revision B.01; Gaussian, Inc.: Wallingford, CT, **2009**.

(4) Krishnan, R.; Binkley, J. S.; Seeger, R.; Pople, J. A. Self-consistent molecular orbital methods. XX. A basis set for correlated wave functions. *J. Chern. Phys.* **1980**, *72*, 650-654.

(5) Tomasi, J.; Persico, M. Molecular Interactions in Solution: An Overview of Methods Based on Continuous Distributions of the Solvent. *Chem. Rev.* **1994**, *94*, 2027-2094.

(6) Marenich, A. V.; Cramer, C. J.; Truhlar, D. G. Universal Solvation Model Based on Solute Electron Density and on a Continuum Model of the Solvent Defined by the Bulk Dielectric Constant and Atomic Surface Tensions. *J. Phys. Chem. B* **2009**, *113*, 6378-6396.

(7) Tsutsumi, T.; Ono, Y.; Arai, Z.; Taketsugu, T. Visualization of the Intrinsic Reaction Coordinate and Global Reaction Route Map by Classical Multidimensional Scaling. *J. Chem. Theory Comput.* **2018**, *14*, 4263-4270.

(8) Chai, J.-D.; Head-Gordon, M. Long-Range Corrected Hybrid Density Functionals with Damped Atom-Atom Dispersion Corrections. *Phys. Chem. Chem. Phys.* **2008**, *10*, 6615-6620.
